# Morphological Diversity as a Proxy for Assessing Genetic Diversity of *Aedes aegypti* (Diptera, Culicidae)

**DOI:** 10.3390/insects17050469

**Published:** 2026-04-30

**Authors:** Fernanda Almeida Lopes, Camila Moratore, Karina Ramos dos Santos, Lucas Fujimori Tani, Marília Lara Peixoto, Lincoln Suesdek

**Affiliations:** 1 Instituto Butantan, São Paulo 05503-900, Brazillincoln.suesdek@butantan.gov.br (L.S.); 2 Faculdade de Medicina, Universidade de São Paulo, Pacaembu 01246-903, Brazil; 3 Instituto de Estudos Avançados, Universidade de São Paulo, São Paulo 05508-050, Brazil

**Keywords:** geometric morphometry, wings, microsatellite DNA, evolution, microevolution, mosquitoes, Culicidae

## Abstract

**Highlights:**

- Morphological diversity of wings is higher in wild than in colonised *Aedes aegypti*
- Colonisation diminishes morphological diversity in *Ae. aegypti*
- Morphological diversity partly corresponds to genetic variability in *Ae. aegypti*
- Wing shape diversity may be used as a proxy to estimate genetic variability

**Simple Summary:**

*Aedes aegypti* mosquito poses a significant global health threat by spreading viruses to millions of people each year. Because current vaccines and medical treatments are often insufficient, scientists have developed genetic tools for monitoring mosquito populations to better control them. Traditionally, these approaches require DNA testing to understand a key parameter, genetic diversity, which allows for the assessment of biological robustness, evolutionary velocity and the dispersal of mosquitoes. However, these methods are often too costly or slow for local health services. We aimed to determine if the geometrical shape of *Ae. aegypti*’s wing could serve as a reliable, low-cost proxy for the genetic analysis. By comparing wild mosquitoes (known to have a high genetic diversity) to those raised in laboratories (with poor genetic diversity), we discovered that wild populations also displayed higher morphological diversity, that is, their wing shapes were more variable. Results suggested a direct link between genetic features and wing geometry. These findings are valuable because they provide a promising “quick and cheap” tool for mosquito surveillance. We hope that health workers can use wing geometry to identify and track medically important mosquitoes without the need for complex laboratory equipment.

**Abstract:**

*Aedes aegypti* transmits viruses to millions of people worldwide. Despite the availability of vaccines, control and monitoring of mosquitoes is mandatory, which in turn requires knowledge of microevolutionary population genetics. Genetic techniques permit the assessment of biological parameters directly linked to the epidemiological importance of the insect (polymorphism, migration, fitness). However, these techniques are costly to most health surveillance services. Even for research laboratories, genotyping and estimation of variability may be unfeasible and time consuming. We conjectured that the wing geometry of *Ae. aegypti* could serve as an alternative indicator of genetic variability in mosquitoes, as wing shape is a useful taxonomic marker determined by quantitative heritage. We investigated this conjecture by testing if wild *Ae. aegypti* populations with high genetic variability had higher wing morphological diversity than inbred colonised populations. Using wing geometric morphometrics and microsatellite DNA genotyping of some populational samples, we confirmed this conjecture. The morphological diversity index was partly correspondent with genetic variability indexes such as theta, gene diversity and alleles per locus. Our findings, although circumscribed to the populational samples studied, indicate that wing geometry may be used as a cheap and quick semi-quantitative proxy for genetic variability.

## 1. Introduction

The urban mosquito *Aedes aegypti* is responsible for vectoring viruses to millions of people in the world annually, including those that cause the diseases Dengue (I–IV), Yellow Fever, Zika, Chikungunya, Mayaro and Oropouche, which are still not satisfactorily mitigated with vaccines or drugs [1,2,3,4].

In this scenario, control of the mosquito is paramount. However, controlling *Ae. aegypti* is a very challenging task because this species has high genetic variability, adaptability, resistance to insecticides, phenotypic plasticity and rapid microevolution [5,6,7], characteristics that make vector control actions extremely difficult. For this reason, *Aedes aegypti* has been the subject of population genetics studies, which allow for the estimation of gene flow, migration, dispersion of resistance, and genetic variability, biological parameters which are directly linked to the epidemiological importance of the insect.

The knowledge on population genetics is especially useful for monitoring *Ae. aegypti* owing to its high genetic variability and other peculiarities [5,8,9,10]. The genetic structure of this species is complex because it is cosmopolitan and also because geographical/temporal populations differ much amongst each other [6,7]. Moreover, studies usually need to qualitatively/quantitatively estimate the genetic variability, as this influences the fitness of these vectors [11,12].

In spite of its usefulness, genetic techniques are costly and usually inaccessible to health surveillance services. Even in research laboratories, tasks such as genotyping, detection of genetic novelties, estimation of genetic variability, etc., may be unfeasible and time consuming when dealing with large samples.

To overcome these obstacles, some researchers have used wing shape characters as alternative indicators of microevolutionary processes and patterns in mosquitoes [6,8,13,14]. The geometric patterns described by wing veins are presumably heritable and determined by quantitative genes [13]. Based on these premises, and also on empirical observations [6], we conjectured that wing shape could serve to quantify genetic variability in mosquitoes, and, somehow, be used as a proxy to detect mosquito populations with high or low genetic variability more quickly and cheaply.

Given this context, we tested the hypothesis that the variability of wing shape is directly proportional to the genetic variability of populations of *Ae. aegypti*. As a complementary approach, we also tested if populations with low genetic variability also had low wing shape variability in comparison to highly generically variable populations.

## 2. Material and Methods

### 2.1. Study Design

We posed two hypotheses concerning the wing shape of *Aedes aegypti*: (A) Free-living “wild” populations have higher morphological diversity than colonised populations; (B) morphological diversity corresponds to the genetic variability of populations (either wild or colonised). To investigate these hypotheses, we respectively performed two tests, Test A and Test B, explained as follows:

#### 2.1.1. Test A—Morphological Diversity: WILD_A_ > COL_A_?

We used morphological diversity [6,15,16] based on the geometry of wing shape to compare among 7 *Ae. aegypti* populational samples: 4 from urban collection (herein referred to as group “WILD_A_”) and 3 from established laboratory colonies (group “COL_A_”). Samples were obtained haphazardly from the WingBank database (https://wingbank.butantan.gov.br, accessed on 10 April 2026) [17] aiming to gather at least 150 representatives of each group. Studied samples are listed in Table 1. More information about the sampling, collecting and datum, are in the Supplementary Materials. Methods of the wing geometry are explained in the following subsections.

#### 2.1.2. Test B—Correspondence Between Morphological Diversity and Genetic Variability?

We used two *Ae. aegypti* populational samples to compare between morphological (MD index and Q_st_) and genetic (6 indexes) methods of variability estimation. The morphological method used geometry of wing shape, whereas the genetic one used allelic data of microsatellite (SSR) loci.

One sample, named “COL_B_”, was a three-generation isolineage established from just three females isolated from an inbred laboratory colony (Higgs strain; +10 years old), in which the overall morpho-genetic variability was presumably low due to inbreeding. The other, a wild populational sample (WILD_B_) was collected in Santos city, Brazil, where individuals recognizably have high genetic variability [18,19,20,21,22]. Studied samples are listed in Table 2 and Table 3, and more information about the isolineage obtainment and field collection may be found at the Supplementary Materials and at WingBank (https://wingbank.butantan.gov.br; accessed on 10 April 2026), respectively. Calculation of morphological and genetic indexes are explained in the following subsections.

### 2.2. Wing Geometry and Morphological Diversity (MD)

**Figure 1:**
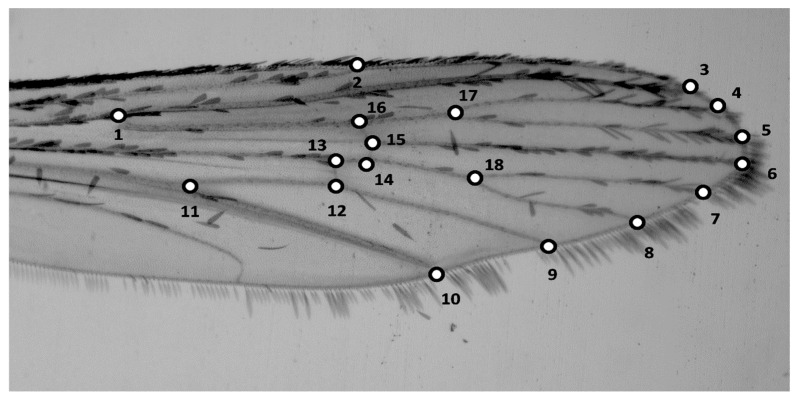
Wing of *Aedes aegypti* (female) depicting the 18 landmarks digitised.

Following standard geometric morphometric protocols [13], we digitised 18 landmarks from mosquito wing images (Figure 1) and transferred their coordinate data to a Cartesian plane using the software TpsDig 1.40 [23]. The Cartesian coordinates were then submitted to Procrustes superposition using the software TpsRelW 1.20 [24], yielding 32 relative warps that are the principal components of shape.

All coordinates of landmarks of the 60 individuals (30 per sample) after Procrustes superimposition were also analysed in a Cartesian plane centred on zero-zero (0,0) in order to estimate their positional variance. Such variance was described in plots of consensus wing. Mean and standard deviations (σ) of each landmark were calculated (Table 4 and Table 5) and submitted to be statistically tested for the hypothesis σ WILD_B_ > σ COL_B_ using *t*-test, linear regression and Pearson correlation.

Based on the principal components, morphological diversity of populational samples WILD_A_, COL_A_, WILD_B_ and COL_B_ were calculated according to Suesdek [6,15,16]. In brief, it consisted of calculating (for each populational sample) the centroid size [25] of the 2-dimensional “cloud” of points formed by individuals in the morphospace of principal components 1 and 2 (relative warps 1 and 2). The larger the centroid size of this “cloud”, the more dispersed the points are and the greater the morphological diversity of that species. We also calculated the phenotypic indicator Q_st_ (MOG software; https://xyom-clic.eu/the-clic-package/, accessed on 10 April 2026) [26] using wing shape data and 1000 iteractions.

### 2.3. SSRs (Microsatellite DNA) Genotyping and Analysis

The DNA of 60 mosquitoes (WILD_B_ and COL_B_) was extracted using DNeasy Blood & Tissue Kit (QIAGEN, Valencia, CA, USA). We genotyped the mosquitoes using the genetic variability based on four SSR microsatellite loci (AG2, AG4, AG7, AT1) according to Slotman et al. [27]. Preliminary amplification protocols were also standardized by our team at Instituto Butantan’s laboratory. The definitive analysed SSRs fragments were amplified by BPI enterprise (BPI—Biotecnologia Pesquisa e Inovação—BPI Botucatu, SP, Brazil) and revised by the authors.

Analysis of genetic data was done using the computer program OSIRIS v.2.16. (National Institutes of Health). Population parameters will be computed according to the method described by Nei [28] and Garza & Williamson [29].

Using the software Arlequin version 3.5.2.2 [30], the genetic parameters calculated (routine with 1000 interactions) from the microsatellite data were the Theta-H index (THETA), average gene diversity over loci (GENDIV), allelic size range (ALSIZERA), index of Garza–Williamson (GARZA), and number of alleles (ALLELES). We also estimated the genetic indicator F-statistics (F_st_) based on the sampled SSR loci (using Arlequin). Factorial correspondence analysis from microsatellite data was performed using DARwin version 6.0.021 software [31].

## 3. Results and Discussion

### 3.1. Test A—Morphological Diversity: WILD_A_ > COL_A_?

**Figure 2:**
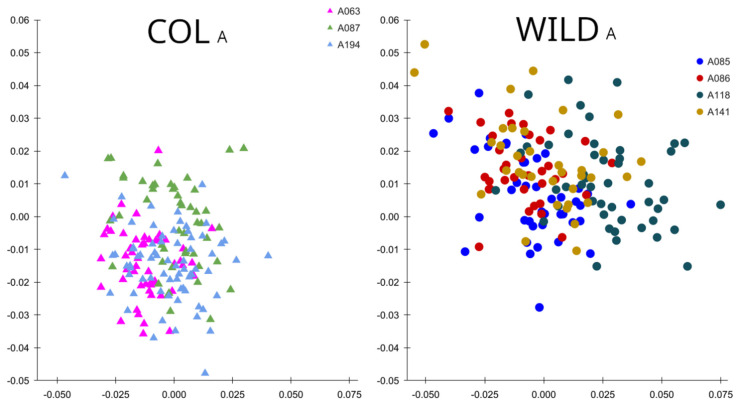
Morphospace of principal components of shape (PC1 and PC2) resulting from the wing shape analysis of *Aedes aegypti* populational samples from groups COL_A_ (triangles) and WILD_A_ (circles). Each dot represents an individual.

Test A showed that the seven populational samples had different MD index values (Table 1). As the samples had a different number of individuals (ranging from 35 to 41), one needs to observe also the mean MD, calculated by the arithmetic mean of a population absolute MD value divided by the number of individuals of that population. Regarding the mean MD, the three colony samples had lower values than those of wild samples, in accordance with the posed A hypothesis. On the morphospace of principal components of shape (relative warps, Figure 2), the overall dispersion of individuals was higher in group WILD_A_, in accordance with the MD index.

### 3.2. Test B—Correspondence Between Morphological Diversity and Genetic Variability?

**Figure 3:**
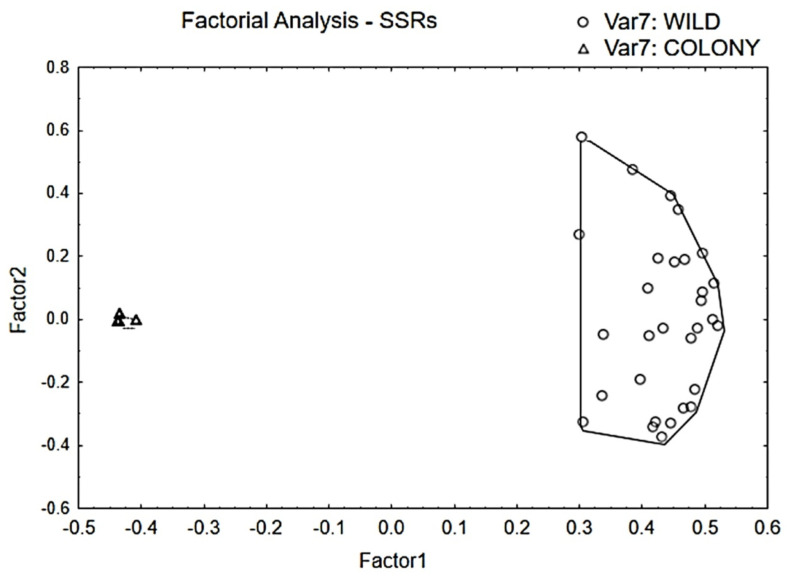
Graphical presentation of factorial analysis based on the genetic variability of SSR markers of populational groups COL_B_ and WILD_B_. Only the first two main factors are represented. There are 60 individuals, 30 from each group. Note that the plot “cloud” of COL_B_ is much smaller, given that most individuals overlapped and are difficult to distinguish.

The genetic analysis of Test B, summarised in Table 2 and Table 3, showed that the allelic profile of COL_B_ was oligomorphic (1.75 average alleles per locus), while that of WILD_B_ was polymorphic (11.75 average alleles per locus). All population genetics parameters (Table 2 and Table 3) and the factorial analysis (Figure 3) confirmed the higher genetic variability of WILD_B_.

**Figure 4:**
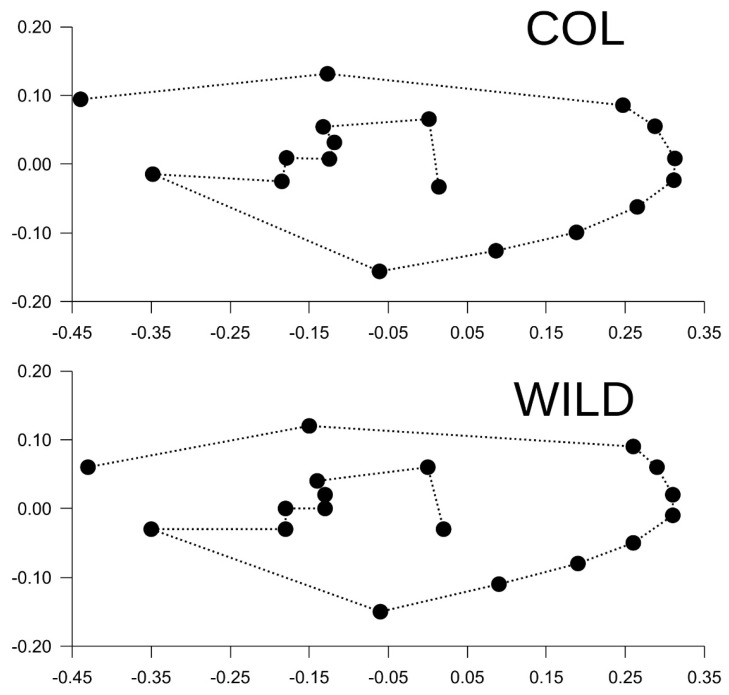
Graphical presentation on consensus wings of COL_B_ and WILD_B_ after Procrustes superimposition. Landmarks are linked by a hypothetical line following the sequence of digitisation just in order to facilitate visual comparison between populations. Note, for instance, that LM2 is more proximal and LM18 is more distal in WILD_B_.

**Figure 5:**
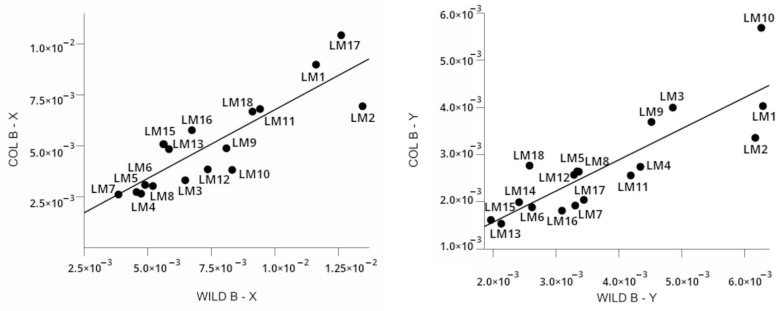
Plot of the standard deviations of positional wing coordinates of landmarks of COL_B_ and WILD_B_. The straight ascending line represents the linear correlation between values of both populational samples. The standard deviations of coordinates of each landmark stem from the *X* axis (left plot) and *Y* axis (right plot) after Procrustes superimposition of wings. Original data are listed in Table 4 and Table 5). Correlations were statistically significant and positive in both axes (*X* axis, R^2^ = 0.739; *Y* axis, R^2^ = 0.734). Standard deviation values were higher in WILD for each landmark (except for LM18 at *Y* axis), as confirmed by the correlation equations COL_B_ = WILD_B_ × 0.674 + 0.0000311 (*X* axis) and COL_B_ = WILD_B_ × 0.659 + 0.000252 (*Y* axis).

Similarly, the morphological analysis revealed that WILD_B_ was more diverse than COL_B_. The higher positional variability (standard deviation values) of WILD_B_ landmarks reached statistical significance (*p* < 0.001; paired *t*-test). However, by observing the wing shape consensus (Figure 4) such differences were sub-visual and could not be discerned without inferential statistics. Linear regression associated with Pearson correlation confirmed that morphological variability is more pronounced in WILD_B_, even when each landmark was analysed separately. See Figure 5 for further details.

**Figure 6:**
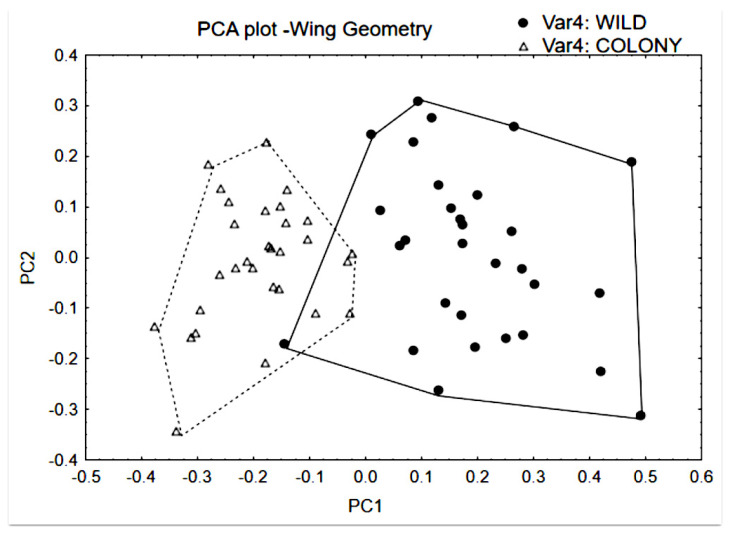
Morphospace of principal components 1 and 2 (respectively, relative warps 1 and 2) from the comparative analysis of wing geometry between groups. Sample = 60 individuals, being 30 of each group. Note that the plot “cloud” of WILD_B_ is larger than that of COL_B_. MD index values are described in the Results section.

**Figure 7:**
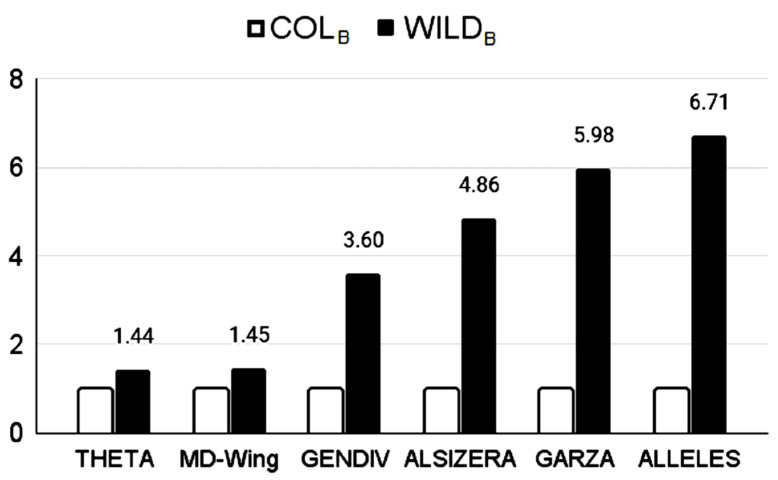
The WILD_B_/COL_B_ ratio according to each indicator of variability (morphological or genetic). To facilitate comparison, COL_B_ values were standardised to =1, whereas WILD_B_ expresses a multiple of COL_B_. Original values are depicted in Table 1, Table 2, Table 3, Table 4 and Table 5 and in the Results section.

Higher morphological variability of WILD_B_ was also observed in the principal component morphospace of wing geometry (Figure 6), where the MD index values were 6.690 for COL_B_, and 9.704 for WILD_B_. The result of the intergroup analysis between COL_B_ and WILD_B_ was that Q_st_ < F_st_. More specifically, the genetic indicator F_st_ was 0.423 (*p* = 0.00001; four loci) whereas the phenotypic indicator Q_st_ was 0.307 (just the 11 most influential relative warps were taken, comprising 95% of the variability). The correspondence between the genetic indicators and the morphological indicator is summarised in Figure 7.

### 3.3. General Discussion

The present results support the posed hypotheses, at least in this particular study. The higher MD index value of field *Aedes aegypti* populations in comparison to colony samples (Test A; Figure 1) was previously expected owing to the typically higher overall genetic variability of wild animals over populations that suffer from the bottleneck effect of being maintained in captivity. Although this is an empirical observation derived from just a few samples, the fact that wing geometry was less diverse in colonised individuals reinforced the theory that wing shape in mosquitoes is heritable and polygenic [6,13].

Coherently, the results of Test B also confirmed that both morphological and genetic diversity indicators were higher in the field population, which was collected in Santos (Brazil), a seaport city known to be an entry point of exotic lineages and a hotspot of variability [18]. Apparently, the isolineage has concomitantly lost genetic and morphological variability during the process of artificial inbreeding, that is, it may have undergone a genetic bottleneck, as observed in other colonised populations of *Ae. aegypti* [32]. Our findings indicate that there is some positive correspondence between genetic and morphological variabilities. One could then interpret that measuring wing variability can be an indirect way of estimating genetic variability of *Ae. aegypti*.

However, the discrepancy between populational groups was less pronounced when morphological characters were used rather than genetic characters (Figure 3, Figure 6 and Figure 7). This means that the correspondence between morphological and genetic indicators was just partial.

We still do not know the biological reasons that render the correspondence partial, but we might think of some hypotheses. Arguably, some kind of developmental constraint or evolutionary canalisation [13] could prevent wing shape from evolving as freely as the SSRs markers do. On the other hand, environmental influences seem not to be capable of totally disrupting that correspondence in *Ae. aegypti*, because phenotypical plasticity does not strongly affect the wing shape of this species, as experimentally demonstrated by Jirakanjanakit et al. [33].

Remarkably, an opposite scenario has been observed by Agboli et al. [34], who reported that wing shape failed to distinguish between genetically structured (mtDNA) field and urban populations of *Ae. aegypti* in West Africa. This apparent discrepancy between that study [34] and our present results might suggest that the correspondence between wing shape and genetics is not universal to the species; however, other explanations are possible. The aims of their work differed from ours, as they employed wing shape to investigate the existence of geographical population structure rather than comparing the magnitude of variability (either genetic or morphological) between populations. Moreover, a preliminary analysis (conducted by us, Figure S3, Supplementary Materials) using the morphospace of principal components of field and urban samples provided by them [34], indicates that the mean MD of the field sample is higher, which is in consonance with our current interpretations.

Despite the fact that correspondence is not perfect and that our knowledge on the reported phenomenon is still incipient, results lead us to believe that wing shape in *Ae. aegypti* is evolutionarily neutral, at least regarding the area comprised by 18 studied landmarks. The usual interpretation of Q_st_ < F_st_ is that the involved quantitative traits are not under natural selection [35,36,37], thus we may consider that wing shape characters are nearly neutral, similar to the SSRs. Therefore, although the correspondence between MD index and genetic indexes is only partial, the neutrality present in both sets of characters supports the idea of the evolutionary informativeness of wing shape.

Taking our results and the previous knowledge from the literature, our comprehensive interpretation is that the MD index based on wing shape can serve at least as a semi-quantitative proxy for estimating variability. However, at this point, the MD index cannot fully substitute the genetic markers (“gold standards”), notedly SSRs, in the assessment of biological inherited variability.

We are aware of the limitations of the current study, as, for example, in Test B we did not investigate the frequency of putative genes that code for wing shape because genetic determination of wing shape is not completely known. Moreover, samples of Test A were collected from the WingBank, which may not be an optimised representative of the natural variability.

However, even under the existence of these unsolved questions, wing shape characters have been extensively used in the literature as taxonomic and populational markers for mosquitoes [38,39]. With the advent of our findings, the use of wing shape to describe microevolutionary patterns (or processes) of populational entities may gain more support.

We conclude that wing shape, although not suitable for calculating gene flow, is sensitive enough to detect bottleneck, genetic drift and polymorphisms, which are helpful information for mosquito surveillance. Detection of reinfestation and recent biological variation is also possible via studies of wing geometry, as revised by Dujardin [13]. Wing characters can continue to be used for preliminary and cheap populational studies before using the most accurate and costly genetic approaches.

The definitive validation of our interpretation may come as further studies are conducted. We believe that the scientific scenario is sufficiently equipped for this, as this is a highly dynamic research field, and there are databases with thousands of wing images publicly available [17,26,40]. Additionally, the global challenge of monitoring mosquito vectors of pathogens remains a significant issue for the world.

## Figures and Tables

**Table 1:** Data and results regarding Test A. Values of MD and of the arithmetic mean MD for each population are represented in decimal notation. For more information about the samples, see the Supplementary Materials using sample codes.

Group	Populational Sample (WingBank Code)	City	MD (×10^1^)	N of Individuals		MD Mean (×10^3^)
COL_A_	A63	São Paulo	1.093	54	173	2.025
	A194	Rio de Janeiro	1.546	71		2.178
	A87	Recife	1.349	48		2.810
WILD_A_	A86	Recife	1.038	35	159	2.966
	A85	Petrolina	1.392	42		3.314
	A118	Santos	1.671	47		3.554
	A141	Santos	1.488	35		4.251

**Table 2:** Genotyping WILD_B_. Alleles (base pairs) of each individual (lines) are shown according to the respective SSR locus (columns). For more information about samples (datum, collecting, images), see the Supplementary Materials. Genetic variability parameters of the whole populational sample are at the bottom of the table.

WILD_B_ (n = 30)	Microsatellite Locus/Alleles			
Specimen Code	AG2	AG4	AG7	AT1
S1	123	171	161, 170	164
S3	120	169, 171	161, 211	170, 174
S5	122, 157	169	162, 166	170
S7	123	171	162	166, 174
S8	138	171	167, 170	166, 178
S9	120, 157	171	161, 170	168
S12	120, 122	169	162, 165	168, 170
S13	120, 140	171	167, 170	164, 174
S14	123, 138	169, 171	162	168, 172
S15	120, 136	169, 171	162, 166	166, 168
S16	123	167, 171	162, 170	173
S17	120, 157	167, 171	162, 170	166, 168
S19	120	169, 171	161, 170	166, 173
S20	147, 163	171	161, 170	168
S21	136, 163	171	170	166, 172
CAST-2	120, 123	168, 170	157, 162	165
CAST-3	124, 137	150, 170	160, 161	165
CENT-146	124, 162	170	162	161, 163
CENT-151	149, 162	170	162, 169	163, 173
CENT-152	124, 164	170	164, 165	163, 169
CENT-155	124, 162	170	169	161, 169
CENT-157	124, 162	150	162, 169	163
CENT-160	124, 164	170	162	161, 163
CENT-162	124, 162	170	162, 169	161, 163
GZGA-10	124, 137	170	161, 162	169, 173
GZGA-8	120, 121	170	161, 169	165
GZGA-9	124, 162	150, 163	169	163, 169
MACC3	122, 137	170	165, 169	161, 173
PONT-92	157	164, 170	162, 165	165, 167
PONT-95	120, 123	170	169	163, 169
ALLELES	11.750			
GARZA	0.4048			
ALSIZERA	34			
GENDIV	0.8435			
THETA	3.28771			

**Table 3:** Genotyping COL_B_. The overall description is similar to Table 2.

COL_B_ (n = 30)	Microsatellite Locus/Alleles			
Specimen Code	AG2	AG4	AG7	AT1
ISO-01	121	150	169	173, 175
ISO-02	121	150	167, 169	163, 175
ISO-03	121	150	167, 169	163, 175
ISO-04	121	150	167, 169	163, 175
ISO-05	121	150	169	175
ISO-06	121	150	167, 169	163, 175
ISO-07	121	150	169	173, 175
ISO-08	121	150	167, 169	163, 175
ISO-09	121	150	167, 169	175
ISO-10	121	150	167, 169	163,175
ISO-11	121	150	167, 169	175
ISO-12	121	150	169	175
ISO-13	121	150	167, 169	163, 175
ISO-14	121	150	167, 169	163, 175
ISO-15	121	150	167, 169	163, 175
ISO-16	121	150	167, 169	163, 175
ISO-17	121	150	167, 169	163, 175
ISO-19	121	150	169	175
ISO-20	121	150	169	175
ISO-21	121	150	167, 169	163, 175
ISO-23	121	150	167, 169	163, 175
ISO-24	121	150	167, 169	163, 175
ISO-25	121	150	169	175
ISO-26	121	150	169	175
ISO-27	121	150	167, 169	163, 175
ISO-29	121	150	169	163, 175
ISO-30	121	150	167, 169	163, 175
ISO-31	121	150	169	175
ISO-32	121	150	167, 169	163, 175
ISO-33	121	150	167, 169	163, 175
ALLELES	1.750			
GARZA	0.06768			
ALSIZERA	7			
GENDIV	0.234			
THETA	2.28917			

**Table 4:** Positional coordinates of landmarks from digitised wings after Procrustes superimposition (mean of 30 individuals) in a Cartesian plane centred on zero-zero (0,0). Only wing coordinates of the X axis are listed here. Respective standard deviations of coordinates of each LM are also listed, showing that all standard deviation values are higher in Wild for each landmark.

	WILD B—X Axis		COL B—X Axis	
	Mean	Std. Deviation	Mean	Std. Deviation
LM1	−0.4316151106	0.011613177	−0.4393144425	0.008984368
LM2	−0.1477323128	0.013442644	−0.1269268975	0.006943304
LM3	0.2598274996	0.006469256	0.2469651005	0.003309255
LM4	0.2935718102	0.004734155	0.2874562717	0.002649760
LM5	0.3130958569	0.004552384	0.3126910372	0.002731581
LM6	0.3107348887	0.004891252	0.3113799041	0.003079127
LM7	0.2649144755	0.003841748	0.2650672731	0.002608530
LM8	0.1933031935	0.005195667	0.1881256422	0.003026397
LM9	0.0922351113	0.008089056	0.0863151319	0.004878375
LM10	−0.0562322519	0.008315214	−0.0610528553	0.003816243
LM11	−0.3507689344	0.009410979	−0.3479686215	0.006807043
LM12	−0.1813297566	0.007355147	−0.1846169283	0.003842012
LM13	−0.1795024261	0.005829910	−0.1786742509	0.004835861
LM14	−0.1314763622	0.005628504	−0.1244861655	0.005076856
LM15	−0.1261085139	0.005604307	−0.1181589638	0.005079688
LM16	−0.1390644865	0.006731053	−0.1323487589	0.005765942
LM17	−0.0024461888	0.012601661	0.0015065368	0.010428570
LM18	0.0185935082	0.009113561	0.0140409866	0.006680104
Mean		0.007412204		0.005030168

**Table 5:** Positional coordinates of landmarks from digitised wings after Procrustes superimposition (mean of 30 individuals) in a Cartesian plane centred on zero-zero (0,0). Only wing coordinates of the Y axis are listed here. Respective standard deviations of coordinates of each LM are also listed, showing that all standard deviation values were higher in Wild for each landmark, except for LM18.

	WILD B—Y		COL B—Y	
	Mean	Std. Deviation	Mean	Std. Deviation
LM1	0.0620296389	0.006294168181	0.0941342492	0.004028072809
LM2	0.1209083901	0.006172743165	0.1313035491	0.0033554143
LM3	0.0892147260	0.004859042637	0.0855673542	0.003995738297
LM4	0.0630034322	0.004342208266	0.0548644524	0.002740990913
LM5	0.0241464599	0.00333493759	0.0081208125	0.00264394452
LM6	−0.0087223215	0.002616157477	−0.0234949822	0.001882474323
LM7	−0.0456744647	0.003304244967	−0.0624223262	0.001920770283
LM8	−0.0809661123	0.003361085526	−0.0994092596	0.002637638977
LM9	−0.1110873598	0.004518861905	−0.1262369058	0.003691234517
LM10	−0.1485640485	0.006268577698	−0.1561821486	0.005685835856
LM11	−0.0348859774	0.004189293621	−0.0147694286	0.002558086497
LM12	−0.0292906797	0.003285263047	−0.0253083146	0.002571441995
LM13	−0.0020813646	0.002125825856	0.0088642330	0.001536003358
LM14	0.0022397424	0.002409825402	0.0072941525	0.001991197308
LM15	0.0244320606	0.001960775578	0.0314223155	0.001615414075
LM16	0.0436403972	0.00309335065	0.0540790797	0.001811693002
LM17	0.0615094555	0.003441632865	0.0654004596	0.002040435007
LM18	−0.0298519744	0.002576766634	−0.0332272918	0.002766727716
mean		0.003786375615		0.00274850632

## Data Availability

The original contributions presented in this study are included in the article/Supplementary Material. Further inquiries can be directed to the corresponding author.

## Supplementary Materials

The following supporting information can be downloaded at: https://www.mdpi.com/article/10.3390/insects17050469/s1. The data that support the findings of this study are available in Wingbank, in the Supplementary Materials or under request. Table S1: List of studied samples available at Wingbank, according to sample codes. Group COL_A_: Table S2: List of studied samples available at Wingbank, according to sample codes. Group WILD_A_: Table S3: List of wing images analysed in Test B, available at WingBank; Figure S1: Heredogram depicting the crossing scheme for obtaining the isolineage COL_B_; Figure S2: Genotyping electropherograms, 60 samples (30 WILD_B_, 30 COL_B_), 4 SSR loci; Figure S3: Calculation of Morphological Diversity of *Aedes aegypti*, based on data from Agboli et al. 2025 [34].
