# Supplementary material for: Morphological Diversity as a Proxy for Assessing Genetic Diversity of Aedes aegypti (Diptera, Culicidae)
Source: Insects. 2026 Apr 30;17(5):469. doi: 10.3390/insects17050469 (PMC13206977; doi:10.3390/insects17050469)

## Supplementary Material

Table S1 - List of studied samples available at Wingbank , according to sample codes, Group $COL_A$. Line 1: Number of individuals and city (Brazil) where colony was kept are show respective to each population sample. Other lines: Each line represents a wing image of a female individual, described by its complete WingBank code. Datum respective to each sample is also available at WingBank.

| | A63, n=54<br>Sao Paulo | A194, n=71<br>Rio de Janeiro | A87, n=48<br>Recife |
|---|---|---|---|
| 1 | A63T4FD1.jpg | 14PT3-D.jpg | Pop.Rec-Lab 01D.jpg |
| 2 | A63T4FD10.jpg | 17PT3-D.jpg | Pop.Rec-Lab 02D.jpg |
| 3 | A63T4FD11.jpg | 25ERLT-D(F).jpg | Pop.Rec-Lab 04D.jpg |
| 4 | A63T4FD2.jpg | 28ERLT-D.jpg | Pop.Rec-Lab 05D.jpg |
| 5 | A63T4FD3.jpg | 28PT1-D.jpg | Pop.Rec-Lab 06D.jpg |
| 6 | A63T4FD6.jpg | 29ERLT-D.jpg | Pop.Rec-Lab 07D.jpg |
| 7 | A63T4FD7.jpg | 30ERLT-D.jpg | Pop.Rec-Lab 08D.jpg |
| 8 | A63T4FD9.jpg | 31ERLT-D.jpg | Pop.Rec-Lab 09D.jpg |
| 9 | A63T5FD1.jpg | 32ERLT-D.jpg | Pop.Rec-Lab 10D.jpg |
| 10 | A63T5FD10.jpg | 33ERLT-D.jpg | Pop.Rec-Lab 11D.jpg |
| 11 | A63T5FD11.jpg | 33PT1-D.jpg | Pop.Rec-Lab 12D.jpg |
| 12 | A63T5FD12.jpg | 33PT2-D.jpg | Pop.Rec-Lab 14D.jpg |
| 13 | A63T5FD13.jpg | 34ERLT-D.jpg | Pop.Rec-Lab 15D.jpg |
| 14 | A63T5FD14.jpg | 34PT2-D.jpg | Pop.Rec-Lab 16D.jpg |
| 15 | A63T5FD15.jpg | 35PT1-D.jpg | Pop.Rec-Lab 17D.jpg |
| 16 | A63T5FD16.jpg | 36ERLT-D.jpg | Pop.Rec-Lab 18D.jpg |
| 17 | A63T5FD18.jpg | 37ERLT-D.jpg | Pop.Rec-Lab 19D.jpg |
| 18 | A63T5FD19.jpg | 38ERLT-D.jpg | Pop.Rec-Lab 20D.jpg |
| 19 | A63T5FD22.jpg | 38PT2-D.jpg | Pop.Rec-Lab 21D.jpg |
| 20 | A63T5FD23.jpg | 39ERLT-D.jpg | Pop.Rec-Lab 23D.jpg |
| 21 | A63T5FD24.jpg | 39PT1-D.jpg | Pop.Rec-Lab 24D.jpg |

| | | | |
|---|---|---|---|
| 22 | A63T5FD25.jpg | 3ERLT-D.jpg | Pop.Rec-Lab 25D.jpg |
| 23 | A63T5FD27.jpg | 3PT1-D.jpg | Pop.Rec-Lab 26D.jpg |
| 24 | A63T5FD29.jpg | 40ERLT-D.jpg | Pop.Rec-Lab 27D.jpg |
| 25 | A63T5FD3.jpg | 40PT1-D.jpg | Pop.Rec-Lab 28D.jpg |
| 26 | A63T5FD30.jpg | 40PT2-D.jpg | Pop.Rec-Lab 29D.jpg |
| 27 | A63T5FD4.jpg | 40PT3-D.jpg | Pop.Rec-Lab 30D.jpg |
| 28 | A63T5FD5.jpg | 41ERLT-D.jpg | Pop.Rec-Lab 31D.jpg |
| 29 | A63T5FD6.jpg | 41PT2-D.jpg | Pop.Rec-Lab 32D.jpg |
| 30 | A63T5FD7.jpg | 41PT3-D.jpg | Pop.Rec-Lab 33D.jpg |
| 31 | A63T5FD8.jpg | 42PT1-D.jpg | Pop.Rec-Lab 34D.jpg |
| 32 | A63T5FD9.jpg | 42PT2-D.jpg | Pop.Rec-Lab 35D.jpg |
| 33 | A63T6FD1.jpg | 43PT2-D.jpg | Pop.Rec-Lab 36D.jpg |
| 34 | A63T6FD11.jpg | 44PT2-D.jpg | Pop.Rec-Lab 37D.jpg |
| 35 | A63T6FD12.jpg | 45ERLT-D.jpg | Pop.Rec-Lab 38D.jpg |
| 36 | A63T6FD13.jpg | 45PT3-D.jpg | Pop.Rec-Lab 39D.jpg |
| 37 | A63T6FD14.jpg | 46ERLT-D.jpg | Pop.Rec-Lab 40D.jpg |
| 38 | A63T6FD15.jpg | 48PT2-D.jpg | Pop.Rec-Lab 41D.jpg |
| 39 | A63T6FD16.jpg | 49PT2-D.jpg | Pop.Rec-Lab 42D.jpg |
| 40 | A63T6FD17.jpg | 50ERLT-D.jpg | Pop.Rec-Lab 43D.jpg |
| 41 | A63T6FD18.jpg | 5ERLT-D.jpg | Pop.Rec-Lab 44D.jpg |
| 42 | A63T6FD19.jpg | 6PT3-D.jpg | Pop.Rec-Lab 45D.jpg |
| 43 | A63T6FD2.jpg | A194T06-PT1Adl24FD.jpg | Pop.Rec-Lab 46D.jpg |
| 44 | A63T6FD20.jpg | A194T06-PT1Adl31FD.jpg | Pop.Rec-Lab 47D.jpg |
| 45 | A63T6FD21.jpg | A194T06-PT1Adl32FD.jpg | Pop.Rec-Lab 48D.jpg |
| 46 | A63T6FD23.jpg | A194T06-PT1Adl34FD.jpg | Pop.Rec-Lab 49D.jpg |
| 47 | A63T6FD25.jpg | A194T06-PT1Adl37FD.jpg | Pop.Rec-Lab 50D.jpg |
| 48 | A63T6FD26.jpg | A194T06-PT1Adl44FD.jpg | Pop.Rec-Lab 51D.jpg |

| | | | |
|---|---|---|---|
| 49 | A63T6FD27.jpg | A194T06-PT1Adl46FD.jpg | |
| 50 | A63T6FD3.jpg | A194T06-PT1Adl50FD.jpg | |
| 51 | A63T6FD4.jpg | A194T06-PT2Adl18FD.jpg | |
| 52 | A63T6FD7.jpg | A194T06-PT2Adl32FD.jpg | |
| 53 | A63T6FD8.jpg | A194T06-PT2Adl34FD.jpg | |
| 54 | A63T6FD9.jpg | A194T06-PT2Adl35FD.jpg | |
| 55 | | A194T06-PT2Adl3FD.jpg | |
| 56 | | A194T06-PT2Adl40FD.jpg | |
| 57 | | A194T06-PT2Adl46FD.jpg | |
| 58 | | A194T06-PT2Adl49FD.jpg | |
| 59 | | A194T06-PT2Adl8FD.jpg | |
| 60 | | A194T06-PT3Adl11FD.jpg | |
| 61 | | A194T06-PT3Adl12FD.jpg | |
| 62 | | A194T06-PT3Adl15FD.jpg | |
| 63 | | A194T06-PT3Adl15FD.jpg | |
| 64 | | A194T06-PT3Adl37FD.jpg | |
| 65 | | A194T06-PT3Adl41FD.jpg | |
| 66 | | A194T06-PT3Adl42FD.jpg | |
| 67 | | A194T06-PT3Adl43FD.jpg | |
| 68 | | A194T06-PT3Adl44FD.jpg | |
| 69 | | A194T06-PT3Adl6FD.jpg | |
| 70 | | A194T06-PT3Adl8FD.jpg | |
| 71 | | A194T06-PT3Adl9FD.jpg | |

Table S2 - List of studied samples available at Wingbank , according to sample codes, Group $WILD_A$ . Line 1: Populational sample, number of individuals and city (Brazil) where samples were collected in the field (approximate geographical coordinates). Other lines: Each line represents a wing image of a female individual, described by its complete WingBank code. Respective Datum for each sample is also available at WingBank.

| | A86, n=35<br>Recife<br>8°03'S 34°53'W | A85, n=42<br>Petrolina<br>9° 23′ S 40°30′W | A118, n=47<br>Santos<br>23° 56′ S 46°19′W | A141, n=35<br>Santos<br>23° 56′S 46°19′W |
|---|---|---|---|---|
| 1 | Pop.Recife 01D.jpg | 11d | A118T1 FD 11.jpg | A141T1 FD1.jpg |
| 2 | Pop.Recife 02D.jpg | Pop.Petrolina01D.jpg | A118T1 FD 12.jpg | A141T1 FD10.jpg |
| 3 | Pop.Recife 03D.jpg | Pop.Petrolina02D.jpg | A118T1 FD 13.jpg | A141T1 FD12.jpg |
| 4 | Pop.Recife 04D.jpg | Pop.Petrolina03D.jpg | A118T1 FD 14.jpg | A141T1 FD13.jpg |
| 5 | Pop.Recife 06D.jpg | Pop.Petrolina04D.jpg | A118T1 FD 15.jpg | A141T1 FD14.jpg |
| 6 | Pop.Recife 07D.jpg | Pop.Petrolina05D.jpg | A118T1 FD 16.jpg | A141T1 FD17.jpg |
| 7 | Pop.Recife 08D.jpg | Pop.Petrolina06D.jpg | A118T1 FD 17.jpg | A141T1 FD18.jpg |
| 8 | Pop.Recife 09D.jpg | Pop.Petrolina07D.jpg | A118T1 FD 18.jpg | A141T1 FD2.jpg |
| 9 | Pop.Recife 11D.jpg | Pop.Petrolina08D.jpg | A118T1 FD 19.jpg | A141T1 FD3.jpg |
| 10 | Pop.Recife 13D.jpg | Pop.Petrolina09D.jpg | A118T1 FD 2.jpg | A141T1 FD4.jpg |
| 11 | Pop.Recife 14D.jpg | Pop.Petrolina12D.jpg | A118T1 FD 20.jpg | A141T1 FD5.jpg |
| 12 | Pop.Recife 15D.jpg | Pop.Petrolina14D.jpg | A118T1 FD 21.jpg | A141T1 FD6.jpg |
| 13 | Pop.Recife 17D.jpg | Pop.Petrolina15D.jpg | A118T1 FD 22.jpg | A141T1 FD7.jpg |
| 14 | Pop.Recife 18D.jpg | Pop.Petrolina16D.jpg | A118T1 FD 23.jpg | A141T1 FD9.jpg |
| 15 | Pop.Recife 19D.jpg | Pop.Petrolina18D.jpg | A118T1 FD 24.jpg | A141T1 FD19.jpg |
| 16 | Pop.Recife 21D.jpg | Pop.Petrolina19D.jpg | A118T1 FD 25.jpg | A141T1 FD20.jpg |
| 17 | Pop.Recife 22D.jpg | Pop.Petrolina20D.jpg | A118T1 FD 26.jpg | A141T1 FD21.jpg |
| 18 | Pop.Recife 24D.jpg | Pop.Petrolina21D.jpg | A118T1 FD 27.jpg | A141T1 FD22.jpg |
| 19 | Pop.Recife 25D.jpg | Pop.Petrolina22D.jpg | A118T1 FD 28.jpg | A141T1 FD23.jpg |
| 20 | Pop.Recife 26D.jpg | Pop.Petrolina23D.jpg | A118T1 FD 29.jpg | A141T1 FD24.jpg |
| 21 | Pop.Recife 27D.jpg | Pop.Petrolina24D.jpg | A118T1 FD 3.jpg | A141T1 FD25.jpg |
| 22 | Pop.Recife 28D.jpg | Pop.Petrolina25D.jpg | A118T1 FD 30.jpg | A141T1 FD26.jpg |
| 23 | Pop.Recife 29D.jpg | Pop.Petrolina26D.jpg | A118T1 FD 31.jpg | A141T1 FD27.jpg |
| 24 | Pop.Recife 30D.jpg | Pop.Petrolina27D.jpg | A118T1 FD 32.jpg | A141T1 FD28.jpg |

| | | | | |
|---|---|---|---|---|
| 25 | Pop.Recife 31D.jpg | Pop.Petrolina28D.jpg | A118T1 FD 33.jpg | A141T1 FD29.jpg |
| 26 | Pop.Recife 32D.jpg | Pop.Petrolina29D.jpg | A118T1 FD 34.jpg | A141T1 FD31.jpg |
| 27 | Pop.Recife 33D.jpg | Pop.Petrolina30D.jpg | A118T1 FD 35.jpg | A141T1 FD32.jpg |
| 28 | Pop.Recife 34D.jpg | Pop.Petrolina31D.jpg | A118T1 FD 36.jpg | A141T1 FD33.jpg |
| 29 | Pop.Recife 35D.jpg | Pop.Petrolina32D.jpg | A118T1 FD 37.jpg | A141T1 FD34.jpg |
| 30 | Pop.Recife 36D.jpg | Pop.Petrolina33D.jpg | A118T1 FD 38.jpg | A141T1 FD36.jpg |
| 31 | Pop.Recife 37D.jpg | Pop.Petrolina35D.jpg | A118T1 FD 39.jpg | A141T1 FD40.jpg |
| 32 | Pop.Recife 38D.jpg | Pop.Petrolina36D.jpg | A118T1 FD 4.jpg | A141T1 FD41.jpg |
| 33 | Pop.Recife 39D.jpg | Pop.Petrolina37D.jpg | A118T1 FD 40.jpg | A141T1 FE30.jpg |
| 34 | Pop.Recife 40D.jpg | Pop.Petrolina38D.jpg | A118T1 FD 41.jpg | A141T1 FE35.jpg |
| 35 | Pop.Recife 41D.jpg | Pop.Petrolina39D.jpg | A118T1 FD 42.jpg | A141T1 FE37.jpg |
| 36 | | Pop.Petrolina40D.jpg | A118T1 FD 43.jpg | |
| 37 | | Pop.Petrolina41D.jpg | A118T1 FD 44.jpg | |
| 38 | | Pop.Petrolina42D.jpg | A118T1 FD 45.jpg | |
| 39 | | Pop.Petrolina43D.jpg | A118T1 FD 46.jpg | |
| 40 | | Pop.Petrolina44D.jpg | A118T1 FD 47.jpg | |
| 41 | | Pop.Petrolina45D.jpg | A118T1 FD 48.jpg | |
| 42 | | Pop.Petrolina46D.jpg | A118T1 FD 49.jpg | |
| 43 | | | A118T1 FD 5.jpg | |
| 44 | | | A118T1 FD 50.jpg | |
| 45 | | | A118T1 FD 6.jpg | |
| 46 | | | A118T1 FD 8.jpg | |
| 47 | | | A118T1 FD 9.jpg | |

Table S3- List of wing images analysed in Test B, available at WingBank. The sample $WILD_B$ was collected from Santos City (23° 56′ S 46°19′W) in. ……2022. The $COL_B$ sample is an isolineage kept in captivity (see Fig. S1).

| | $WILD_B$ B014 | $COL_B$ B015 |
|---|---|---|
| 1 | S1.jpg | ISO-01.jpg |
| 2 | S3.jpg | ISO-02.jpg |
| 3 | S5.jpg | ISO-03.jpg |
| 4 | S7.jpg | ISO-04.jpg |
| 5 | S8.jpg | ISO-05.jpg |
| 6 | S9.jpg | ISO-06.jpg |
| 7 | S12.jpg | ISO-07.jpg |
| 8 | S13.jpg | ISO-08.jpg |
| 9 | S14.jpg | ISO-09.jpg |
| 10 | S15.jpg | ISO-10.jpg |
| 11 | S16.jpg | ISO-11.jpg |
| 12 | S17.jpg | ISO-12.jpg |
| 13 | S19.jpg | ISO-13.jpg |
| 14 | S20.jpg | ISO-14.jpg |
| 15 | S21.jpg | ISO-15.jpg |
| 16 | CAST-2.jpg | ISO-16.jpg |
| 17 | CAST-3.jpg | ISO-17.jpg |
| 18 | CENT-146.jpg | ISO-19.jpg |
| 19 | CENT-151.jpg | ISO-20.jpg |
| 20 | CENT-152.jpg | ISO-21.jpg |
| 21 | CENT-155.jpg | ISO-23.jpg |
| 22 | CENT-157.jpg | ISO-24.jpg |
| 23 | CENT-160.jpg | ISO-25.jpg |
| 24 | CENT-162.jpg | ISO-26.jpg |
| 25 | GZGA-10.jpg | ISO-27.jpg |
| 26 | GZGA-8.jpg | ISO-29.jpg |

| | | |
|---|---|---|
| 27 | GZGA-9.jpg | ISO-30.jpg |
| 28 | MACC3.jpg | ISO-31.jpg |
| 29 | PONT-92.jpg | ISO-32.jpg |
| 30 | PONT-95.jpg | ISO-33.jpg |

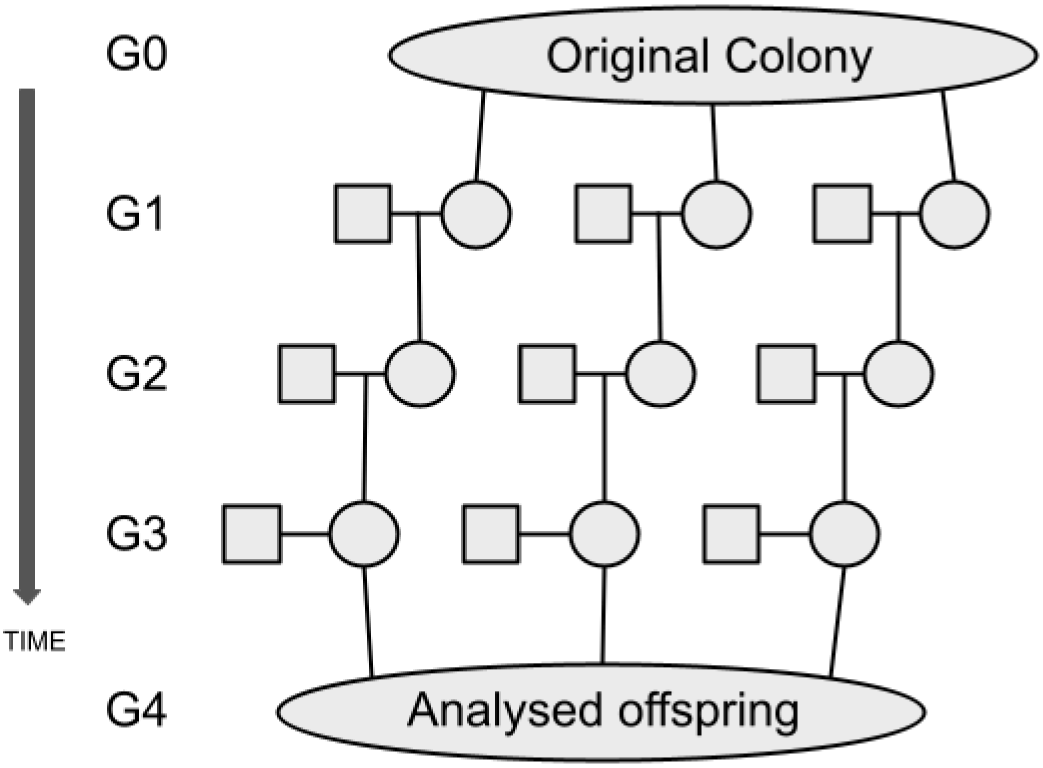


Figure S1. Heredogram depicting the crossing scheme for obtaining the isolineage named $COL_B$, comprising five successive generations, from G0 to G4. The "Original Colony" was an *Aedes aegypti* Higgs strain colony maintained at Butantan Institute since the year 2007. From the G0 step we selected 3 females that crossed with males from the same batch, and from their offsprings, just one female of each offspring was selected to originate the next generation, and so on, to G3. Finally, from G3, we collected 30 females to be analyzed as $COL_B$.

Figure S2 - Genotyping electropherograms, 60 samples (30 $WILD_B$, 30 $COL_B$), 4 SSR loci.

**Genotyping, raw data**

Here are all the chromatograms yielded after genotyping of 30 individual samples from Colony, 30 individual samples from Wild, Loci AG2. AG4. AG7 and AT1. Allele size determination may not be precise in those cases where peaks were multiple. One has to take into consideration that amongst the multiple peaks there are actual alleles and spurious peaks. Three of our authors (3 observers) analyzed the chromatograms and suggested peak readings in each case. This procedure has a certain degree of subjectivism. In the end, the consensus among the 3 readings was used for subsequent analyses.

**Colony. Locus**

**AG2**

**1- Colony. Locus AG2 sample 01 (0353)**

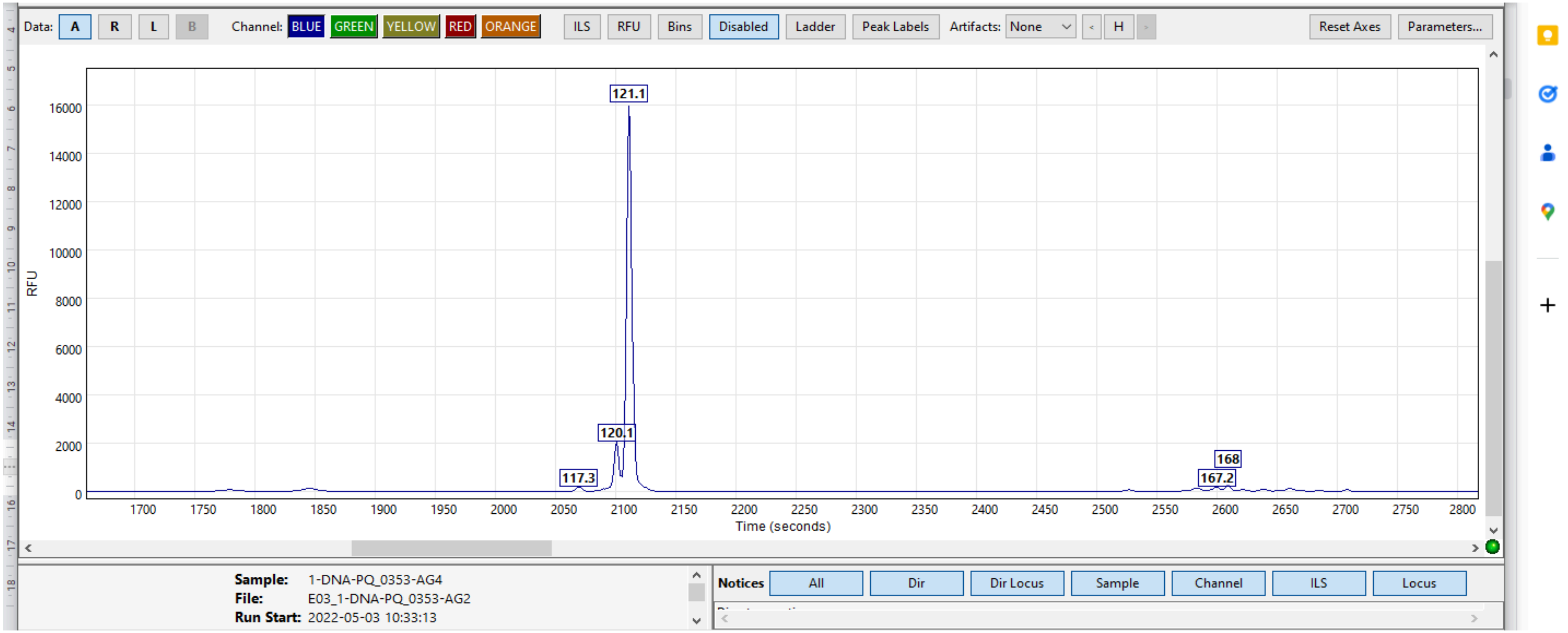


| | |
|---|---|
| **Observer 1** | **121 base pairs** |
| **Observer 2** | **121.1 base pairs** |
| **Observer 3** | **121 base pairs** |

## 2- Colony. Locus AG2 sample 04 (0354)

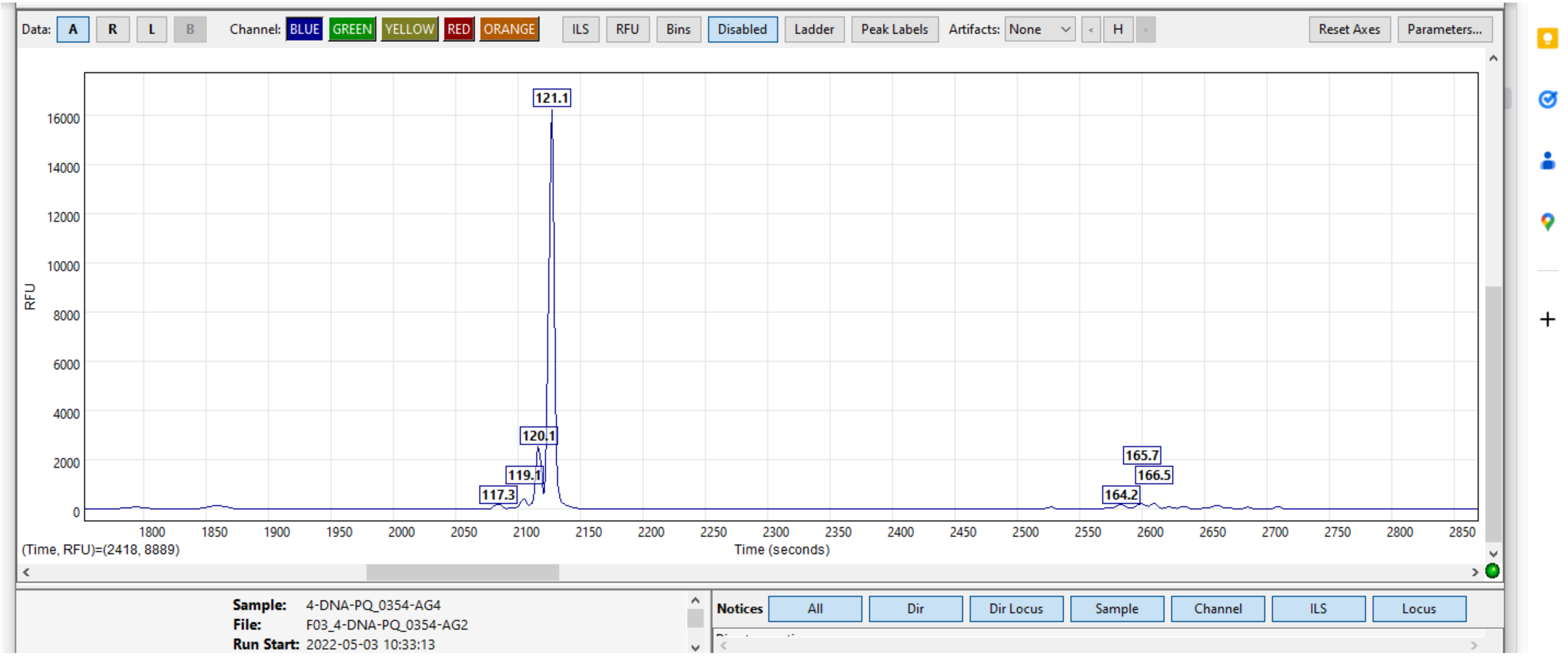


| | |
|---|---|
| **Observer 1** | 121 |
| **Observer 2** | 121.1 |
| **Observer 3** | 121 |

## 3- Colony. Locus AG2 sample 05 (0355)

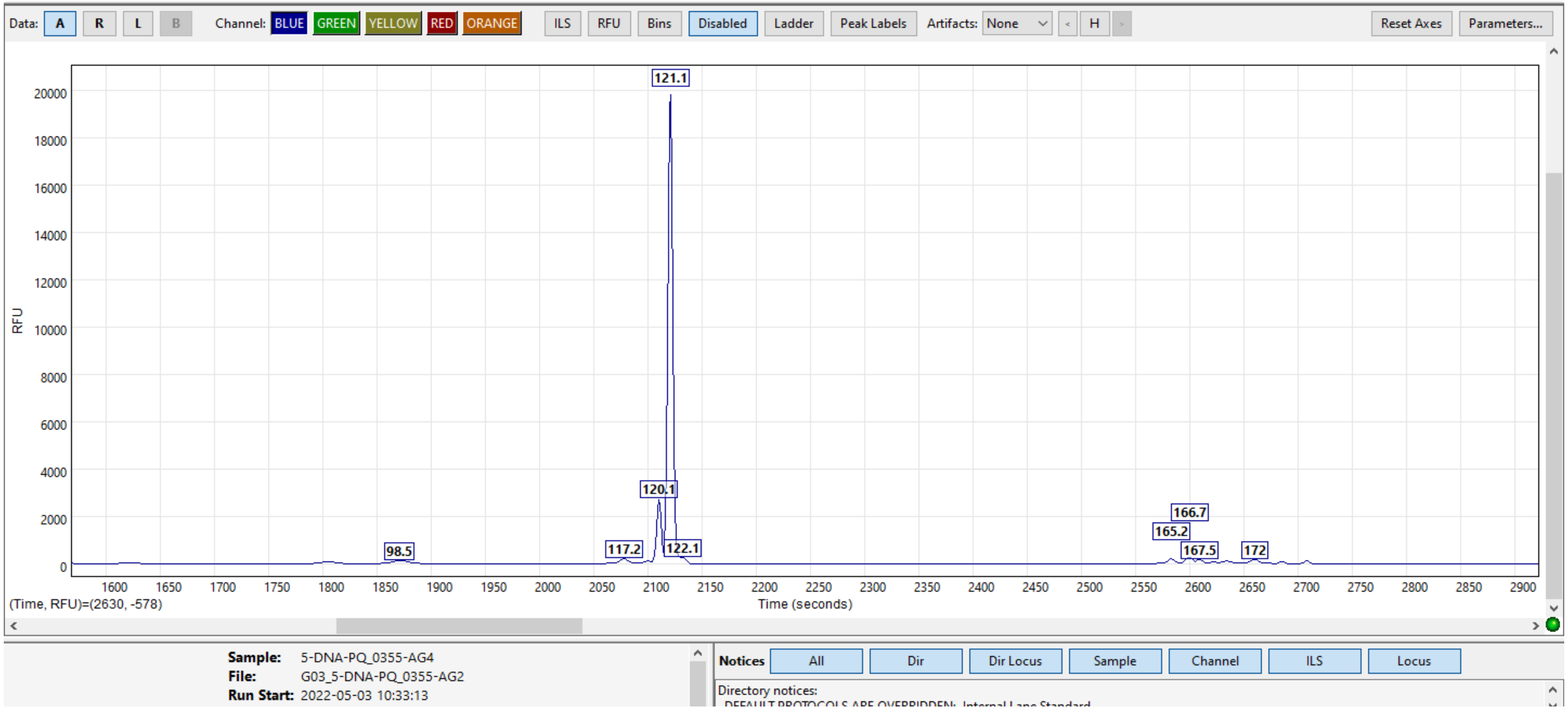


| | |
|---|---|
| **Observer 1** | **121** |
| **Observer 2** | **121.1** |
| **Observer 3** | **121** |

## 4- Colony. Locus AG2 sample 06 (0356)

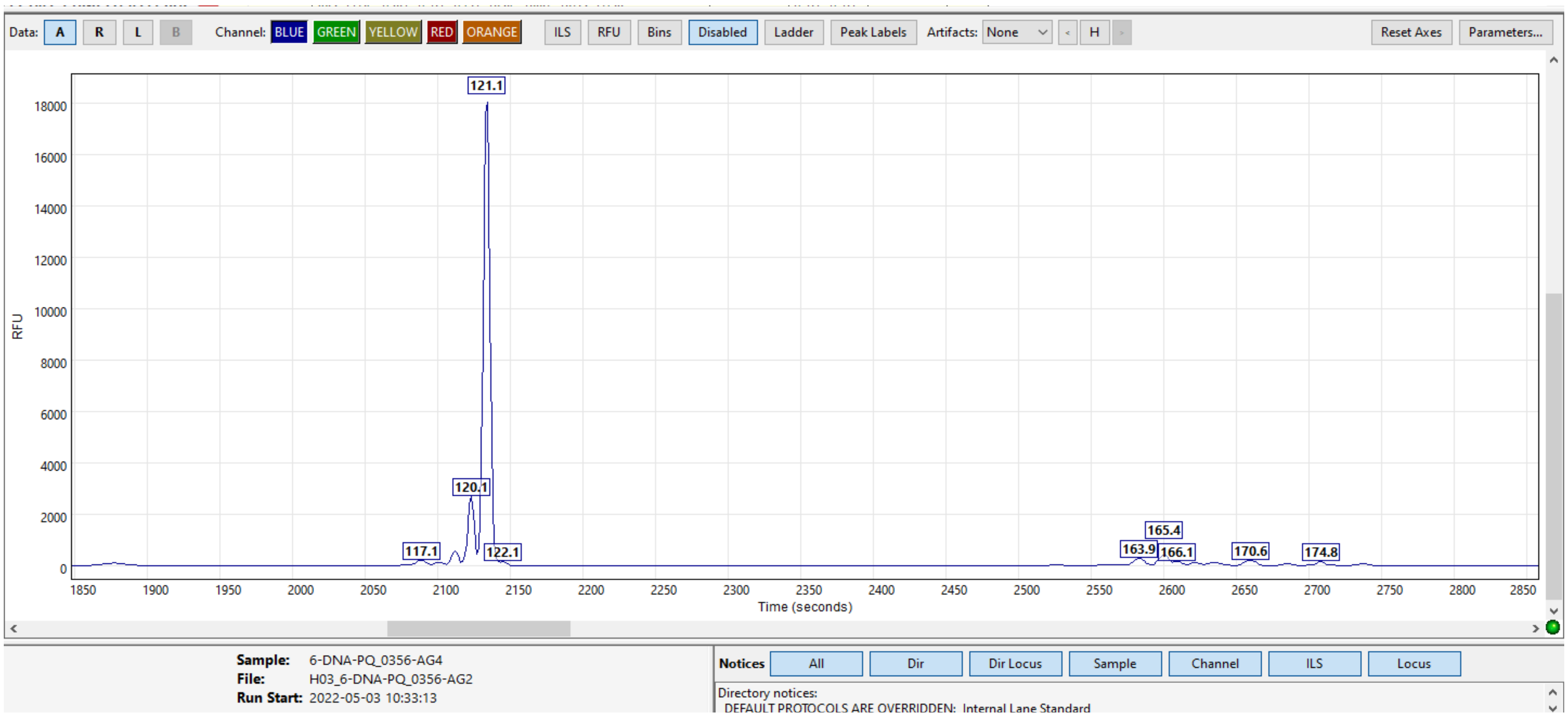

**5- Colony. Locus AG2 sample 07 (0357)**

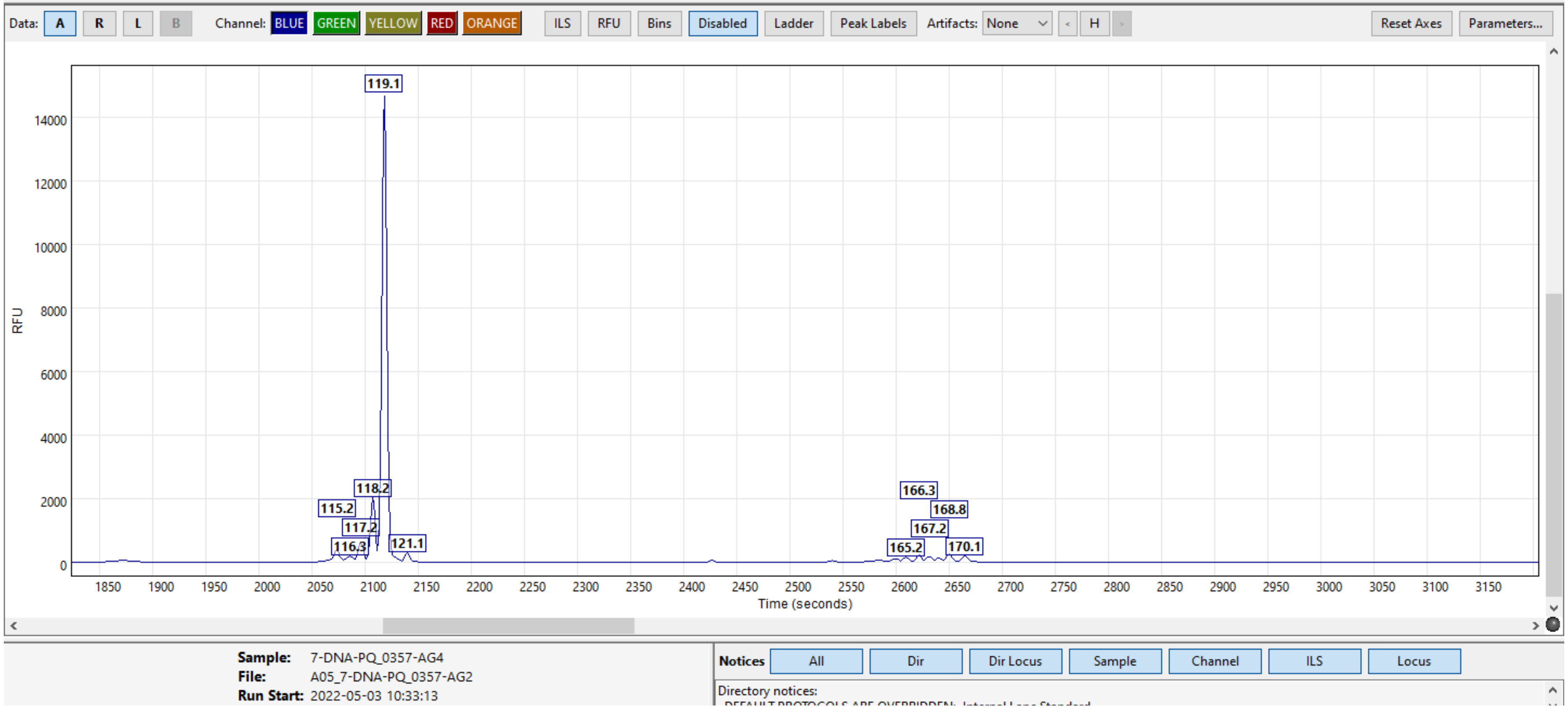


| | |
|---|---|
| **Observer 1** | **119** |
| **Observer 2** | **119.1** |
| **Observer 3** | **119** |

## 6- Colony. Locus AG2 sample 08 (0358)

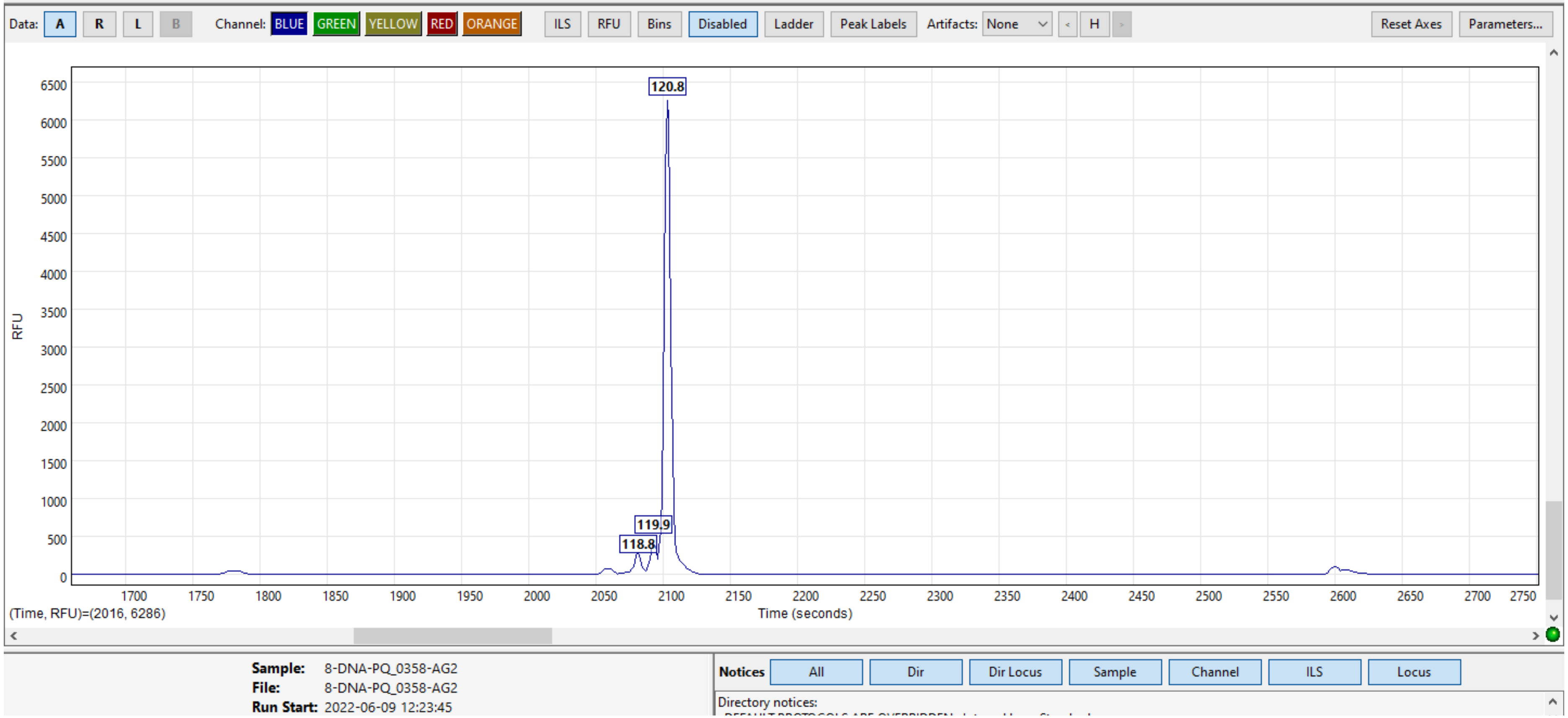


| | |
|---|---|
| **Observer 1** | **121** |
| **Observer 2** | **120.8** |
| **Observer 3** | **121** |

## 7- Colony. Locus AG2 sample 10 (0359)

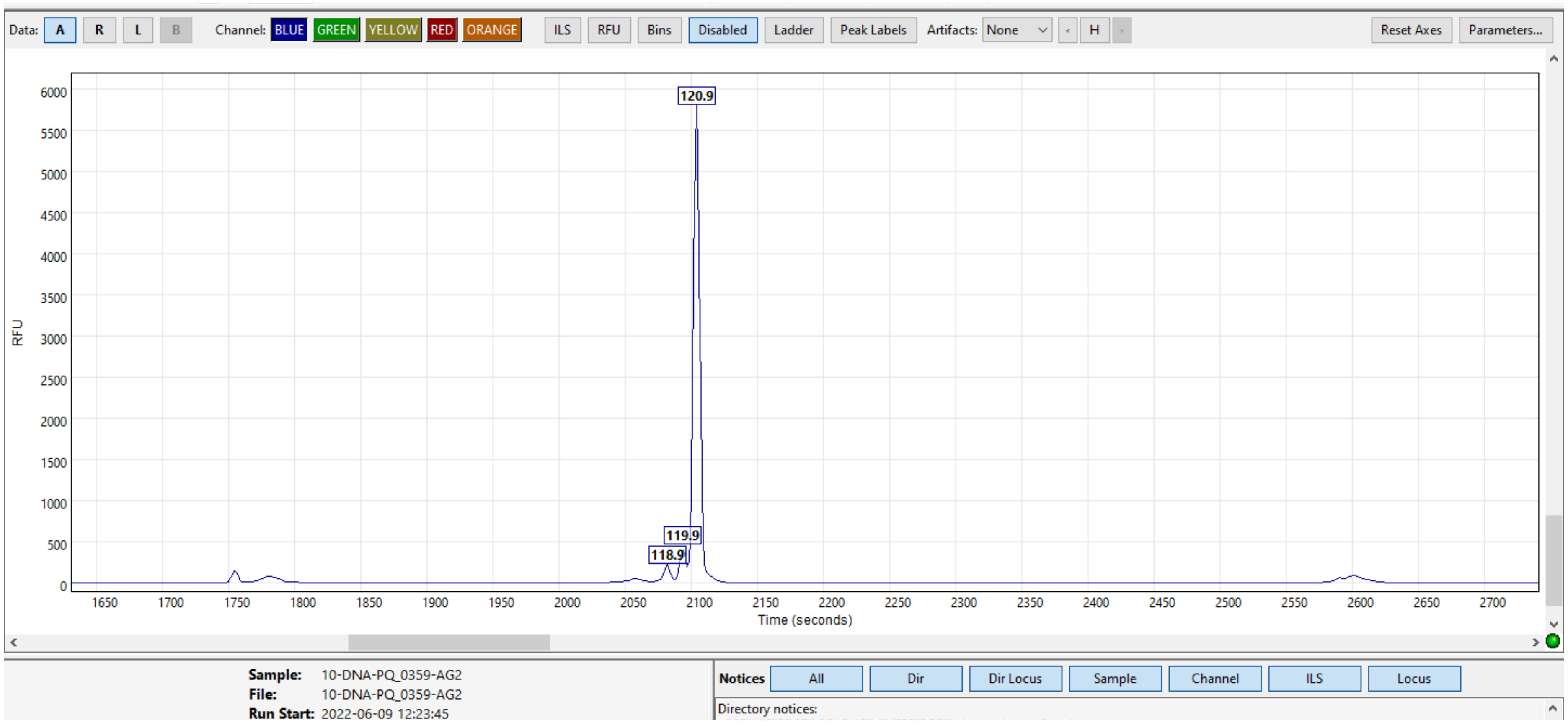


| | |
|---|---|
| **Observer 1** | **121** |
| **Observer 2** | **120.9** |
| **Observer 3** | **121** |

## 8- Colony. Locus AG2 sample 11 (0360)

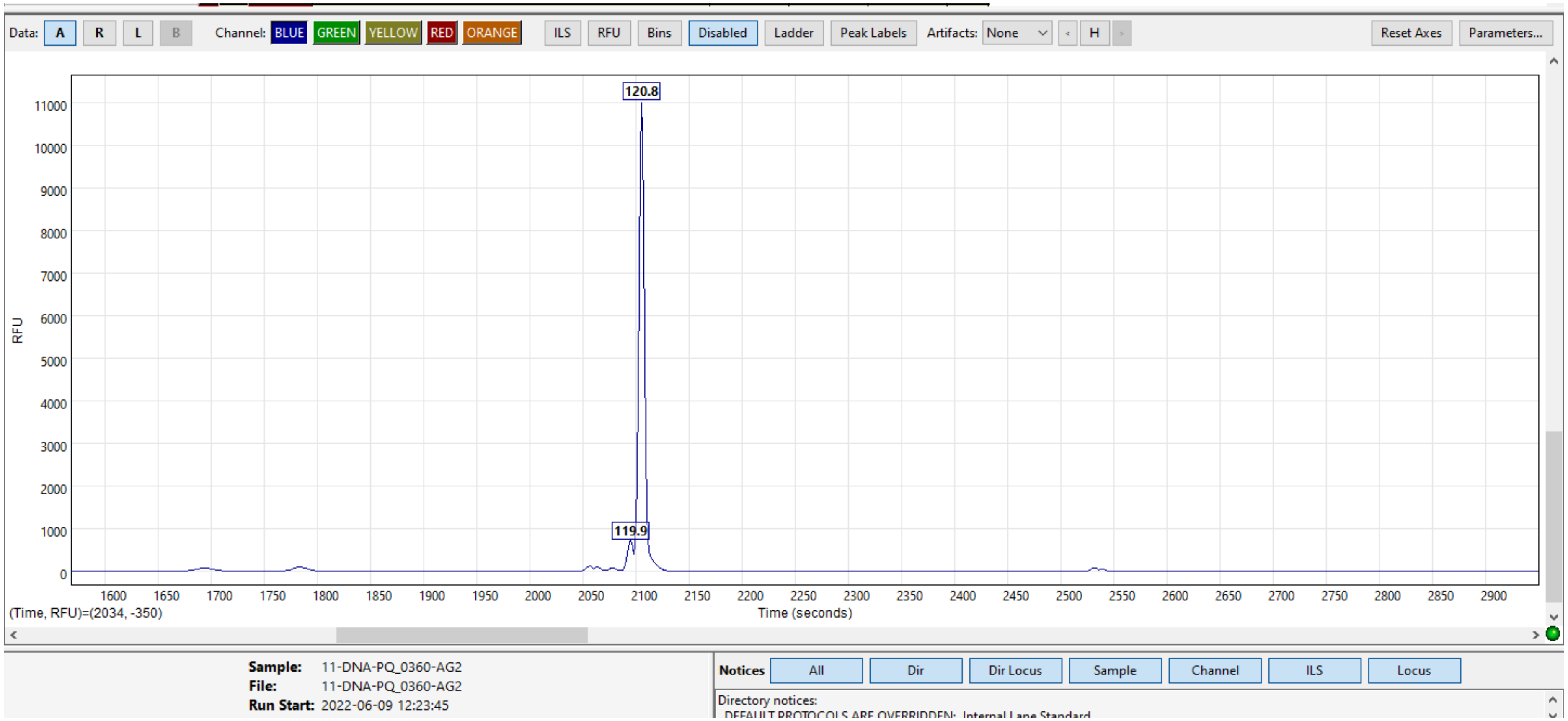


| | |
|---|---|
| **Observer 1** | **121** |
| **Observer 2** | **120.8** |
| **Observer 3** | **121** |

### 9- Colony. Locus AG2 sample 12 (0361)

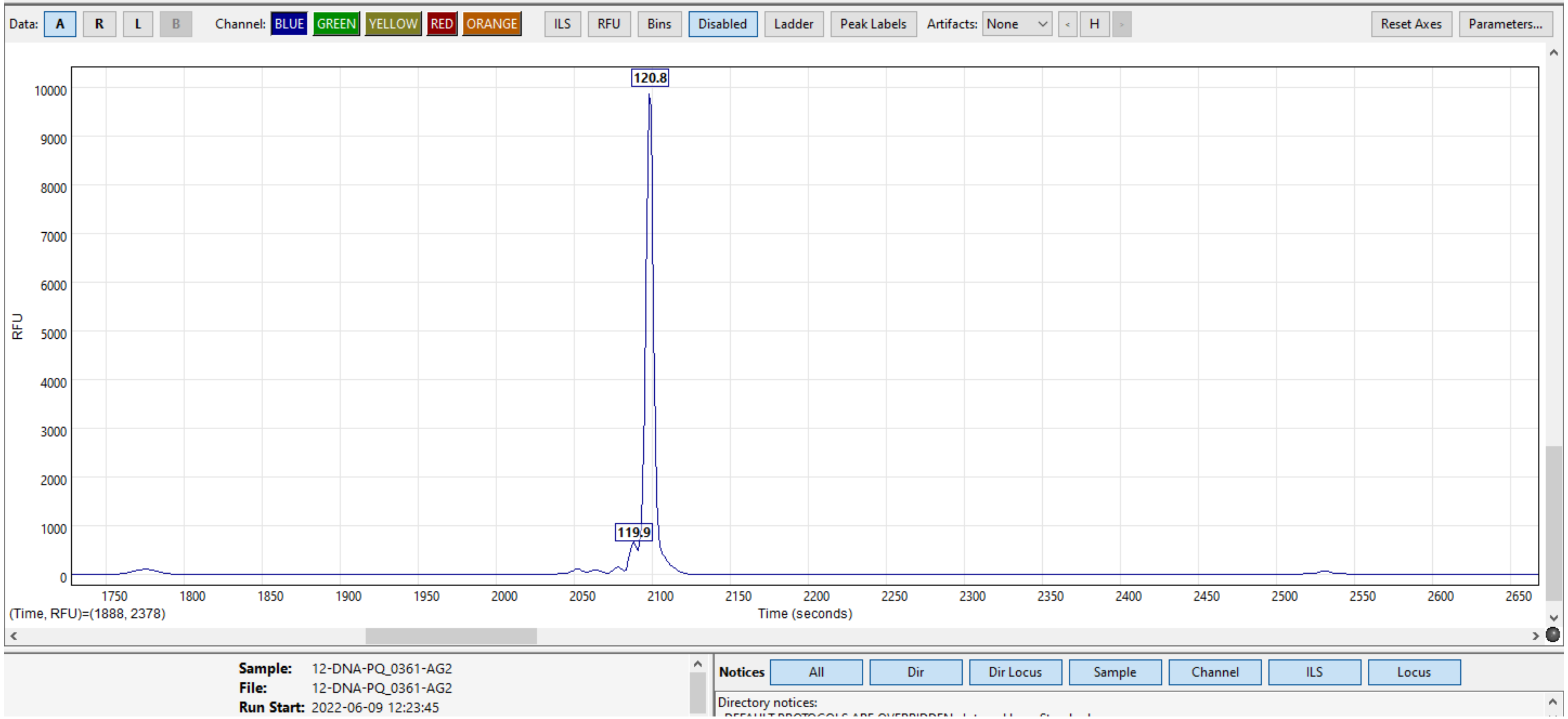


| | |
|---|---|
| **Observer 1** | **121** |
| **Observer 2** | **120.8** |
| **Observer 3** | **121** |

## 10- Colony. Locus AG2 sample 13 (0362)

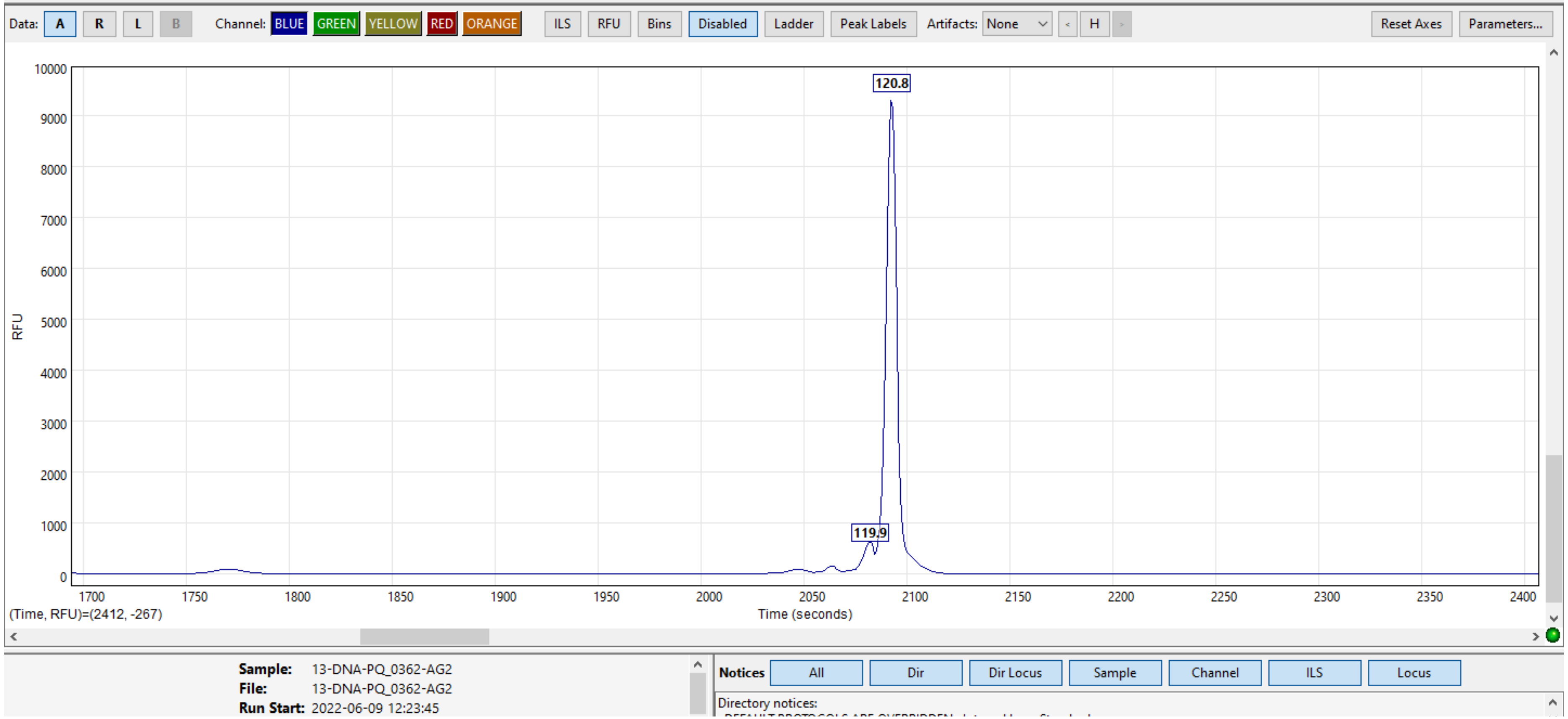


| | |
|---|---|
| **Observer 1** | **121** |
| **Observer 2** | **120.8** |
| **Observer 3** | **121** |

## 11- Colony. Locus AG2 sample 14 (0363)

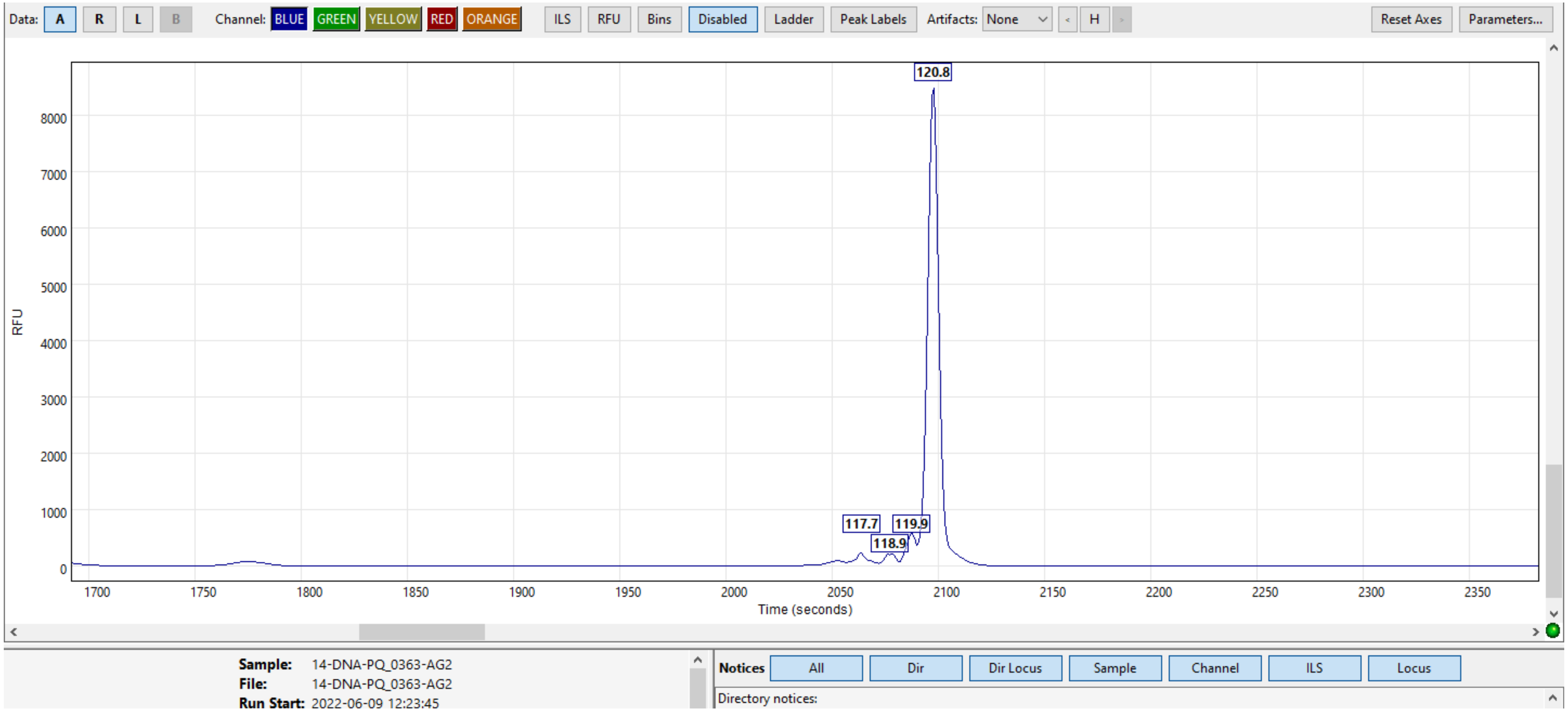


| | |
|---|---|
| **Observer 1** | **121** |
| **Observer 2** | **120.8** |
| **Observer 3** | **121** |

**12- Colony. Locus AG2 sample 16 (0364)**

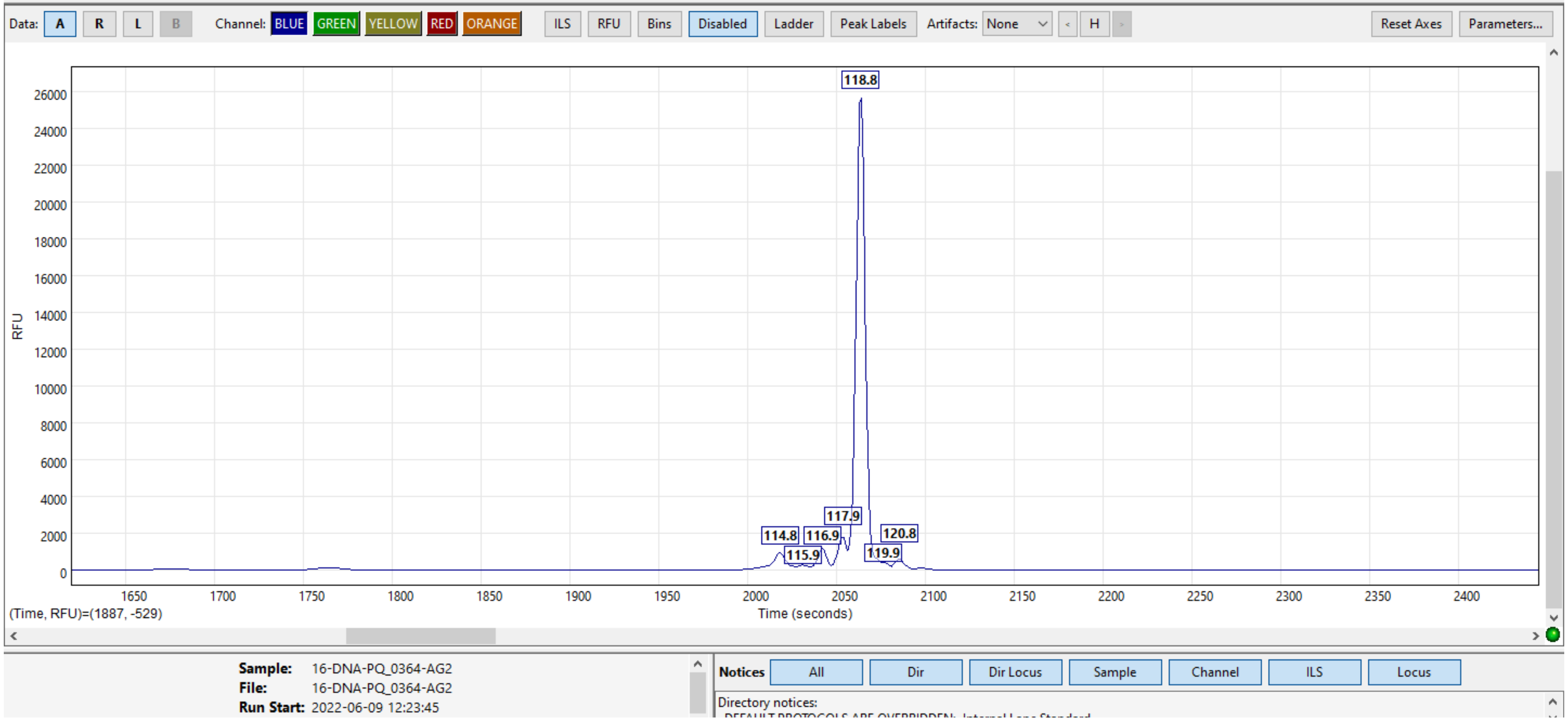


| | |
|---|---|
| **Observer 1** | **119** |
| **Observer 2** | **118.8** |
| **Observer 3** | **119** |

## 13- Colony. Locus AG2 sample 17 (0365)

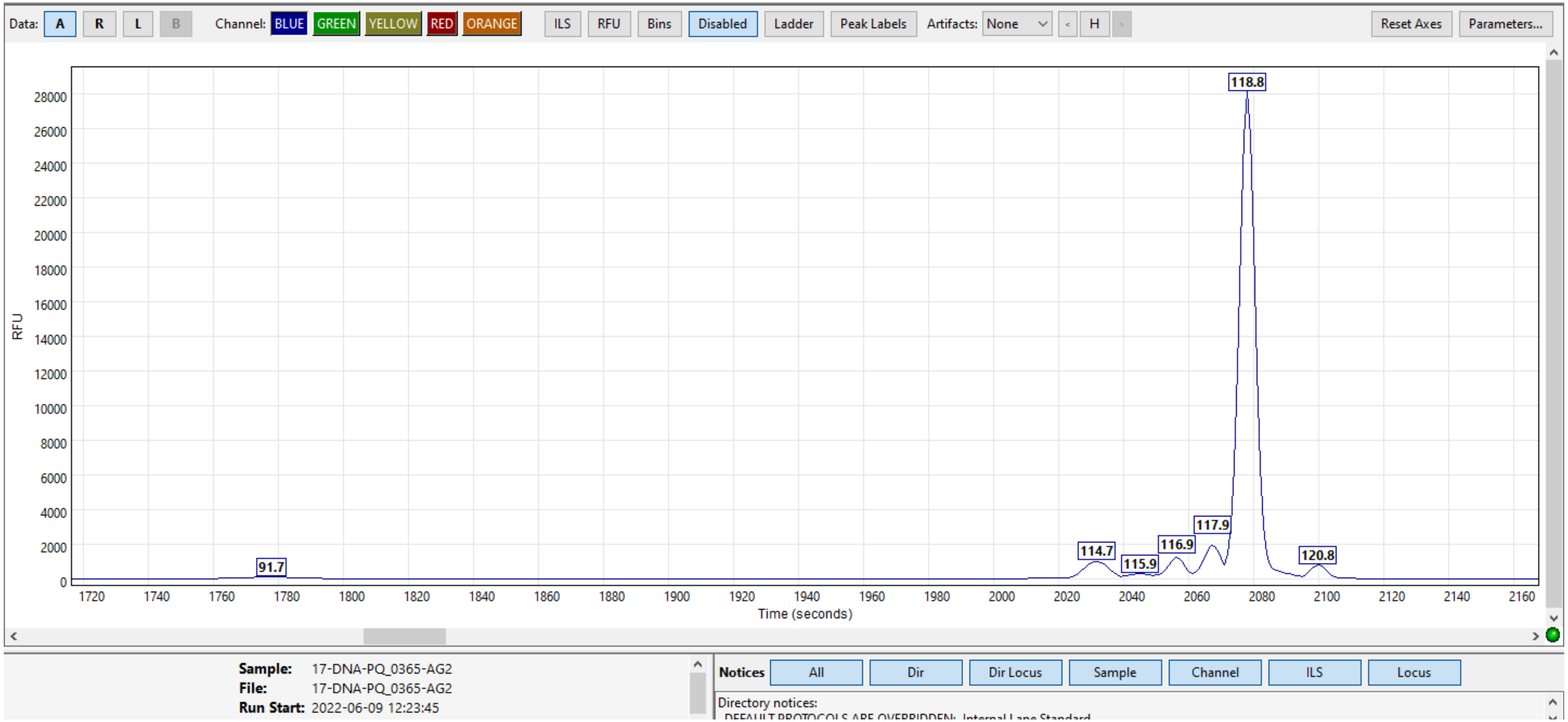


| | |
|---|---|
| **Observer 1** | **119** |
| **Observer 2** | **118.8** |
| **Observer 3** | **119** |

## 14- Colony. Locus AG2 sample 18 (0366)

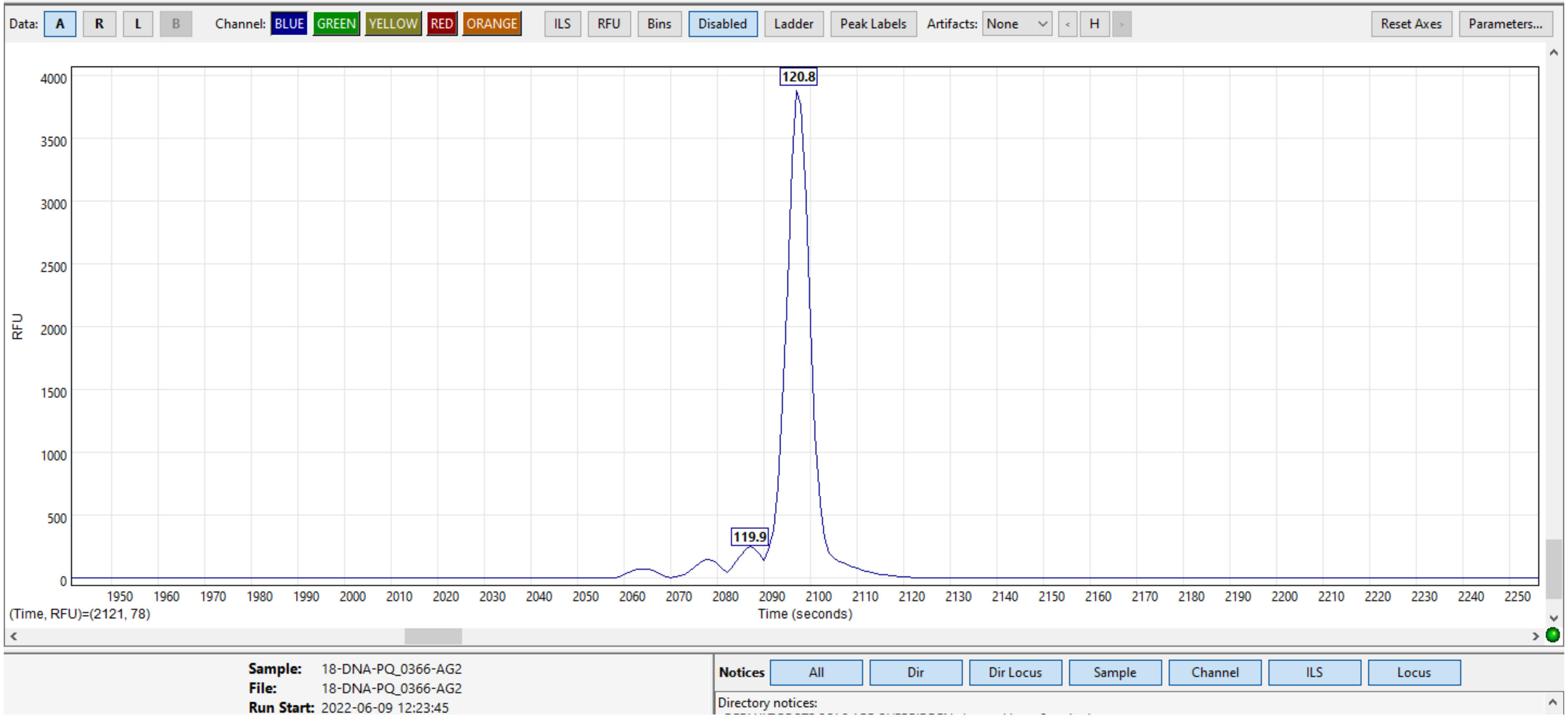


| | |
|---|---|
| **Observer 1** | **121** |
| **Observer 2** | **120.8** |
| **Observer 3** | **121** |

**15- Colony. Locus AG2 sample 20 (0923)**

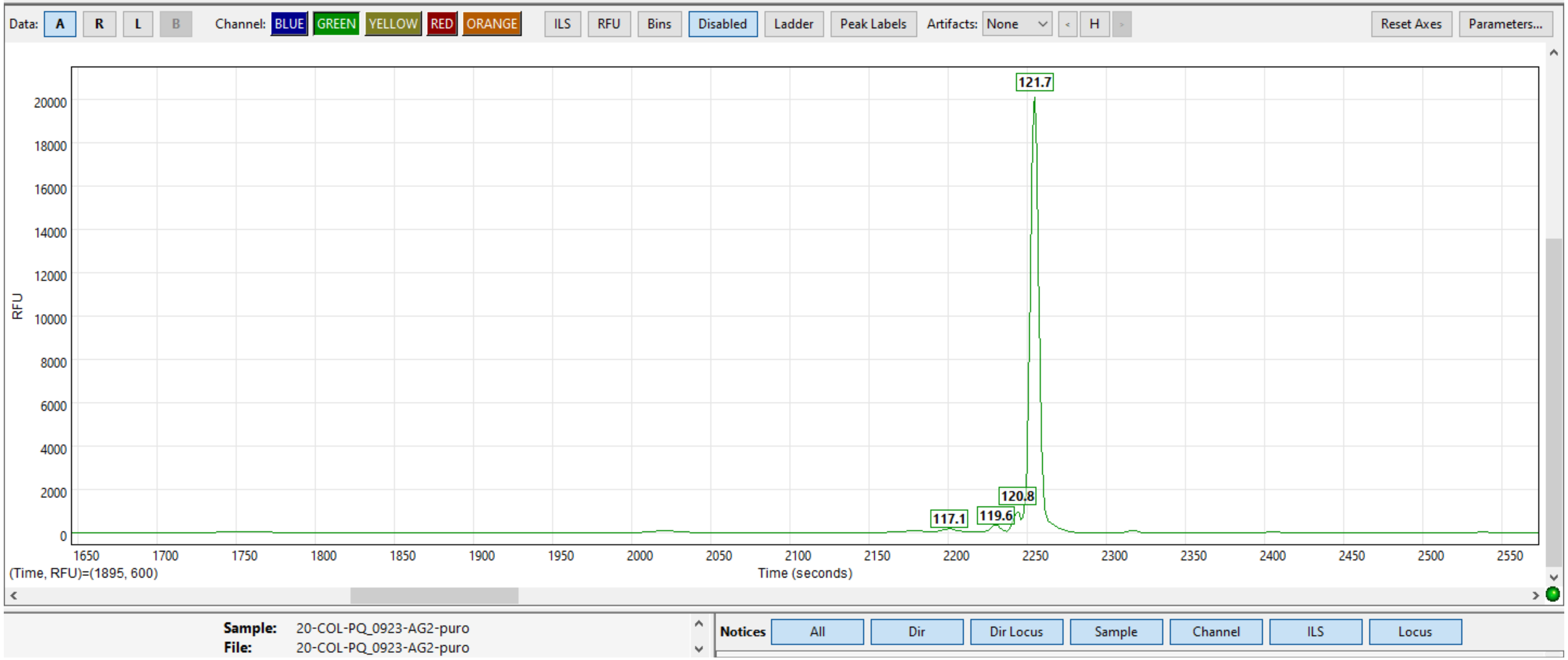


| | |
|---|---|
| **Observer 1** | **122** |
| **Observer 2** | **121.7** |
| **Observer 3** | **122** |

**AG4**

**01- Colony. Locus AG4 sample 01 (0353)**

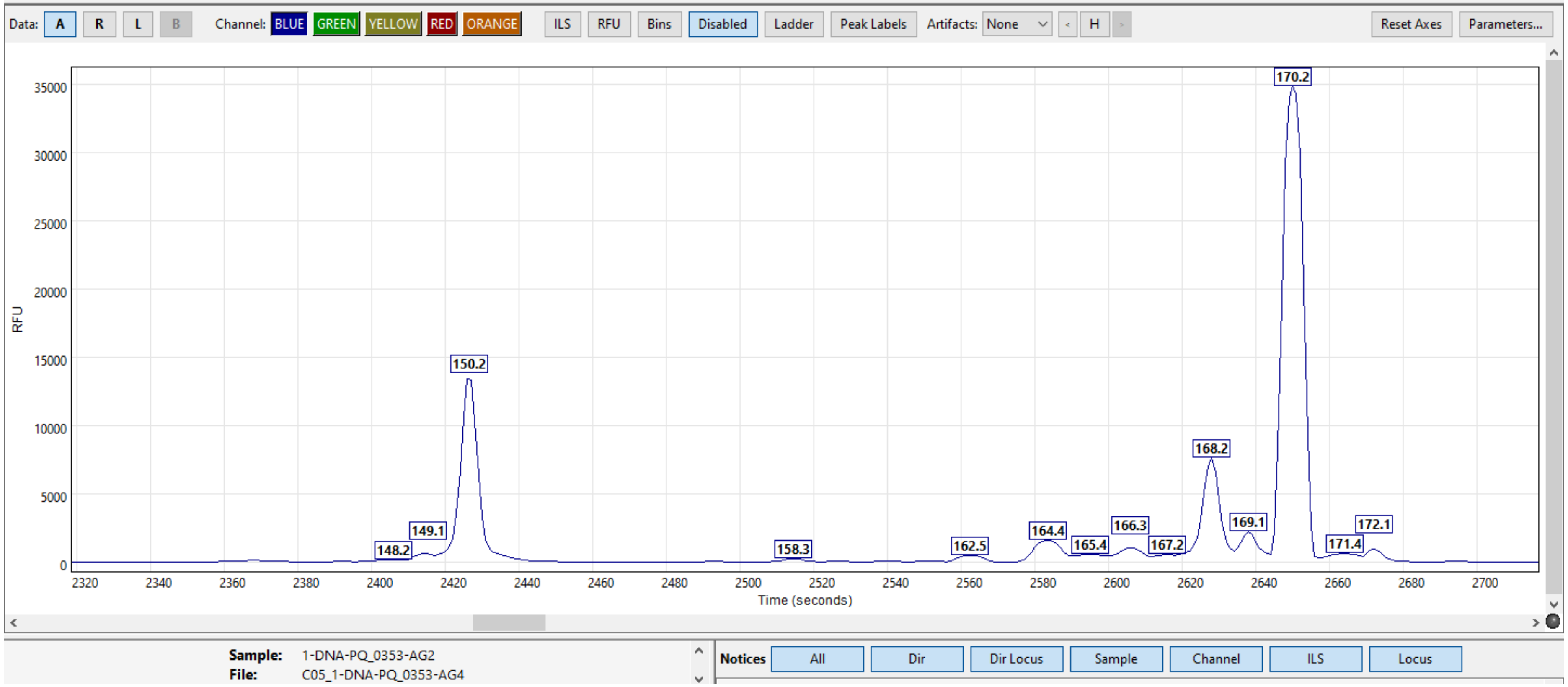


| | |
|---|---|
| **Observer 1** | **150;170** |
| **Observer 2** | **150.2;170.2** |
| **Observer 3** | **150;170** |

## 2- Colony. Locus AG4 sample 04 (0354)

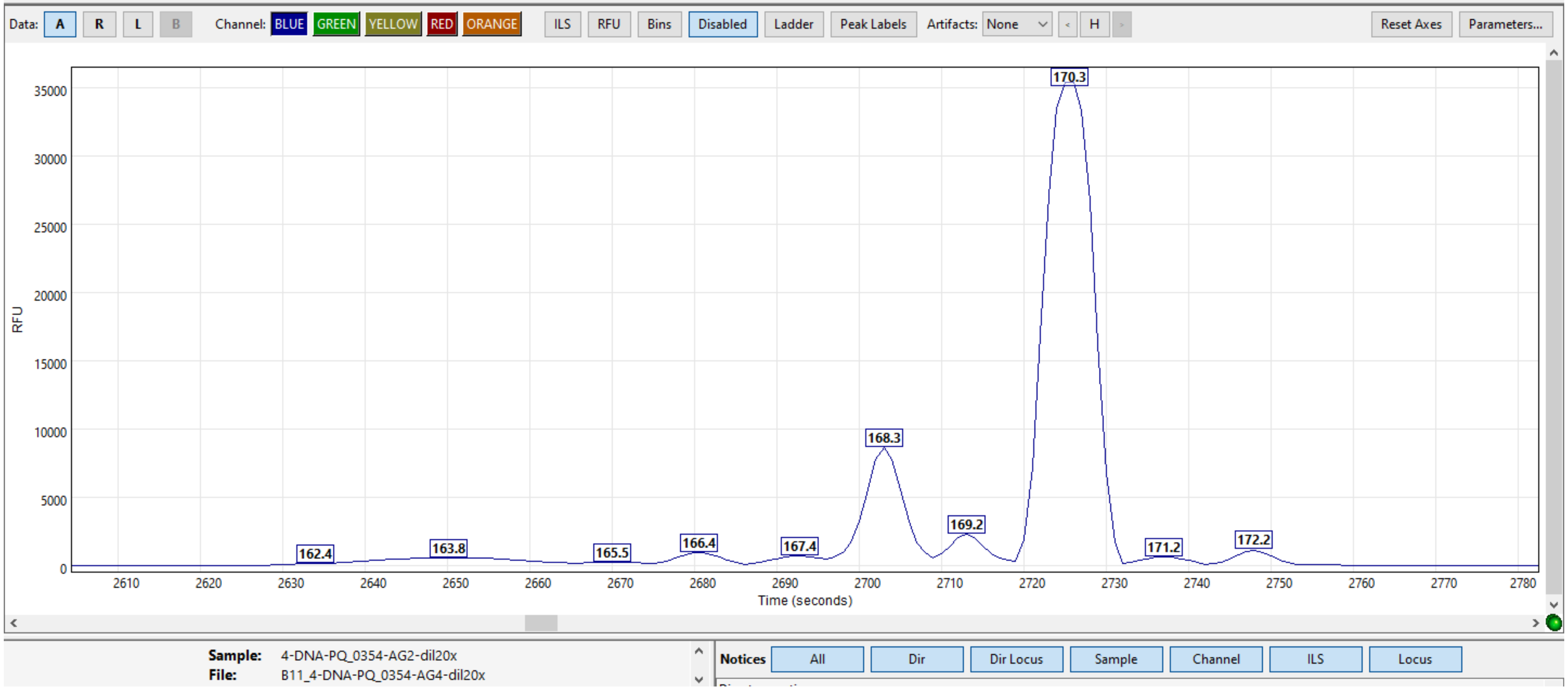


| | |
|---|---|
| **Observer 1** | **170** |
| **Observer 2** | **168.3; 170.3** |
| **Observer 3** | **170** |

### 3- Colony. Locus AG4 sample 05 (0355)

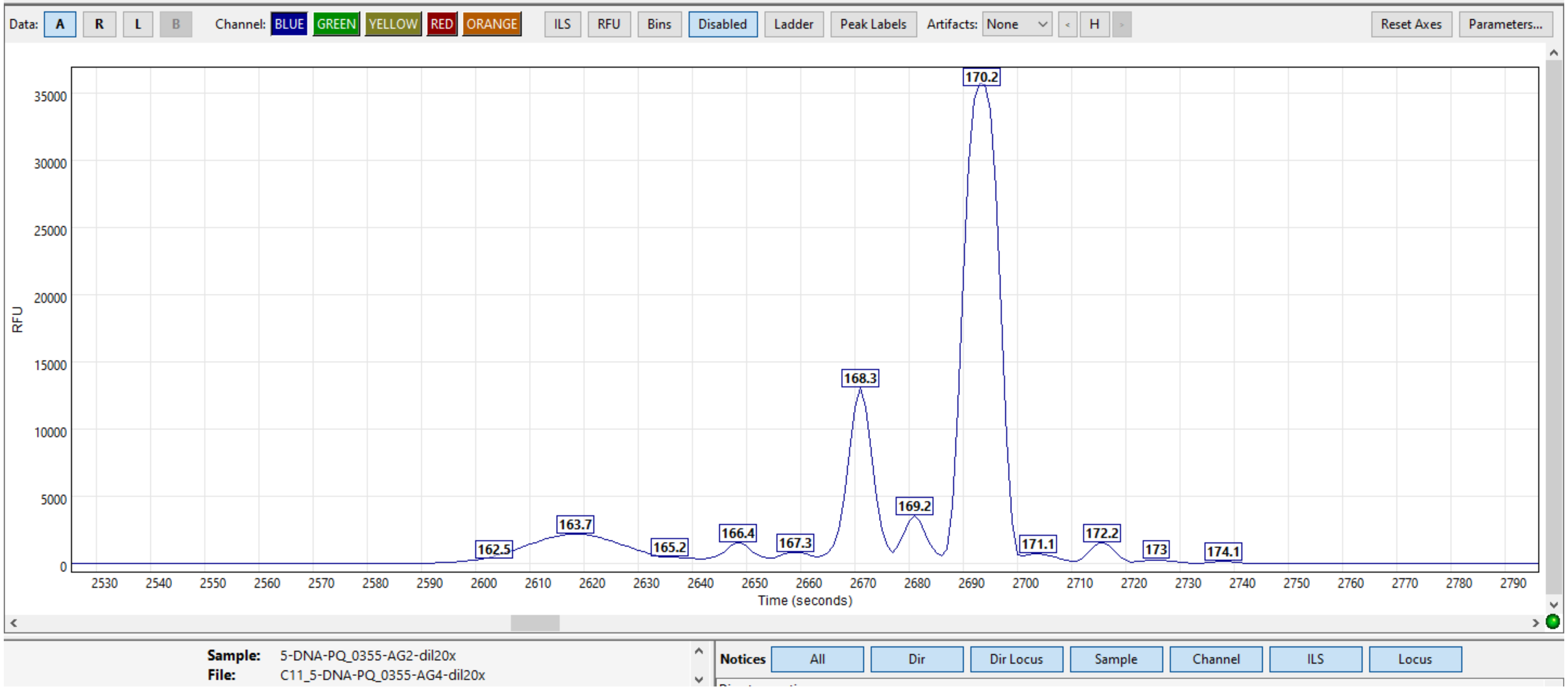


| | |
|---|---|
| **Observer 1** | **170** |
| **Observer 2** | **168.3 ; 170.2** |
| **Observer 3** | **168;170** |

### 4- Colony. Locus AG4 sample 06 (0356)

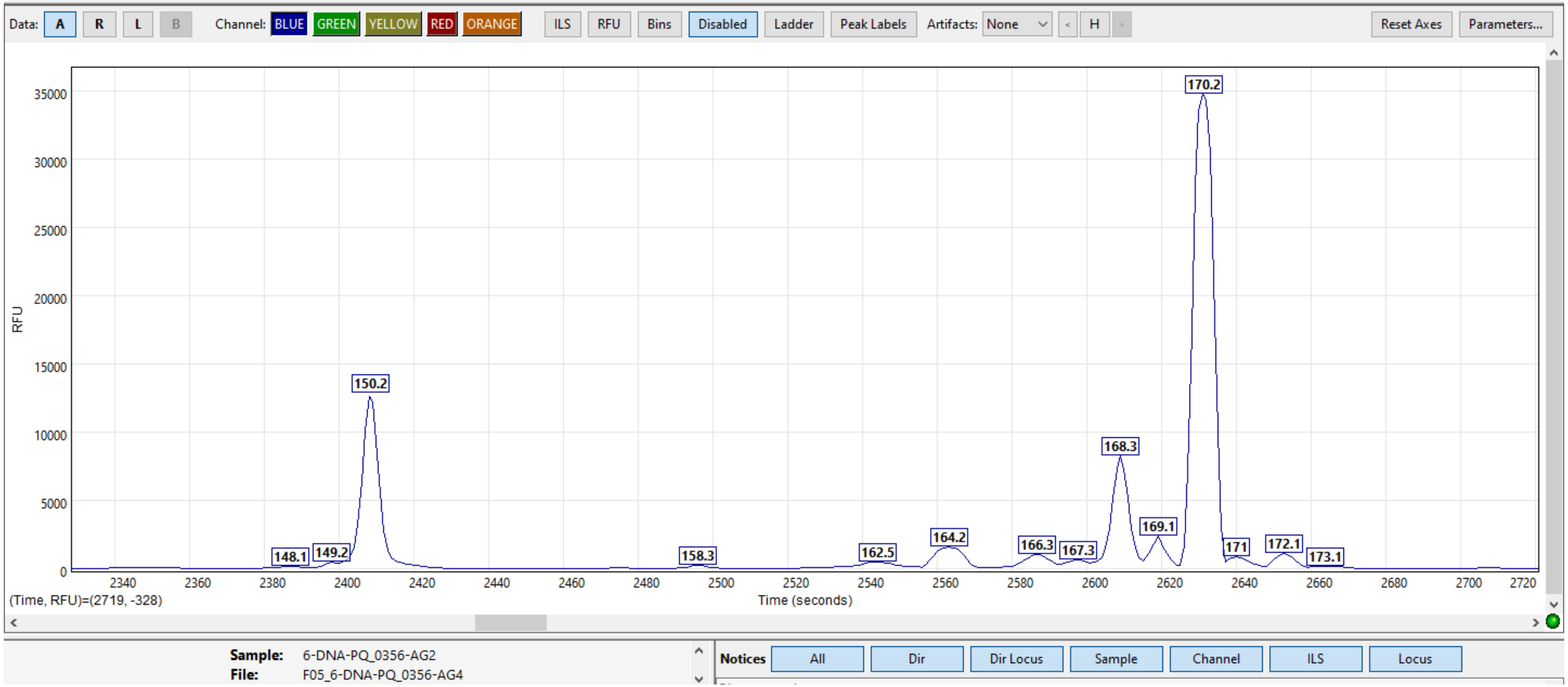


| | |
|---|---|
| **Observer 1** | **150;170** |
| **Observer 2** | **150.2 ; 170.2** |
| **Observer 3** | **150;170** |

## 5- Colony. Locus AG4 sample 07 (0357)

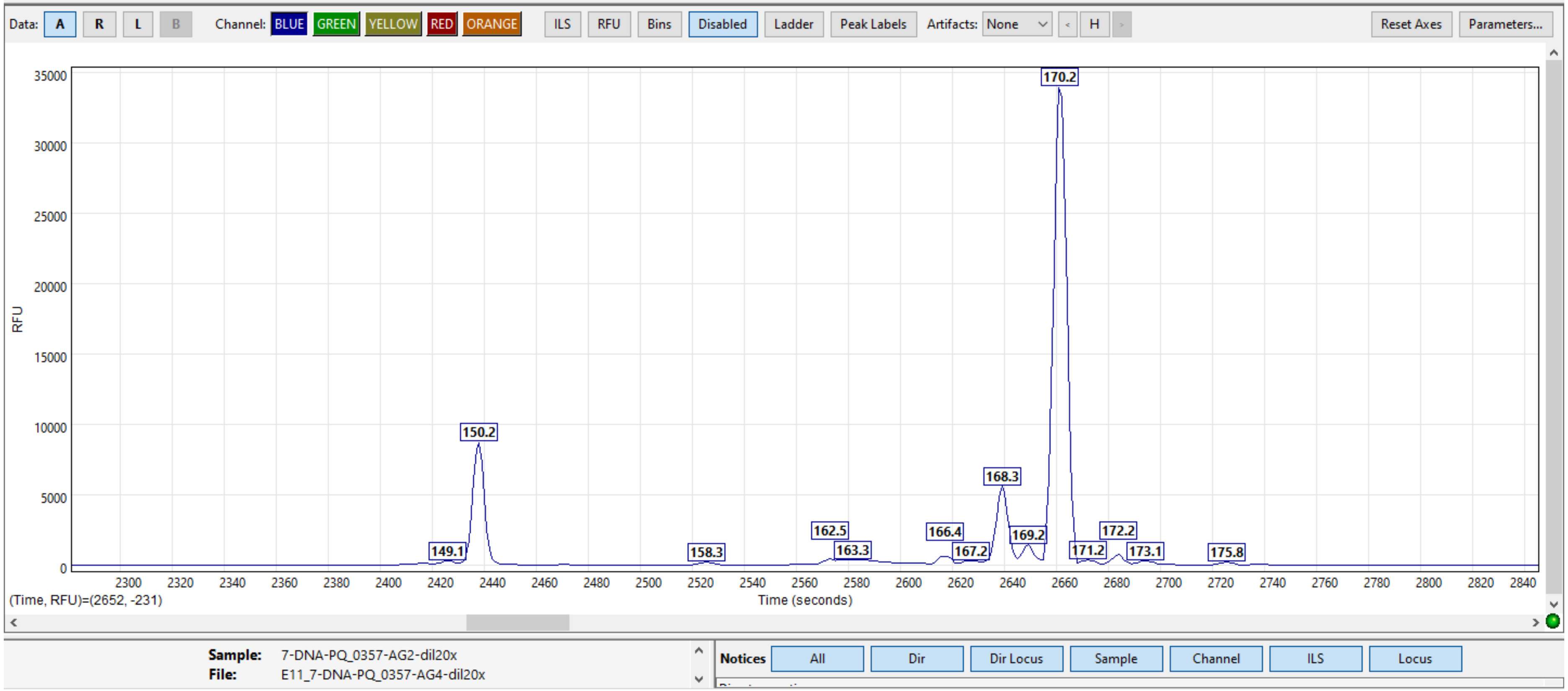


| | |
|---|---|
| **Observer 1** | **150;170** |
| **Observer 2** | **150.2 ; 170.2** |
| **Observer 3** | **150;170** |

## 6- Colony. Locus AG4 sample 08 (0358)

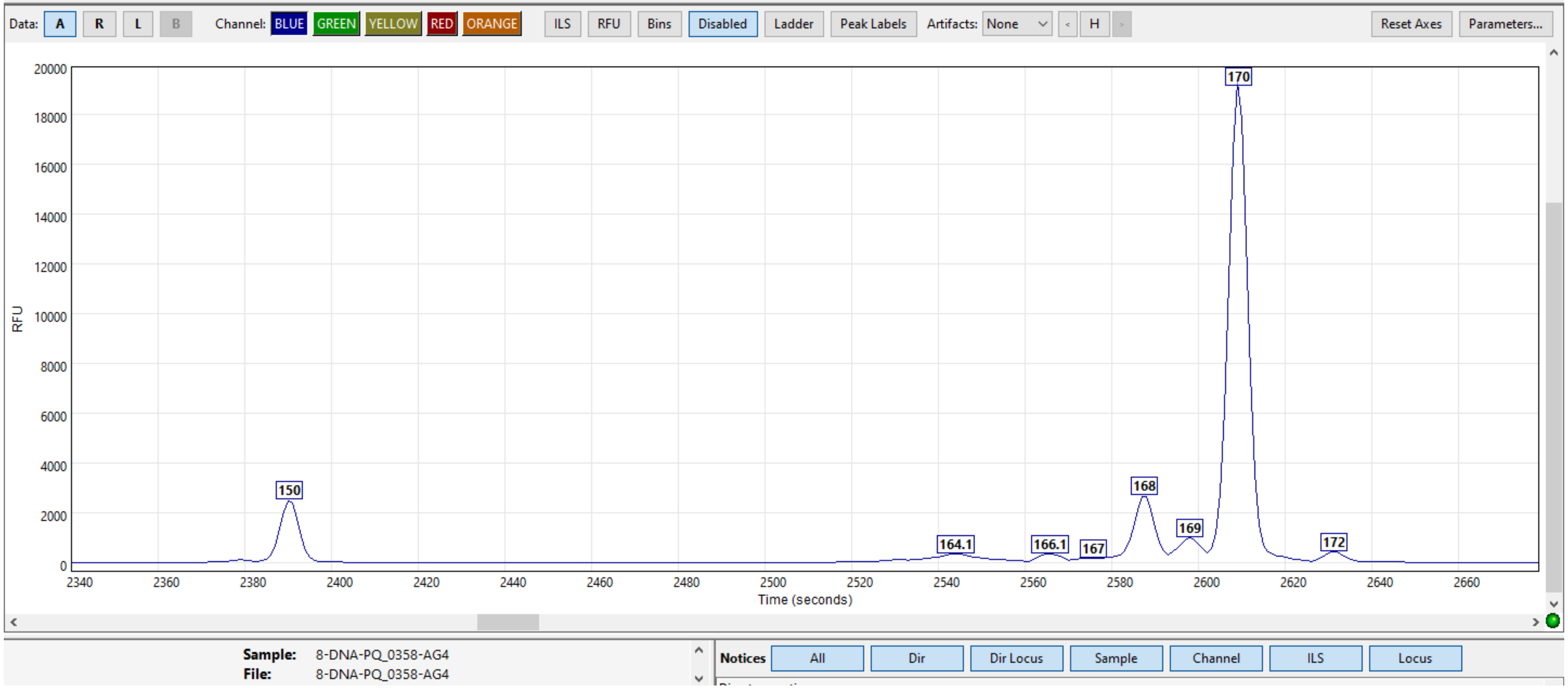


| | |
|---|---|
| **Observer 1** | **150;170** |
| **Observer 2** | **170** |
| **Observer 3** | **170** |

### 7- Colony. Locus AG4 sample 10 (0359)

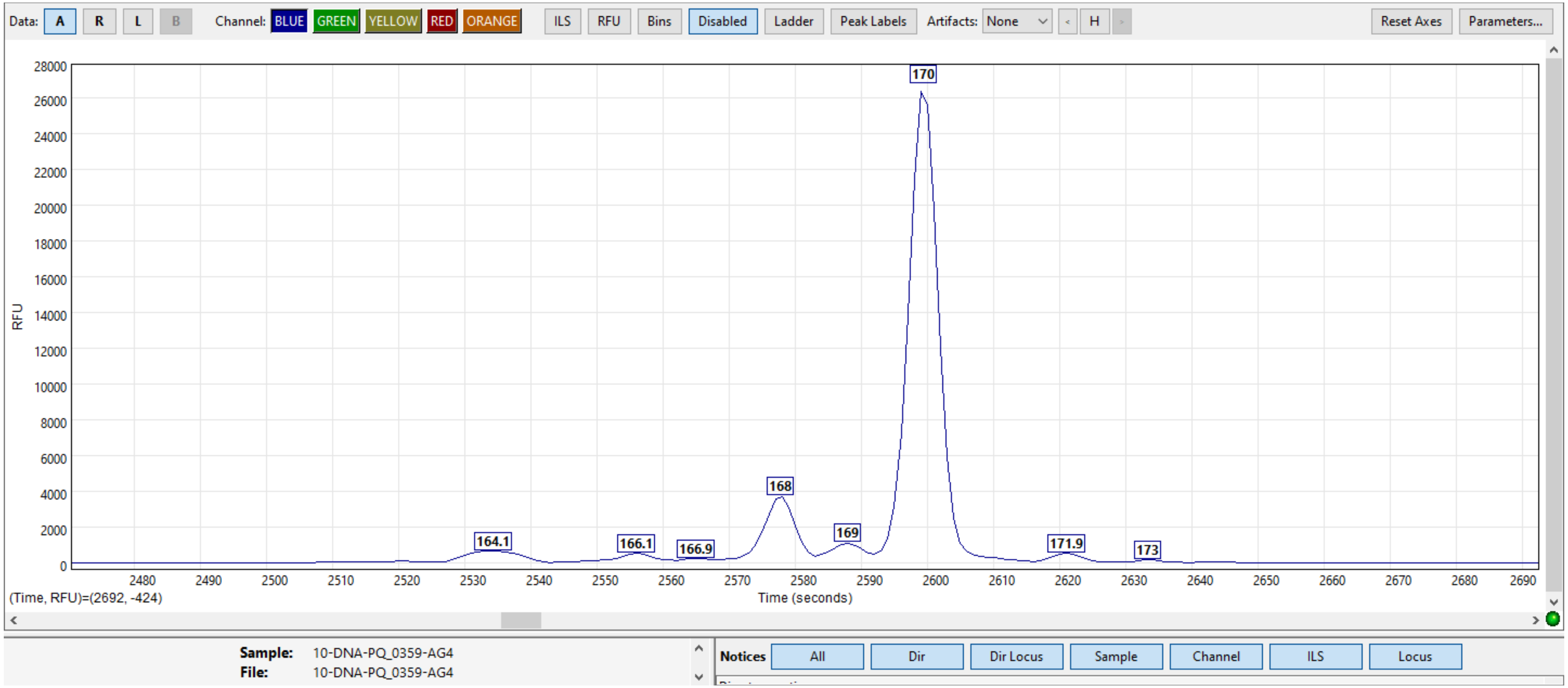


| | |
|---|---|
| **Observer 1** | **170** |
| **Observer 2** | **170** |
| **Observer 3** | **170** |

**8- Colony. Locus AG4 sample 11 (0360)**

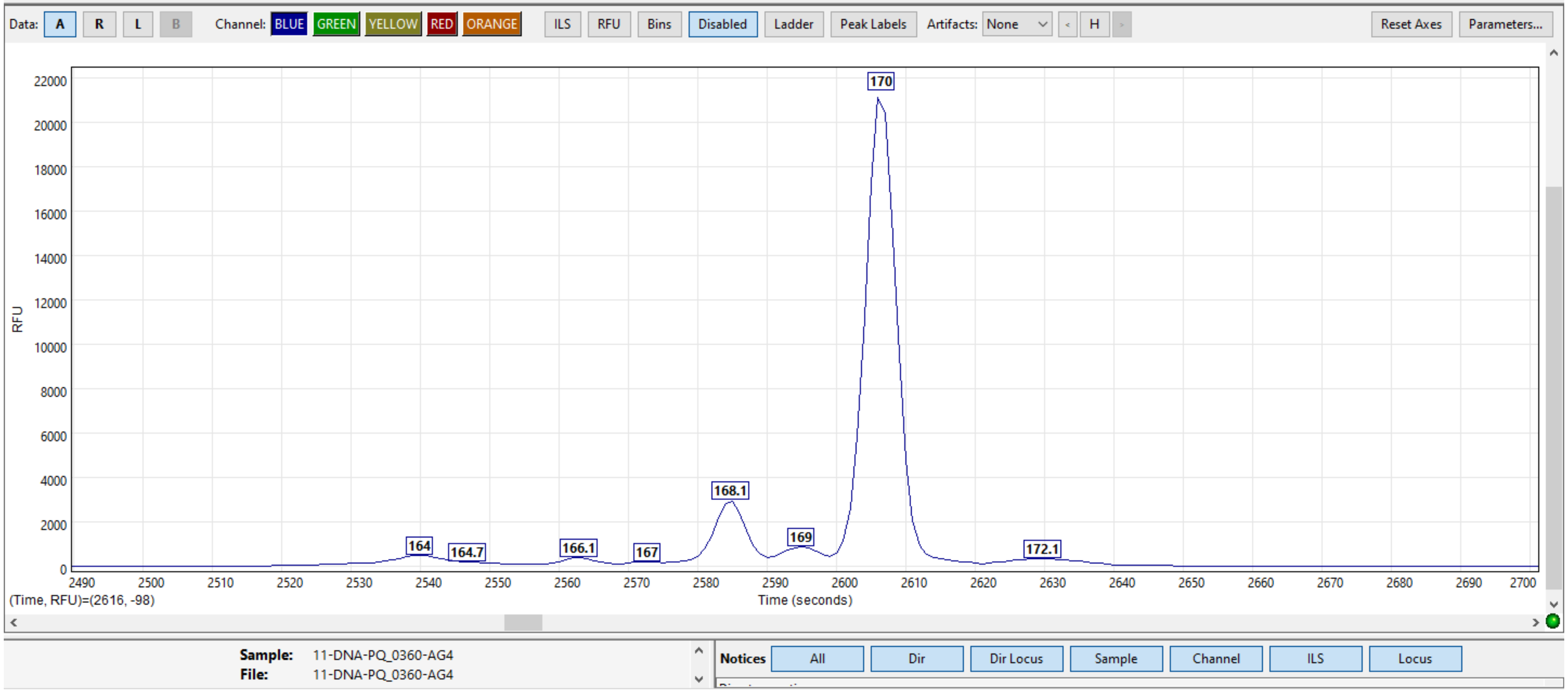


| | |
|---|---|
| **Observer 1** | **170** |
| **Observer 2** | **170** |
| **Observer 3** | **170** |

**9- Colony. Locus AG4 sample 12 (0361)**

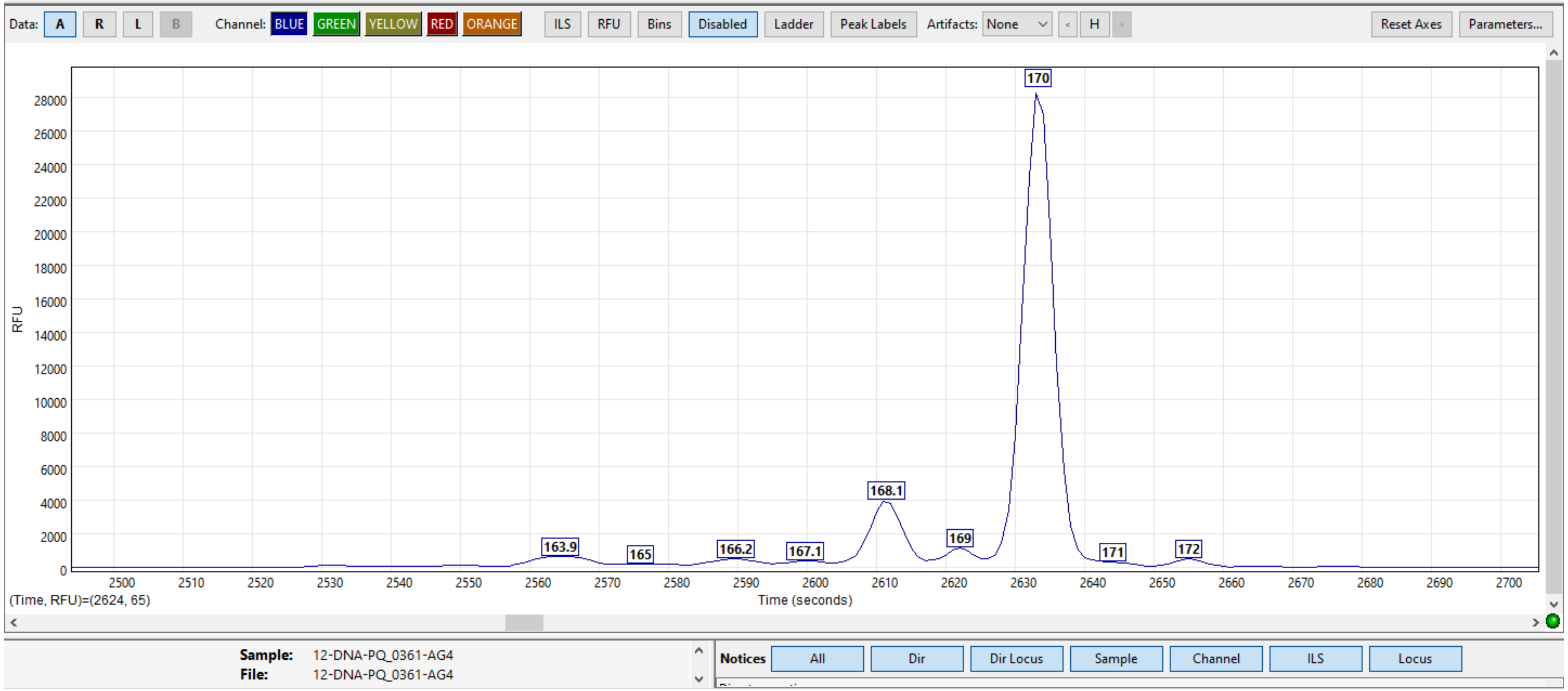


| | |
|---|---|
| **Observer 1** | **170** |
| **Observer 2** | **170** |
| **Observer 3** | **170** |

**10- Colony. Locus AG4 sample 13 (0362)**

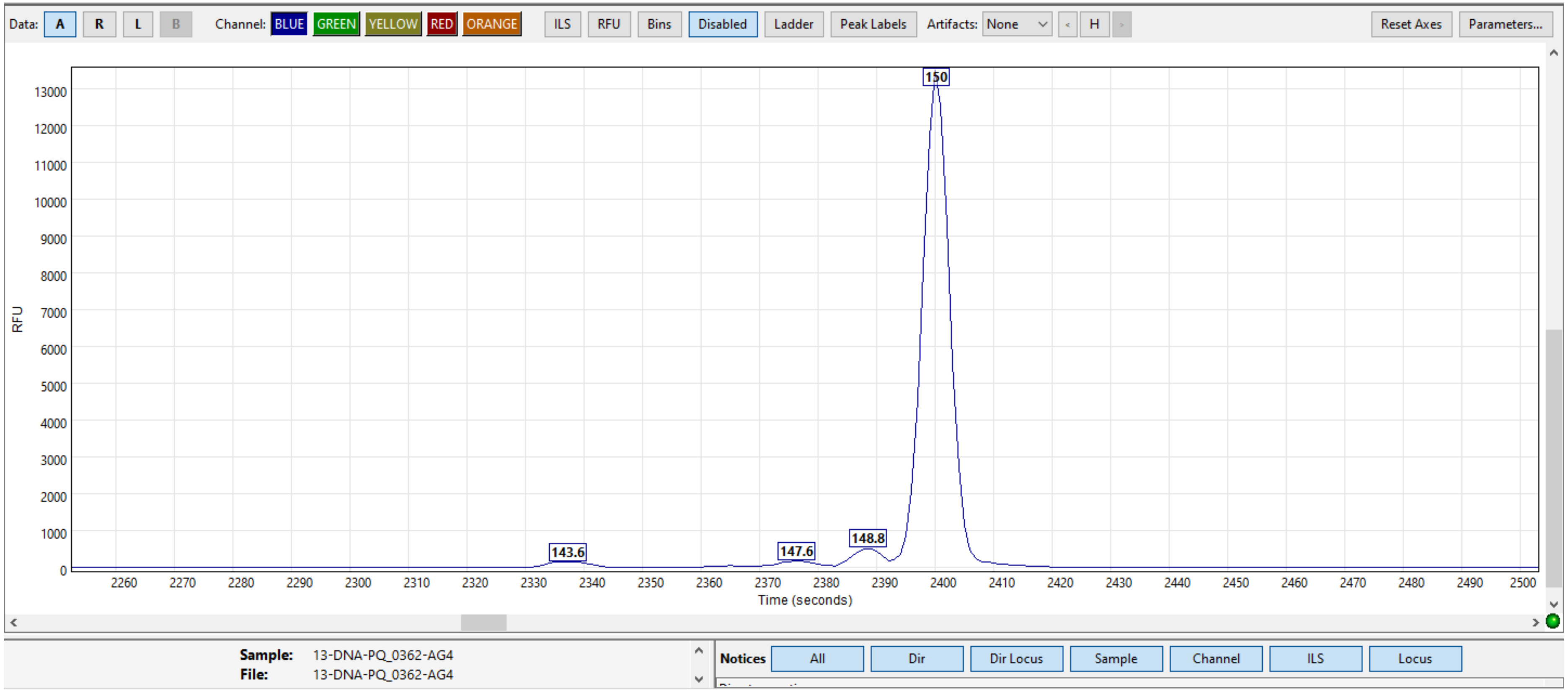


| | |
|---|---|
| **Observer 1** | **150** |
| **Observer 2** | **150** |
| **Observer 3** | **150** |

## 11- Colony. Locus AG4 sample 14 (0363)

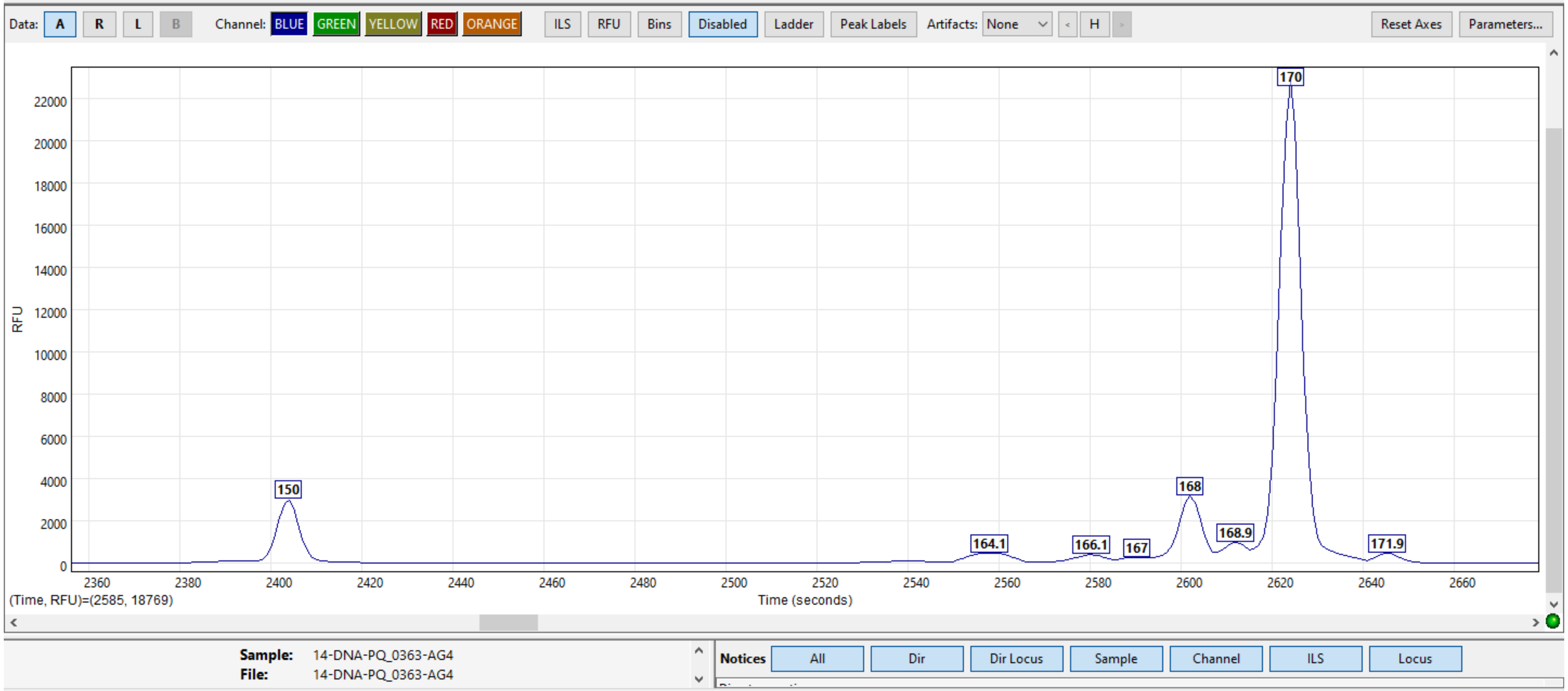


| | |
|---|---|
| **Observer 1** | **150;170** |
| **Observer 2** | **170** |
| **Observer 3** | **170** |

**12- Colony. Locus AG4 sample 16 (0364)**

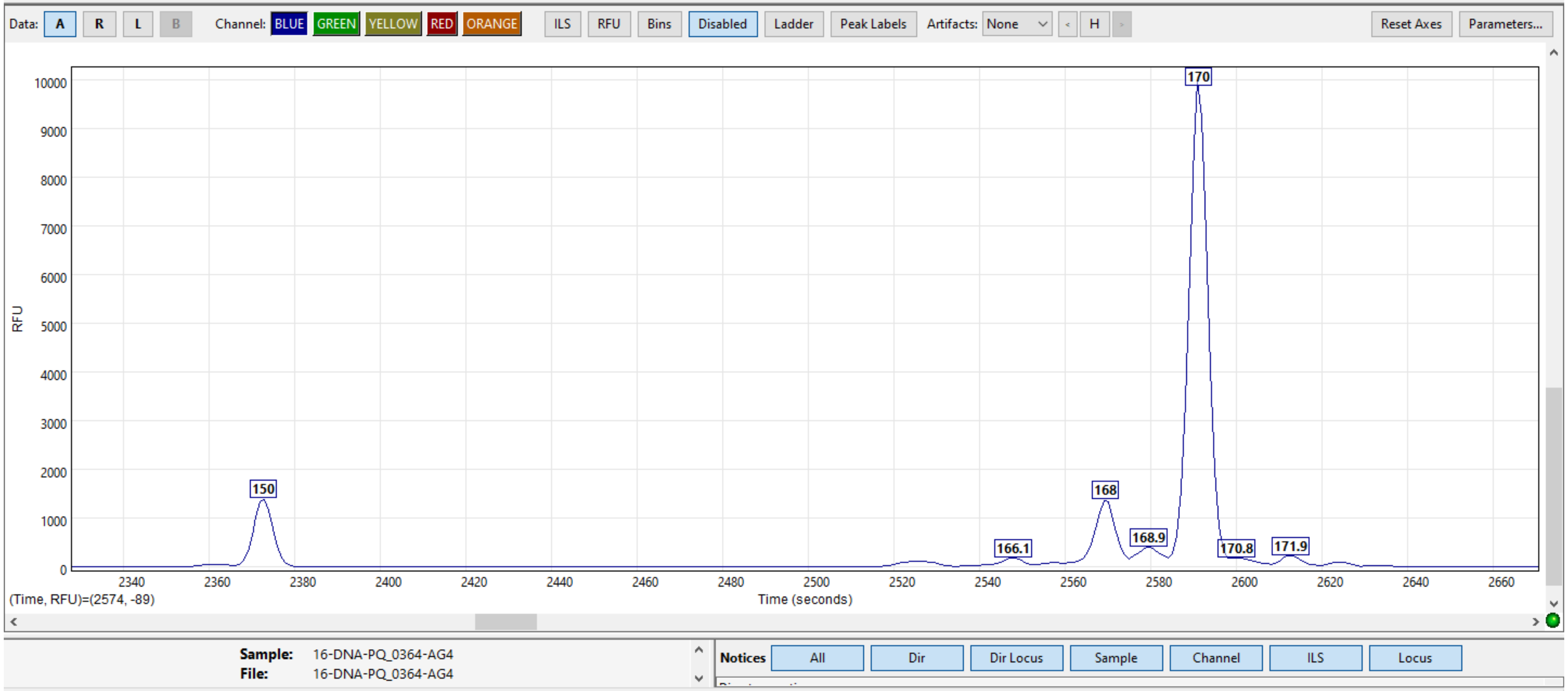


| | |
|---|---|
| Observer 1 | 150;170 |
| Observer 2 | 170 |
| Observer 3 | 170 |

## 13- Colony. Locus AG4 sample 17 (0365)

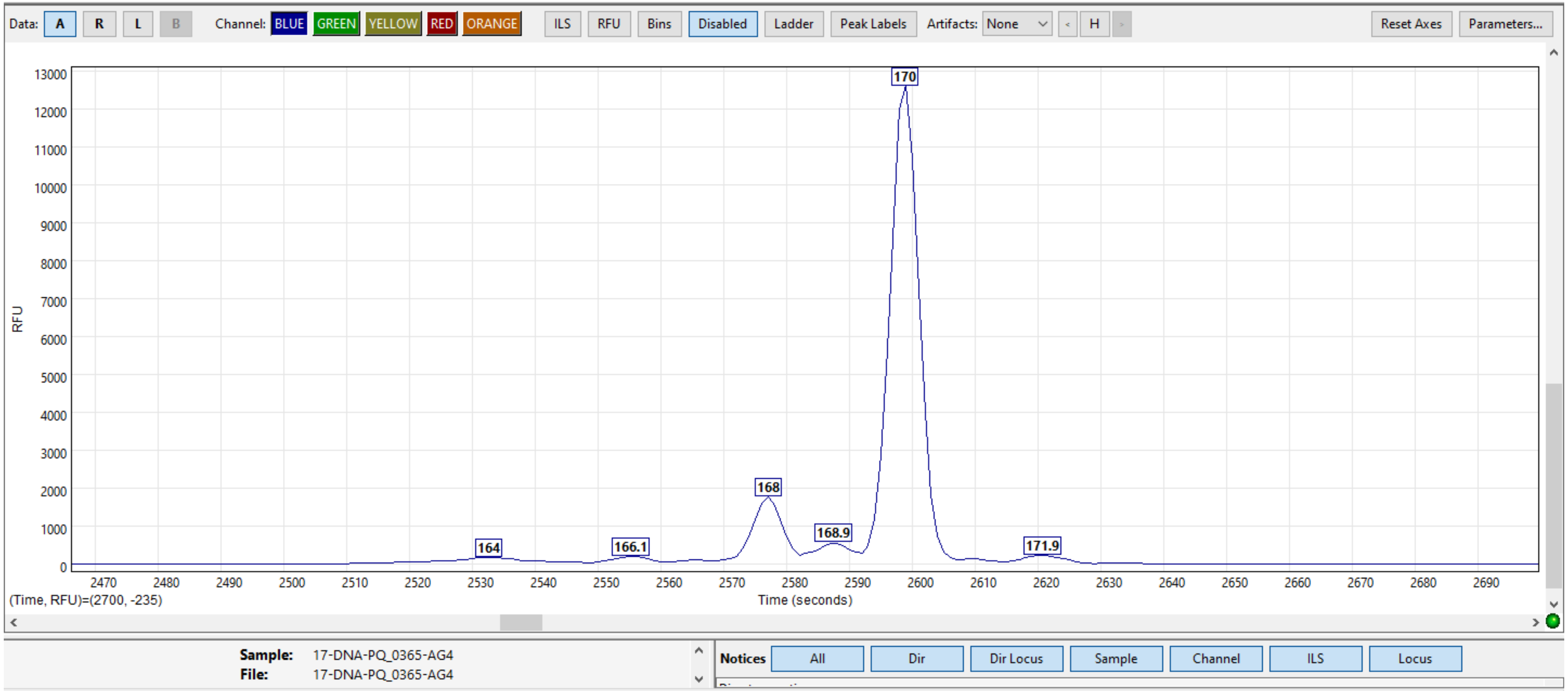


| | |
|---|---|
| **Observer 1** | **170** |
| **Observer 2** | **170** |
| **Observer 3** | **170** |

### 14- Colony. Locus AG4 sample 18 (0366)

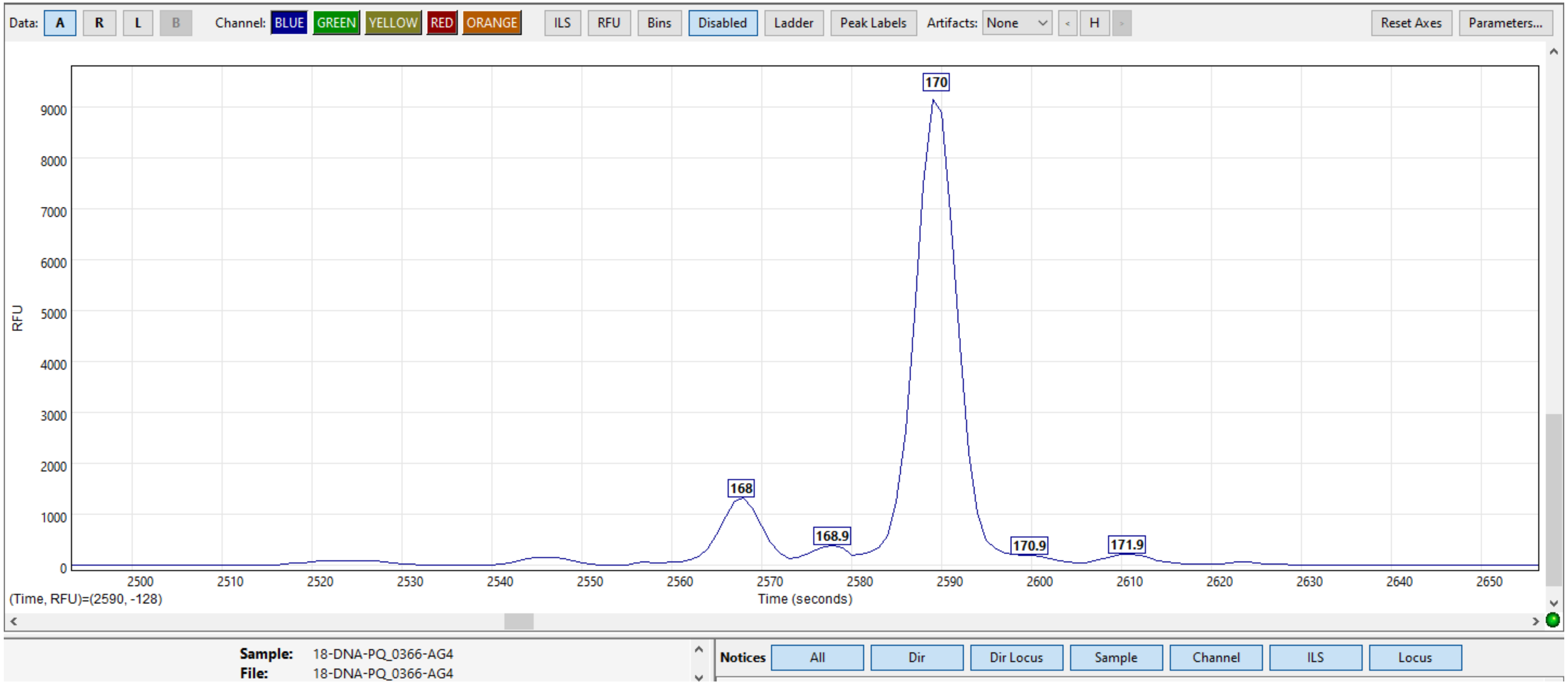


| | |
|---|---|
| **Observer 1** | **170** |
| **Observer 2** | **170** |
| **Observer 3** | **170** |

## 15- Colony. Locus AG4 sample 20 (0923)

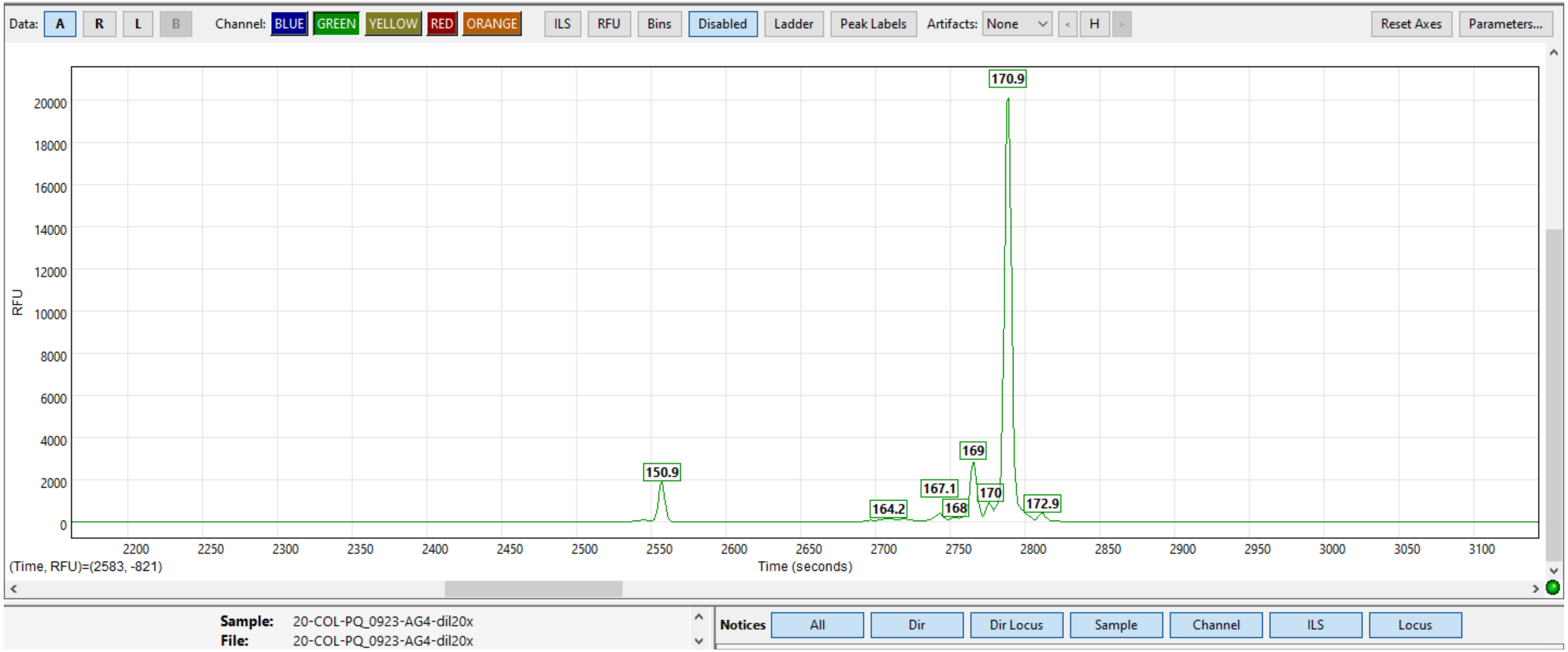


| | |
|---|---|
| **Observer 1** | **151;171** |
| **Observer 2** | **170.9** |
| **Observer 3** | **170** |

## AG7

### 1- Colony. Locus AG7 sample 01 (0353)

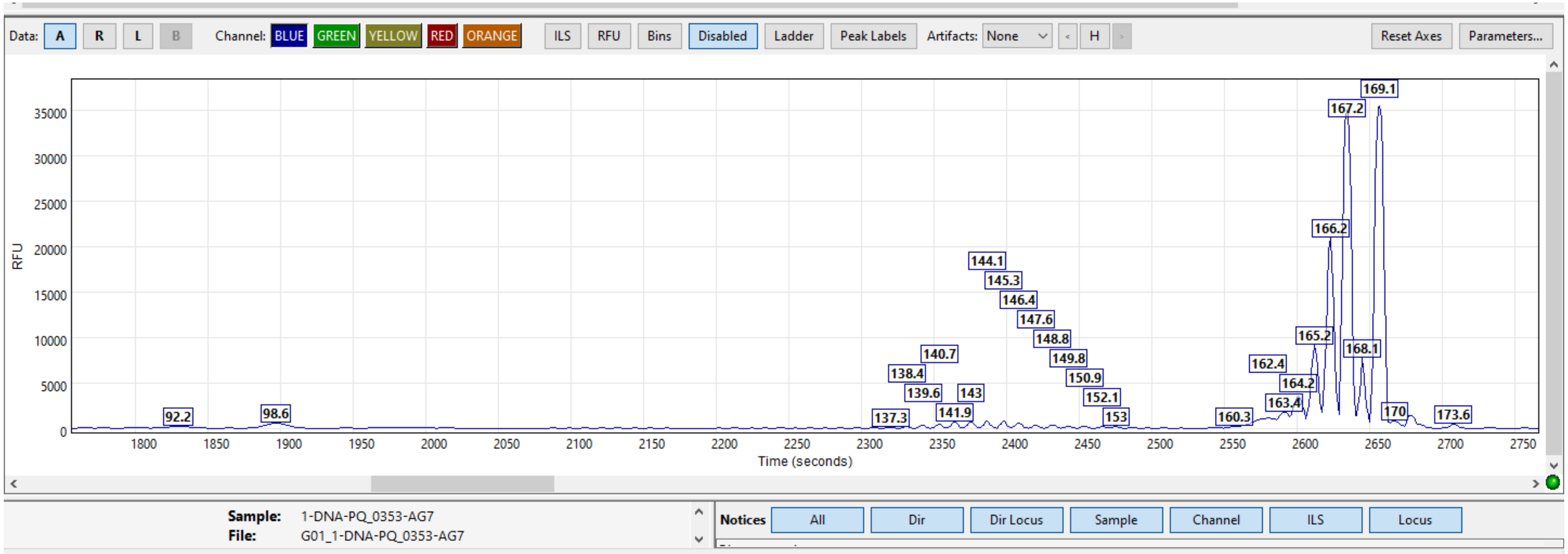


| | |
|---|---|
| **Observer 1** | **169** |
| **Observer 2** | **167.2;169.1** |
| **Observer 3** | **167;169** |

## 2- Colony. Locus AG7 sample 04 (0354)

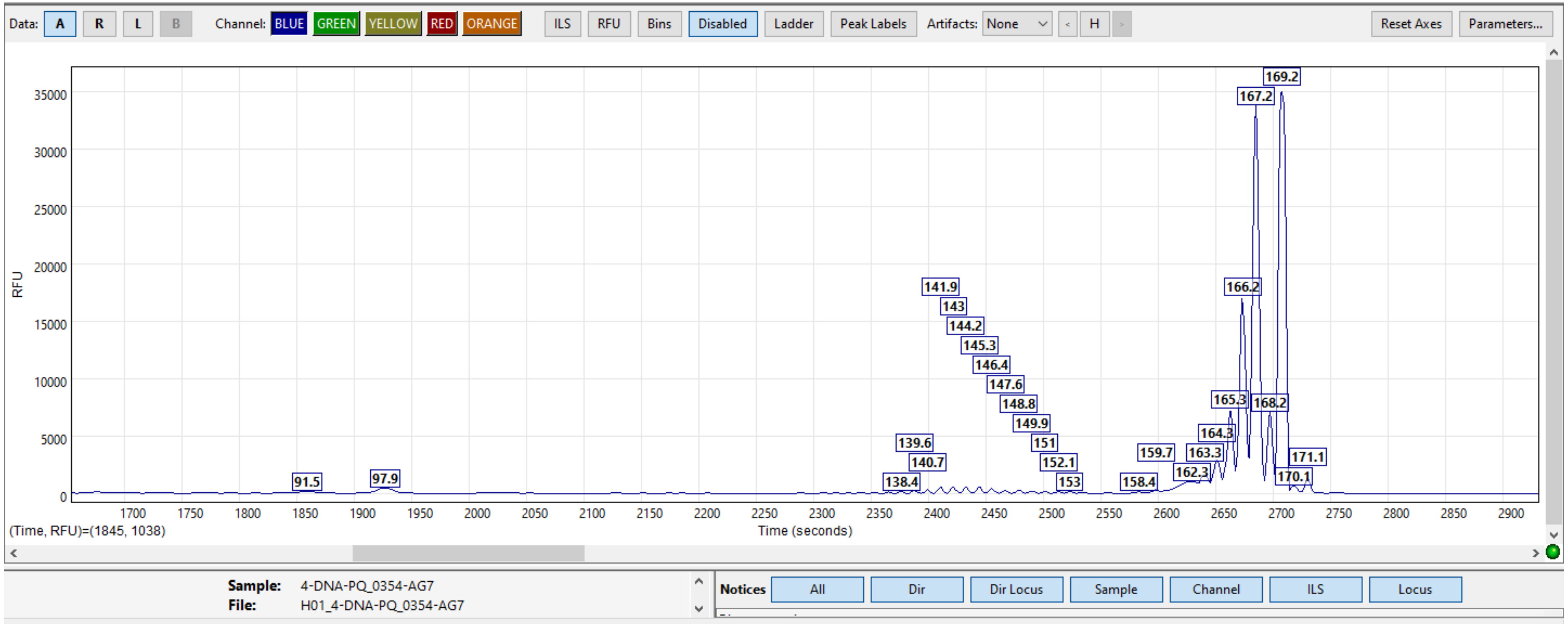


| | |
|---|---|
| **Observer 1** | **169** |
| **Observer 2** | **167.2;169.2** |
| **Observer 3** | **167;169** |

## 3- Colony. Locus AG7 sample 05 (0355)

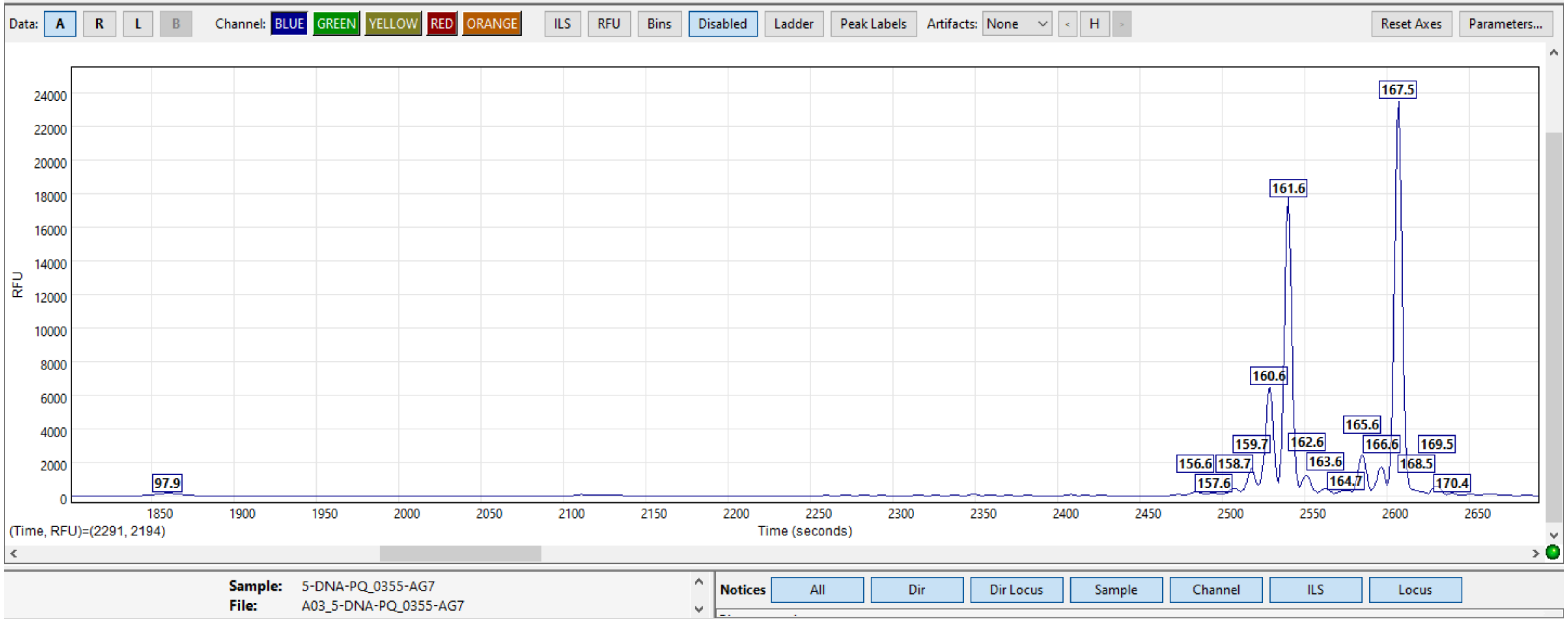


| | |
|---|---|
| **Observer 1** | **167.5;161** |
| **Observer 2** | **161.6;167.5** |
| **Observer 3** | **161;167** |

**4- Colony. Locus AG7 sample 06 (0356)**

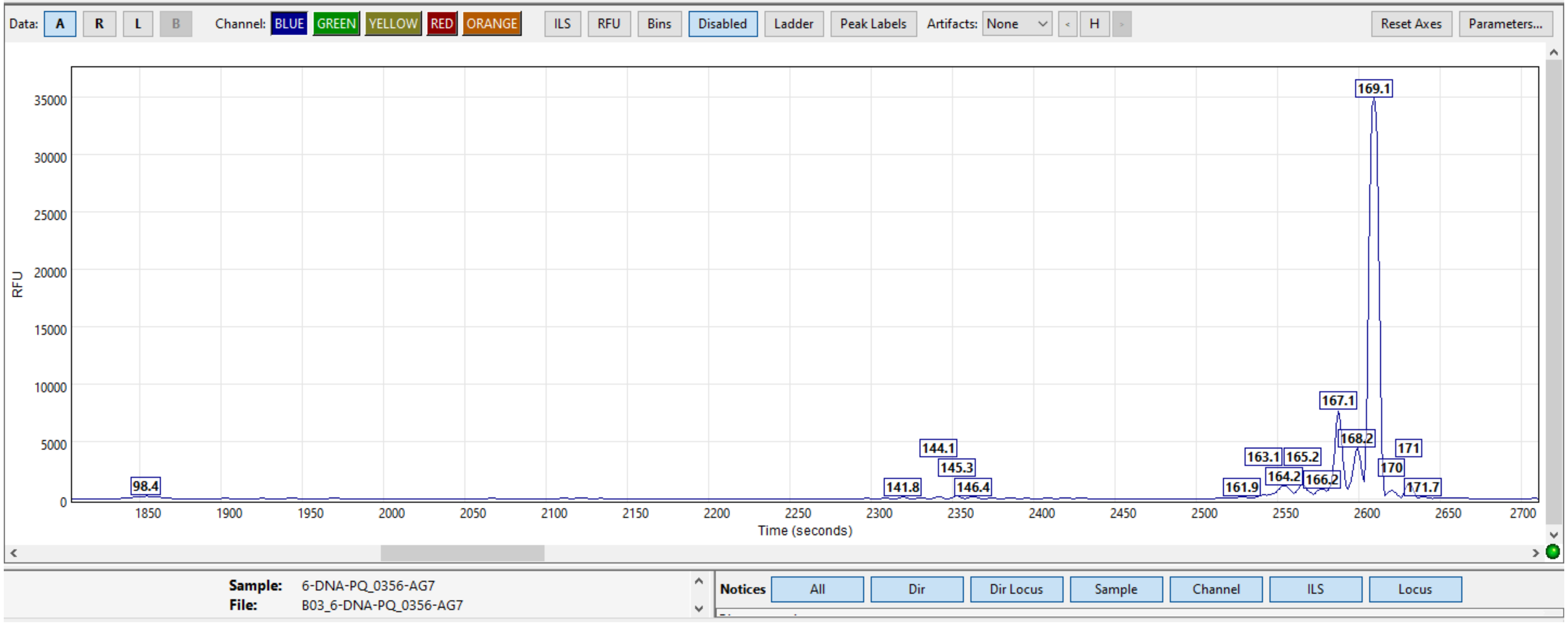


| | |
|---|---|
| **Observer 1** | **169;98** |
| **Observer 2** | **169.1** |
| **Observer 3** | **169** |

## 5- Colony. Locus AG7 sample 07 (0357)

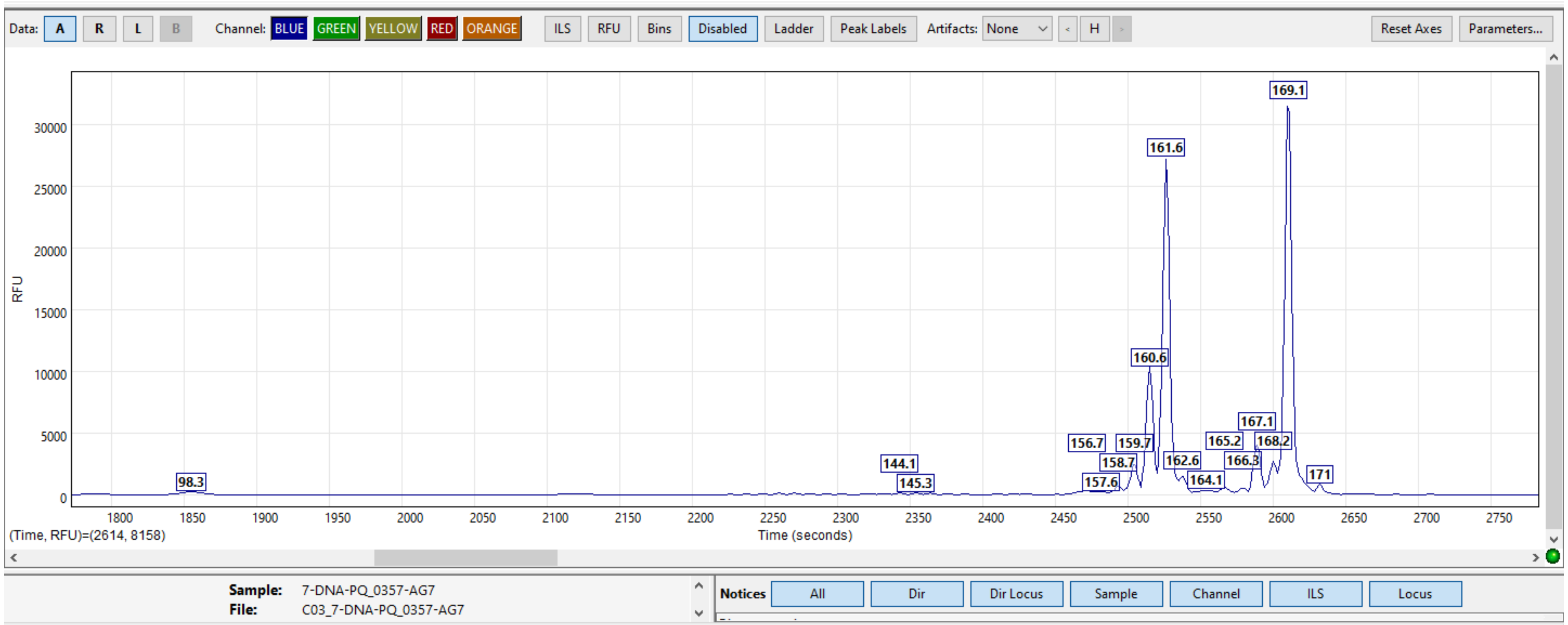


| | |
|---|---|
| **Observer 1** | **162 ; 169** |
| **Observer 2** | **161.6;169.1** |
| **Observer 3** | **161;169** |

## 6- Colony. Locus AG7 sample 08 (0358)

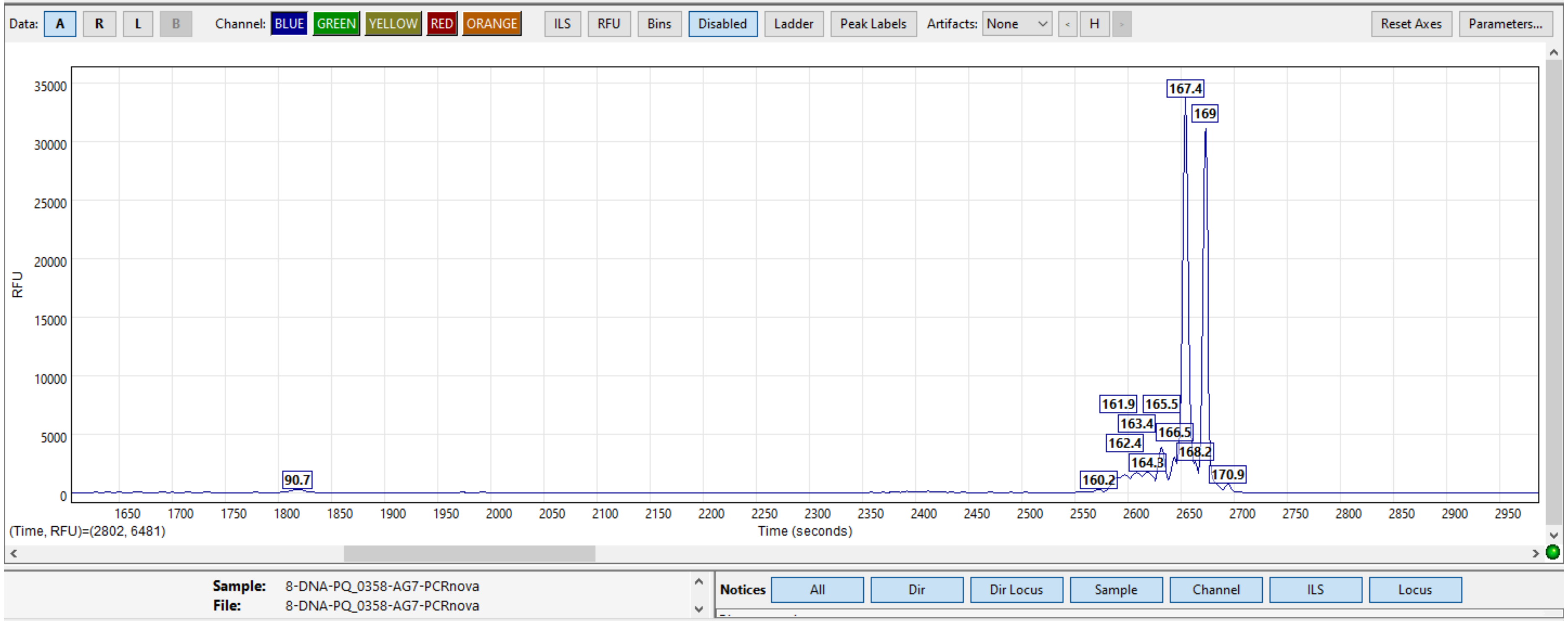


| | |
|---|---|
| **Observer 1** | **167;91** |
| **Observer 2** | **167.4;169** |
| **Observer 3** | **167;169** |

**7- Colony. Locus AG7 sample 10 (0359)**

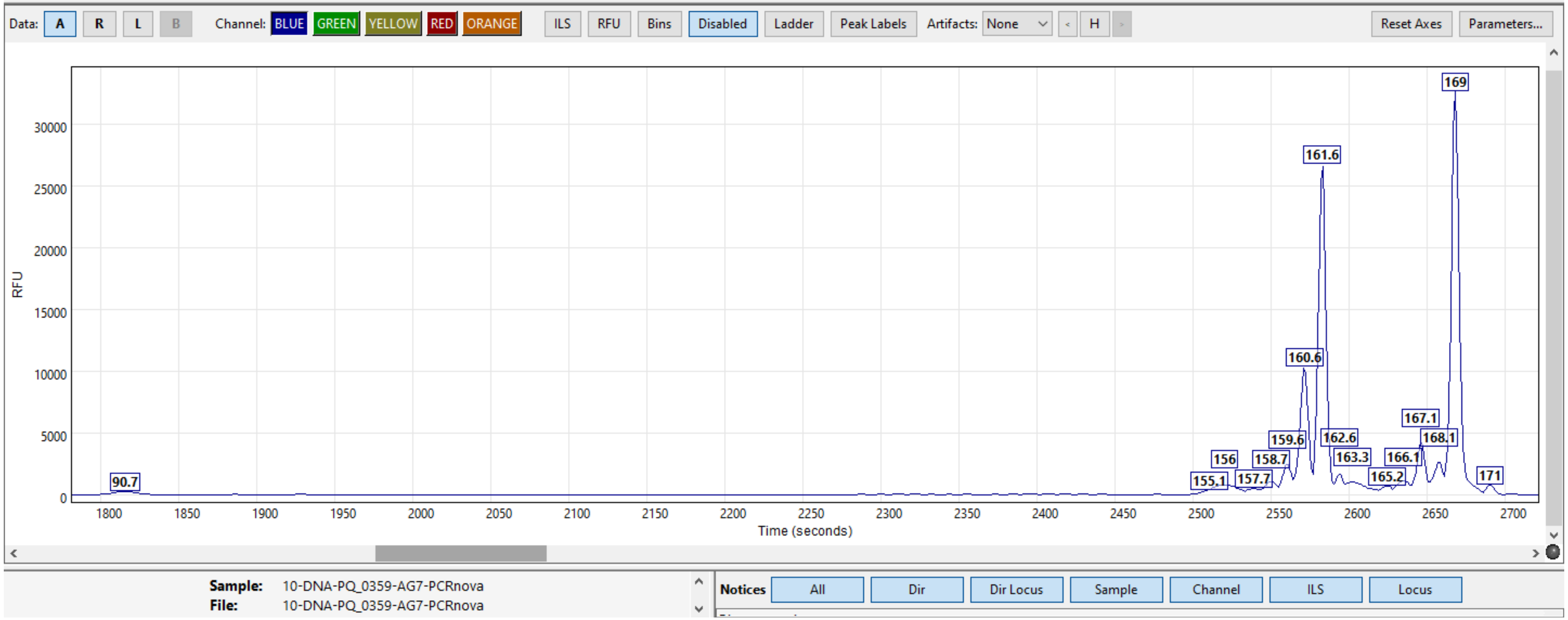


| | |
|---|---|
| **Observer 1** | **162 ; 169** |
| **Observer 2** | **161.6 ; 169** |
| **Observer 3** | **161 ; 169** |

**8- Colony. Locus AG7 sample 11 (0360)**

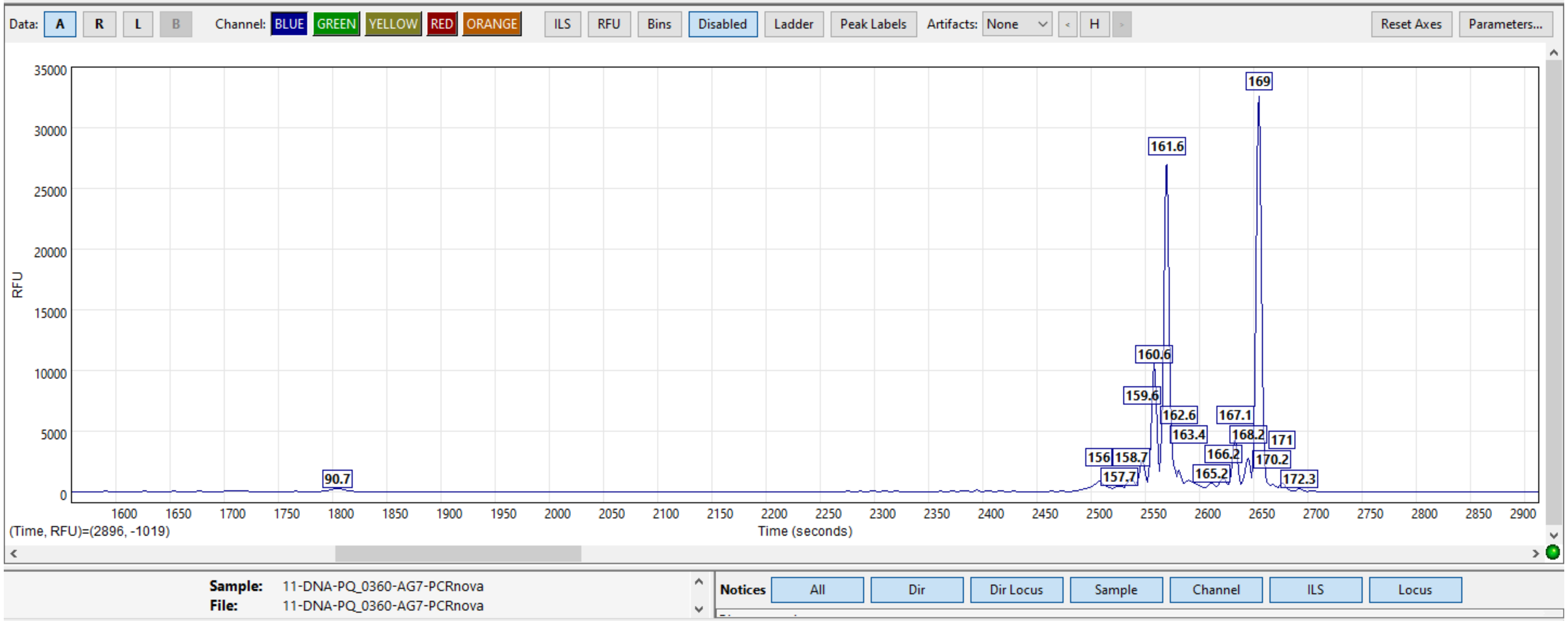


| | |
|---|---|
| **Observer 1** | **162 ; 169** |
| **Observer 2** | **161.6 ; 169** |
| **Observer 3** | **161 ; 169** |

## 9- Colony. Locus AG7 sample 12 (0361)

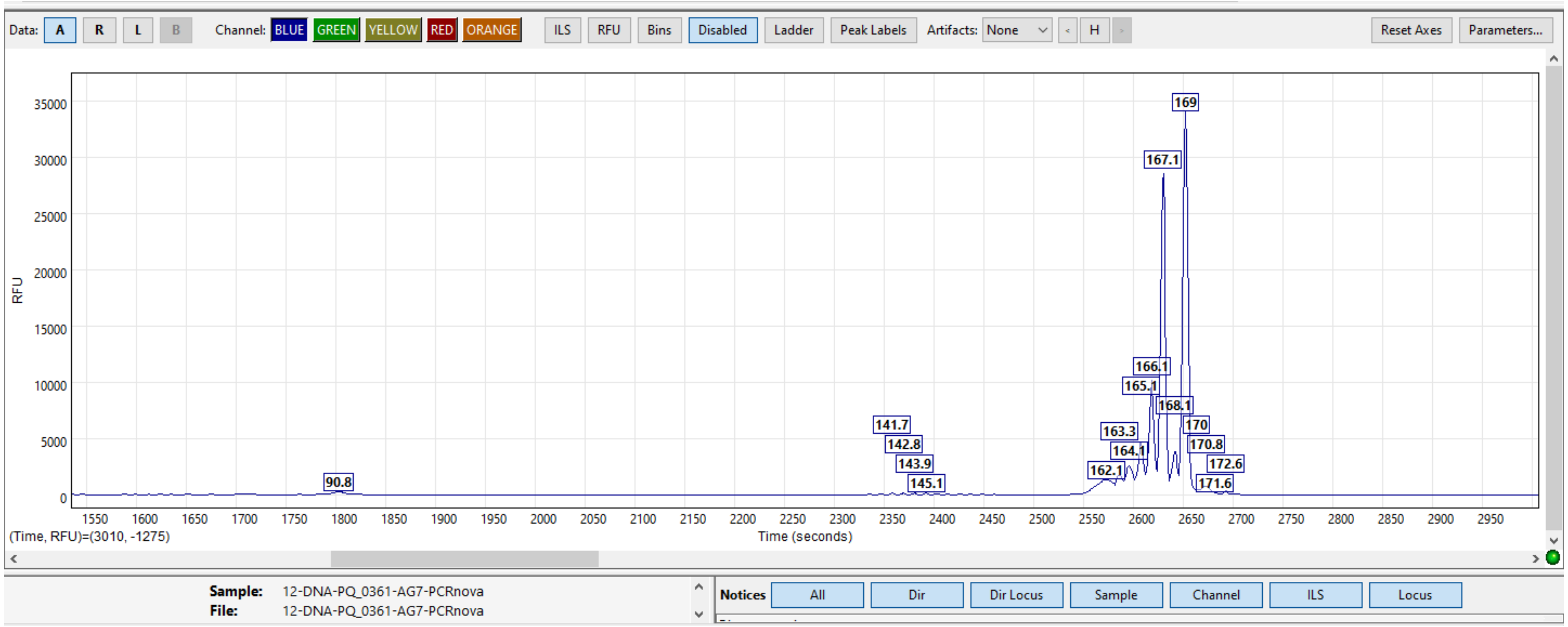


| | |
|---|---|
| **Observer 1** | **169;91** |
| **Observer 2** | **167.1;169** |
| **Observer 3** | **167;169** |

**10- Colony. Locus AG7 sample 13 (0362)**

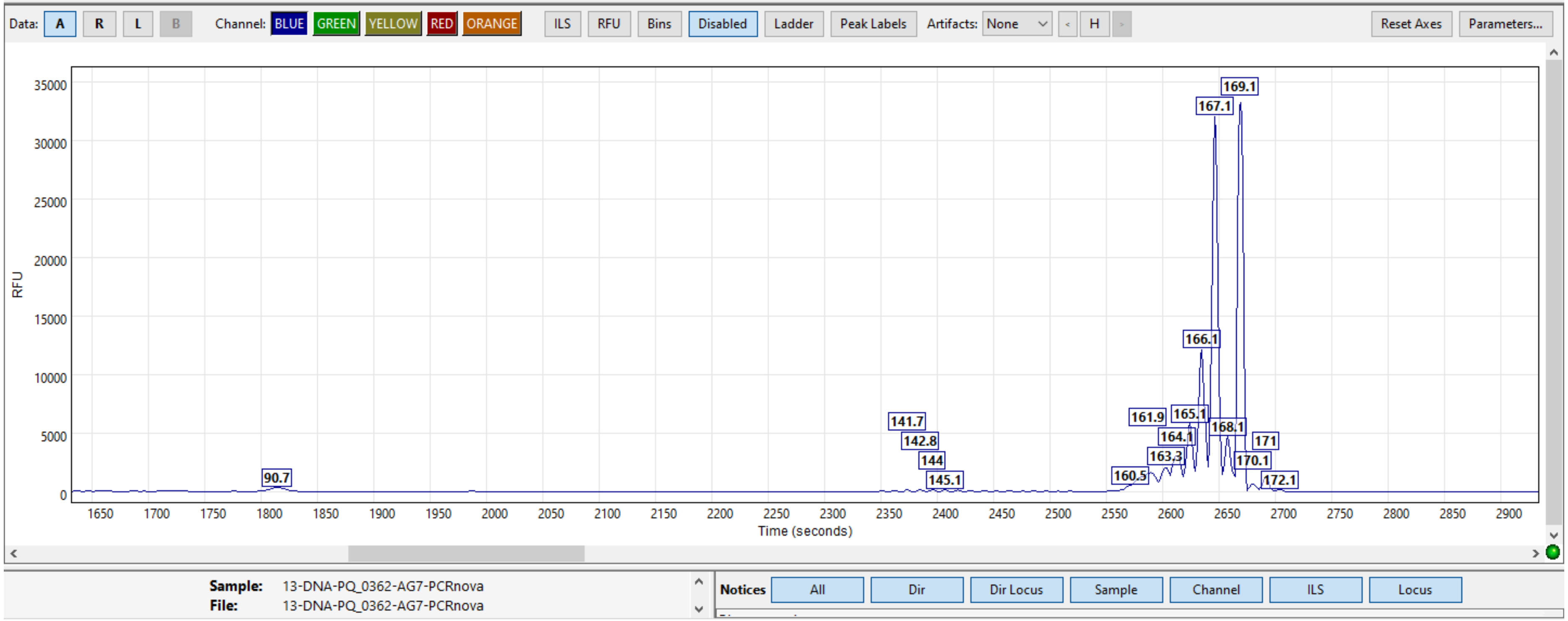


| | |
|---|---|
| **Observer 1** | **169;91** |
| **Observer 2** | **167.1;169.1** |
| **Observer 3** | **167;169** |

## 11- Colony. Locus AG7 sample 14 (0363)

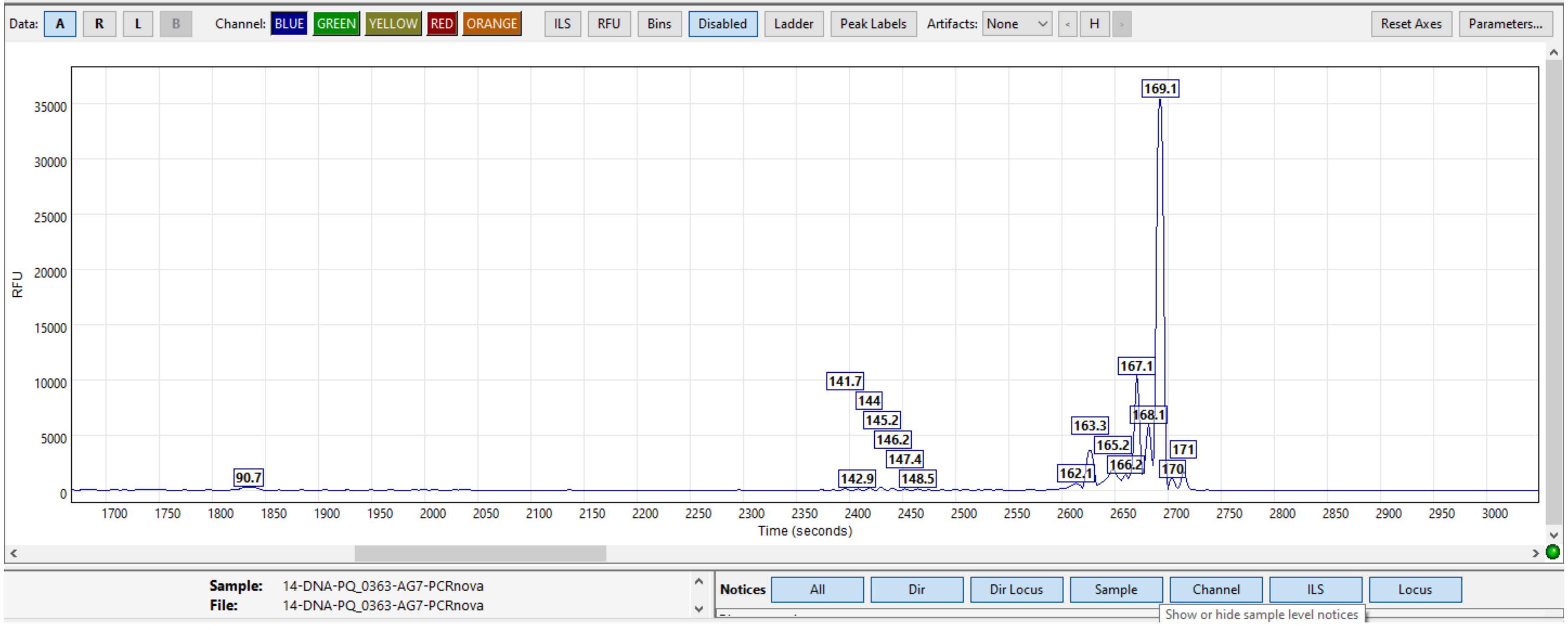


| | |
|---|---|
| **Observer 1** | **169;91** |
| **Observer 2** | **169.1** |
| **Observer 3** | **169** |

**12- Colony. Locus AG7 sample 16 (0364)**

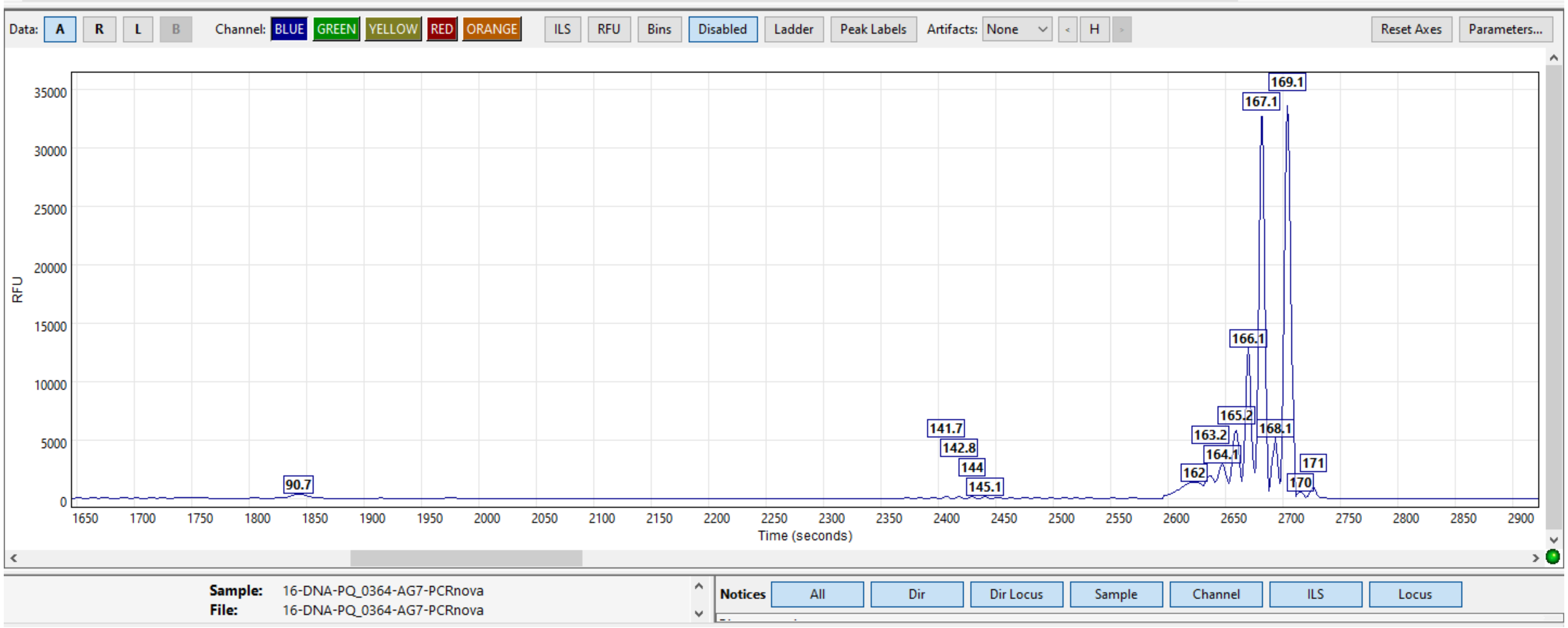


| | |
|---|---|
| **Observer 1** | 169;91 |
| **Observer 2** | 167.1;169.1 |
| **Observer 3** | 167;169 |

**13- Colony. Locus AG7 sample 17 (0365)**

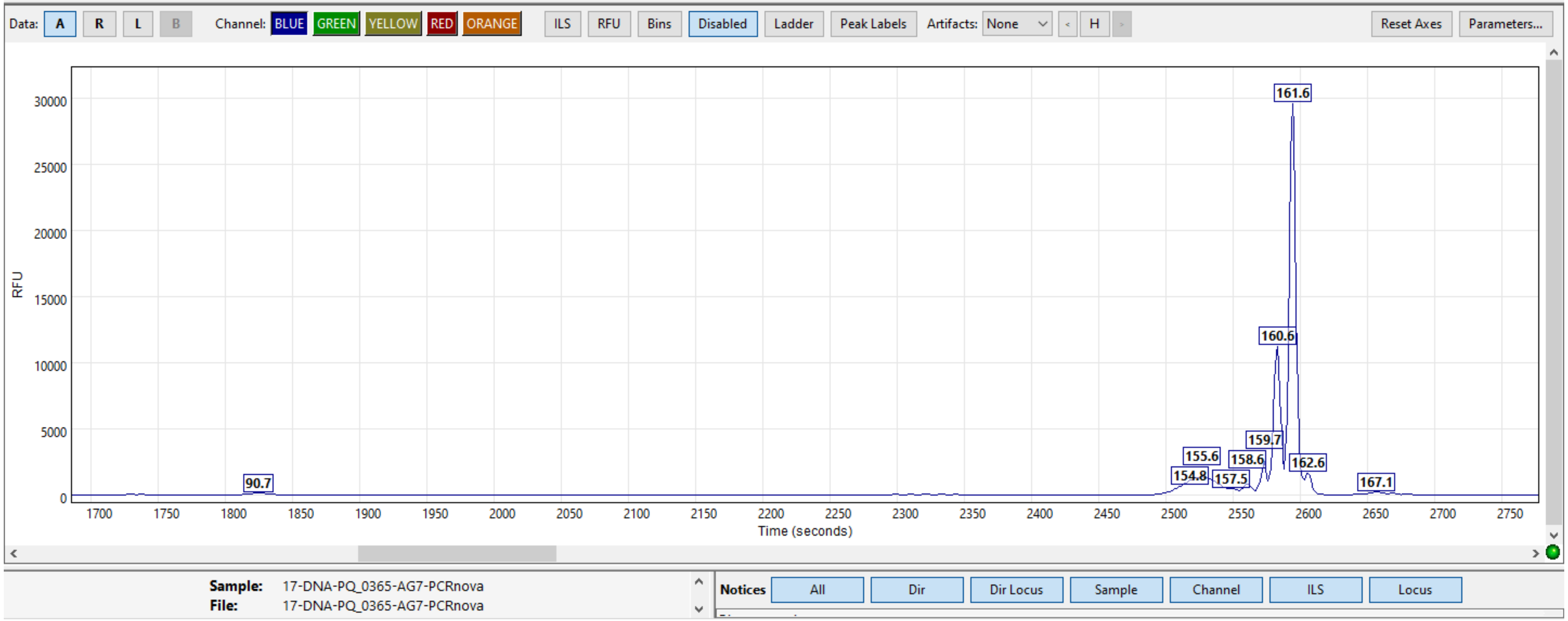


| | |
|---|---|
| **Observer 1** | **162;91** |
| **Observer 2** | **161.6** |
| **Observer 3** | **161** |

**14- Colony. Locus AG7 sample 18 (0366)**

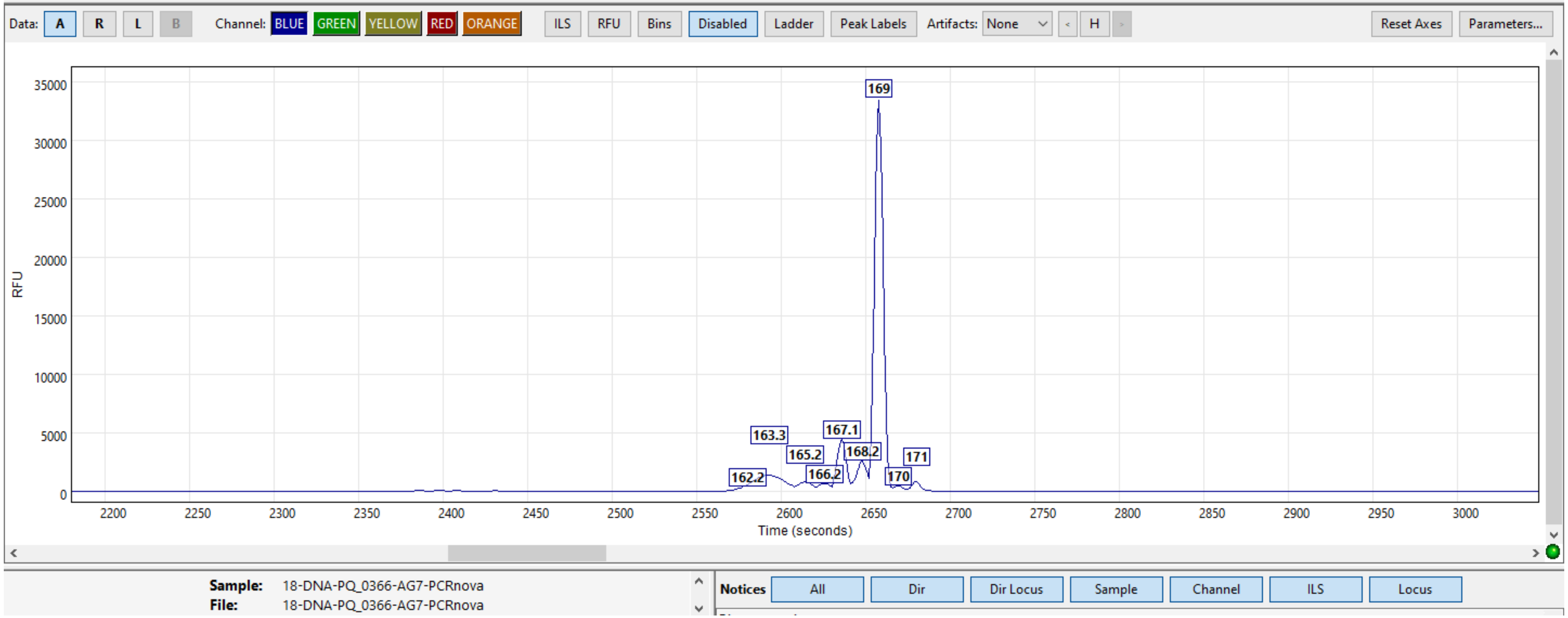


| | |
|---|---|
| **Observer 1** | **169** |
| **Observer 2** | **169** |
| **Observer 3** | **169** |

### 15- Colony. Locus AG7 sample 20 (0923)

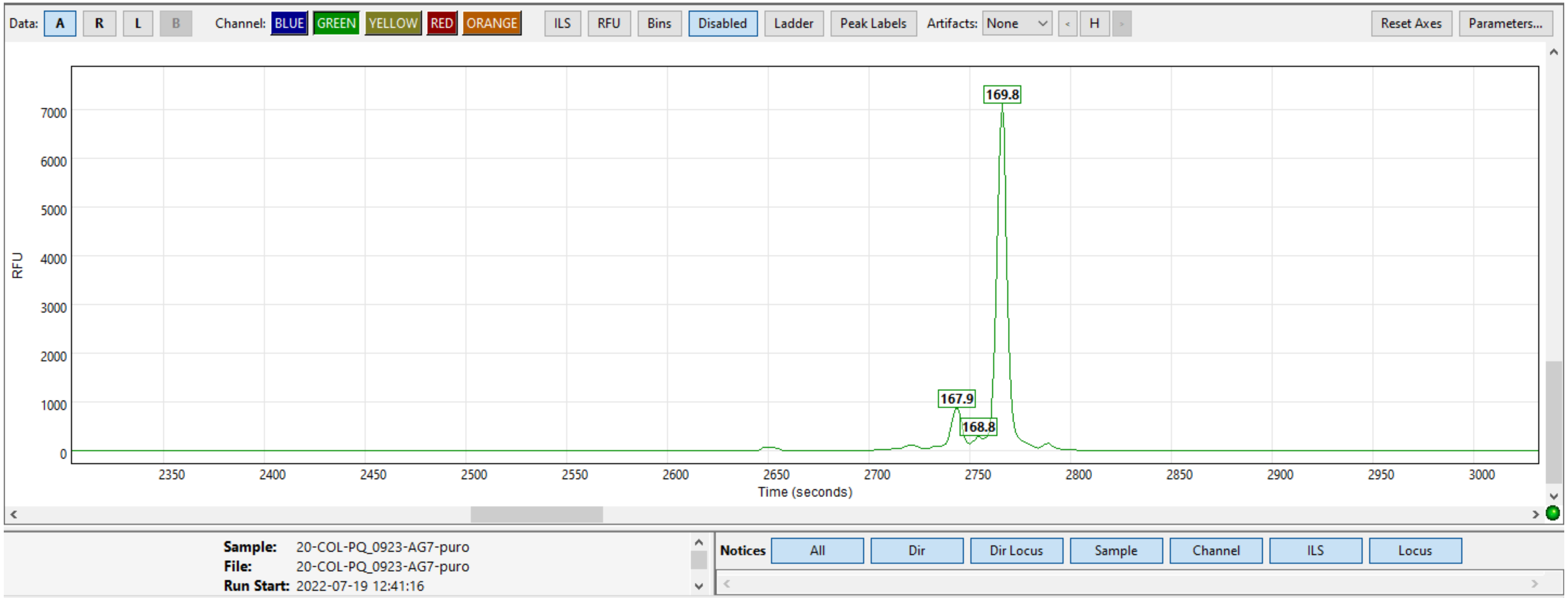


| | |
|---|---|
| **Observer 1** | **170** |
| **Observer 2** | **169.8** |
| **Observer 3** | **169** |

**AT1**

**1- Colony. Locus AT1 sample 01 (0353)**

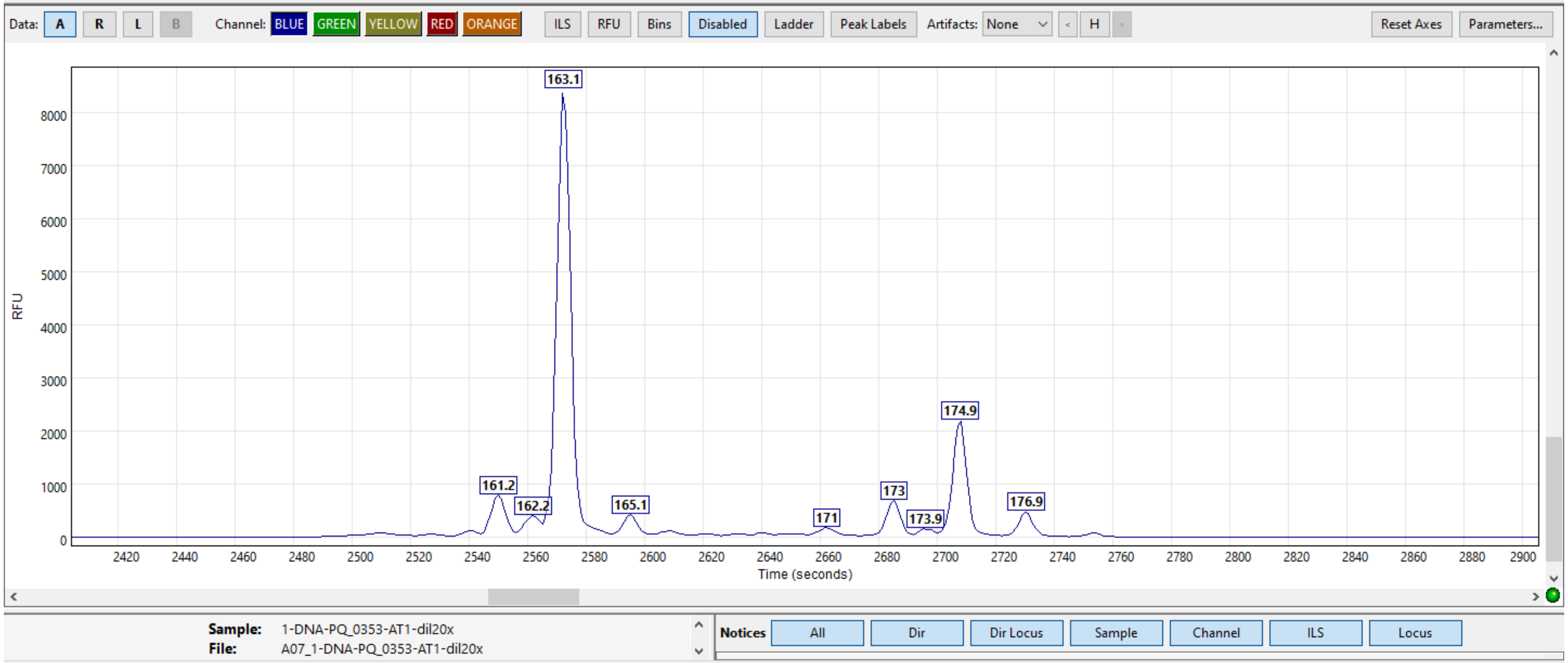


| | |
|---|---|
| **Observer 1** | **163;175** |
| **Observer 2** | **163.1;174.9** |
| **Observer 3** | **163;175** |

## 2- Colony. Locus AT1 sample 04 (0354)

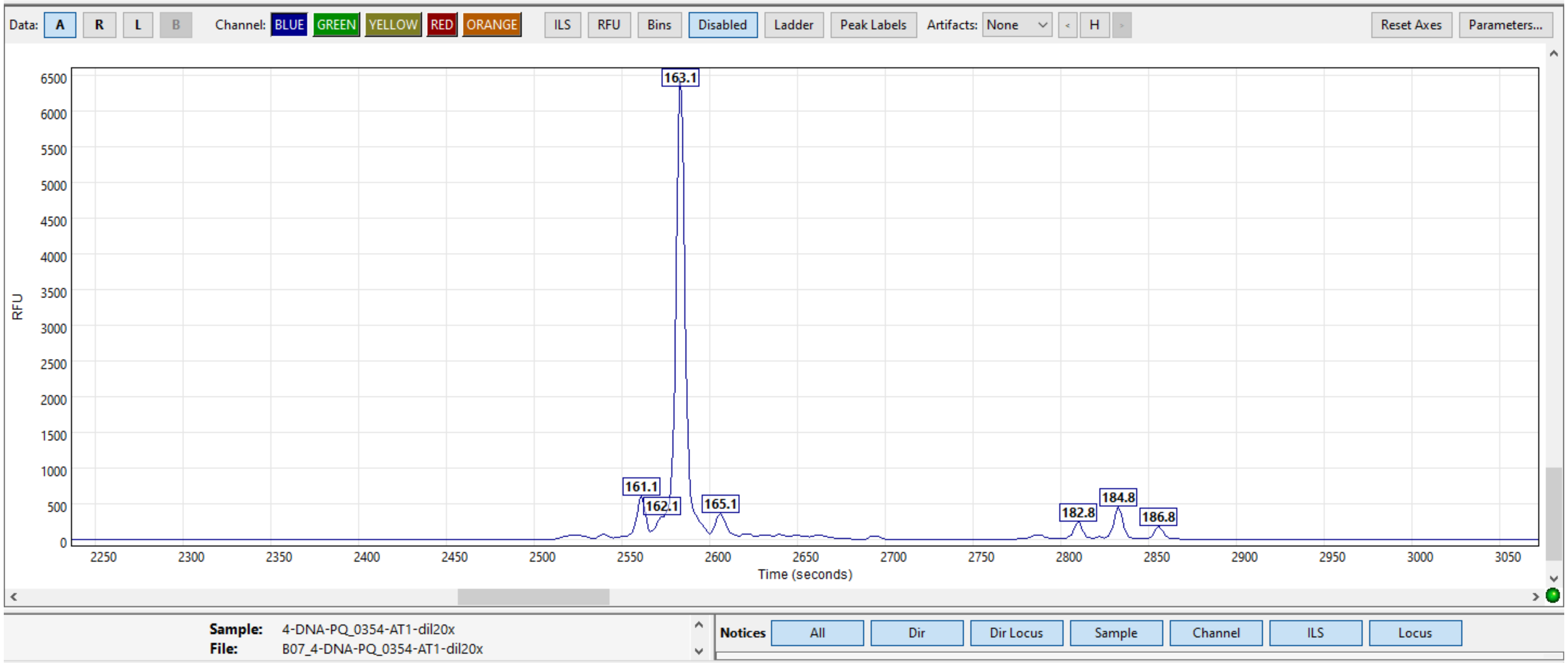


| | |
|---|---|
| **Observer 1** | **163** |
| **Observer 2** | **163.1** |
| **Observer 3** | **163** |

### 3- Colony. Locus AT1 sample 05 (0355)

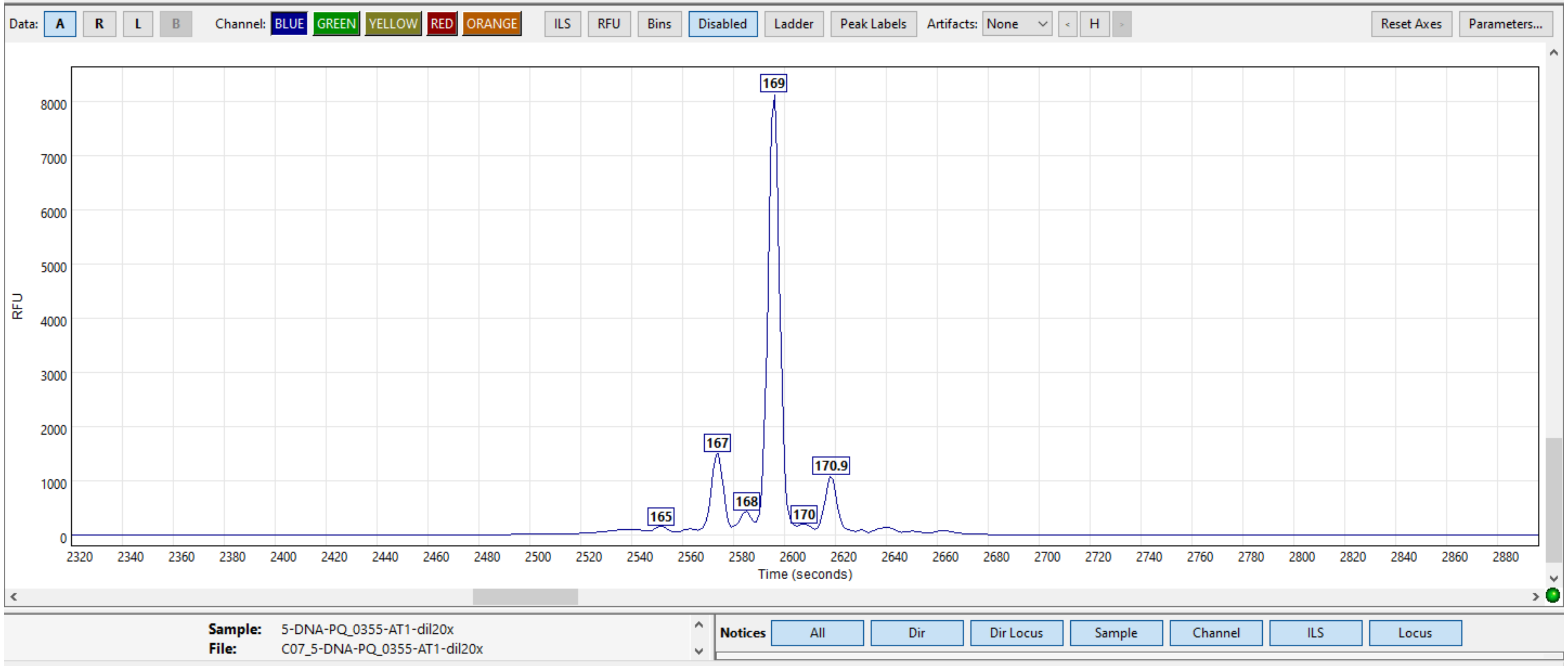


| | |
|---|---|
| **Observer 1** | **169** |
| **Observer 2** | **169** |
| **Observer 3** | **169** |

4- Colony. Locus AT1 sample 06 (0356)

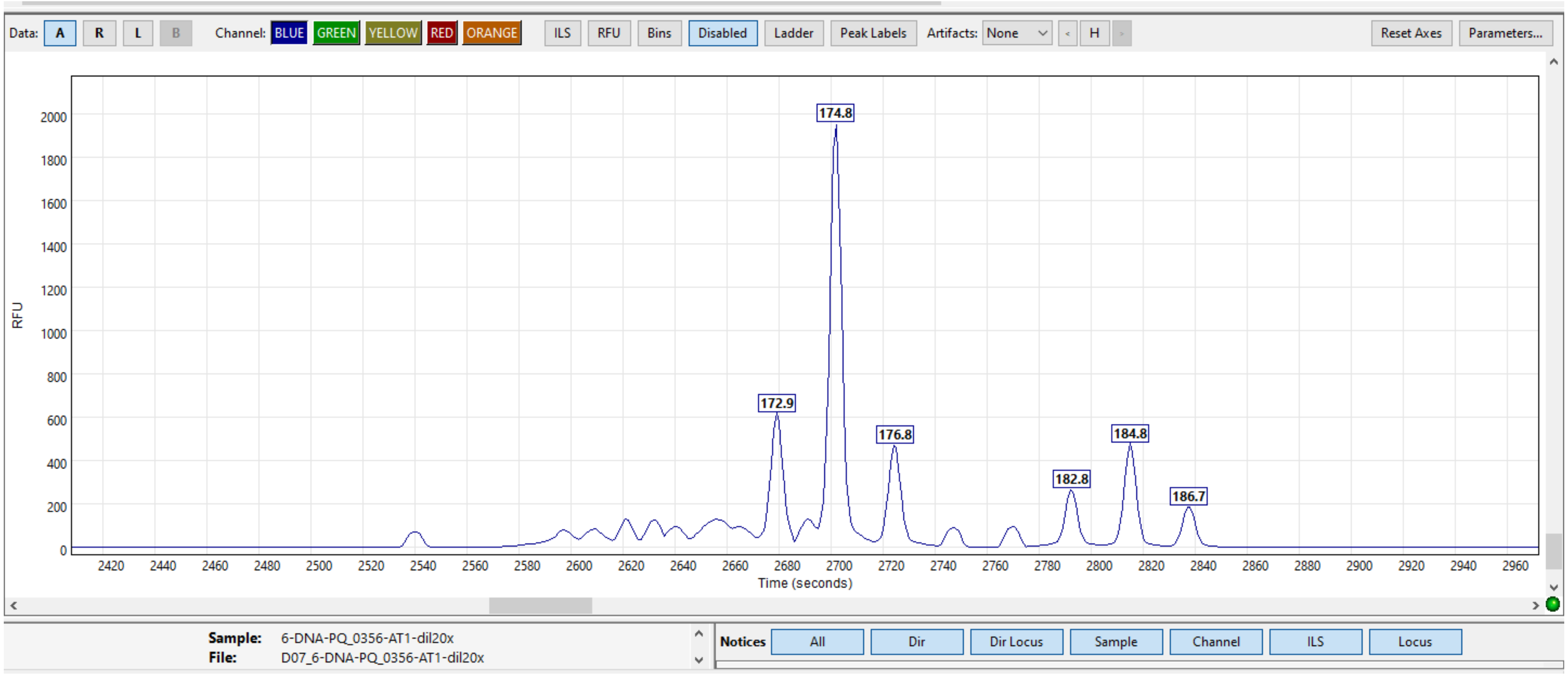


| | |
|---|---|
| **Observer 1** | **175** |
| **Observer 2** | **174.8** |
| **Observer 3** | **175** |

**5- Colony. Locus AT1 sample 07 (0357)**

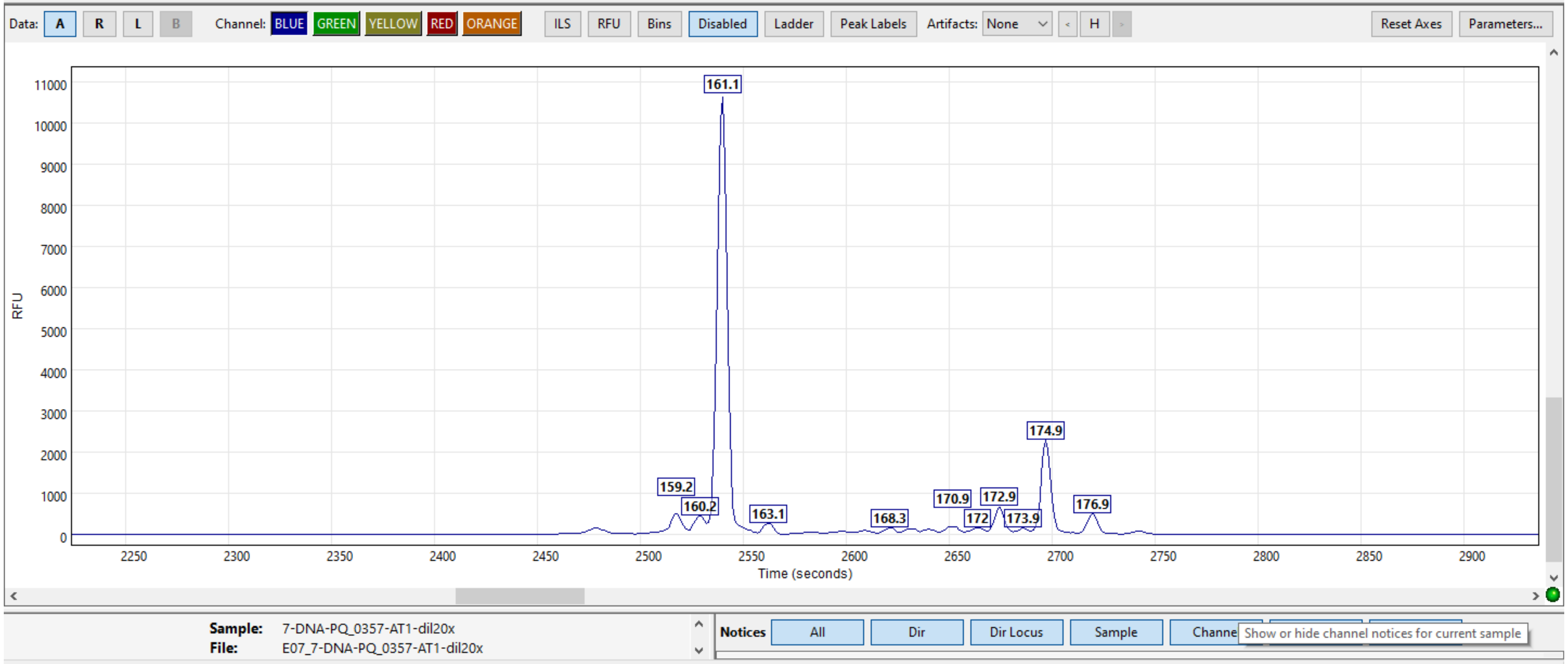


| | |
|---|---|
| **Observer 1** | **161;175** |
| **Observer 2** | **161.1;174.9** |
| **Observer 3** | **161;175** |

**6- Colony. Locus AT1 sample 08 (0358)**

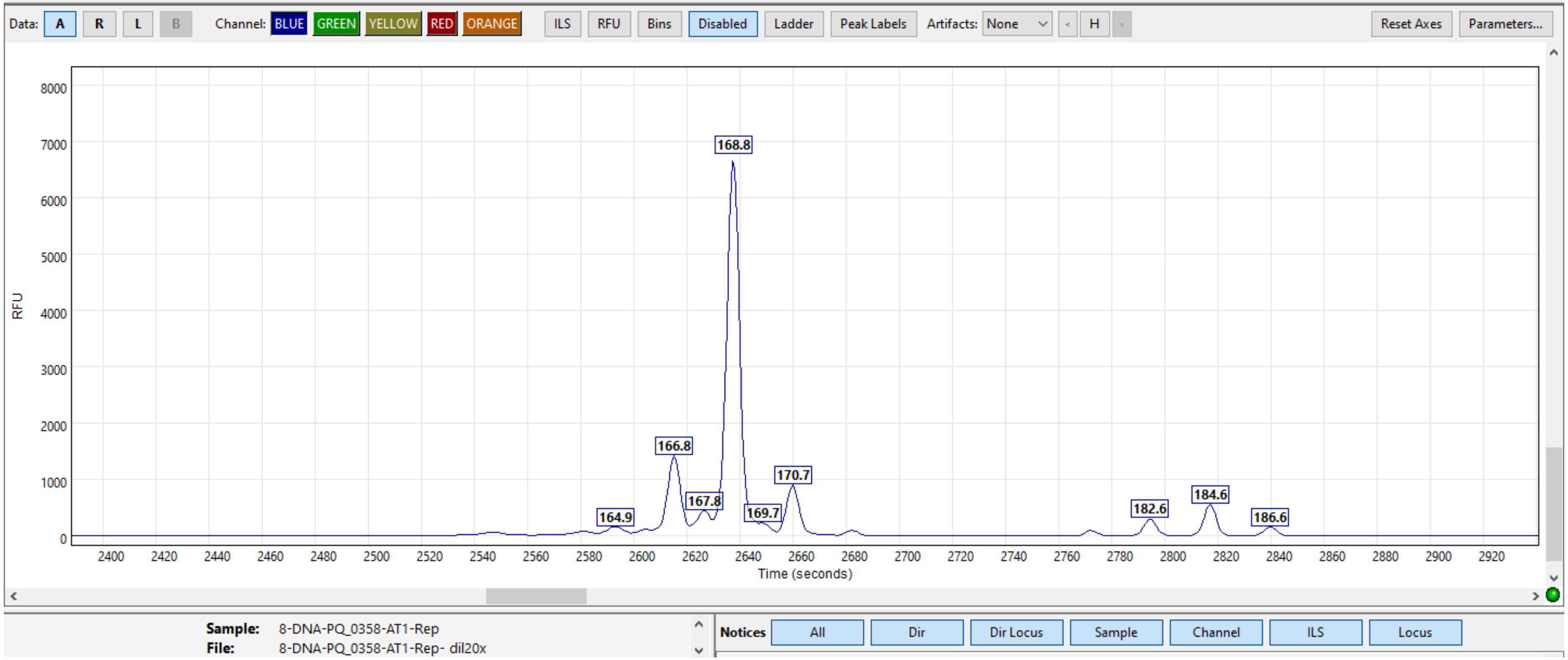


| | |
|---|---|
| **Observer 1** | **169** |
| **Observer 2** | **168.8** |
| **Observer 3** | **169** |

**7- Colony. Locus AT1 sample 10 (0359)**

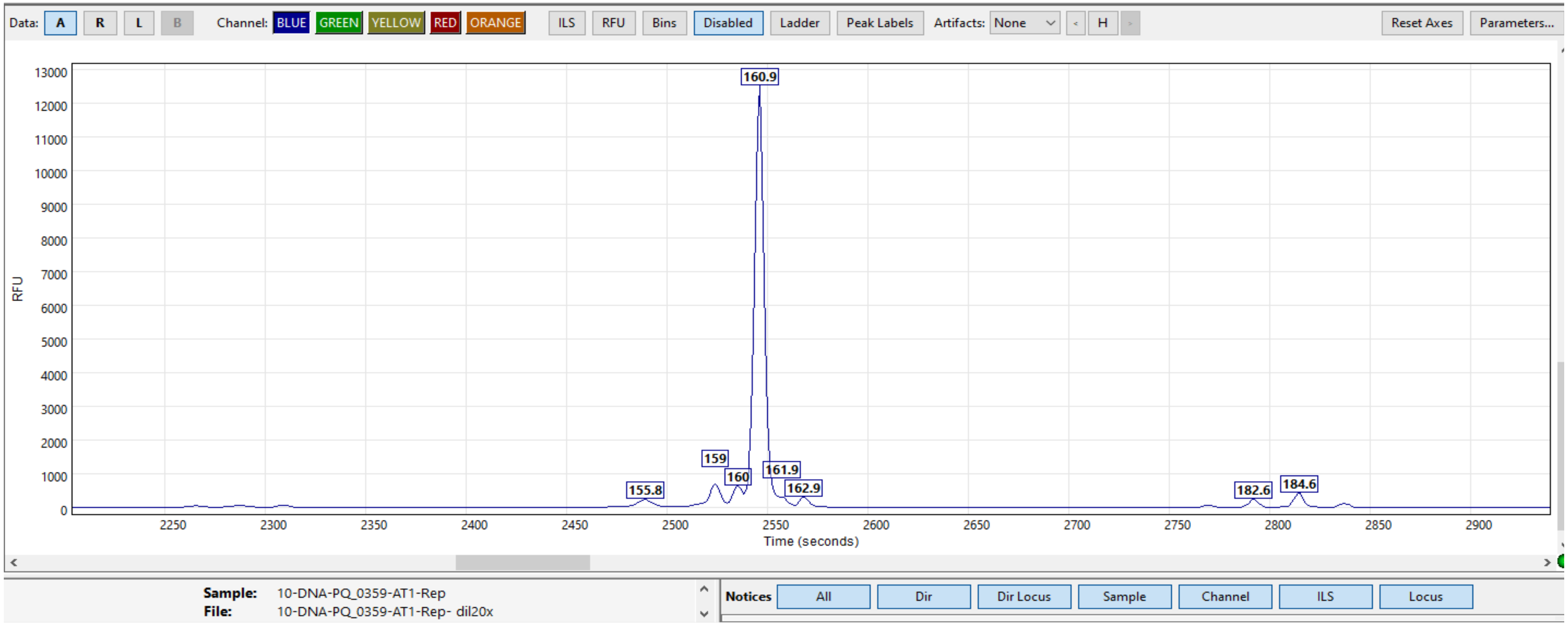


| | |
|---|---|
| **Observer 1** | **161** |
| **Observer 2** | **160.9** |
| **Observer 3** | **161** |

## 8- Colony. Locus AT1 sample 11 (0360)

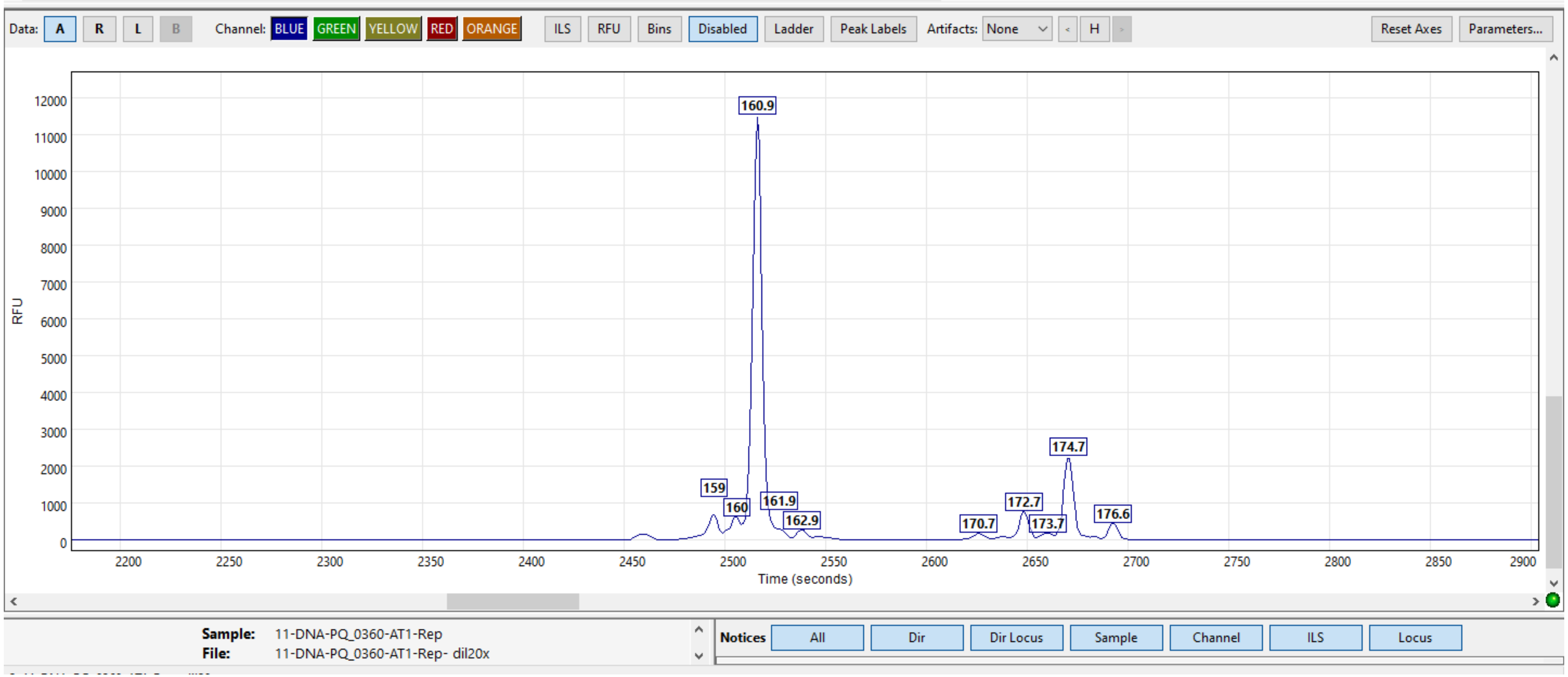


| | |
|---|---|
| **Observer 1** | **161;175** |
| **Observer 2** | **160.9;174.7** |
| **Observer 3** | **161;175** |

### 9- Colony. Locus AT1 sample 12 (0361)

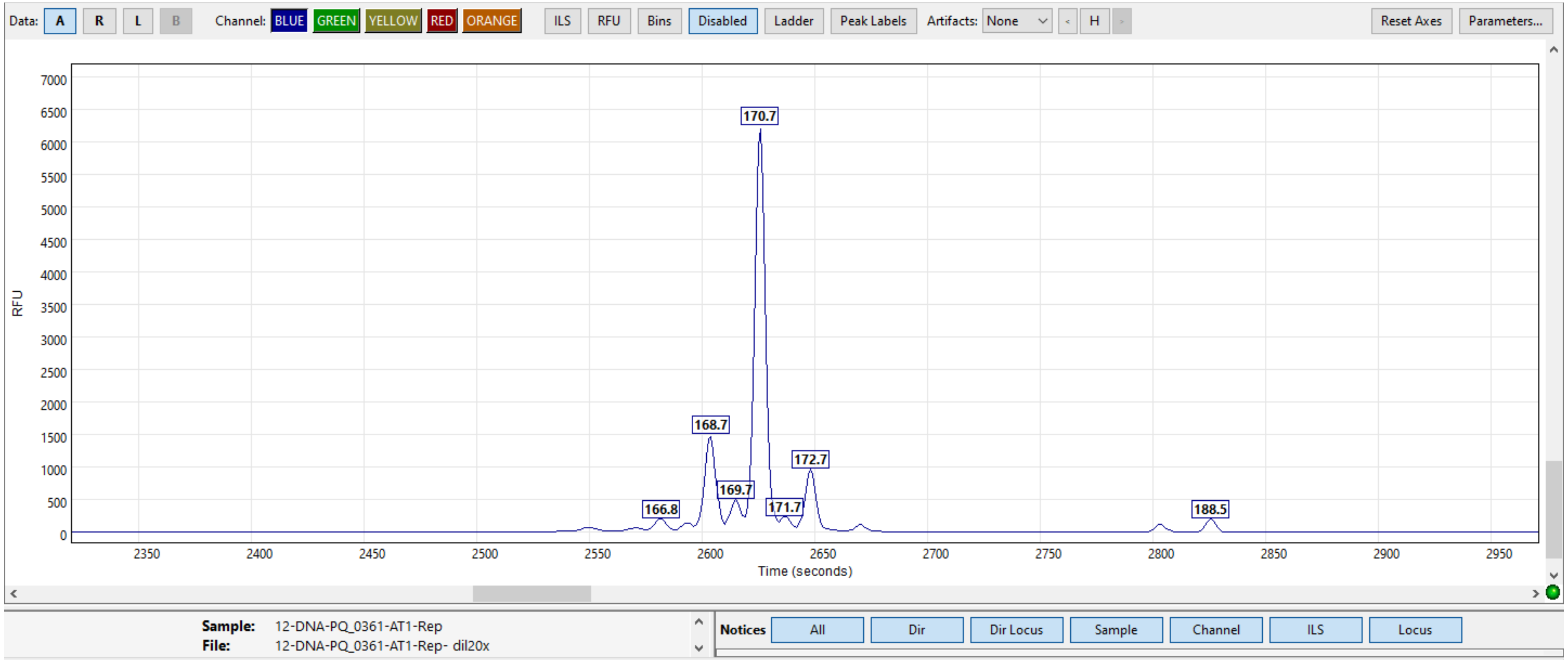


| | |
|---|---|
| **Observer 1** | **171** |
| **Observer 2** | **170.7** |
| **Observer 3** | **171** |

**10- Colony. Locus AT1 sample 13 (0362)**

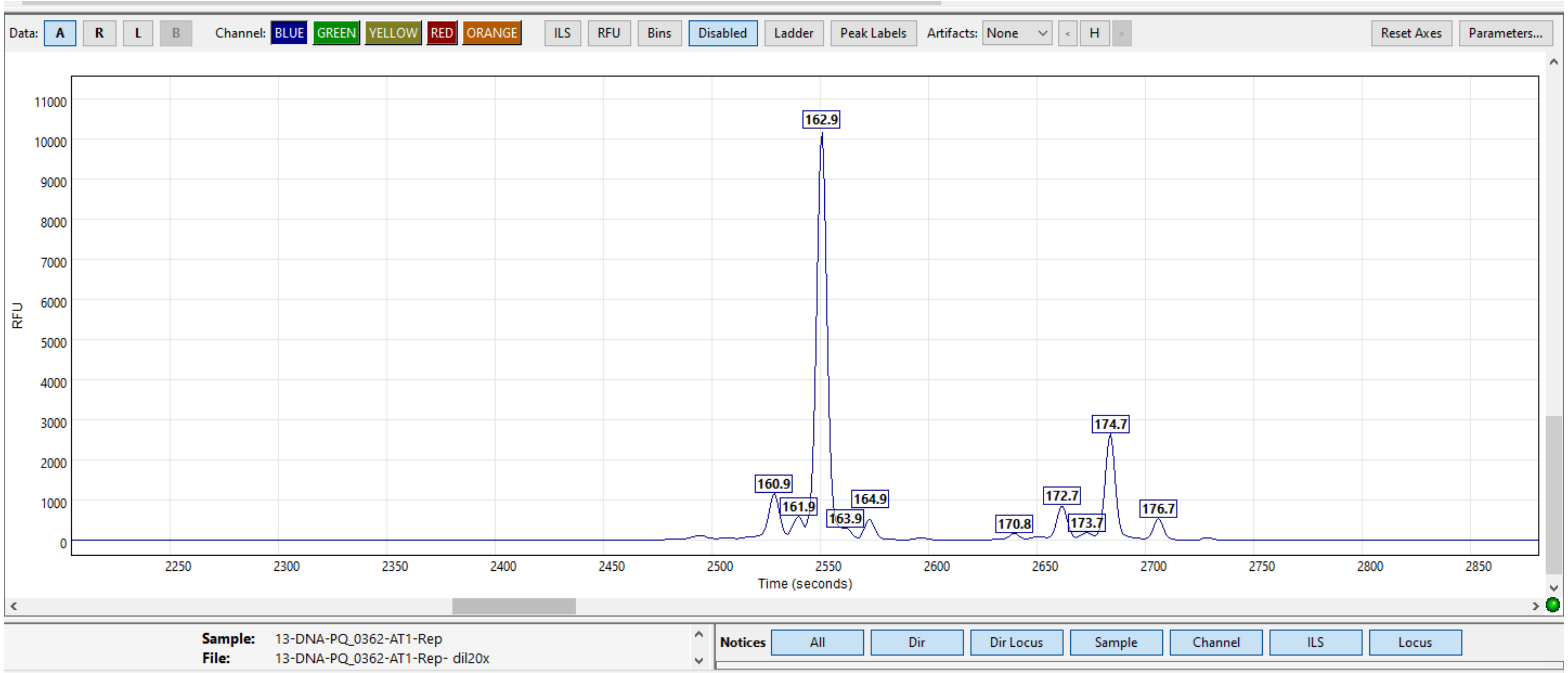


| | |
|---|---|
| **Observer 1** | **163;175** |
| **Observer 2** | **162.9;174.7** |
| **Observer 3** | **163;175** |

## 11- Colony. Locus AT1 sample 14 (0363)

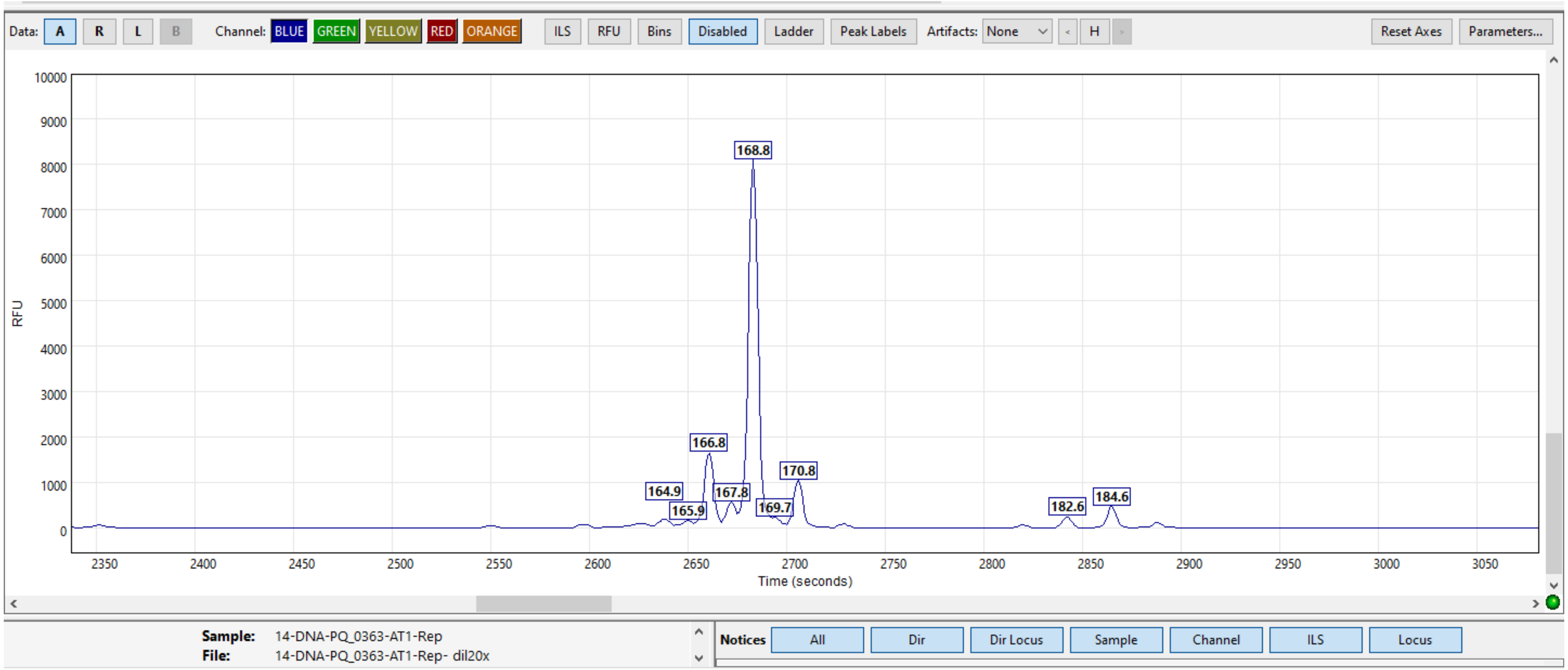


| | |
|---|---|
| **Observer 1** | **169** |
| **Observer 2** | **168.8** |
| **Observer 3** | **169** |

## 12- Colony. Locus AT1 sample 16 (0364)

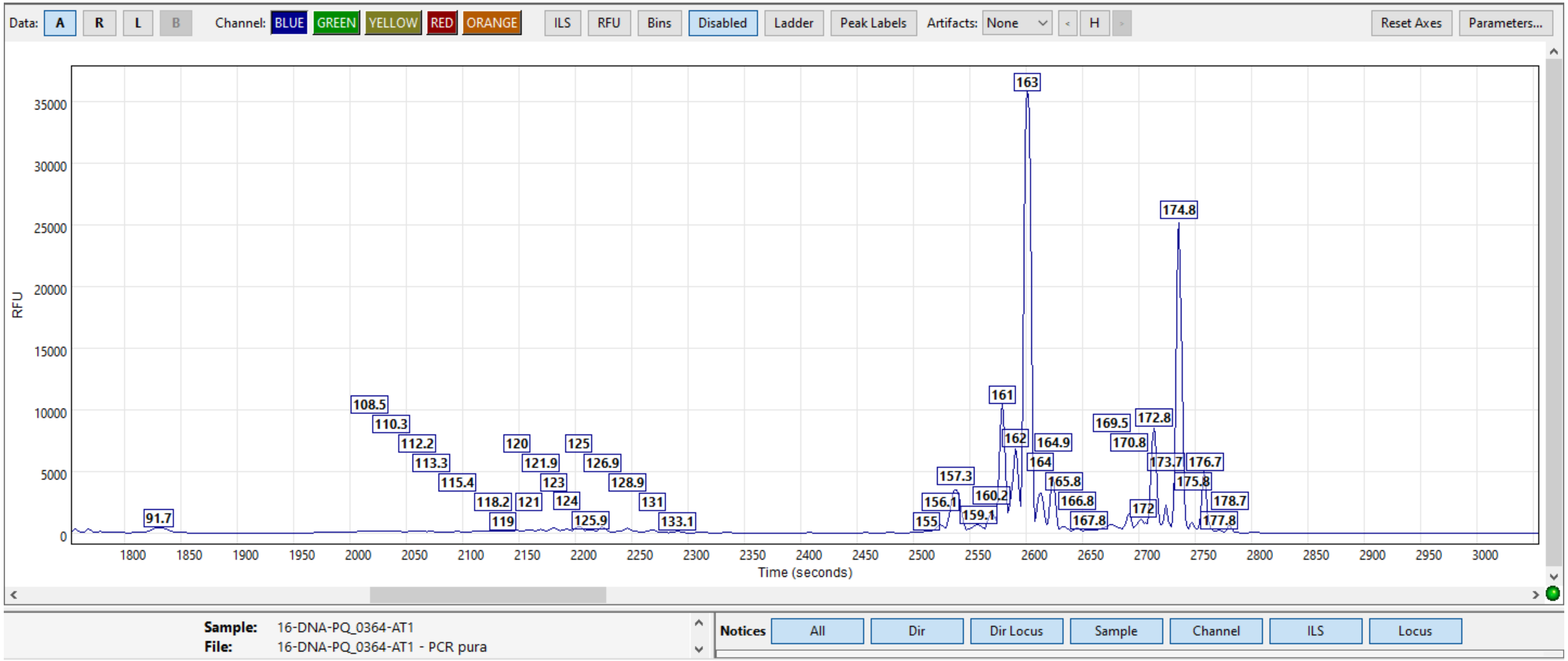


| | |
|---|---|
| **Observer 1** | **163;175** |
| **Observer 2** | **163;174.8** |
| **Observer 3** | **163;175** |

**13- Colony. Locus AT1 sample 17 (0365)**

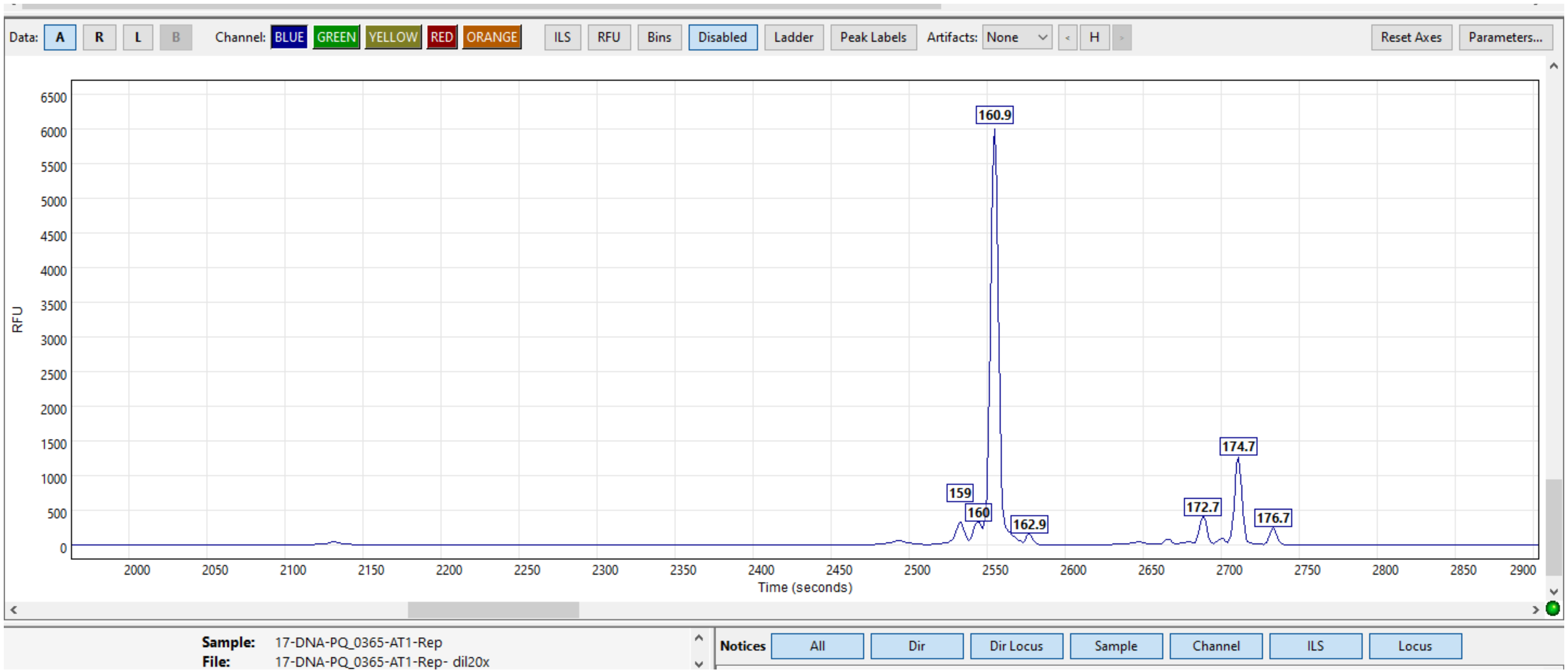


| | |
|---|---|
| **Observer 1** | **161;175** |
| **Observer 2** | **160.9;174.7** |
| **Observer 3** | **161;175** |

### 14- Colony. Locus AT1 sample 18 (0366)

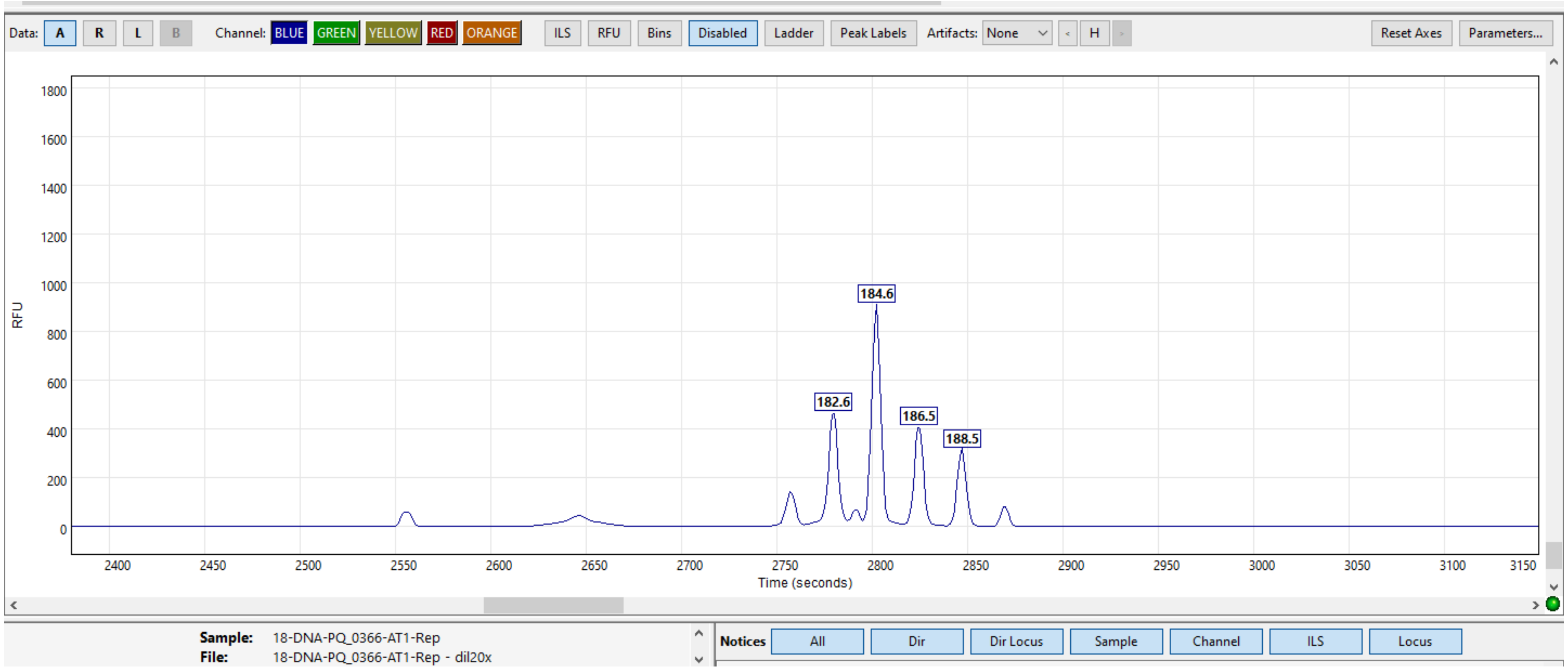


| | |
|---|---|
| **Observer 1** | **185** |
| **Observer 2** | **184.6** |
| **Observer 3** | **185** |

**15- Colony. Locus AT1 sample 20 (0923)**

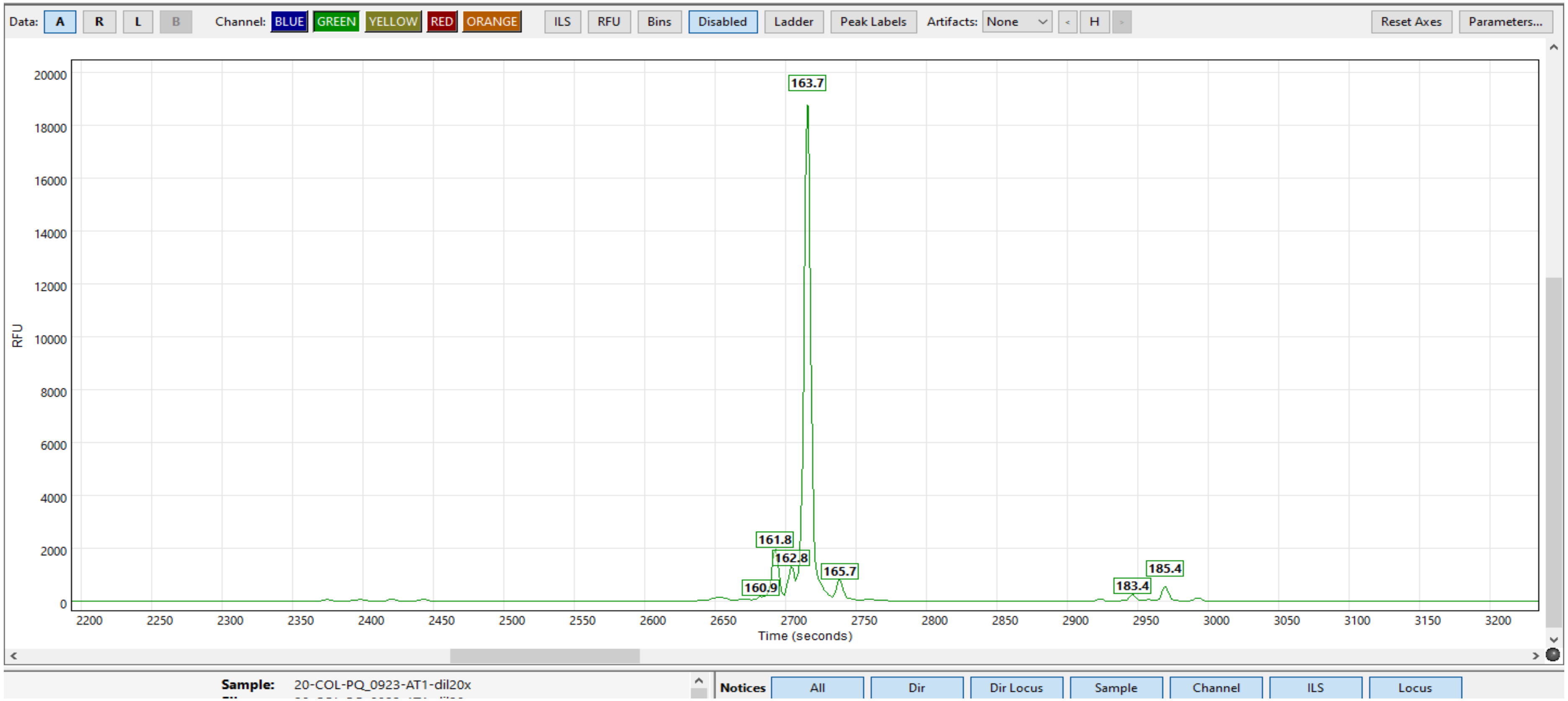


| | |
|---|---|
| **Observer 1** | **164** |
| **Observer 2** | **163.7** |
| **Observer 3** | **163** |

**Samples Wild. Locus AG2**

**1- Wild. Locus AG2 sample 01 (0909)**

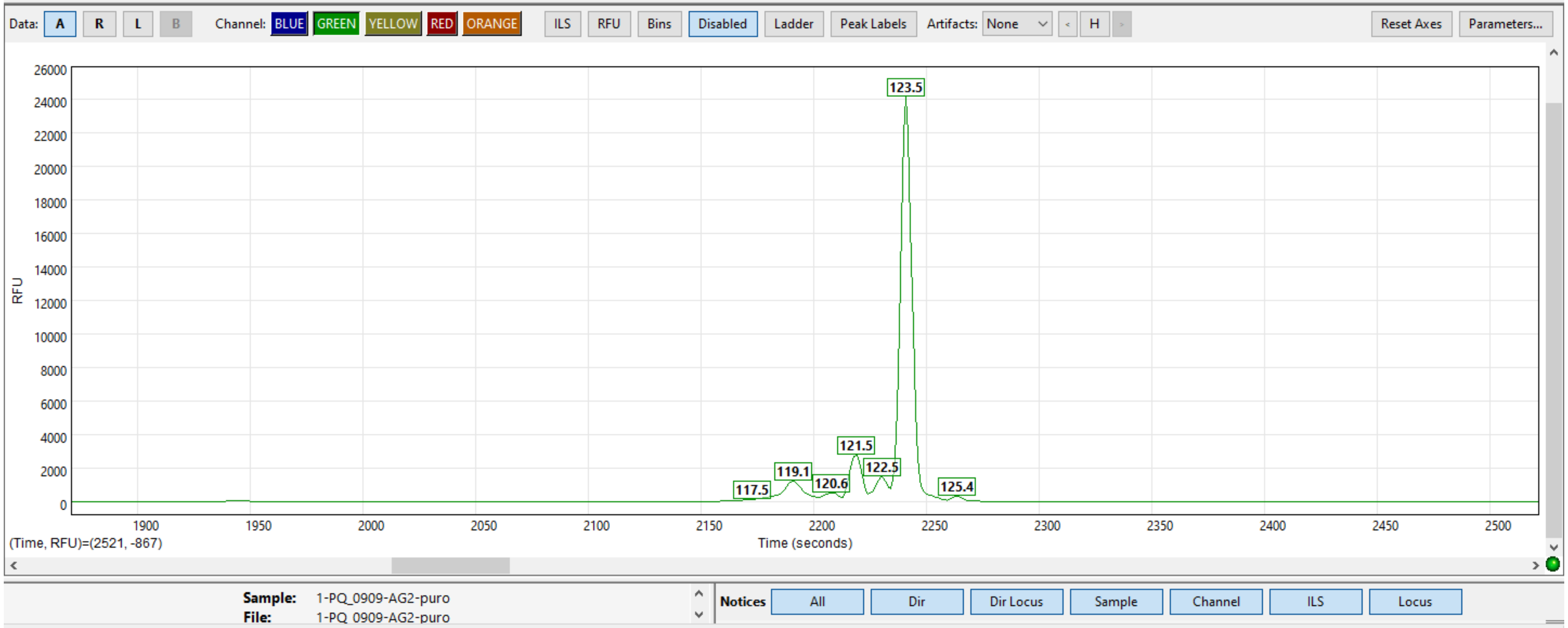


| | |
|---|---|
| **Observer 1** | **123** |
| **Observer 2** | **123.5** |
| **Observer 3** | **123** |

## 2- Wild. Locus AG2 sample 03 (0910)

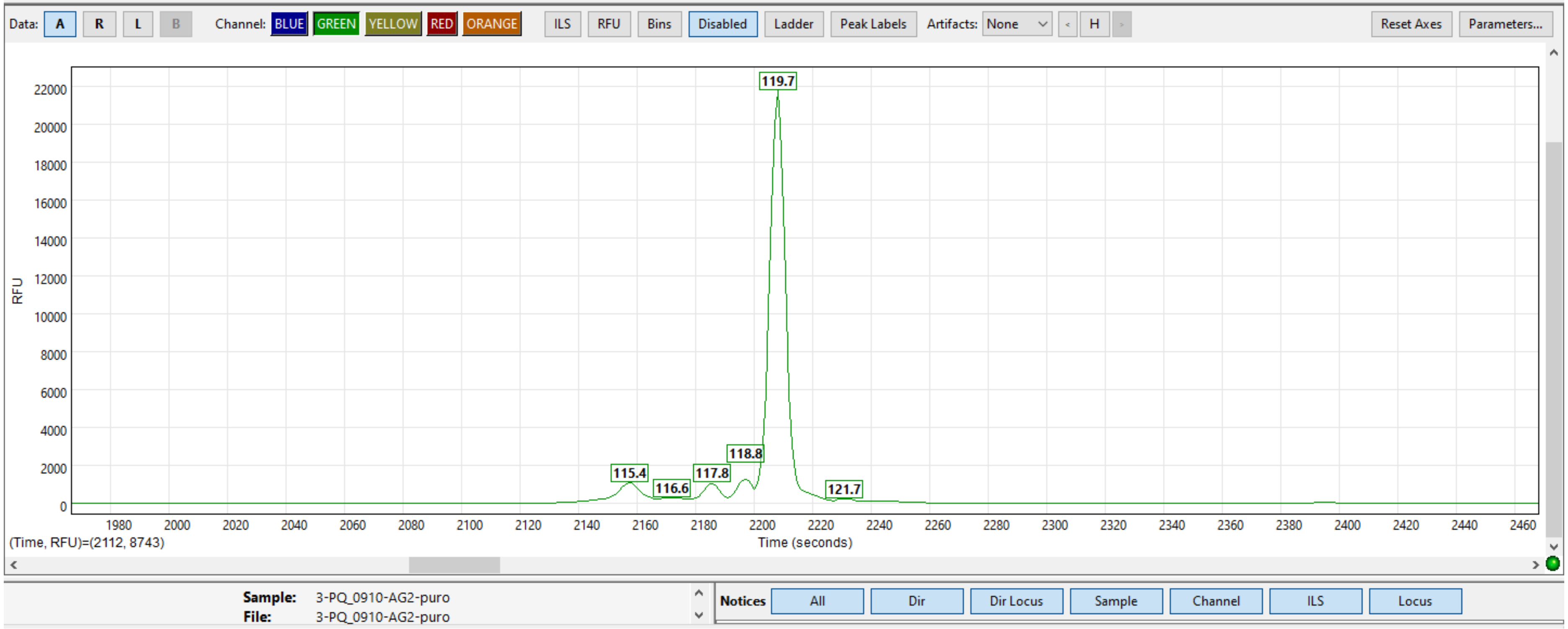


| | |
|---|---|
| **Observer 1** | **120** |
| **Observer 2** | **119.7** |
| **Observer 3** | **120** |

### 3- Wild. Locus AG2 sample 05 (0911)

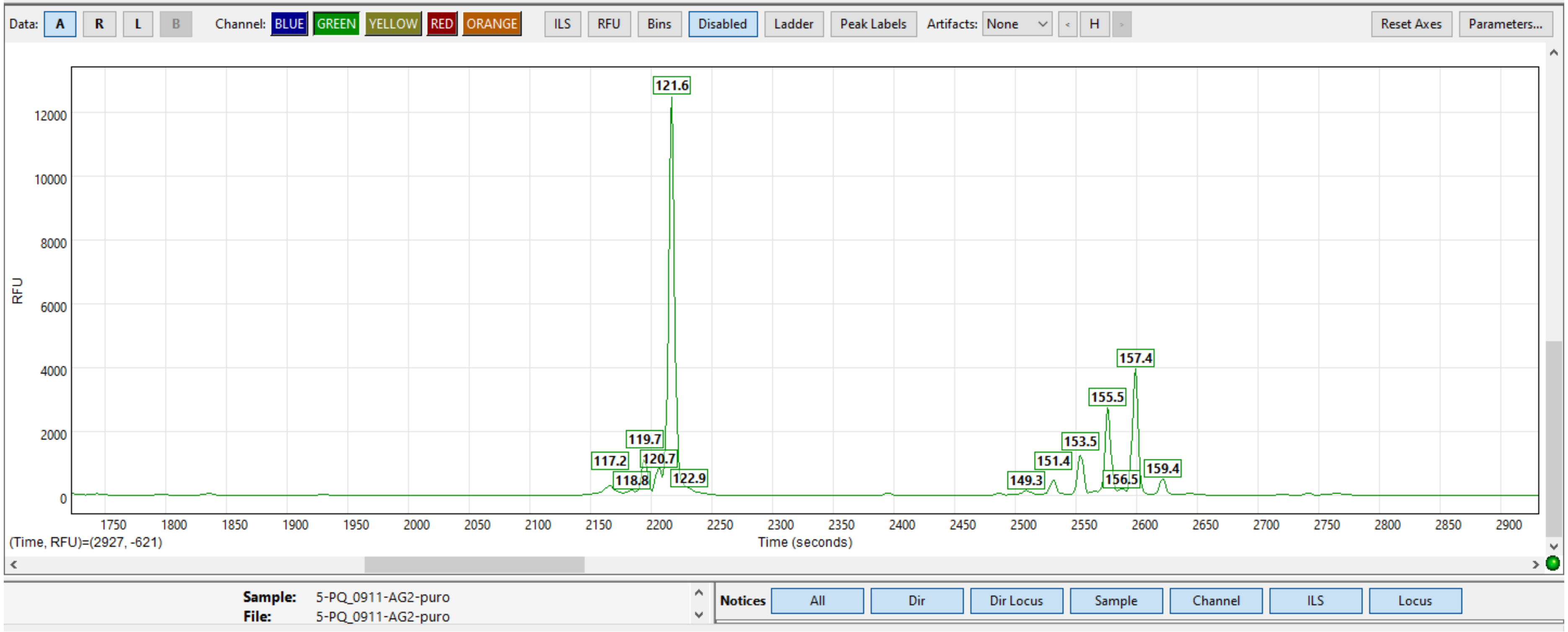


| | |
|---|---|
| **Observer 1** | **122;157** |
| **Observer 2** | **121.6;157.4** |
| **Observer 3** | **122;157** |

**4- Wild. Locus AG2 sample 07 (0912)**

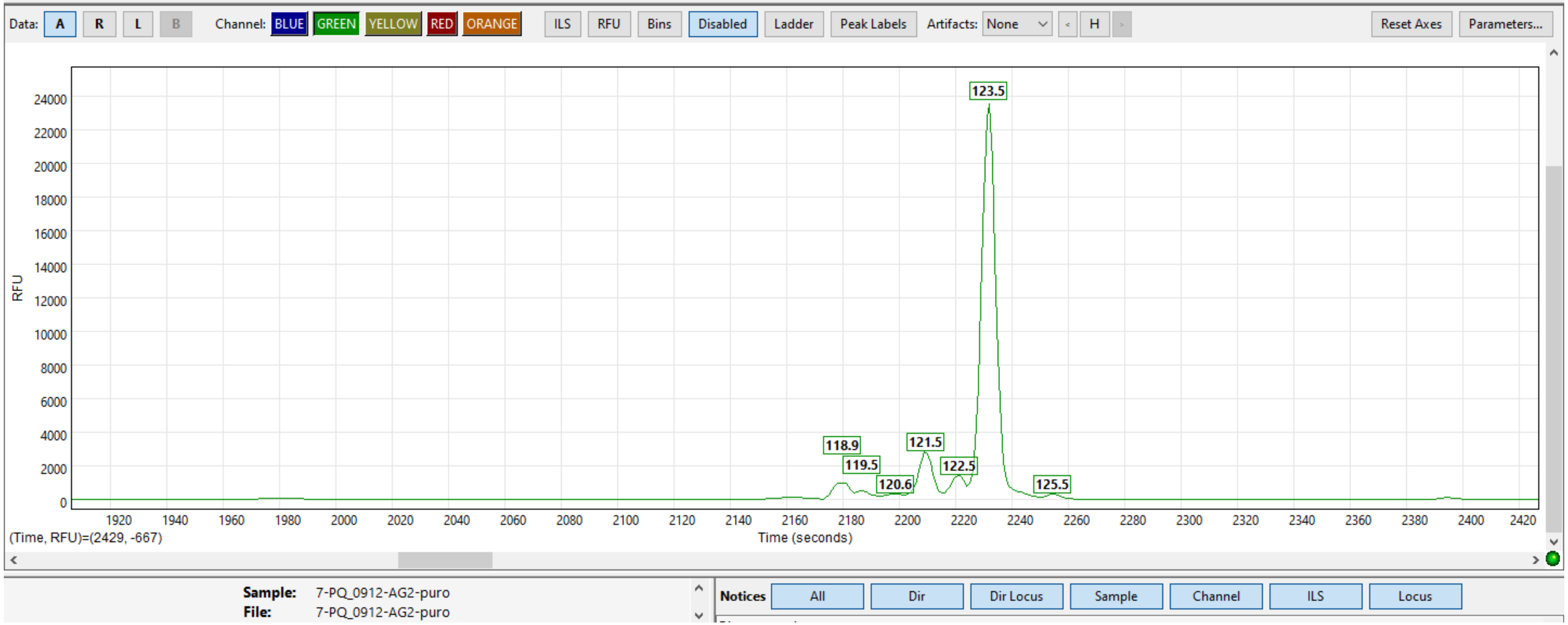


| | |
|---|---|
| **Observer 1** | **123** |
| **Observer 2** | **123.5** |
| **Observer 3** | **123** |

**5- Wild. Locus AG2 sample 08 (0913)**

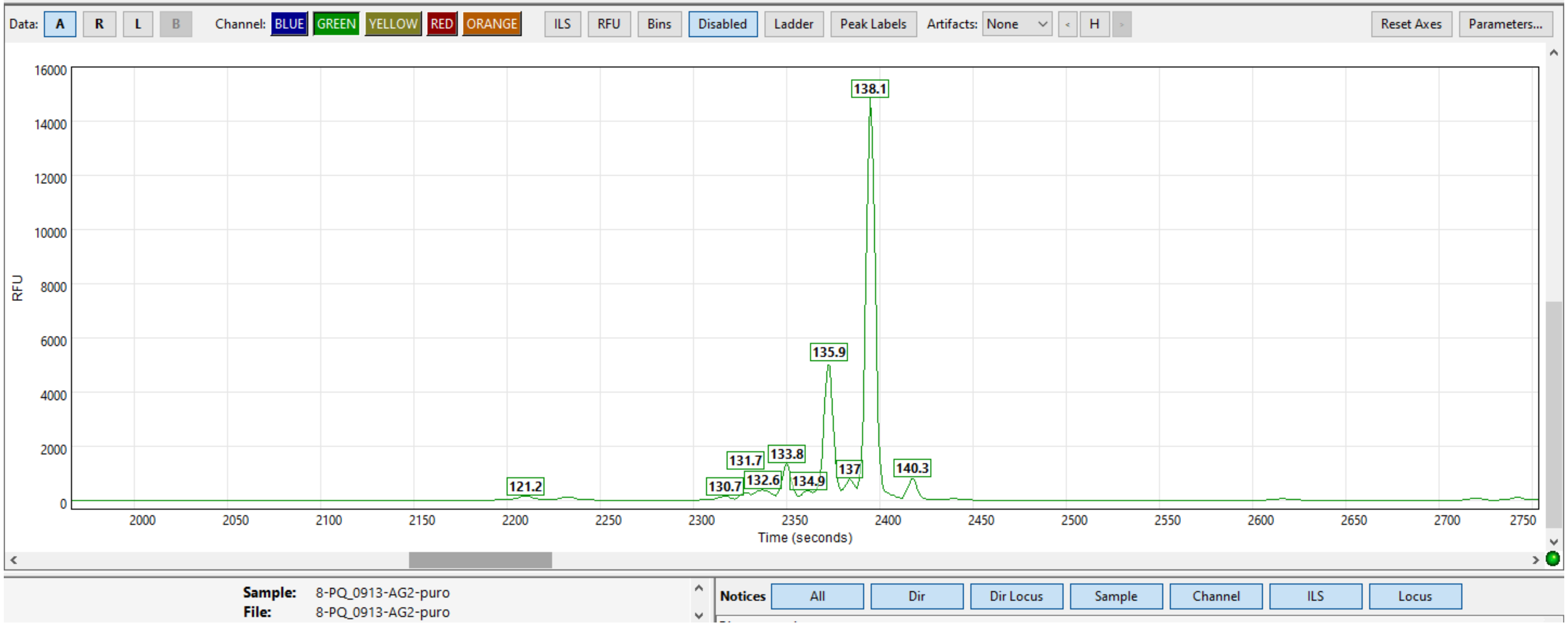


| | |
|---|---|
| **Observer 1** | **138** |
| **Observer 2** | **138.1** |
| **Observer 3** | **138** |

## 6- Wild. Locus AG2 sample 09 (0914)

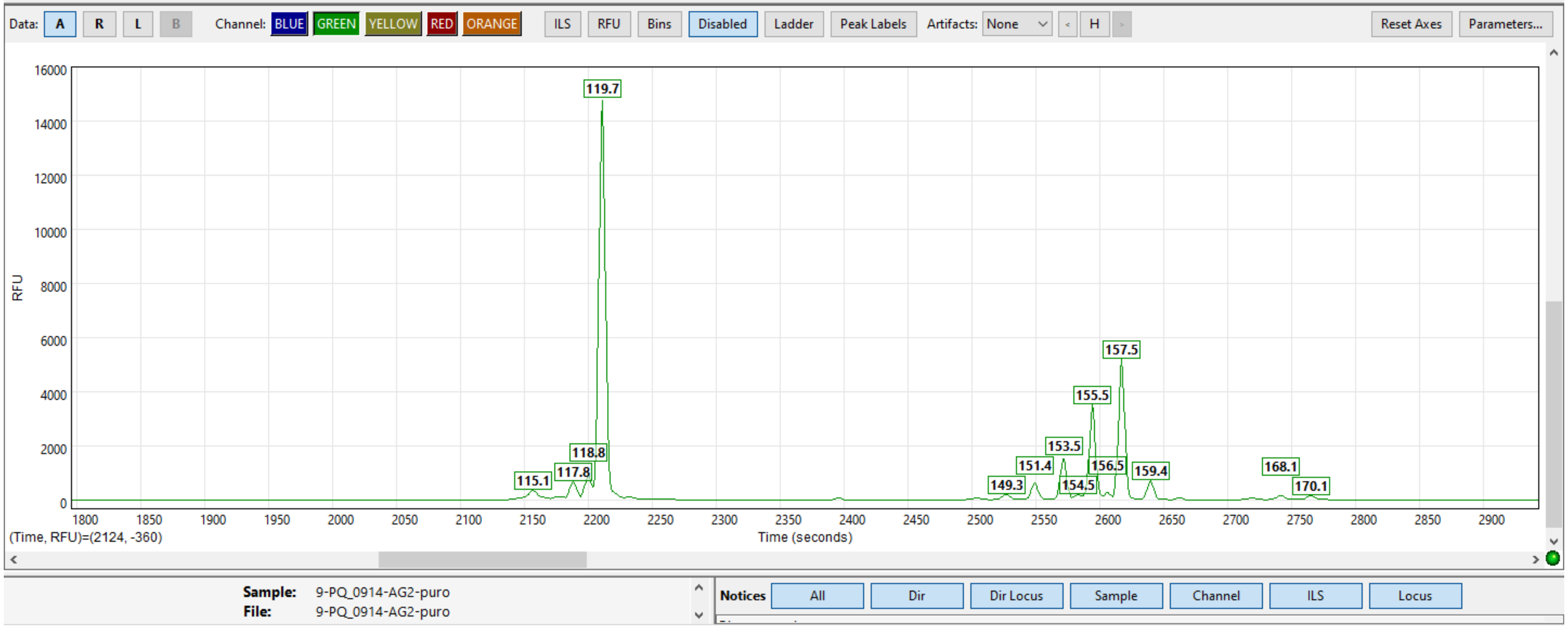


| | |
|---|---|
| **Observer 1** | **120;157** |
| **Observer 2** | **119.7;157.5** |
| **Observer 3** | **120;157** |

## 7- Wild. Locus AG2 sample 12 (0915)

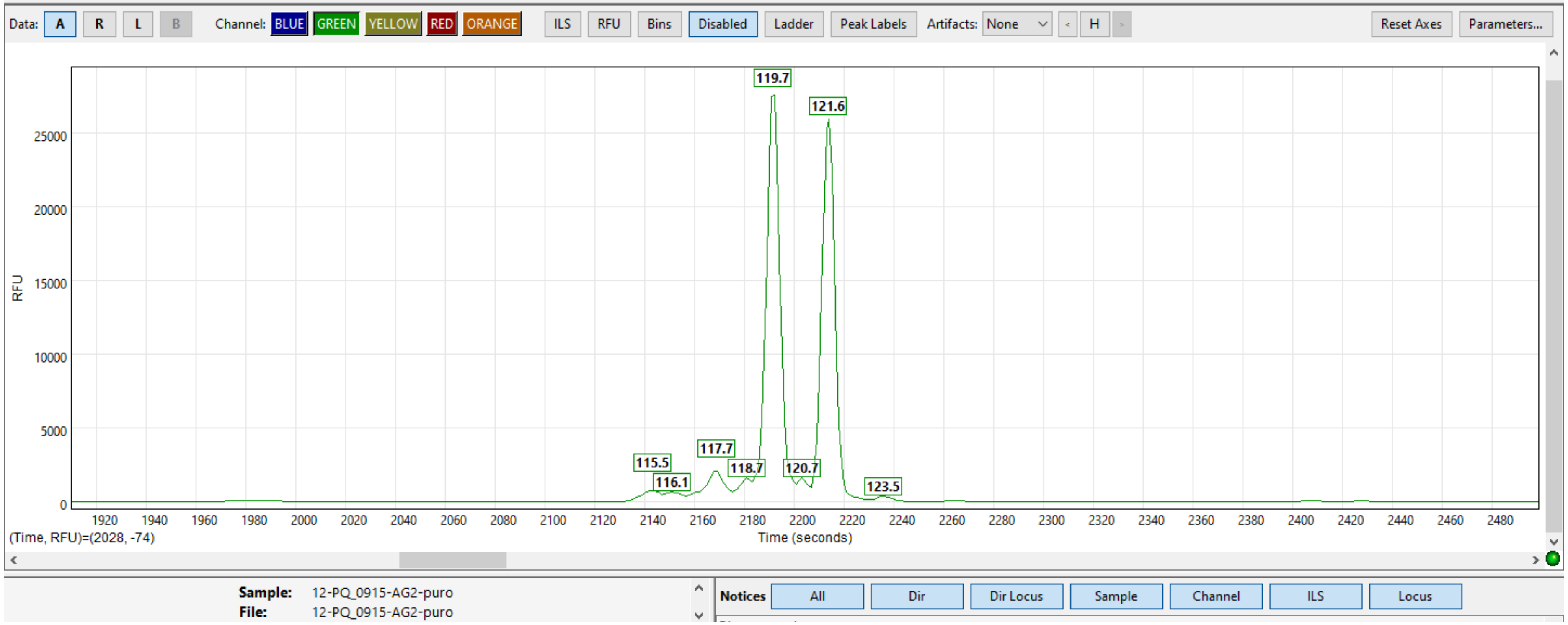


| | |
|---|---|
| **Observer 1** | **120;122** |
| **Observer 2** | **119.7;121.6** |
| **Observer 3** | **120;122** |

## 8- Wild. Locus AG2 sample 13 (0916)

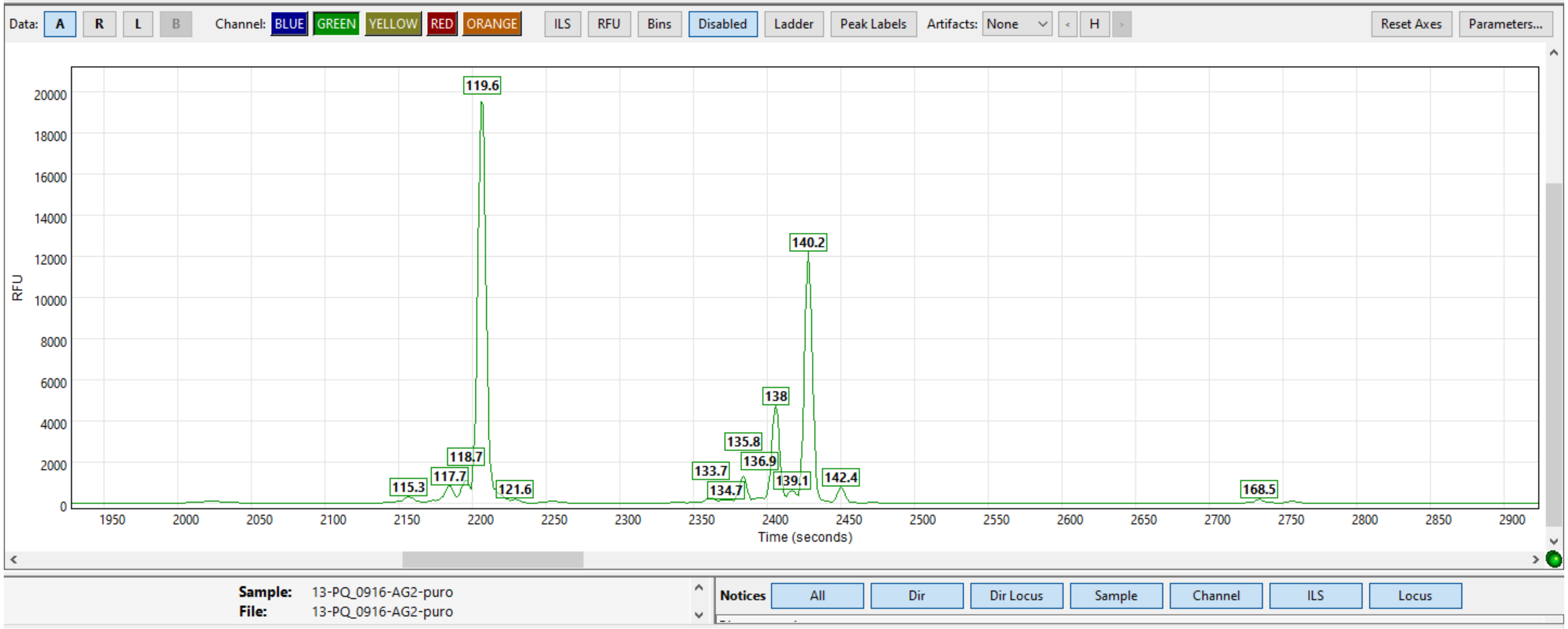


| | |
|---|---|
| **Observer 1** | **120;140** |
| **Observer 2** | **119.6;140.2** |
| **Observer 3** | **120;140** |

## 9- Wild. Locus AG2 sample 14 (0917)

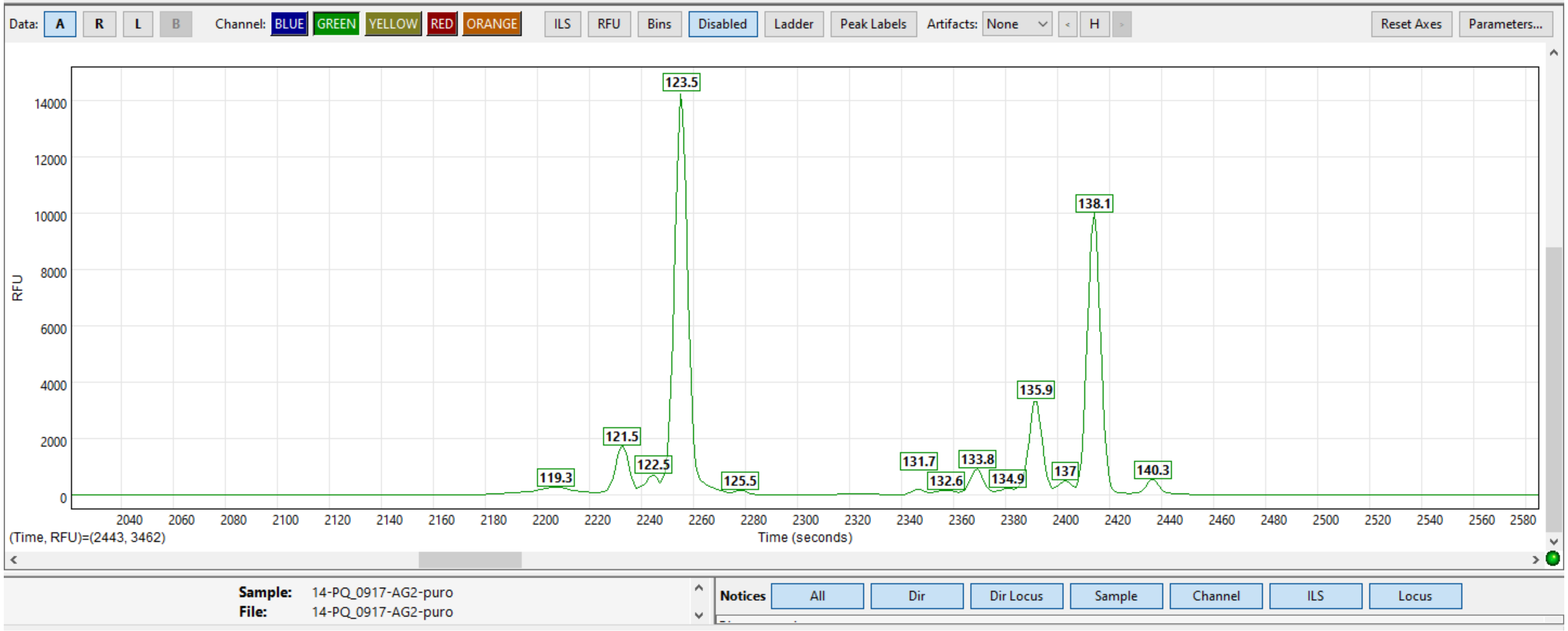


| | |
|---|---|
| **Observer 1** | **123;138** |
| **Observer 2** | **123.5;138.1** |
| **Observer 3** | **123;138** |

## 10- Wild. Locus AG2 sample 15 (0918)

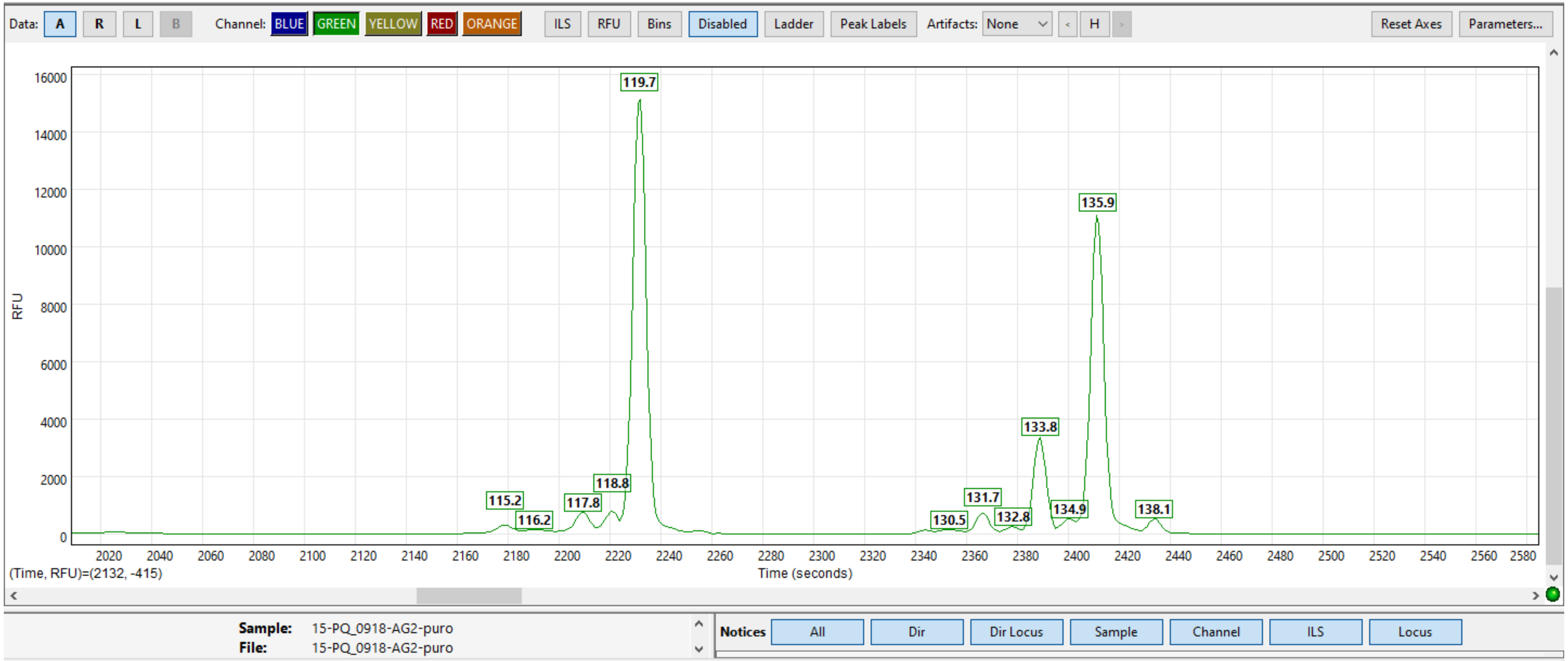


| | |
|---|---|
| **Observer 1** | **120;136** |
| **Observer 2** | **119.7;135.9** |
| **Observer 3** | **120;136** |

## 11- Wild. Locus AG2 sample 16 (0919)

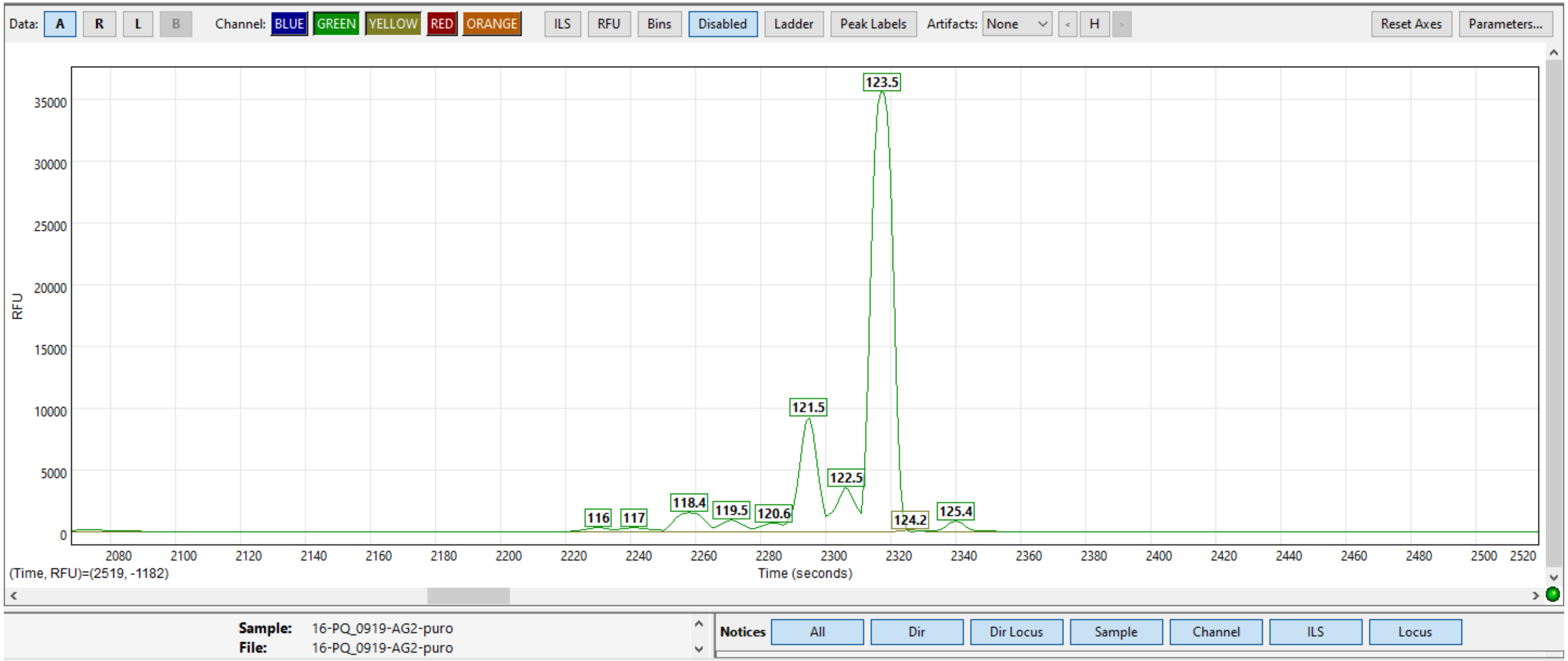


| | |
|---|---|
| **Observer 1** | **123** |
| **Observer 2** | **123.5** |
| **Observer 3** | **123** |

## 12- Wild. Locus AG2 sample 17 (0920)

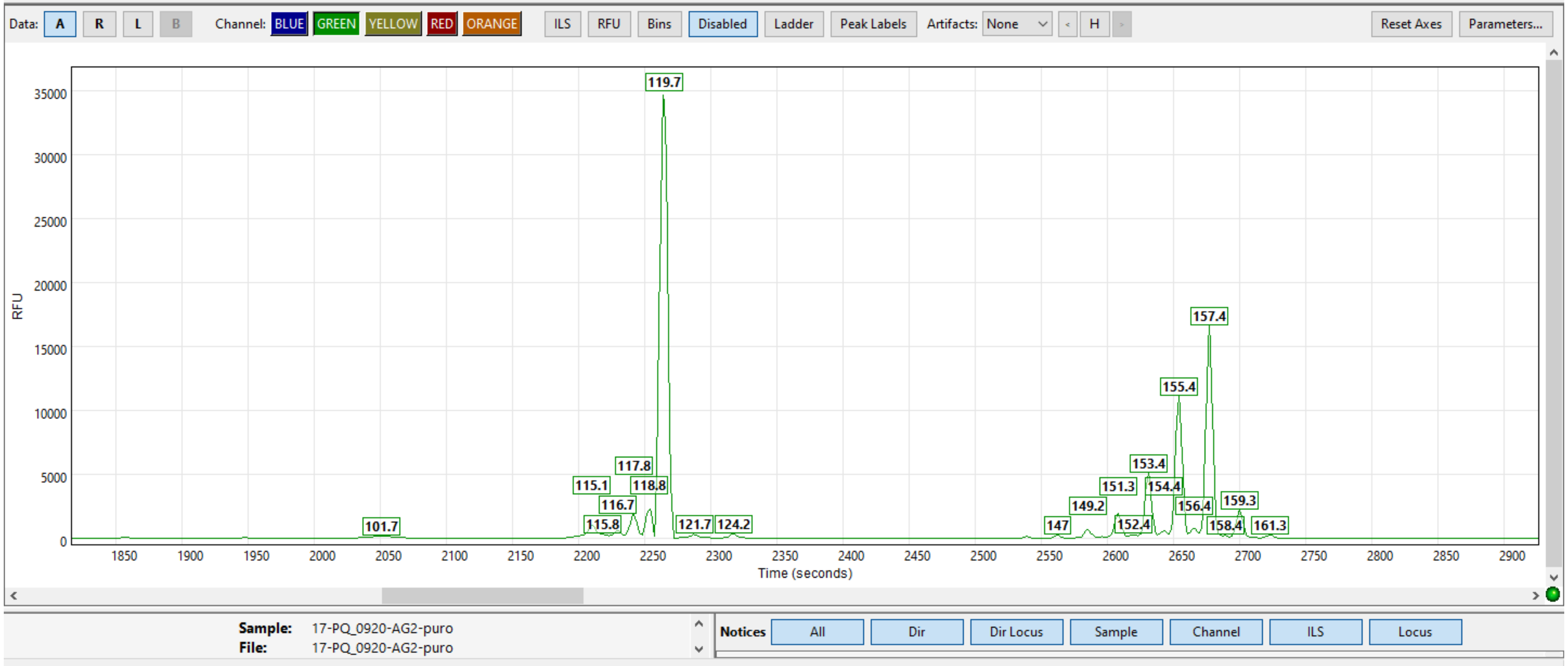


| | |
|---|---|
| **Observer 1** | **120;157** |
| **Observer 2** | **119.7;157.4** |
| **Observer 3** | **120;157** |

**13- Wild. Locus AG2 sample 19 (0921)**

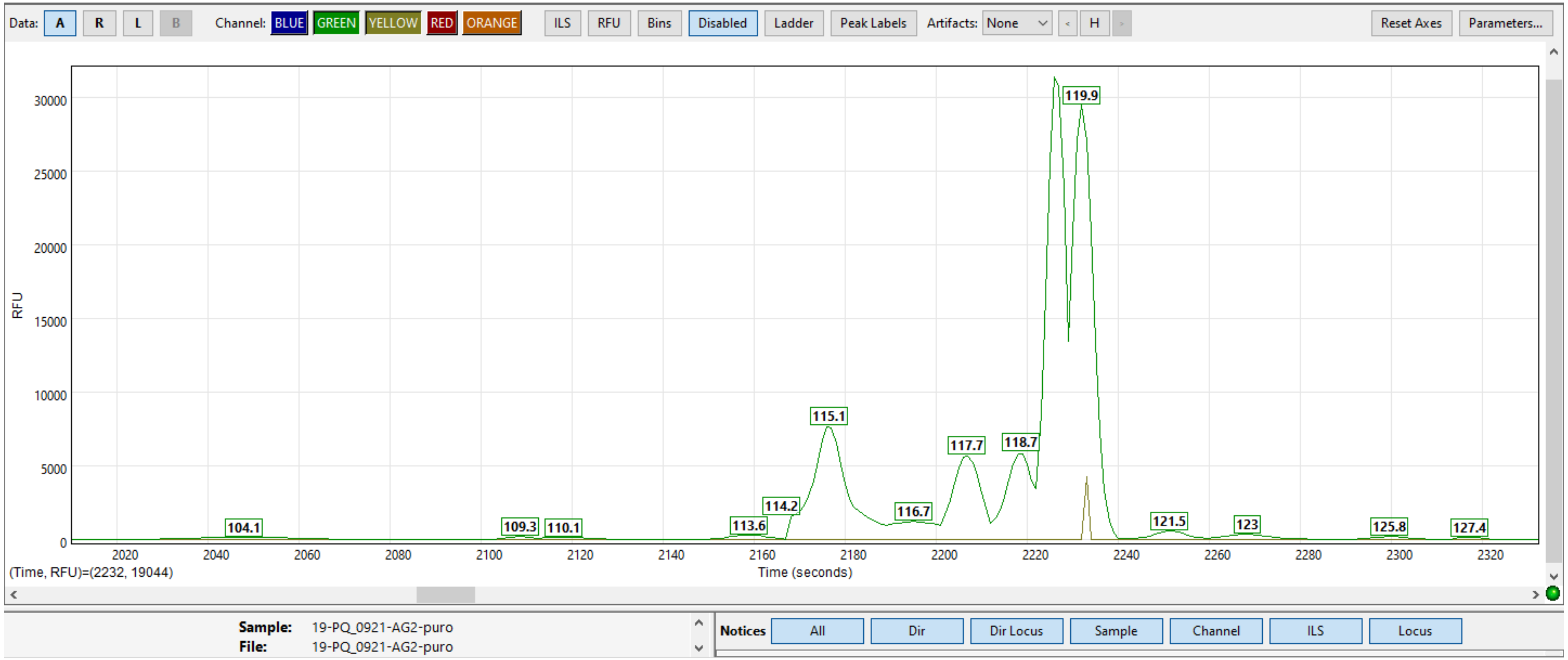


| | |
|---|---|
| **Observer 1** | **120** |
| **Observer 2** | **119.9** |
| **Observer 3** | **120** |

**14- Wild. Locus AG2 sample 20 (0922)**

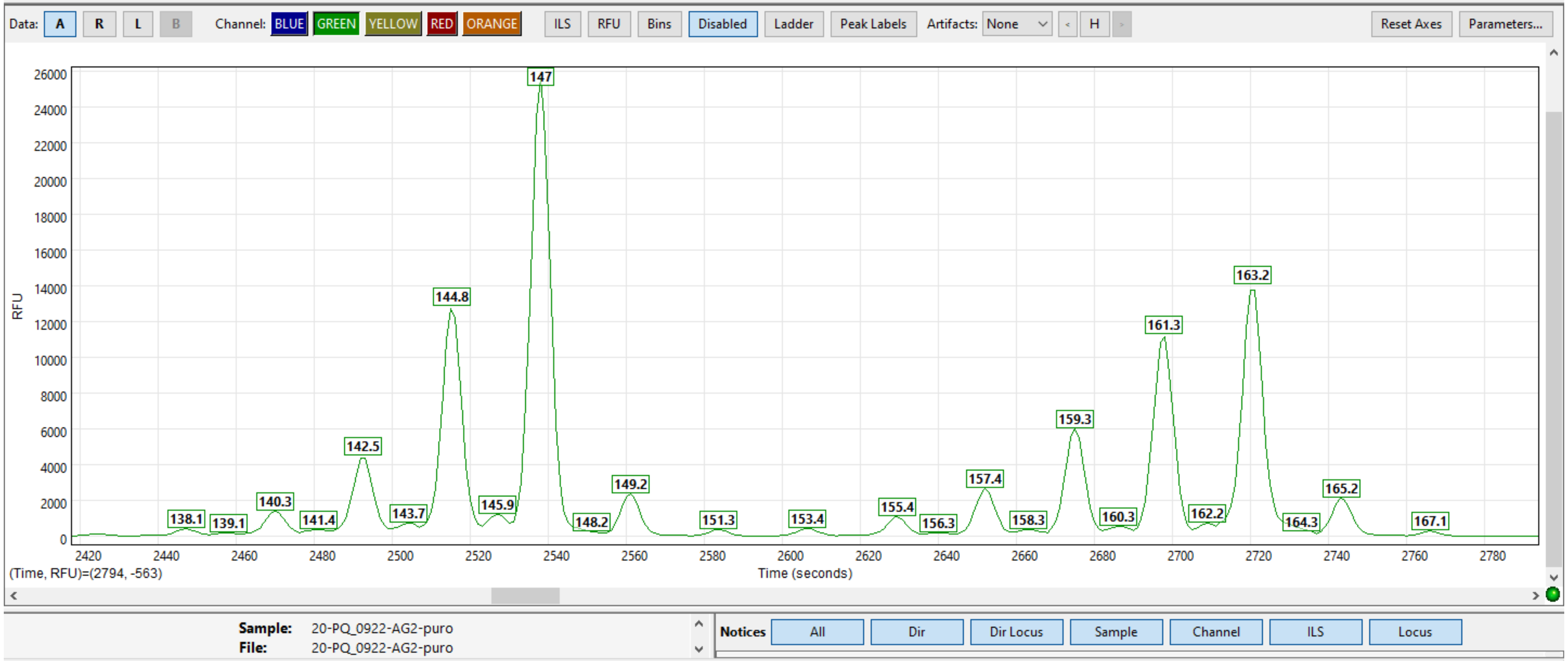


| | |
|---|---|
| **Observer 1** | **147;163** |
| **Observer 2** | **147;163.2** |
| **Observer 3** | **147;163** |

## 15- Wild. Locus AG2 sample 21 (0924)

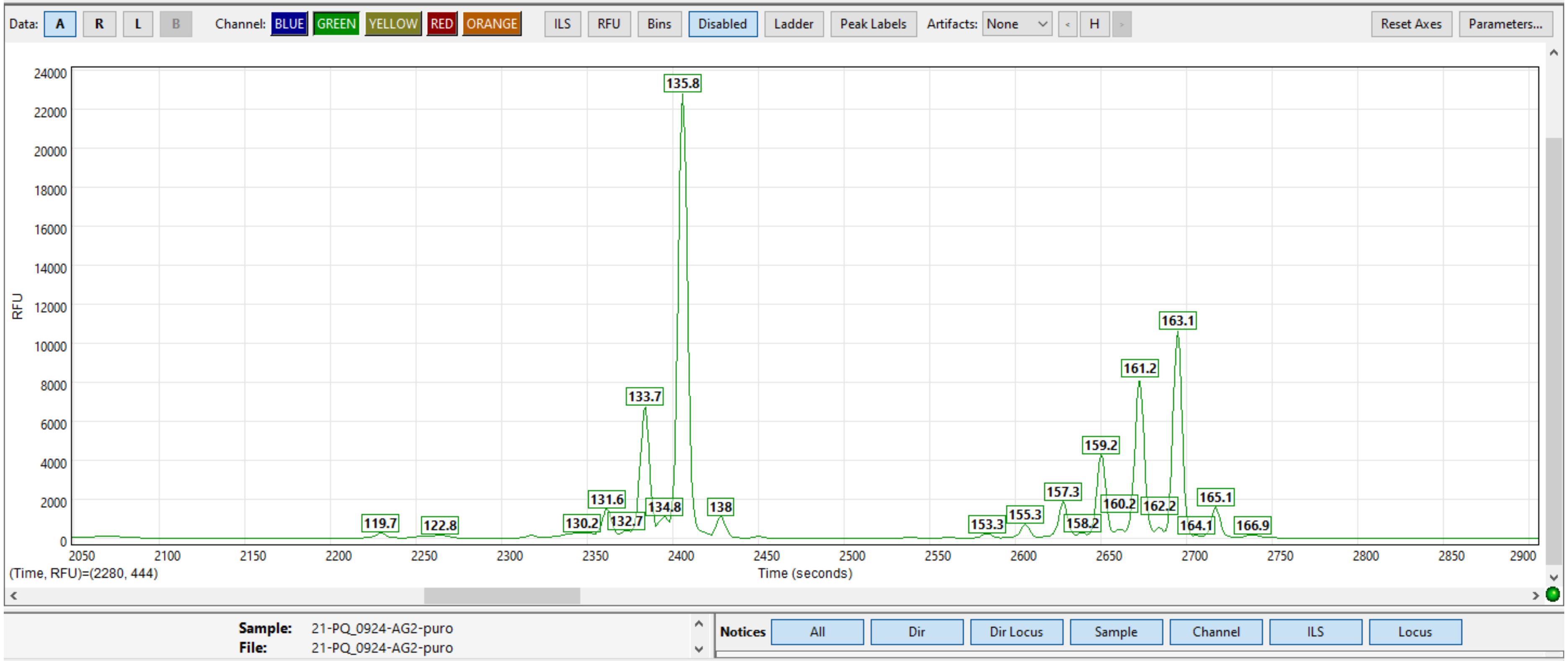


| | |
|---|---|
| **Observer 1** | **136;163** |
| **Observer 2** | **135.8;163.1** |
| **Observer 3** | **136;163** |

## 16- Wild. Locus AG2 sample CAST2 (0144)

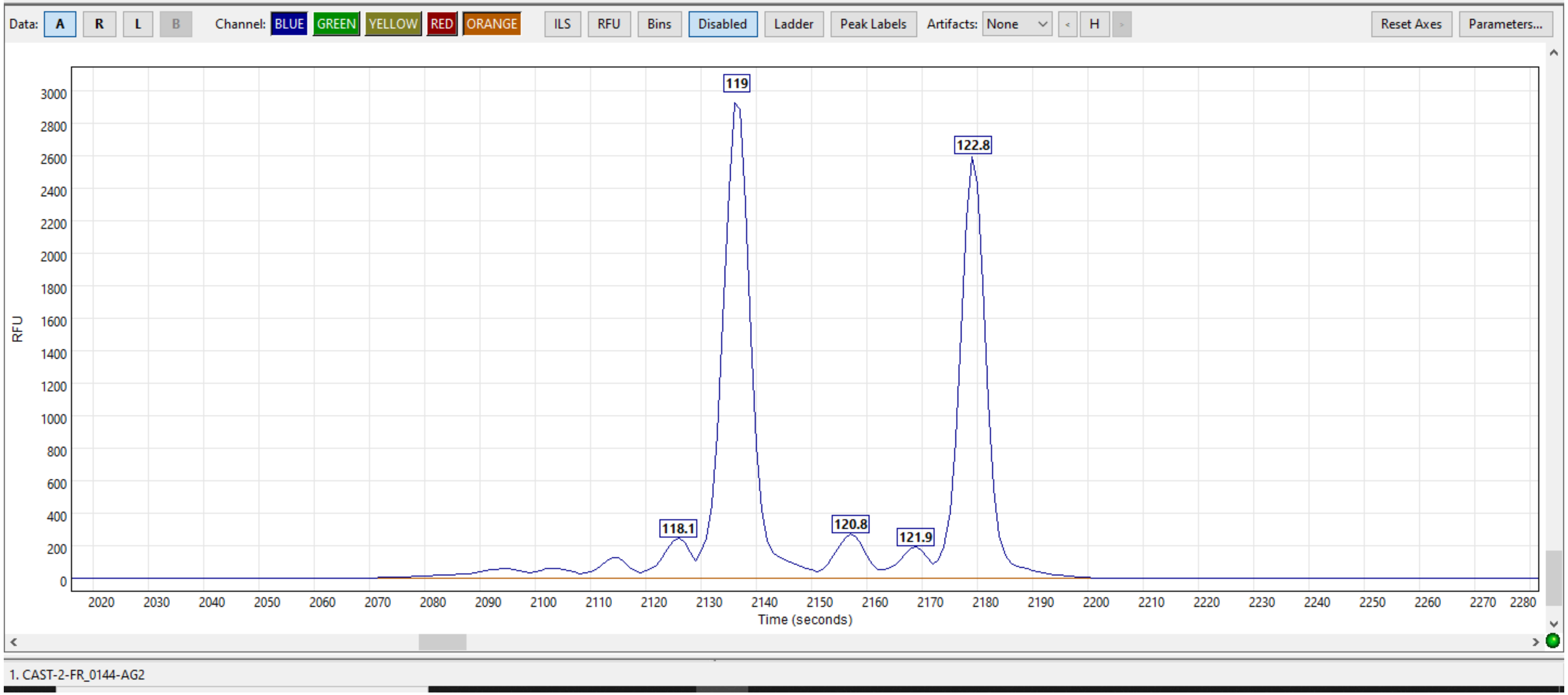


| | |
|---|---|
| **Observer 1** | **119;123** |
| **Observer 2** | **119;122.8** |
| **Observer 3** | **119;123** |

**17- Wild. Locus AG2 sample CAST3 (0145)**

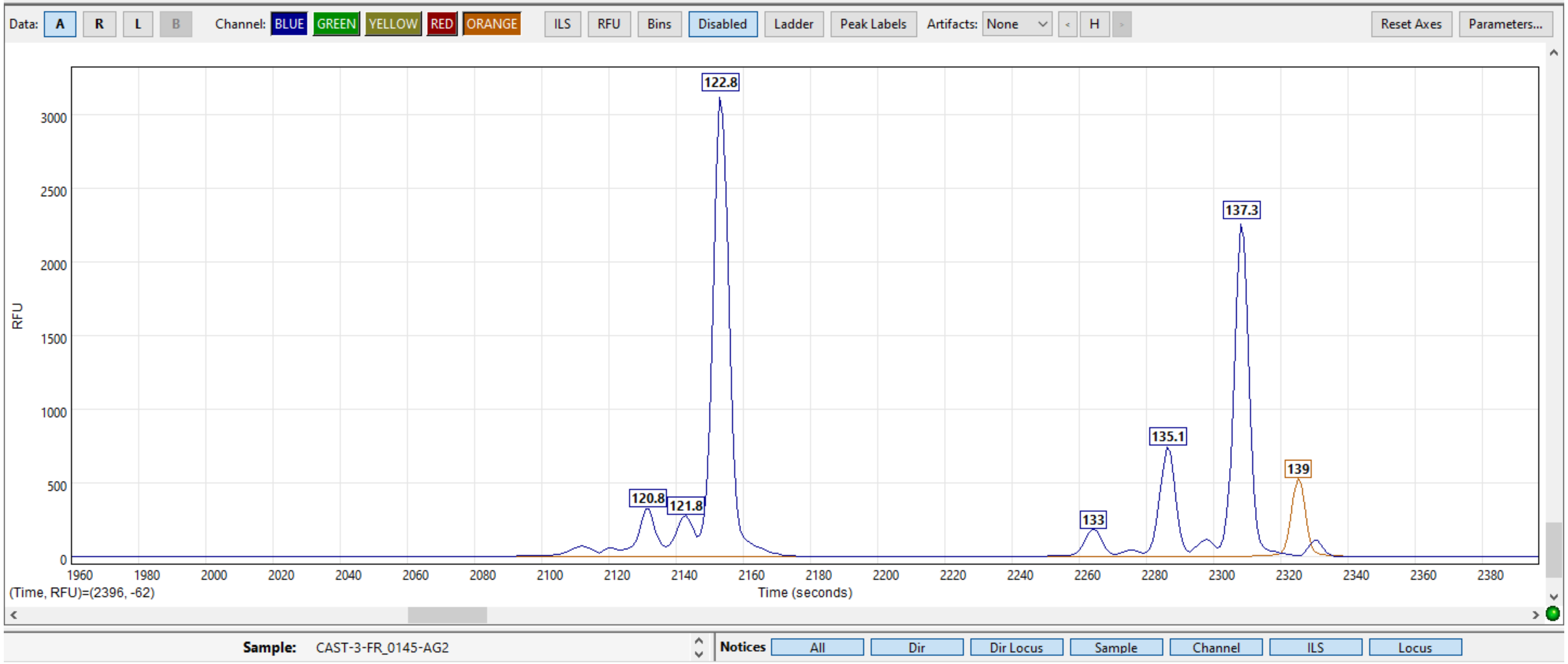


| | |
|---|---|
| **Observer 1** | **123;137** |
| **Observer 2** | **122.8;137.3** |
| **Observer 3** | **123. 137** |

**18- Wild. Locus AG2 sample CENT146 (0146)**

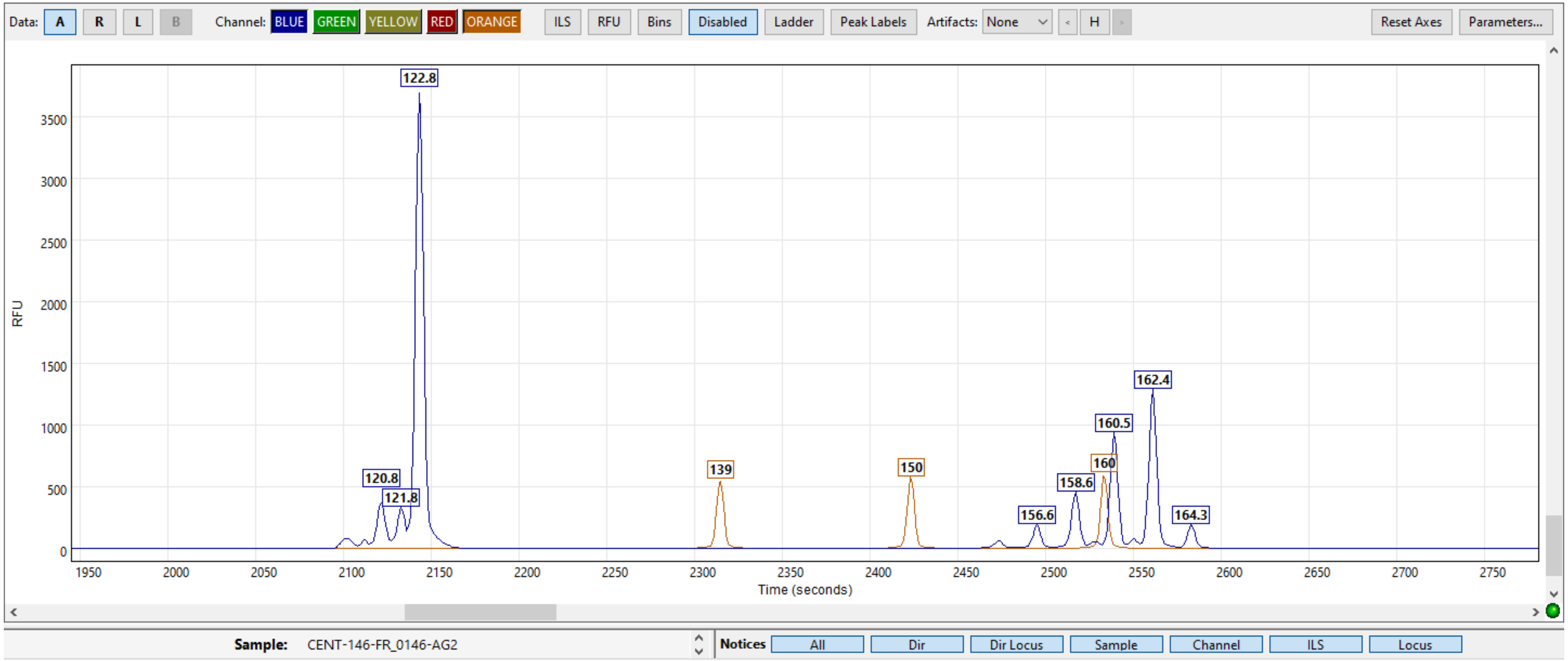


| | |
|---|---|
| **Observer 1** | **123;162** |
| **Observer 2** | **122.8;162.4** |
| **Observer 3** | **122.8;162.4** |

**19- Wild. Locus AG2 sample CENT151 (0147)**

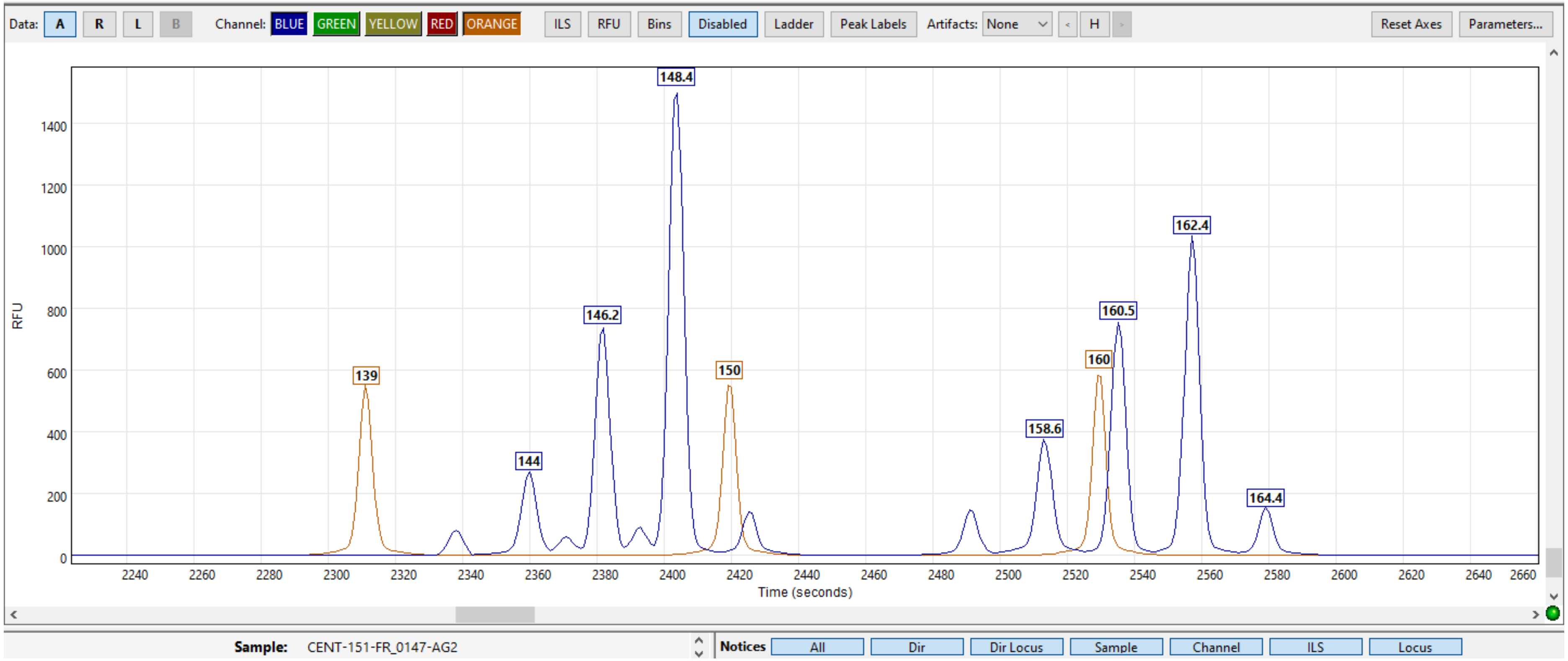


| | |
|---|---|
| **Observer 1** | **148;162** |
| **Observer 2** | **148.4;162.4** |
| **Observer 3** | **148.4;162.4** |

## 20- Wild. Locus AG2 sample CENT152 (0148)

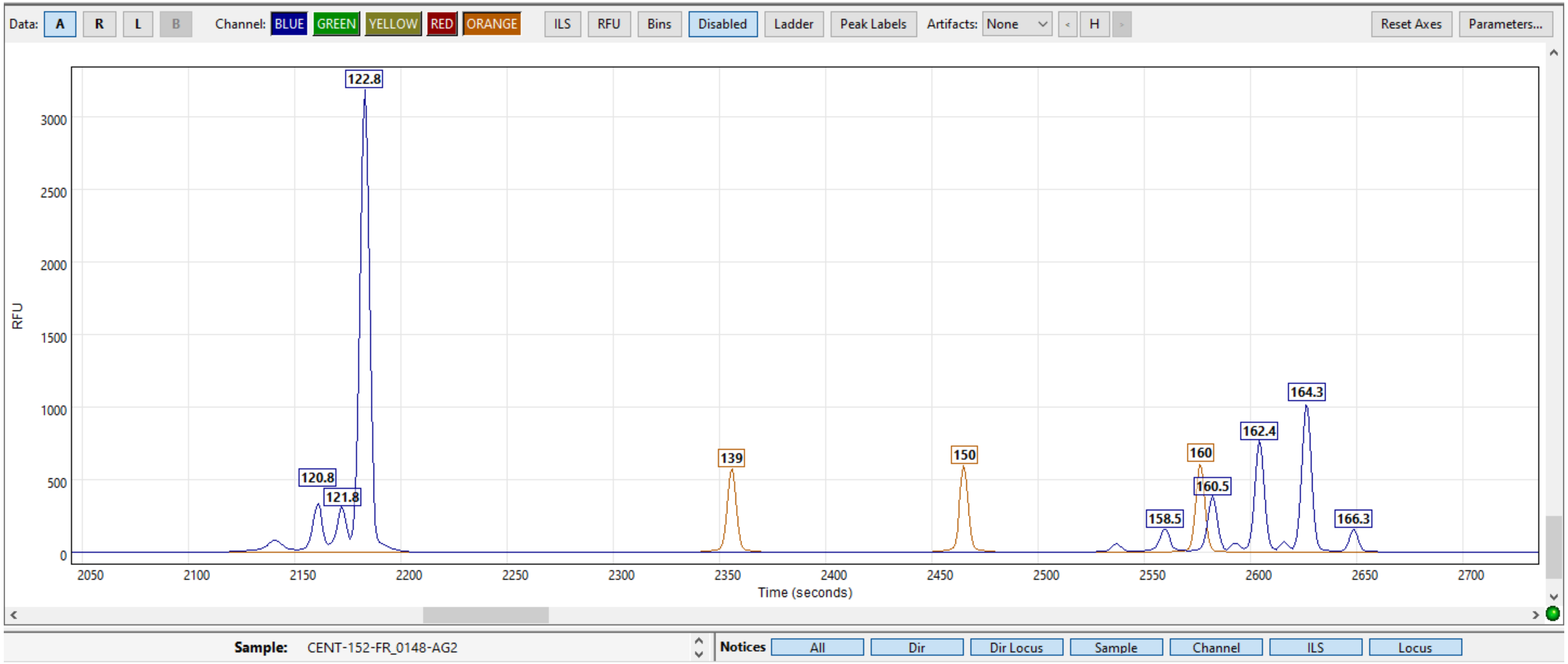


| | |
|---|---|
| **Observer 1** | **123;164** |
| **Observer 2** | **122.8;164.3** |
| **Observer 3** | **122.8;164.3** |

## 21- Wild. Locus AG2 sample CENT155 (0149)

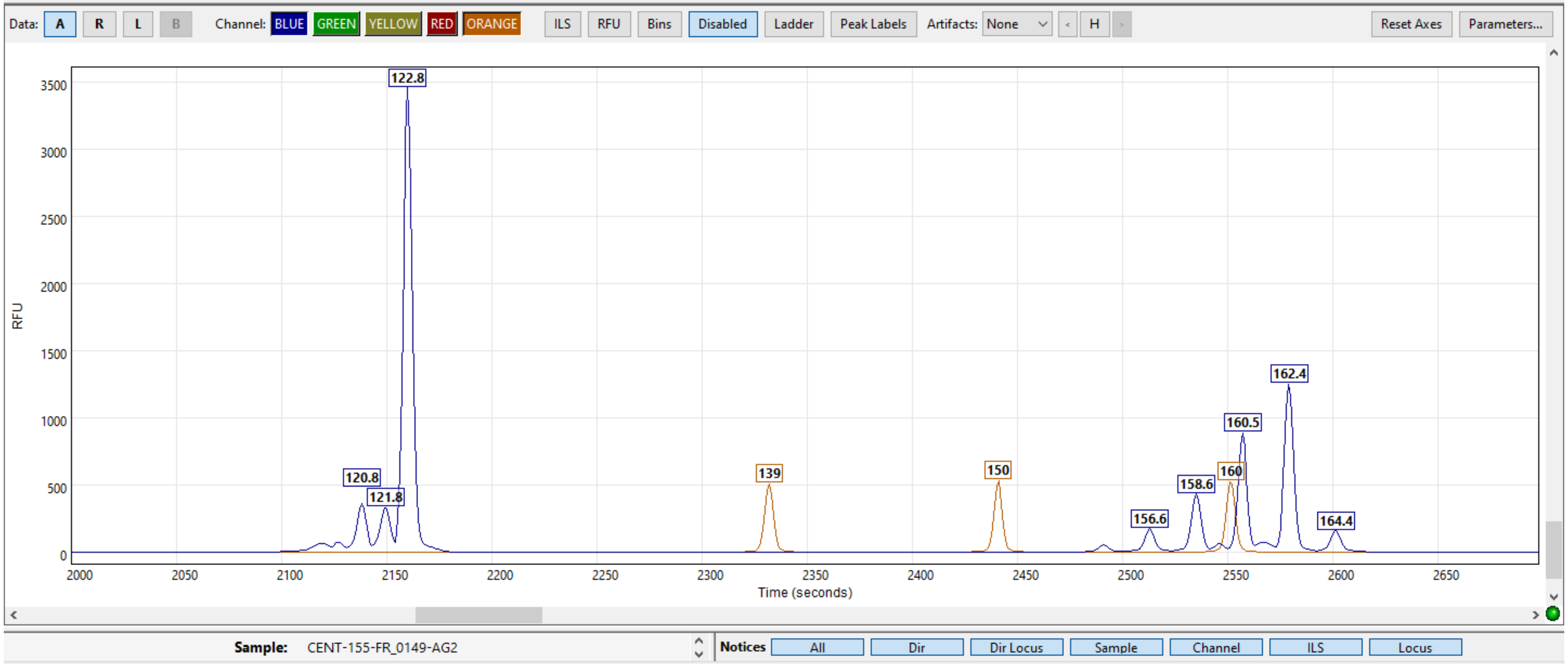


| | |
|---|---|
| **Observer 1** | **123;162** |
| **Observer 2** | **122.8;162.4** |
| **Observer 3** | **122.8;162.4** |

**22- Wild. Locus AG2 sample CENT157 (0150)**

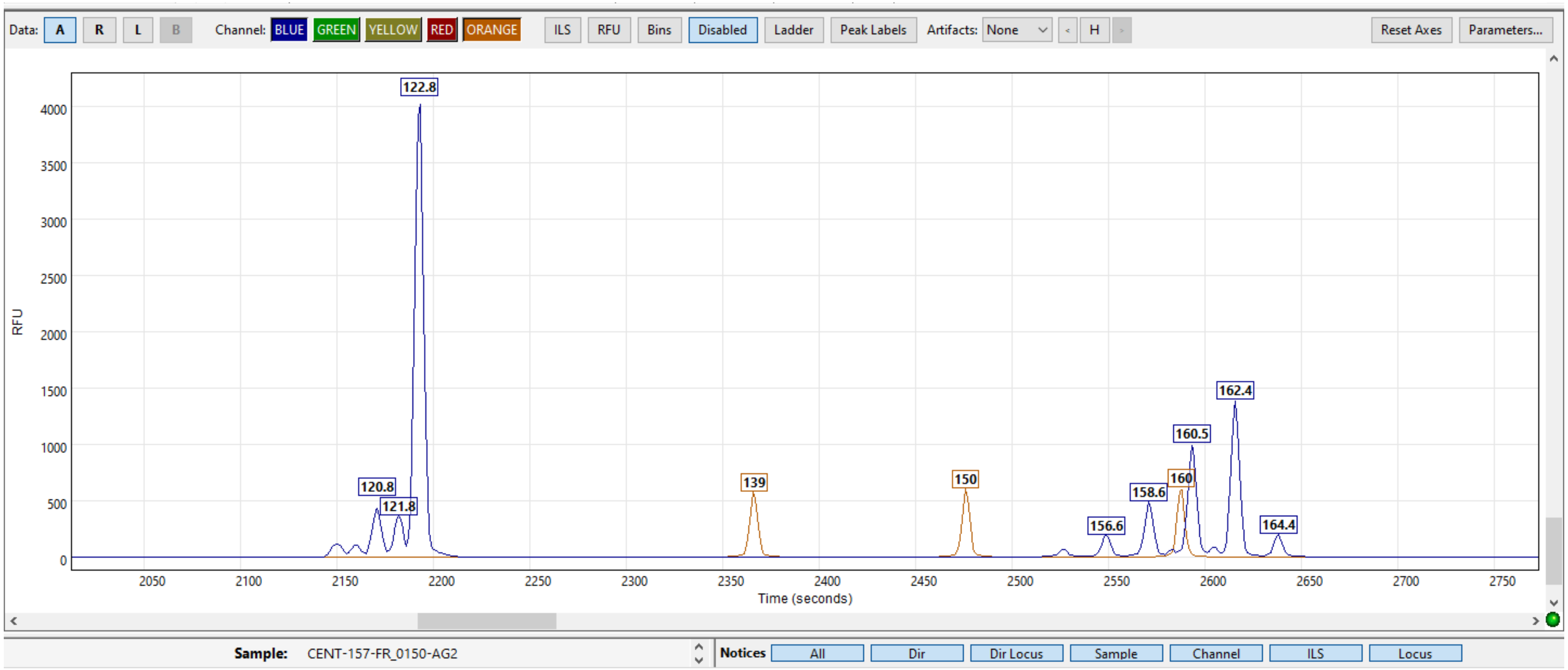


| | |
|---|---|
| **Observer 1** | **123;162** |
| **Observer 2** | **122.8;162.4** |
| **Observer 3** | **122.8;162.4** |

## 23- Wild. Locus AG2 sample CENT160 (0151)

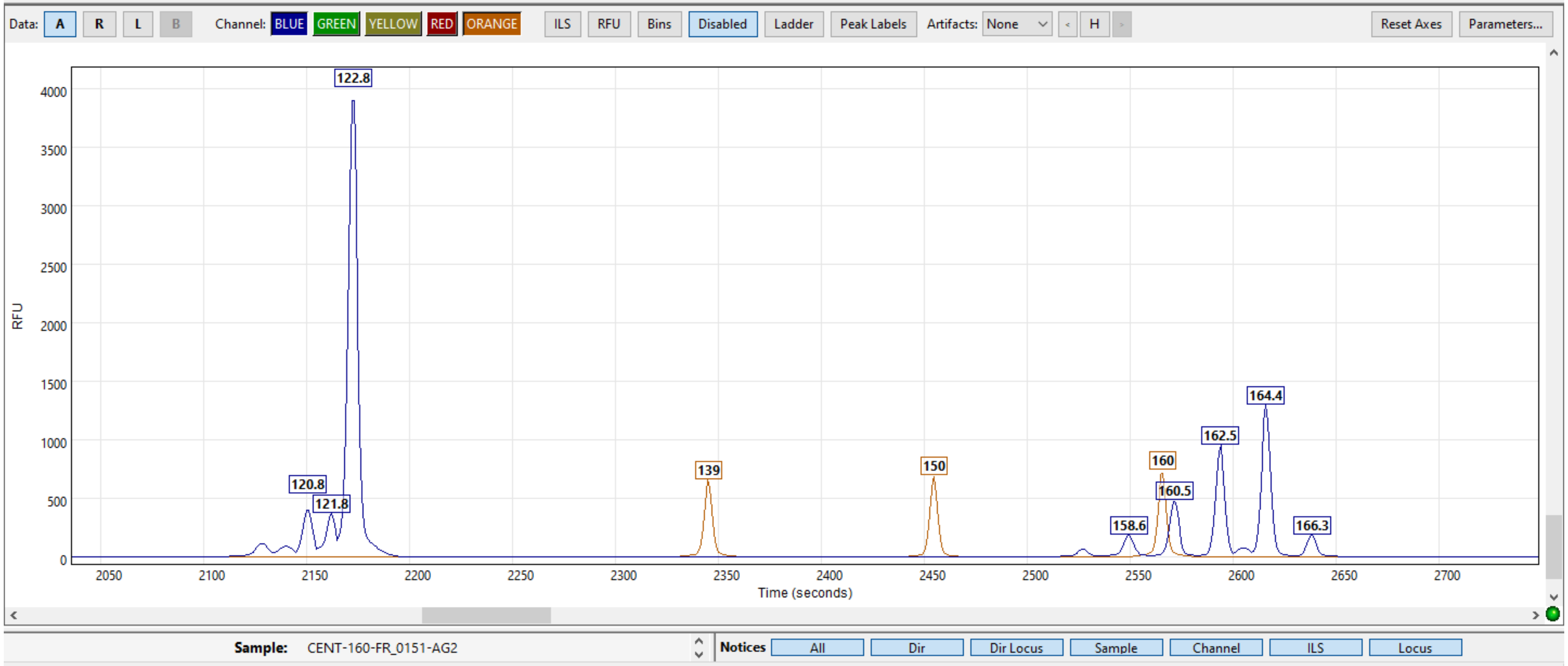


| | |
|---|---|
| **Observer 1** | **123;164** |
| **Observer 2** | **122.8;164.4** |
| **Observer 3** | **122.8;164.4** |

**24- Wild. Locus AG2 sample CENT162 (0152)**

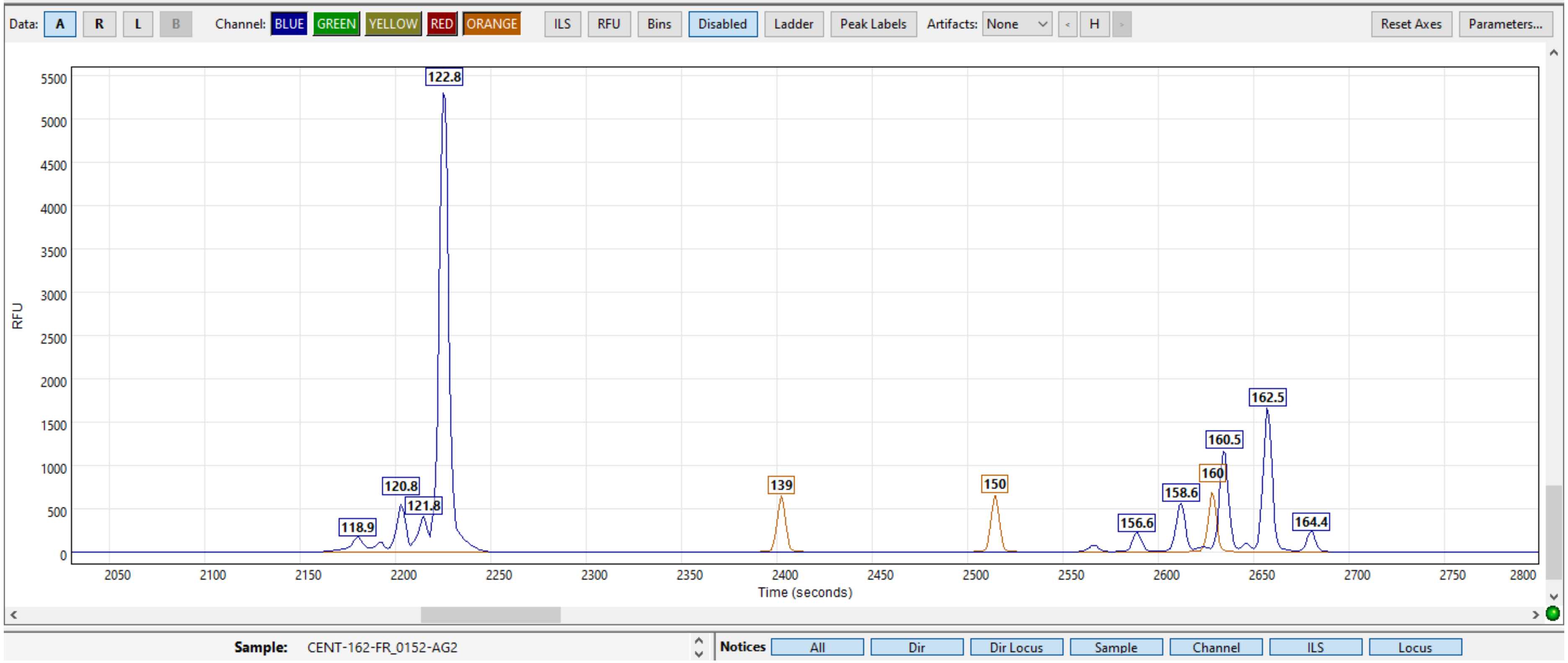


| | |
|---|---|
| **Observer 1** | **123;162.5** |
| **Observer 2** | **122.8;162.5** |
| **Observer 3** | **122.8;162.5** |

## 25- Wild. Locus AG2 sample GZGA10 (0153)

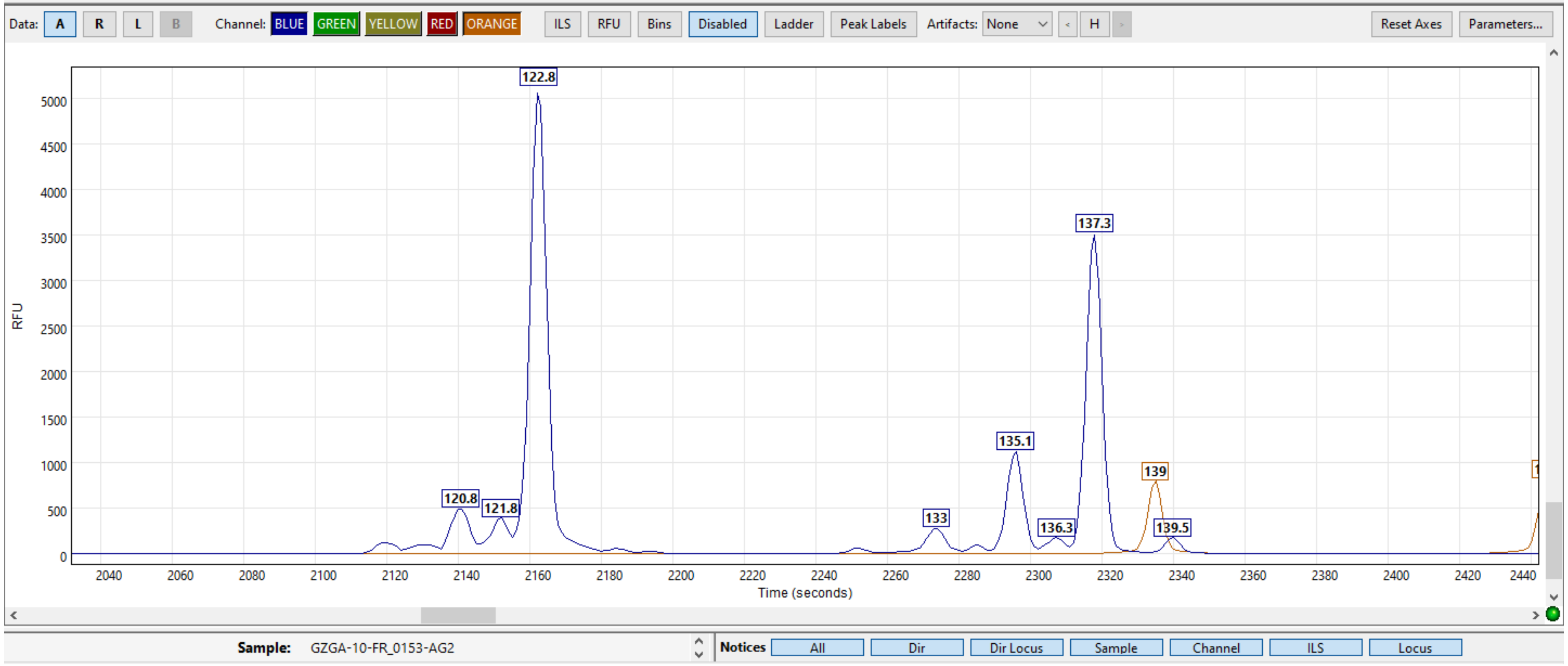


| | |
|---|---|
| **Observer 1** | **123;137** |
| **Observer 2** | **122.8;137.3** |
| **Observer 3** | **122.8;137.3** |

## 26- Wild. Locus AG2 sample GZGA8 (0154)

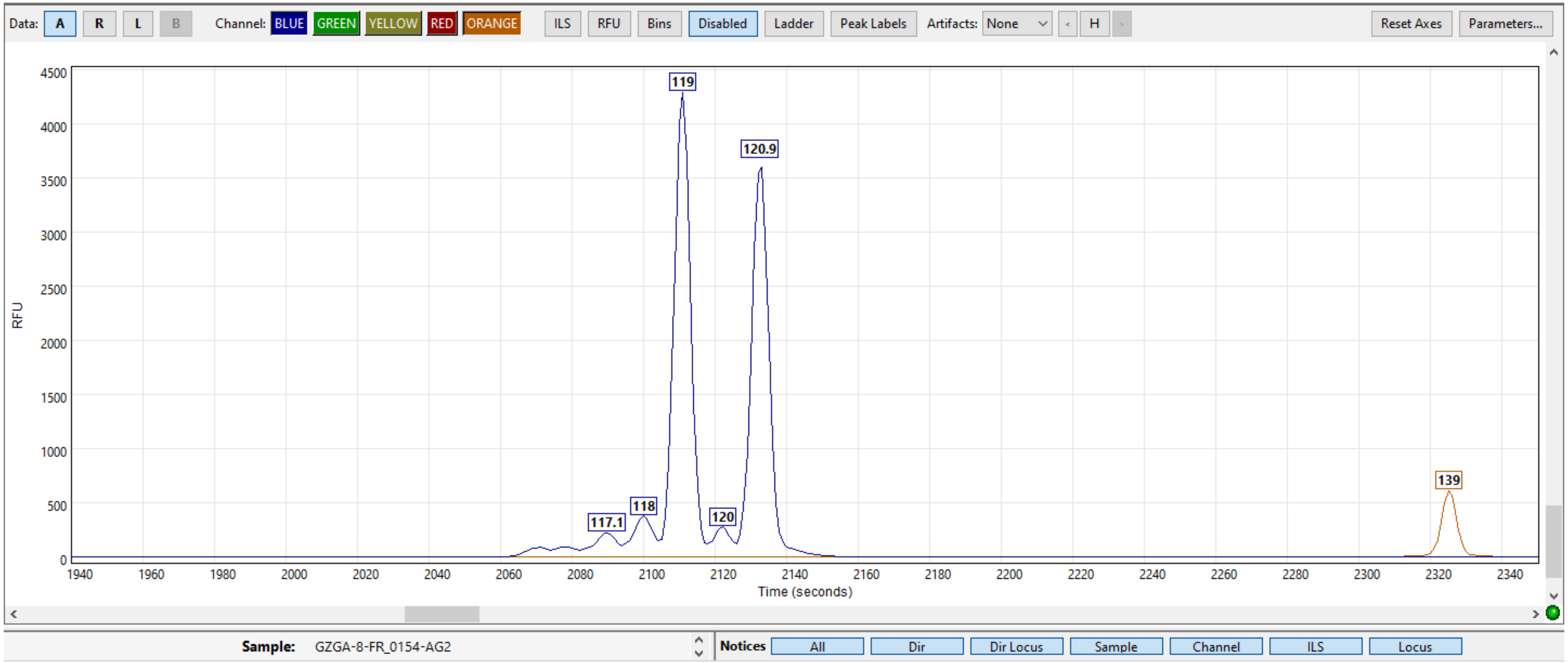


| | |
|---|---|
| **Observer 1** | **119;121** |
| **Observer 2** | **119;120.9** |
| **Observer 3** | **119;120.9** |

## 27- Wild. Locus AG2 sample GZGA9 (0155)

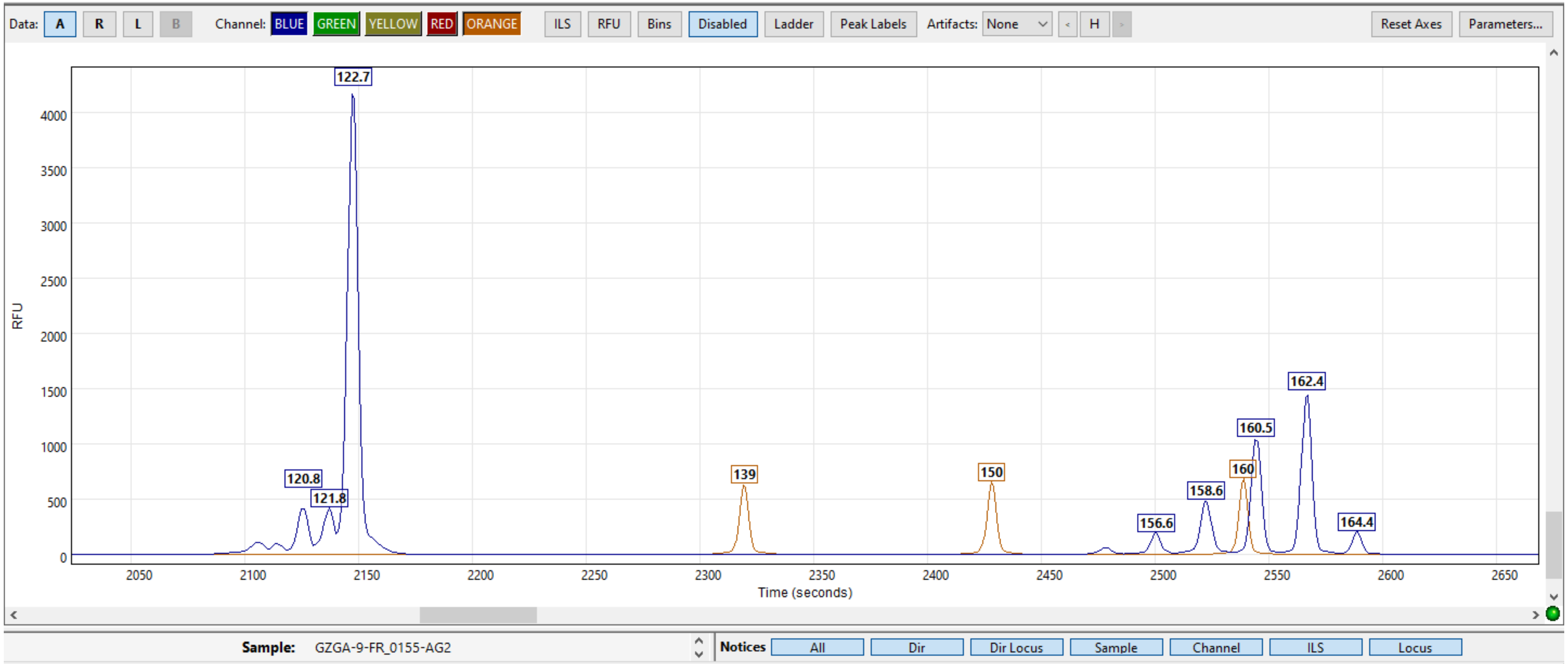


| | |
|---|---|
| **Observer 1** | **123;162** |
| **Observer 2** | **122.7;162.4** |
| **Observer 3** | **122.7;162.4** |

**28- Wild. Locus AG2 sample MACC3 (0156)**

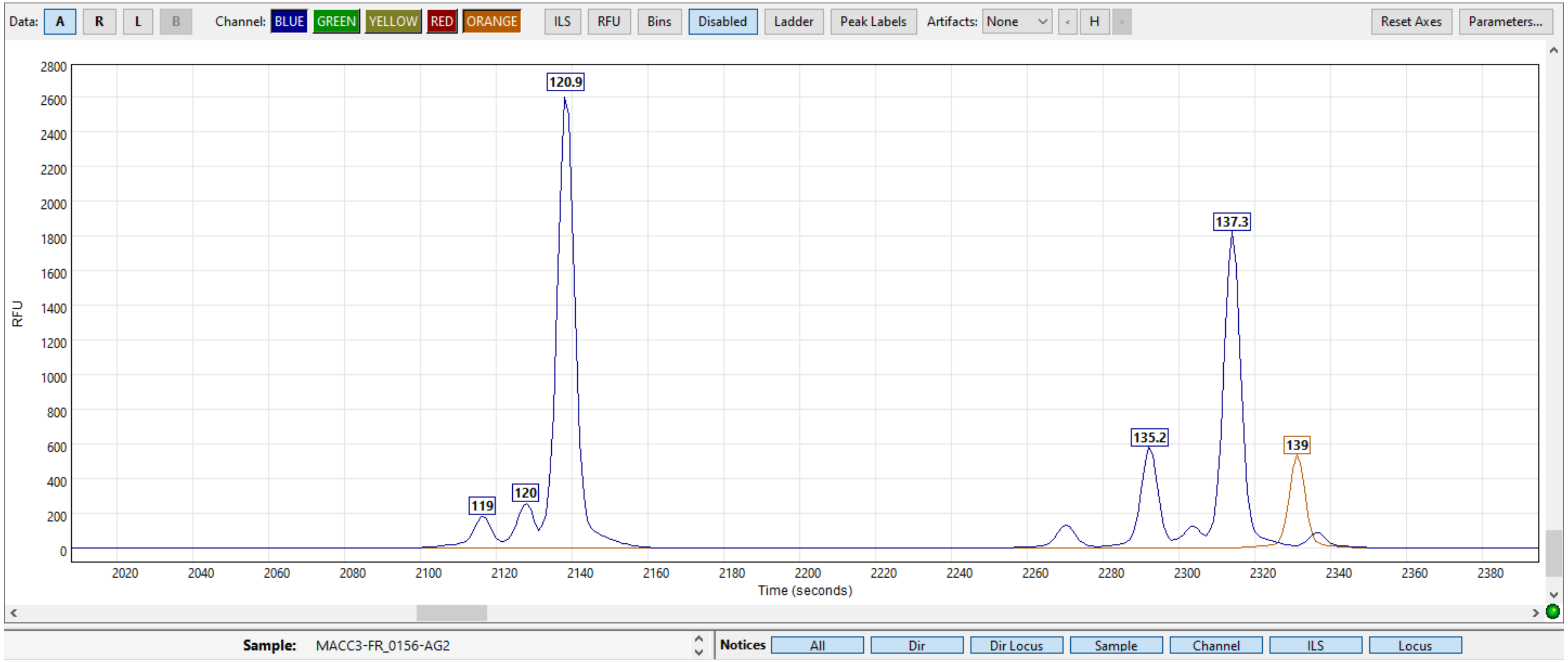


| | |
|---|---|
| **Observer 1** | **121;137** |
| **Observer 2** | **120.9;137.3** |
| **Observer 3** | **121;137** |

**29- Wild. Locus AG2 sample PONT92 (0157)**

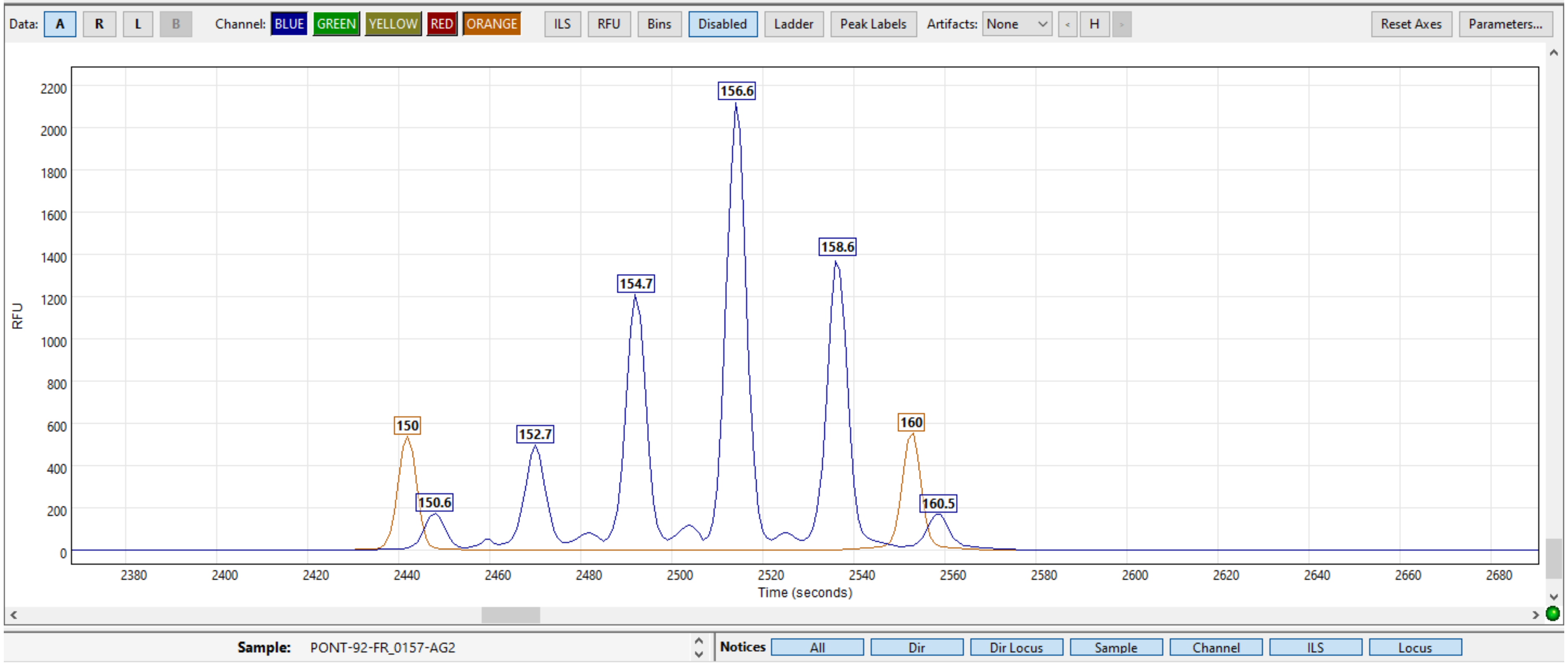


| | |
|---|---|
| **Observer 1** | **157** |
| **Observer 2** | **156.6** |
| **Observer 3** | **156.6** |

### 30- Wild. Locus AG2 sample PONT95 (0158)

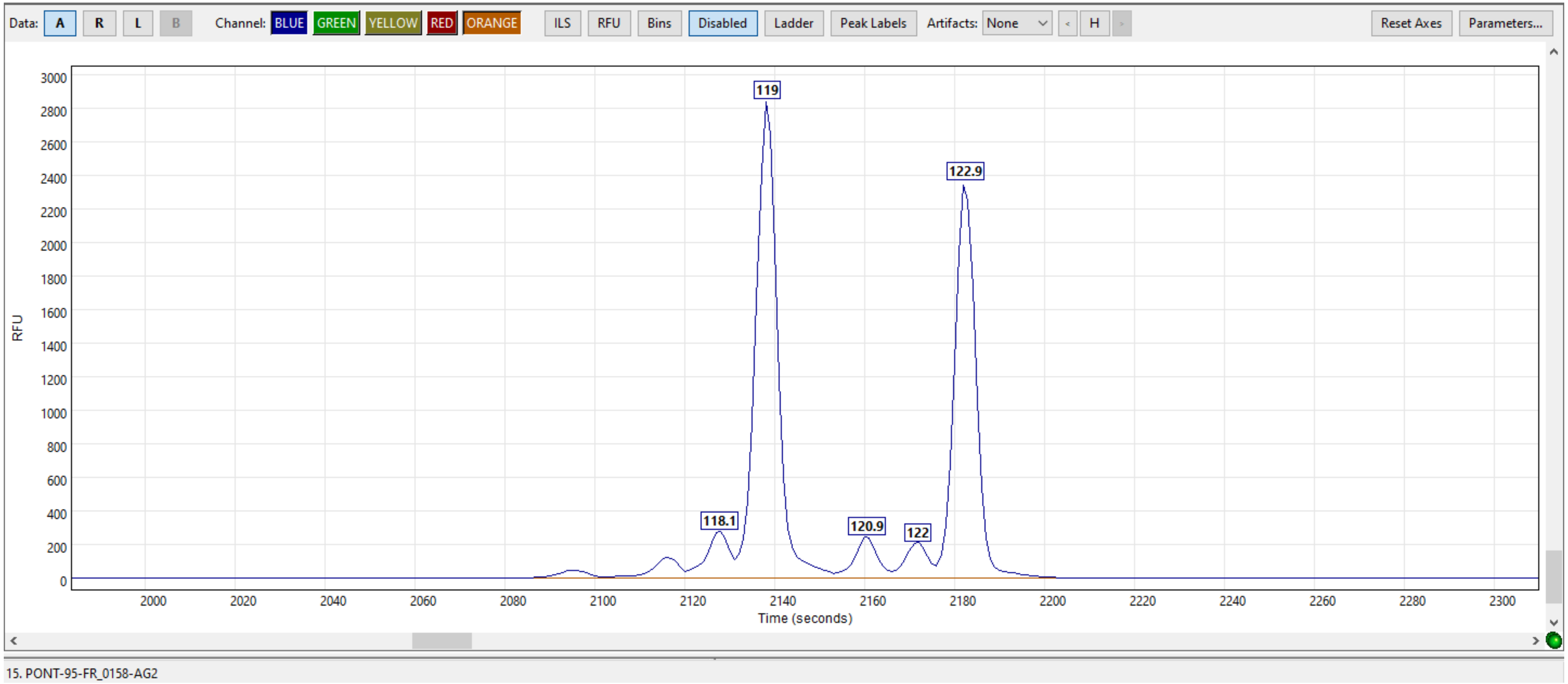


| | |
|---|---|
| **Observer 1** | **119;123** |
| **Observer 2** | **119;122.9** |
| **Observer 3** | **119;123** |

## AG4

### 1- Wild. Locus AG4 sample 01 (0909)

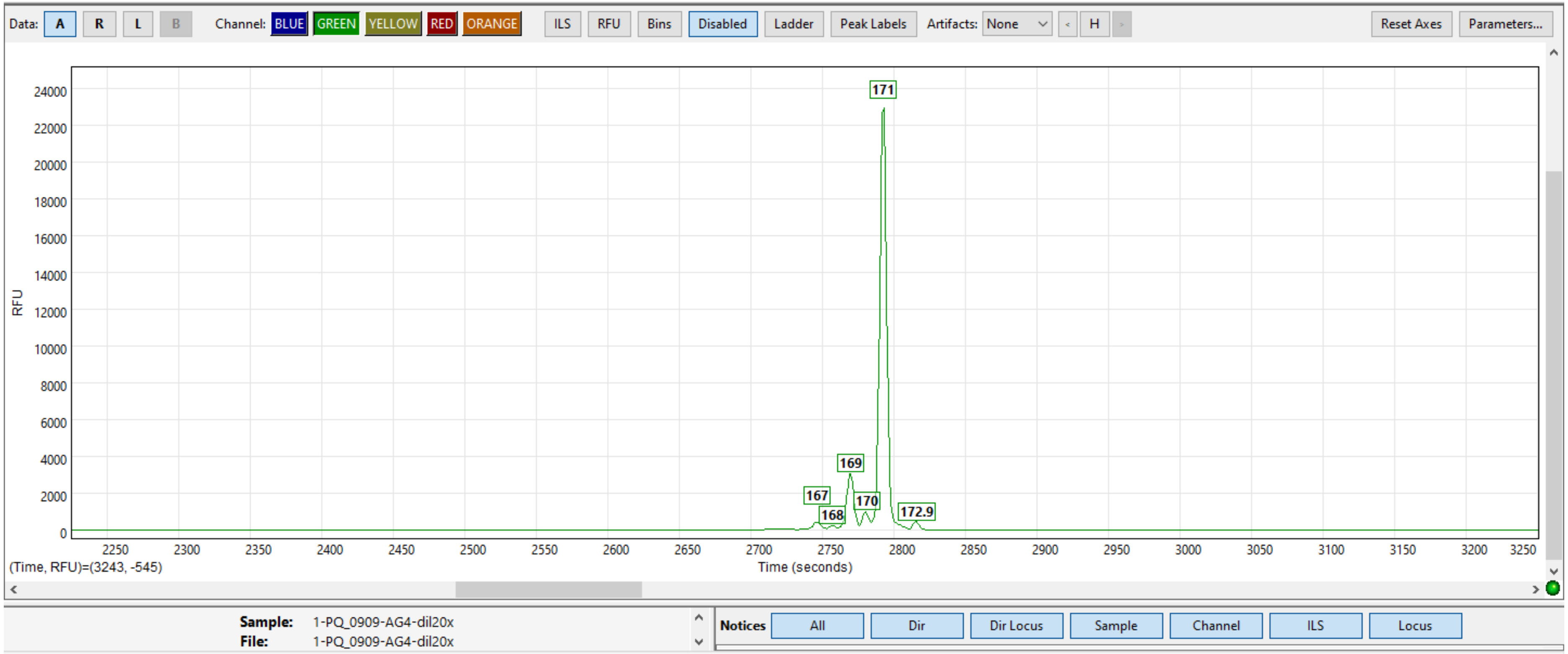


| | |
|---|---|
| **Observer 1** | **171** |
| **Observer 2** | **171** |
| **Observer 3** | **171** |

## 2- Wild. Locus AG4 sample 03 (0910)

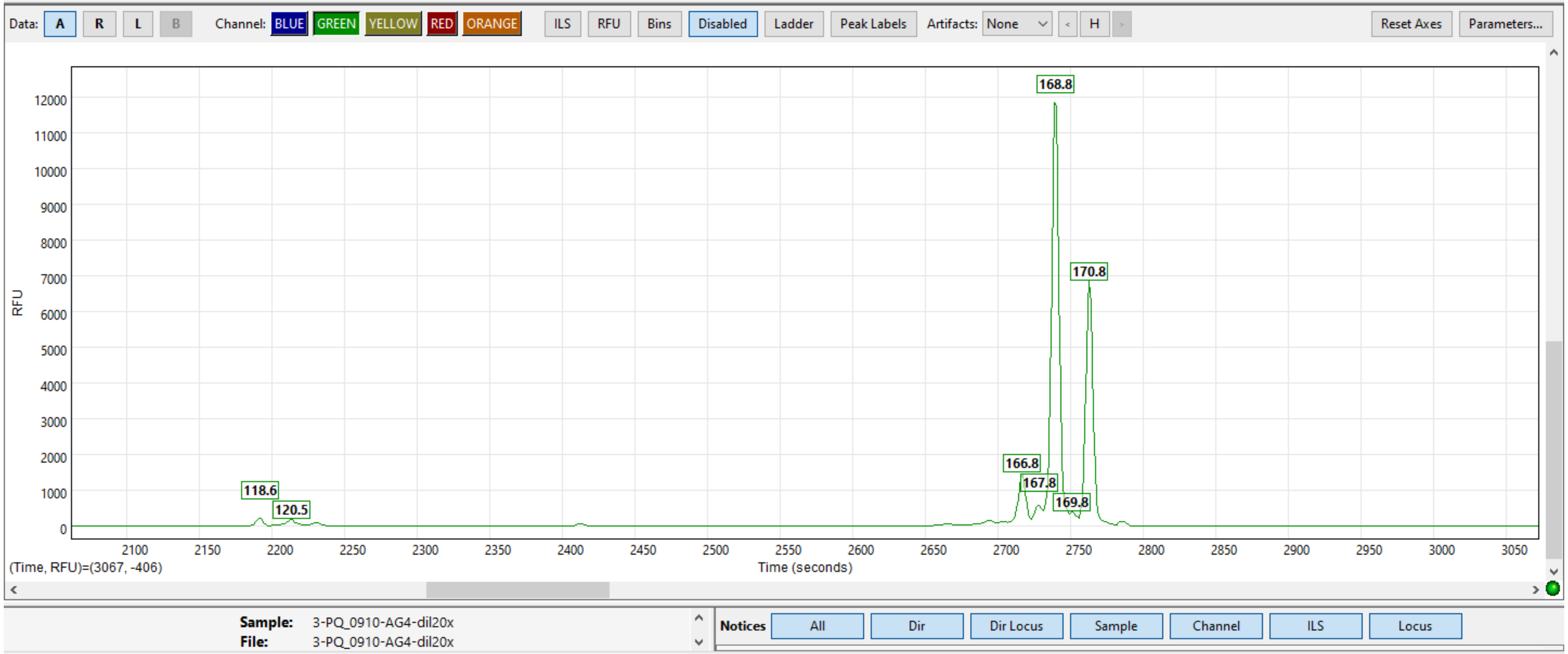


| | |
|---|---|
| **Observer 1** | **169;171** |
| **Observer 2** | **168.8;170.8** |
| **Observer 3** | **169;171** |

**3- Wild. Locus AG4 sample 05 (0911)**

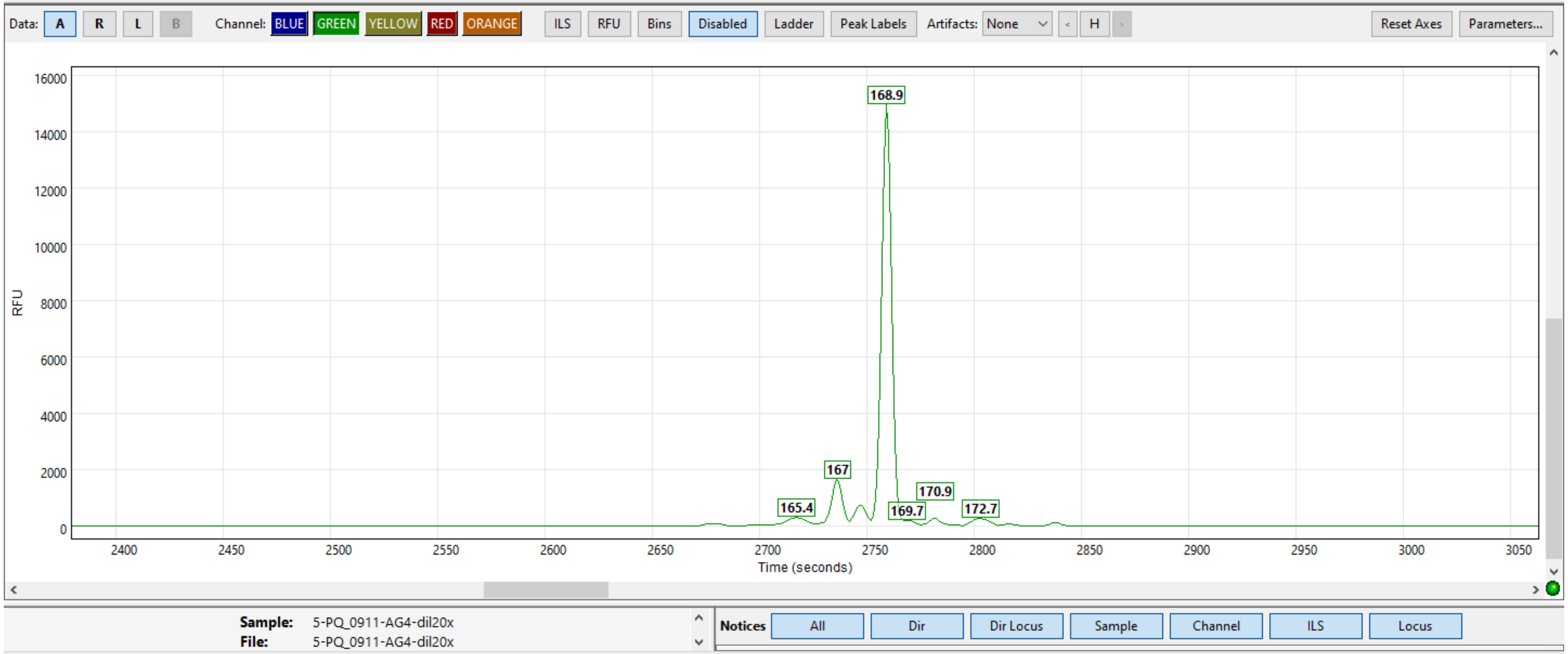


| | |
|---|---|
| **Observer 1** | **169** |
| **Observer 2** | **168.9** |
| **Observer 3** | **169** |

**4- Wild. Locus AG4 sample 07 (0912)**

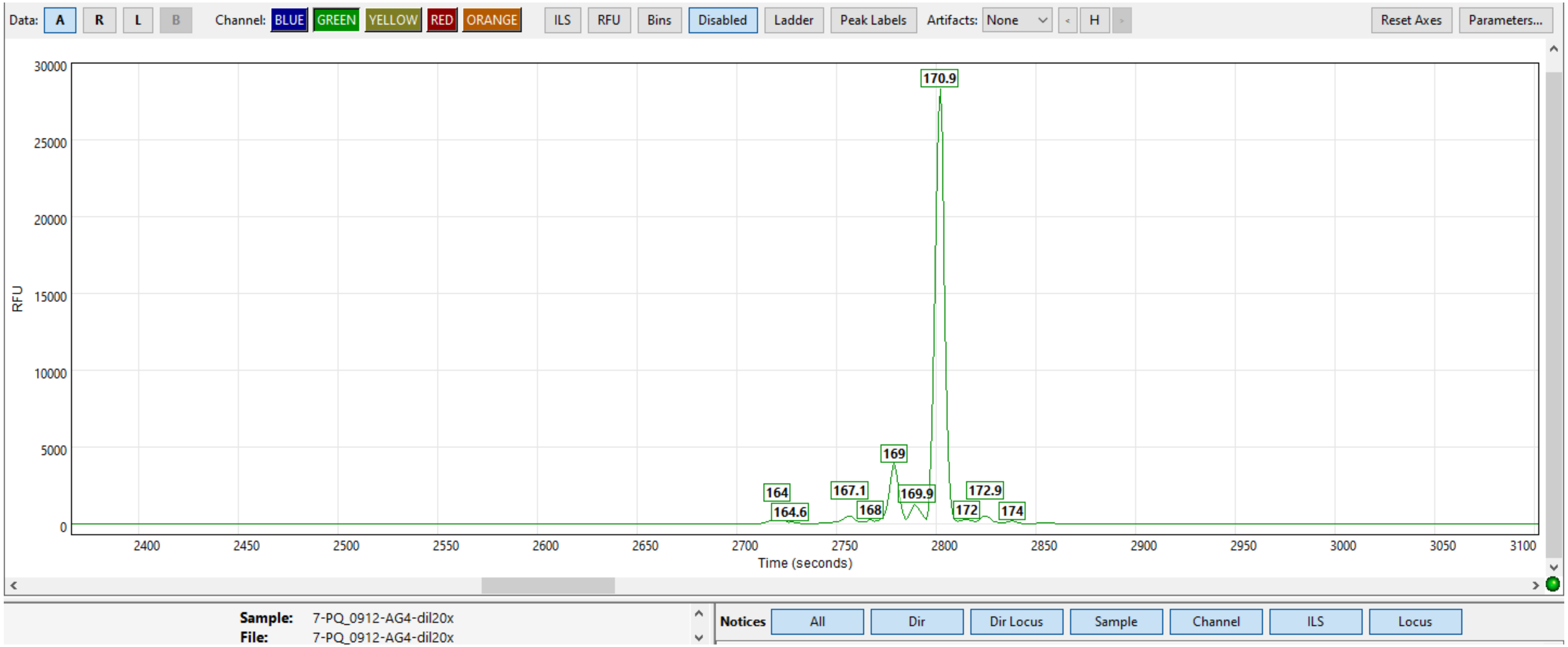


| | |
|---|---|
| **Observer 1** | **171** |
| **Observer 2** | **170.9** |
| **Observer 3** | **171** |

## 5- Wild. Locus AG4 sample 08 (0913)

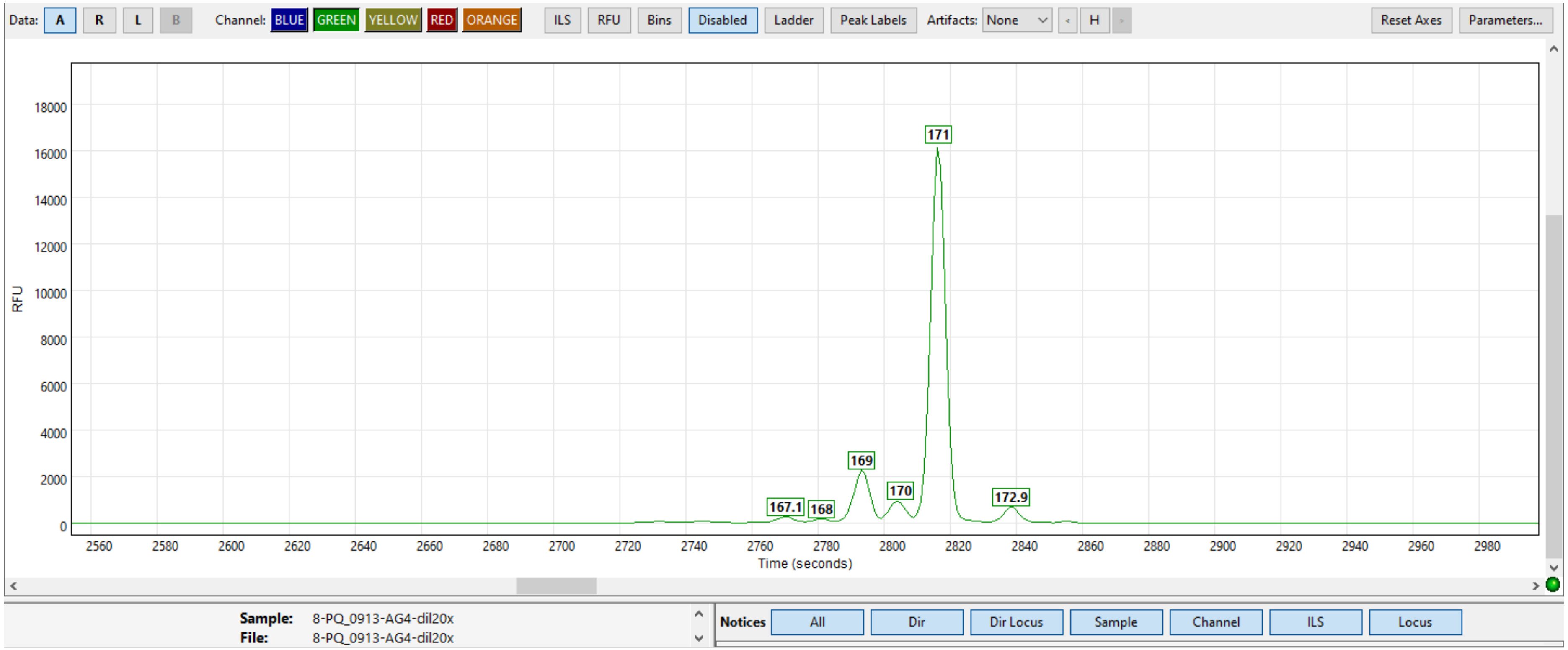


| | |
|---|---|
| **Observer 1** | **171** |
| **Observer 2** | **171** |
| **Observer 3** | **171** |

### 6- Wild. Locus AG4 sample 09 (0914)

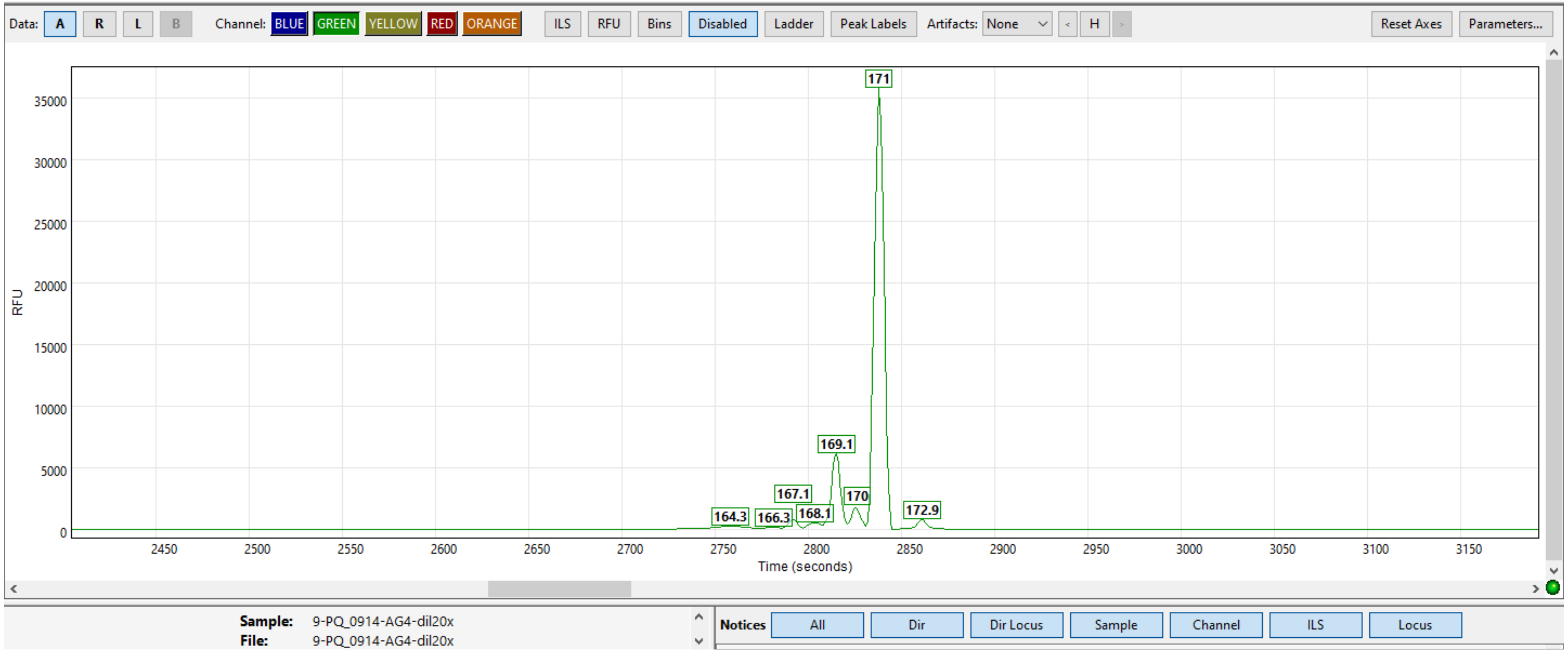


| | |
|---|---|
| **Observer 1** | **171** |
| **Observer 2** | **171** |
| **Observer 3** | **171** |

**7- Wild. Locus AG4 sample 12 (0915)**

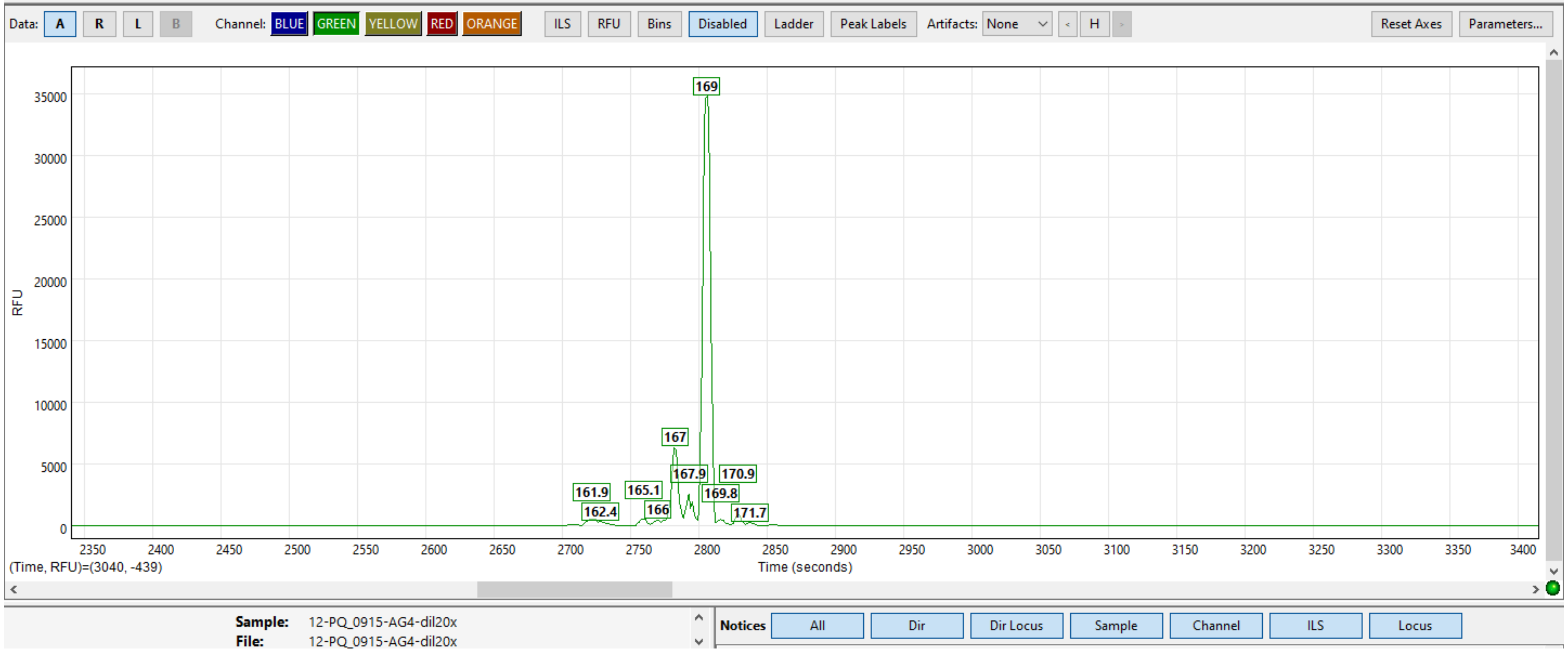


| | |
|---|---|
| **Observer 1** | **169** |
| **Observer 2** | **169** |
| **Observer 3** | **169** |

## 8- Wild. Locus AG4 sample 13 (0916)

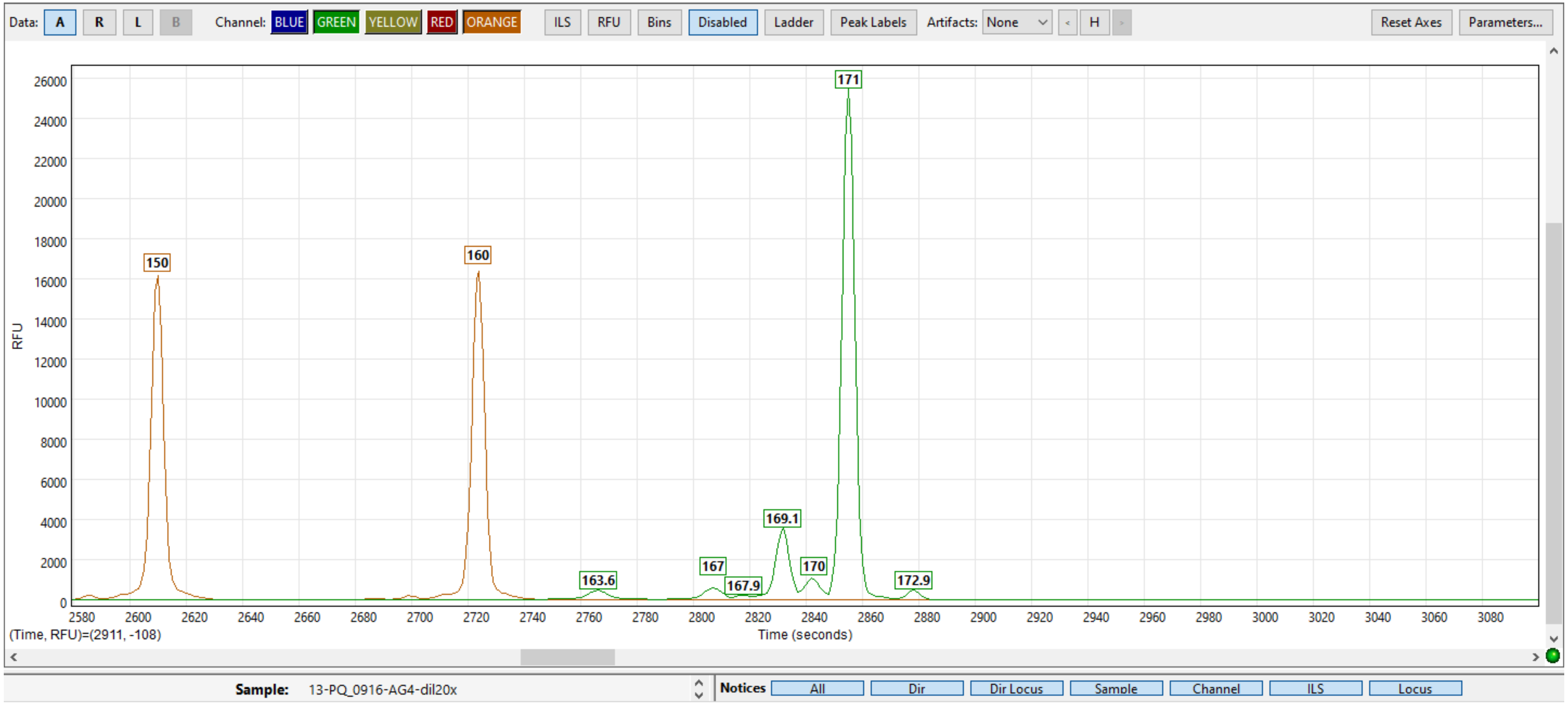


| | |
|---|---|
| **Observer 1** | **171** |
| **Observer 2** | **171** |
| **Observer 3** | **171** |

**9- Wild. Locus AG4 sample 14 (0917)**

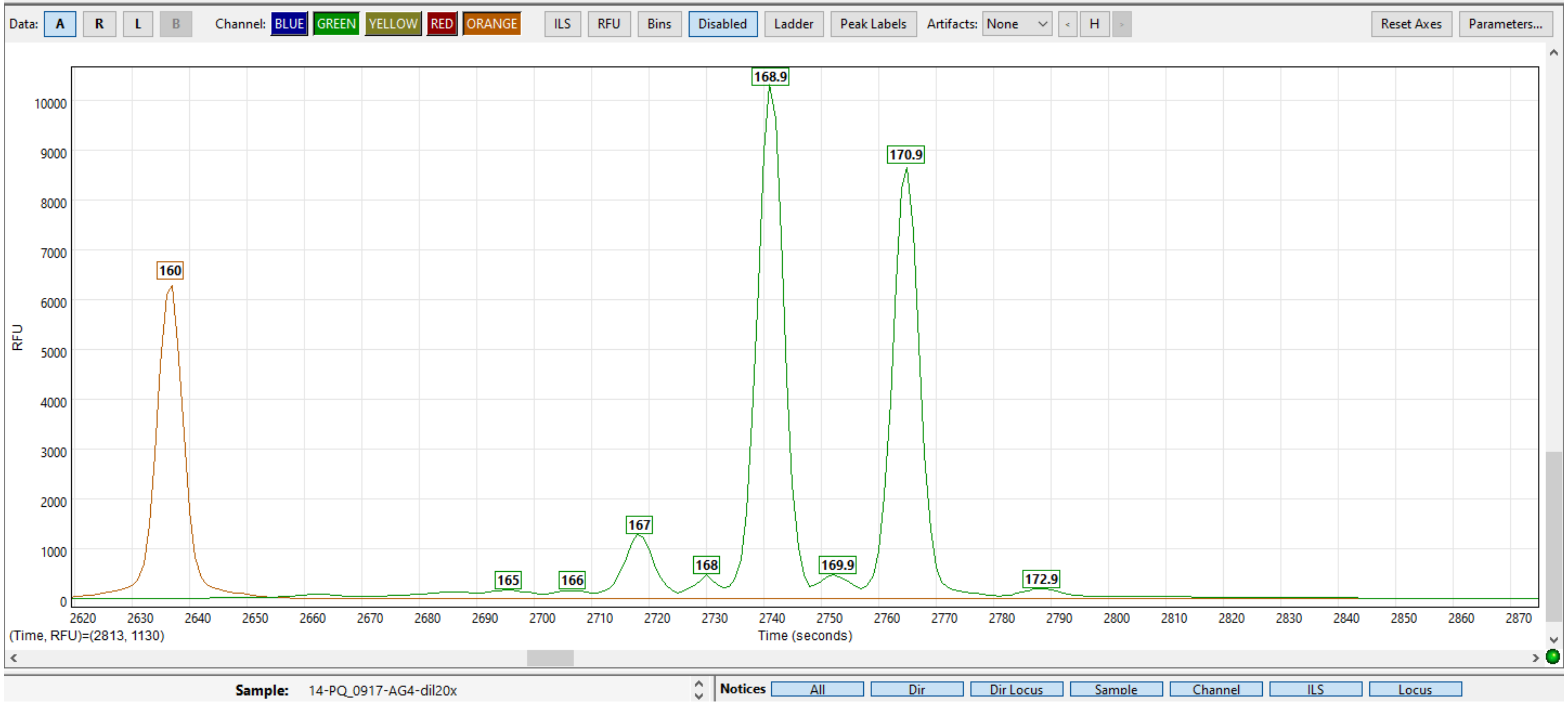


| | |
|---|---|
| **Observer 1** | **169;171** |
| **Observer 2** | **168.9;170.9** |
| **Observer 3** | **169;171** |

**10- Wild. Locus AG4 sample 15 (0918)**

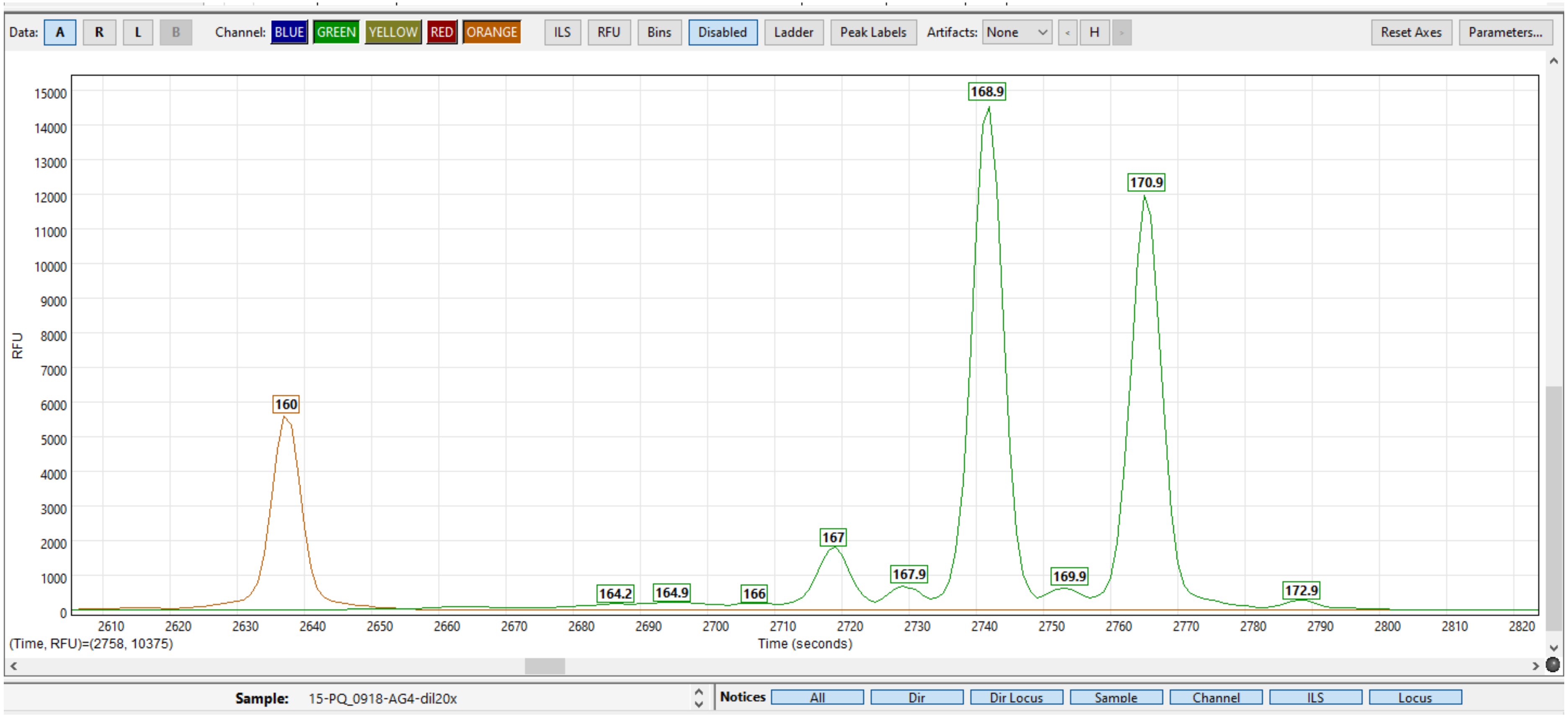


| | |
|---|---|
| **Observer 1** | **169;171** |
| **Observer 2** | **168.9;170.9** |
| **Observer 3** | **169;171** |

### 11- Wild. Locus AG4 sample 16 (0919)

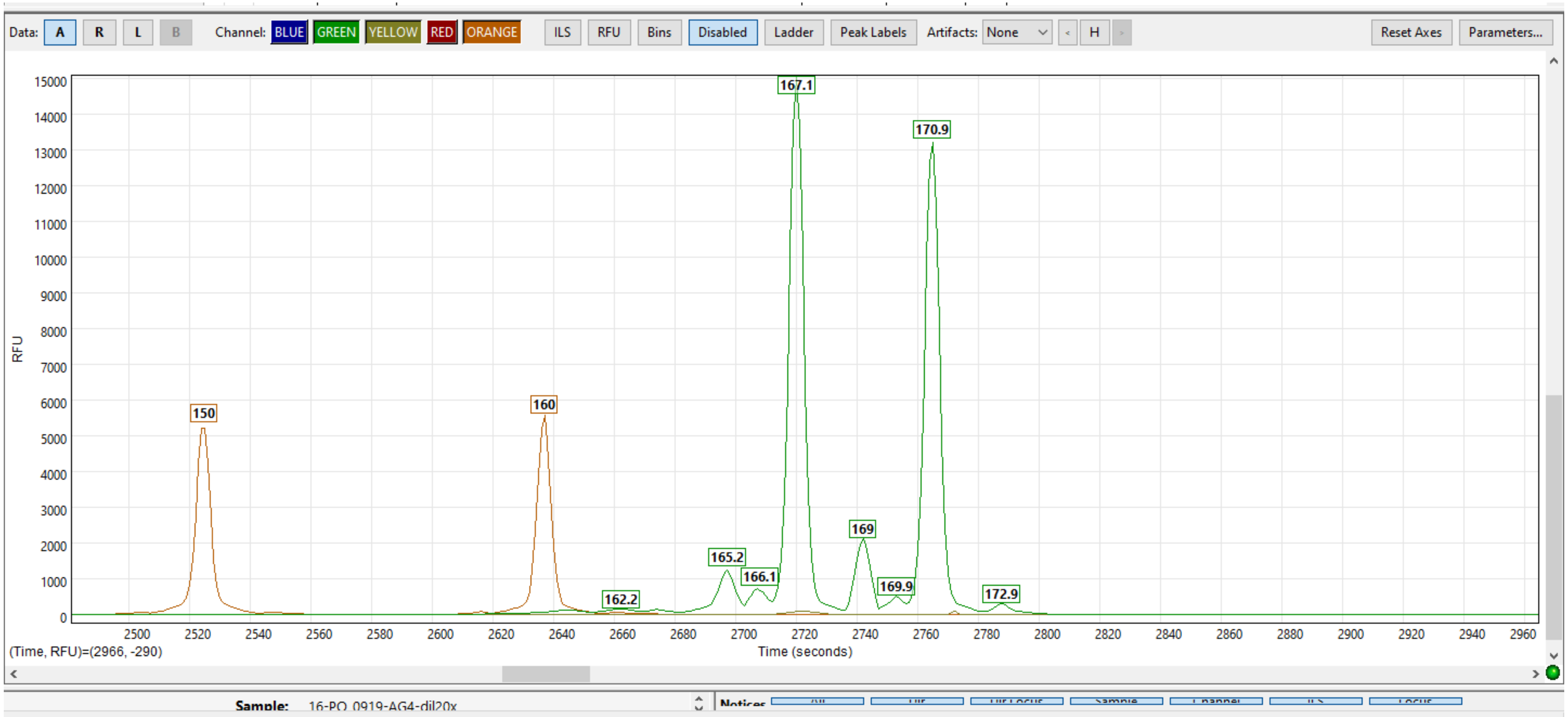


| | |
|---|---|
| **Observer 1** | **167;171** |
| **Observer 2** | **167.1;170.9** |
| **Observer 3** | **167;171** |

**12- Wild. Locus AG4 sample 17 (0920)**

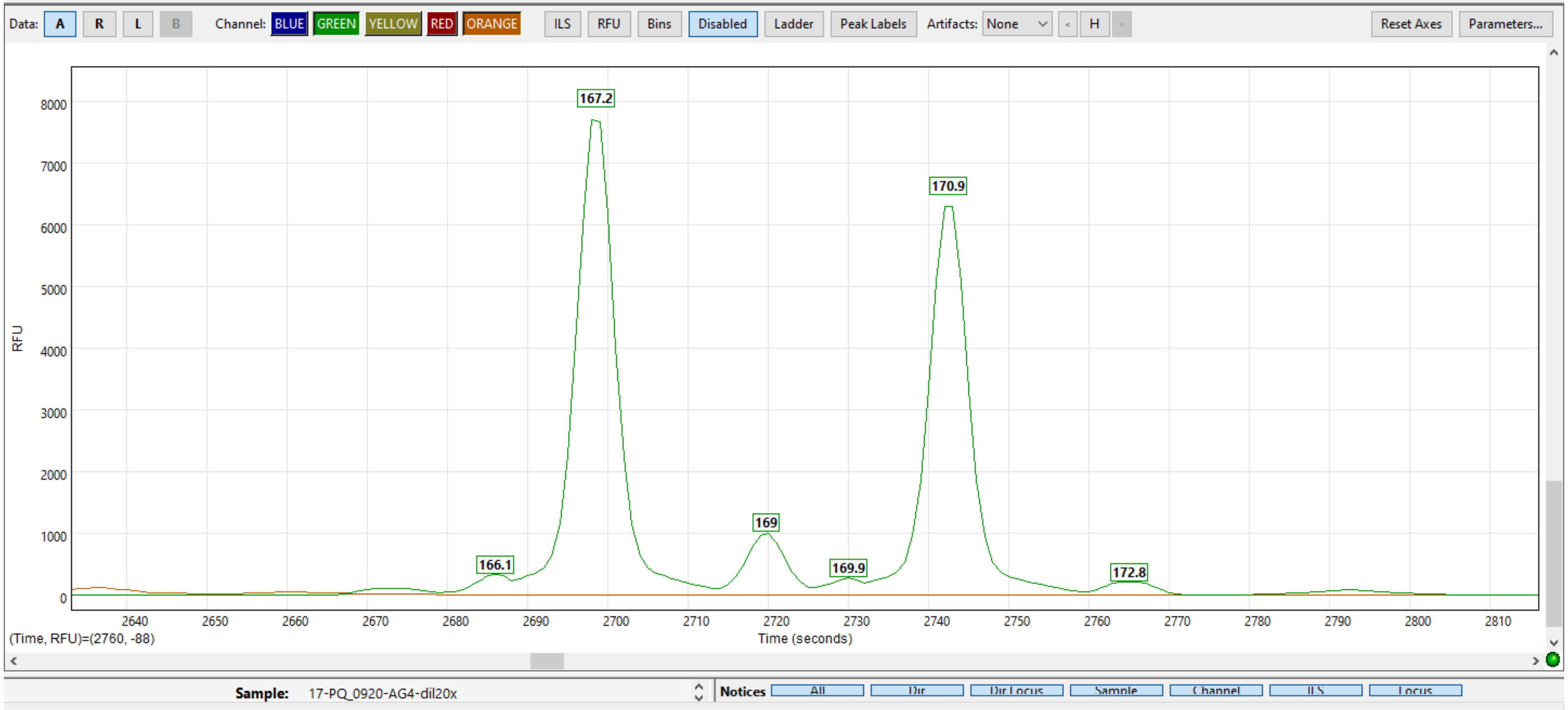


| | |
|---|---|
| **Observer 1** | **167;171** |
| **Observer 2** | **167.2;170.9** |
| **Observer 3** | **167;171** |

### 13- Wild. Locus AG4 sample 19 (0921)

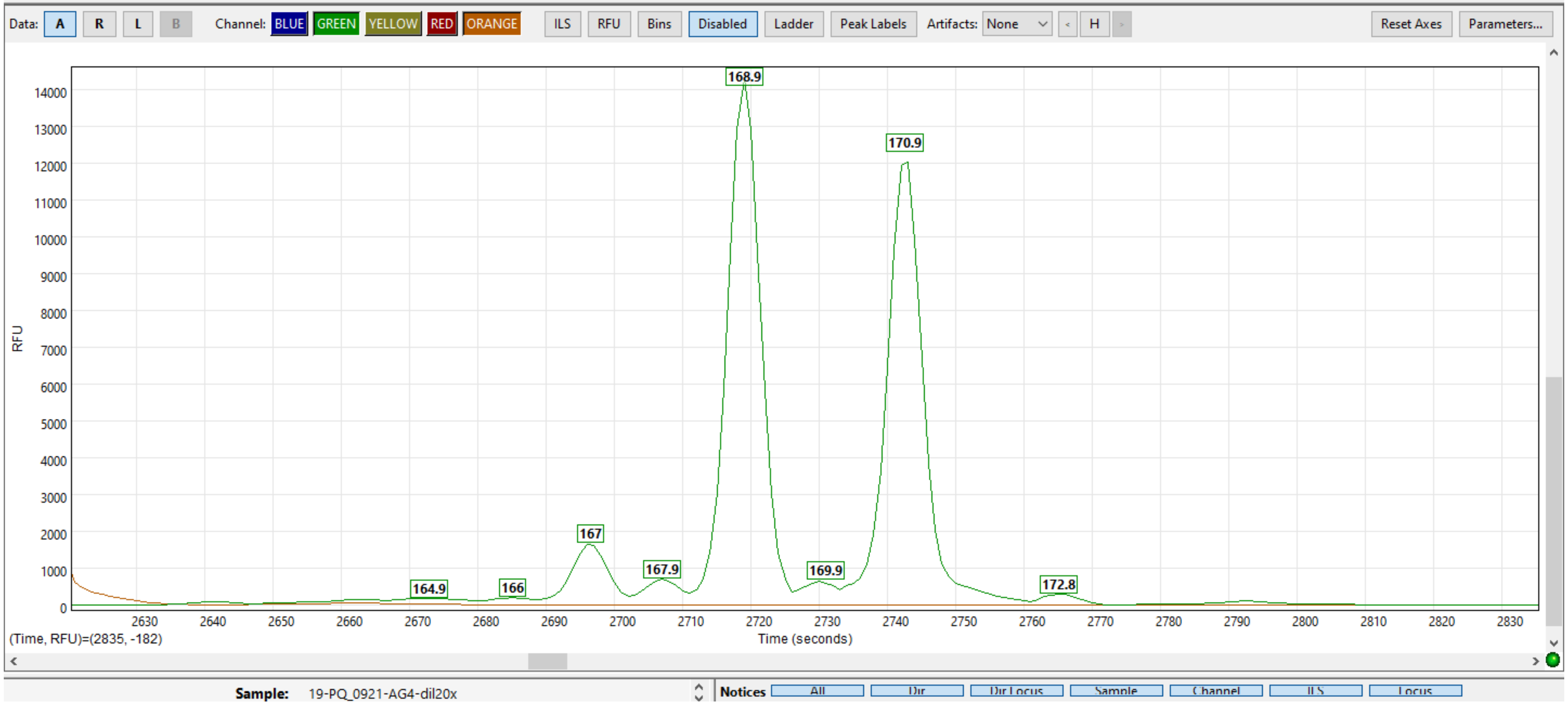


| | |
|---|---|
| **Observer 1** | **169;171** |
| **Observer 2** | **168.9;170.9** |
| **Observer 3** | **169;171** |

**14- Wild. Locus AG4 sample 20 (0922)**

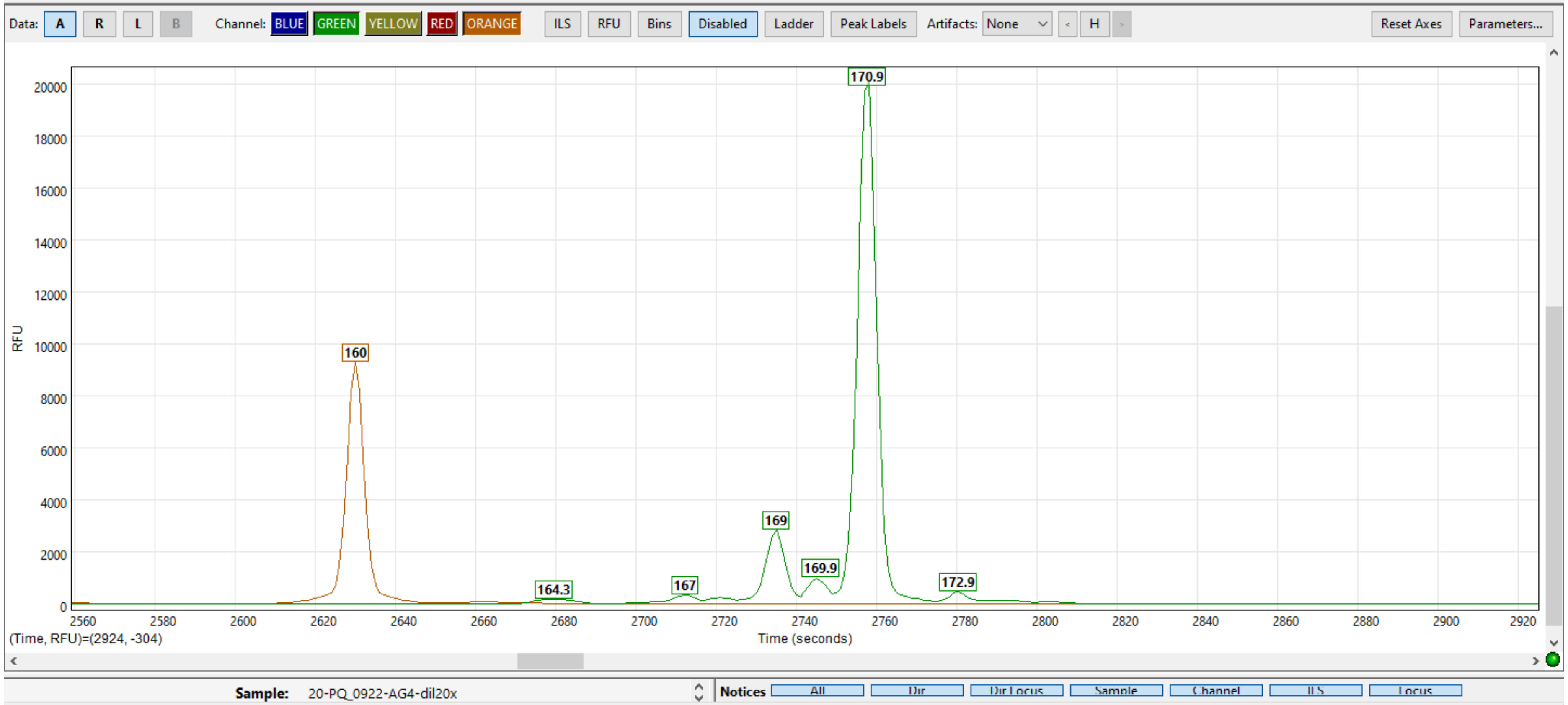


| | |
|---|---|
| **Observer 1** | **169;171** |
| **Observer 2** | **170.9** |
| **Observer 3** | **171** |

## 15- Wild. Locus AG4 sample 21 (0924)

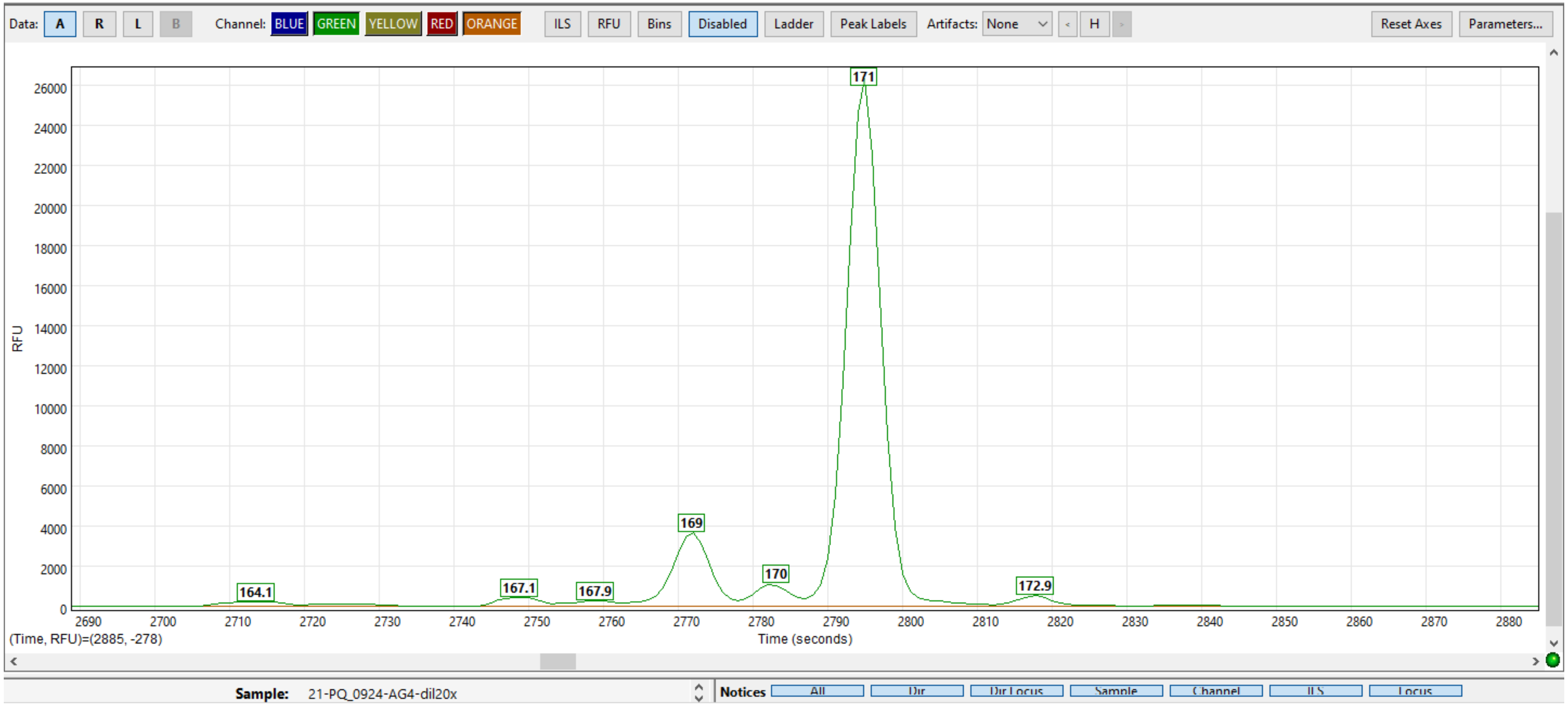


| | |
|---|---|
| **Observer 1** | **171** |
| **Observer 2** | **171** |
| **Observer 3** | **171** |

**16- Wild. Locus AG4 sample CAST2 (0144)**

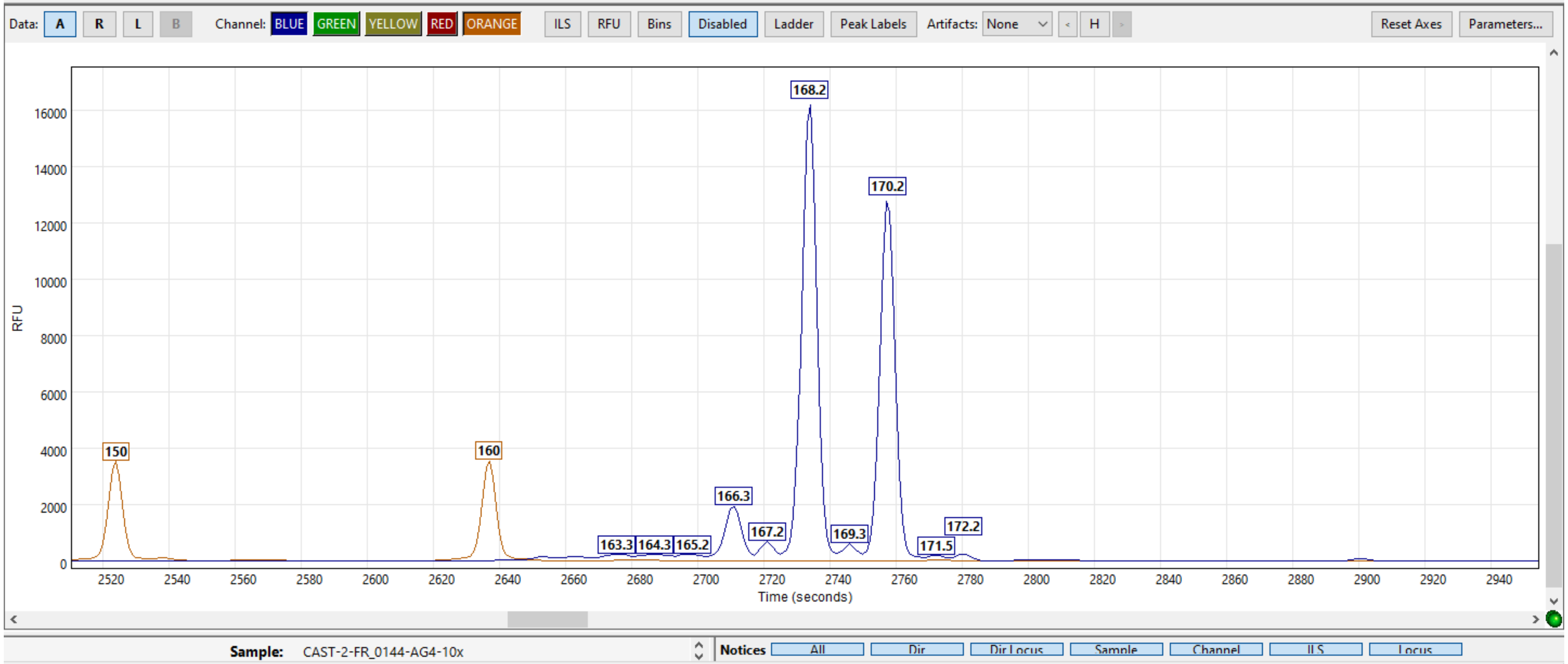


| | |
|---|---|
| **Observer 1** | **168;170** |
| **Observer 2** | **168.2;170.2** |
| **Observer 3** | **168;170** |

**17- Wild. Locus AG4 sample CAST3 (0145)**

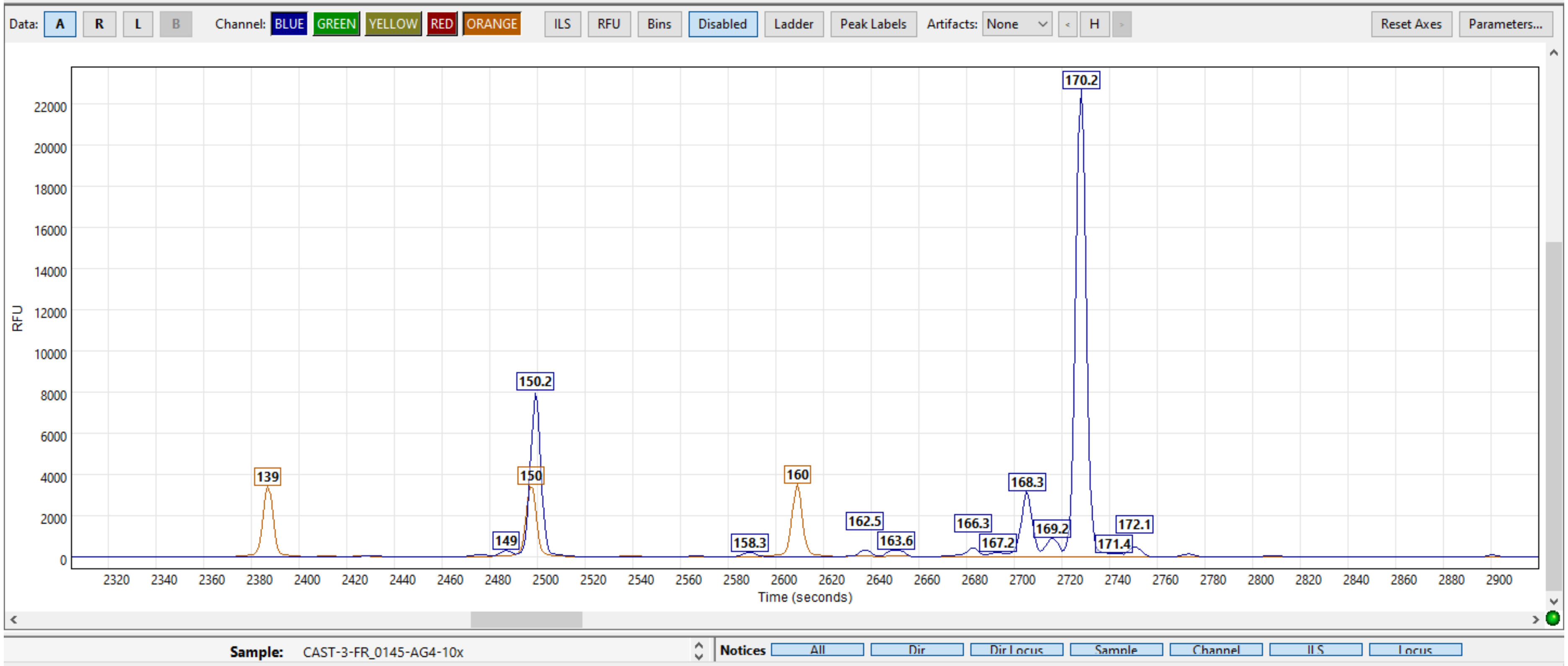


| | |
|---|---|
| **Observer 1** | **150;170** |
| **Observer 2** | **150.2;170.2** |
| **Observer 3** | **150;170** |

**18- Wild. Locus AG4 sample CENT146 (0146)**

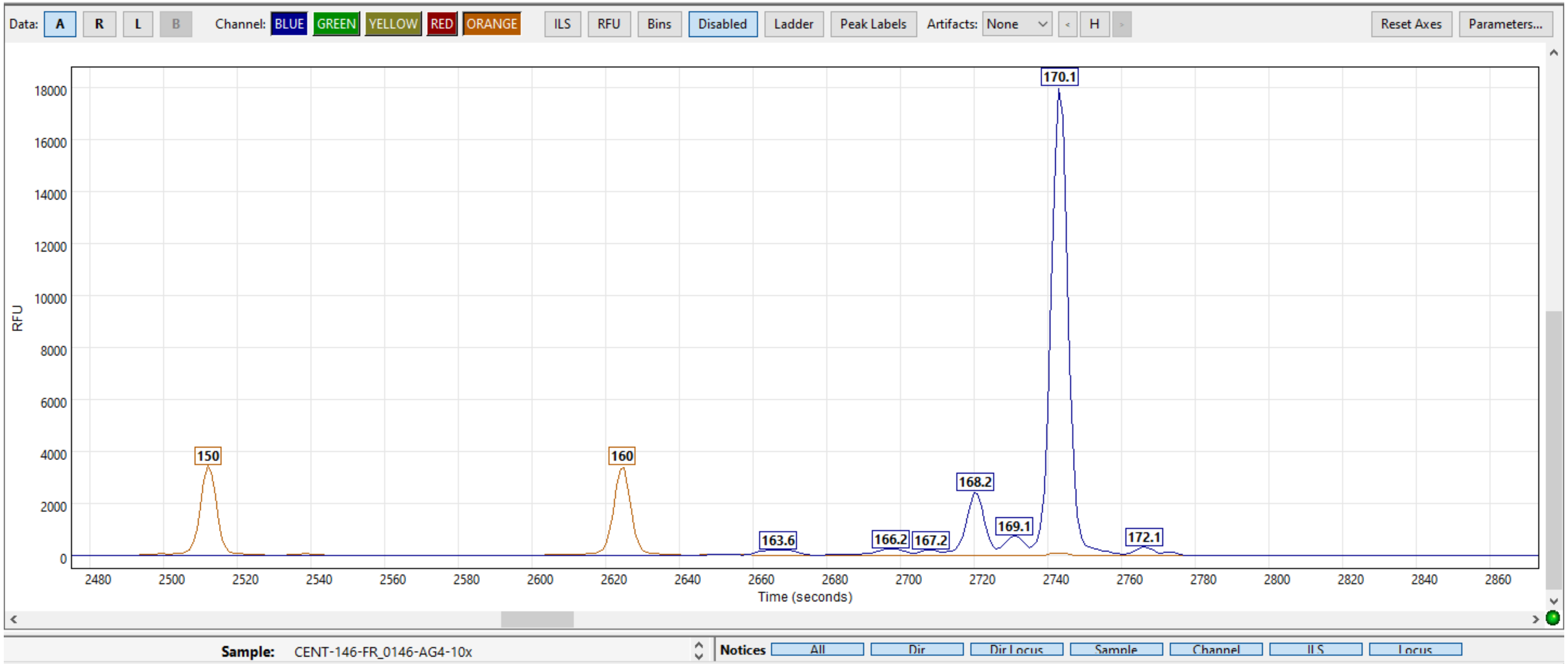


| | |
|---|---|
| **Observer 1** | **168;170** |
| **Observer 2** | **170.1** |
| **Observer 3** | **170.1** |

## 19- Wild. Locus AG4 sample CENT151 (0147)

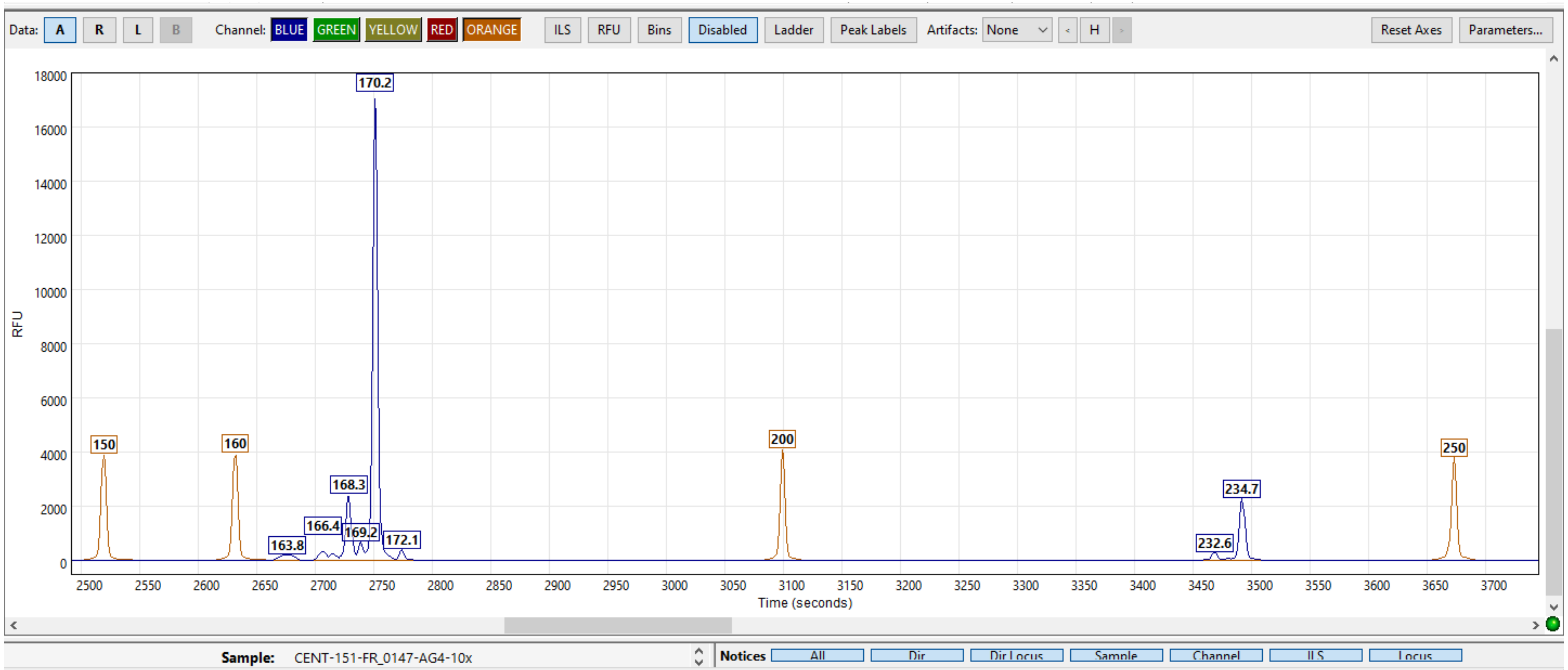


| | |
|---|---|
| **Observer 1** | **170;235** |
| **Observer 2** | **170.2** |
| **Observer 3** | **170.2** |

## 20- Wild. Locus AG4 sample CENT152 (0148)

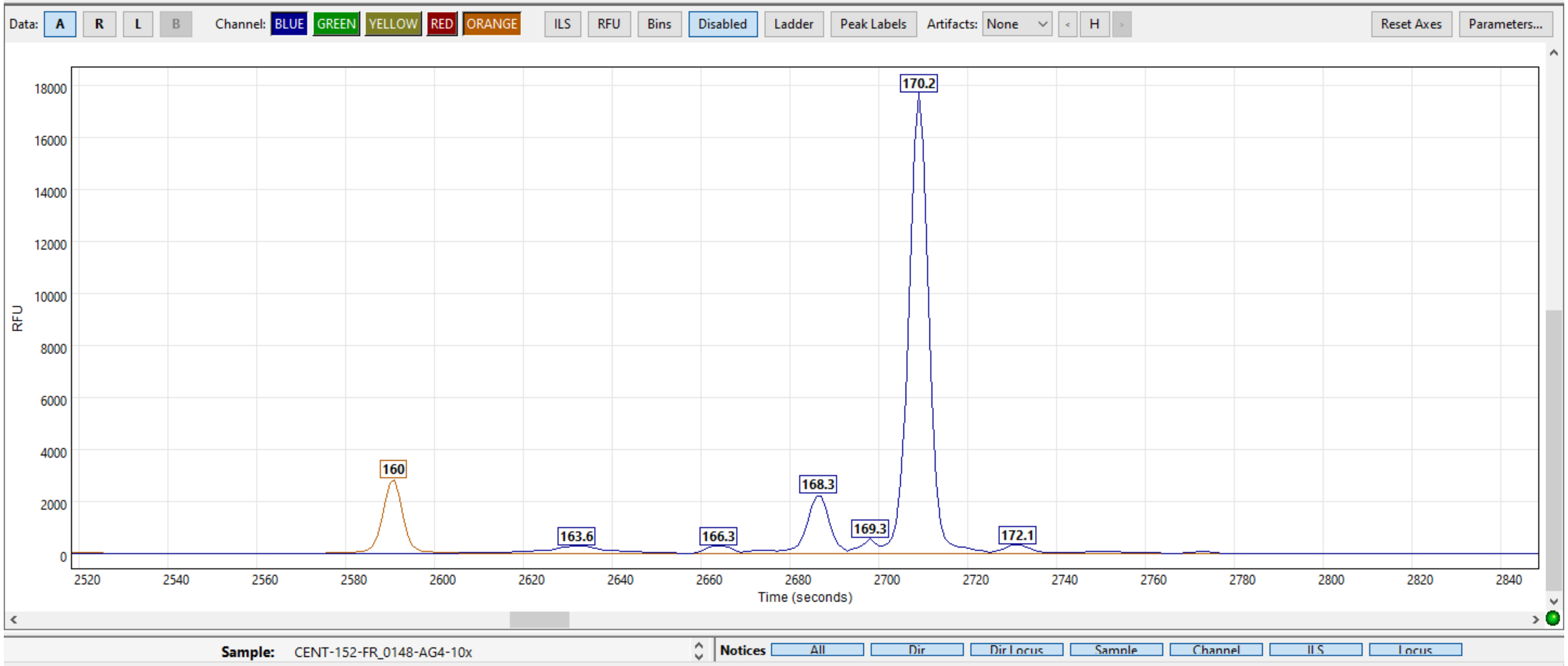


| | |
|---|---|
| **Observer 1** | **168;170** |
| **Observer 2** | **170.2** |
| **Observer 3** | **170.2** |

## 21- Wild. Locus AG4 sample CENT155 (0149)

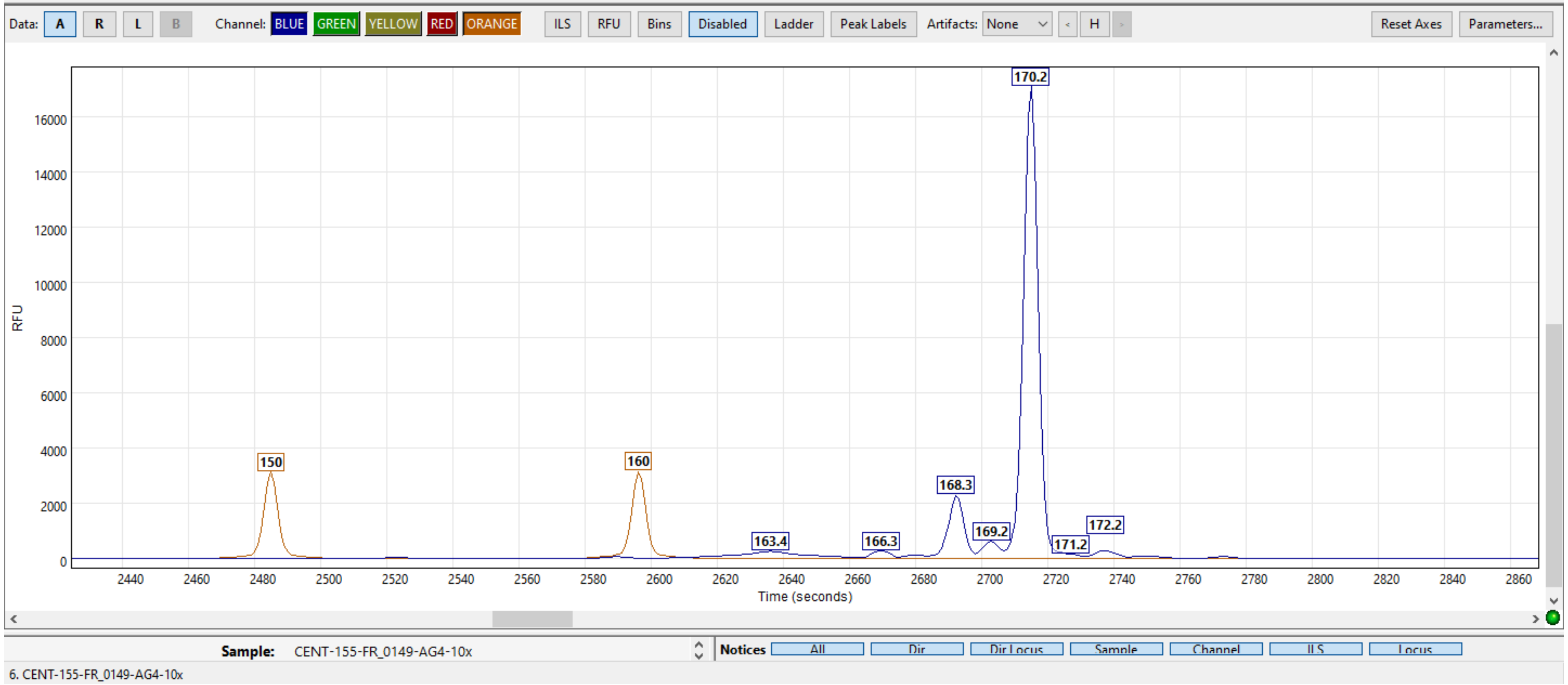


| | |
|---|---|
| **Observer 1** | **168;170** |
| **Observer 2** | **170.2** |
| **Observer 3** | **170.2** |

### 22- Wild. Locus AG4 sample CENT157 (0150)

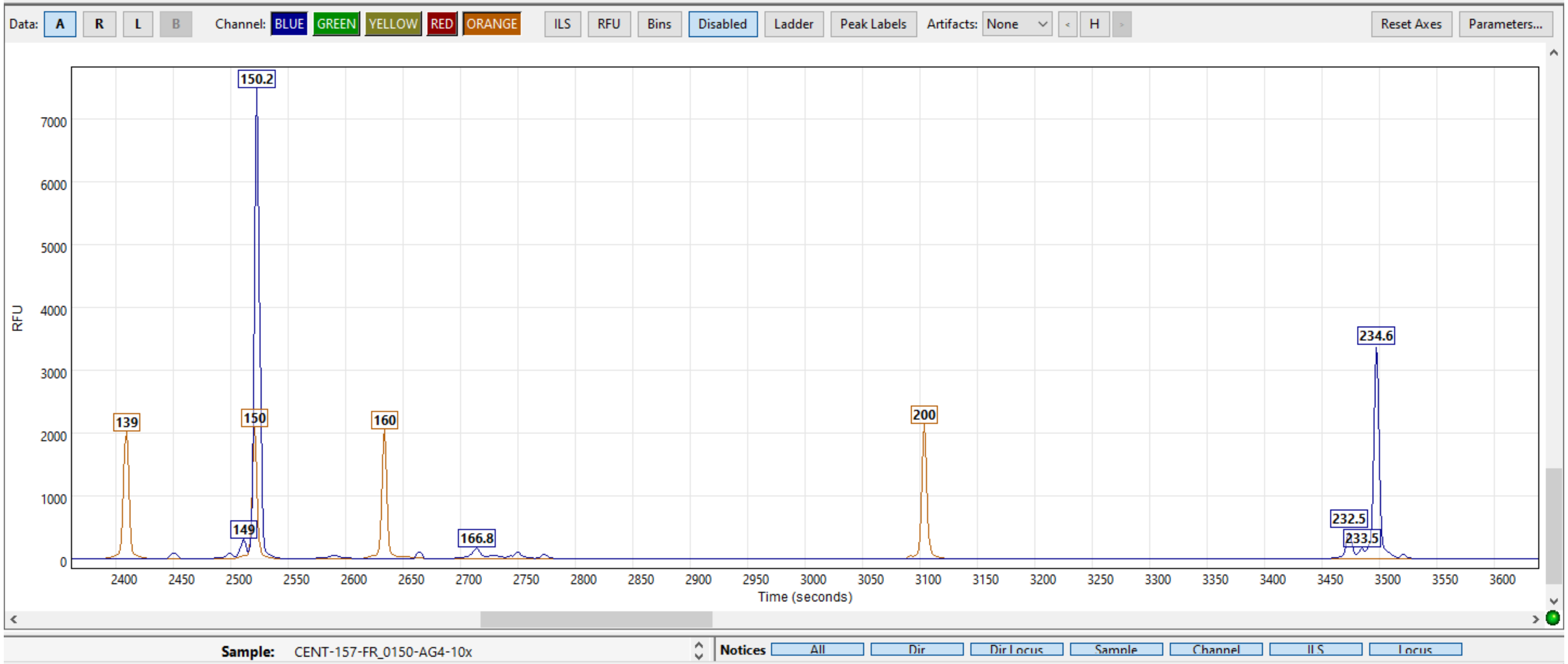


| | |
|---|---|
| **Observer 1** | **150;235** |
| **Observer 2** | **150.2;- 234.6** |
| **Observer 3** | **150;235** |

## 23- Wild. Locus AG4 sample CENT160 (0151)

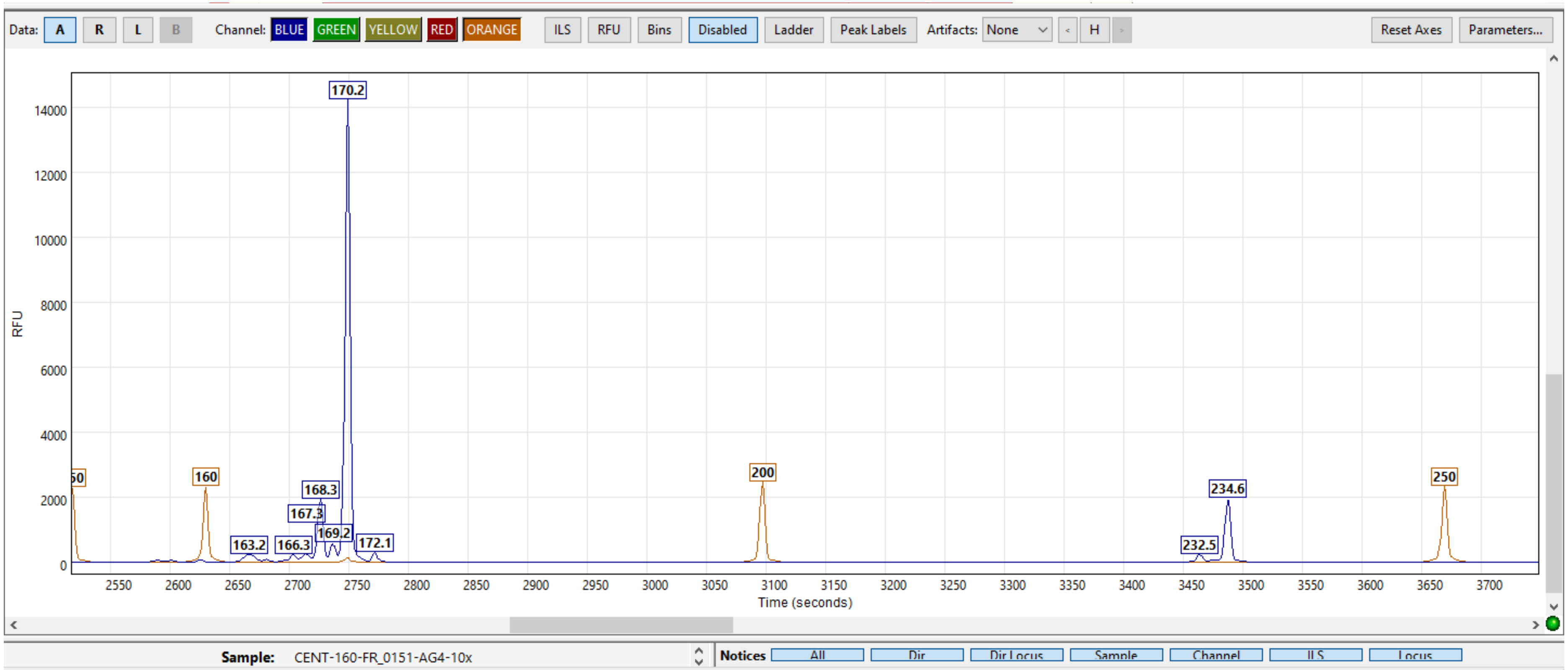


| | |
|---|---|
| **Observer 1** | **170;235** |
| **Observer 2** | **170.2 ; 234.6** |
| **Observer 3** | **170;235** |

**24- Wild. Locus AG4 sample CENT162 (0152)**

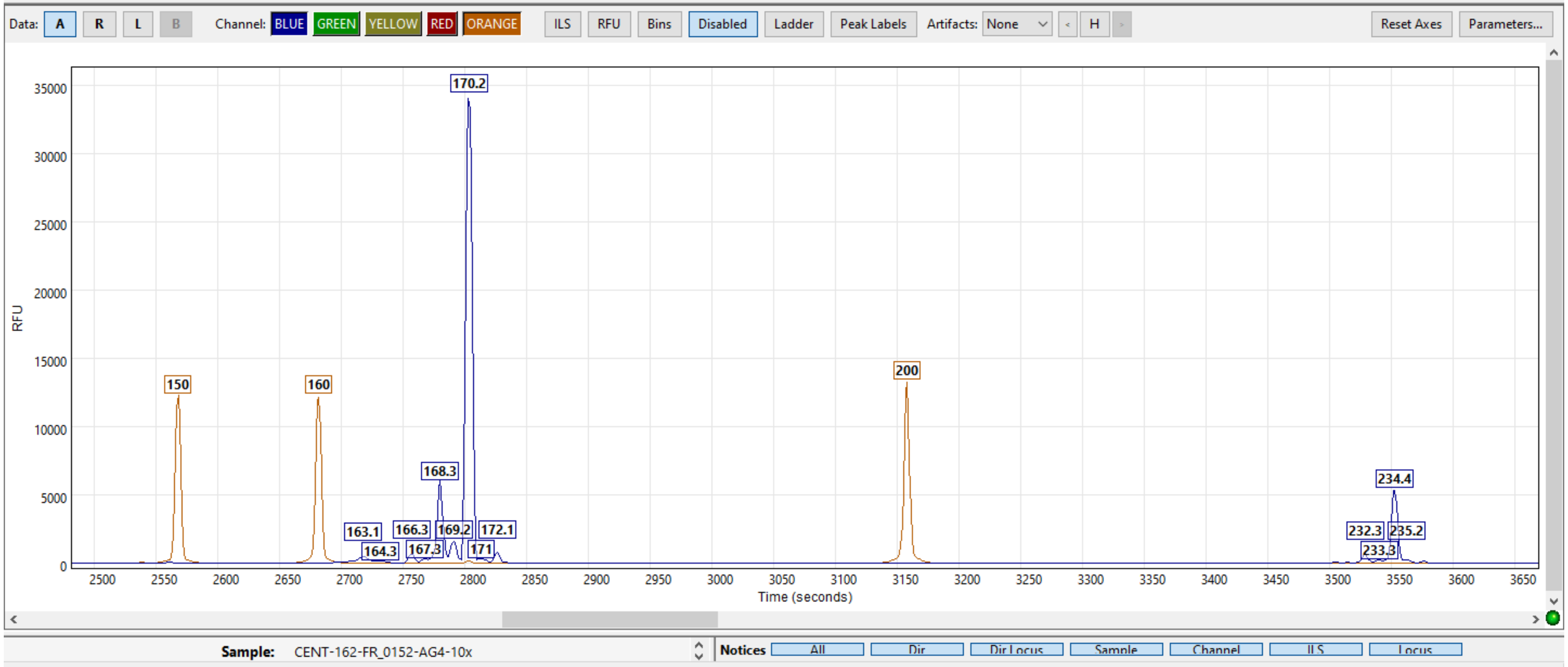


| | |
|---|---|
| **Observer 1** | **170;235** |
| **Observer 2** | **170.2; 234.4** |
| **Observer 3** | **170;235** |

## 25- Wild. Locus AG4 sample GZGA10 (0153)

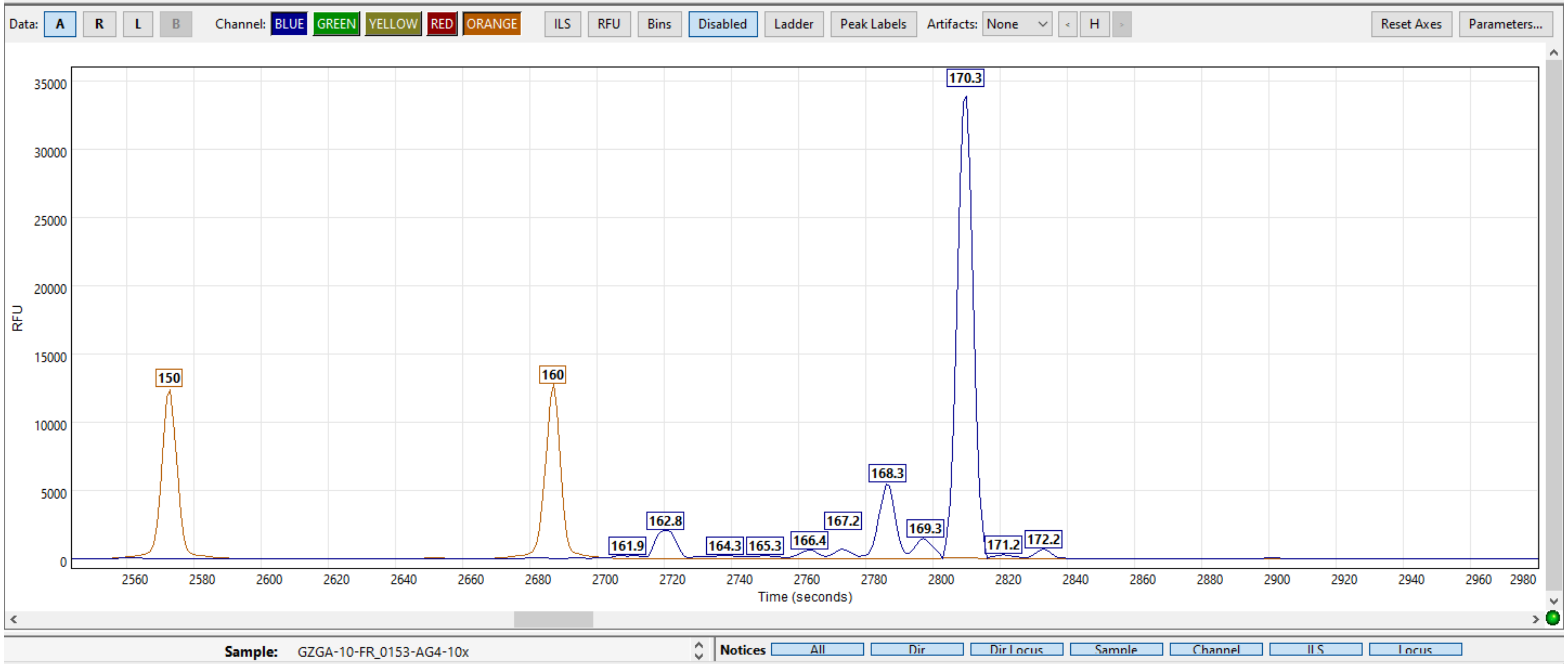


| | |
|---|---|
| **Observer 1** | **168;170** |
| **Observer 2** | **170.3** |
| **Observer 3** | **170.3** |

## 26- Wild. Locus AG4 sample GZGA8 (0154)

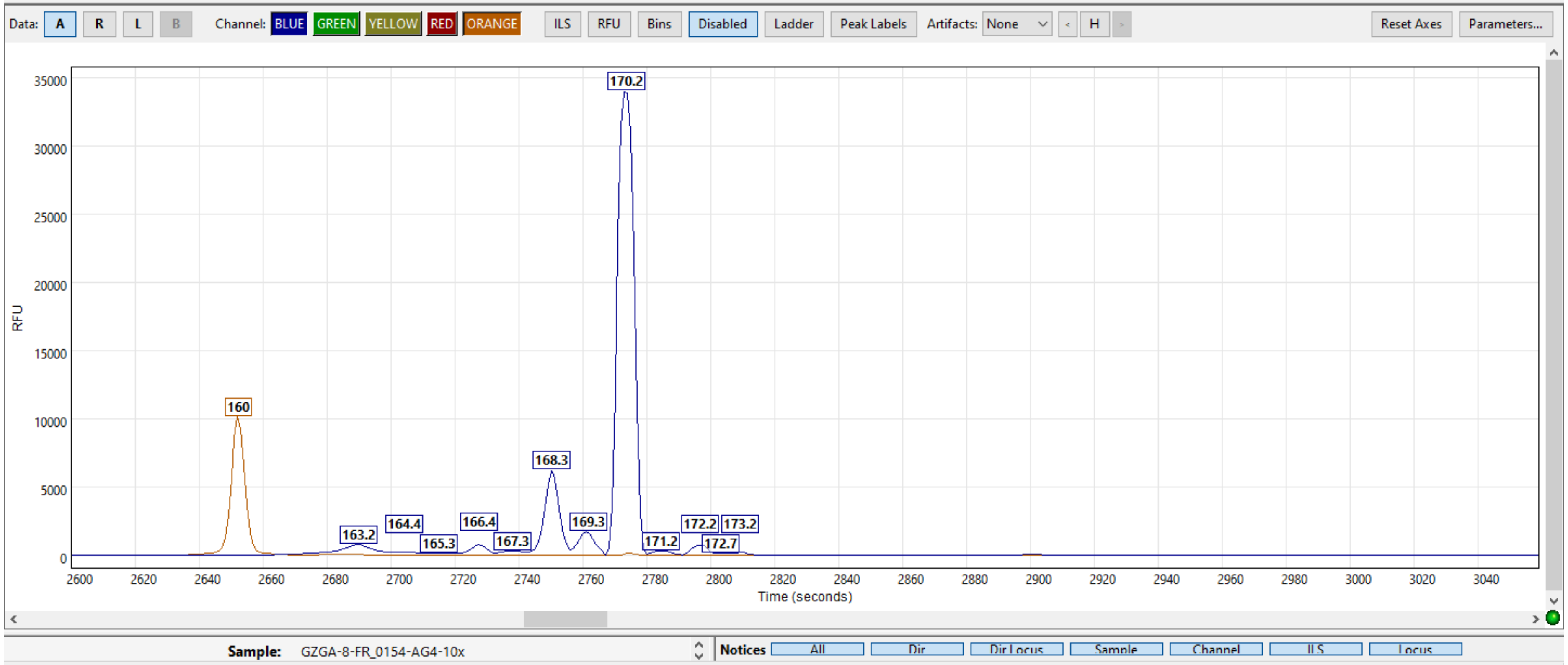


| | |
|---|---|
| **Observer 1** | **168;170** |
| **Observer 2** | **170.2** |
| **Observer 3** | **170.2** |

### 27- Wild. Locus AG4 sample GZGA9 (0155)

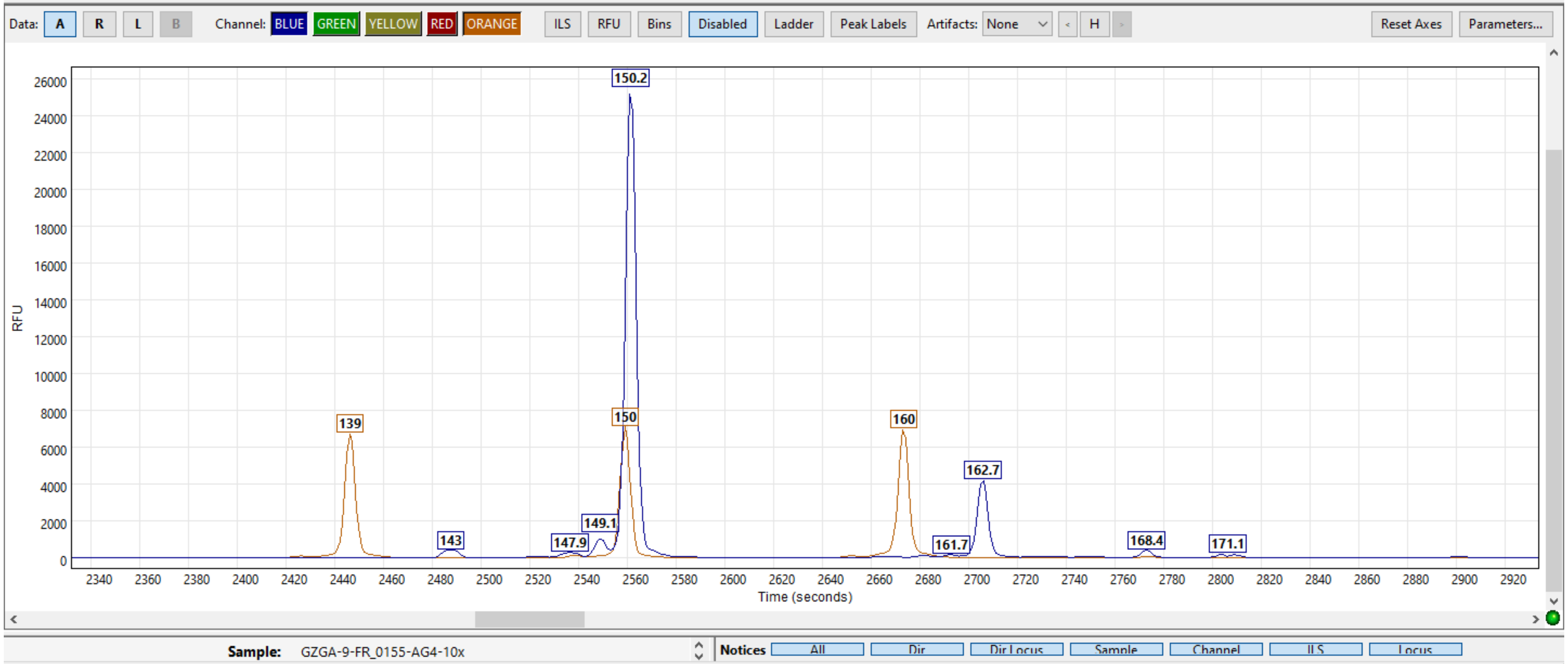


| | |
|---|---|
| **Observer 1** | **150;163** |
| **Observer 2** | **150.2 ; 162.7** |
| **Observer 3** | **150;163** |

**28- Wild. Locus AG4 sample MACC3 (0156)**

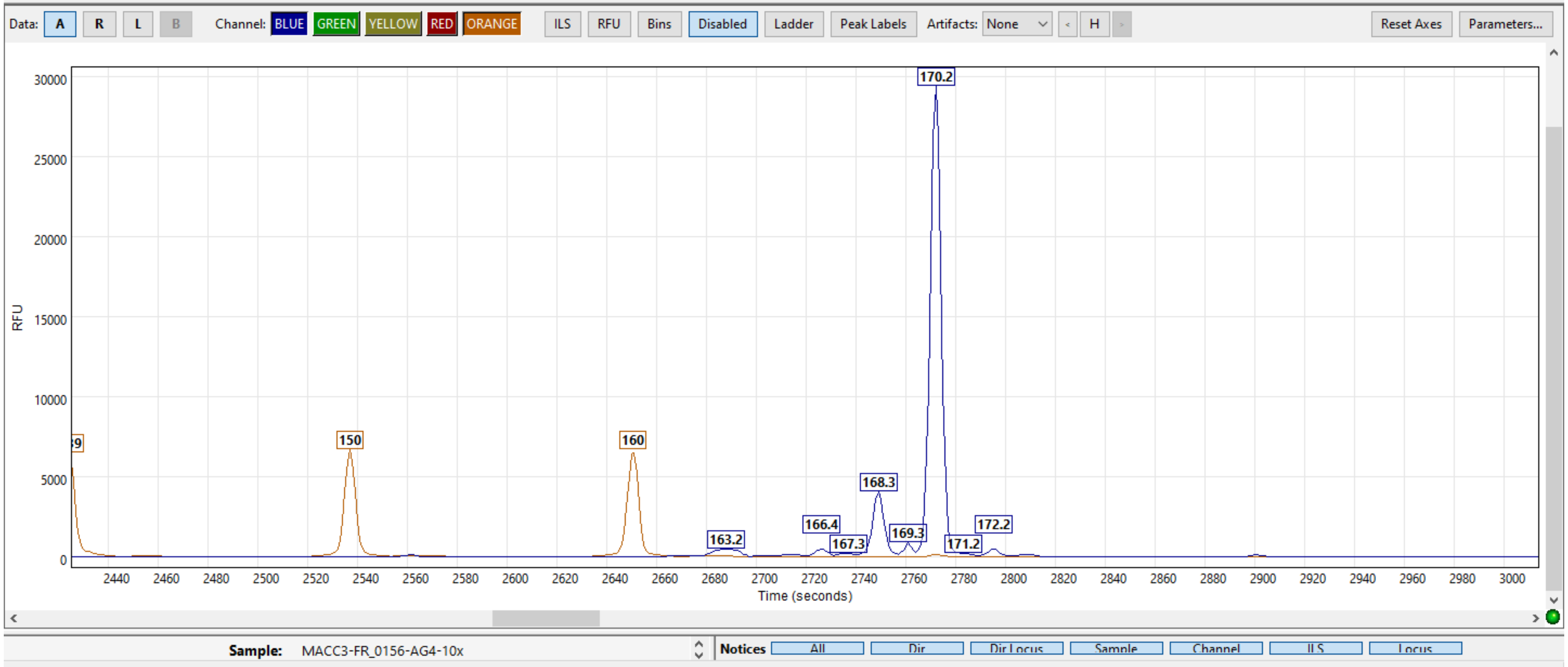


| | |
|---|---|
| **Observer 1** | **168;170** |
| **Observer 2** | **170.2** |
| **Observer 3** | **170.2** |

**29- Wild. Locus AG4 sample PONT92 (0157)**

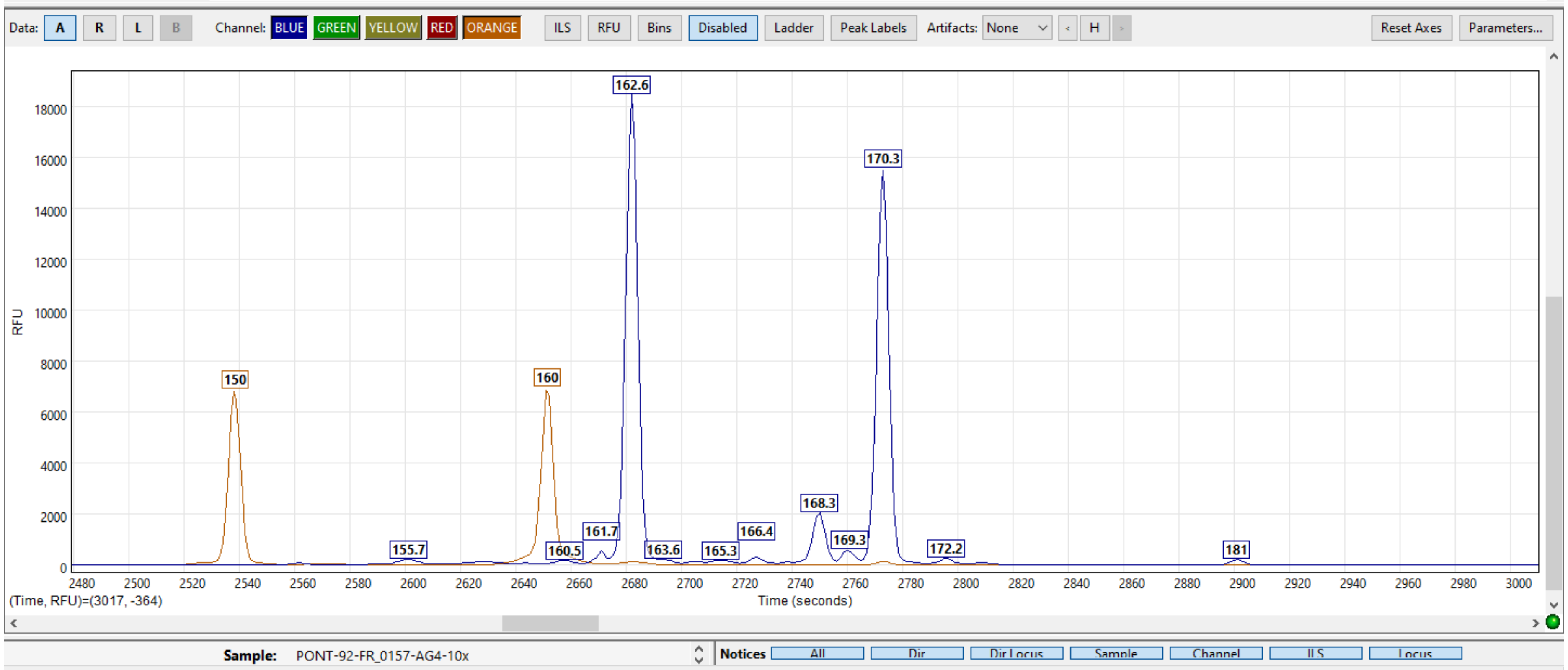


| | |
|---|---|
| **Observer 1** | **163;170** |
| **Observer 2** | **162.6;170.3** |
| **Observer 3** | **163;170** |

### 30- Wild. Locus AG4 sample PONT95 (0158)

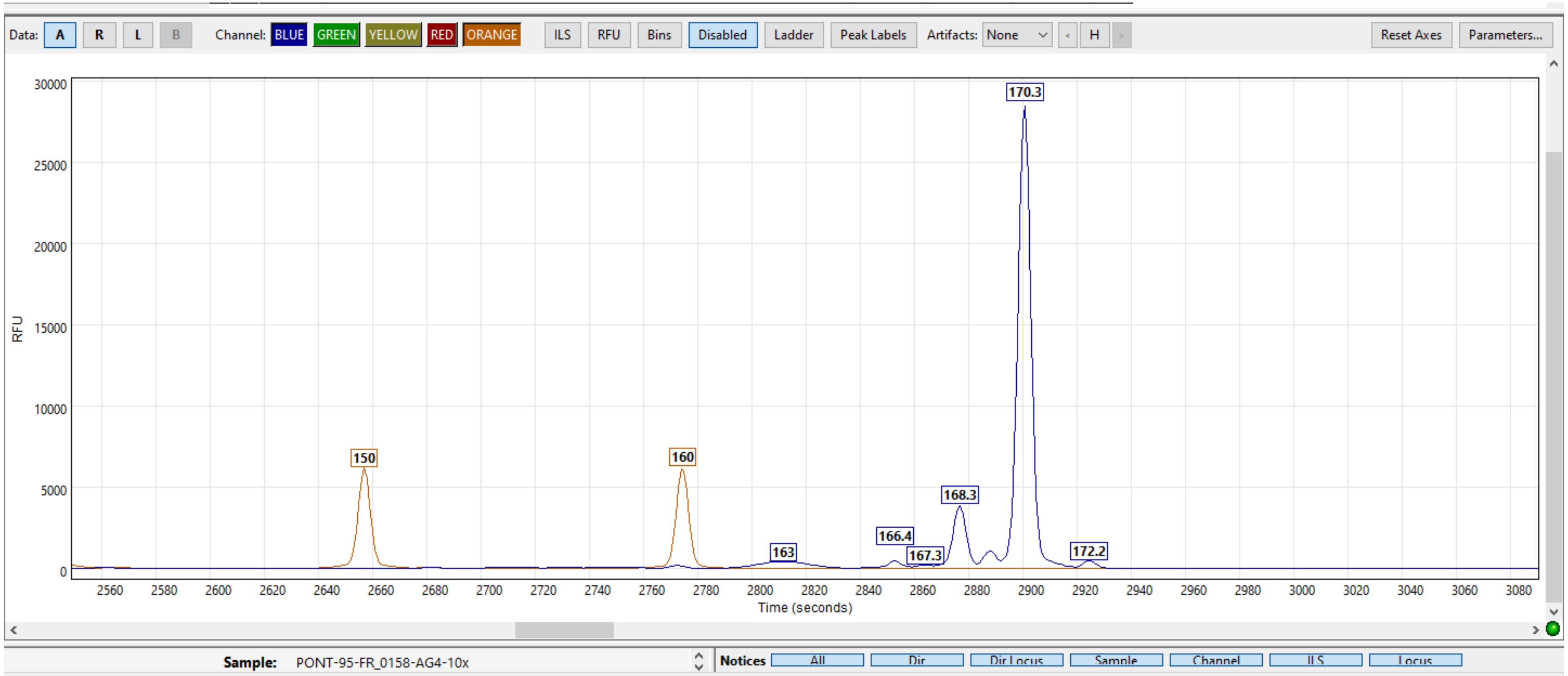


| | |
|---|---|
| **Observer 1** | **168;170** |
| **Observer 2** | **170.3** |
| **Observer 3** | **170.3** |

## AG7

## 1- Wild. Locus AG7 sample 01 (0909)

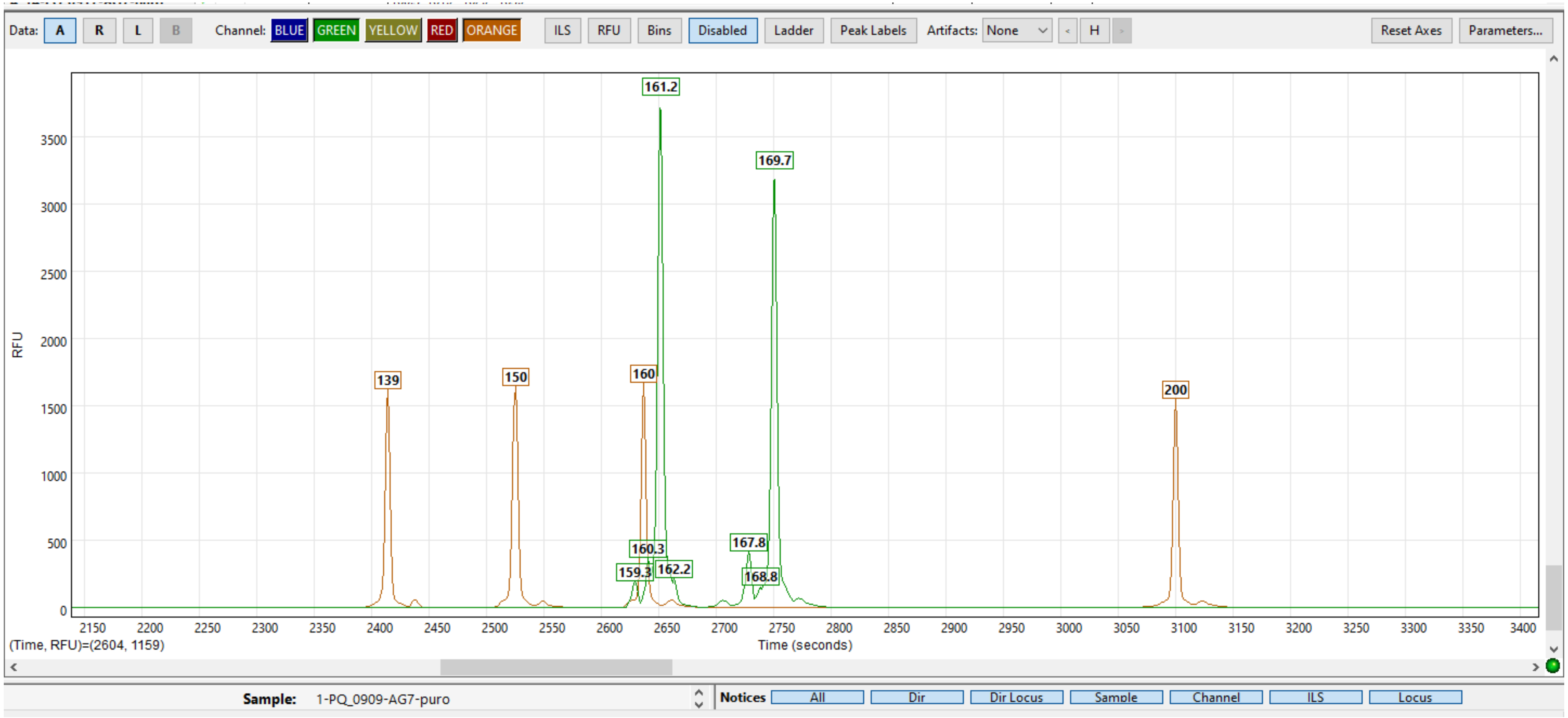


| | |
|---|---|
| **Observer 1** | **161;170** |
| **Observer 2** | **161.2;169.7** |
| **Observer 3** | **161;170** |

**2- Wild. Locus AG7 sample 03 (0910)**

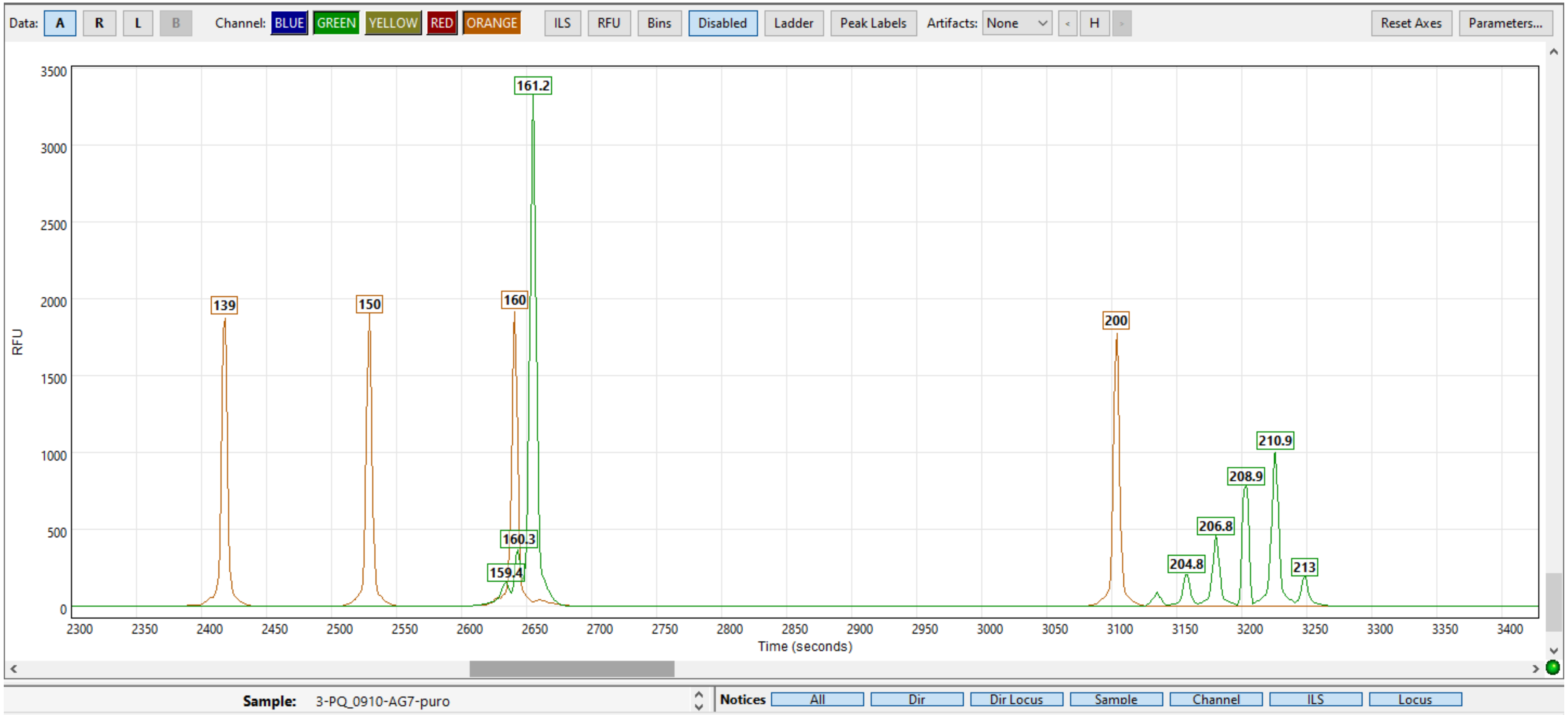


| | |
|---|---|
| **Observer 1** | **161;211** |
| **Observer 2** | **161.2;210.9** |
| **Observer 3** | **161;211** |

### 3- Wild. Locus AG7 sample 05 (0911)

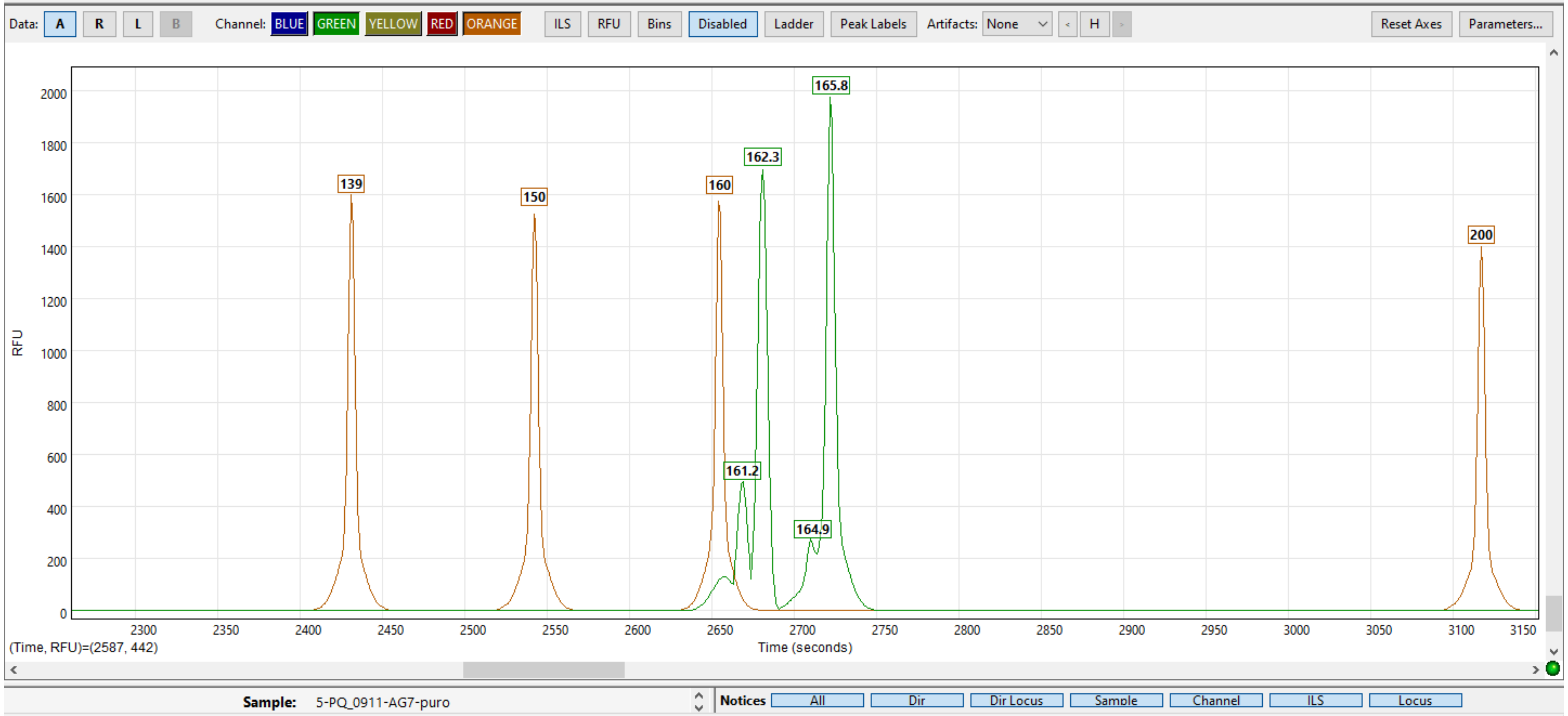


| | |
|---|---|
| **Observer 1** | **162;166** |
| **Observer 2** | **162.3;165.8** |
| **Observer 3** | **162;166** |

**4- Wild. Locus AG7 sample 07 (0912)**

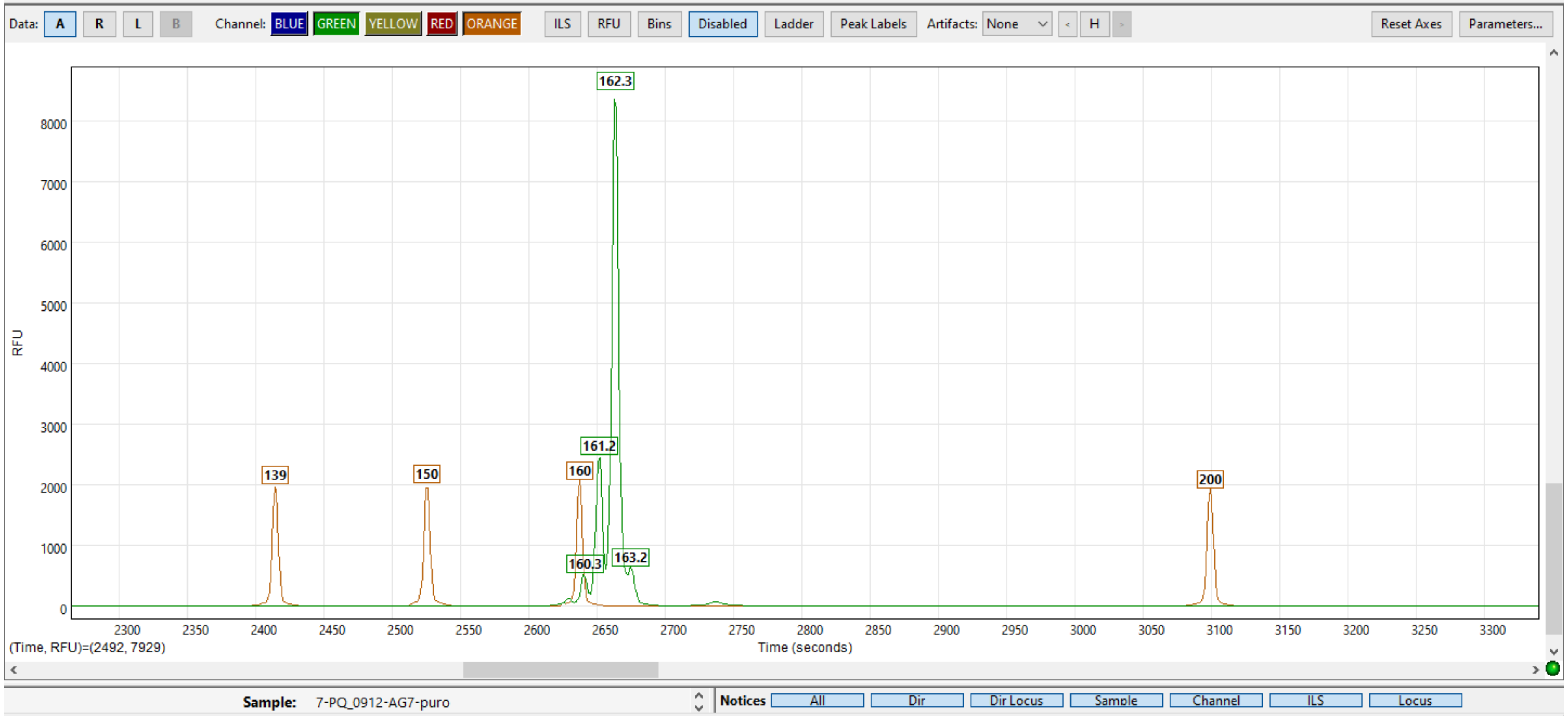


| | |
|---|---|
| **Observer 1** | **162** |
| **Observer 2** | **162.3** |
| **Observer 3** | **162** |

## 5- Wild. Locus AG7 sample 08 (0913)

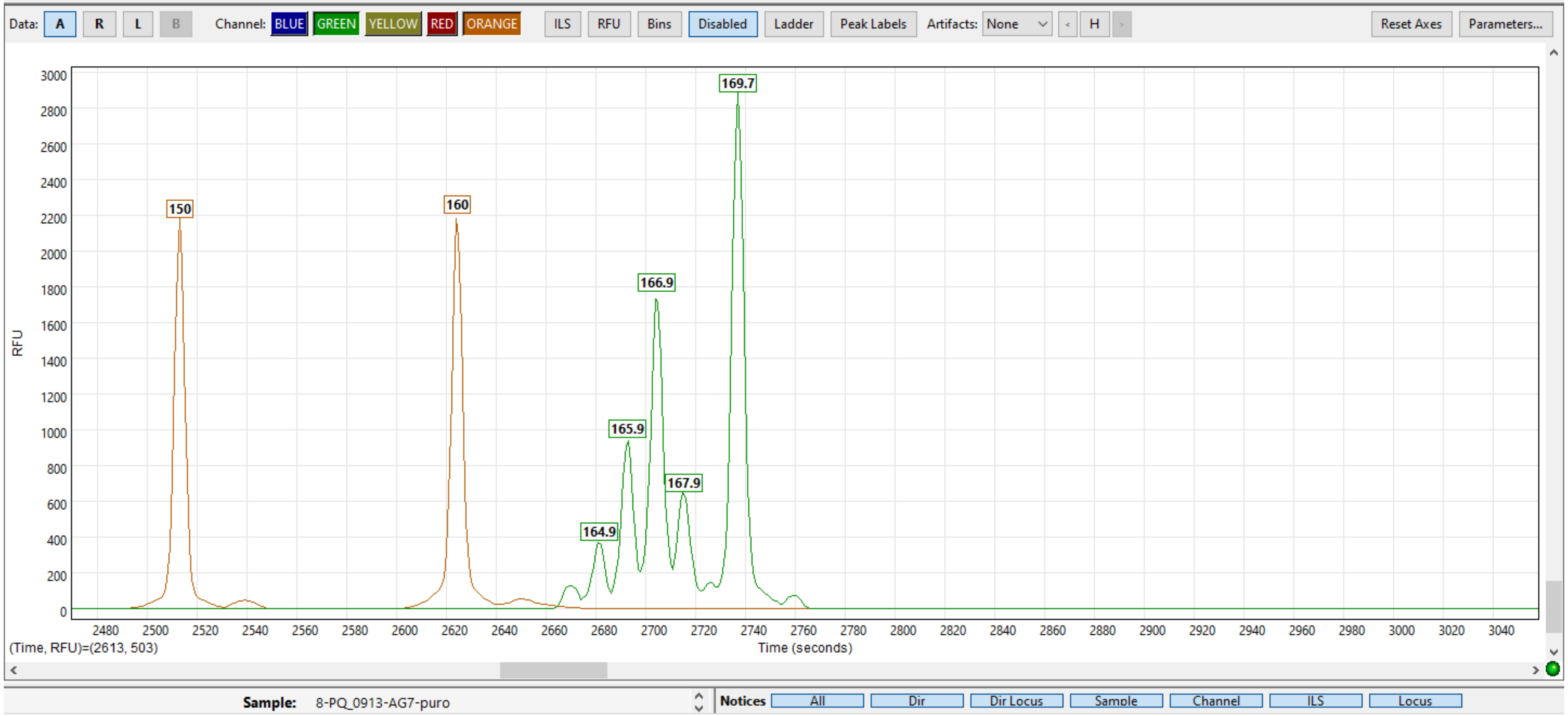


| | |
|---|---|
| **Observer 1** | **167;170** |
| **Observer 2** | **166.9;169.7** |
| **Observer 3** | **167;170** |

**6- Wild. Locus AG7 sample 09 (0914)**

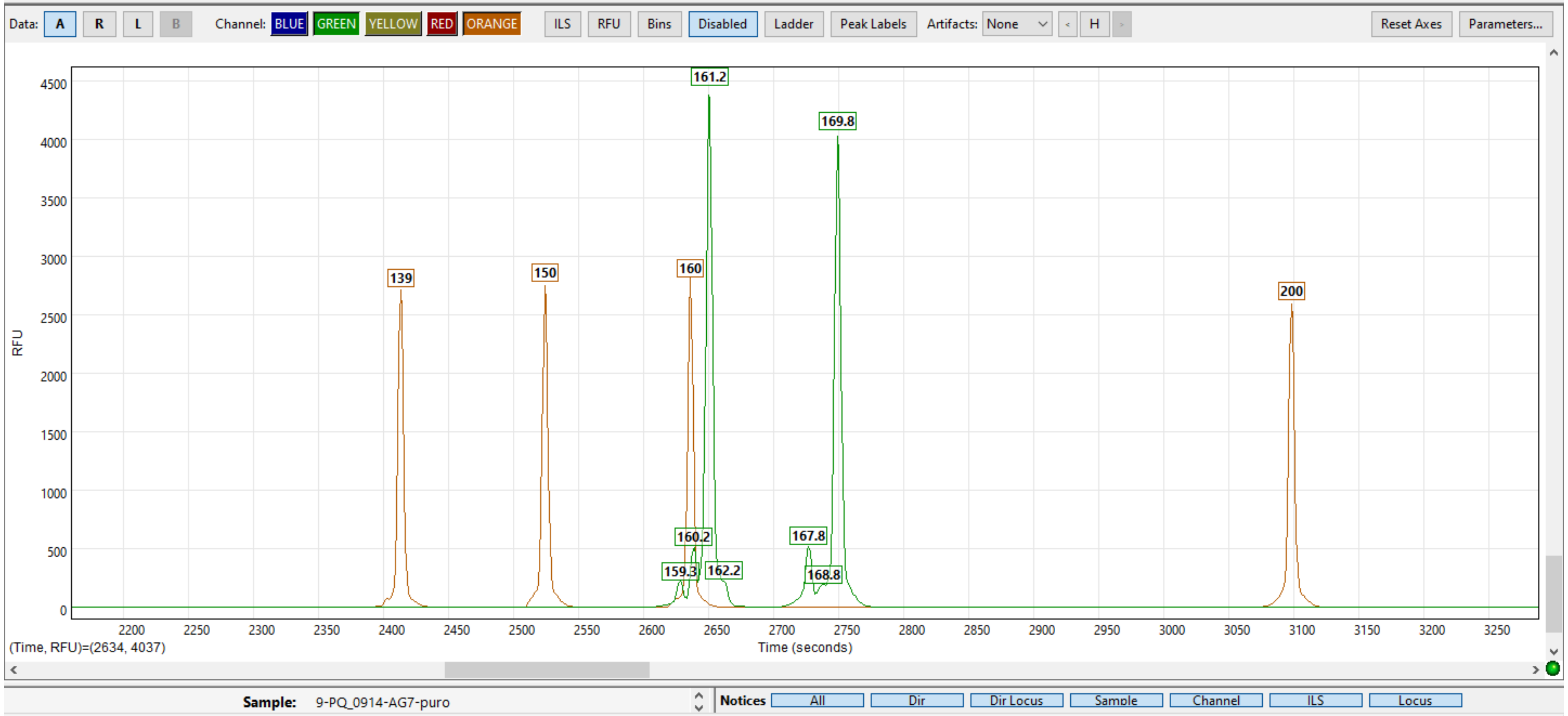


| | |
|---|---|
| **Observer 1** | **161;170** |
| **Observer 2** | **161.2;169.8** |
| **Observer 3** | **161;170** |

**7- Wild. Locus AG7 sample 12 (0915)**

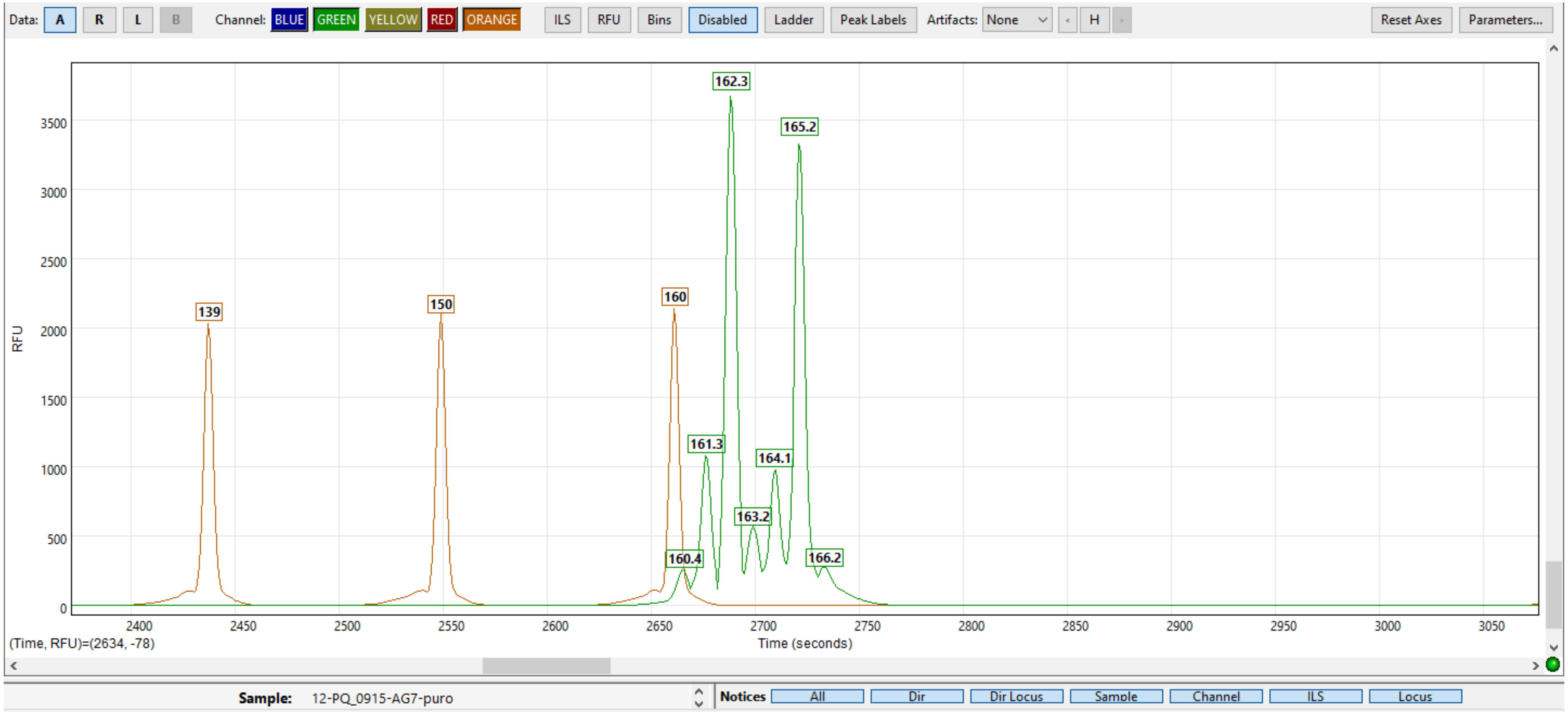


| | |
|---|---|
| **Observer 1** | **162;165** |
| **Observer 2** | **162.3;165.2** |
| **Observer 3** | **162;165** |

## 8- Wild. Locus AG7 sample 13 (0916)

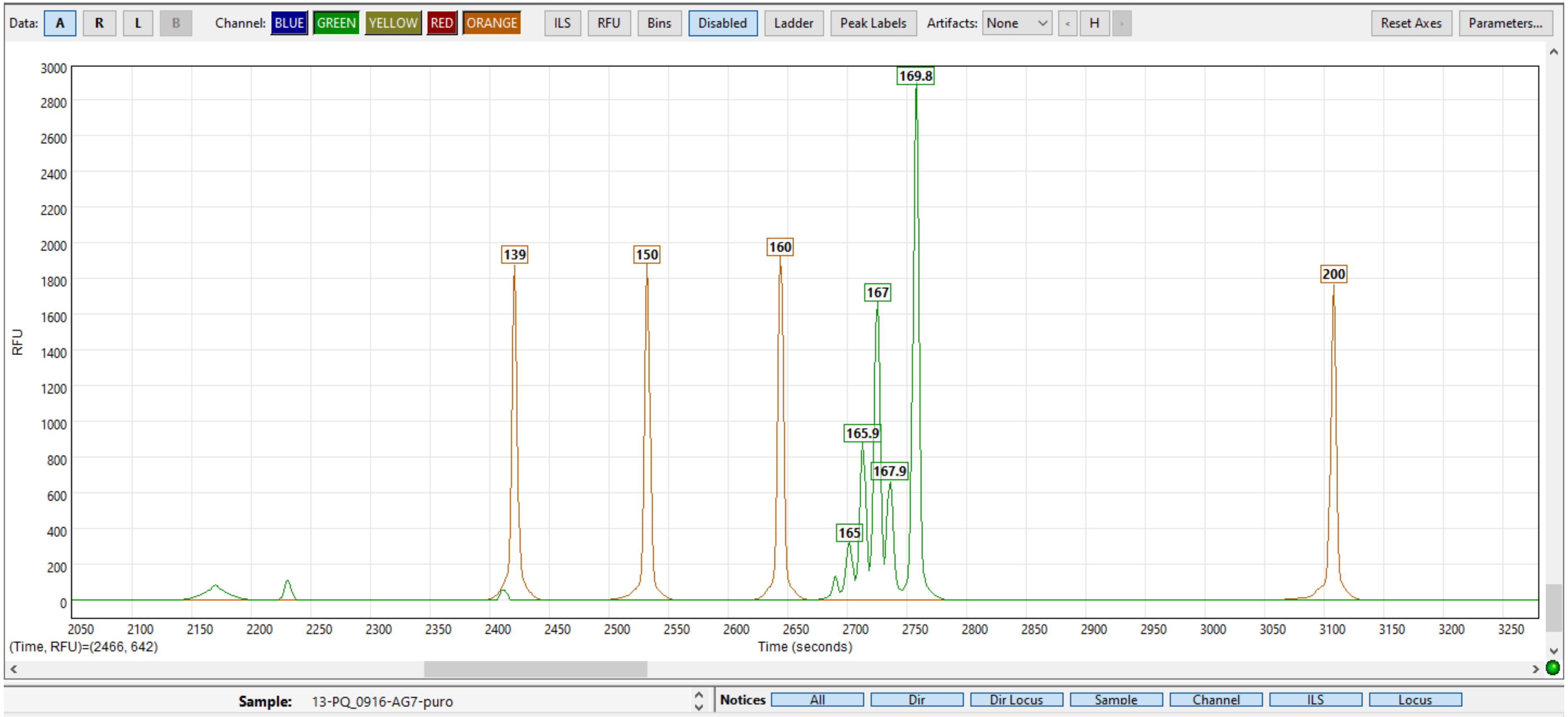


| | |
|---|---|
| **Observer 1** | **167;170** |
| **Observer 2** | **167;169.8** |
| **Observer 3** | **167;170** |

9- Wild. Locus AG7 sample 14 (0917)

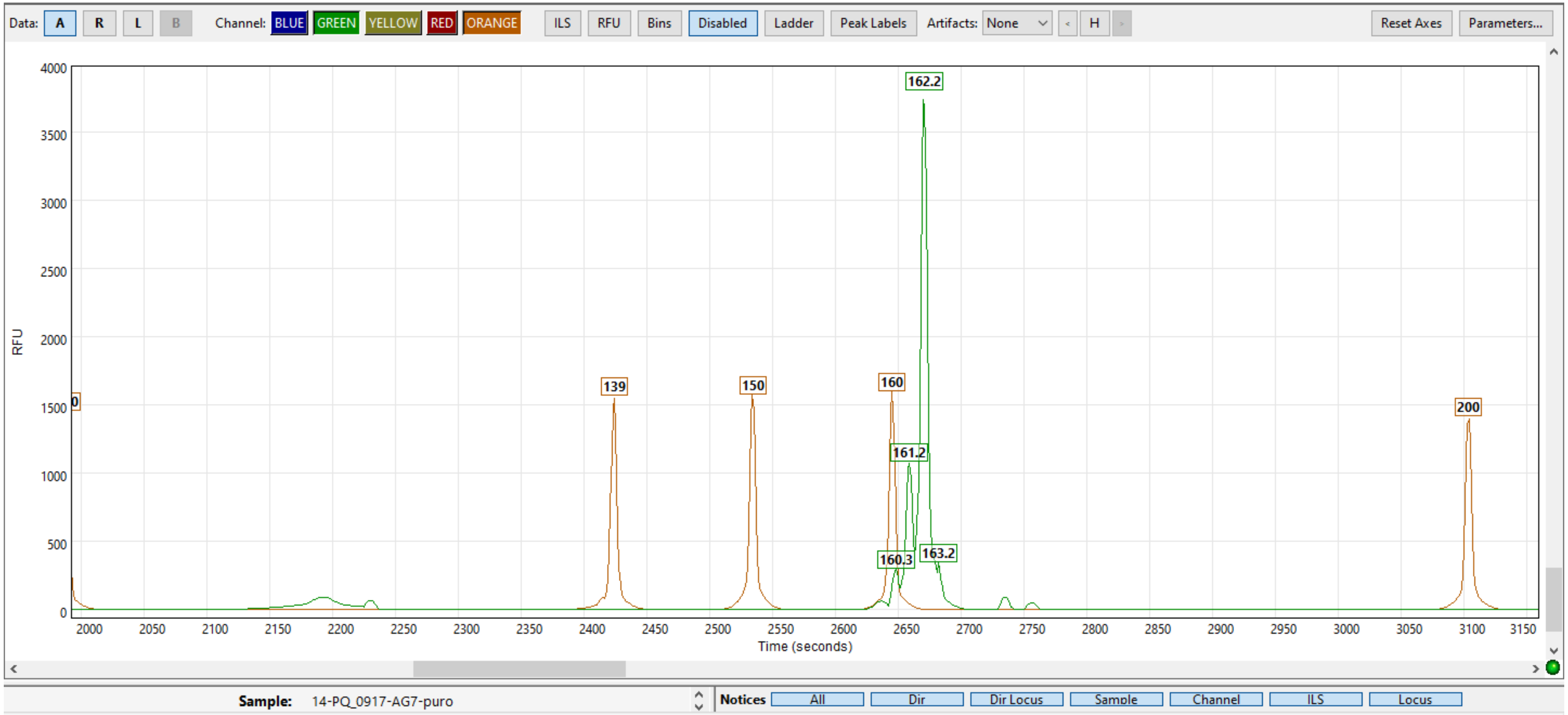


| | |
|---|---|
| **Observer 1** | **162** |
| **Observer 2** | **162.2** |
| **Observer 3** | **162** |

**10- Wild. Locus AG7 sample 15 (0918)**

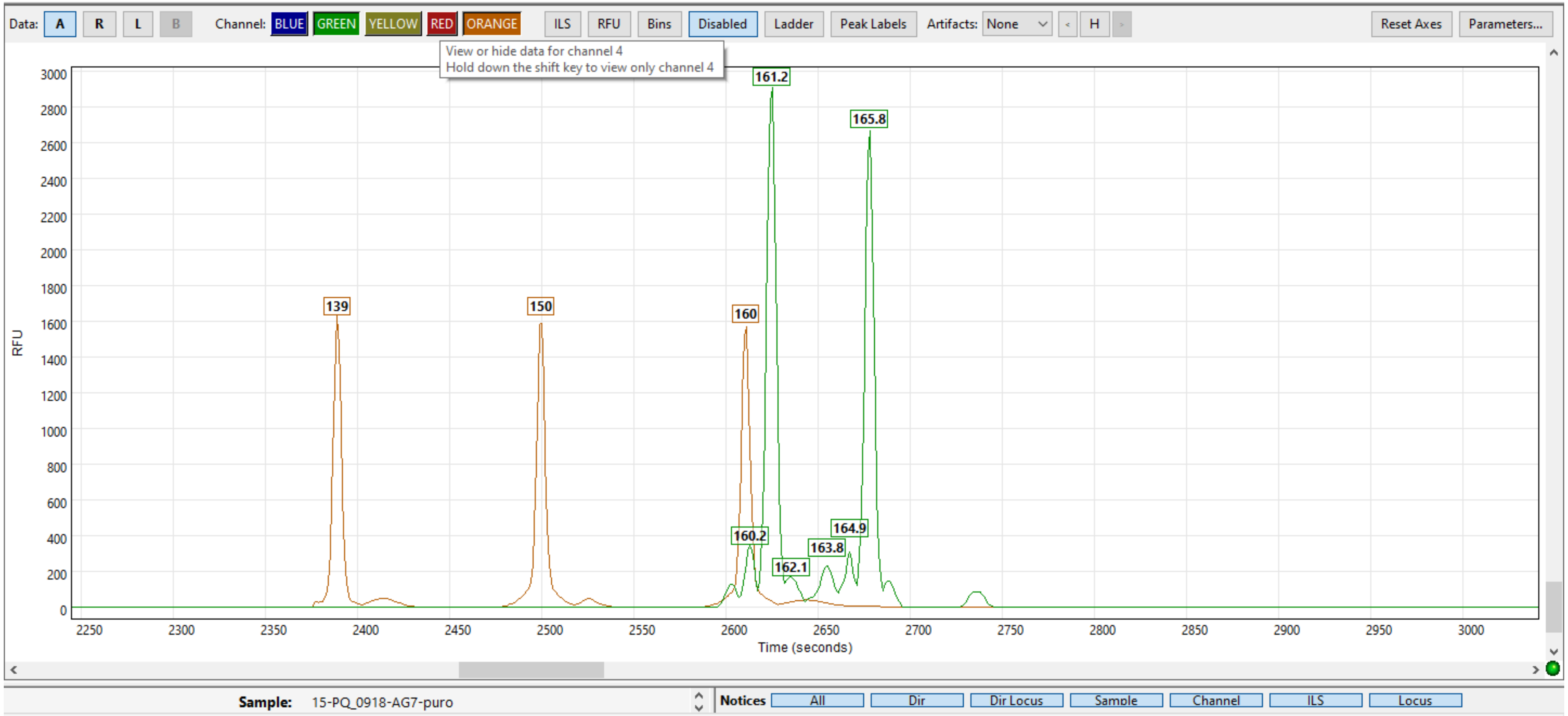


| | |
|---|---|
| **Observer 1** | **161;166** |
| **Observer 2** | **161.2;165.8** |
| **Observer 3** | **162;166** |

**11- Wild. Locus AG7 sample 16 (0919)**

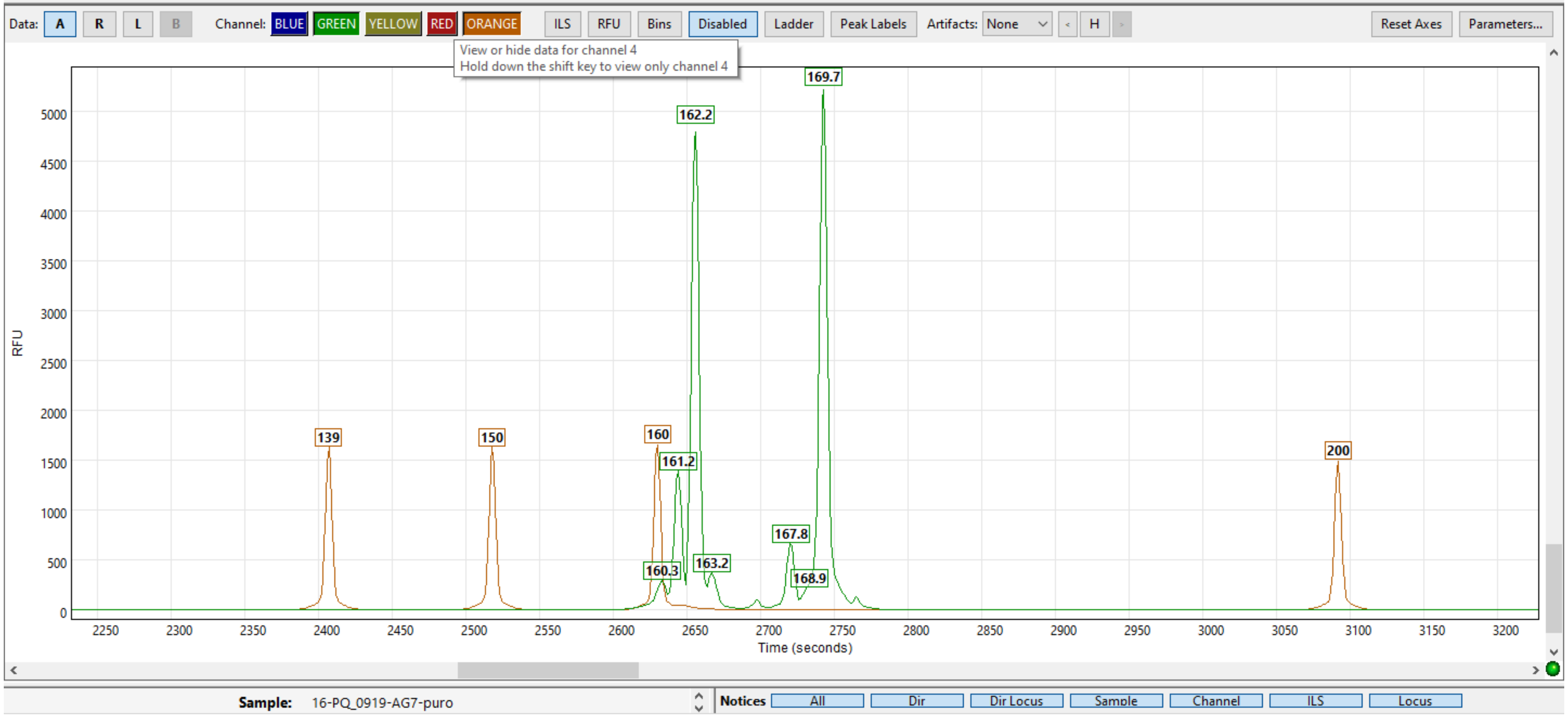


| | |
|---|---|
| **Observer 1** | **162;170** |
| **Observer 2** | **162.2;169.7** |
| **Observer 3** | **162;170** |

## 12- Wild. Locus AG7 sample 17 (0920)

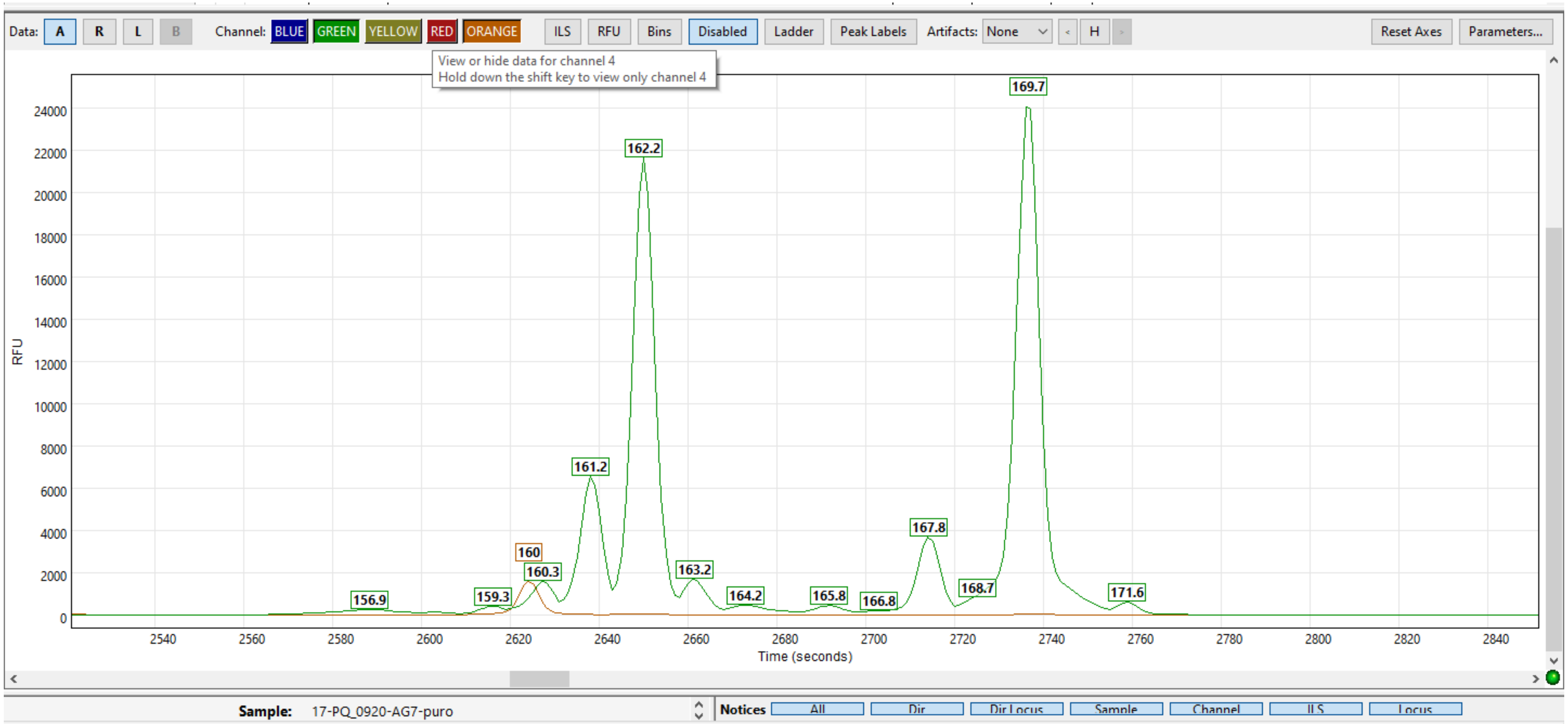


| | |
|---|---|
| **Observer 1** | **162;170** |
| **Observer 2** | **162.2;169.7** |
| **Observer 3** | **162;170** |

## 13- Wild. Locus AG7 sample 19 (0921)

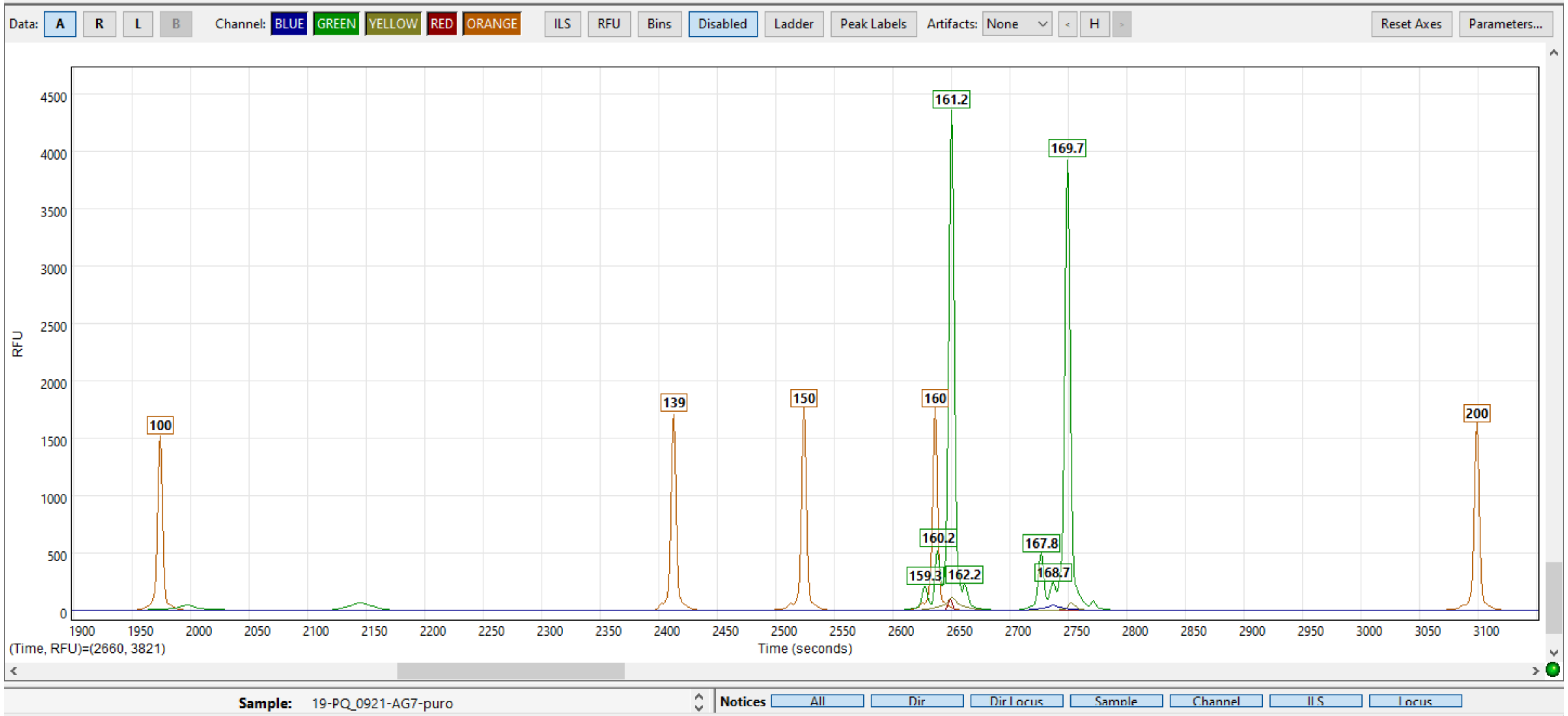


| | |
|---|---|
| **Observer 1** | **161;170** |
| **Observer 2** | **161.2;169.7** |
| **Observer 3** | **161;170** |

**14- Wild. Locus AG7 sample 20 (0922)**

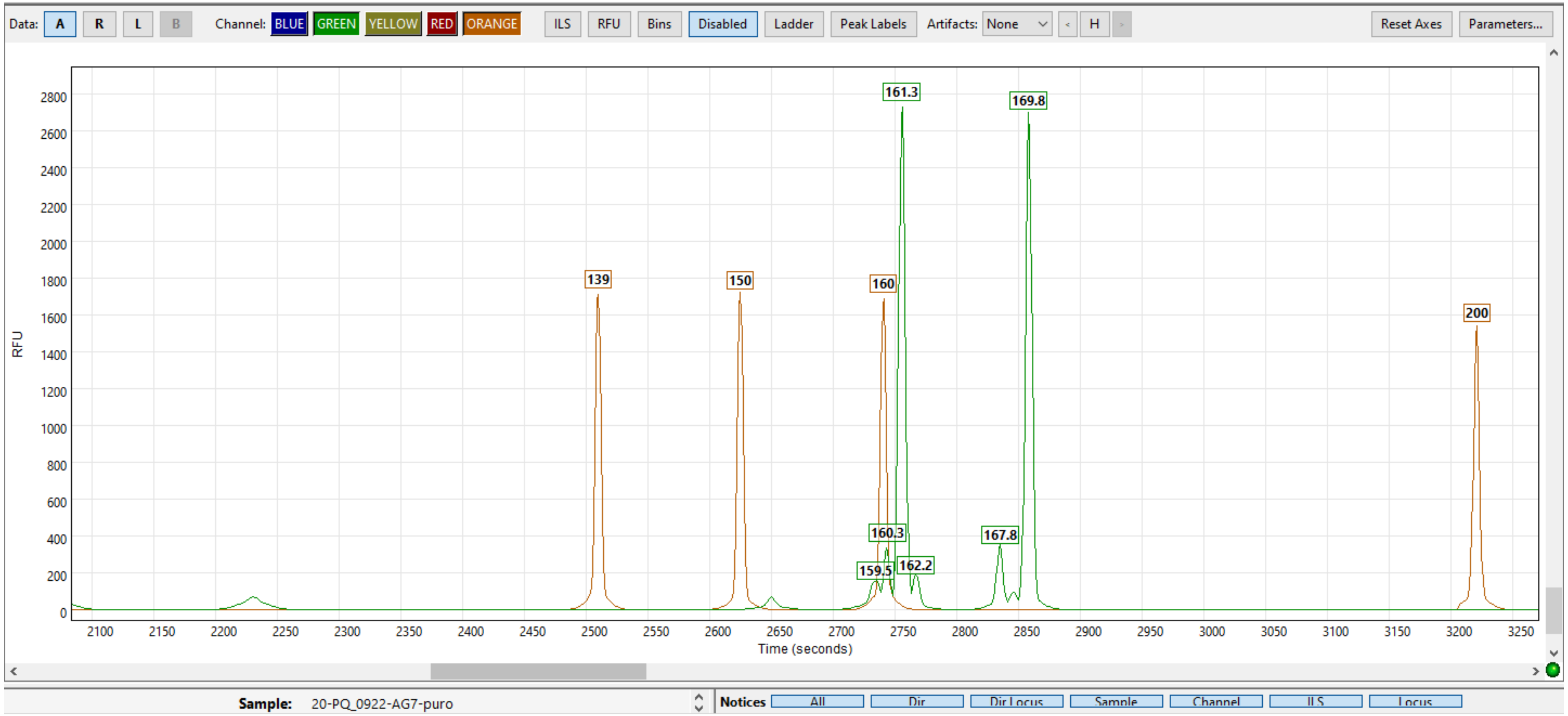


| | |
|---|---|
| **Observer 1** | **161;170** |
| **Observer 2** | **161.3;169.8** |
| **Observer 3** | **161;170** |

## 15- Wild. Locus AG7 sample 21 (0924)

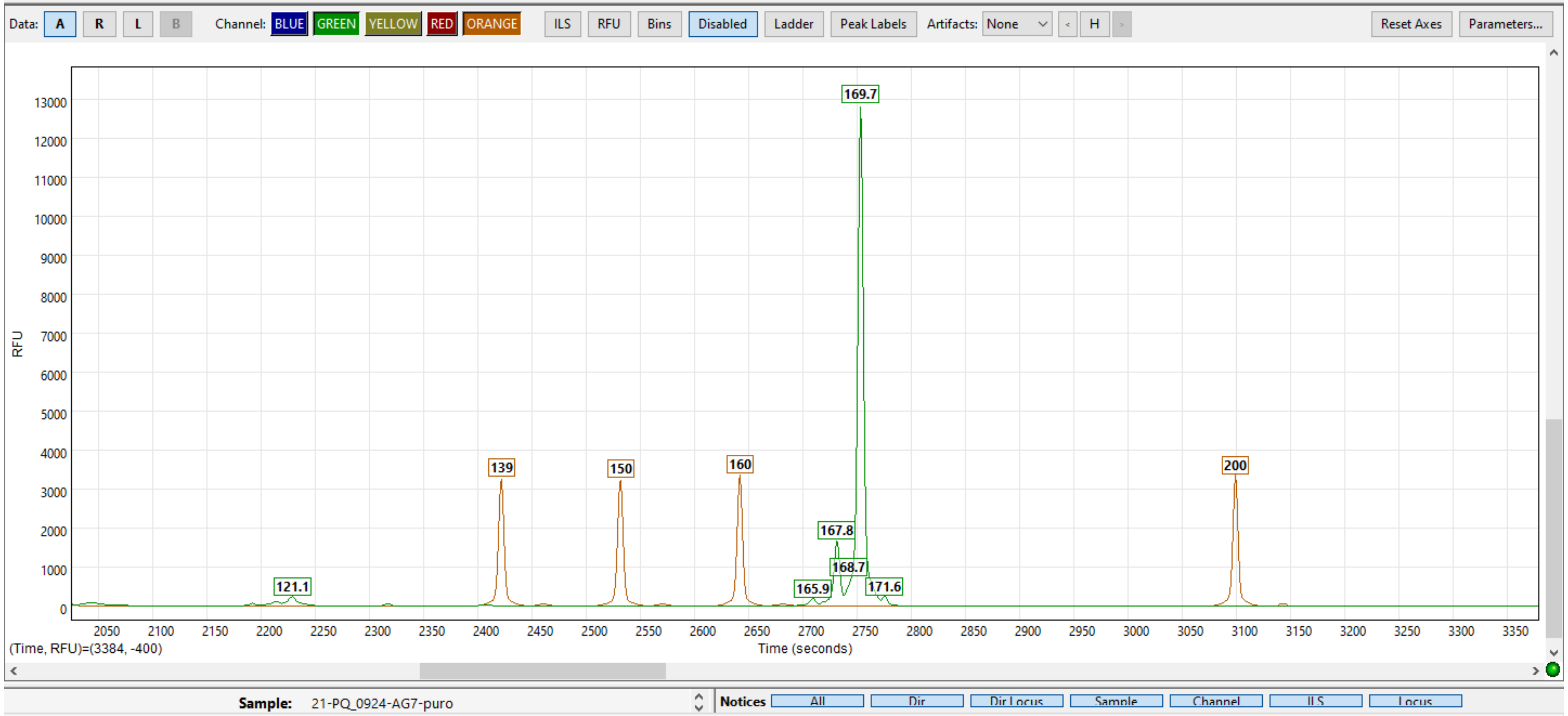


| | |
|---|---|
| **Observer 1** | **170** |
| **Observer 2** | **169.7** |
| **Observer 3** | **170** |

**16- Wild. Locus AG7 sample CAST2 (0144)**

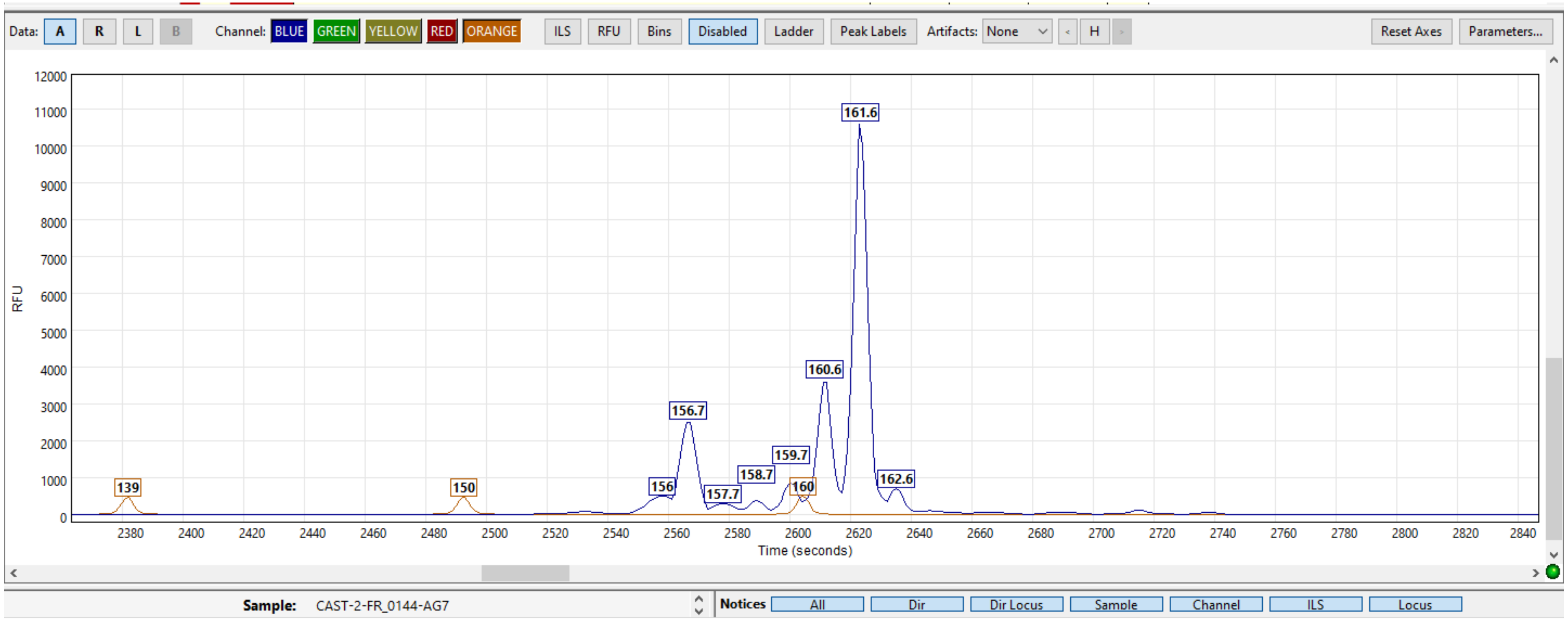


| | |
|---|---|
| **Observer 1** | **157;162** |
| **Observer 2** | **161.6** |
| **Observer 3** | **157;162** |

## 17- Wild. Locus AG7 sample CAST3 (0145)

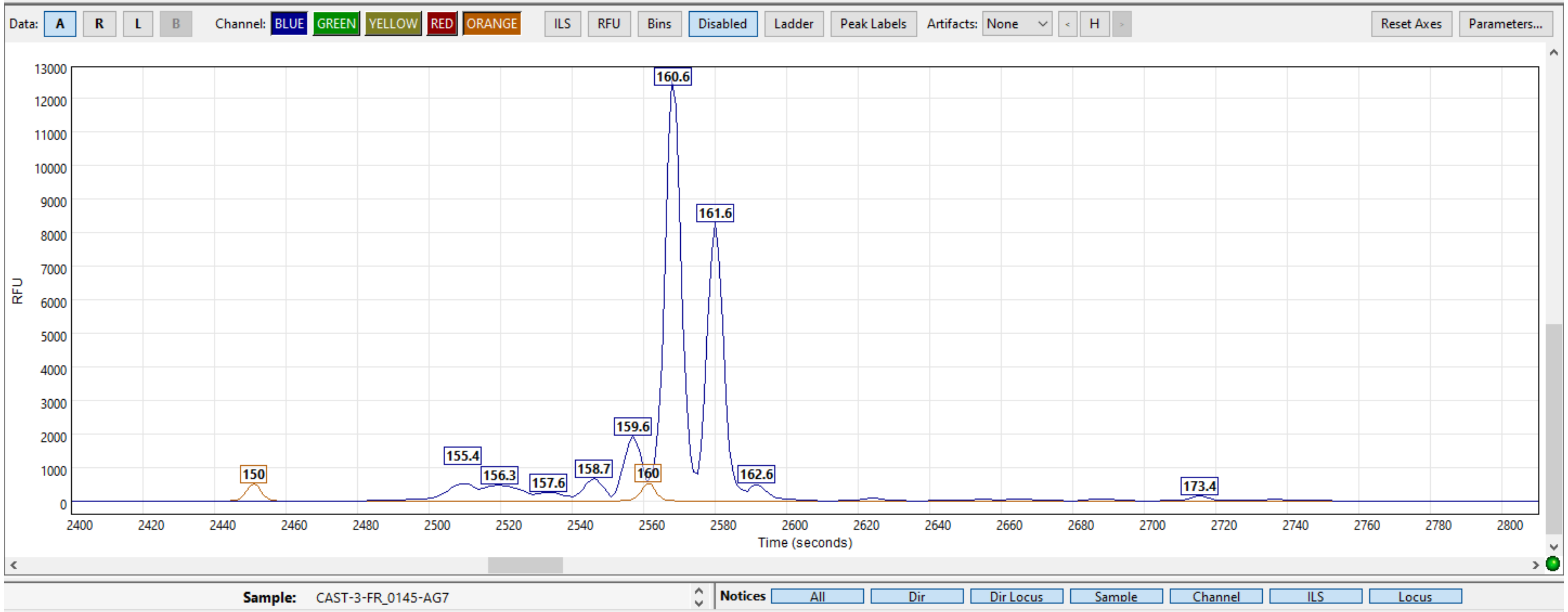


| | |
|---|---|
| **Observer 1** | **161** |
| **Observer 2** | **160.6** |
| **Observer 3** | **160;161** |

## 18- Wild. Locus AG7 sample CENT146 (0146)

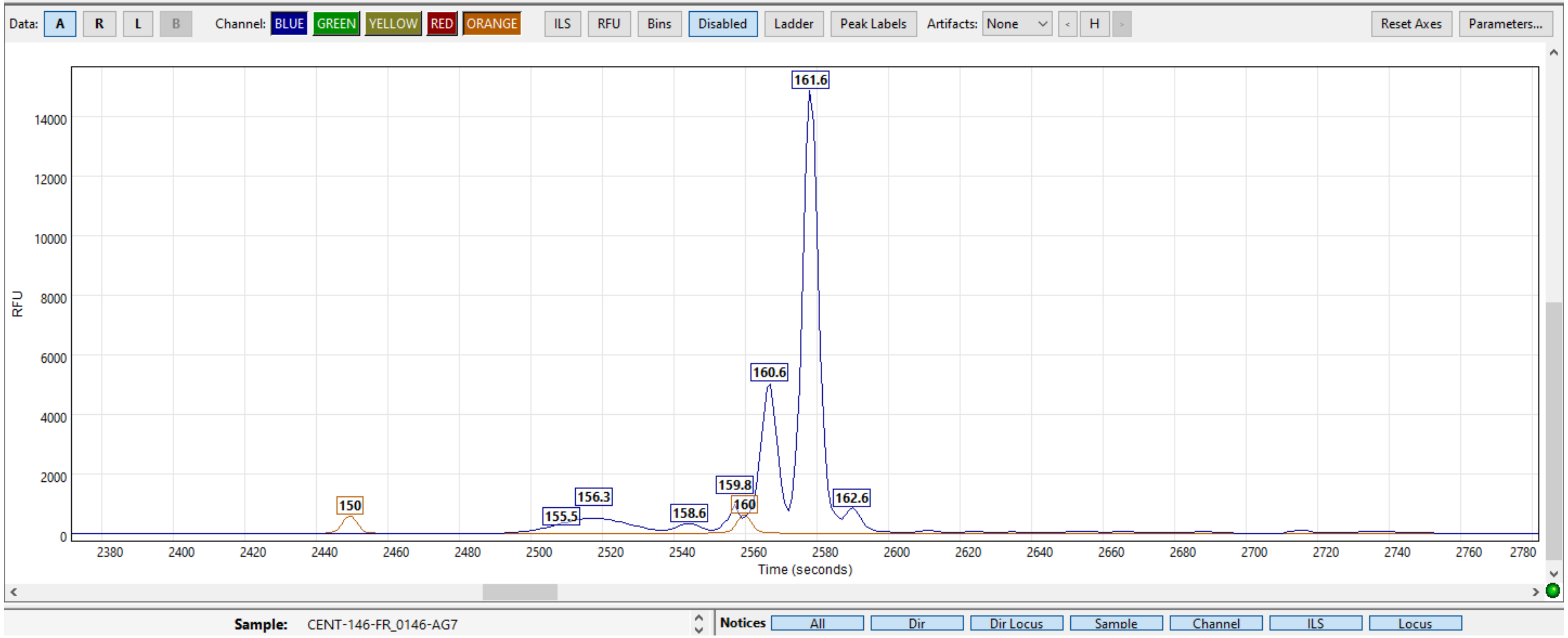


| | |
|---|---|
| **Observer 1** | **162** |
| **Observer 2** | **161.6** |
| **Observer 3** | **162** |

## 19- Wild. Locus AG7 sample CENT151 (0147)

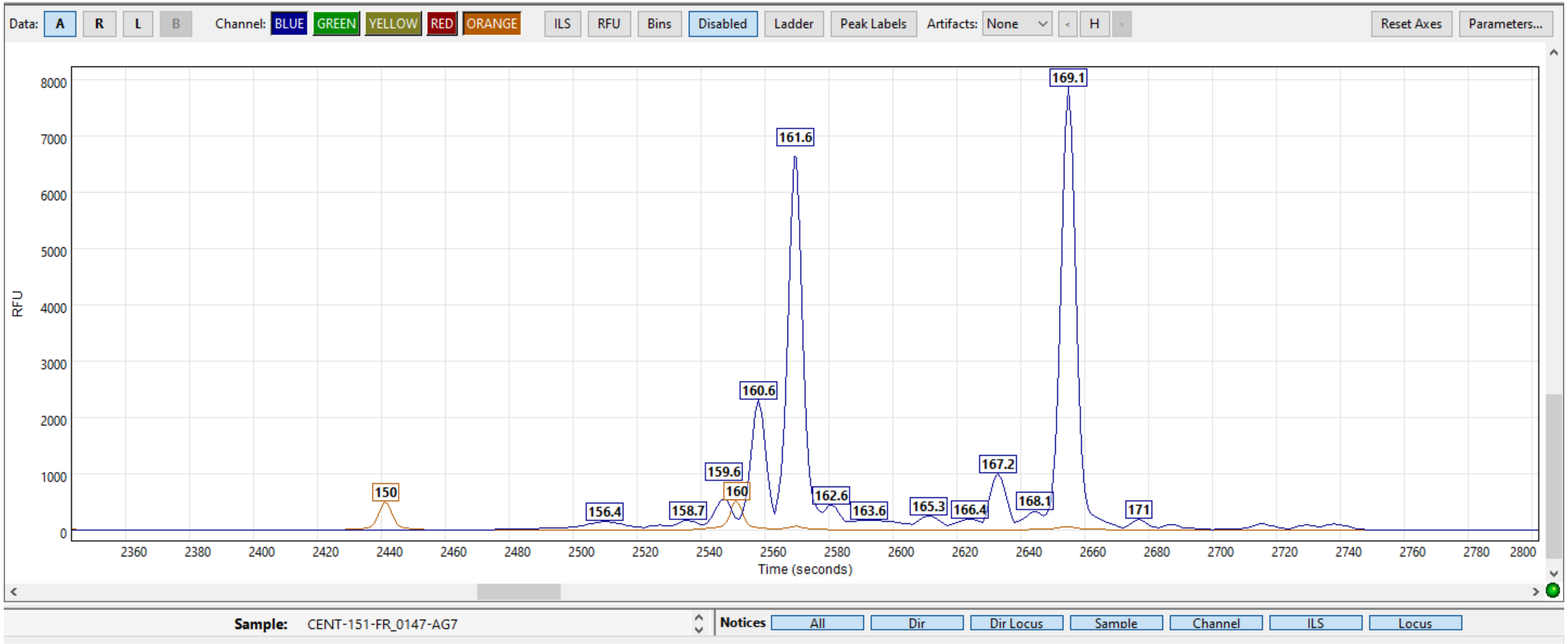


| | |
|---|---|
| **Observer 1** | **162;169** |
| **Observer 2** | **161.6;169.1** |
| **Observer 3** | **162;169** |

## 20- Wild. Locus AG7 sample CENT152 (0148)

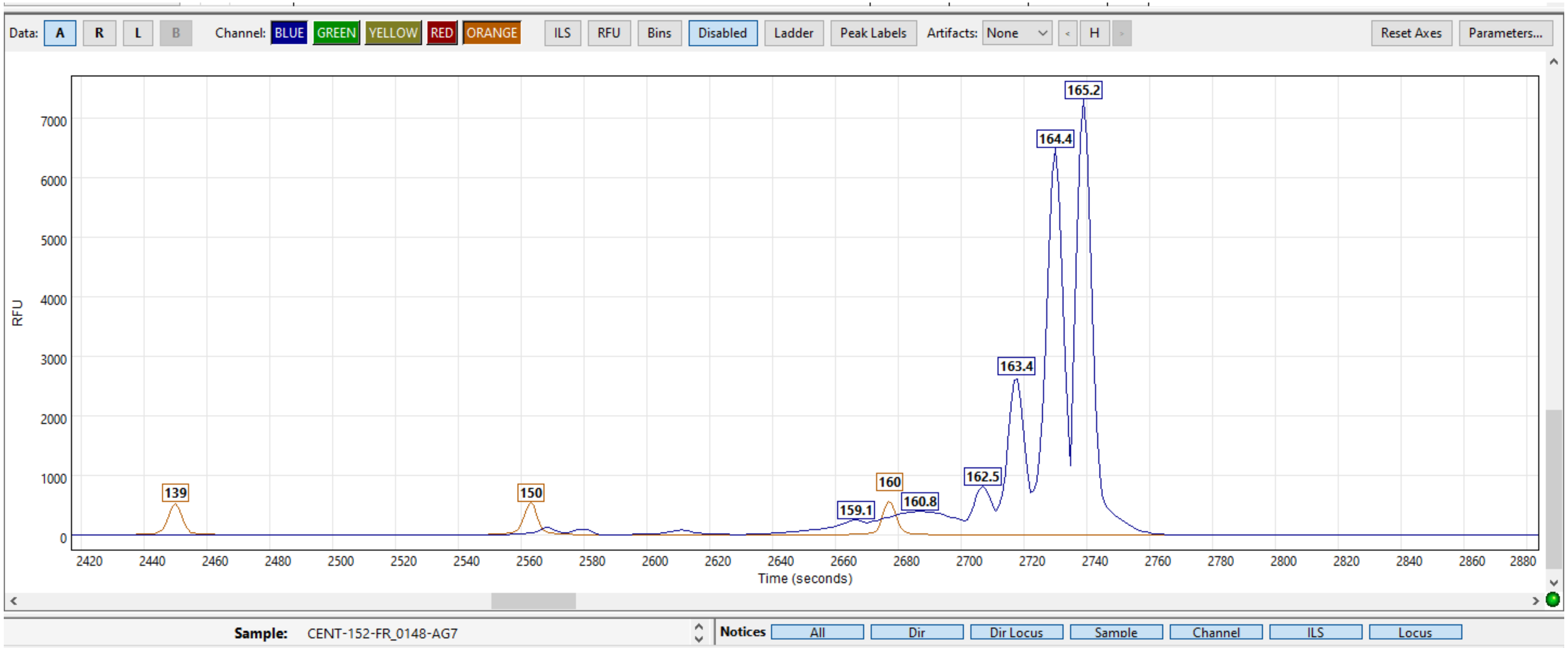


| | |
|---|---|
| **Observer 1** | **165** |
| **Observer 2** | **165.2** |
| **Observer 3** | **164;165** |

**21- Wild. Locus AG7 sample CENT155 (0149)**

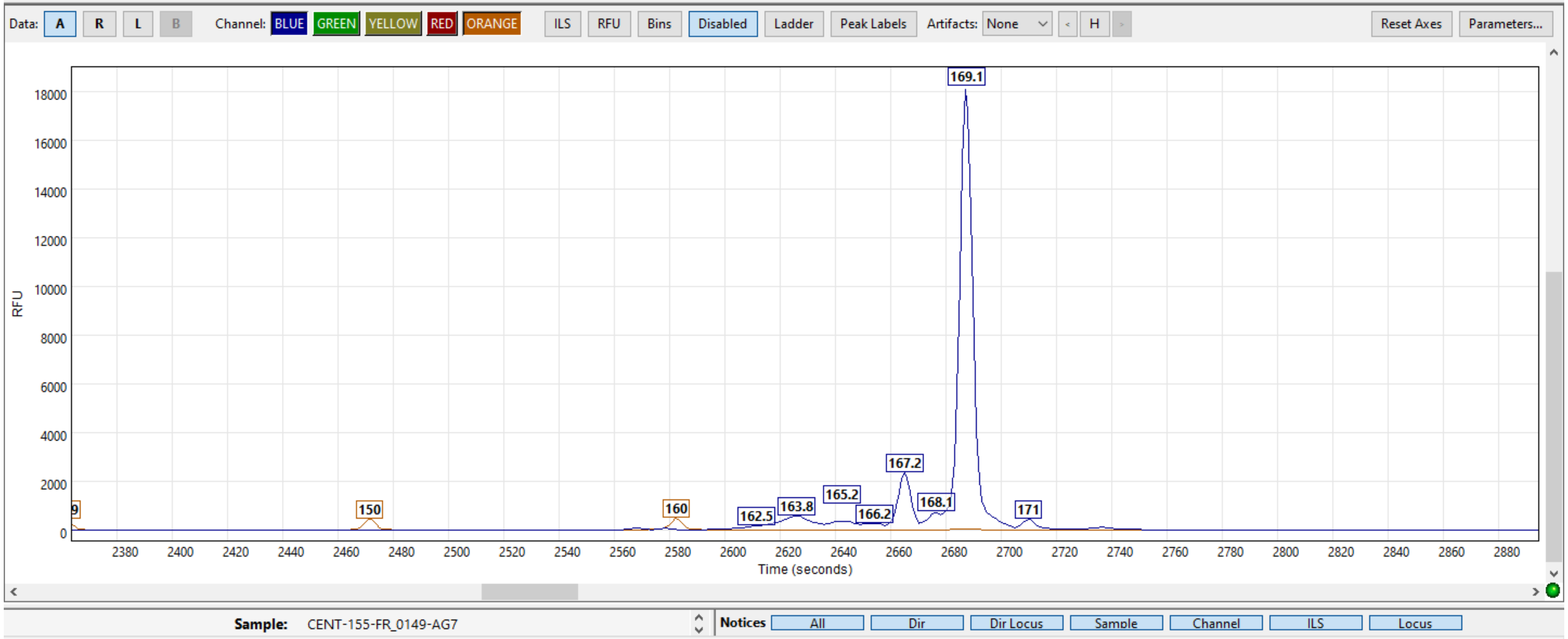


| | |
|---|---|
| **Observer 1** | **169** |
| **Observer 2** | **169.1** |
| **Observer 3** | **169** |

22- Wild. Locus AG7 sample CENT157 (0150)

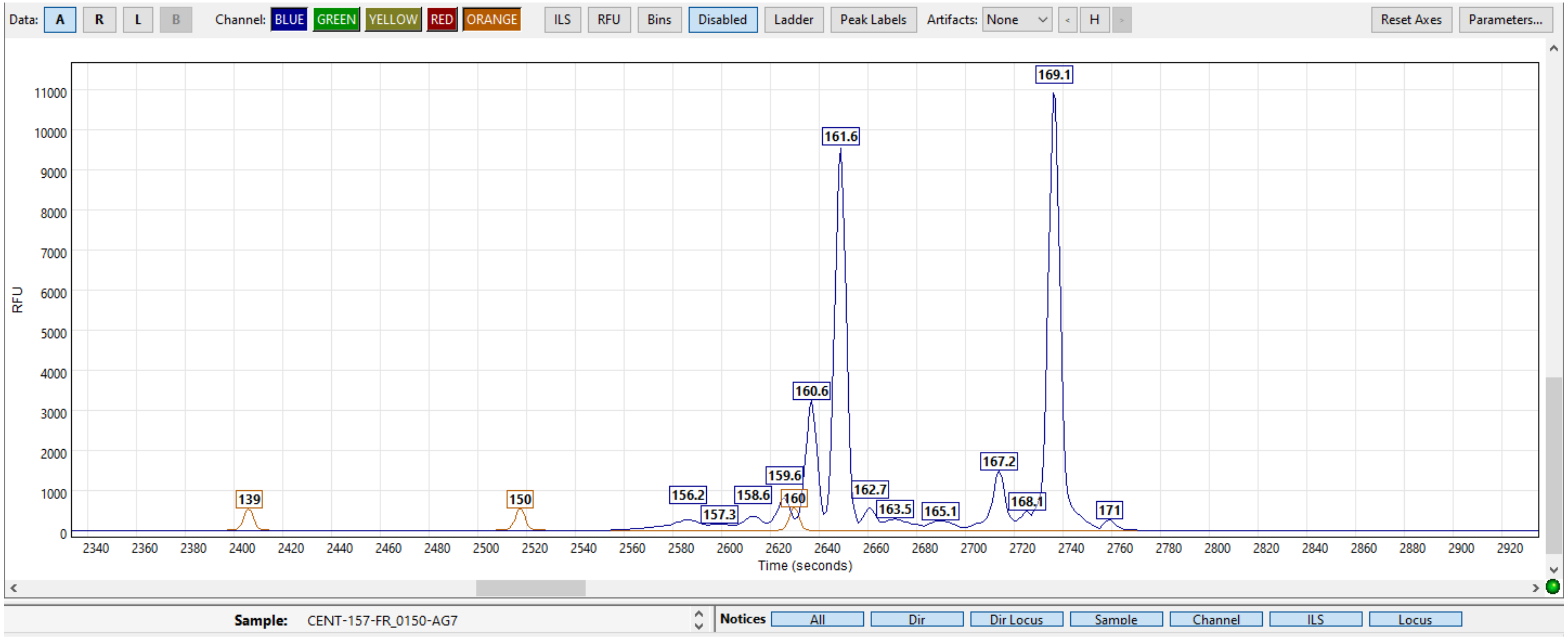


| | |
|---|---|
| **Observer 1** | **162;169** |
| **Observer 2** | **161.6;169.1** |
| **Observer 3** | **162;169** |

## 23- Wild. Locus AG7 sample CENT160 (0151)

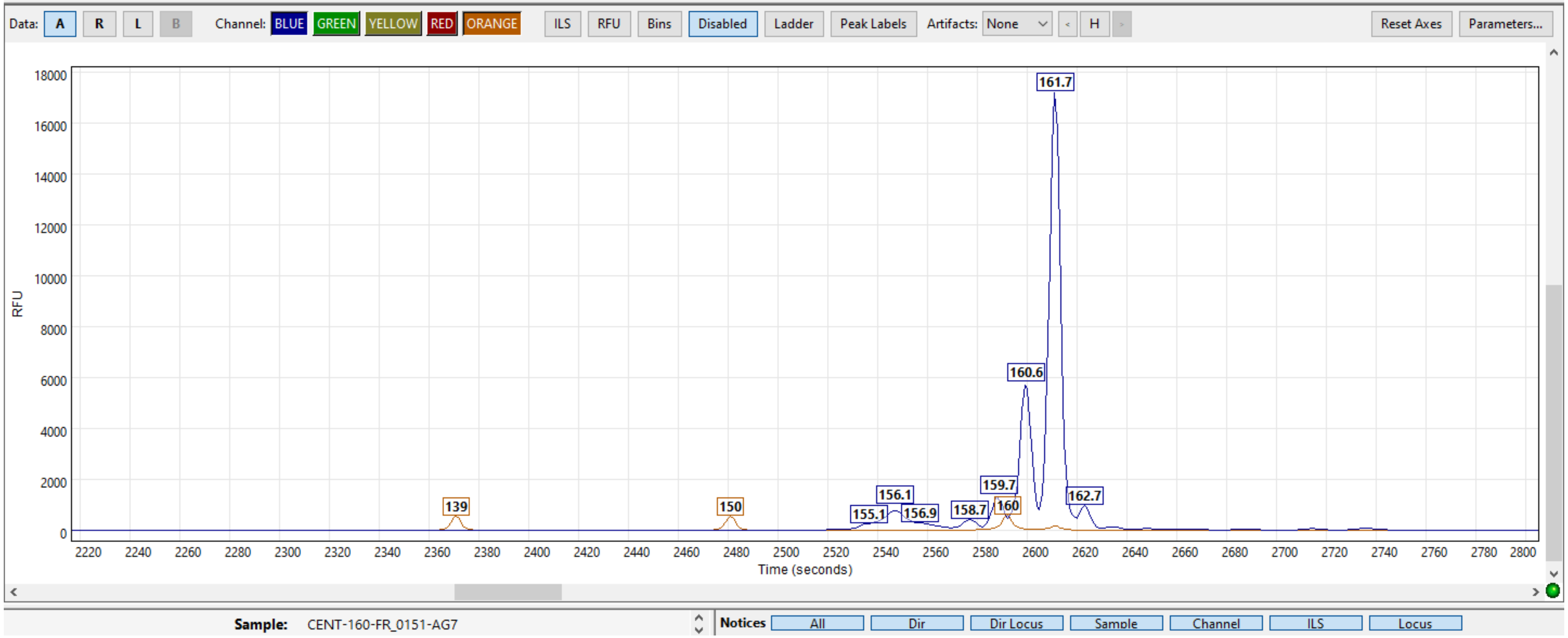


| | |
|---|---|
| **Observer 1** | **162** |
| **Observer 2** | **161.7** |
| **Observer 3** | **162** |

## 24- Wild. Locus AG7 sample CENT162 (0152)

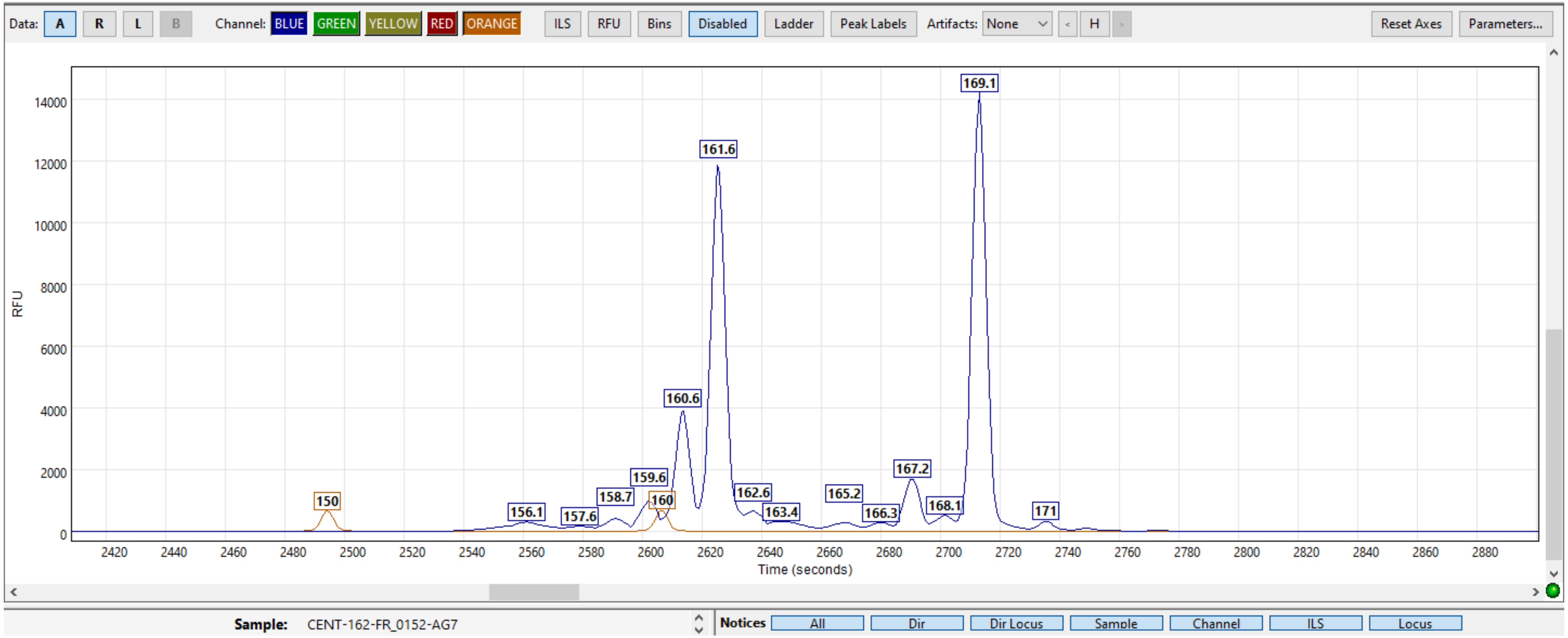


| | |
|---|---|
| **Observer 1** | **162;169** |
| **Observer 2** | **161.6;169.1** |
| **Observer 3** | **162;169** |

## 25- Wild. Locus AG7 sample GZGA10 (0153)

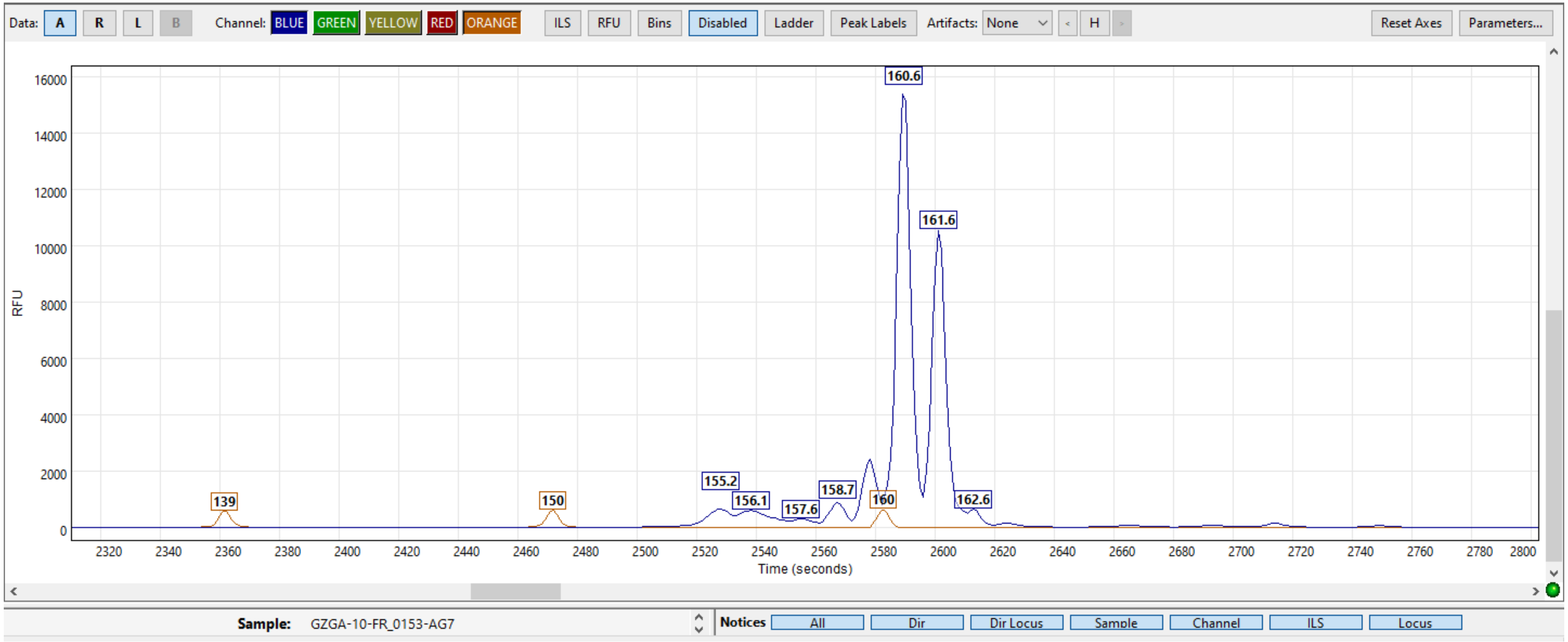


| | |
|---|---|
| **Observer 1** | **161** |
| **Observer 2** | **160.6** |
| **Observer 3** | **161;162** |

**26- Wild. Locus AG7 sample GZGA8 (0154)**

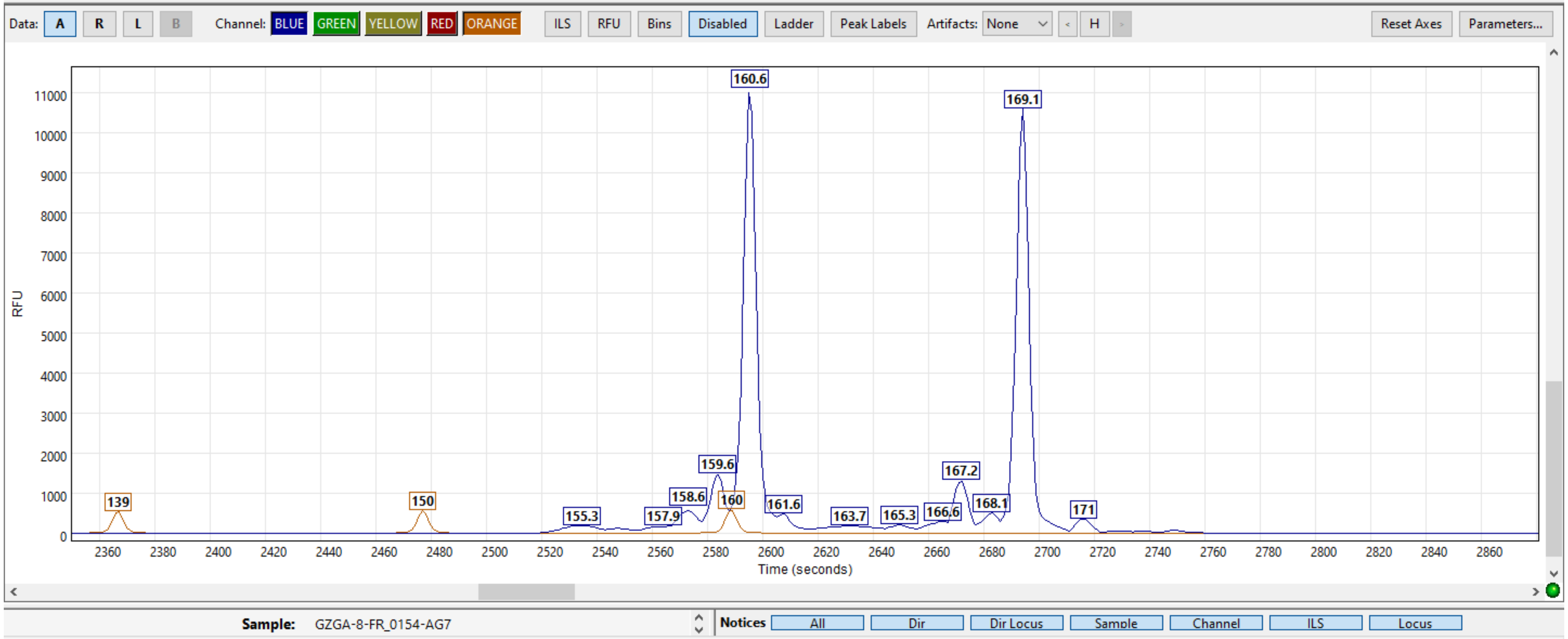


| | |
|---|---|
| **Observer 1** | **161;169** |
| **Observer 2** | **160.6;169.1** |
| **Observer 3** | **161;169** |

**27- Wild. Locus AG7 sample GZGA9 (0155)**

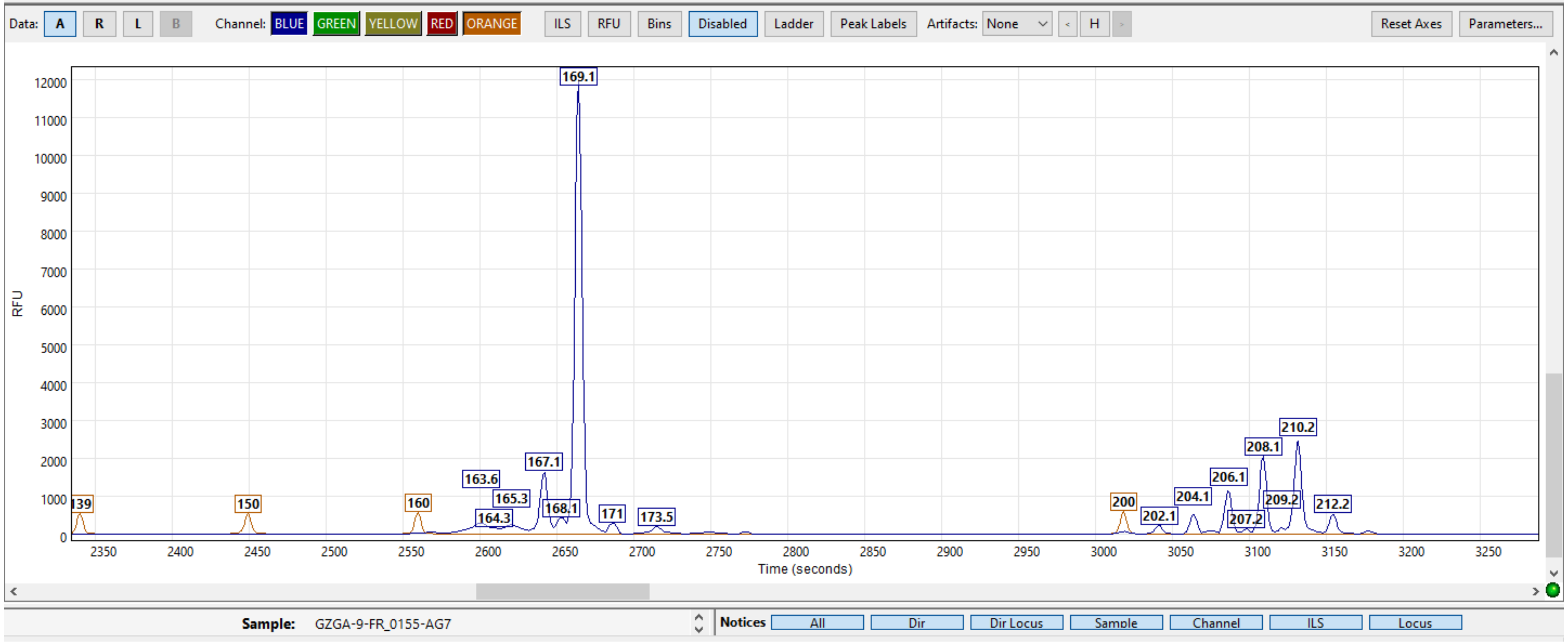


| | |
|---|---|
| **Observer 1** | **169;210** |
| **Observer 2** | **169.1 ; 210.2** |
| **Observer 3** | **169;210** |

**28- Wild. Locus AG7 sample MACC3 (0156)**

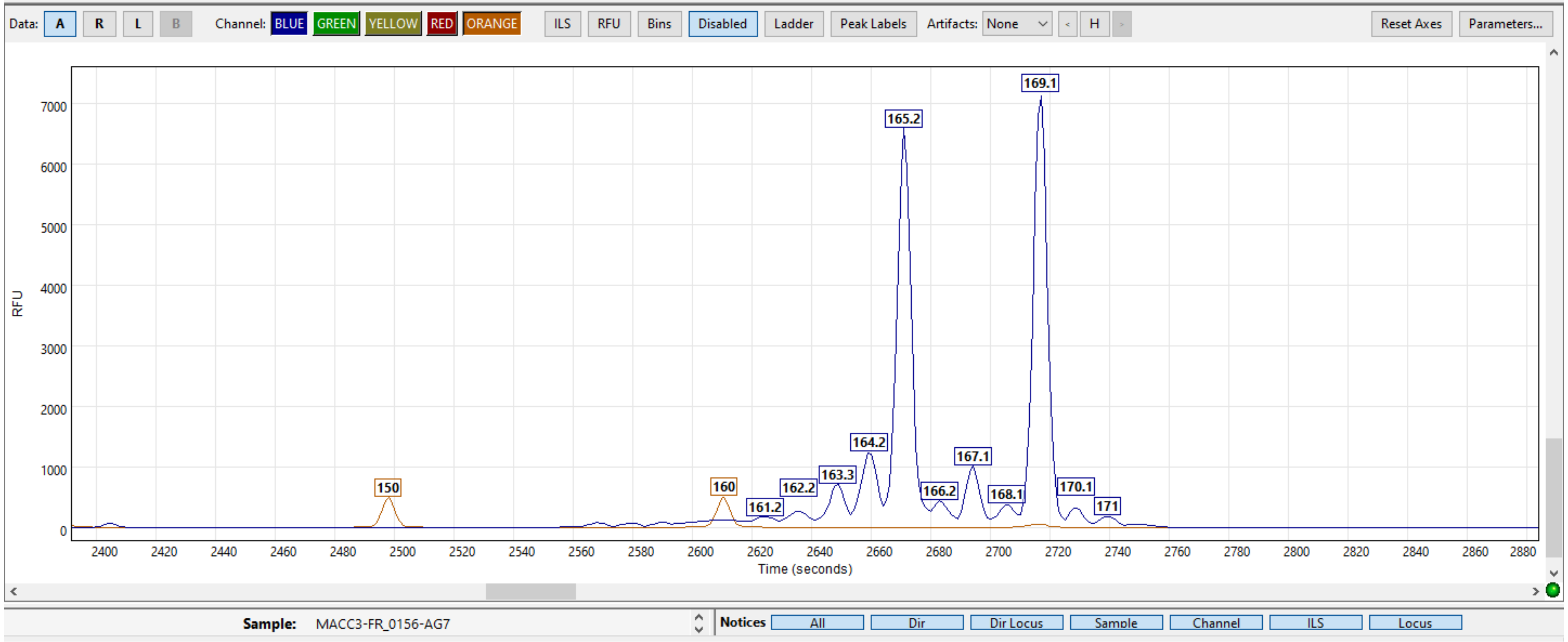


| | |
|---|---|
| **Observer 1** | **165;169** |
| **Observer 2** | **165.2;169.1** |
| **Observer 3** | **165;169** |

## 29- Wild. Locus AG7 sample PONT92 (0157)

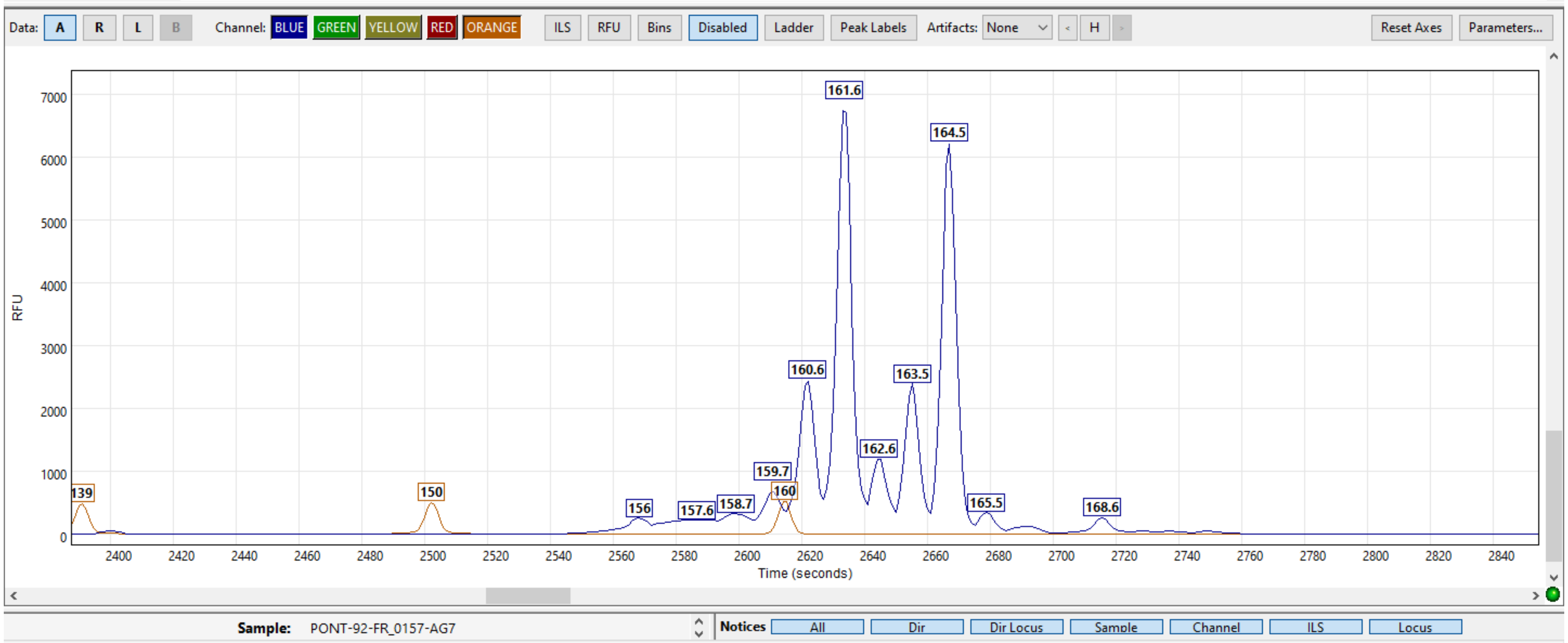


| | |
|---|---|
| **Observer 1** | **162;164** |
| **Observer 2** | **161.6;164.5** |
| **Observer 3** | **162;165** |

### 30- Wild. Locus AG7 sample PONT95 (0158)

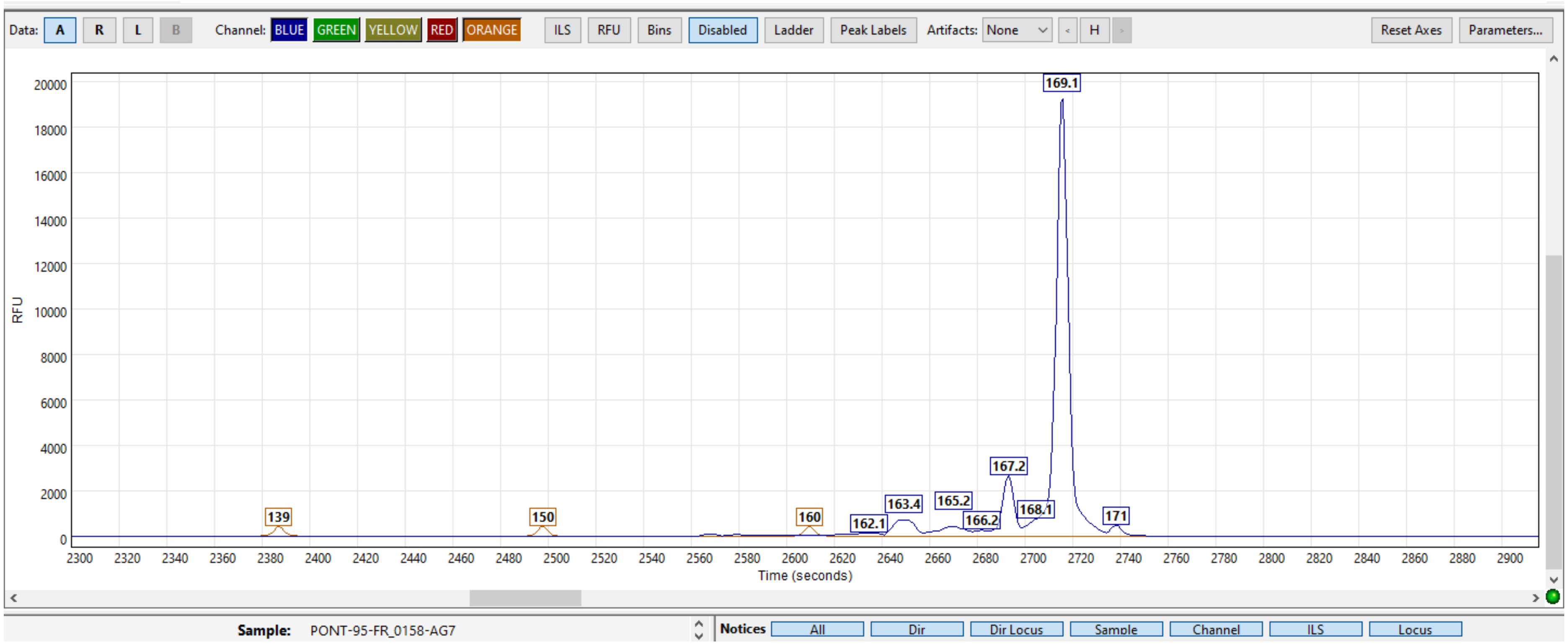


| | |
|---|---|
| **Observer 1** | **167;169** |
| **Observer 2** | **169.1** |
| **Observer 3** | **169** |

## AT1

### 1- Wild. Locus AT1 sample 01 (0909)

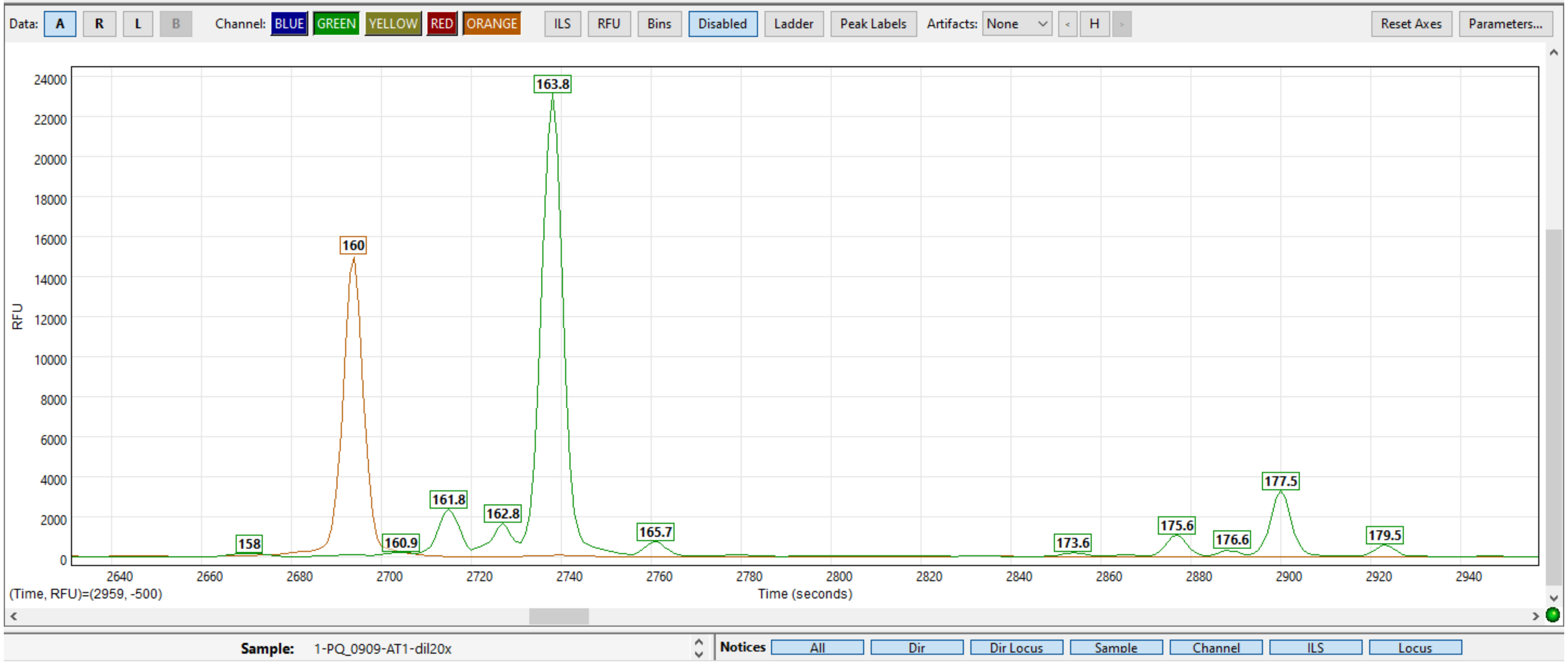


| | |
|---|---|
| **Observer 1** | **164;177** |
| **Observer 2** | **163.8** |
| **Observer 3** | **164** |

**2- Wild. Locus AT1 sample 03 (0910)**

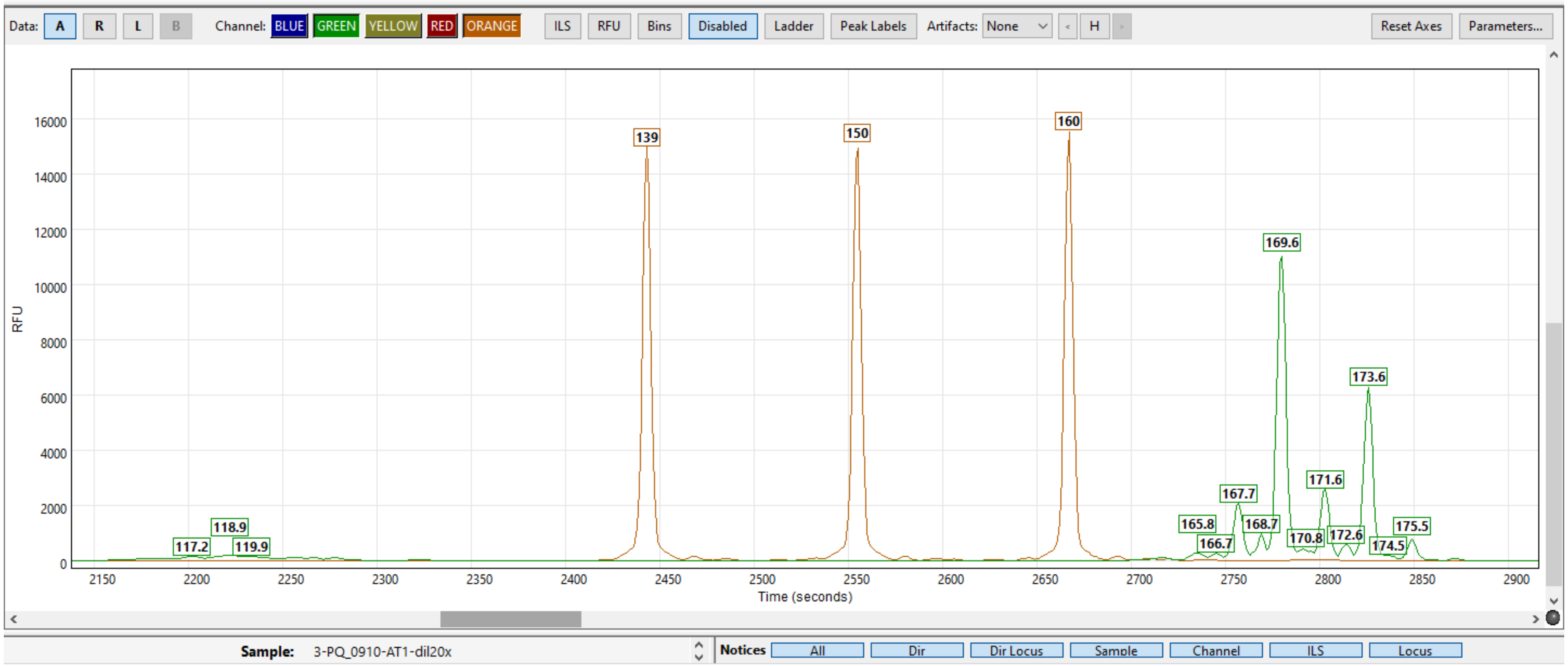


| | |
|---|---|
| **Observer 1** | **170;174** |
| **Observer 2** | **169.6;173.6** |
| **Observer 3** | **170;174** |

### 3- Wild. Locus AT1 sample 05 (0911)

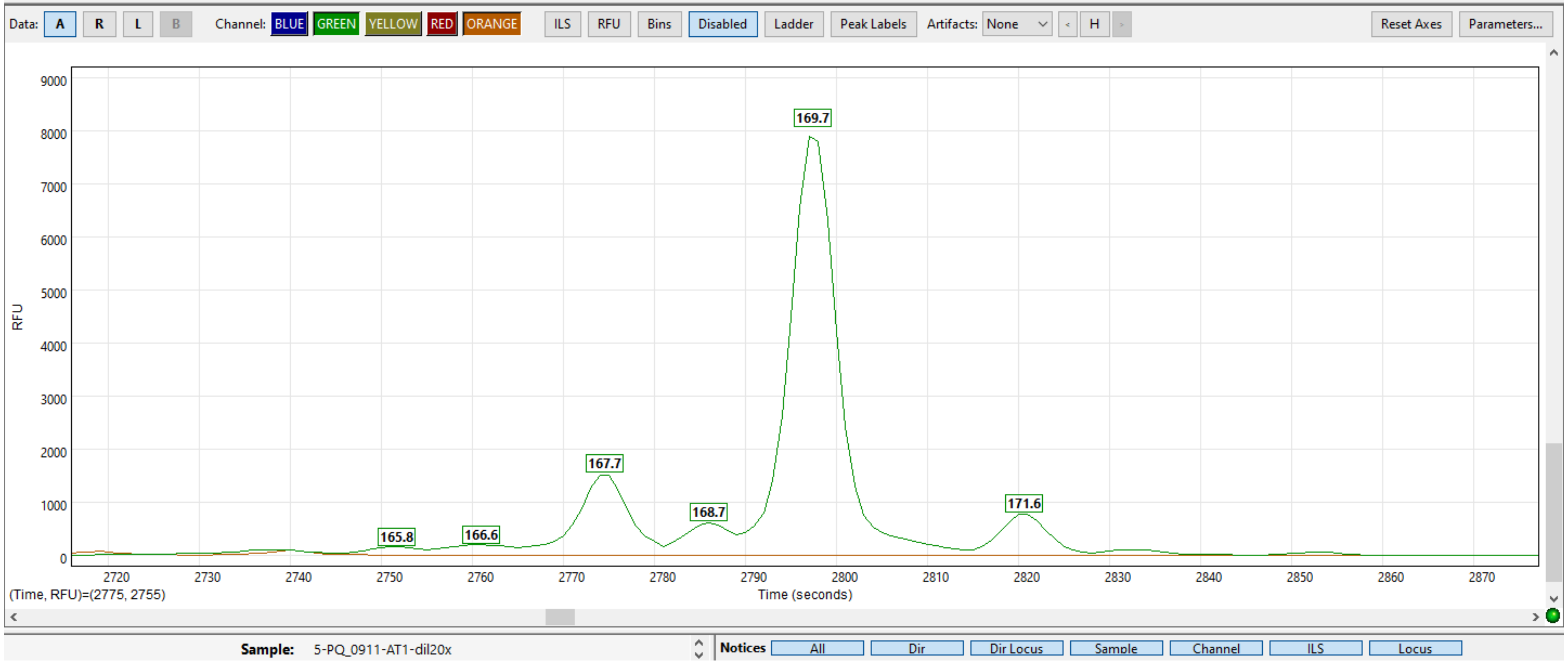


| | |
|---|---|
| **Observer 1** | **170** |
| **Observer 2** | **169.7** |
| **Observer 3** | **170** |

## 4- Wild. Locus AT1 sample 07 (0912)

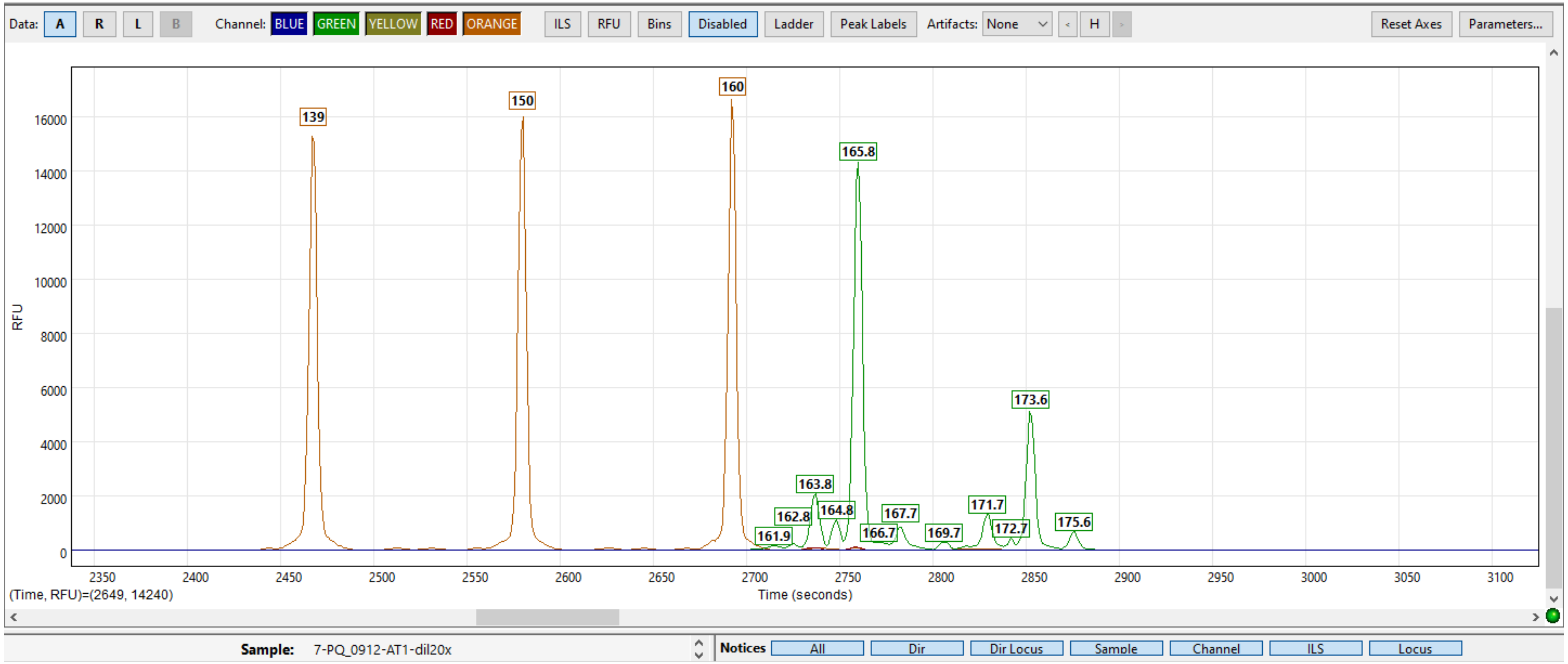


| | |
|---|---|
| **Observer 1** | **166;174** |
| **Observer 2** | **165.8;173.6** |
| **Observer 3** | **166;174** |

## 5- Wild. Locus AT1 sample 08 (0913)

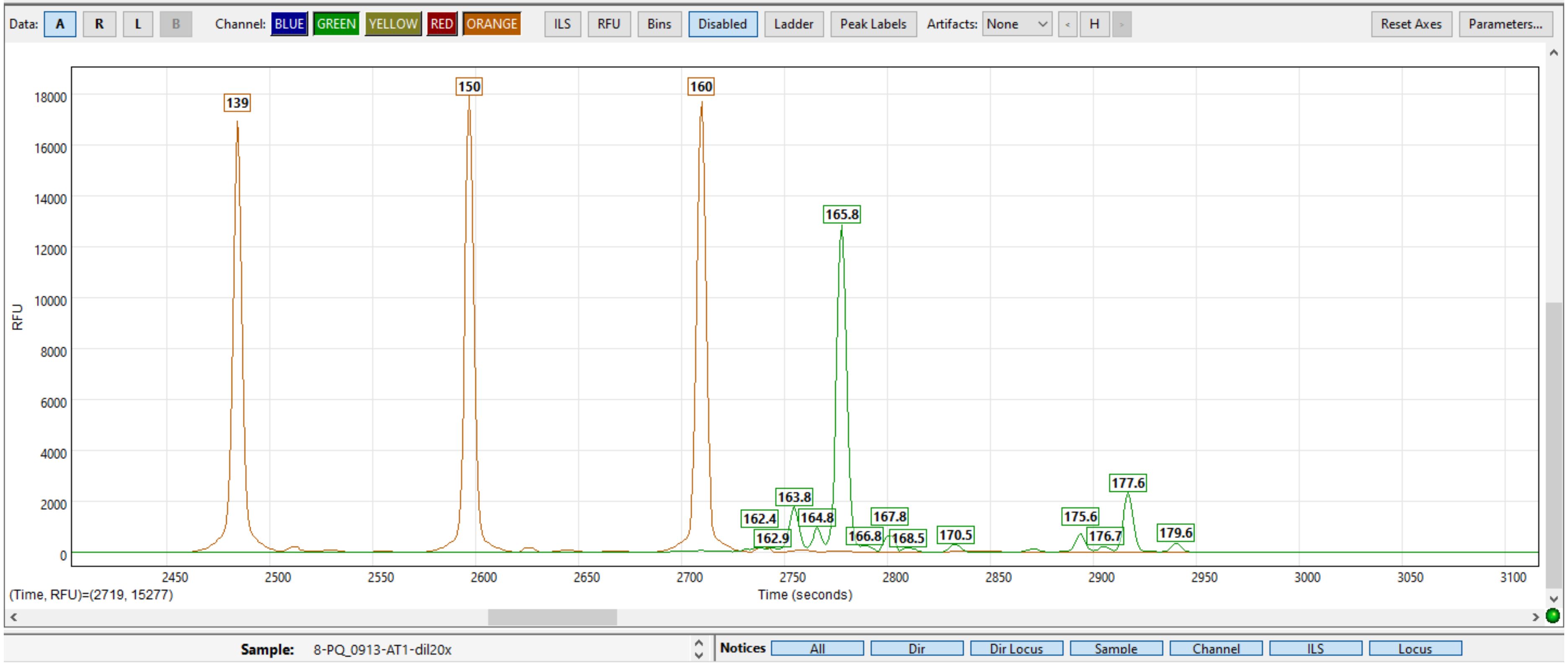


| | |
|---|---|
| **Observer 1** | **166;178** |
| **Observer 2** | **165.8;177.6** |
| **Observer 3** | **166;178** |

## 6- Wild. Locus AT1 sample 09 (0914)

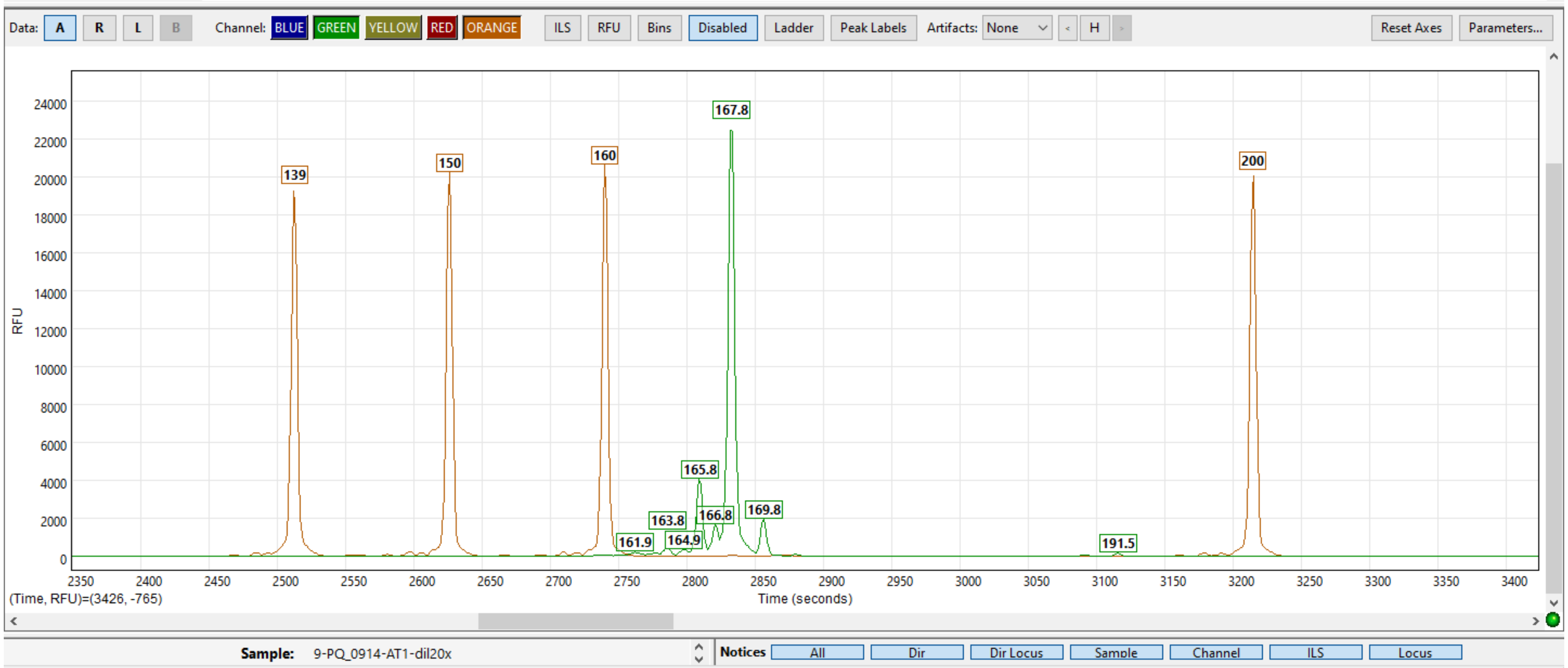


| | |
|---|---|
| **Observer 1** | **166;168** |
| **Observer 2** | **165.8 ;167.8** |
| **Observer 3** | **168** |

## 7- Wild. Locus AT1 sample 12 (0915)

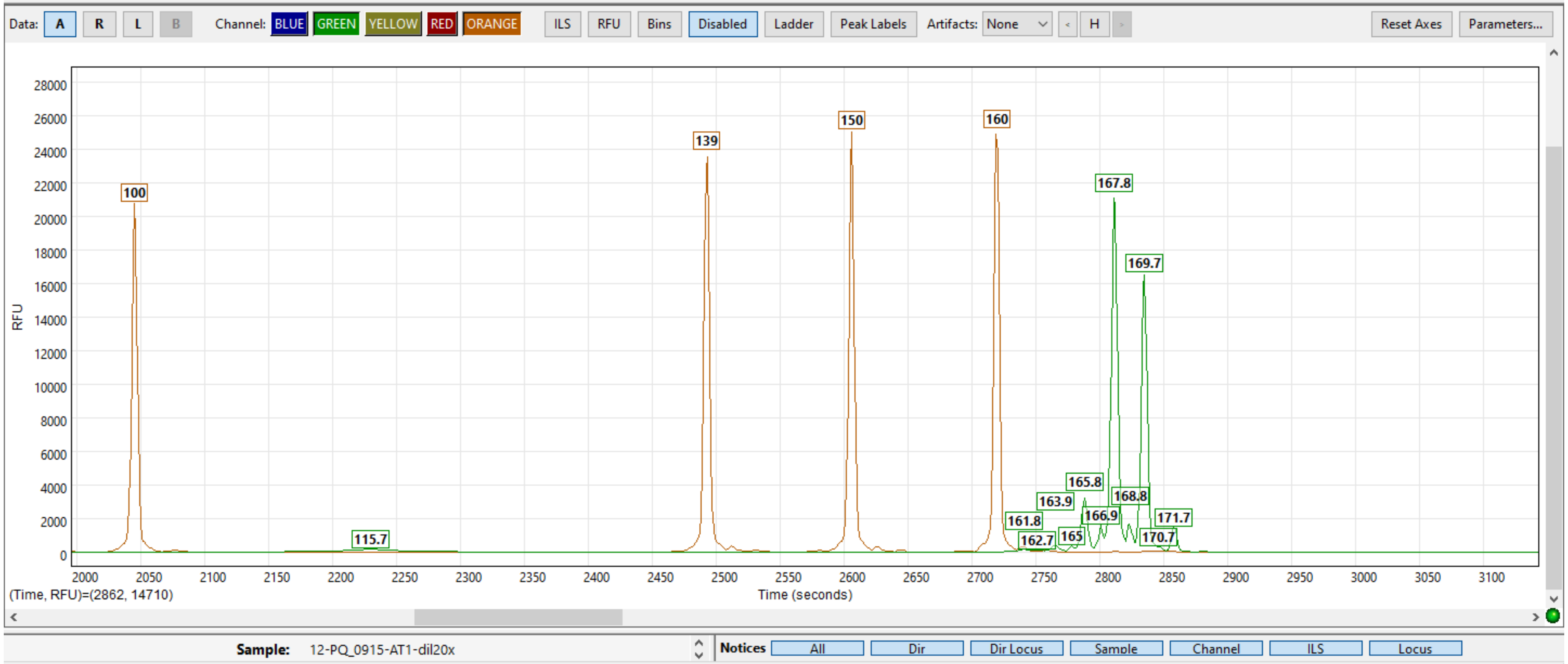


| | |
|---|---|
| **Observer 1** | **168;170** |
| **Observer 2** | **167.8;169.7** |
| **Observer 3** | **168;170** |

**8- Wild. Locus AT1 sample 13 (0916)**

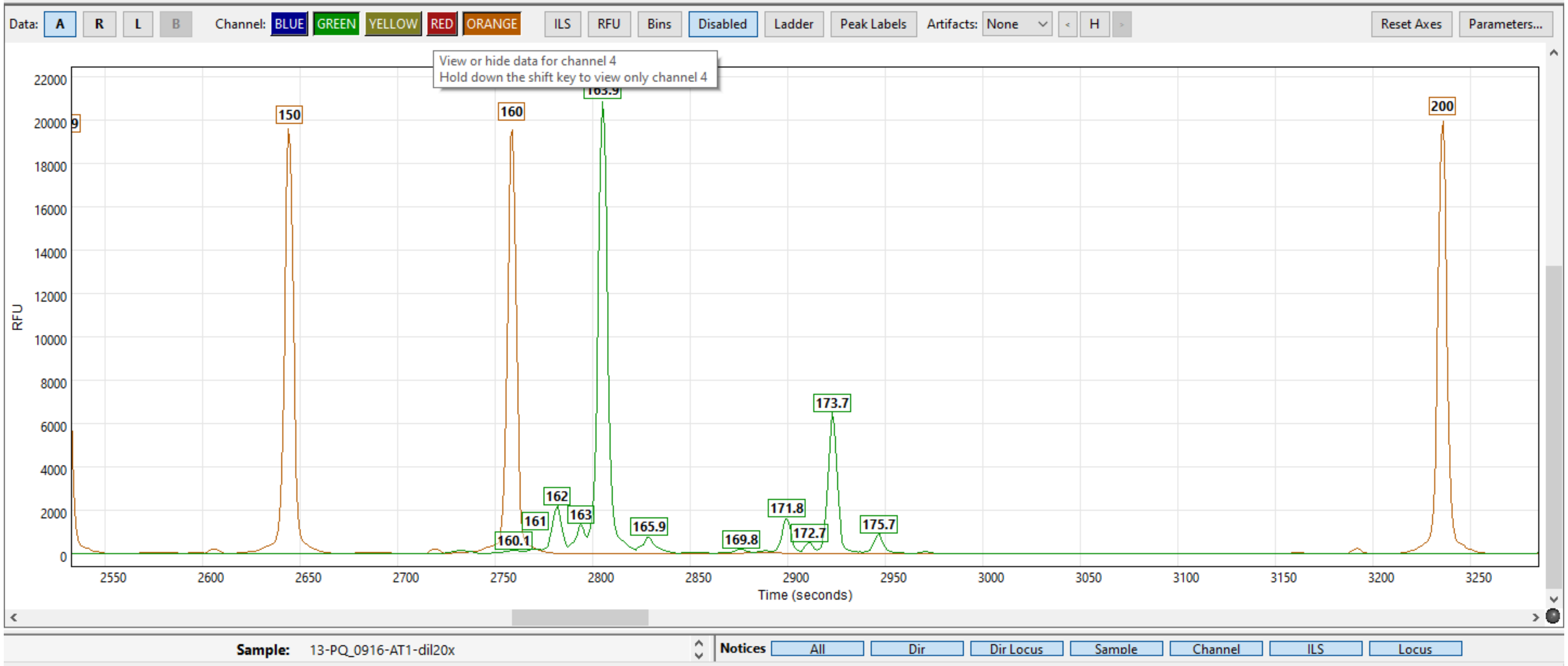


| | |
|---|---|
| **Observer 1** | **164;174** |
| **Observer 2** | **163.9;173.7** |
| **Observer 3** | **164;174** |

## 9- Wild. Locus AT1 sample 14 (0917)

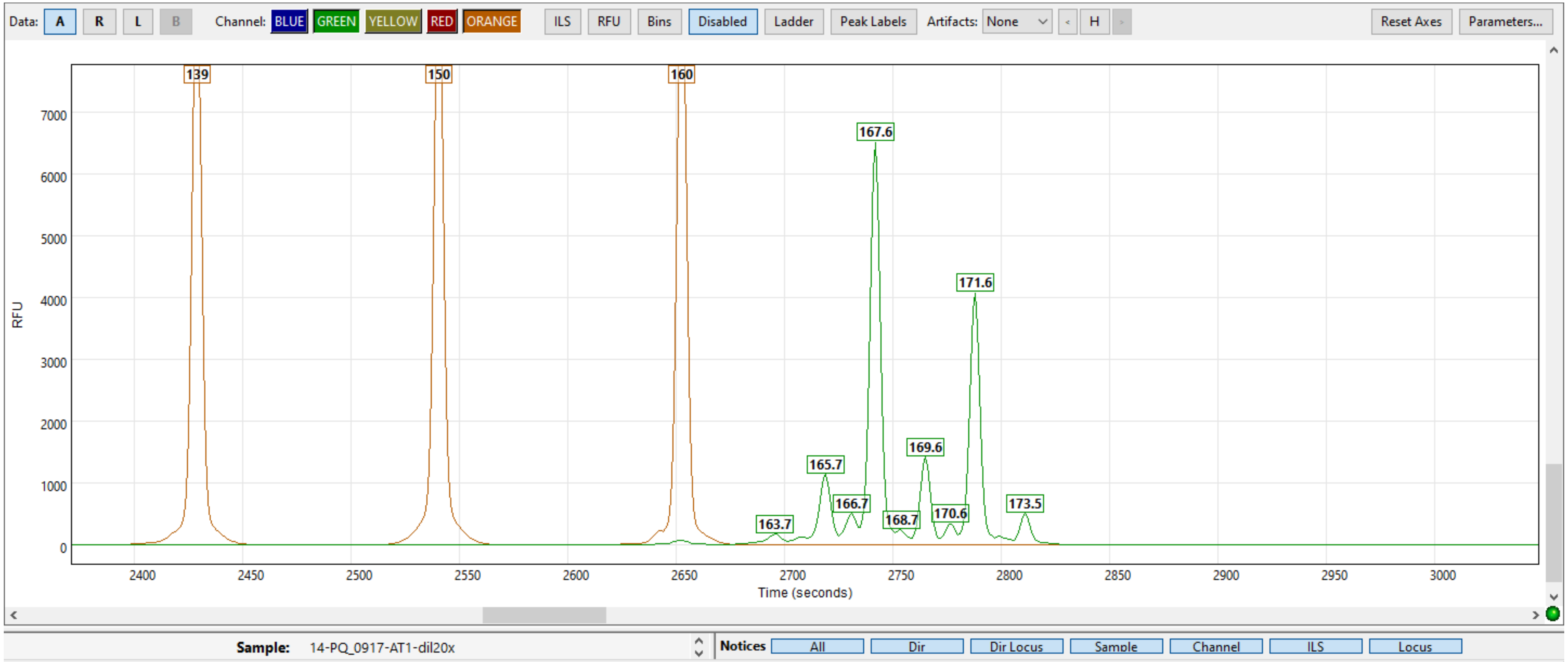


| | |
|---|---|
| **Observer 1** | **168;172** |
| **Observer 2** | **167.6;171.6** |
| **Observer 3** | **168;172** |

## 10- Wild. Locus AT1 sample 15 (0918)

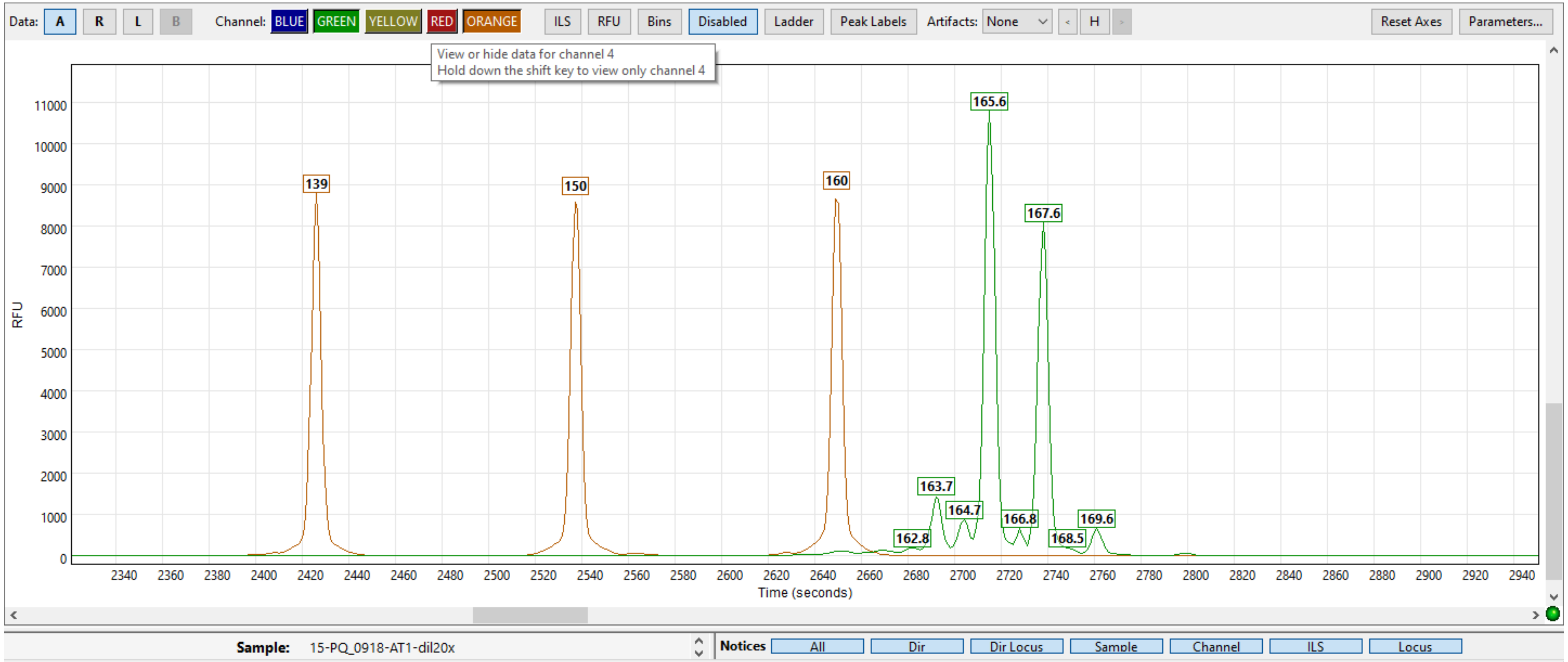


| | |
|---|---|
| **Observer 1** | **166;168** |
| **Observer 2** | **165.6;167.6** |
| **Observer 3** | **166;168** |

**11- Wild. Locus AT1 sample 16 (0919)**

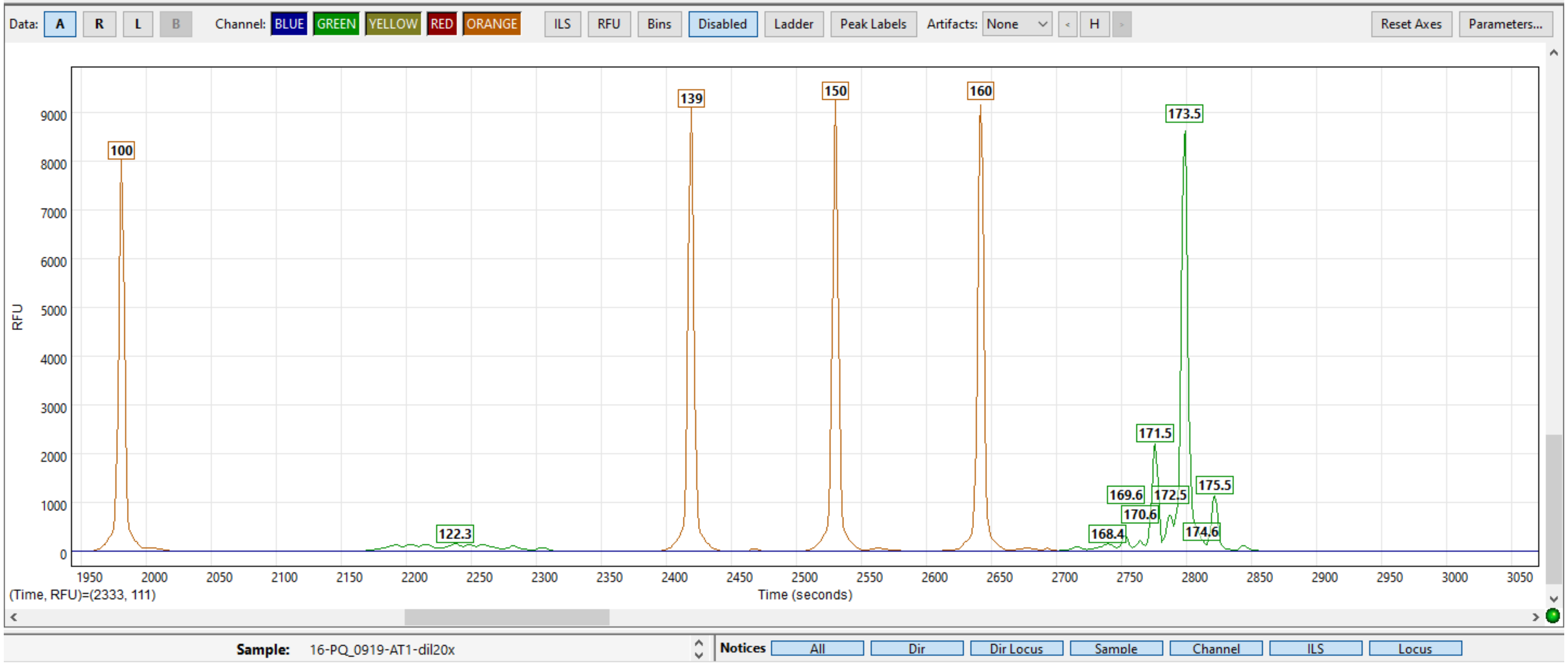


| | |
|---|---|
| **Observer 1** | **173** |
| **Observer 2** | **173.5** |
| **Observer 3** | **173** |

**12- Wild. Locus AT1 sample 17 (0920)**

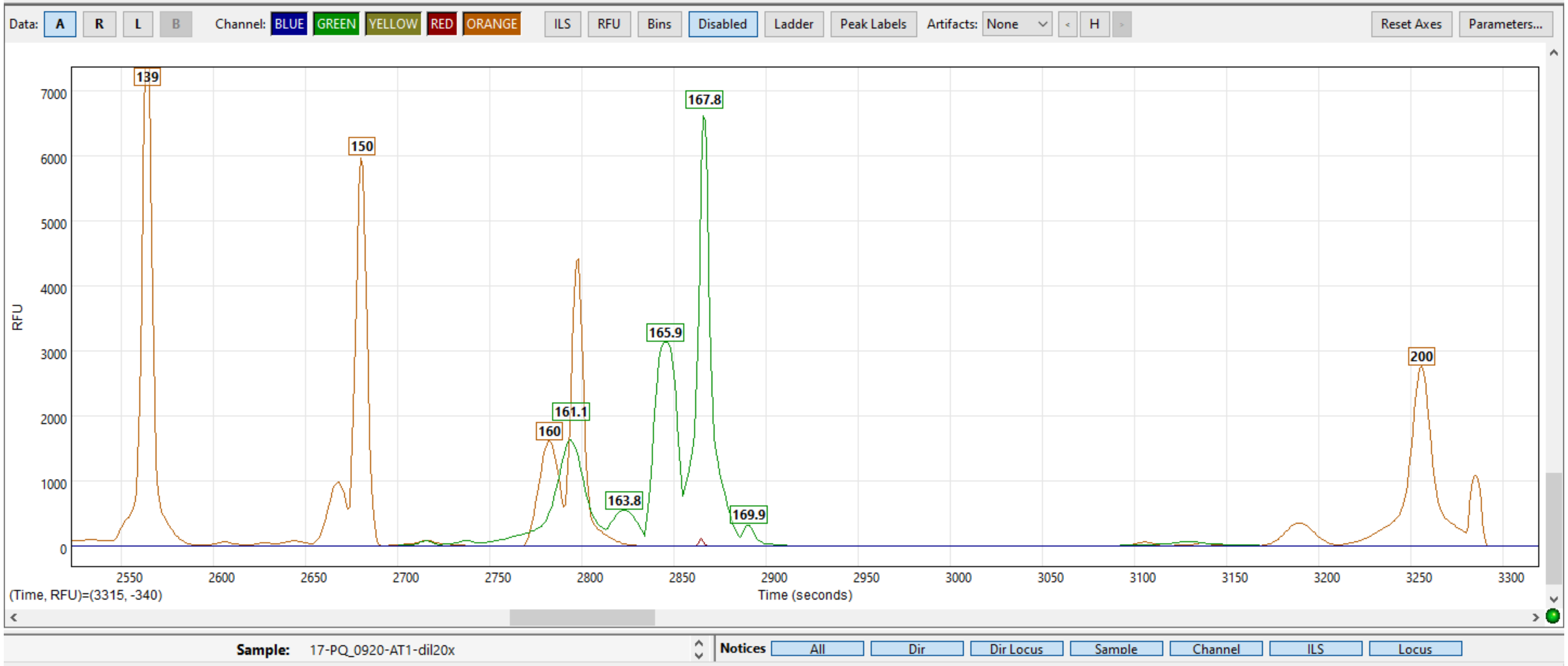


| | |
|---|---|
| **Observer 1** | **168** |
| **Observer 2** | **167.8** |
| **Observer 3** | **166;168** |

**13- Wild. Locus AT1 sample 19 (0921)**

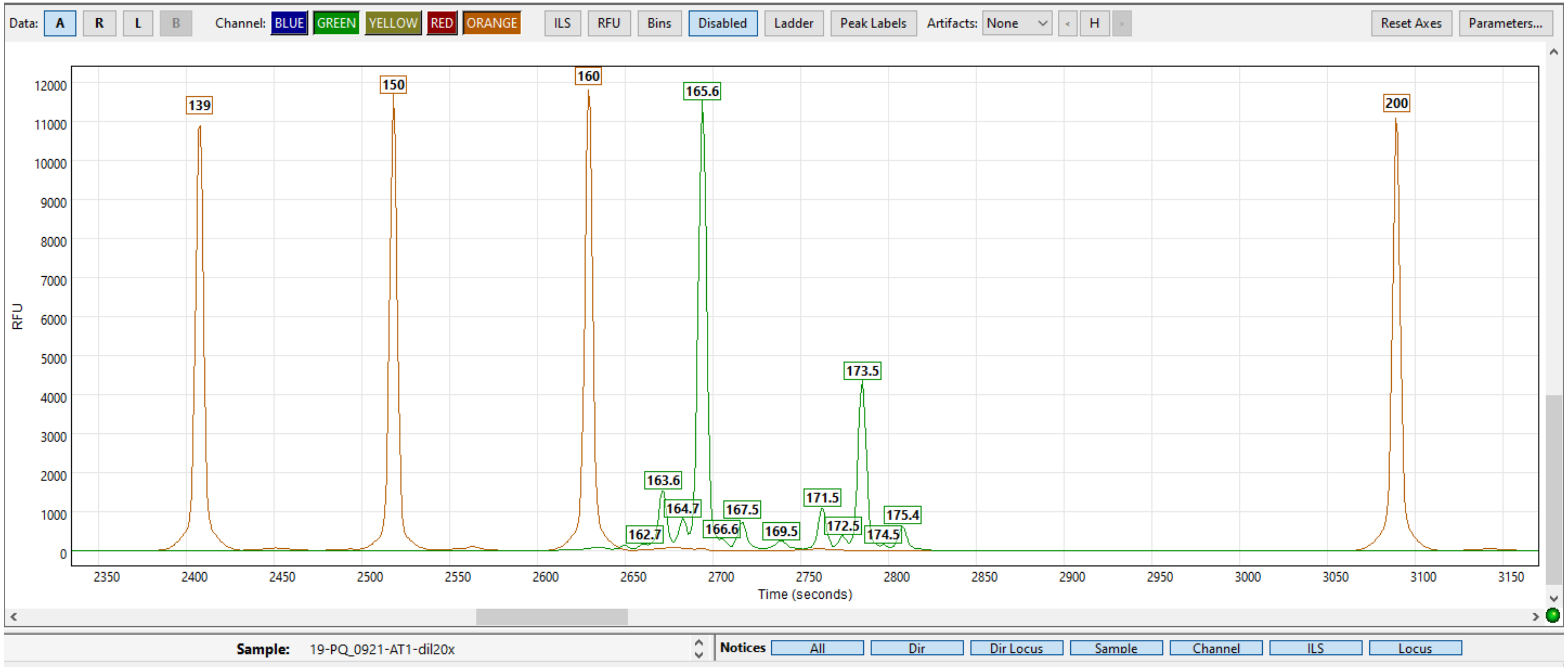


| | |
|---|---|
| **Observer 1** | **166;173** |
| **Observer 2** | **165.6;173.5** |
| **Observer 3** | **166;173** |

**14- Wild. Locus AT1 sample 20 (0922)**

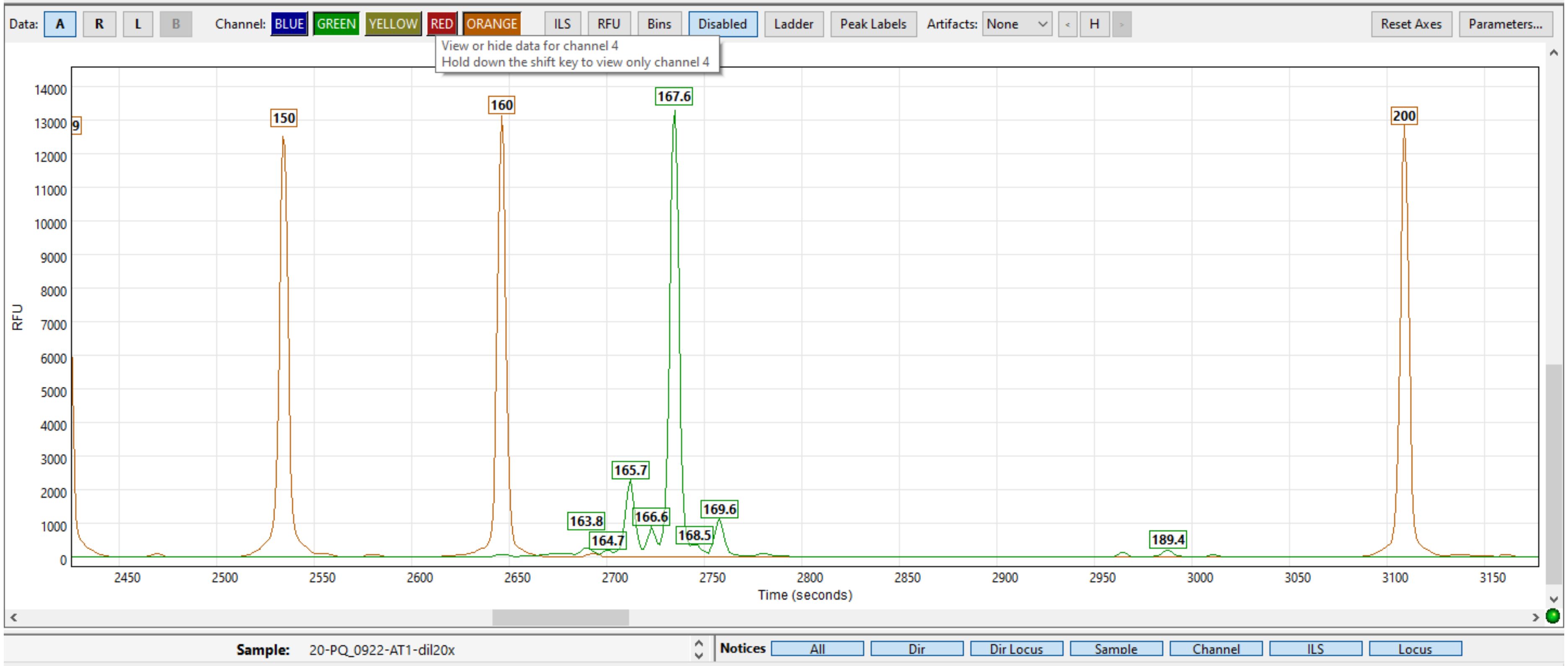


| | |
|---|---|
| **Observer 1** | **168** |
| **Observer 2** | **167.6** |
| **Observer 3** | **168** |

# 15- Wild. Locus AT1 sample 21 (0924)

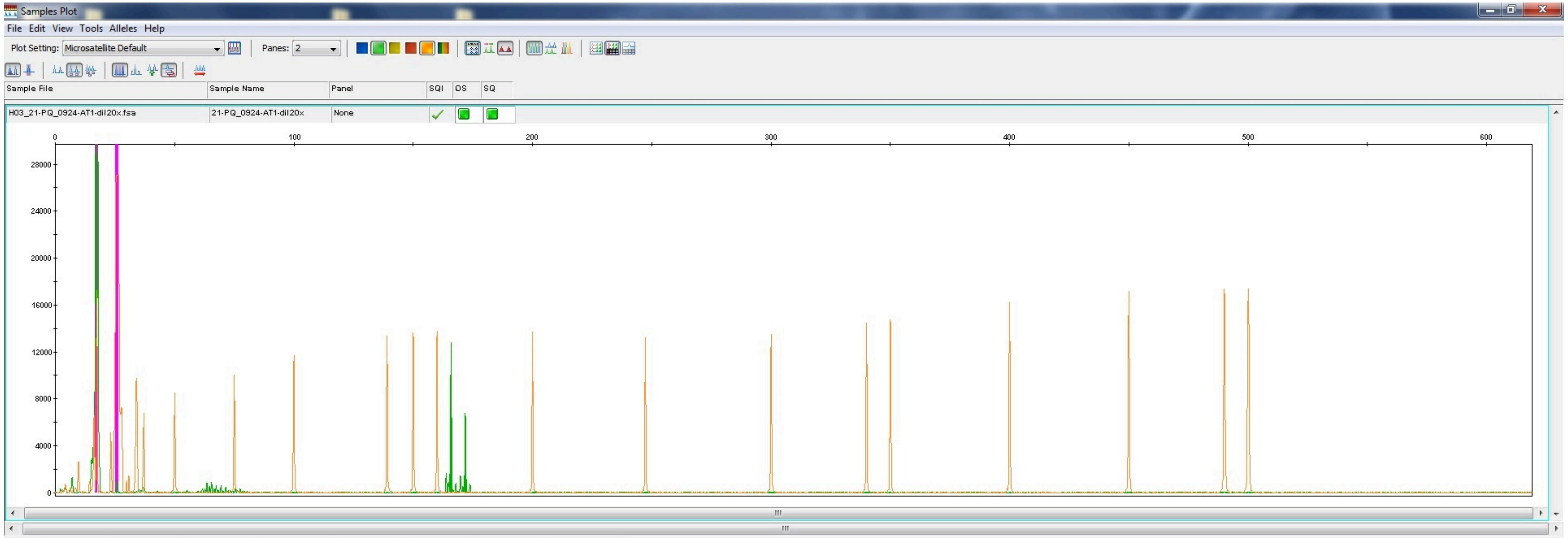


| Dye/Sample Peak | Sample File Name | Marker | Allele | Size | Height | Area | Data Point |
|---|---|---|---|---|---|---|---|
| G,99 | H03_21-PQ_0924 | | | 165.82 | 12773 | 70650 | 2744 |
| G,100 | H03_21-PQ_0924 | | | 165.82 | 12773 | 70650 | 2744 |
| G,101 | H03_21-PQ_0924 | | | 166.78 | 189 | 929 | 2755 |
| G,102 | H03_21-PQ_0924 | | | 166.78 | 189 | 929 | 2755 |
| G,103 | H03_21-PQ_0924 | | | 167.82 | 838 | 3802 | 2767 |
| G,104 | H03_21-PQ_0924 | | | 167.82 | 838 | 3802 | 2767 |
| G,105 | H03_21-PQ_0924 | | | 168.85 | 126 | 446 | 2779 |
| G,106 | H03_21-PQ_0924 | | | 168.85 | 126 | 446 | 2779 |
| G,107 | H03_21-PQ_0924 | | | 169.81 | 1443 | 7568 | 2790 |
| G,108 | H03_21-PQ_0924 | | | 169.81 | 1443 | 7568 | 2790 |
| G,109 | H03_21-PQ_0924 | | | 170.84 | 513 | 2673 | 2802 |
| G,110 | H03_21-PQ_0924 | | | 170.84 | 513 | 2673 | 2802 |
| G,111 | H03_21-PQ_0924 | | | 171.79 | 6782 | 36601 | 2813 |
| G,112 | H03_21-PQ_0924 | | | 171.79 | 6782 | 36601 | 2813 |
| G,113 | H03_21-PQ_0924 | | | 172.91 | 70 | 270 | 2826 |
| G,114 | H03_21-PQ_0924 | | | 172.91 | 70 | 270 | 2826 |

| | |
|---|---|
| Observer 1 | 166;172 |
| Observer 2 | 165.8;171.8 |
| Observer 3 | 166;172 |

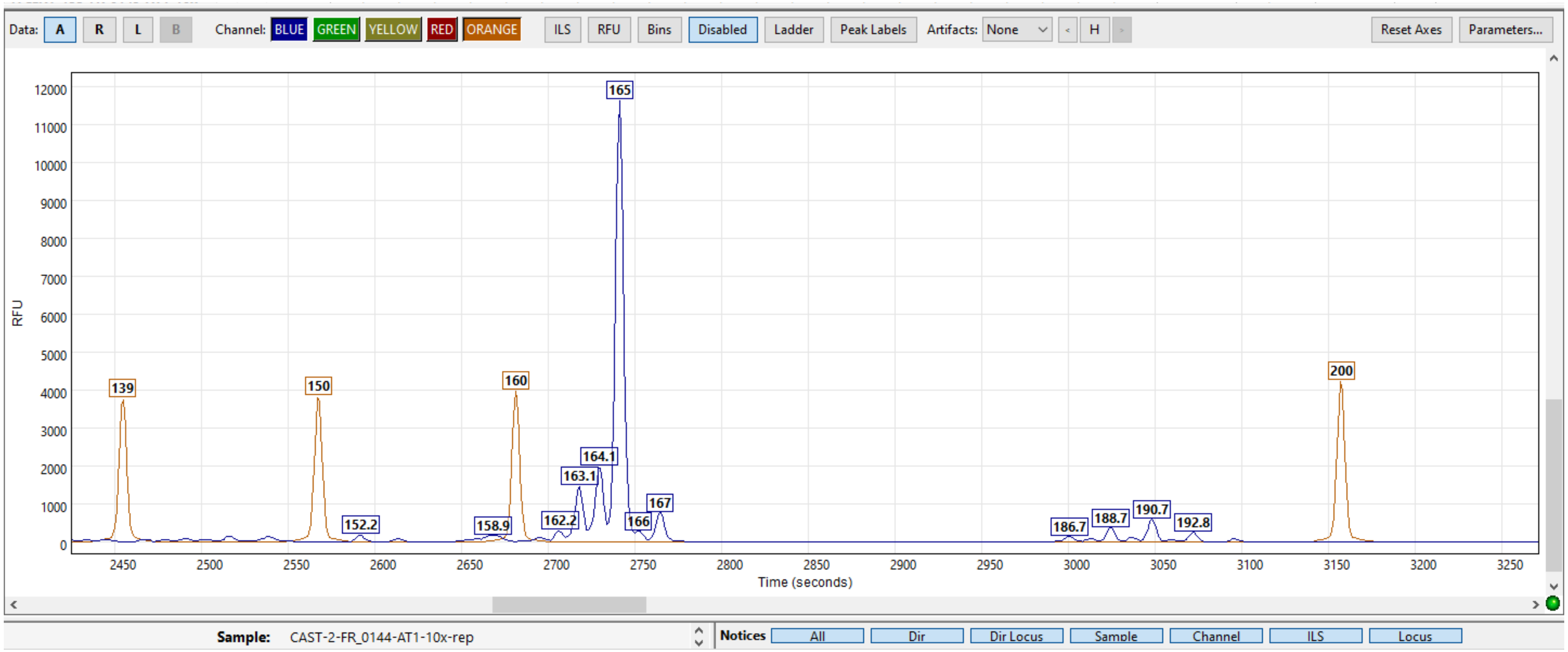


| | |
|---|---|
| **Observer 1** | **165;191** |
| **Observer 2** | **165** |
| **Observer 3** | **165** |

## 17- Wild. Locus AT1 sample CAST3 (0145)

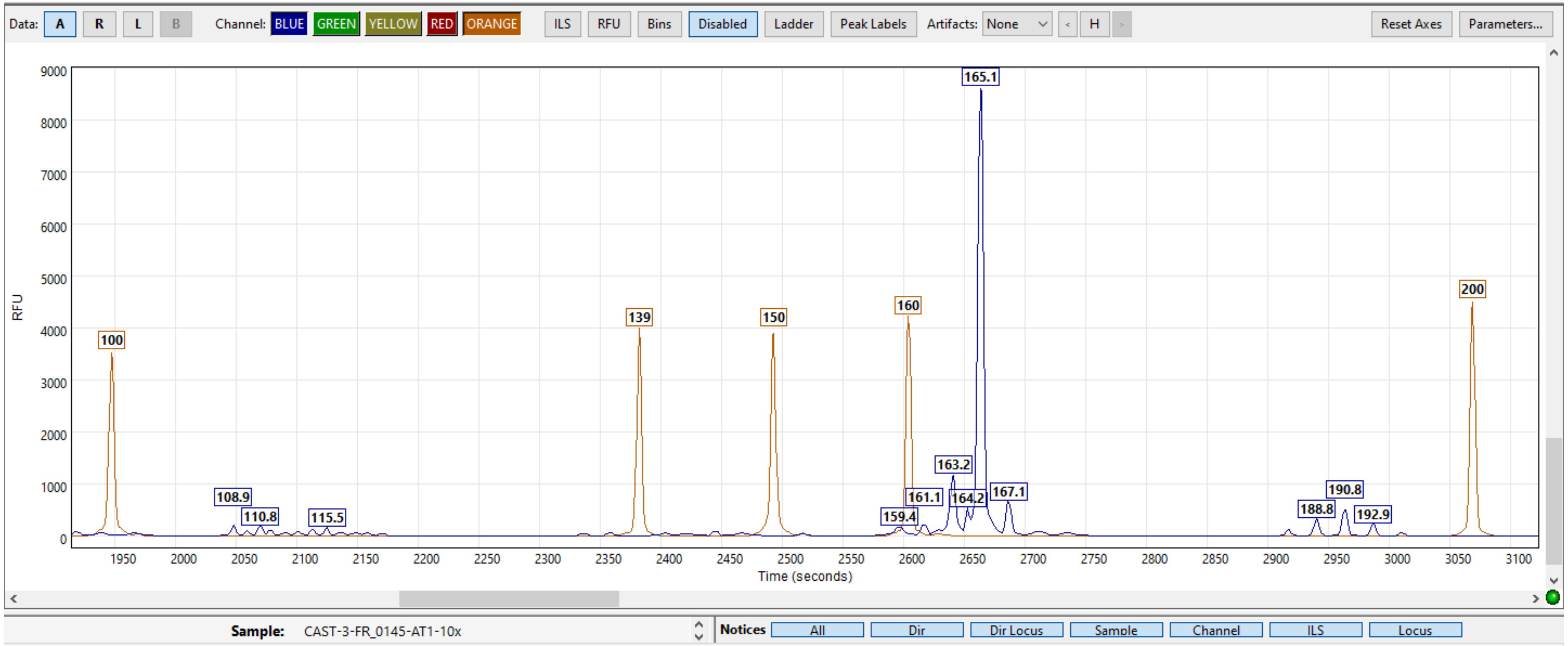


| | |
|---|---|
| **Observer 1** | **165;191** |
| **Observer 2** | **165.1** |
| **Observer 3** | **165** |

## 18- Wild. Locus AT1 sample CENT146 (0146)

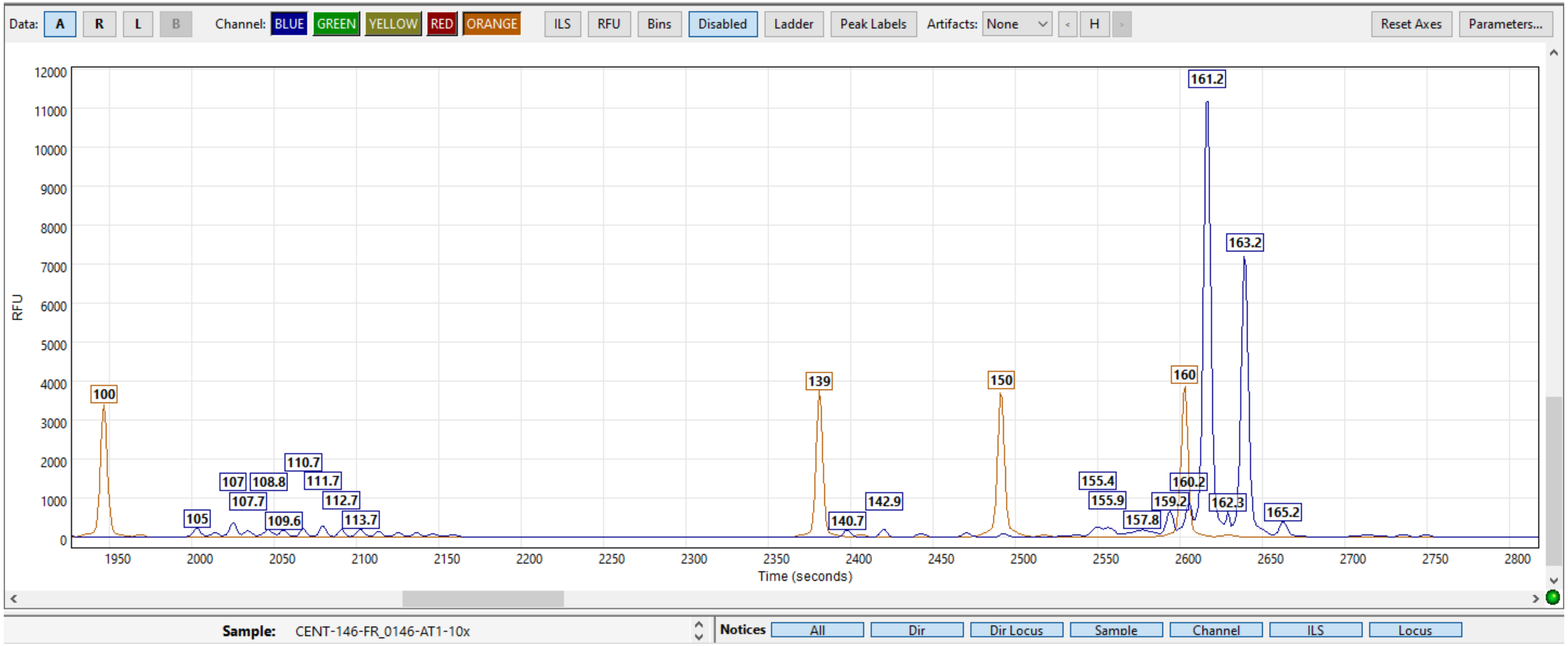


| | |
|---|---|
| **Observer 1** | **161;163** |
| **Observer 2** | **161.2;163.2** |
| **Observer 3** | **161.2;163.2** |

## 19- Wild. Locus AT1 sample CENT151 (0147)

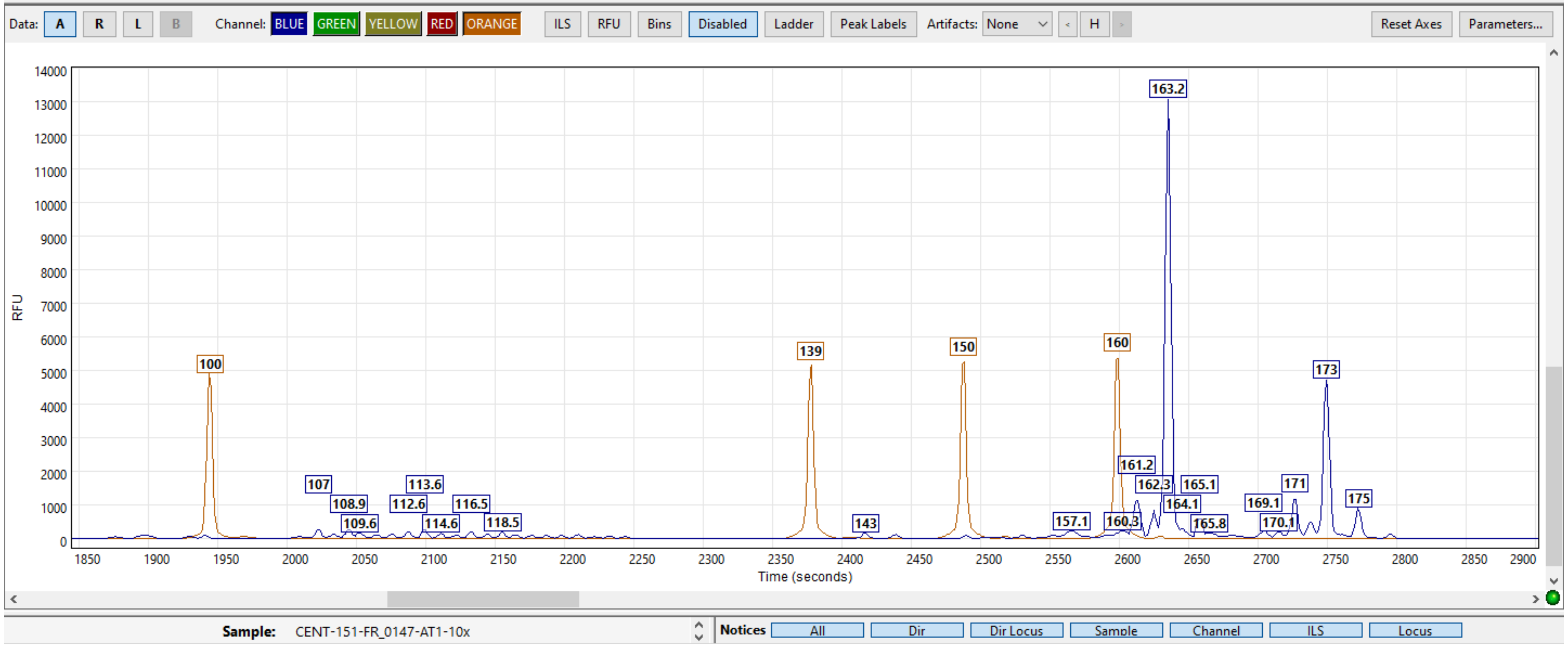


| | |
|---|---|
| **Observer 1** | **163;173** |
| **Observer 2** | **163.2;173** |
| **Observer 3** | **163;173** |

## 20- Wild. Locus AT1 sample CENT152 (0148)

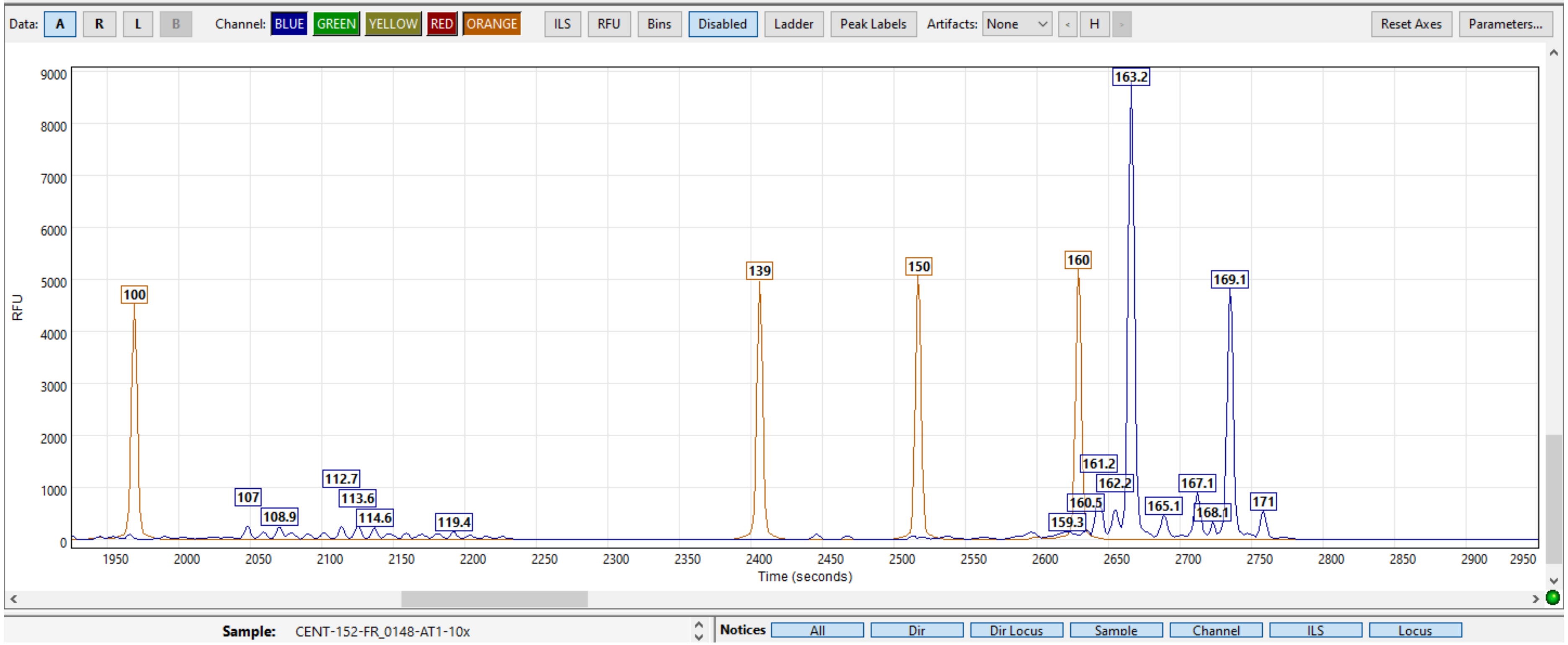


| | |
|---|---|
| **Observer 1** | **163;169** |
| **Observer 2** | **163.2;169.1** |
| **Observer 3** | **163;169** |

## 21- Wild. Locus AT1 sample CENT155 (0149)

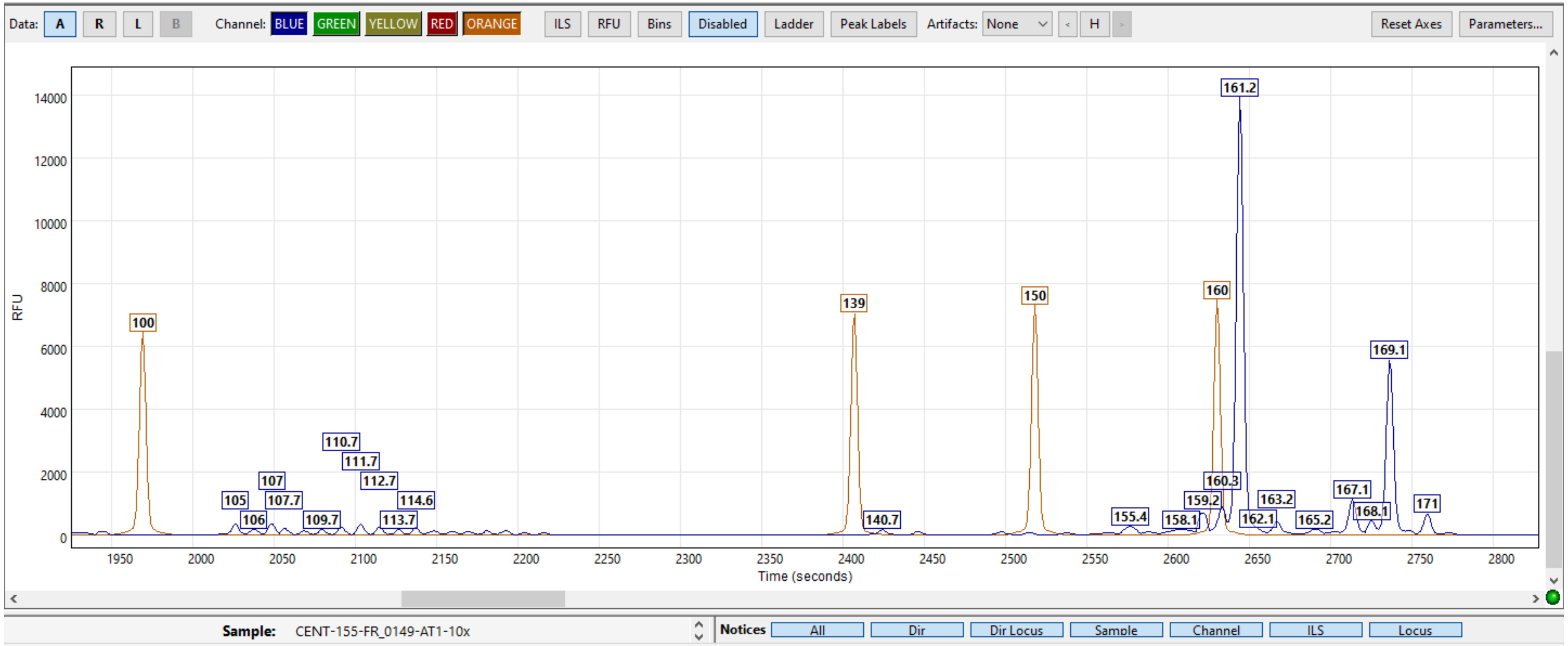


| | |
|---|---|
| **Observer 1** | **161;169** |
| **Observer 2** | **161.2;169.1** |
| **Observer 3** | **161;169** |

**22- Wild. Locus AT1 sample CENT157 (0150)**

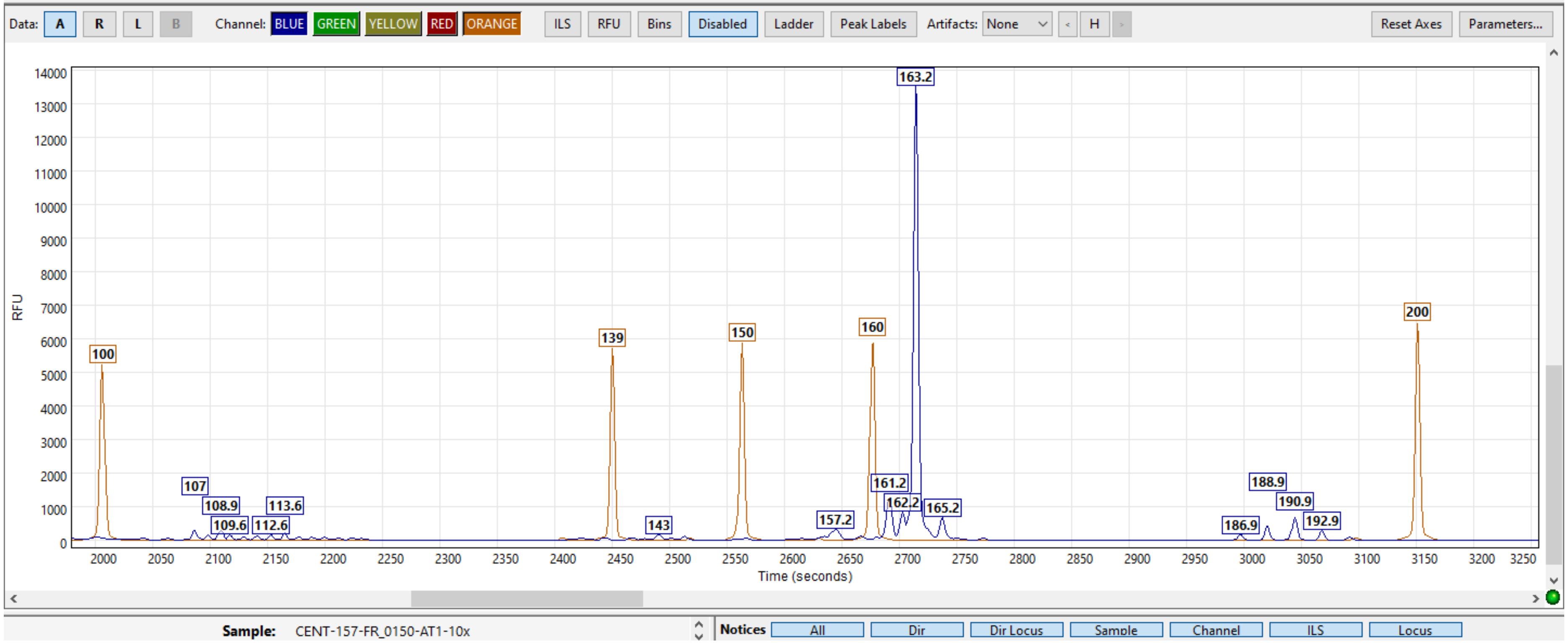


| | |
|---|---|
| **Observer 1** | **163;191** |
| **Observer 2** | **163.2** |
| **Observer 3** | **163.2** |

## 23- Wild. Locus AT1 sample CENT160 (0151)

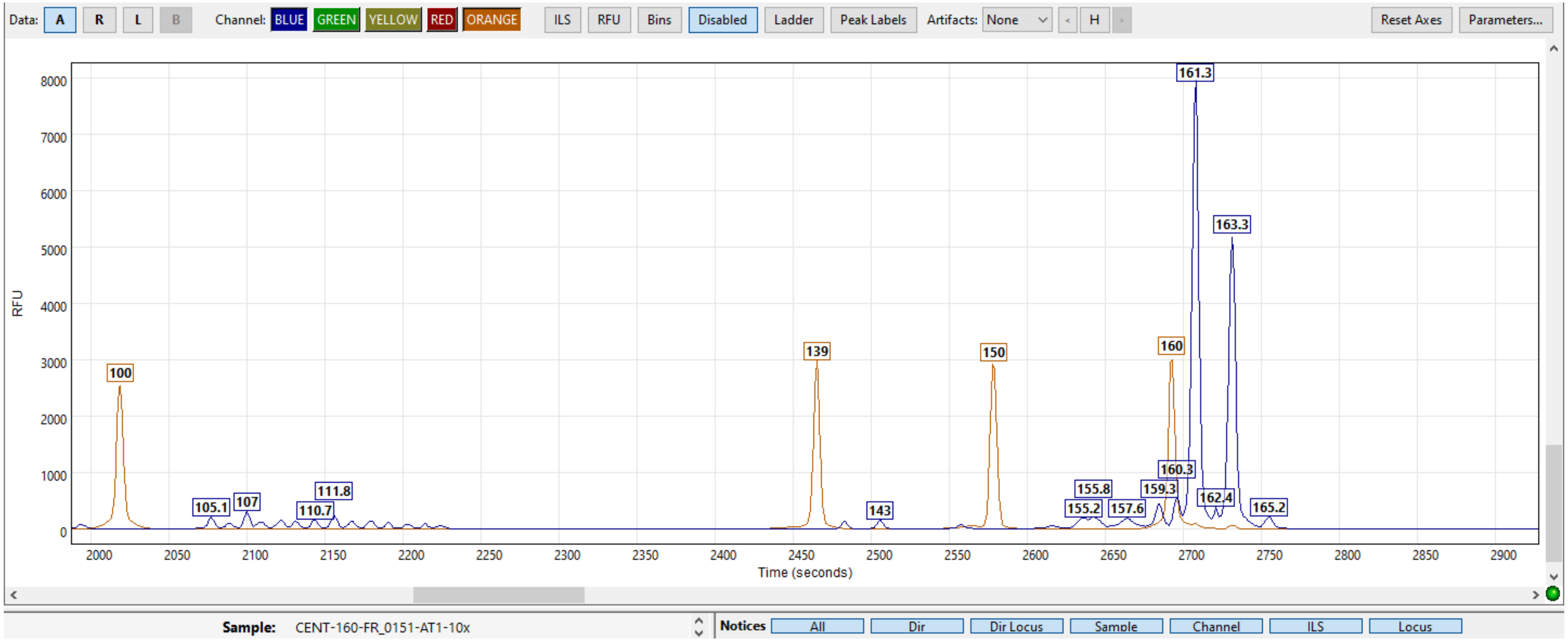


| | |
|---|---|
| **Observer 1** | **161;163** |
| **Observer 2** | **161.3;163.3** |
| **Observer 3** | **161.3;163.3** |

## 24- Wild. Locus AT1 sample CENT162 (0152)

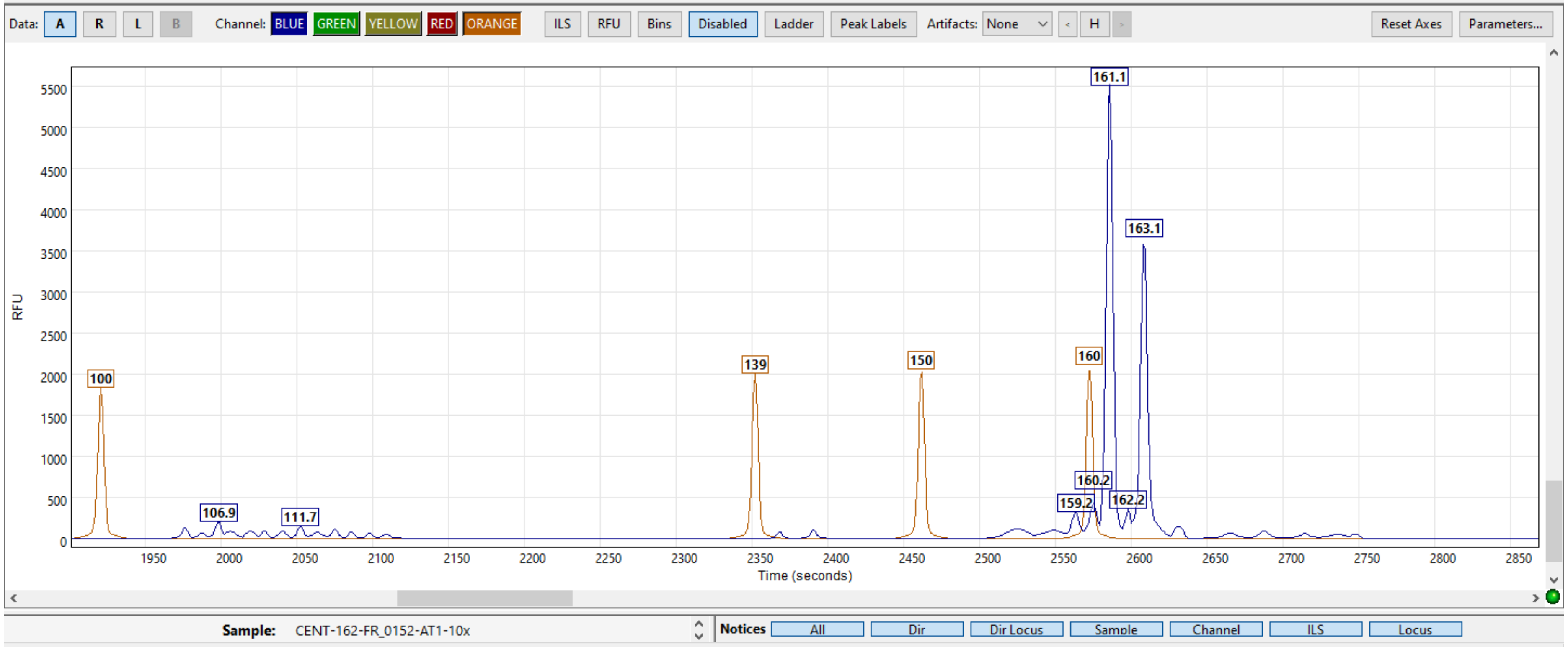


| | |
|---|---|
| **Observer 1** | **161;163** |
| **Observer 2** | **161.1;163.1** |
| **Observer 3** | **161.1;163.1** |

**25- Wild. Locus AT1 sample GZGA10 (0153)**

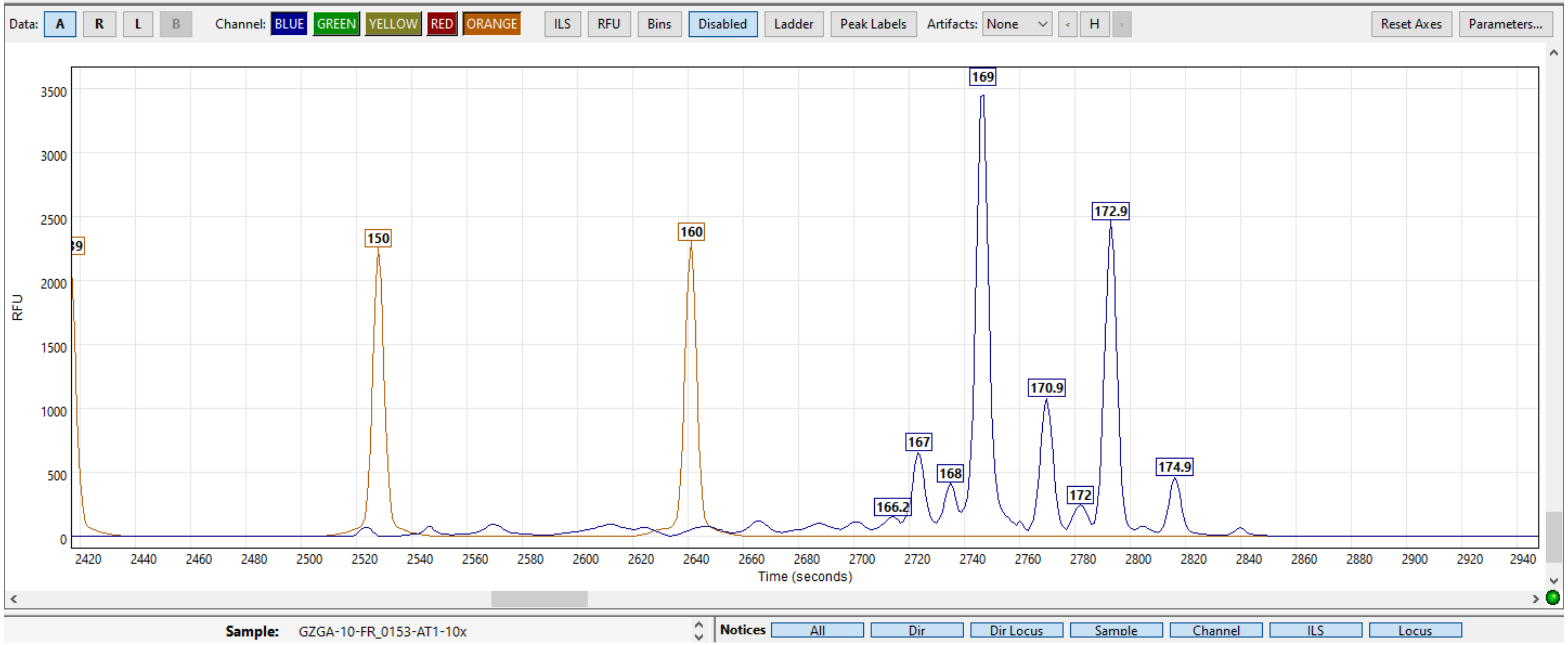


| | |
|---|---|
| **Observer 1** | **169;173** |
| **Observer 2** | **169;172.9** |
| **Observer 3** | **169;172.9** |

## 26- Wild. Locus AT1 sample GZGA8 (0154)

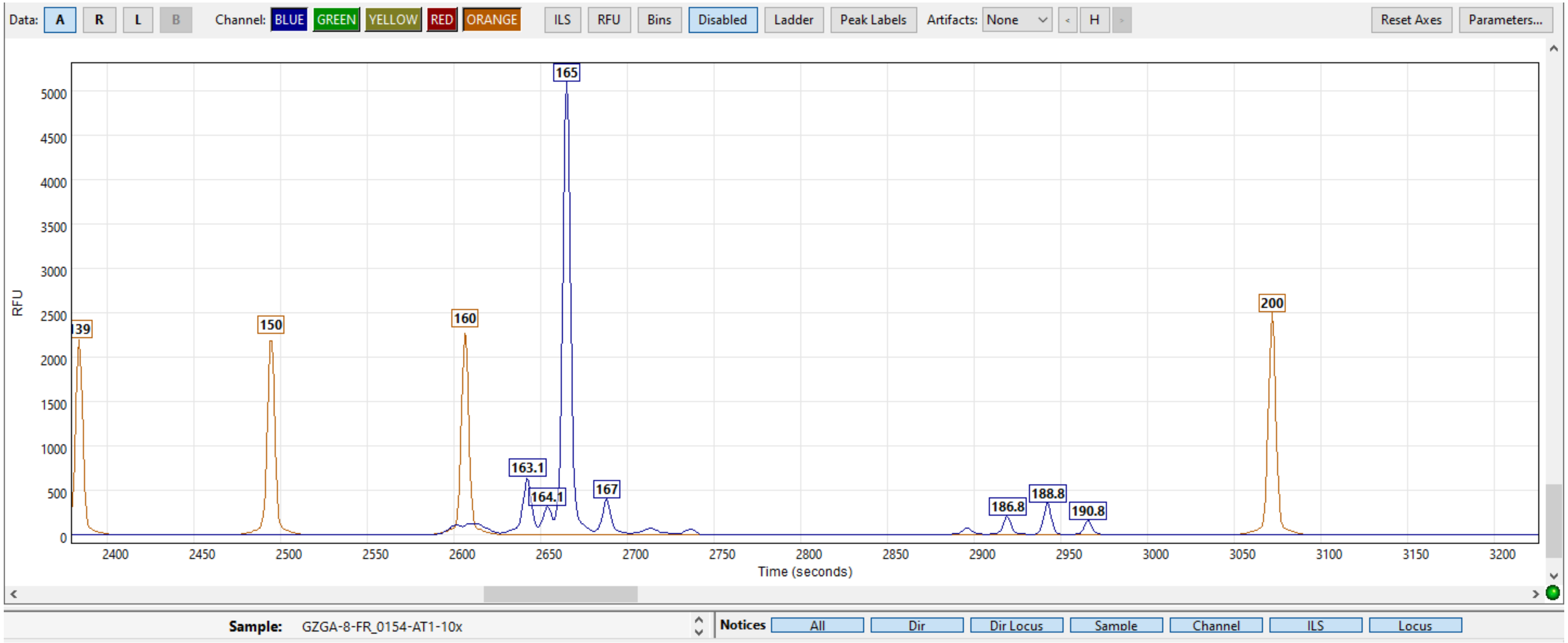


| | |
|---|---|
| **Observer 1** | **165;189** |
| **Observer 2** | **165** |
| **Observer 3** | **165** |

**27- Wild. Locus AT1 sample GZGA9 (0155)**

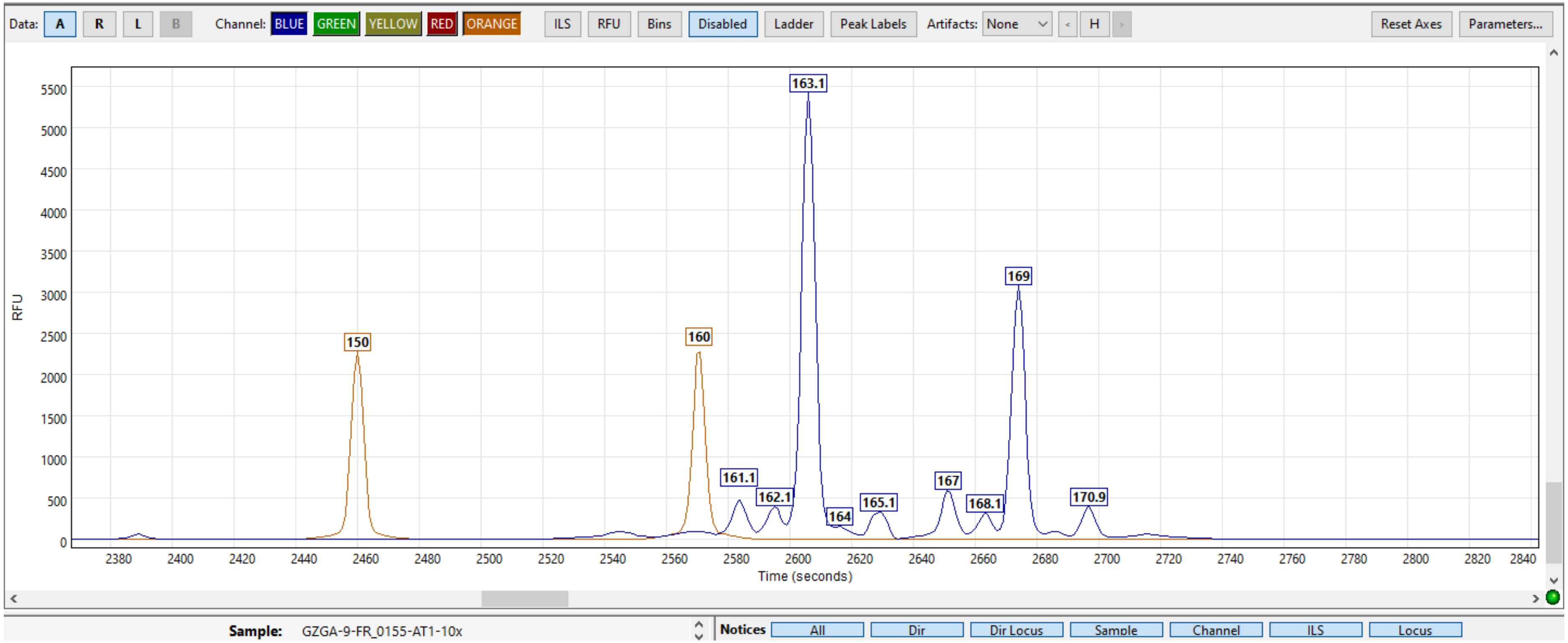


| | |
|---|---|
| **Observer 1** | **163;169** |
| **Observer 2** | **163.1;169** |
| **Observer 3** | **163;169** |

**28- Wild. Locus AT1 sample MACC3 (0156)**

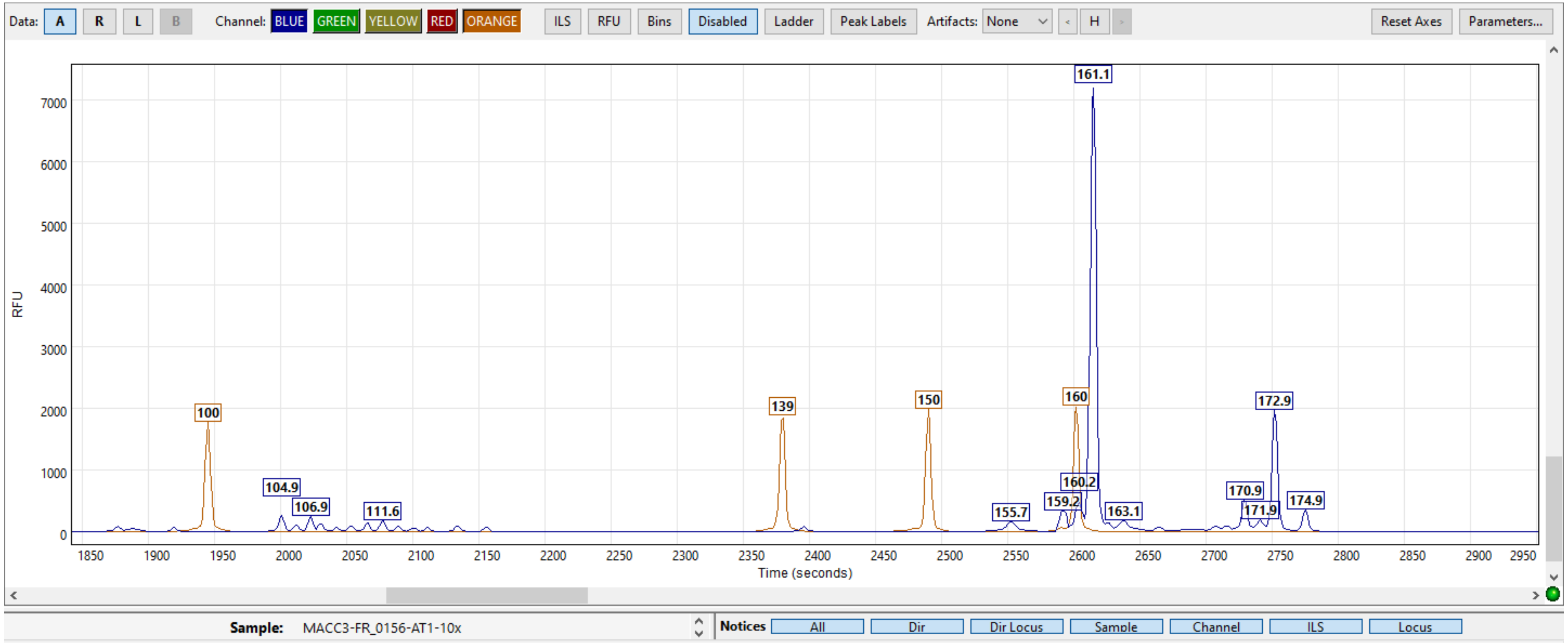


| | |
|---|---|
| **Observer 1** | **161;173** |
| **Observer 2** | **161.1;172.9** |
| **Observer 3** | **161.1;172.9** |

## 29- Wild. Locus AT1 sample PONT92 (0157)

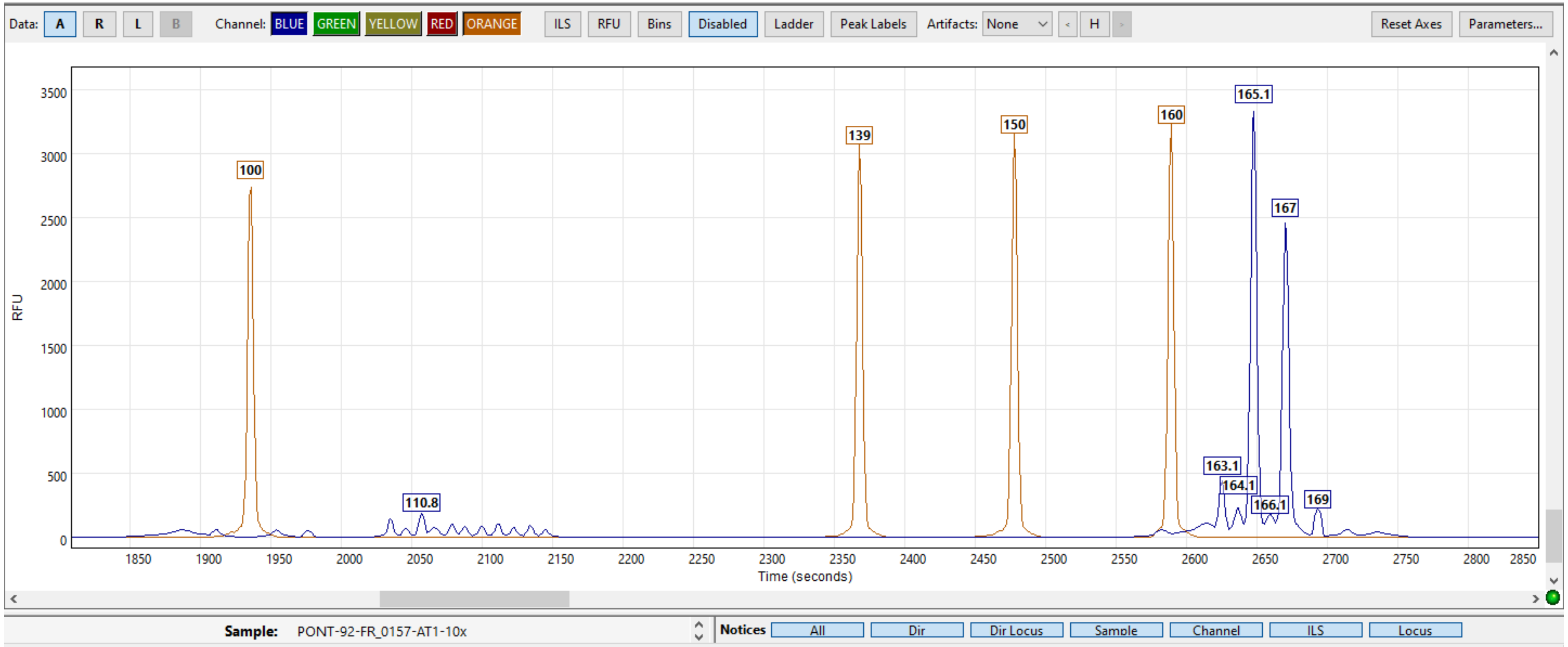


| | |
|---|---|
| **Observer 1** | **165;167** |
| **Observer 2** | **165.1** |
| **Observer 3** | **165.1** |

## 30- Wild. Locus AT1 sample PONT95 (0158)

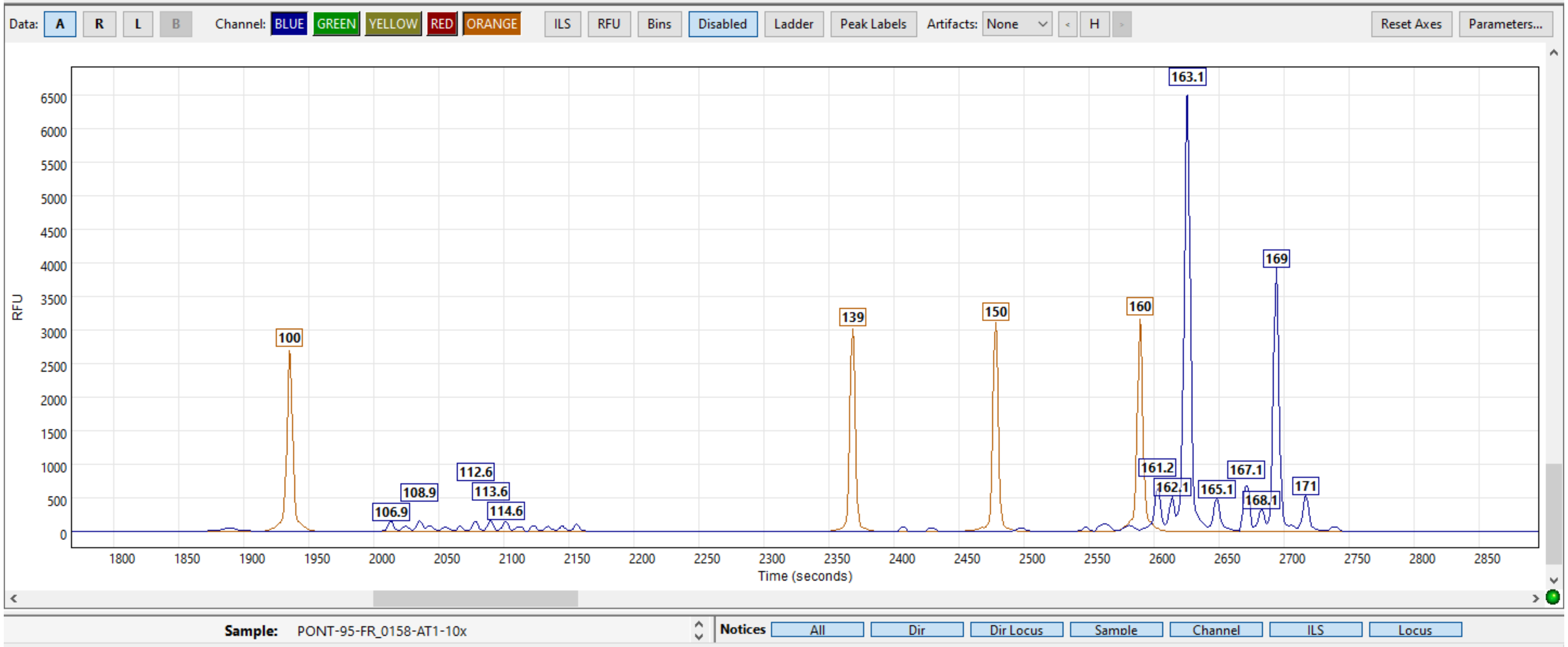


| | |
|---|---|
| **Observer 1** | 163;169 |
| **Observer 2** | 163.1;169 |
| **Observer 3** | 163.1;169 |

**Samples - Colony. Locus AG2**

**1- Colony. Locus ISO AG2 sample 01 (0114)**

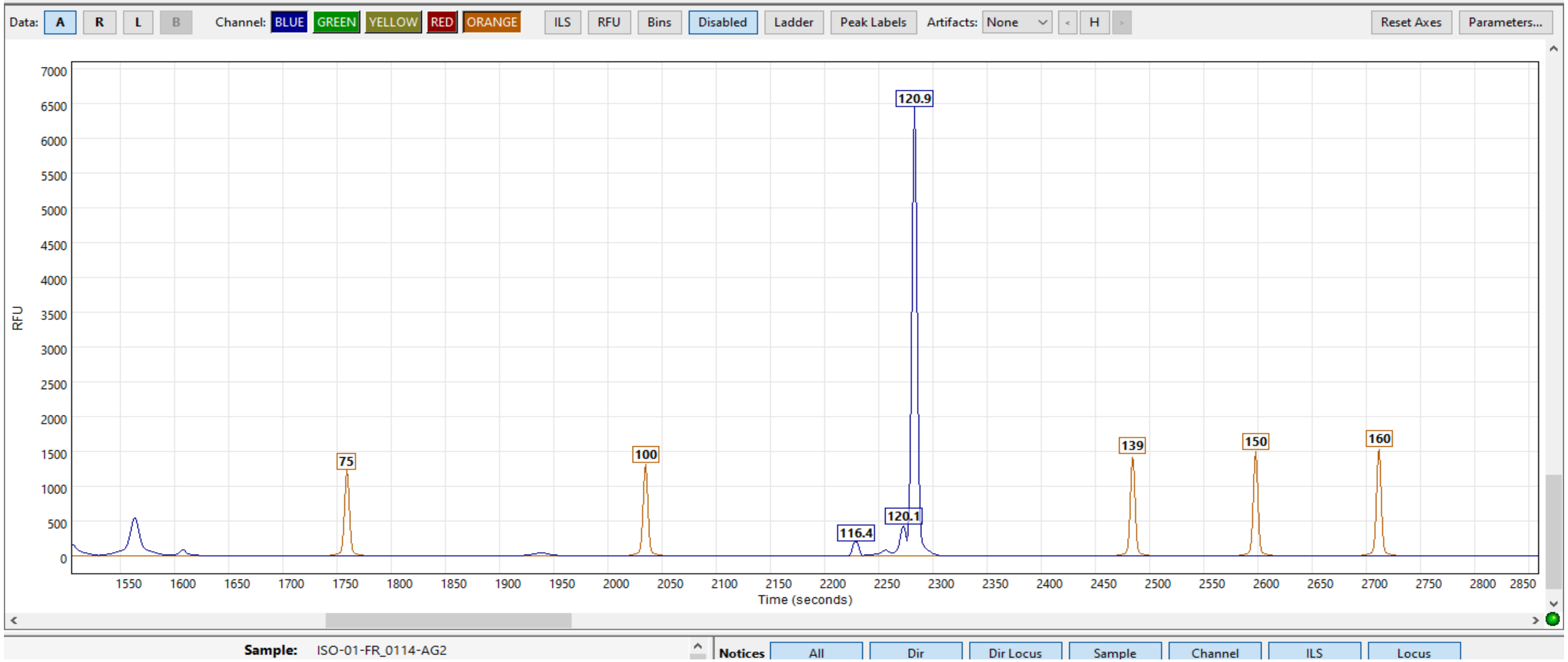


| | |
|---|---|
| **Observer 1** | **121** |
| **Observer 2** | **121** |
| **Observer 3** | **121** |

**2- Colony. Locus ISO AG2 sample 02 (0115)**

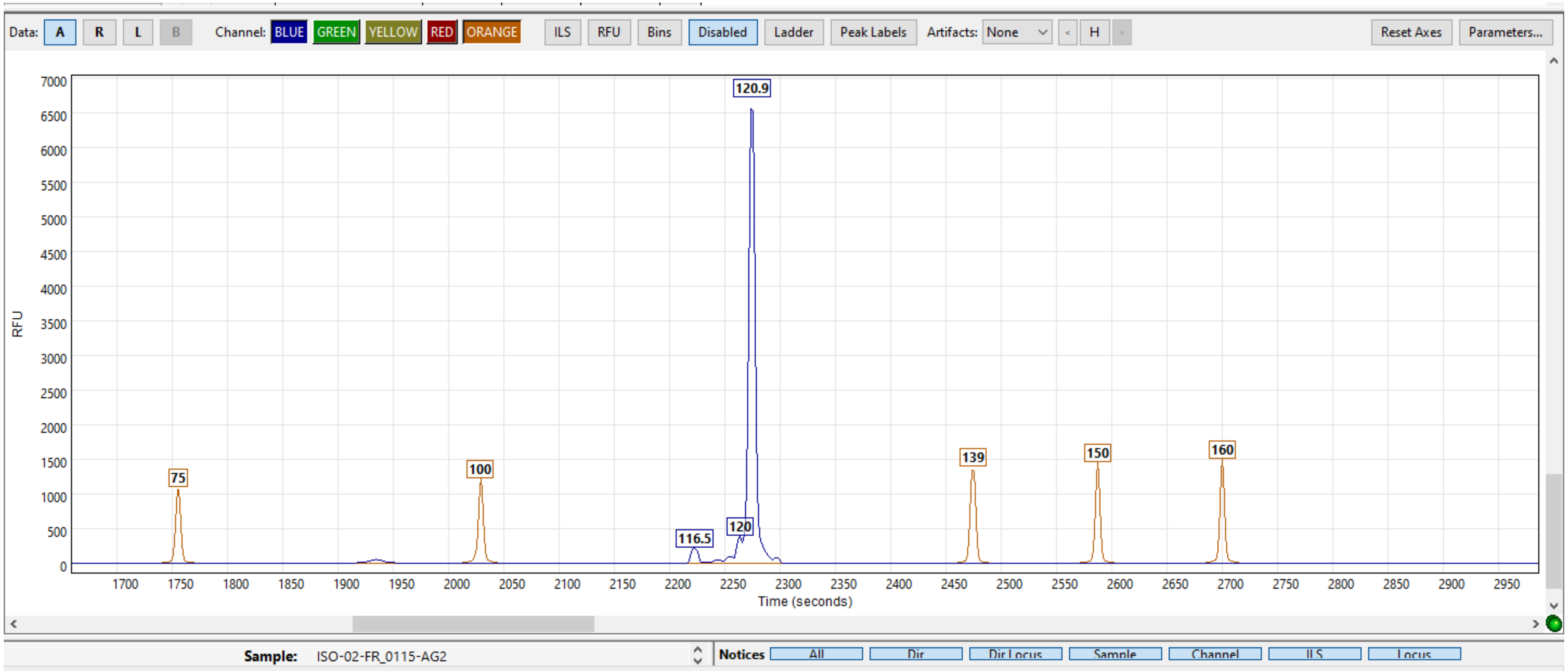


| | |
|---|---|
| **Observer 1** | **121** |
| **Observer 2** | **121** |
| **Observer 3** | **121** |

**3- Colony. Locus ISO AG2 sample 03 (0116)**

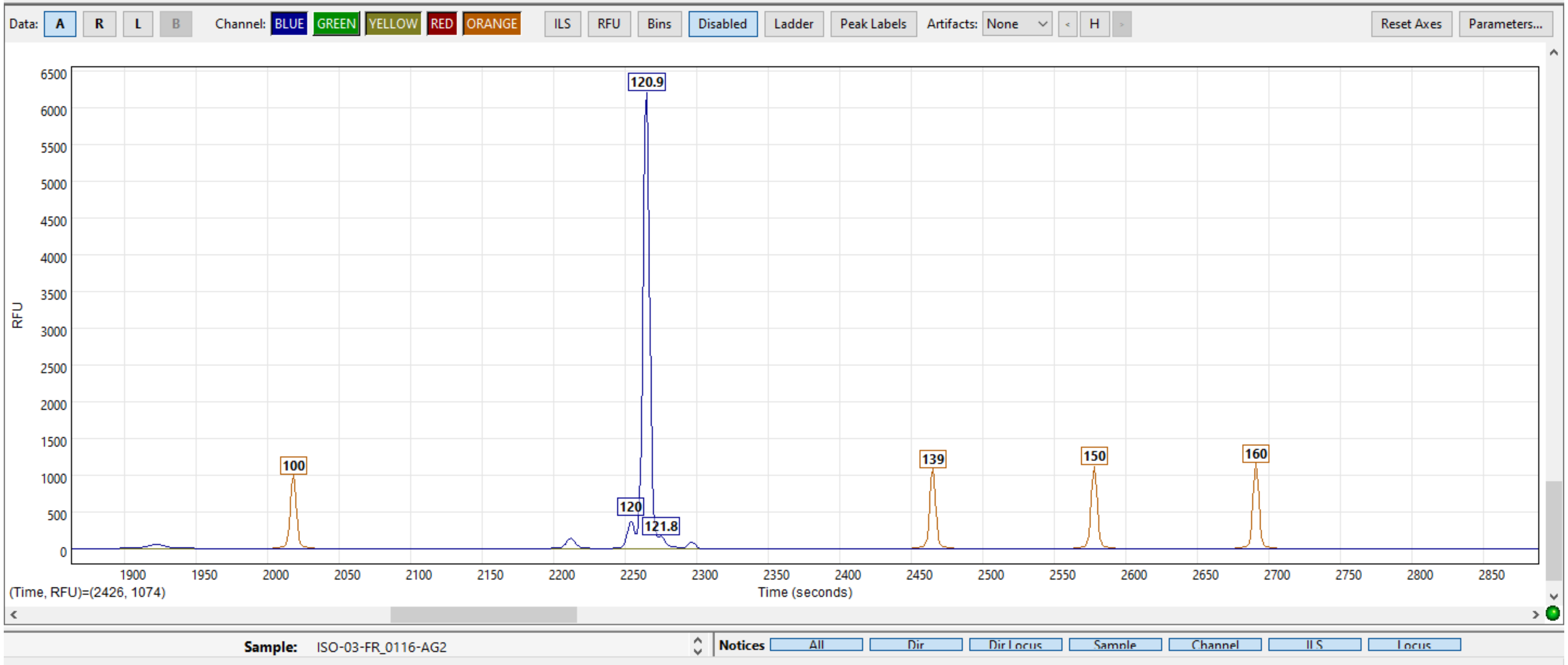


| | |
|---|---|
| **Observer 1** | **121** |
| **Observer 2** | **121** |
| **Observer 3** | **121** |

**4- Colony. Locus ISO AG2 sample 04 (0117)**

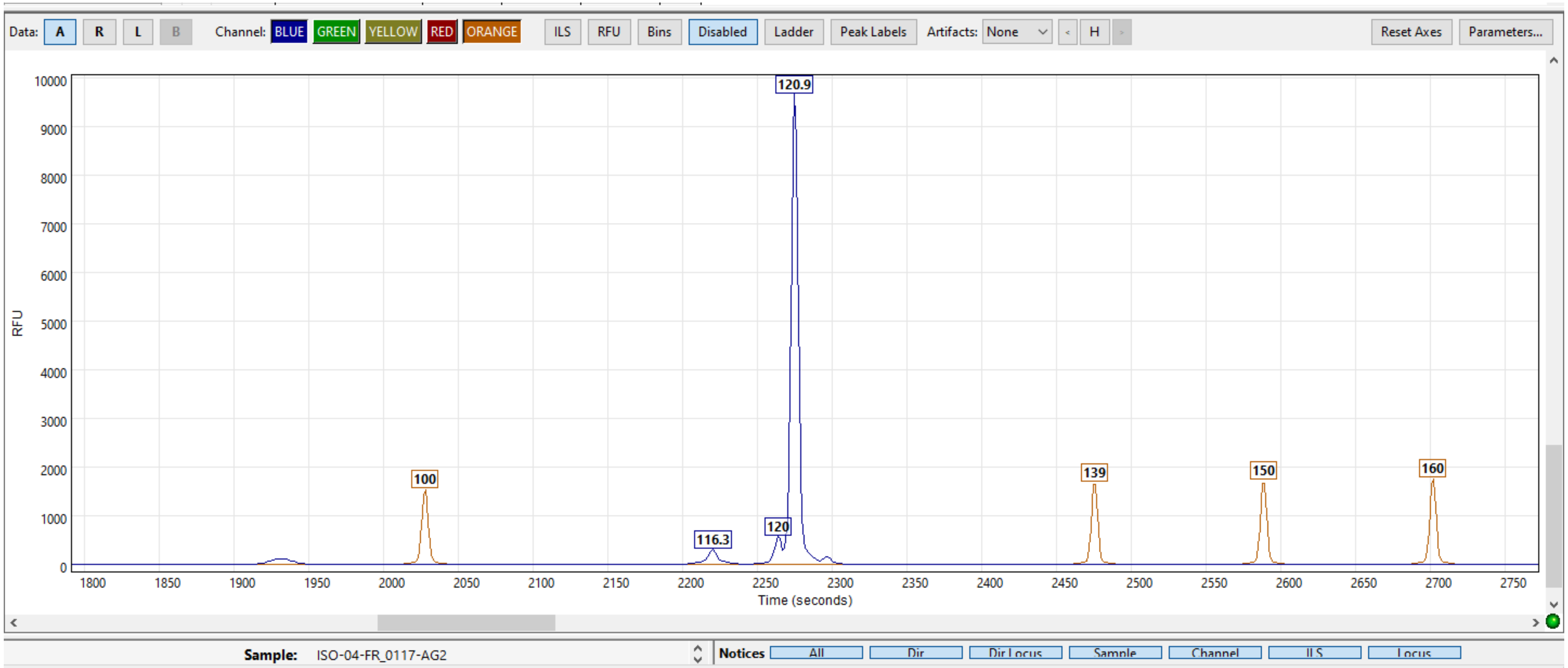


| | |
|---|---|
| **Observer 1** | **121** |
| **Observer 2** | **121** |
| **Observer 3** | **121** |

**5- Colony. Locus ISO AG2 sample 05 (0118)**

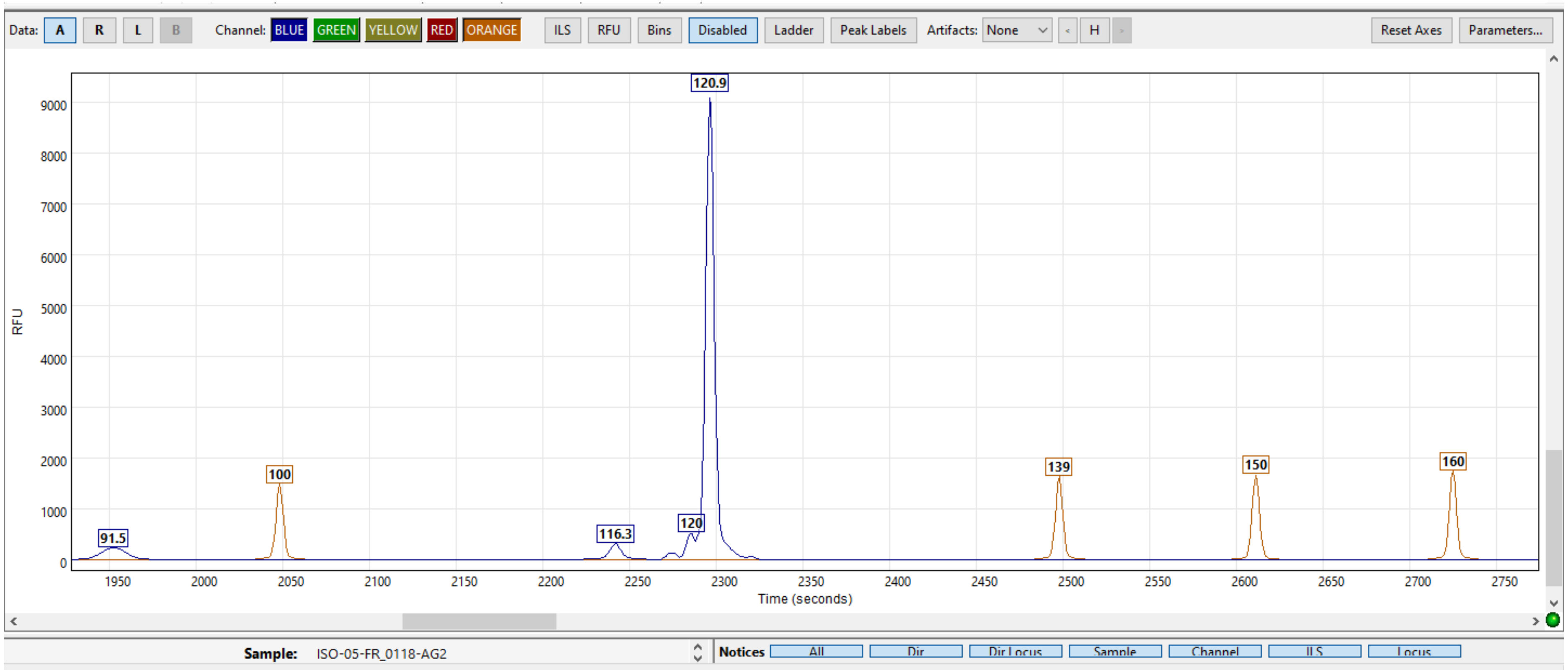


| | |
|---|---|
| **Observer 1** | **121** |
| **Observer 2** | **121** |
| **Observer 3** | **121** |

**6- Colony. Locus ISO AG2 sample 06 (0119)**

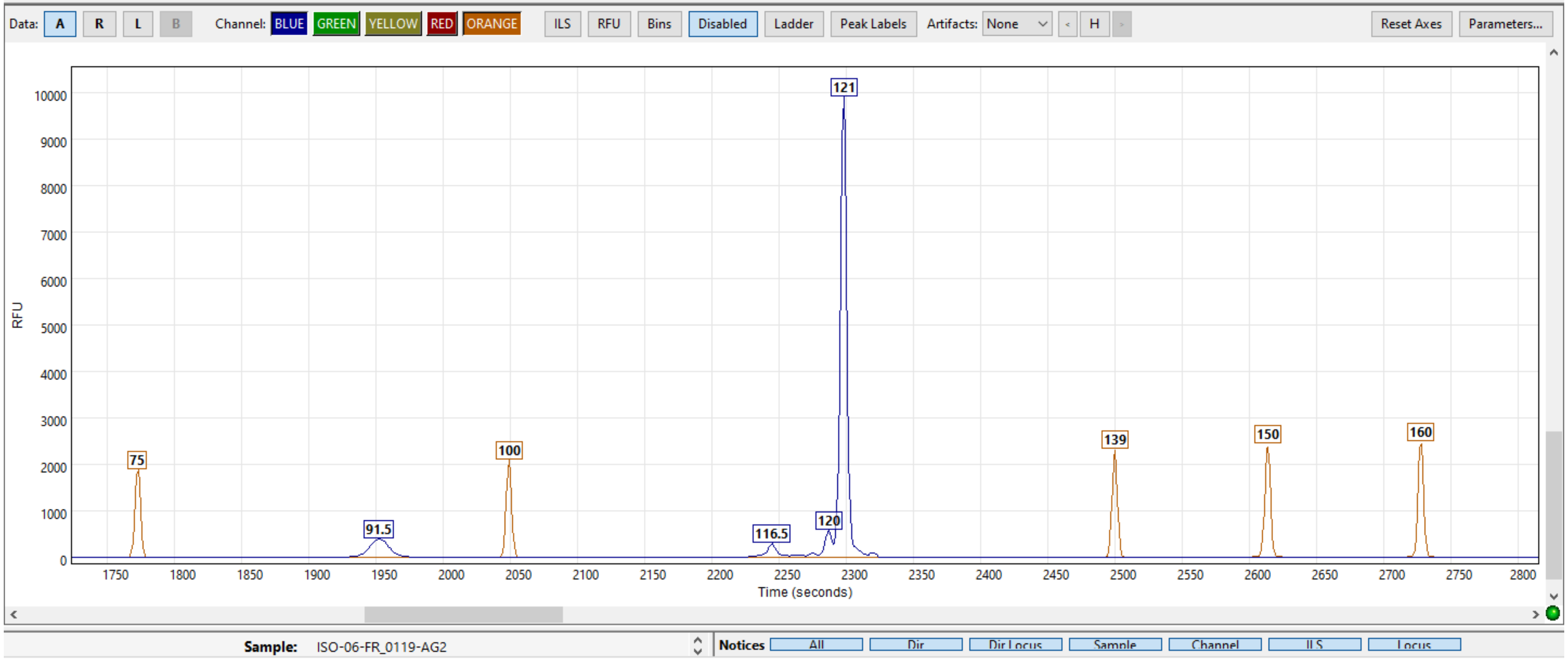


| | |
|---|---|
| **Observer 1** | **121** |
| **Observer 2** | **121** |
| **Observer 3** | **121** |

**7- Colony. Locus ISO AG2 sample 07 (0120)**

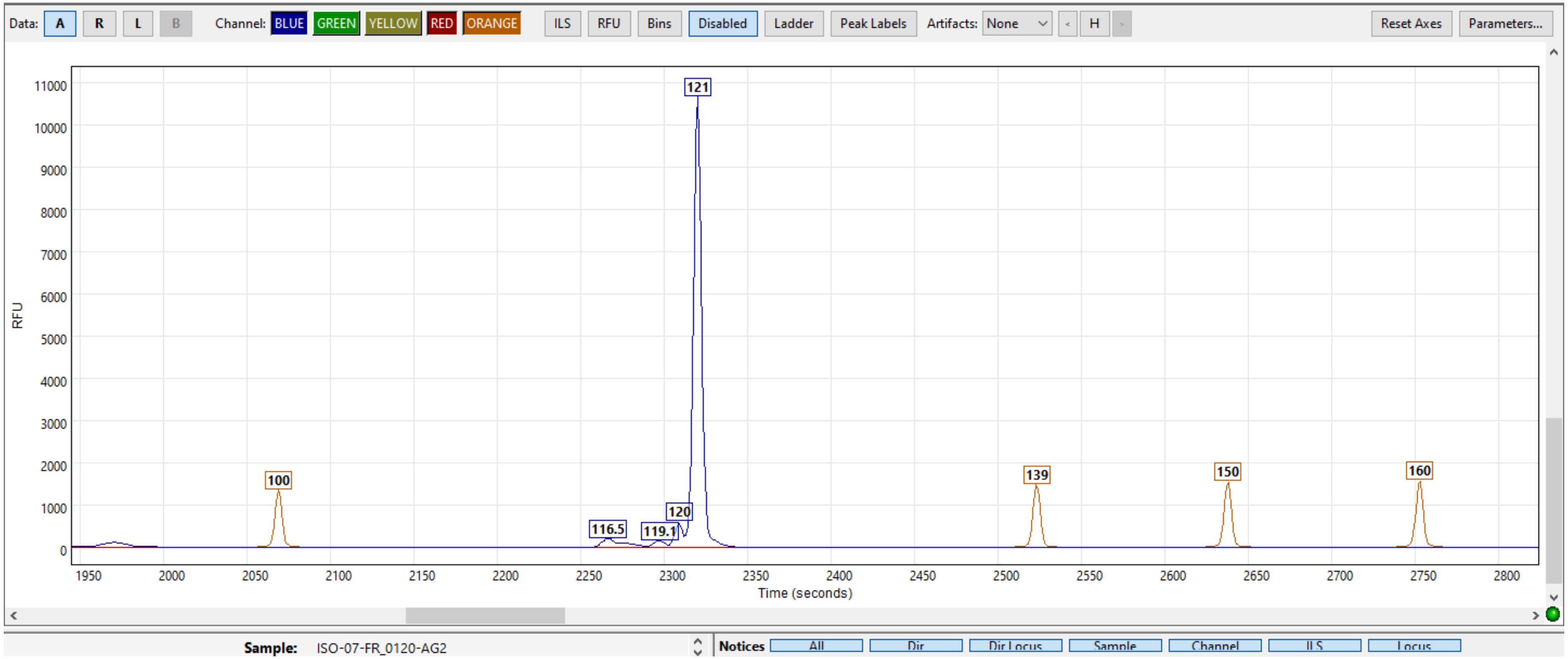


| | |
|---|---|
| **Observer 1** | **121** |
| **Observer 2** | **121** |
| **Observer 3** | **121** |

## 8- Colony. Locus ISO AG2 sample 08 (0121)

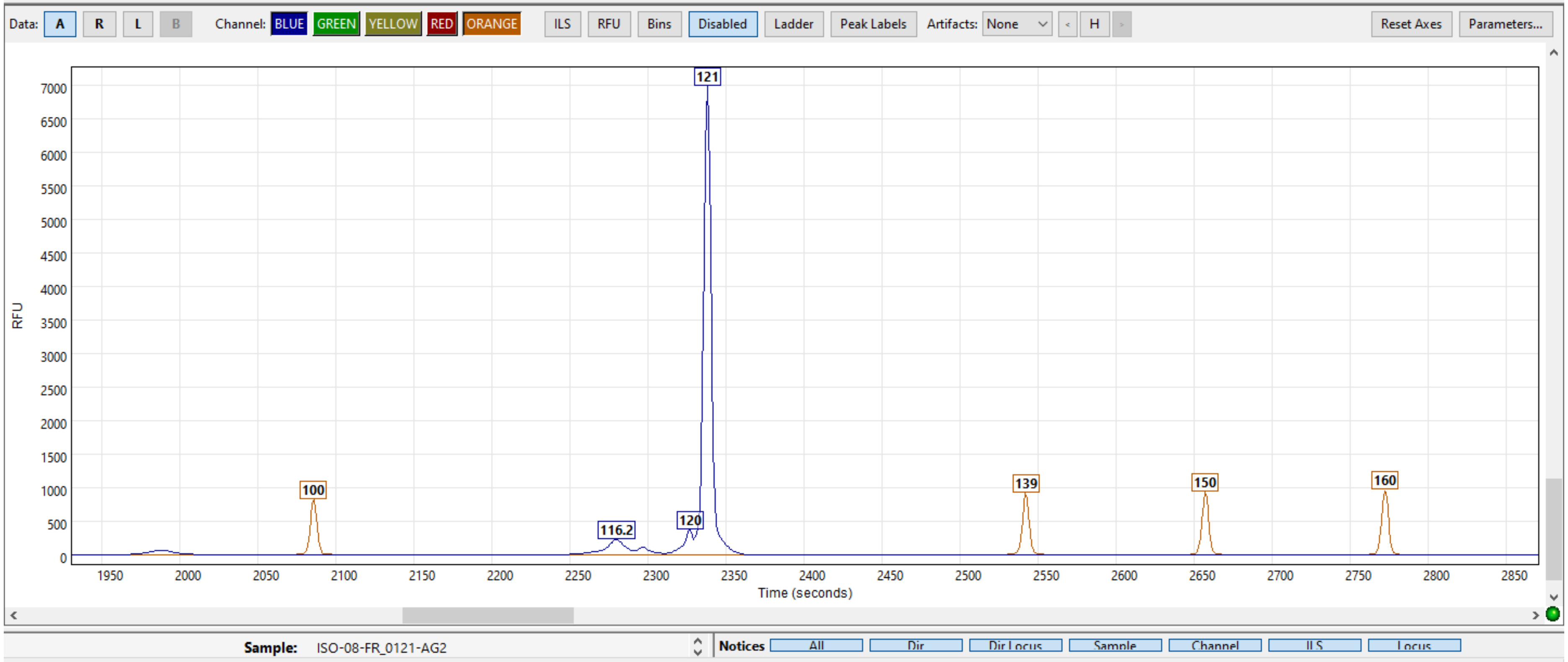


| | |
|---|---|
| **Observer 1** | **121** |
| **Observer 2** | **121** |
| **Observer 3** | **121** |

**9- Colony. Locus ISO AG2 sample 09 (0122)**

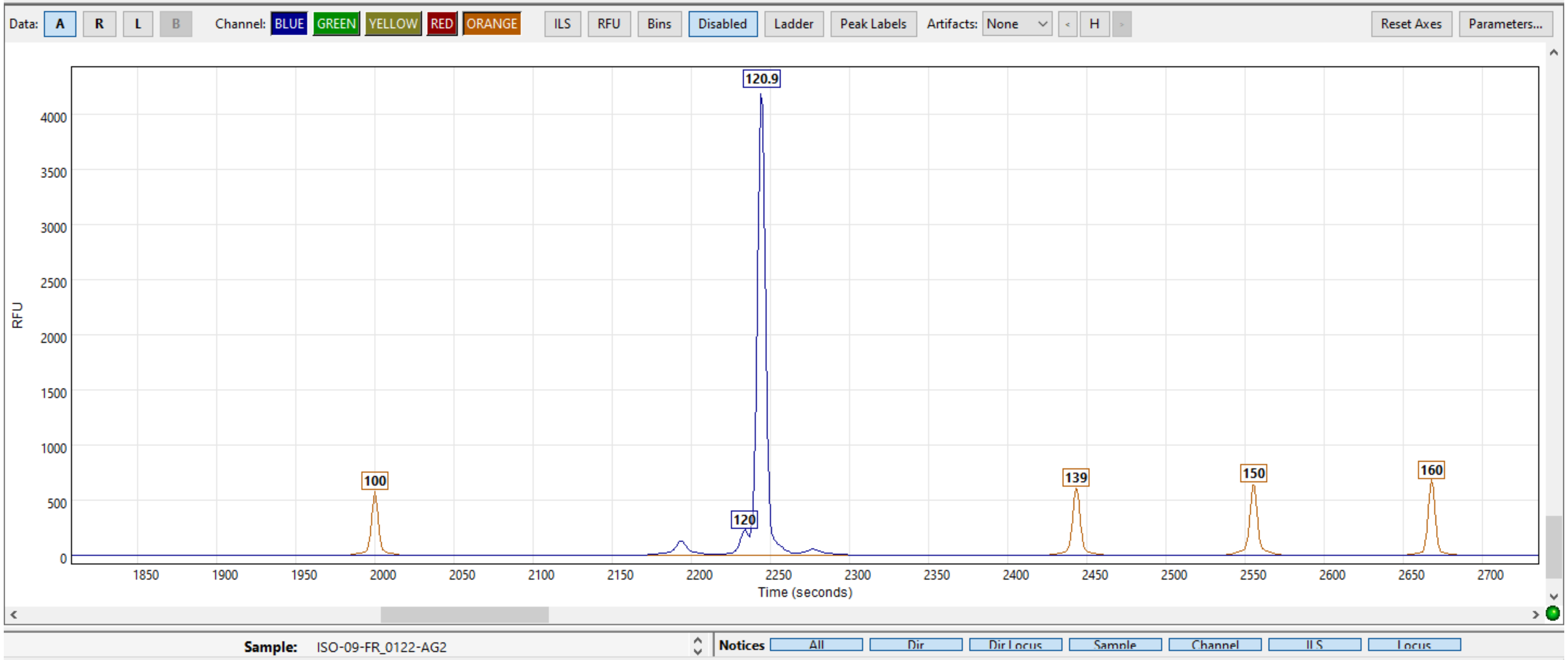


| | |
|---|---|
| **Observer 1** | **121** |
| **Observer 2** | **121** |
| **Observer 3** | **121** |

**10- Colony. Locus ISO AG2 sample 10 (0123)**

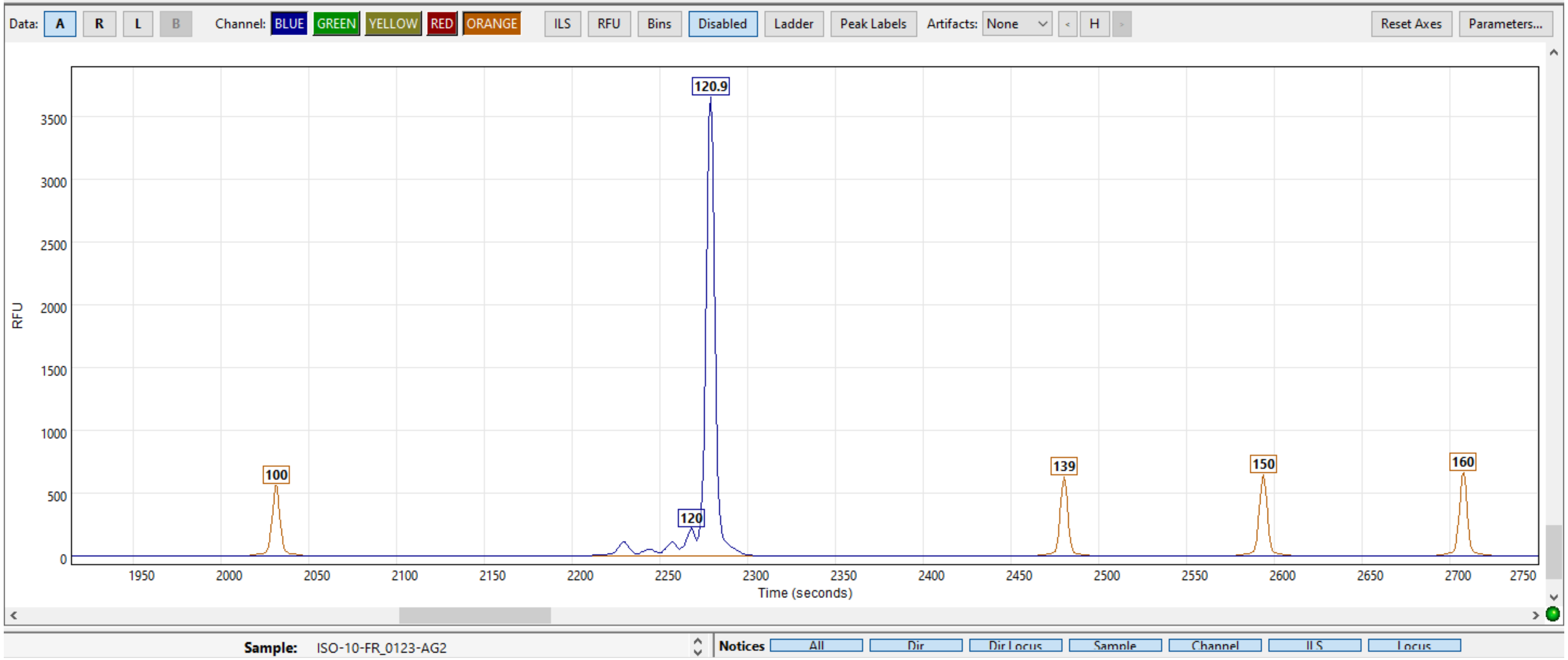


| | |
|---|---|
| **Observer 1** | **121** |
| **Observer 2** | **121** |
| **Observer 3** | **121** |

**11- Colony. Locus ISO AG2 sample 11 (0124)**

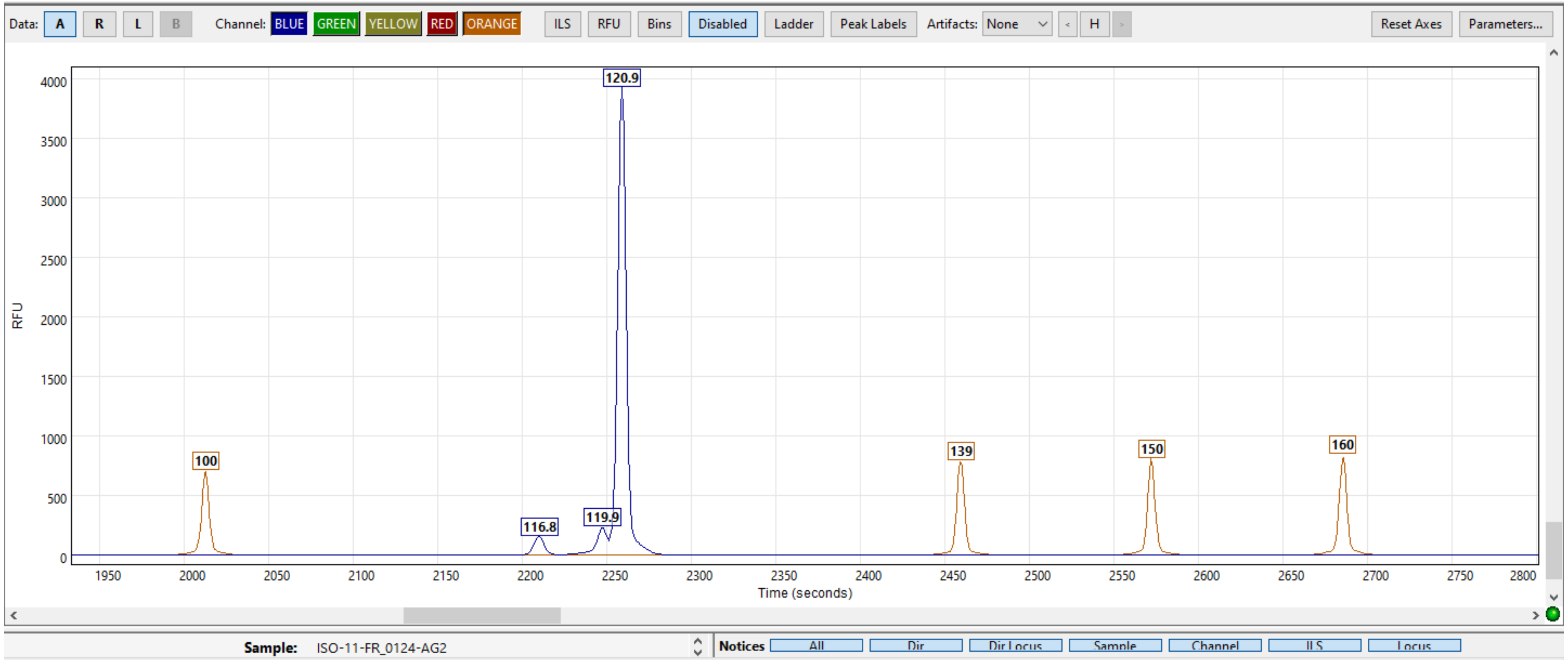


| | |
|---|---|
| **Observer 1** | **121** |
| **Observer 2** | **121** |
| **Observer 3** | **121** |

**12- Colony. Locus ISO AG2 sample 12 (0125)**

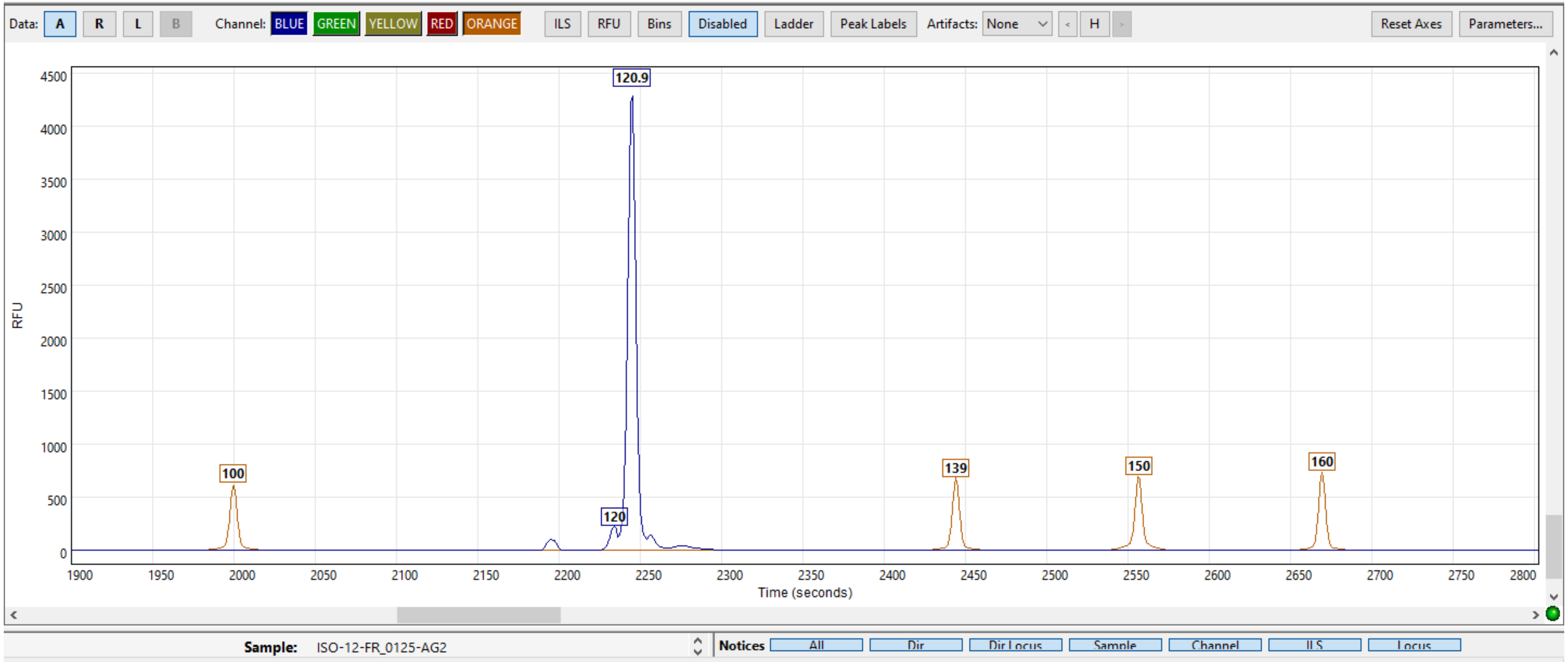


| | |
|---|---|
| **Observer 1** | **121** |
| **Observer 2** | **121** |
| **Observer 3** | **121** |

## 13- Colony. Locus ISO AG2 sample 13 (0126)

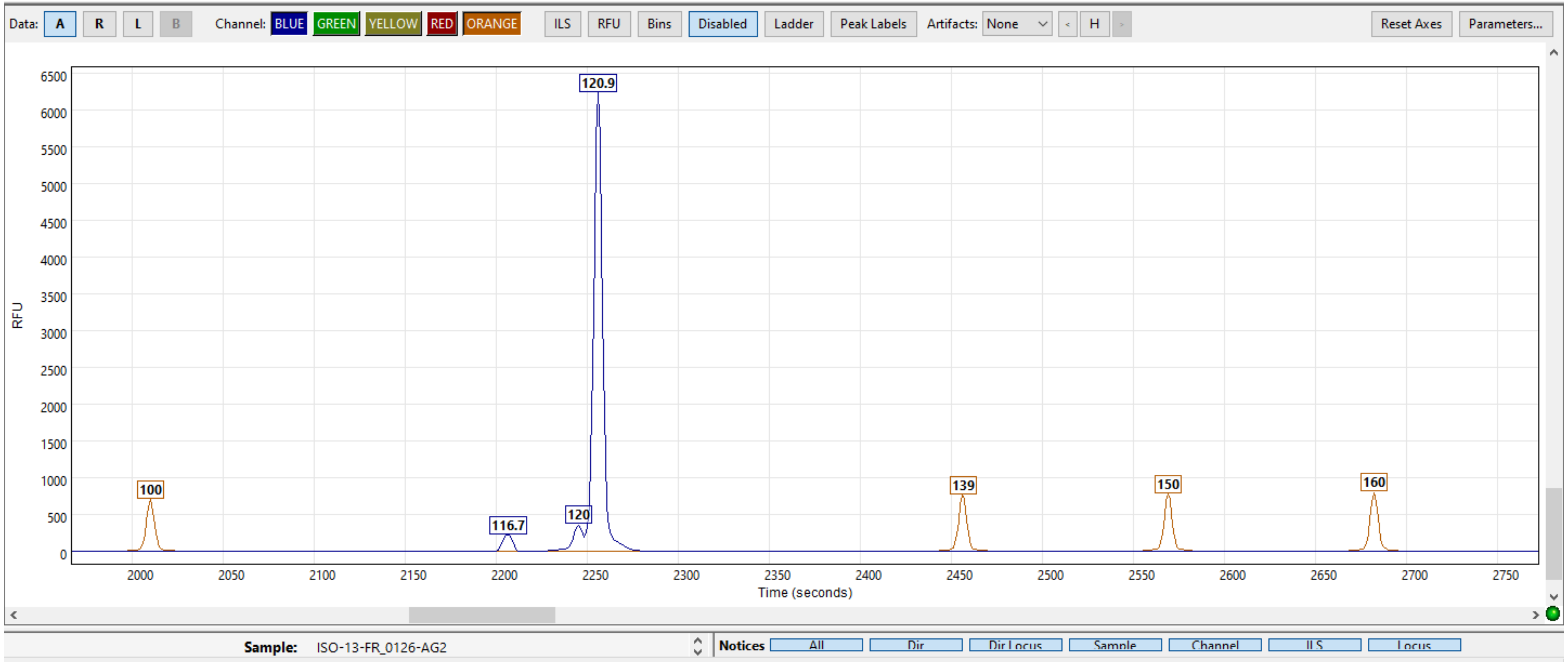


| | |
|---|---|
| **Observer 1** | **121** |
| **Observer 2** | **121** |
| **Observer 3** | **121** |

### 14- Colony. Locus ISO AG2 sample 14 (0127)

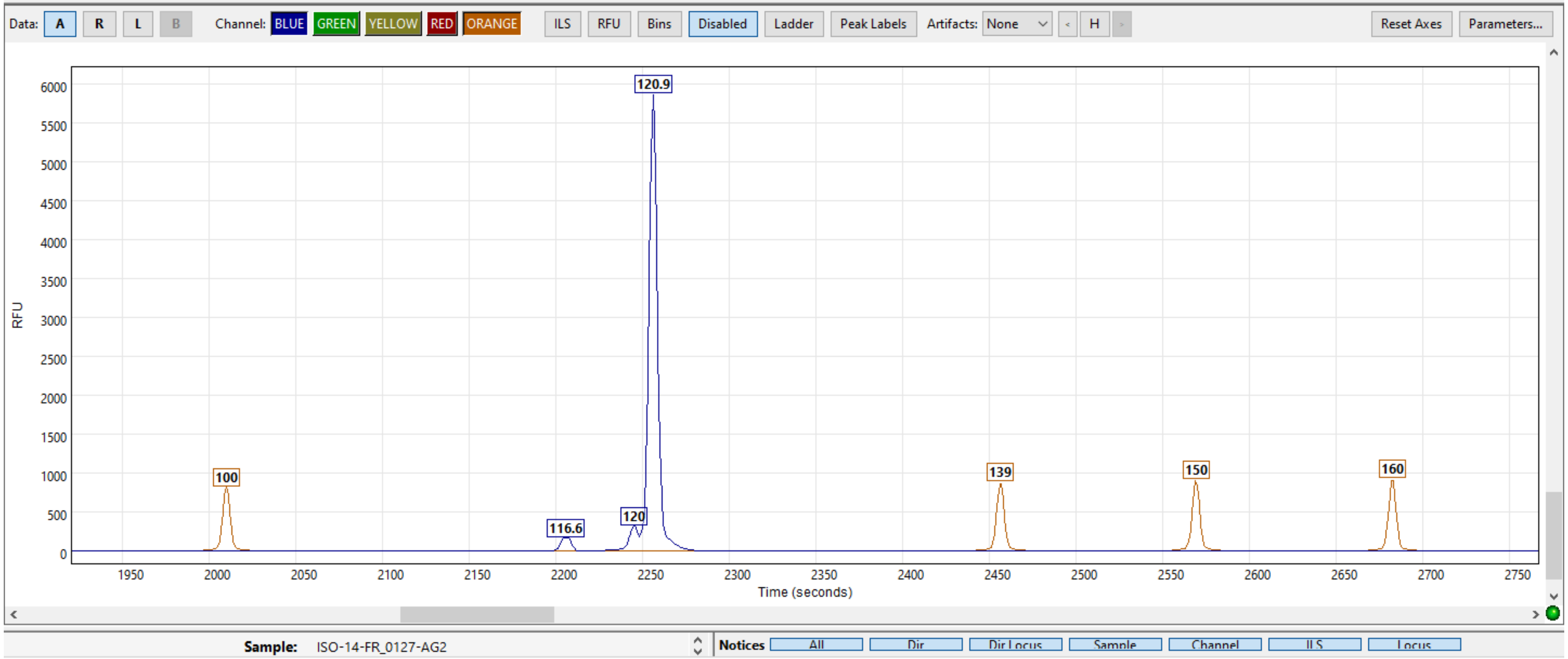


| | |
|---|---|
| **Observer 1** | **121** |
| **Observer 2** | **121** |
| **Observer 3** | **121** |

**15- Colony. Locus ISO AG2 sample 15 (0128)**

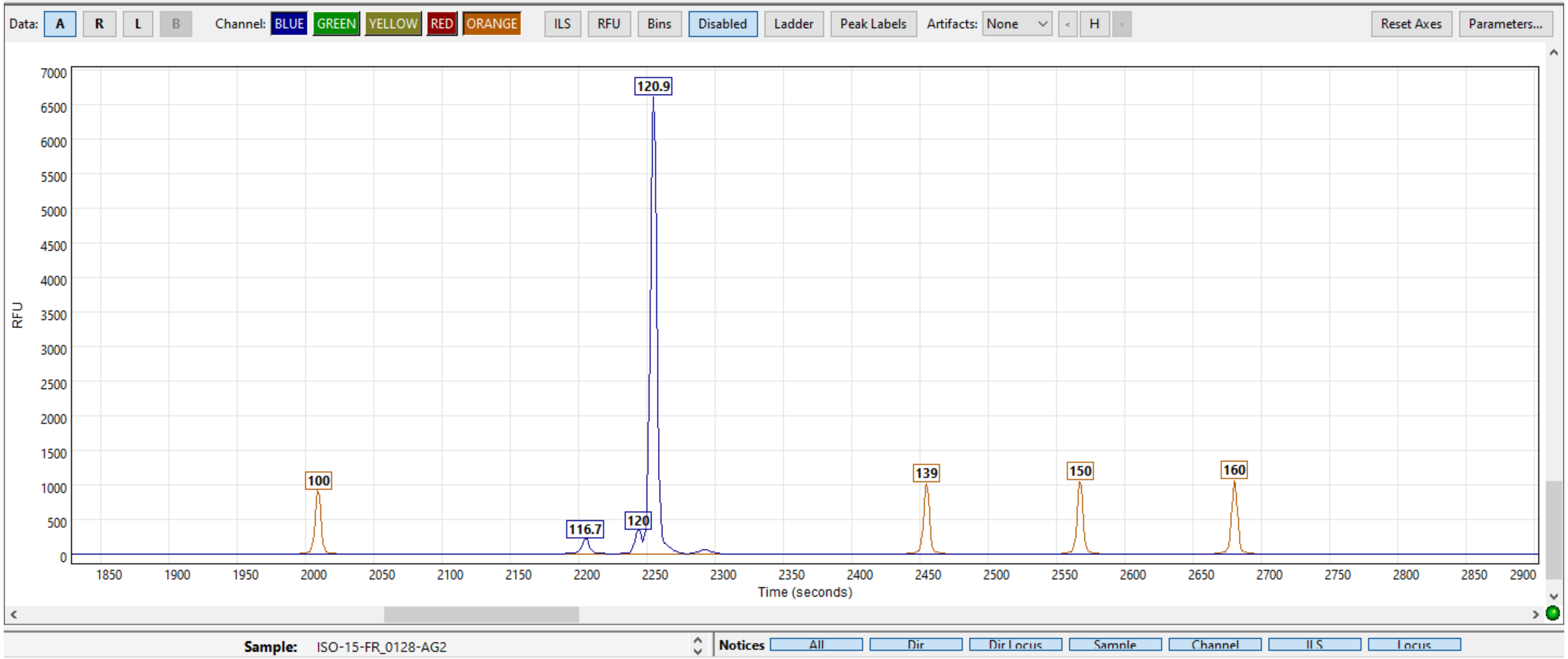


| | |
|---|---|
| **Observer 1** | **121** |
| **Observer 2** | **121** |
| **Observer 3** | **121** |

### 16- Colony. Locus ISO AG2 sample 16 (0129)

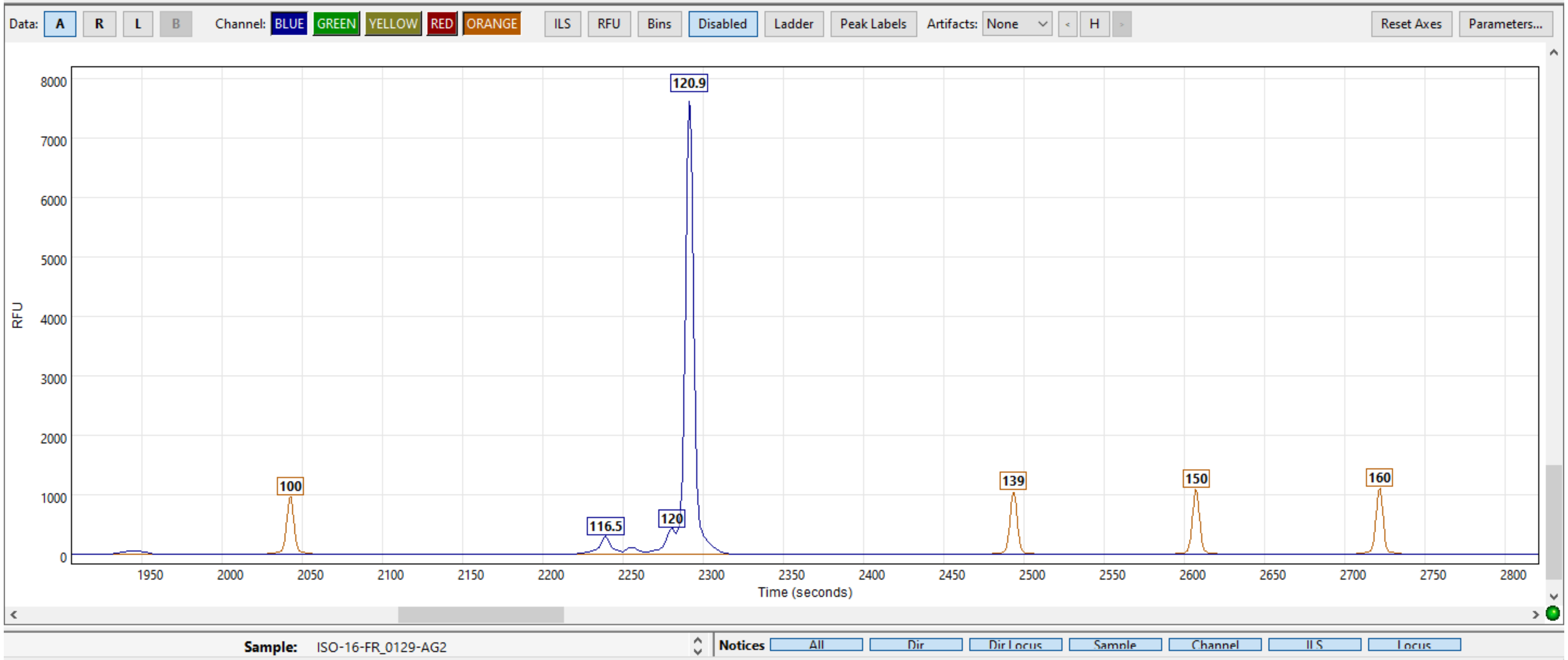


| | |
|---|---|
| **Observer 1** | **121** |
| **Observer 2** | **121** |
| **Observer 3** | **121** |

**17- Colony. Locus ISO AG2 sample 17 (0130)**

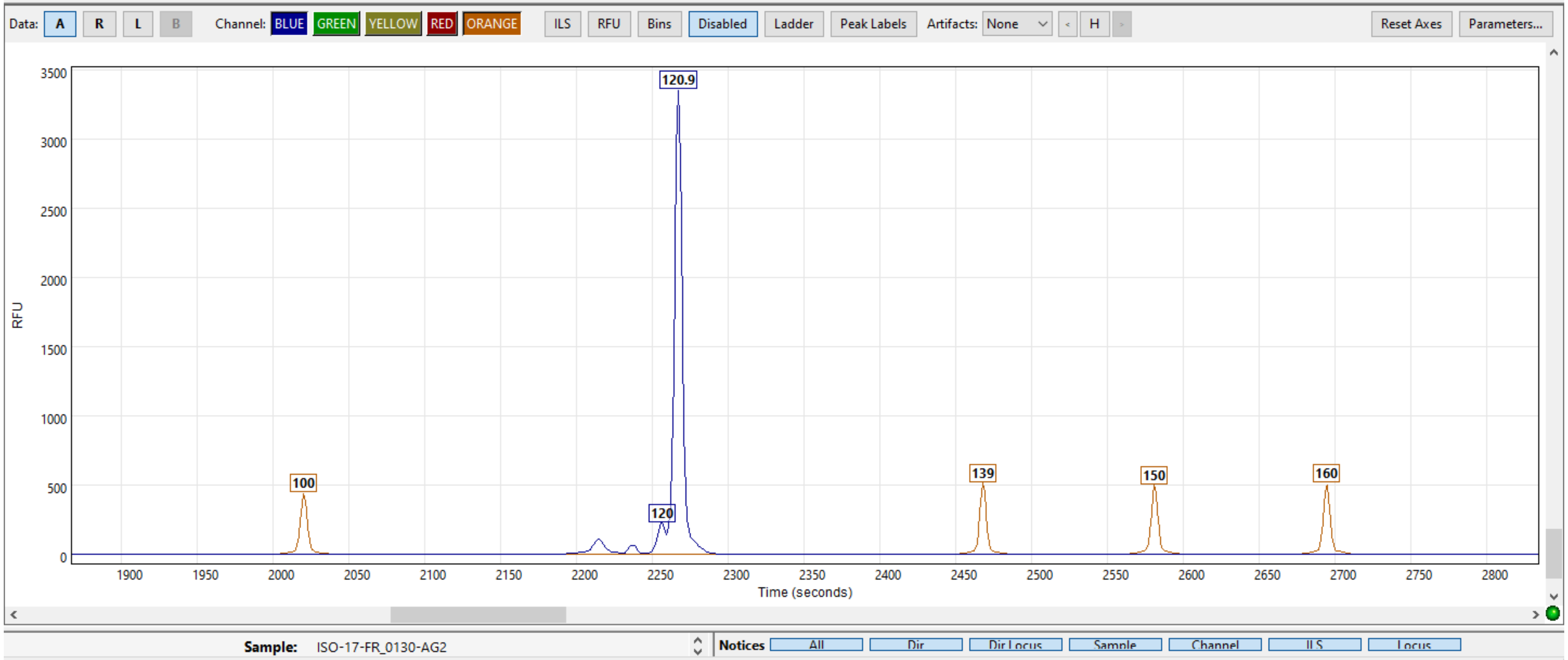


| | |
|---|---|
| **Observer 1** | **121** |
| **Observer 2** | **121** |
| **Observer 3** | **121** |

**18- Colony. Locus ISO AG2 sample 19 (0131)**

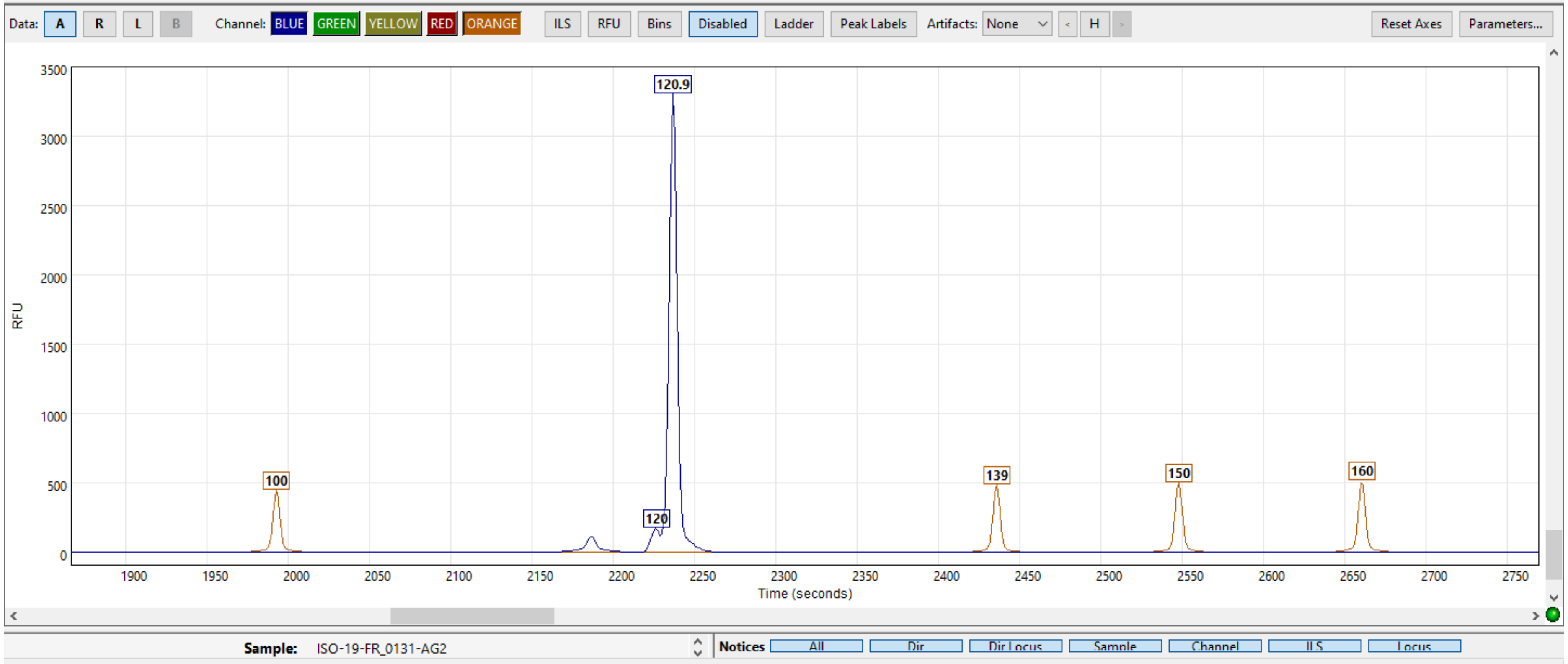


| | |
|---|---|
| **Observer 1** | **121** |
| **Observer 2** | **121** |
| **Observer 3** | **121** |

**19- Colony. Locus ISO AG2 sample 20 (0132)**

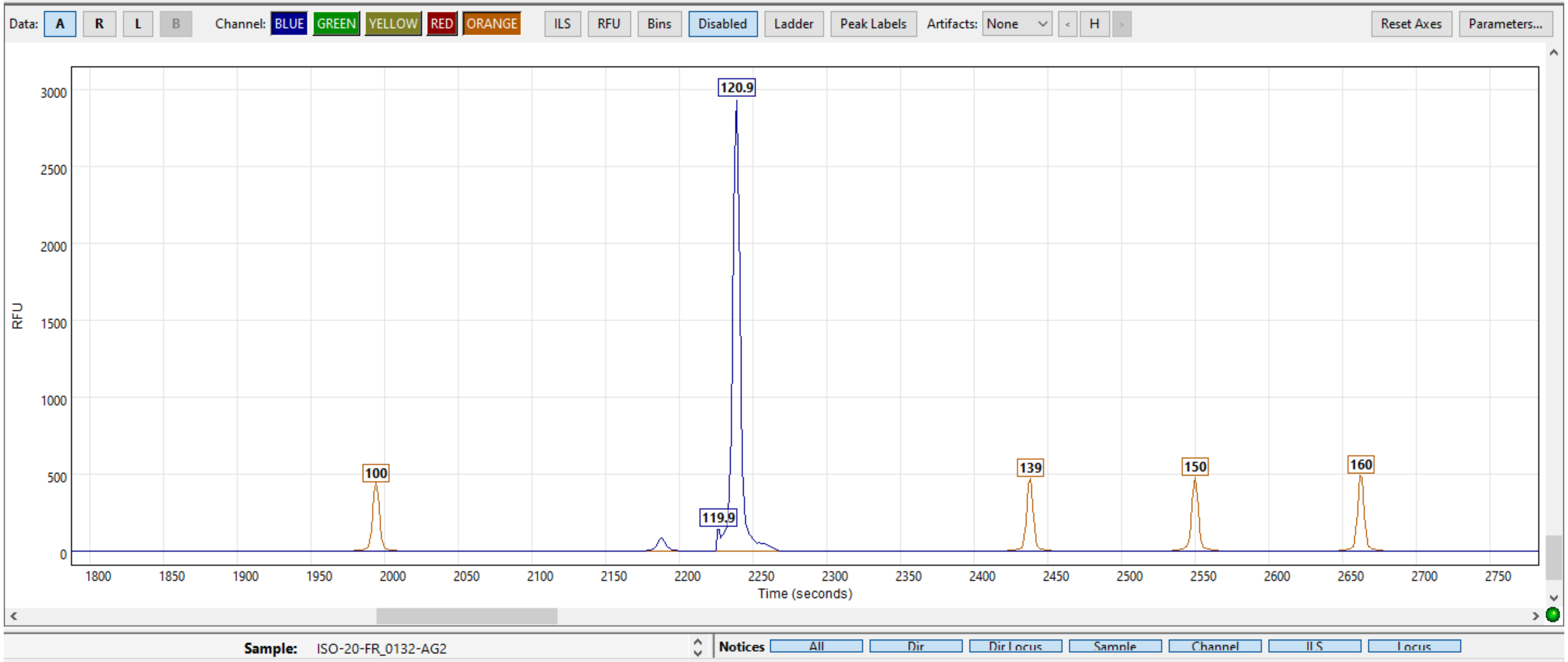


| | |
|---|---|
| **Observer 1** | **121** |
| **Observer 2** | **121** |
| **Observer 3** | **121** |

**20- Colony. Locus ISO AG2 sample 21 (0133)**

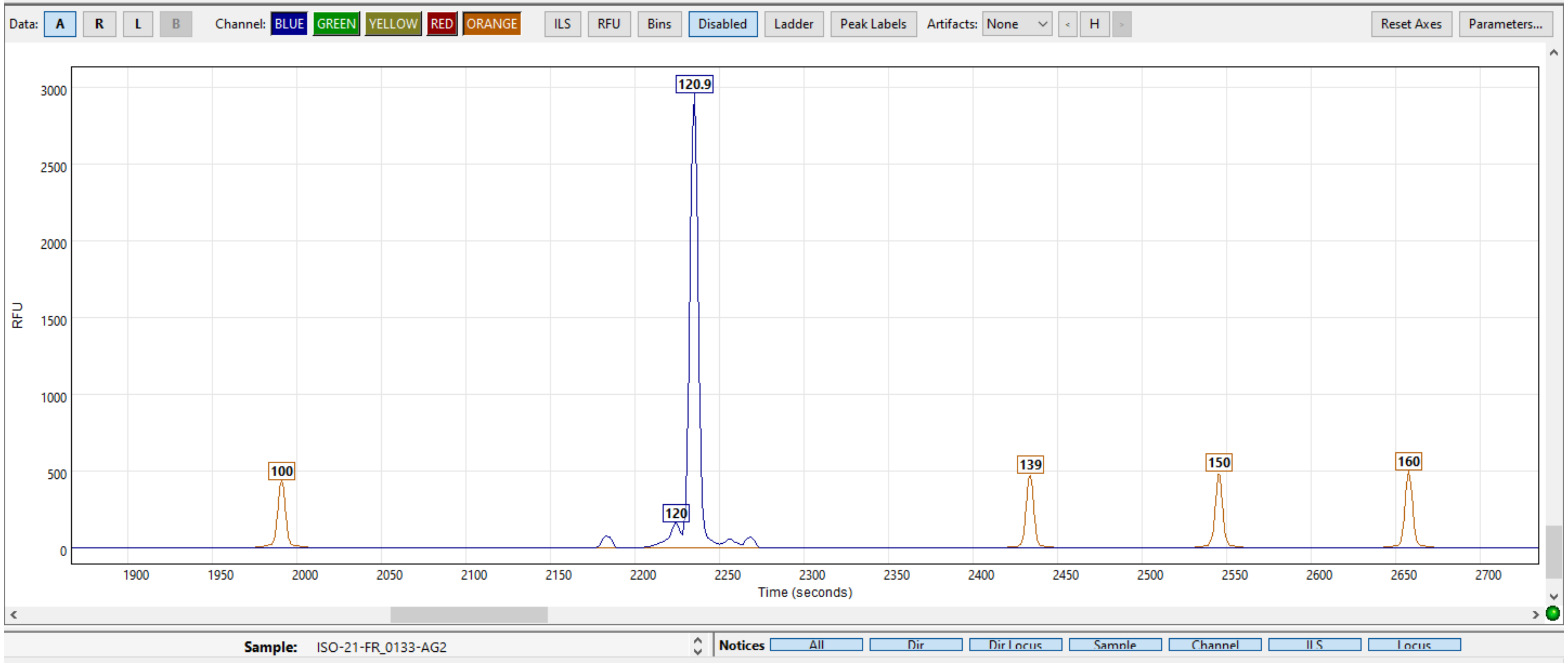


| | |
|---|---|
| **Observer 1** | **121** |
| **Observer 2** | **121** |
| **Observer 3** | **121** |

**21- Colony. Locus ISO AG2 sample 23 (0134)**

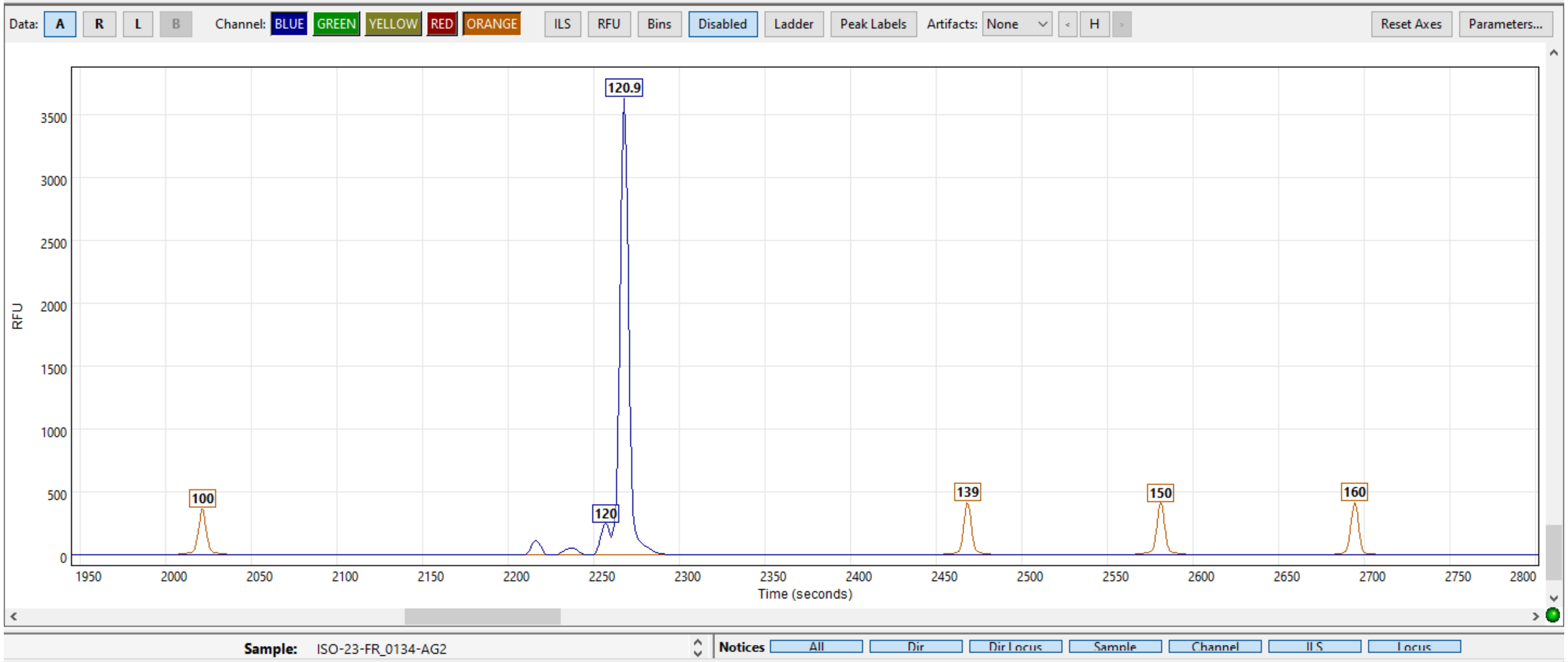


| | |
|---|---|
| **Observer 1** | **121** |
| **Observer 2** | **121** |
| **Observer 3** | **121** |

## 22- Colony. Locus ISO AG2 sample 24 (0135)

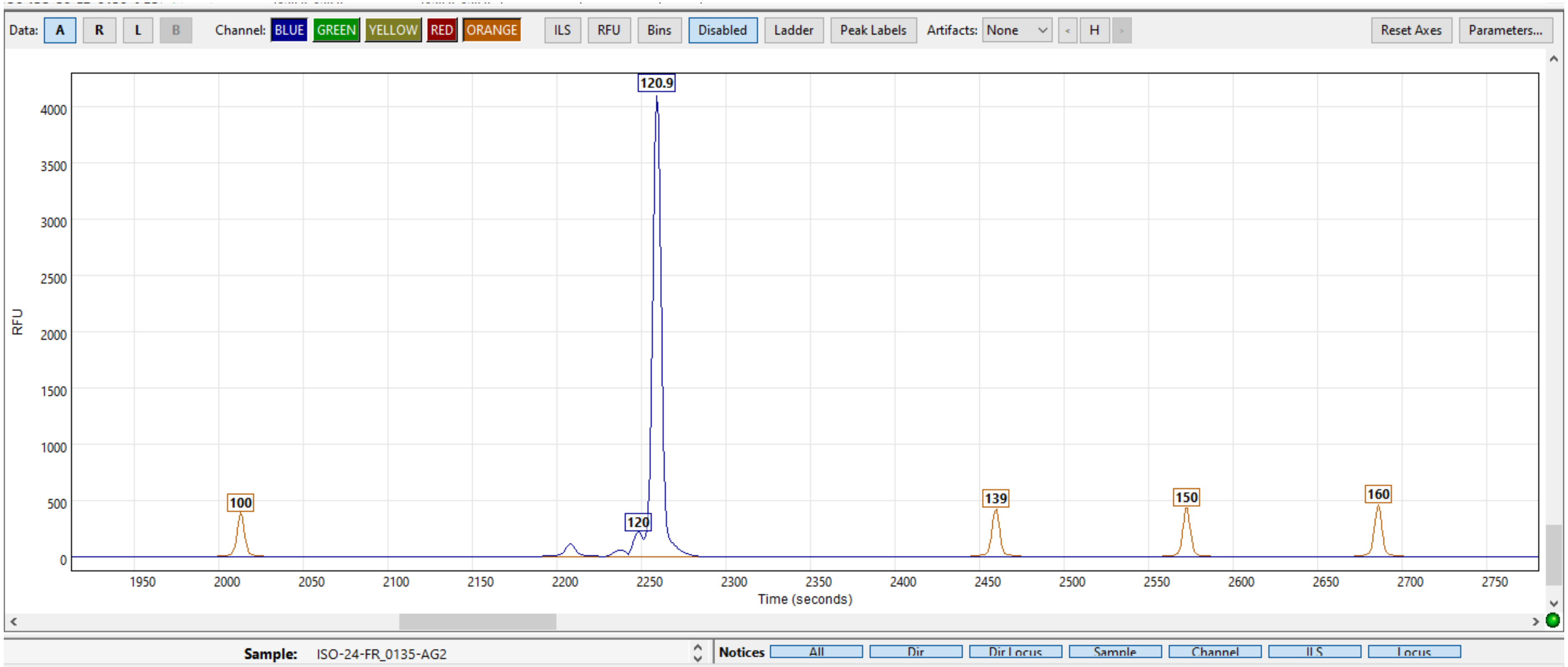


| | |
|---|---|
| **Observer 1** | **121** |
| **Observer 2** | **121** |
| **Observer 3** | **121** |

## 23- Colony. Locus ISO AG2 sample 25 (0136)

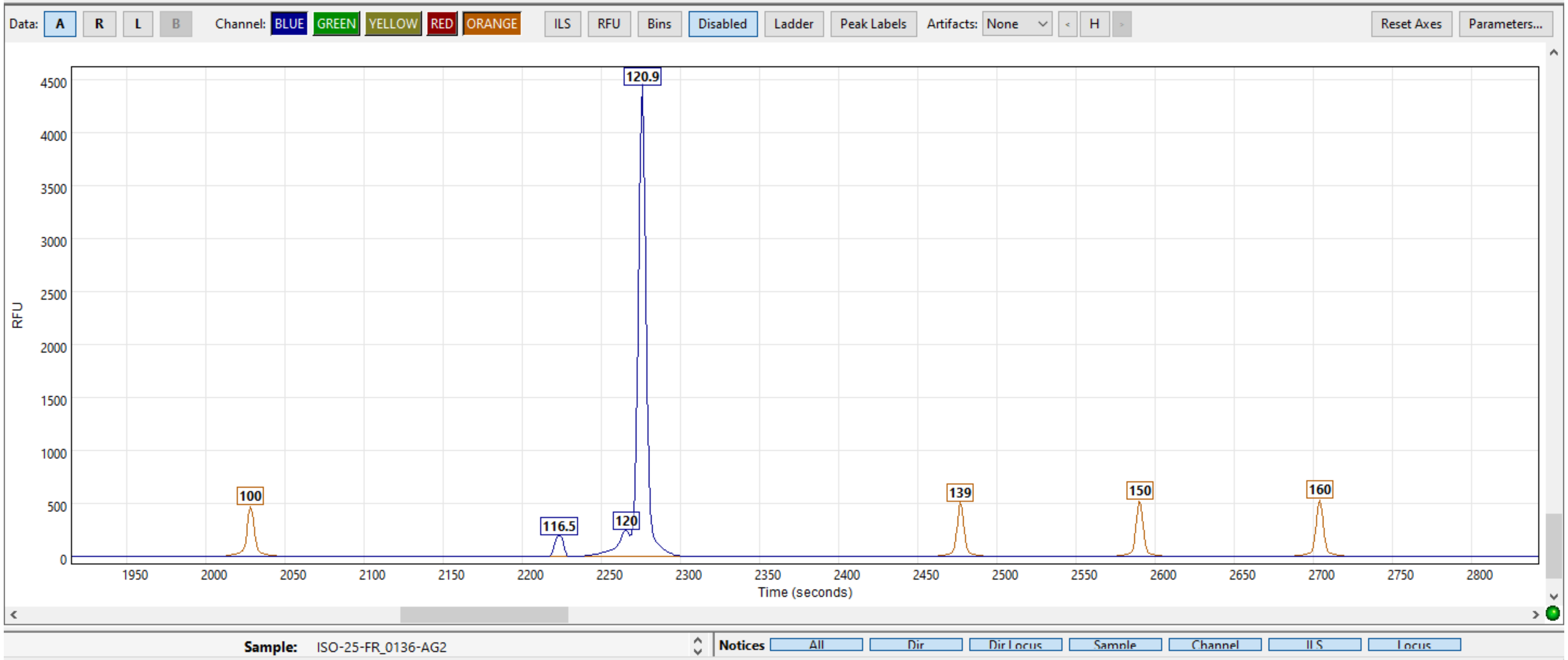


| | |
|---|---|
| **Observer 1** | **121** |
| **Observer 2** | **121** |
| **Observer 3** | **121** |

**24- Colony. Locus ISO AG2 sample 26 (0137)**

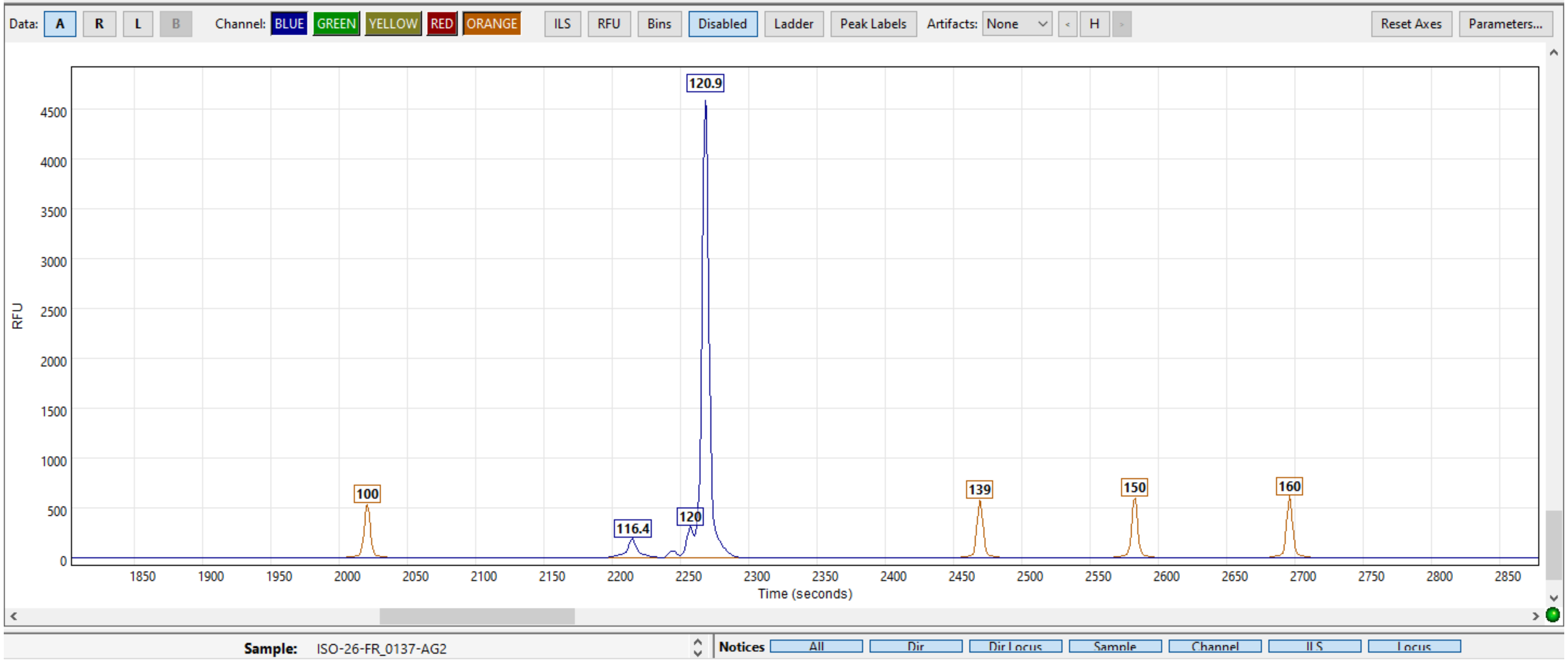


| | |
|---|---|
| **Observer 1** | **121** |
| **Observer 2** | **121** |
| **Observer 3** | **121** |

## 25- Colony. Locus ISO AG2 sample 27 (0138)

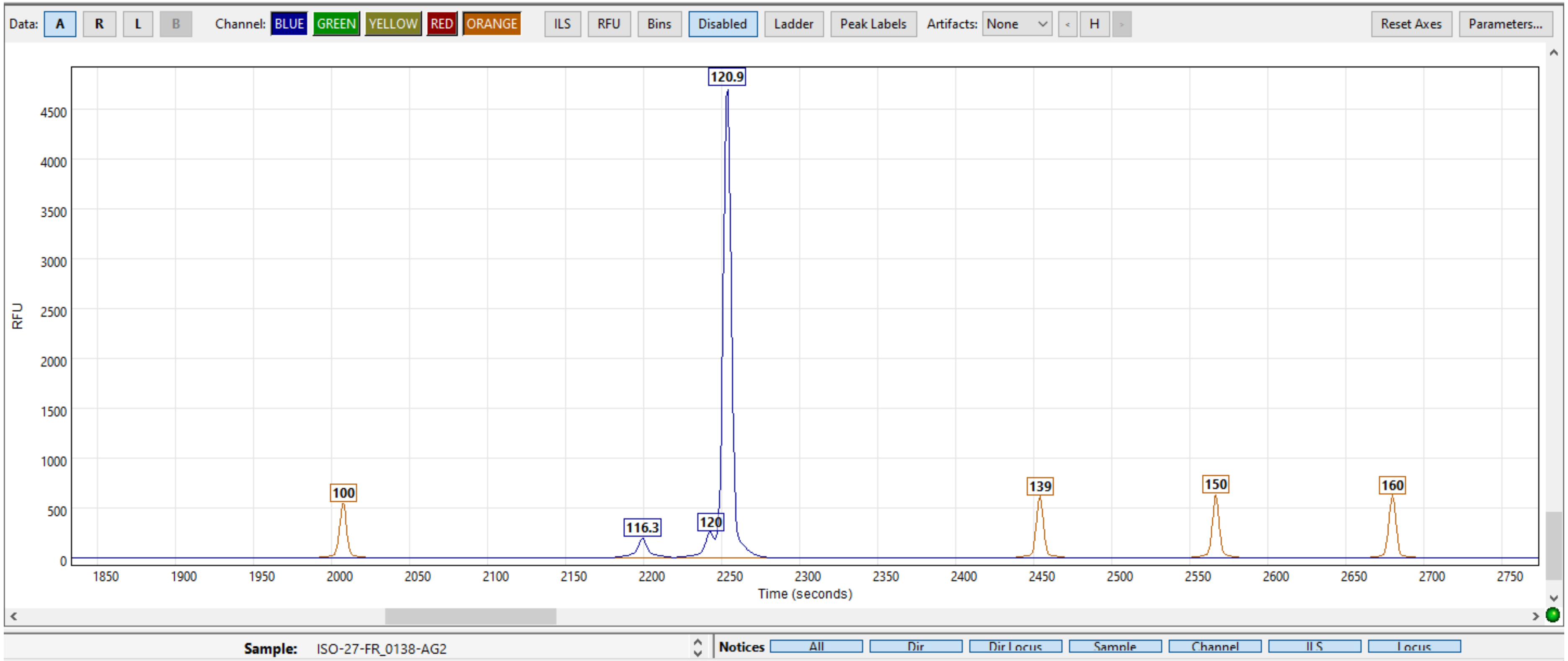


| | |
|---|---|
| **Observer 1** | **121** |
| **Observer 2** | **121** |
| **Observer 3** | **121** |

**26- Colony. Locus ISO AG2 sample 29 (0139)**

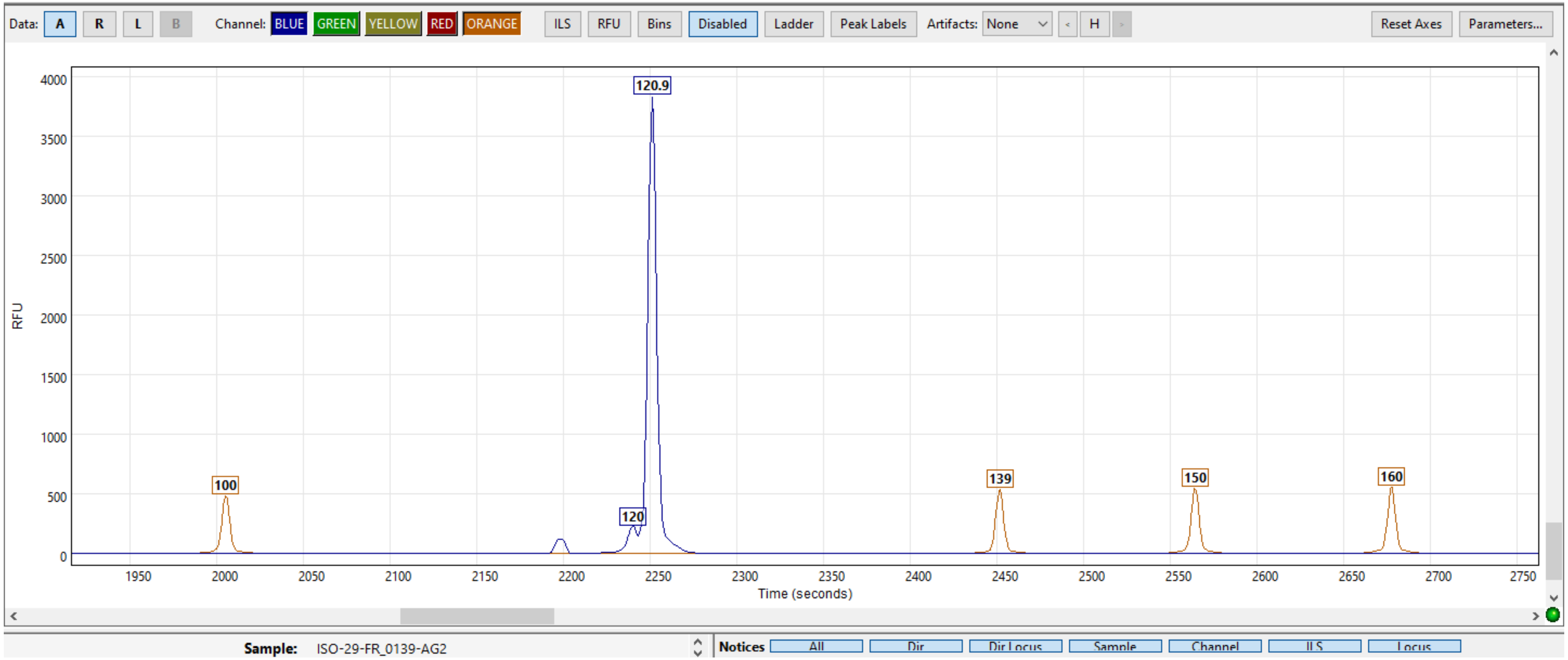


| | |
|---|---|
| **Observer 1** | **121** |
| **Observer 2** | **121** |
| **Observer 3** | **121** |

**27- Colony. Locus ISO AG2 sample 30 (0140)**

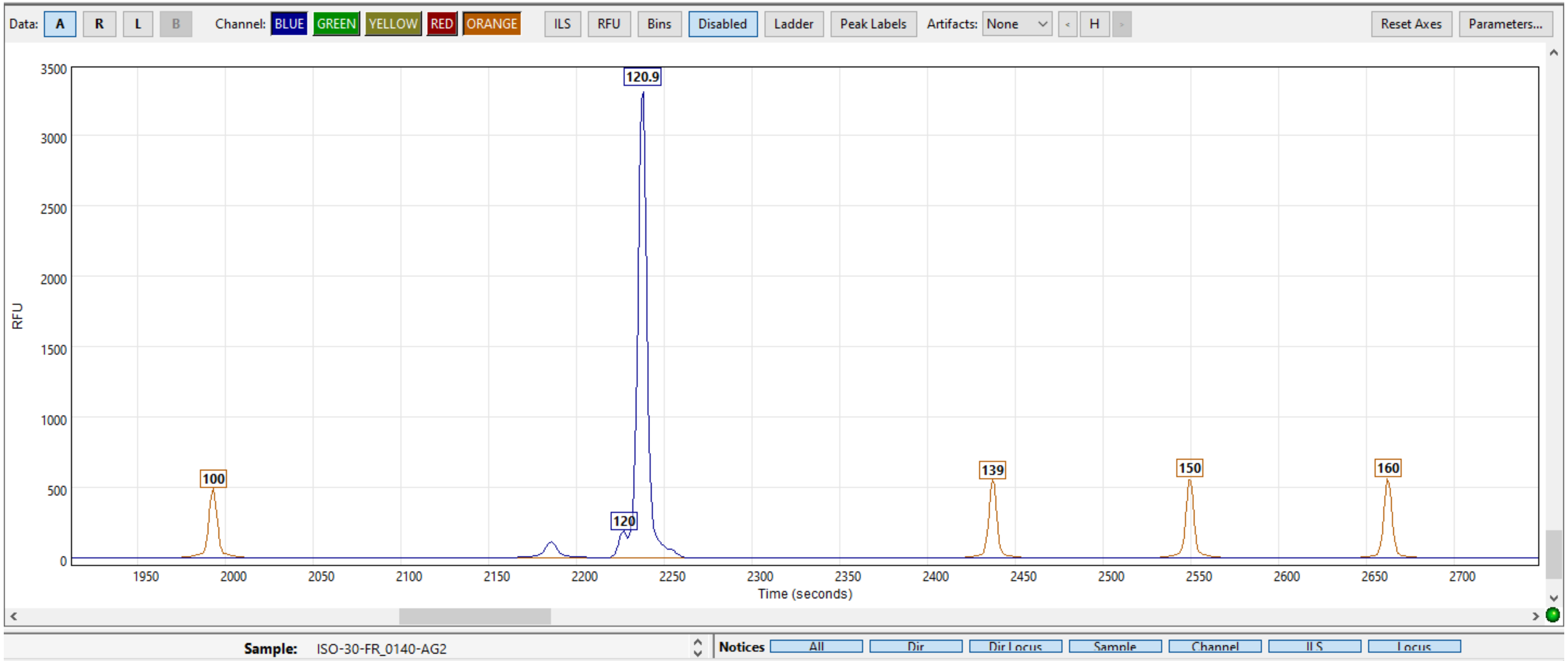


| | |
|---|---|
| **Observer 1** | **121** |
| **Observer 2** | **121** |
| **Observer 3** | **121** |

## 28- Colony. Locus ISO AG2 sample 31 (0141)

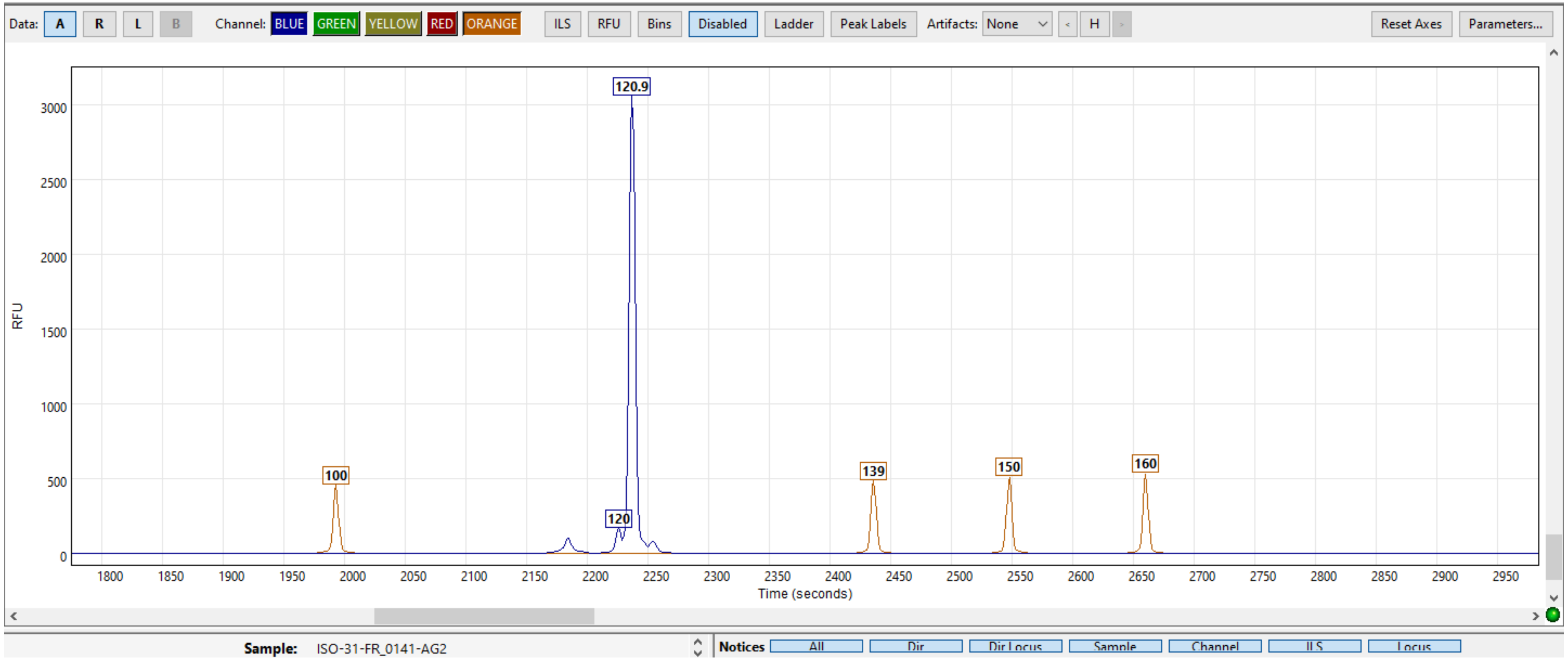


| | |
|---|---|
| **Observer 1** | **121** |
| **Observer 2** | **121** |
| **Observer 3** | **121** |

## 29- Colony. Locus ISO AG2 sample 32 (0142)

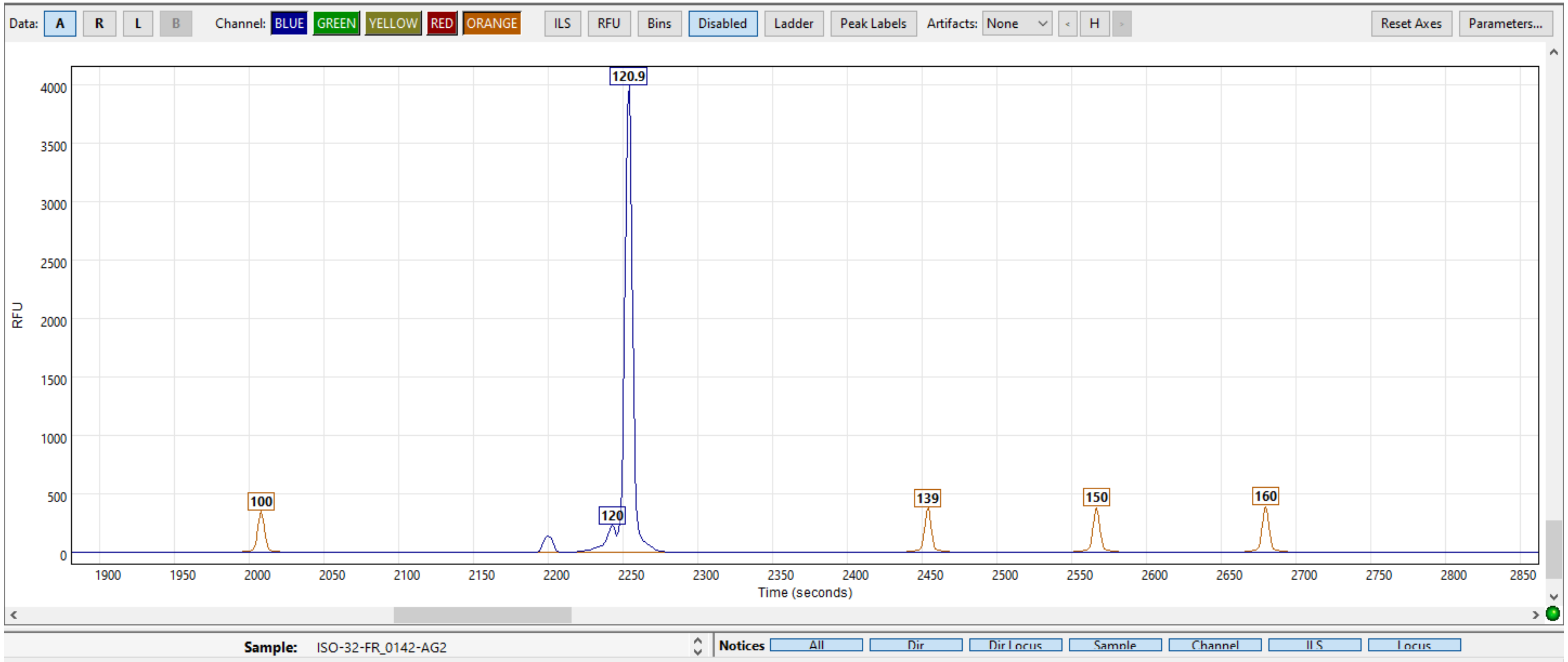


| | |
|---|---|
| **Observer 1** | **121** |
| **Observer 2** | **121** |
| **Observer 3** | **121** |

## 30- Colony. Locus ISO AG2 sample 33 (0143)

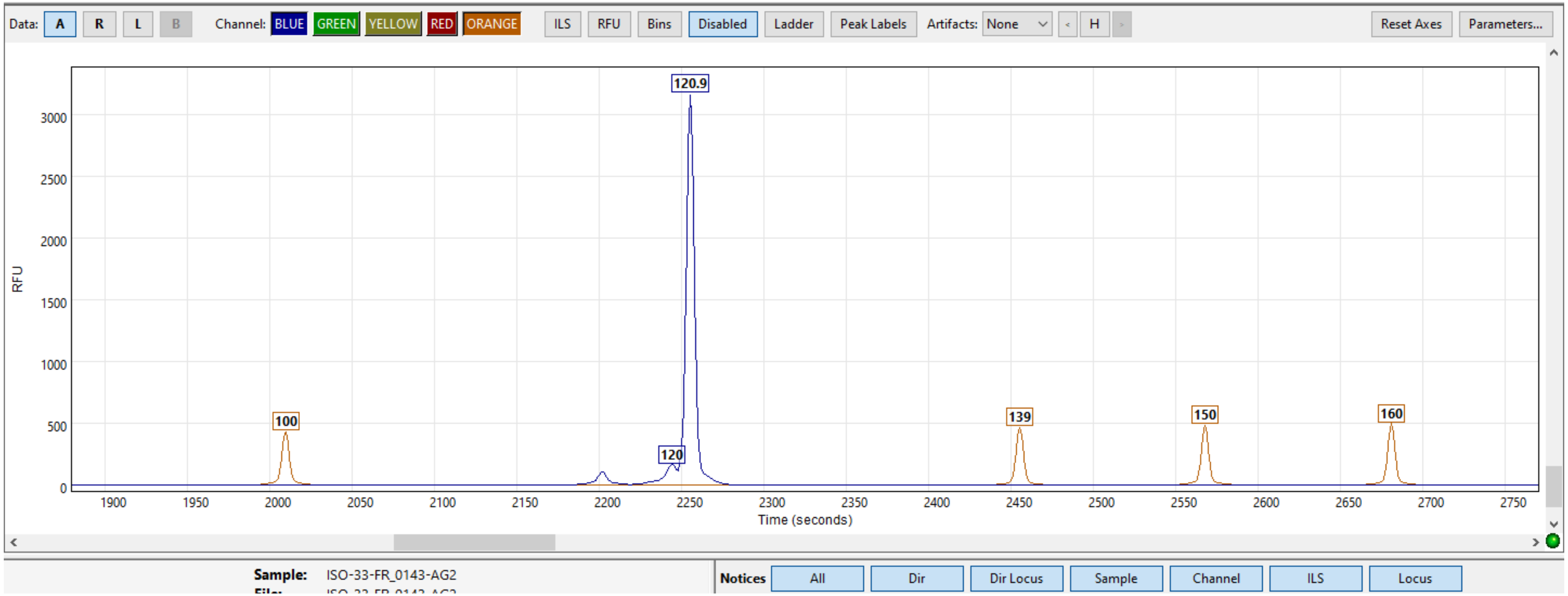


| | |
|---|---|
| **Observer 1** | **121** |
| **Observer 2** | **121** |
| **Observer 3** | **121** |

## AG4

## 1- Colony. Locus ISO AG4 sample 01 (0114)

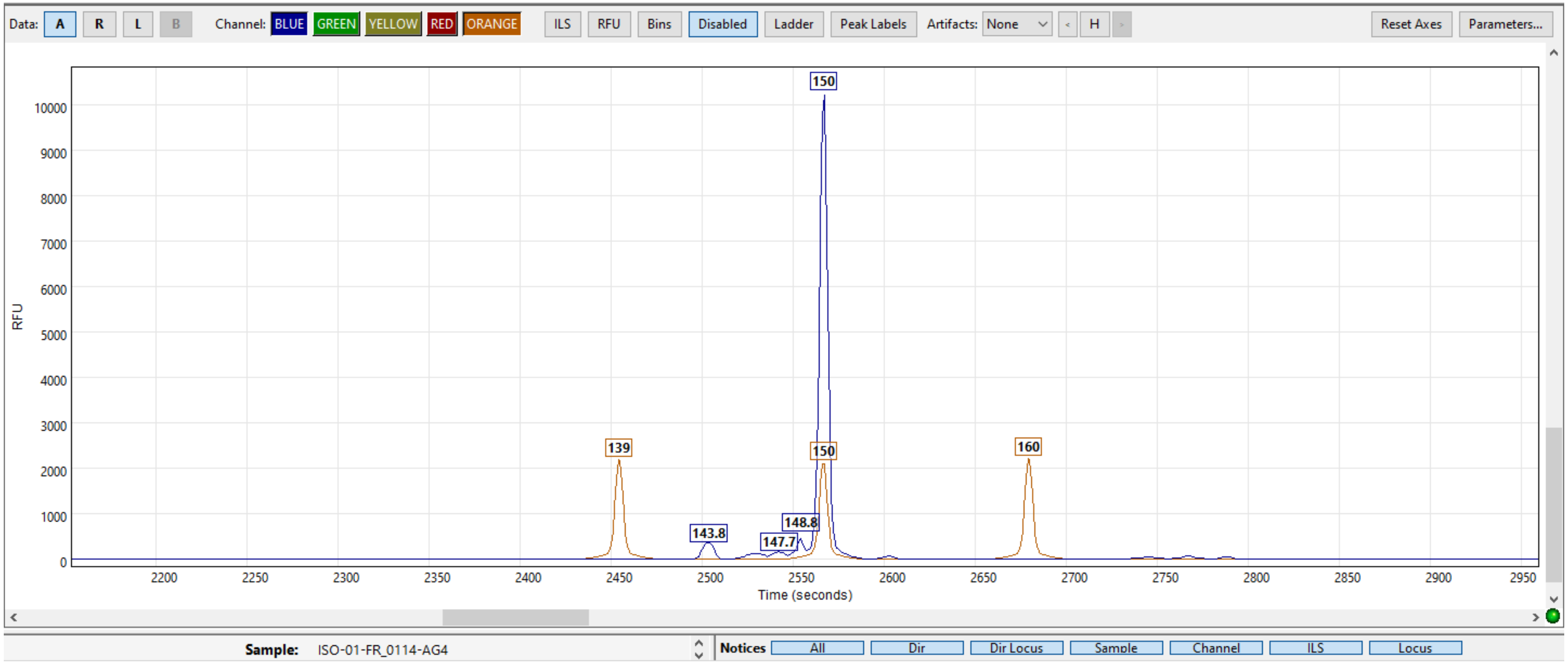


| | |
|---|---|
| **Observer 1** | **150** |
| **Observer 2** | **150** |
| **Observer 3** | **150** |

## 2- Colony. Locus ISO AG4 sample 02 (0115)

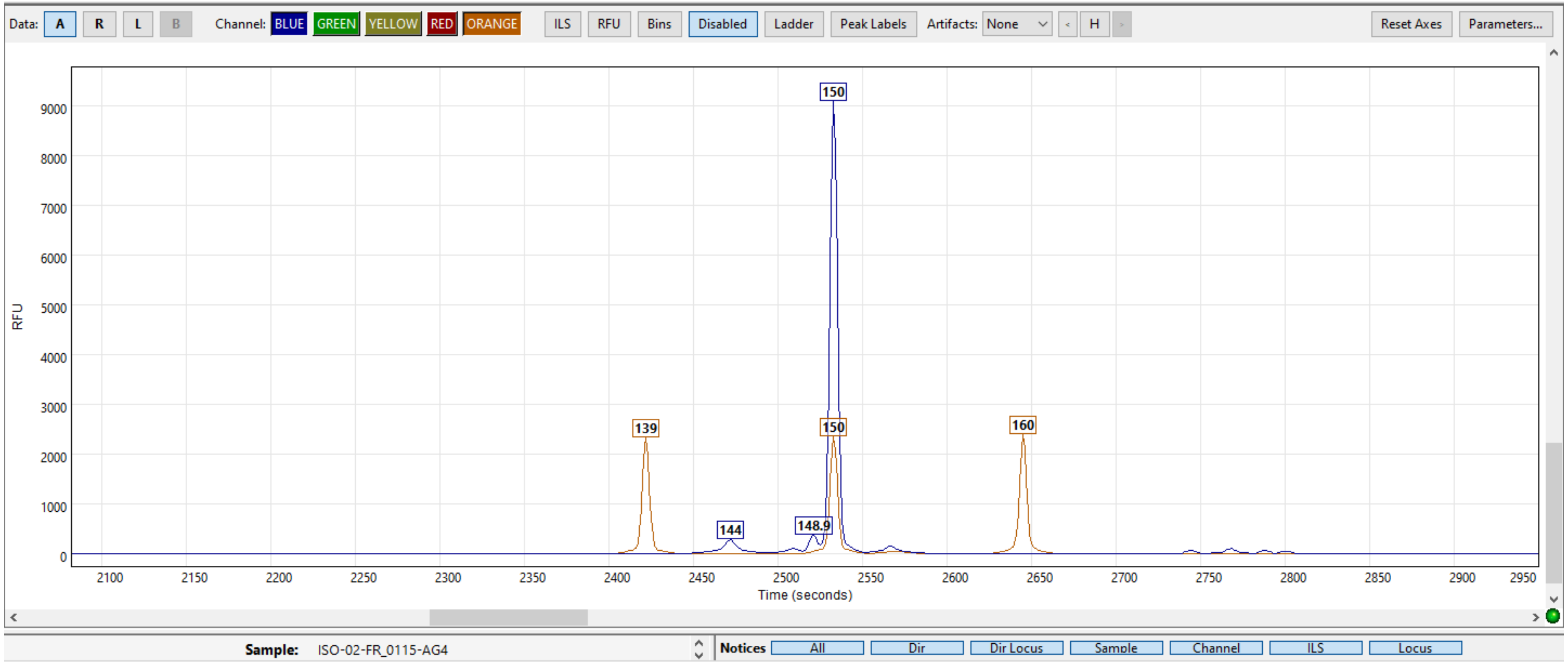


| | |
|---|---|
| **Observer 1** | **150** |
| **Observer 2** | **150** |
| **Observer 3** | **150** |

### 3- Colony. Locus ISO AG4 sample 03 (0116)

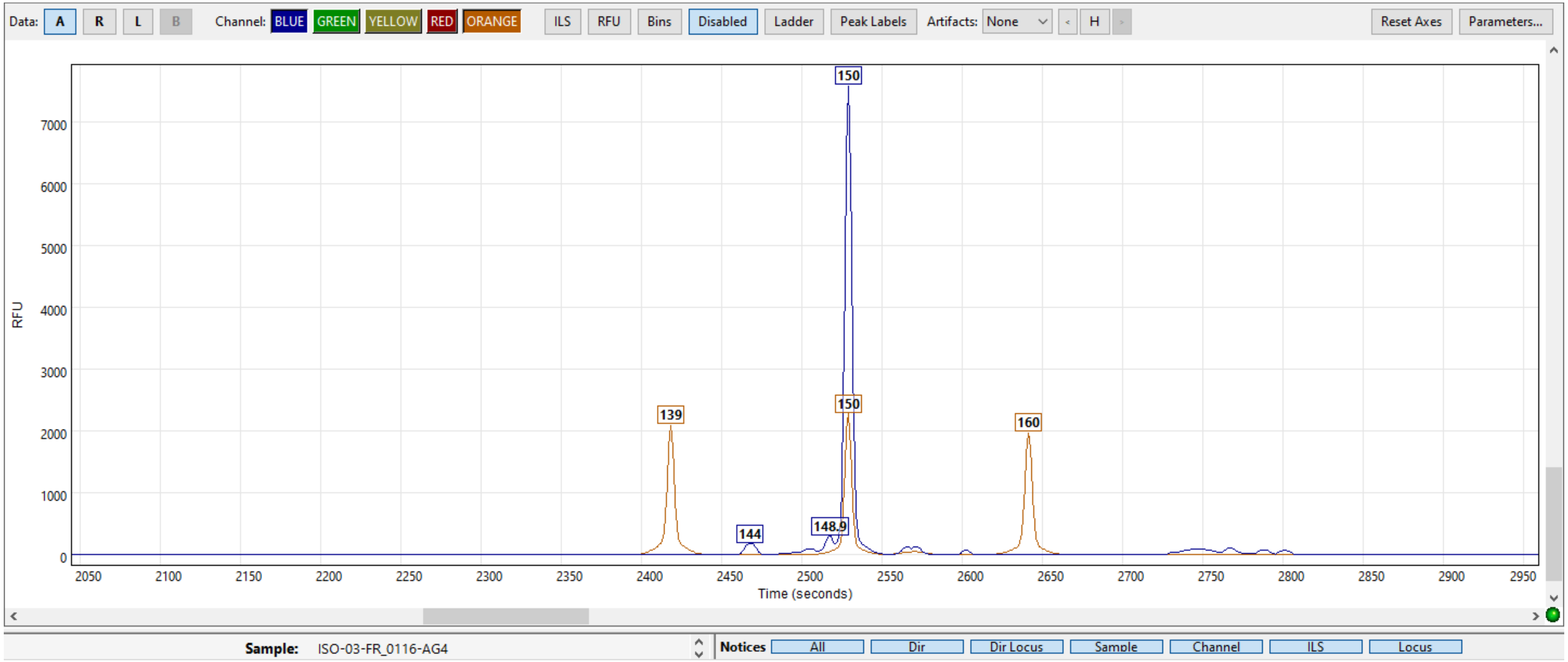


| | |
|---|---|
| **Observer 1** | **150** |
| **Observer 2** | **150** |
| **Observer 3** | **150** |

### 4- Colony. Locus ISO AG4 sample 04 (0117)

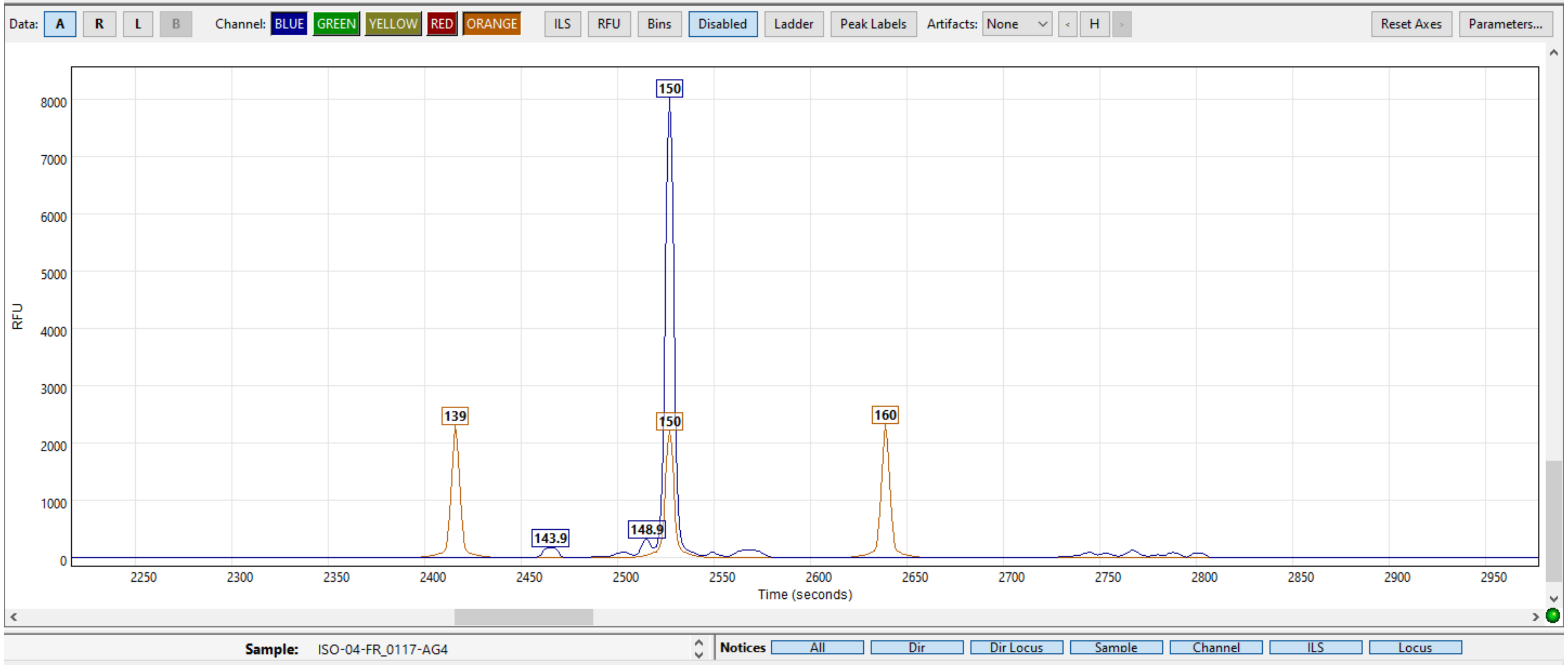


| | |
|---|---|
| **Observer 1** | **150** |
| **Observer 2** | **150** |
| **Observer 3** | **150** |

**5- Colony. Locus ISO AG4 sample 05 (0118)**

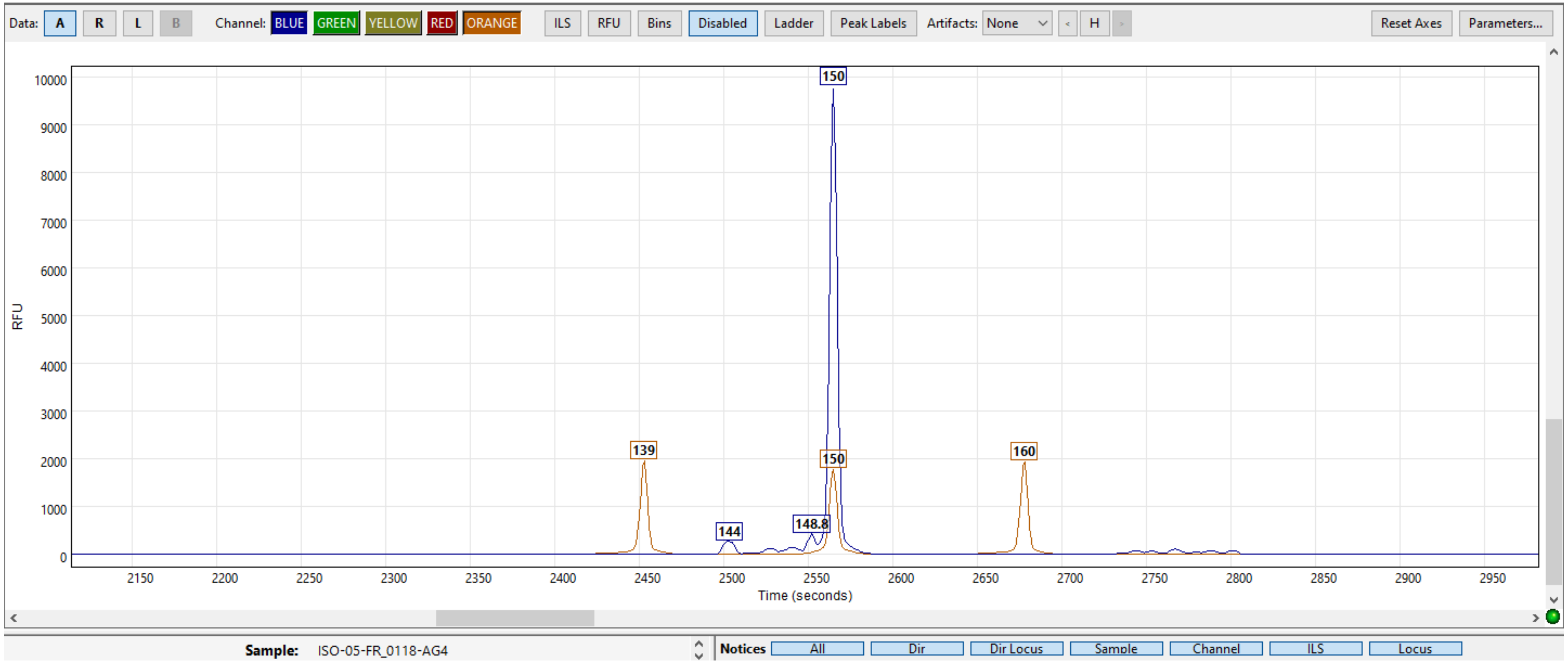


| | |
|---|---|
| **Observer 1** | **150** |
| **Observer 2** | **150** |
| **Observer 3** | **150** |

## 6- Colony. Locus ISO AG4 sample 06 (0119)

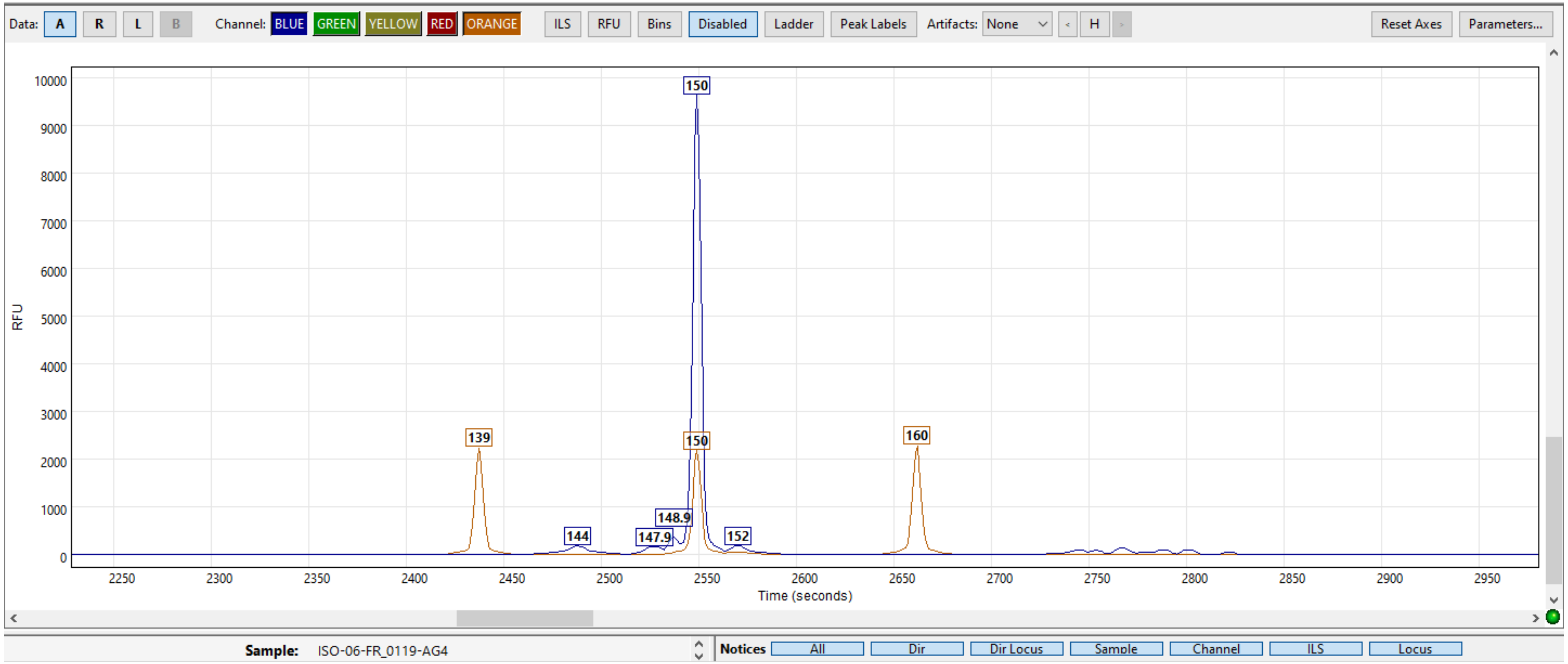


| | |
|---|---|
| **Observer 1** | **150** |
| **Observer 2** | **150** |
| **Observer 3** | **150** |

### 7- Colony. Locus ISO AG4 sample 07 (0120)

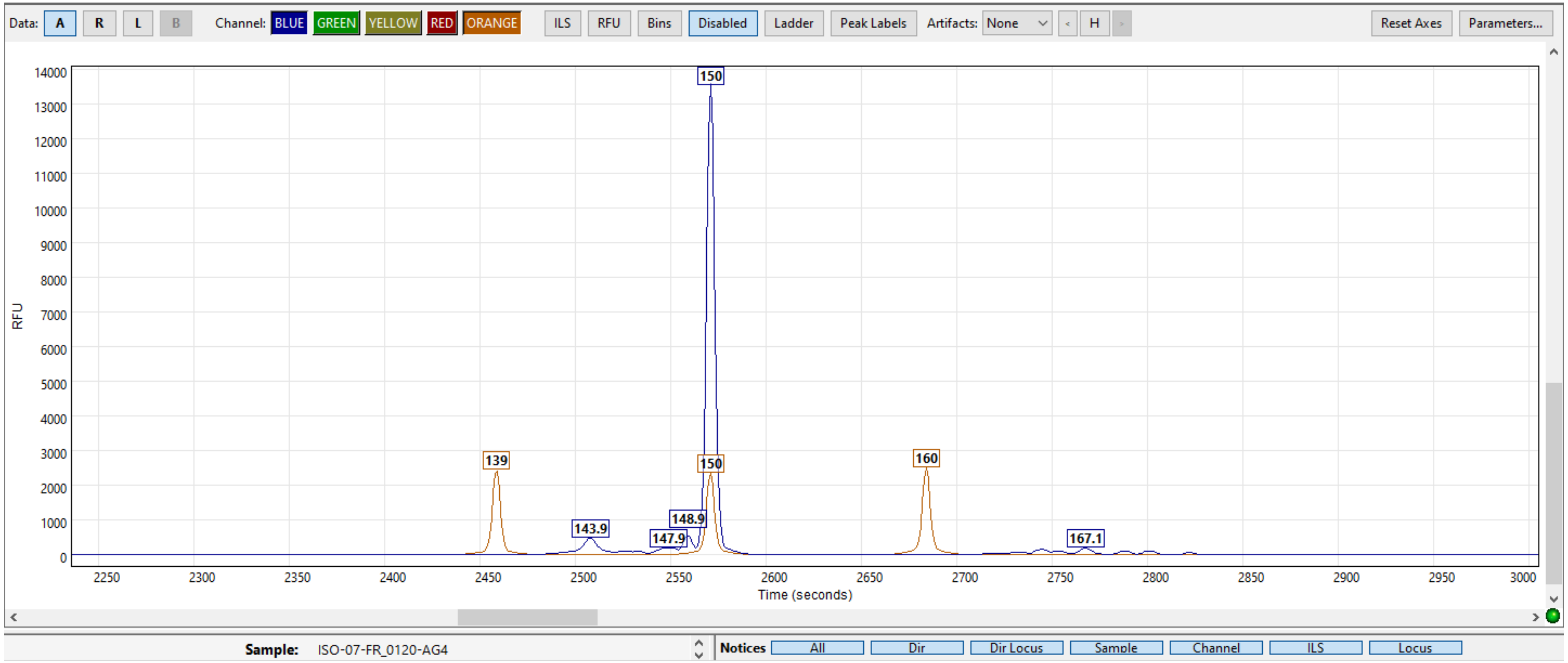


| | |
|---|---|
| **Observer 1** | **150** |
| **Observer 2** | **150** |
| **Observer 3** | **150** |

## 8- Colony. Locus ISO AG4 sample 08 (0121)

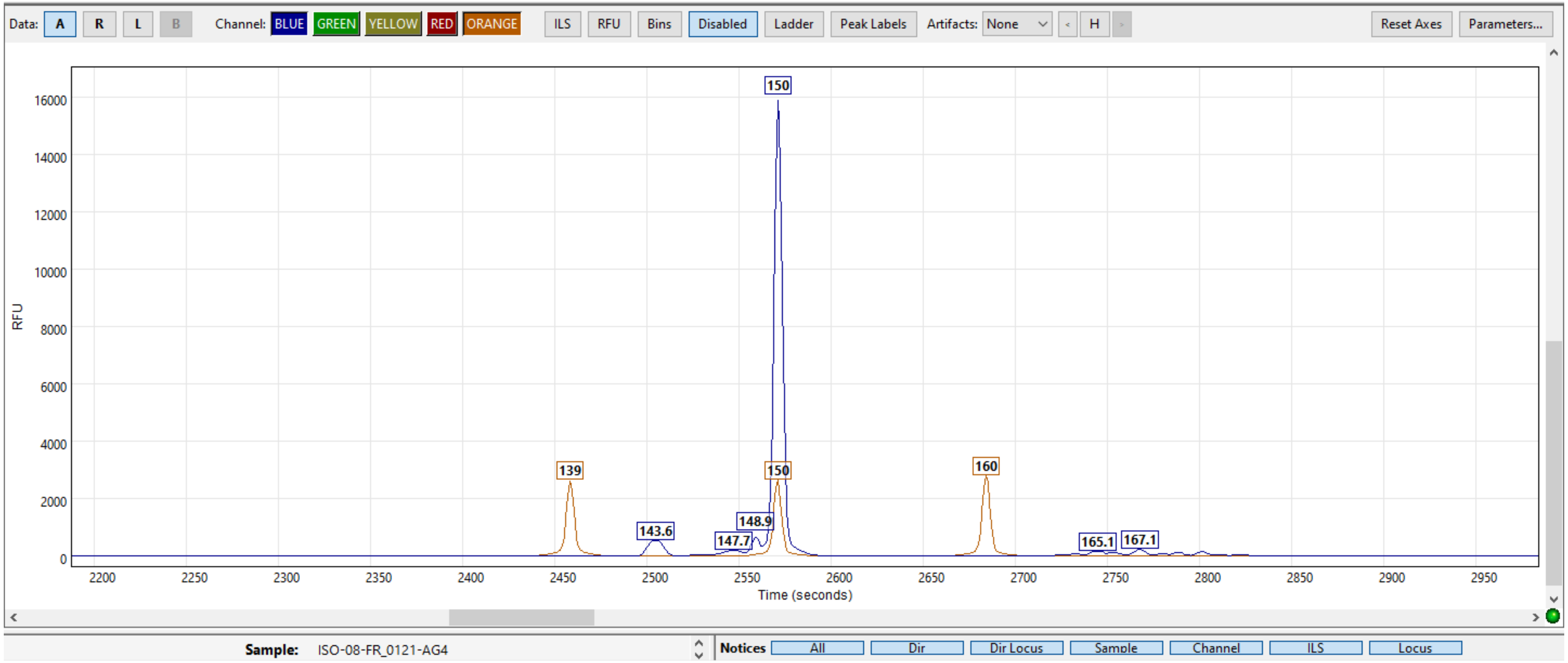


| | |
|---|---|
| **Observer 1** | **150** |
| **Observer 2** | **150** |
| **Observer 3** | **150** |

**9- Colony. Locus ISO AG4 sample 09 (0122)**

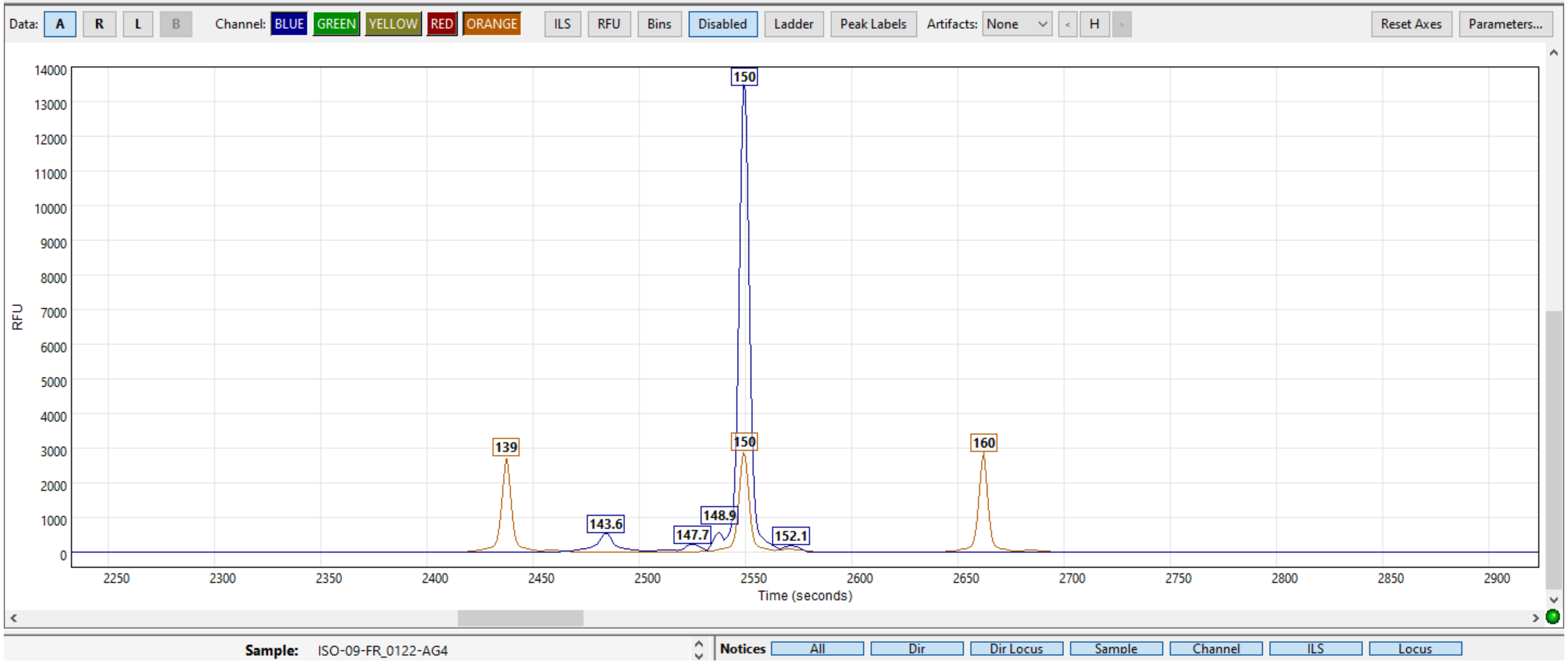


| | |
|---|---|
| **Observer 1** | **150** |
| **Observer 2** | **150** |
| **Observer 3** | **150** |

**10- Colony. Locus ISO AG4 sample 10 (0123)**

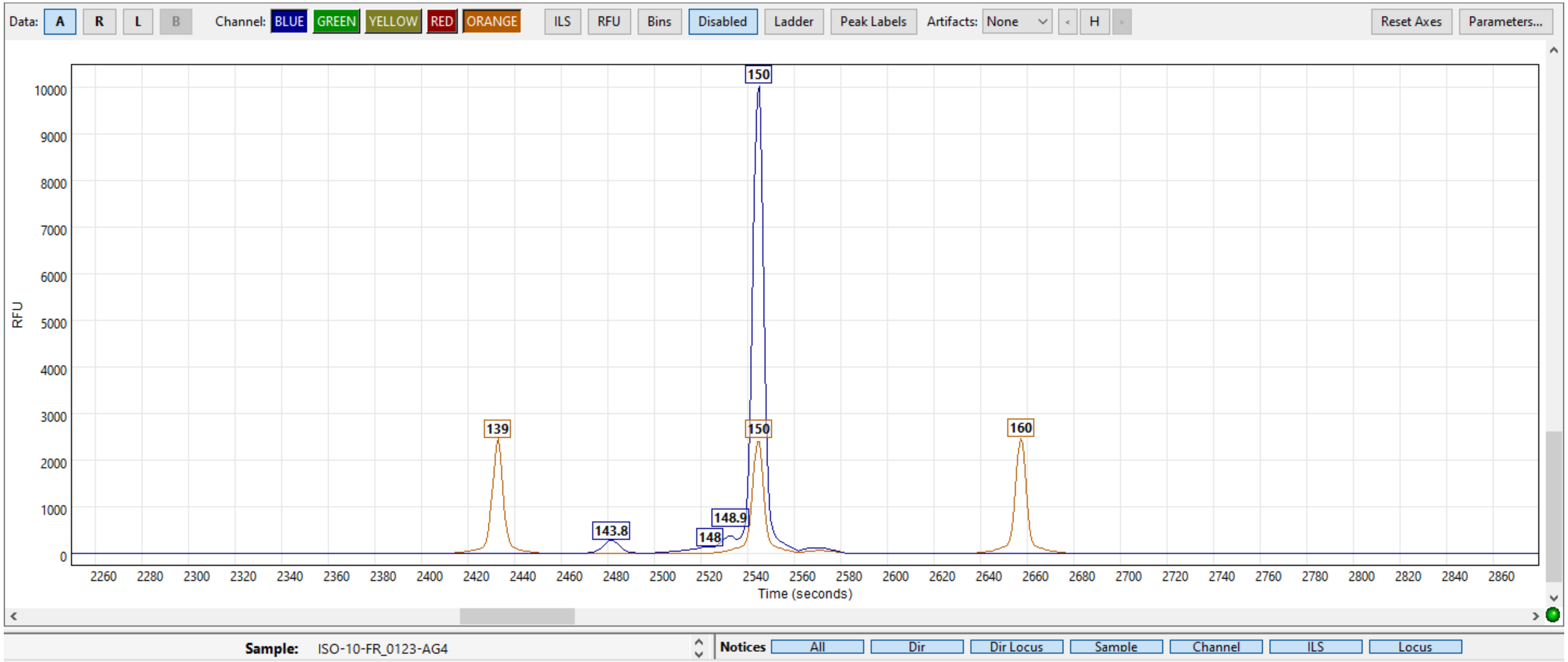


| | |
|---|---|
| **Observer 1** | **150** |
| **Observer 2** | **150** |
| **Observer 3** | **150** |

## 11- Colony. Locus ISO AG4 sample 11 (0124)

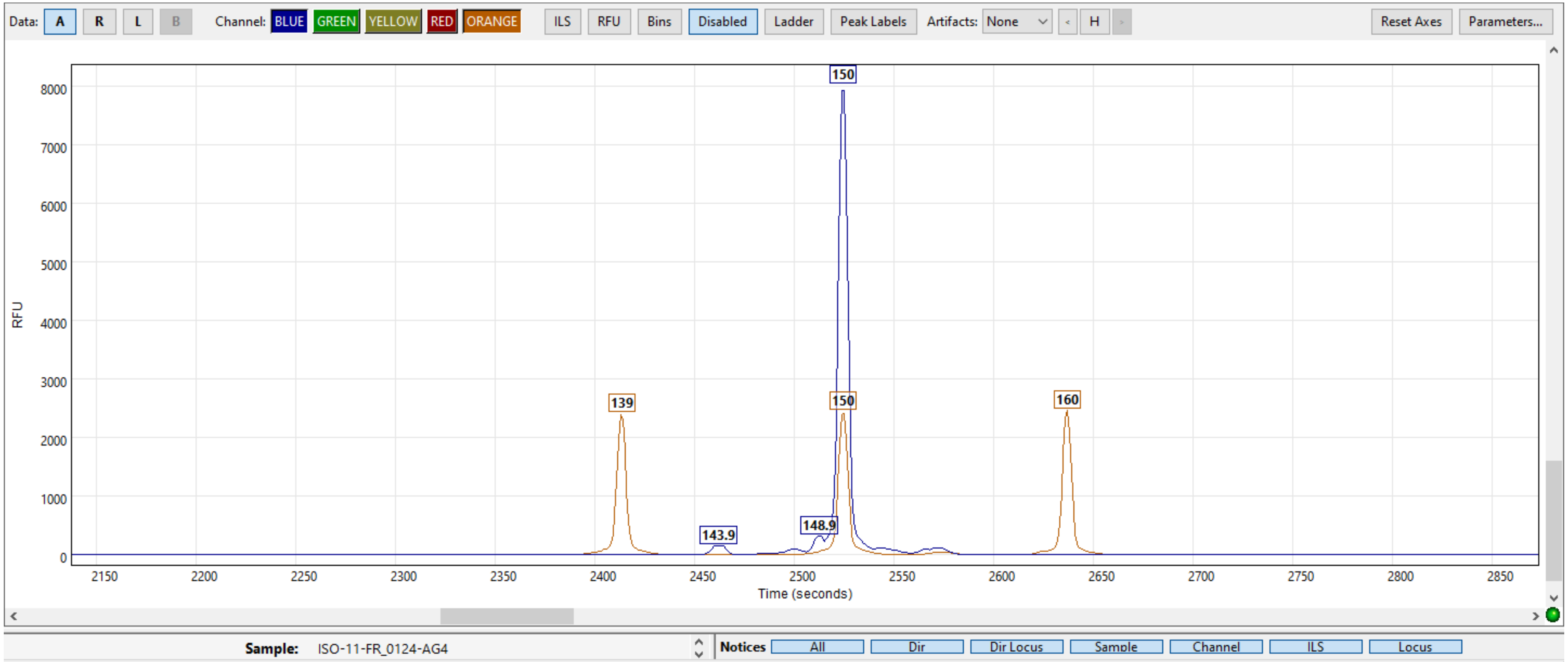


| | |
|---|---|
| **Observer 1** | **150** |
| **Observer 2** | **150** |
| **Observer 3** | **150** |

**12- Colony. Locus ISO AG4 sample 12 (0125)**

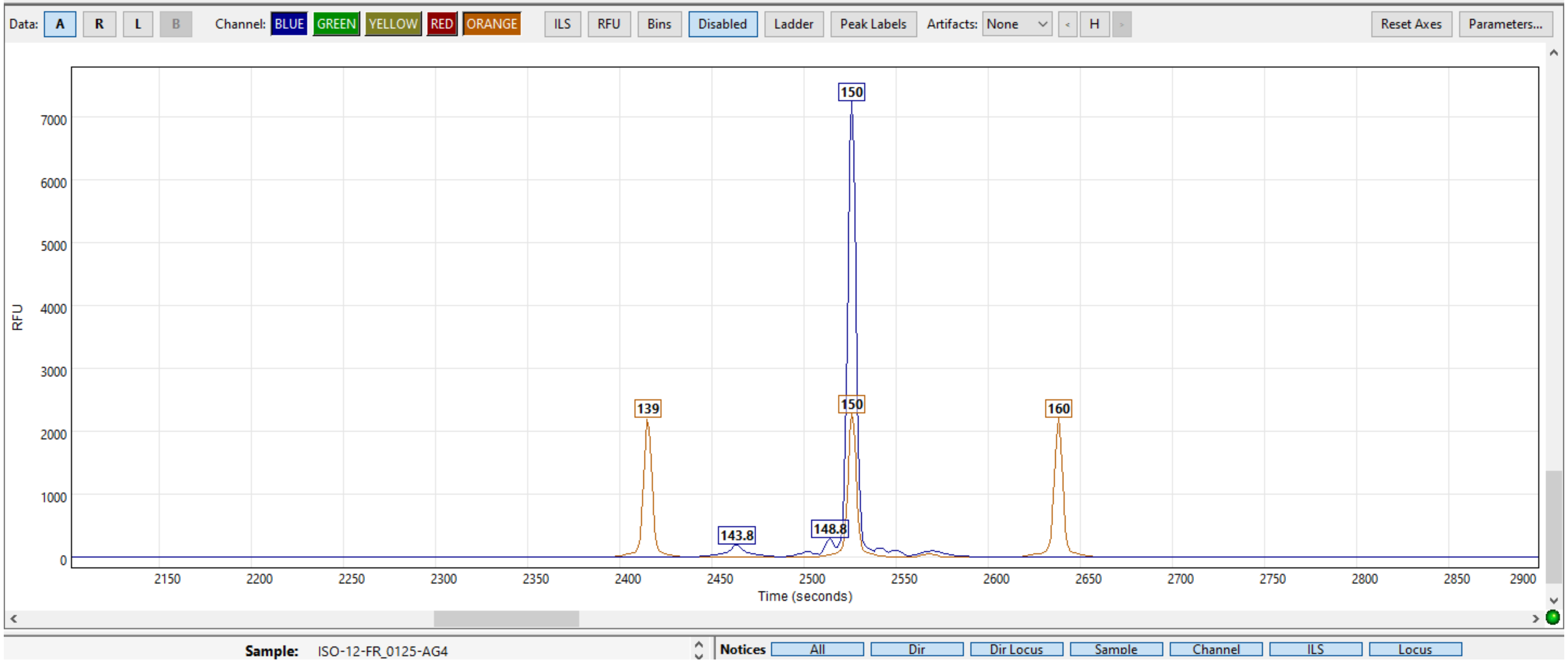


| | |
|---|---|
| **Observer 1** | **150** |
| **Observer 2** | **150** |
| **Observer 3** | **150** |

**13- Colony. Locus ISO AG4 sample 13 (0126)**

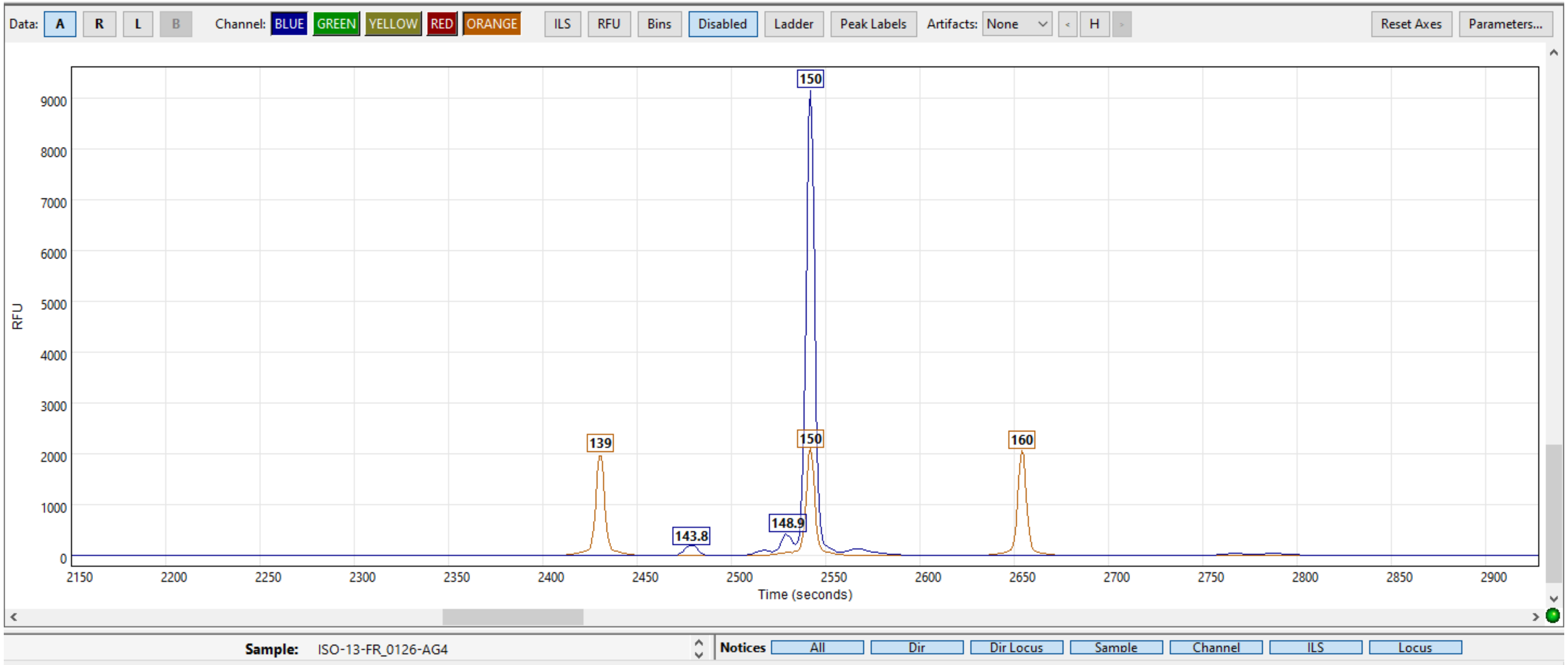


| | |
|---|---|
| **Observer 1** | **150** |
| **Observer 2** | **150** |
| **Observer 3** | **150** |

**14- Colony. Locus ISO AG4 sample 14 (0127)**

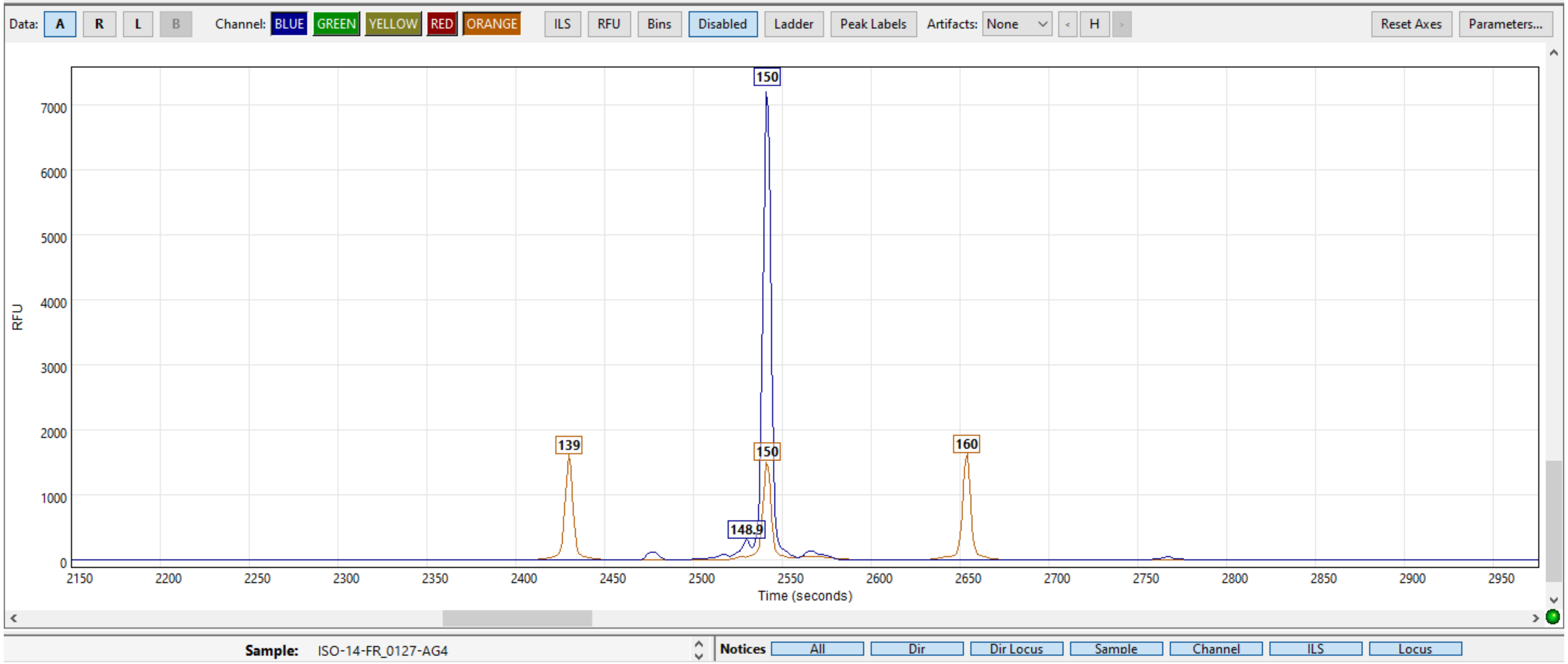


| | |
|---|---|
| **Observer 1** | **150** |
| **Observer 2** | **150** |
| **Observer 3** | **150** |

### 15- Colony. Locus ISO AG4 sample 15 (0128)

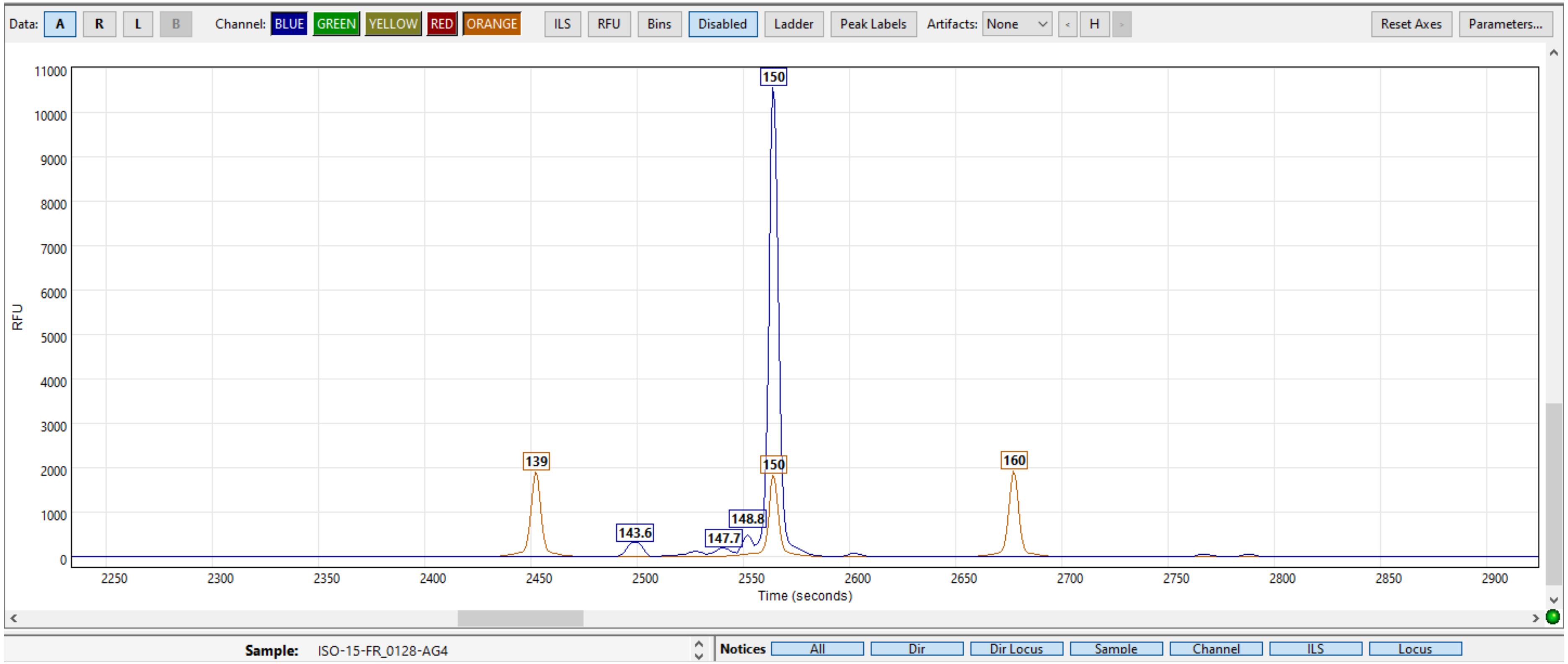


| | |
|---|---|
| **Observer 1** | **150** |
| **Observer 2** | **150** |
| **Observer 3** | **150** |

## 16- Colony. Locus ISO AG4 sample 16 (0129)

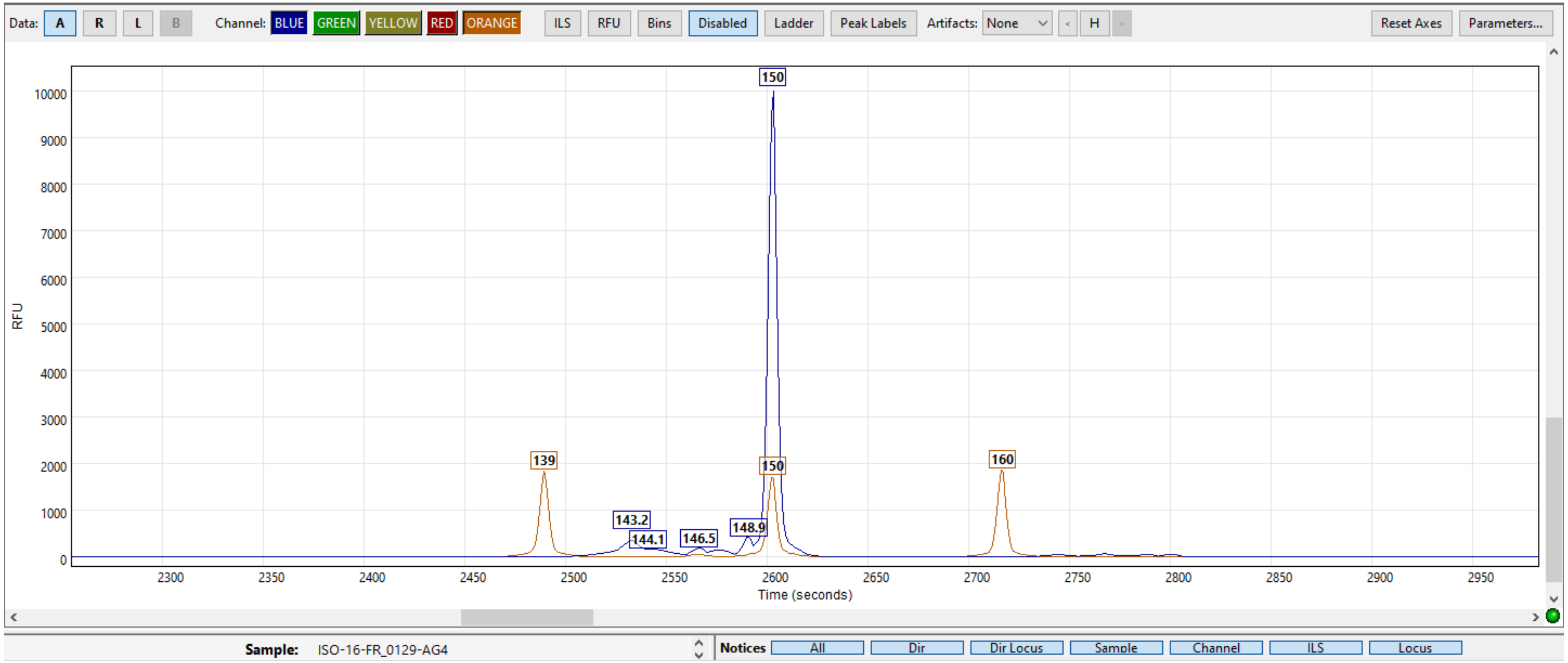


| | |
|---|---|
| **Observer 1** | **150** |
| **Observer 2** | **150** |
| **Observer 3** | **150** |

**17- Colony. Locus ISO AG4 sample 17 (0130)**

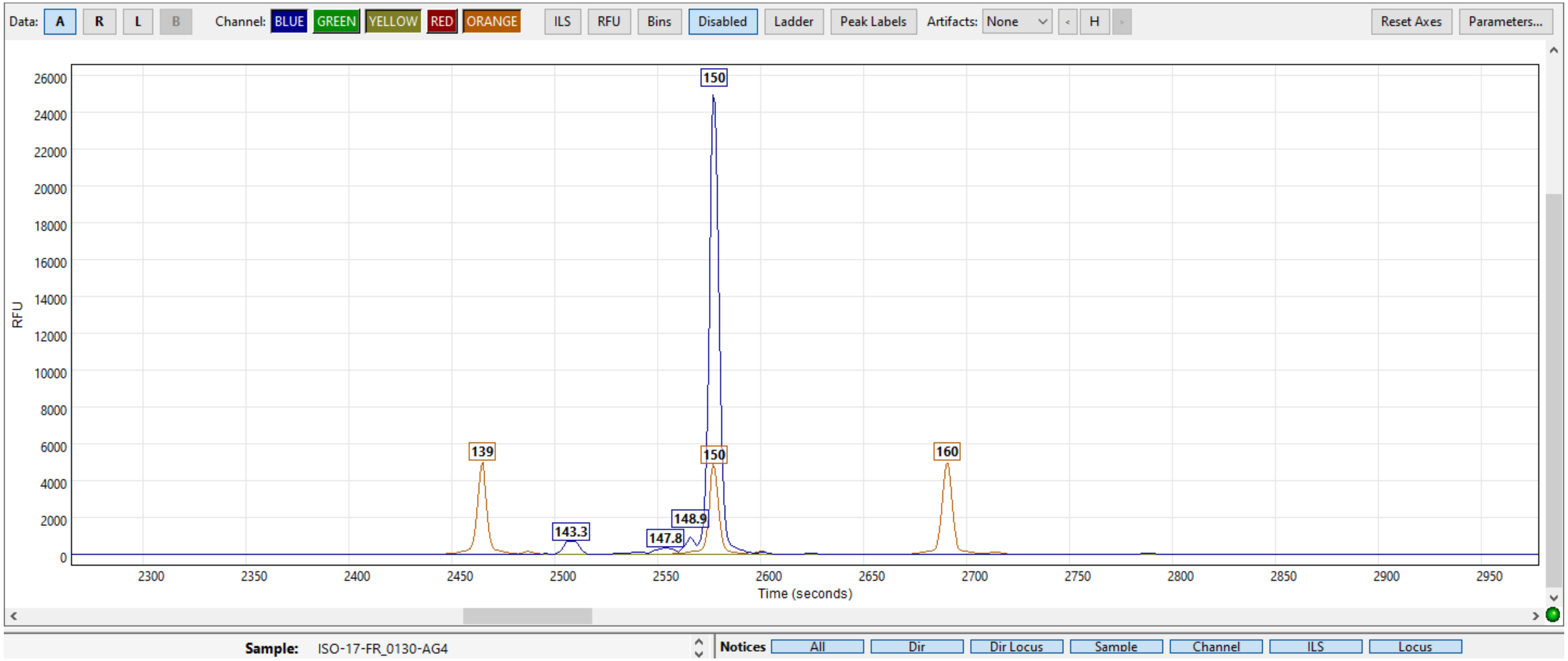


| | |
|---|---|
| **Observer 1** | **150** |
| **Observer 2** | **150** |
| **Observer 3** | **150** |

**18- Colony. Locus ISO AG4 sample 19 (0131)**

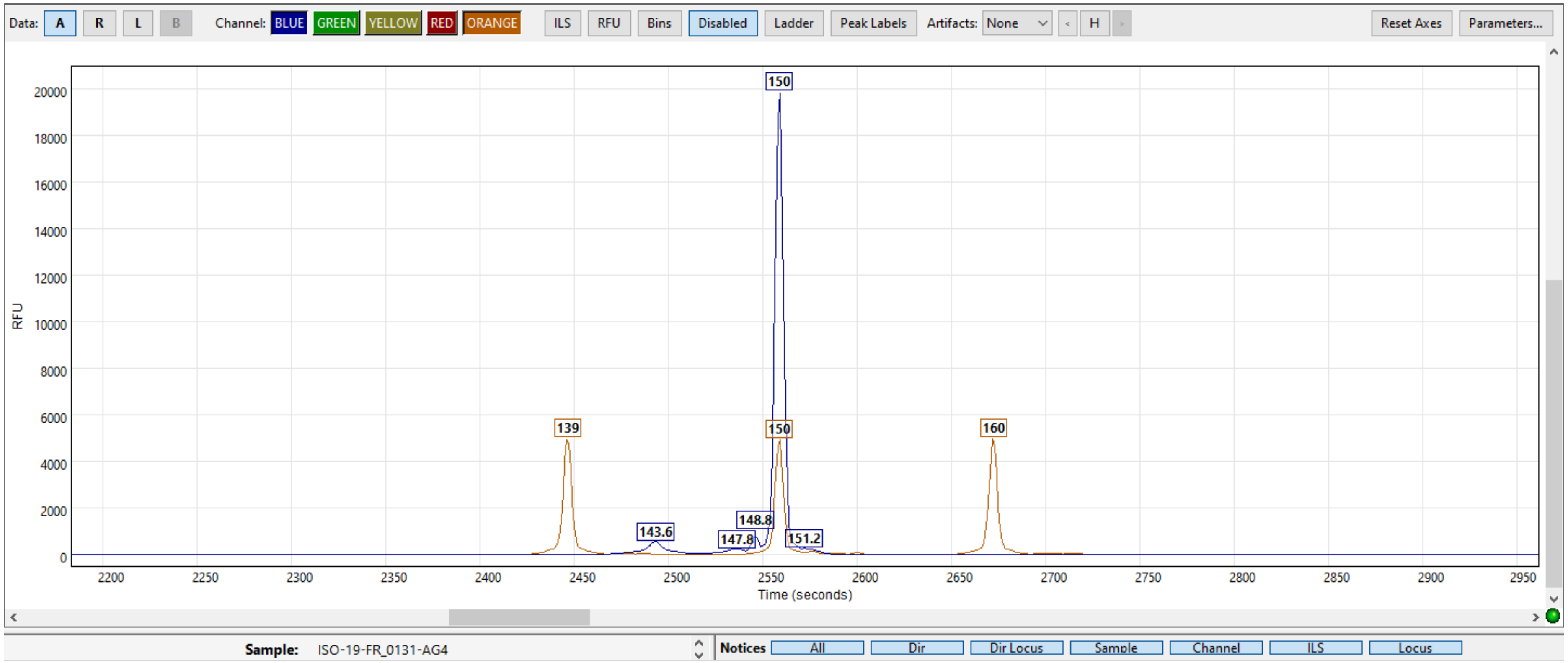


| | |
|---|---|
| **Observer 1** | **150** |
| **Observer 2** | **150** |
| **Observer 3** | **150** |

**19- Colony. Locus ISO AG4 sample 20 (0132)**

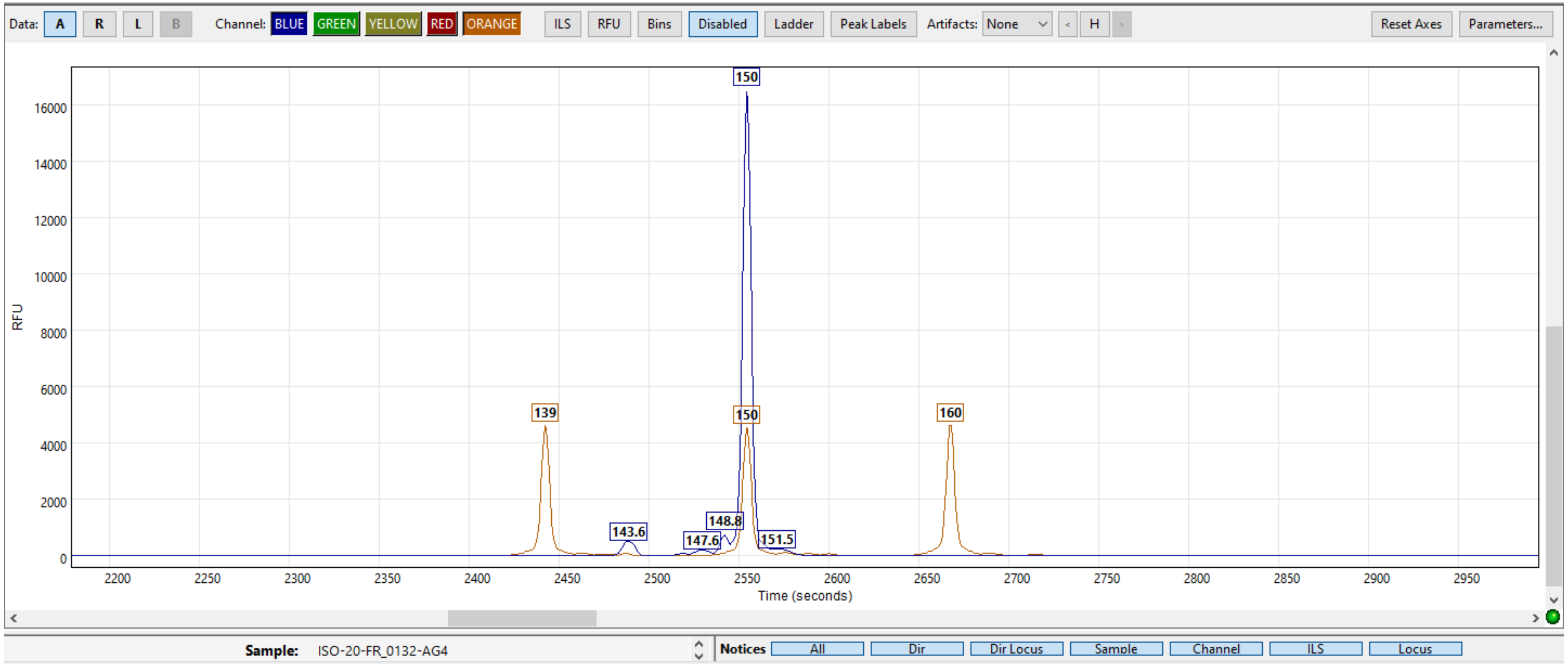


| | |
|---|---|
| **Observer 1** | **150** |
| **Observer 2** | **150** |
| **Observer 3** | **150** |

## 20- Colony. Locus ISO AG4 sample 21 (0133)

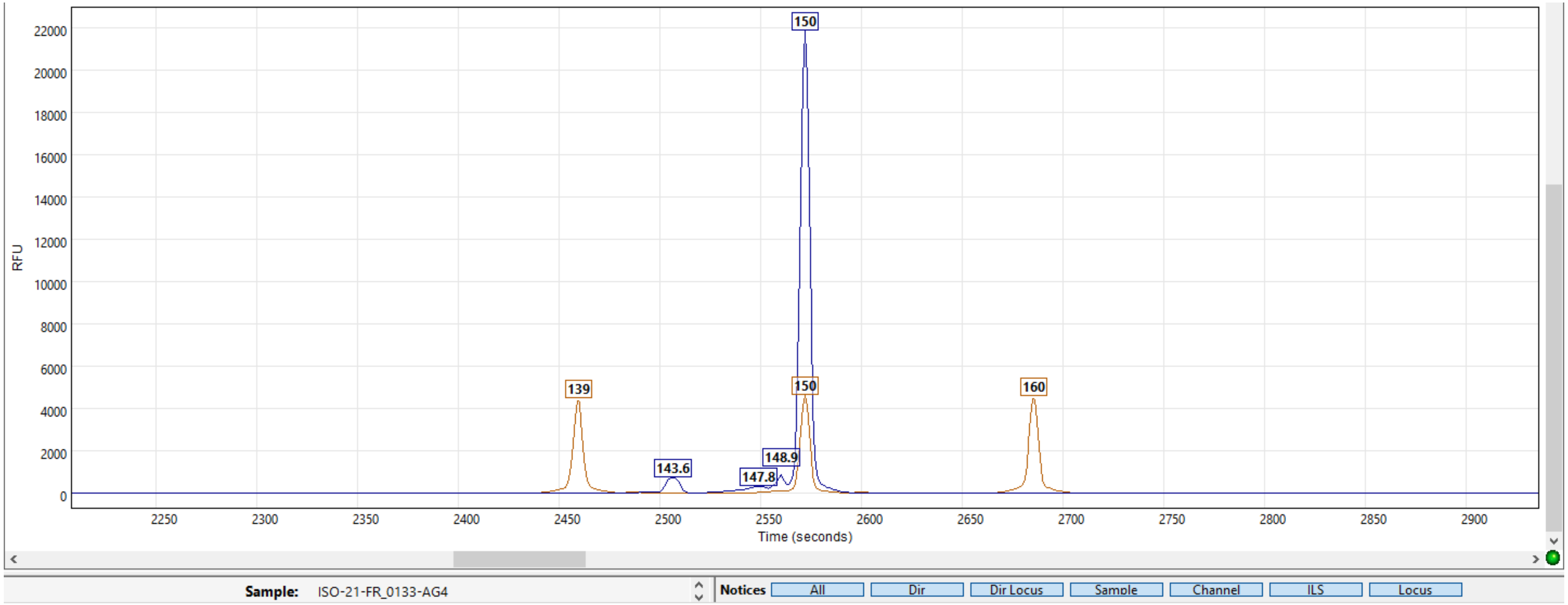


| | |
|---|---|
| **Observer 1** | **150** |
| **Observer 2** | **150** |
| **Observer 3** | **150** |

**21- Colony. Locus ISO AG4 sample 23 (0134)**

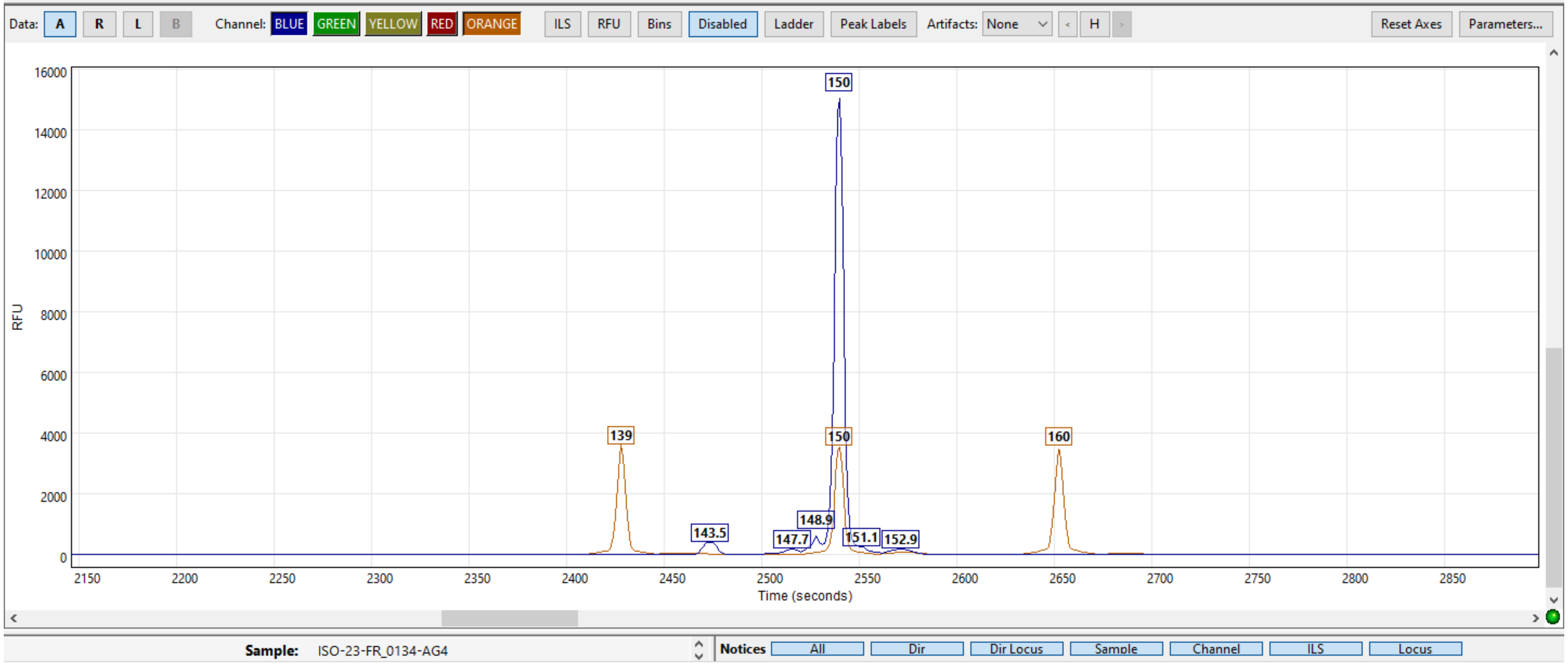


| | |
|---|---|
| **Observer 1** | **150** |
| **Observer 2** | **150** |
| **Observer 3** | **150** |

## 22- Colony. Locus ISO AG4 sample 24 (0135)

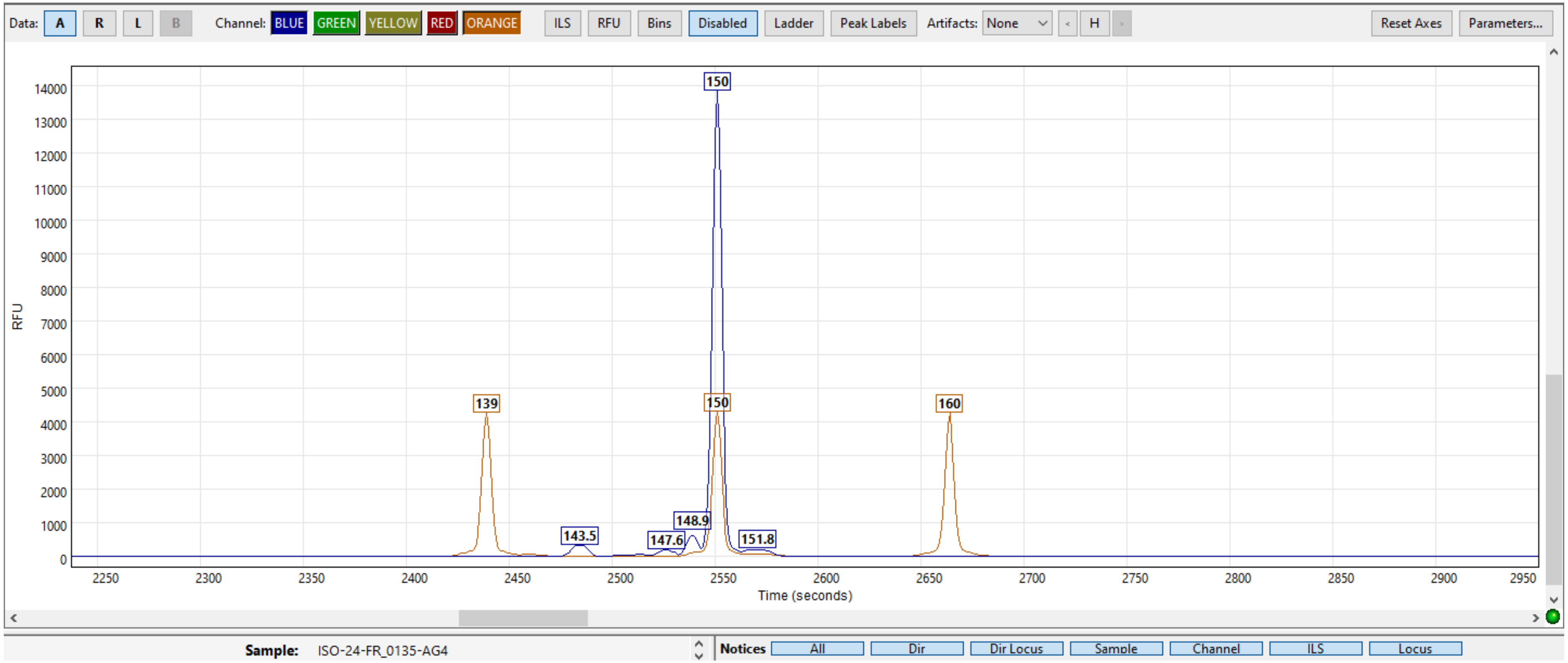


| | |
|---|---|
| **Observer 1** | **150** |
| **Observer 2** | **150** |
| **Observer 3** | **150** |

## 23- Colony. Locus ISO AG4 sample 25 (0136)

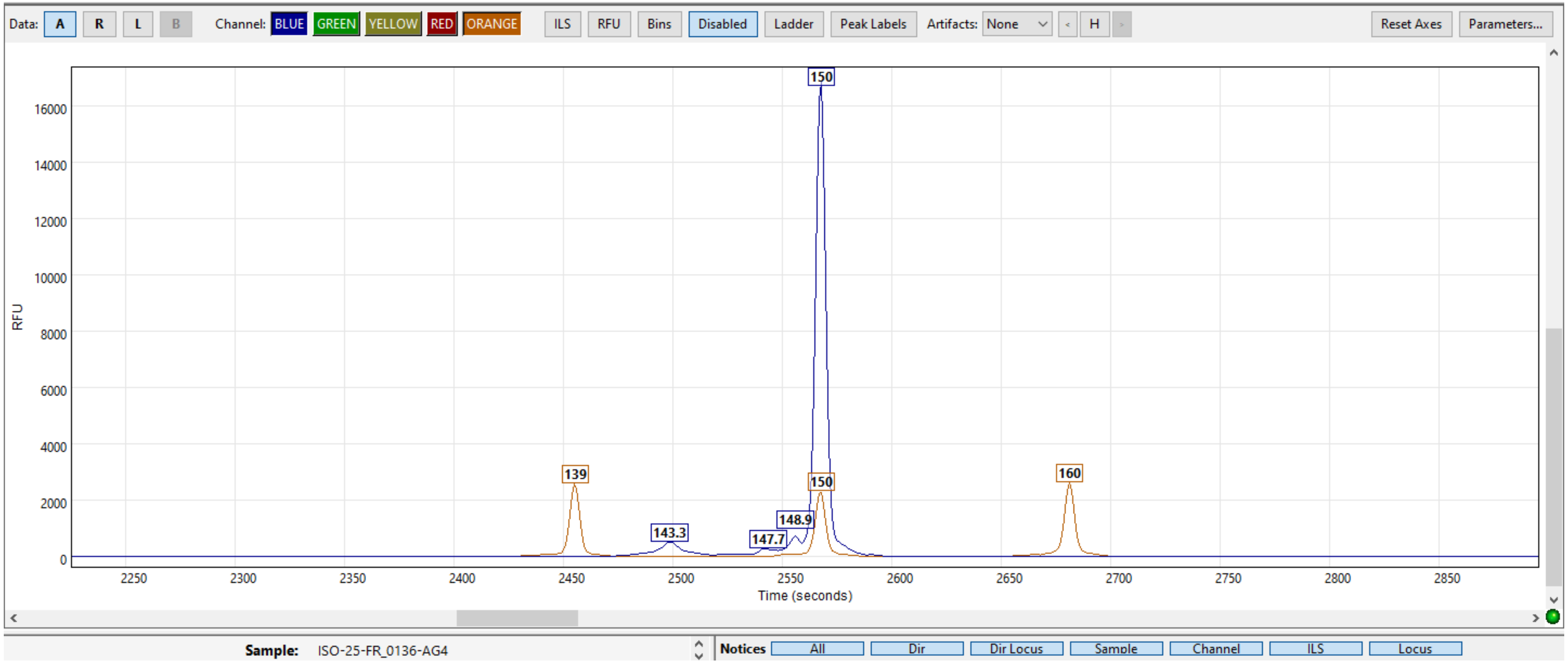


| | |
|---|---|
| **Observer 1** | **150** |
| **Observer 2** | **150** |
| **Observer 3** | **150** |

**24- Colony. Locus ISO AG4 sample 26 (0137)**

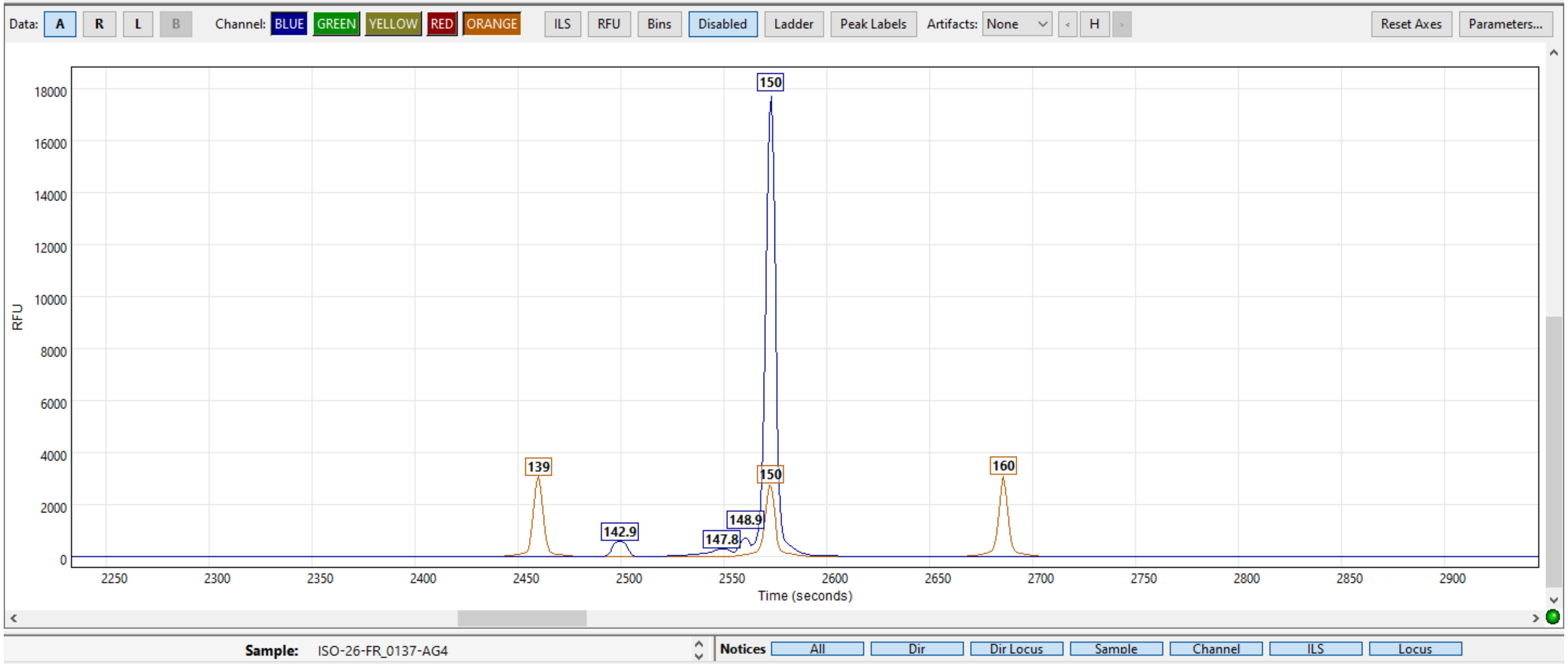


| | |
|---|---|
| **Observer 1** | **150** |
| **Observer 2** | **150** |
| **Observer 3** | **150** |

## 25- Colony. Locus ISO AG4 sample 27 (0138)

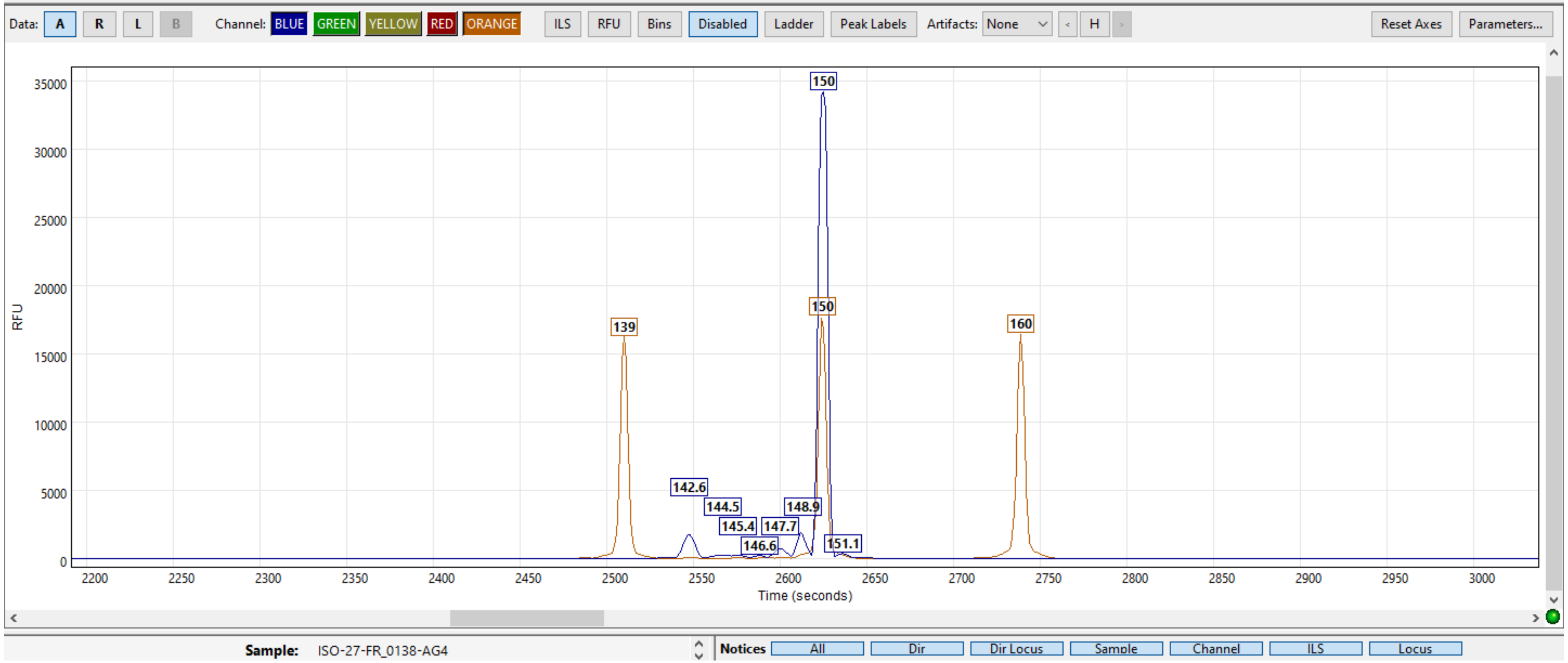


| | |
|---|---|
| **Observer 1** | **150** |
| **Observer 2** | **150** |
| **Observer 3** | **150** |

**26- Colony. Locus ISO AG4 sample 29 (0139)**

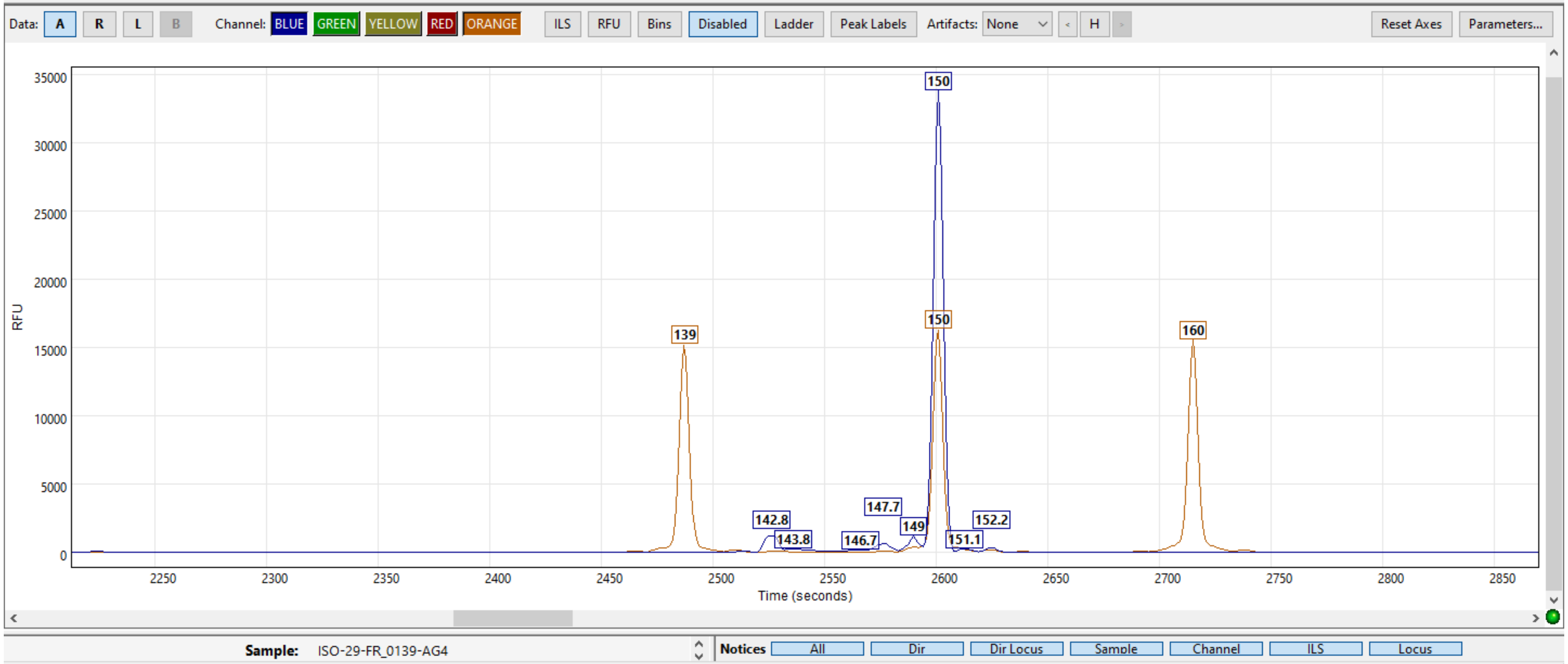


| | |
|---|---|
| **Observer 1** | **150** |
| **Observer 2** | **150** |
| **Observer 3** | **150** |

## 27- Colony. Locus ISO AG4 sample 30 (0140)

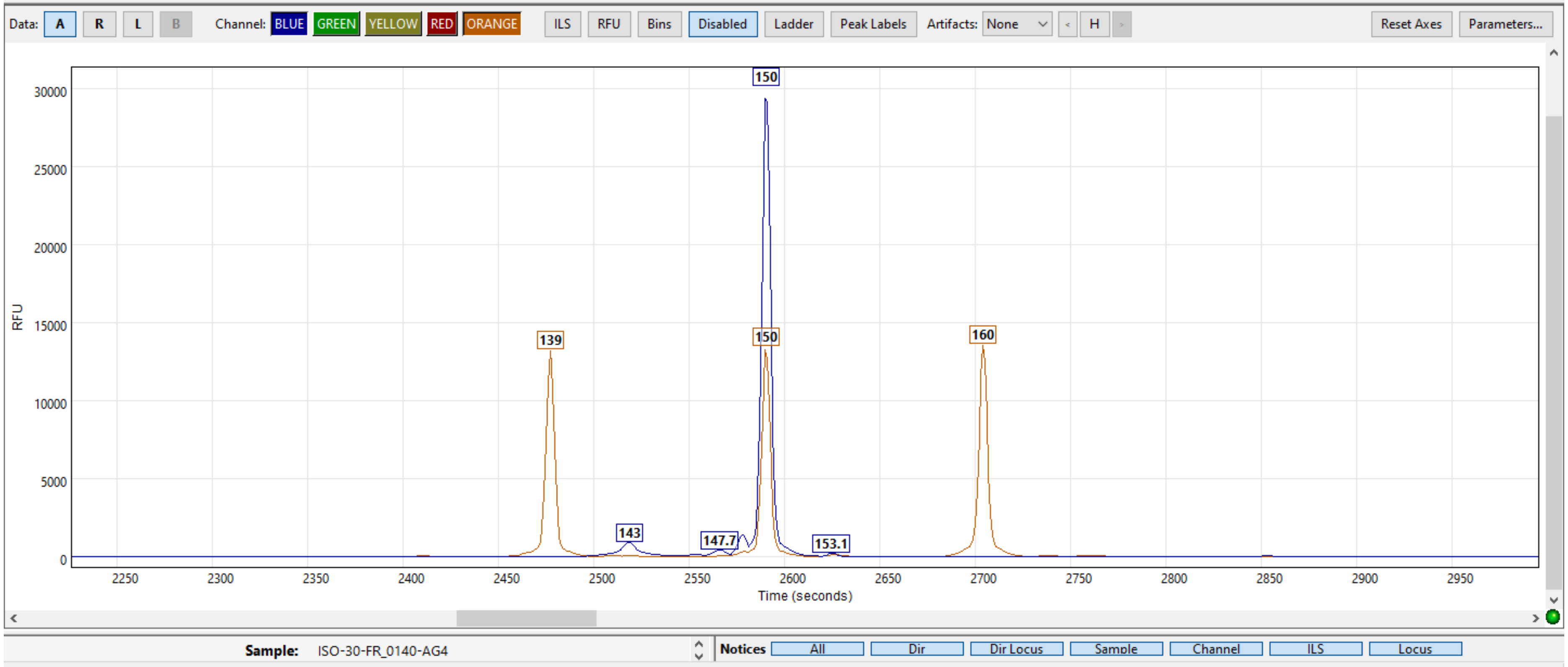


| | |
|---|---|
| **Observer 1** | **150** |
| **Observer 2** | **150** |
| **Observer 3** | **150** |

**28- Colony. Locus ISO AG4 sample 31 (0141)**

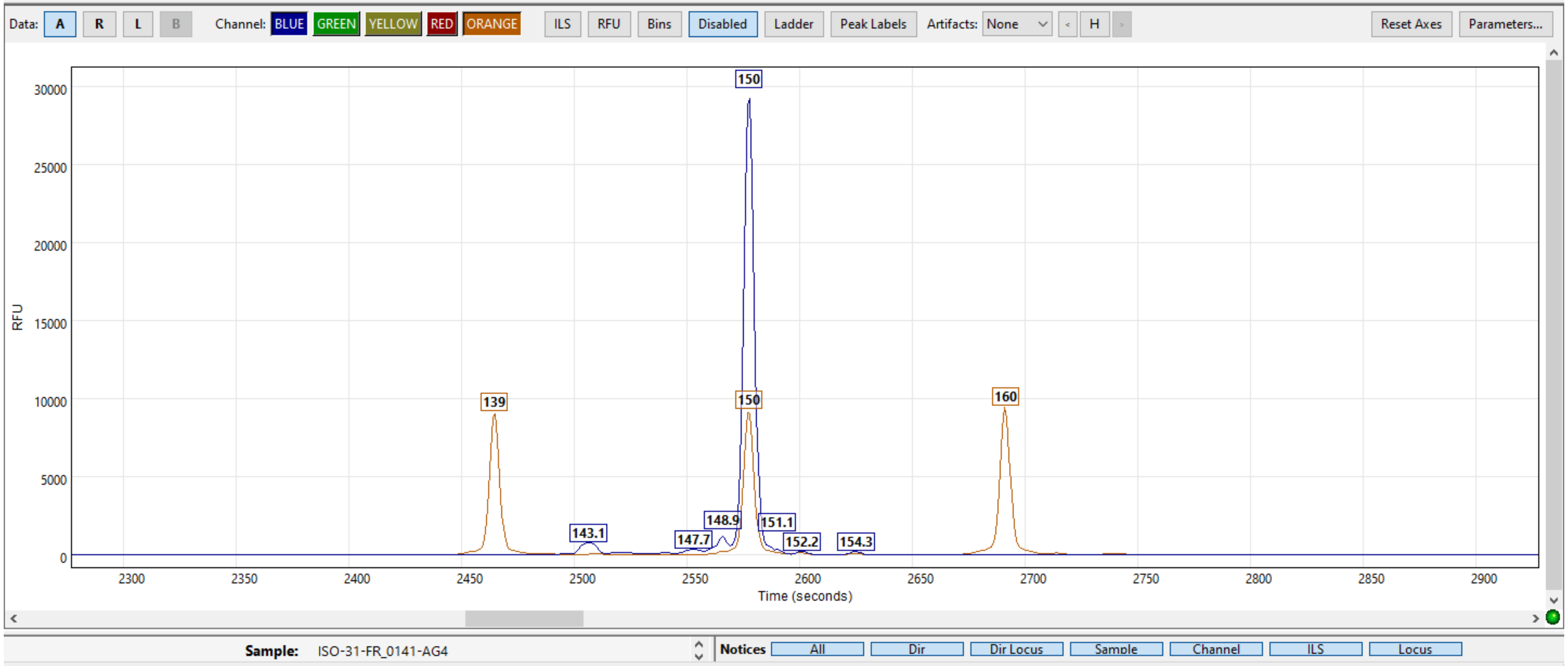


| | |
|---|---|
| **Observer 1** | **150** |
| **Observer 2** | **150** |
| **Observer 3** | **150** |

29- Colony. Locus ISO AG4 sample 32 (0142)

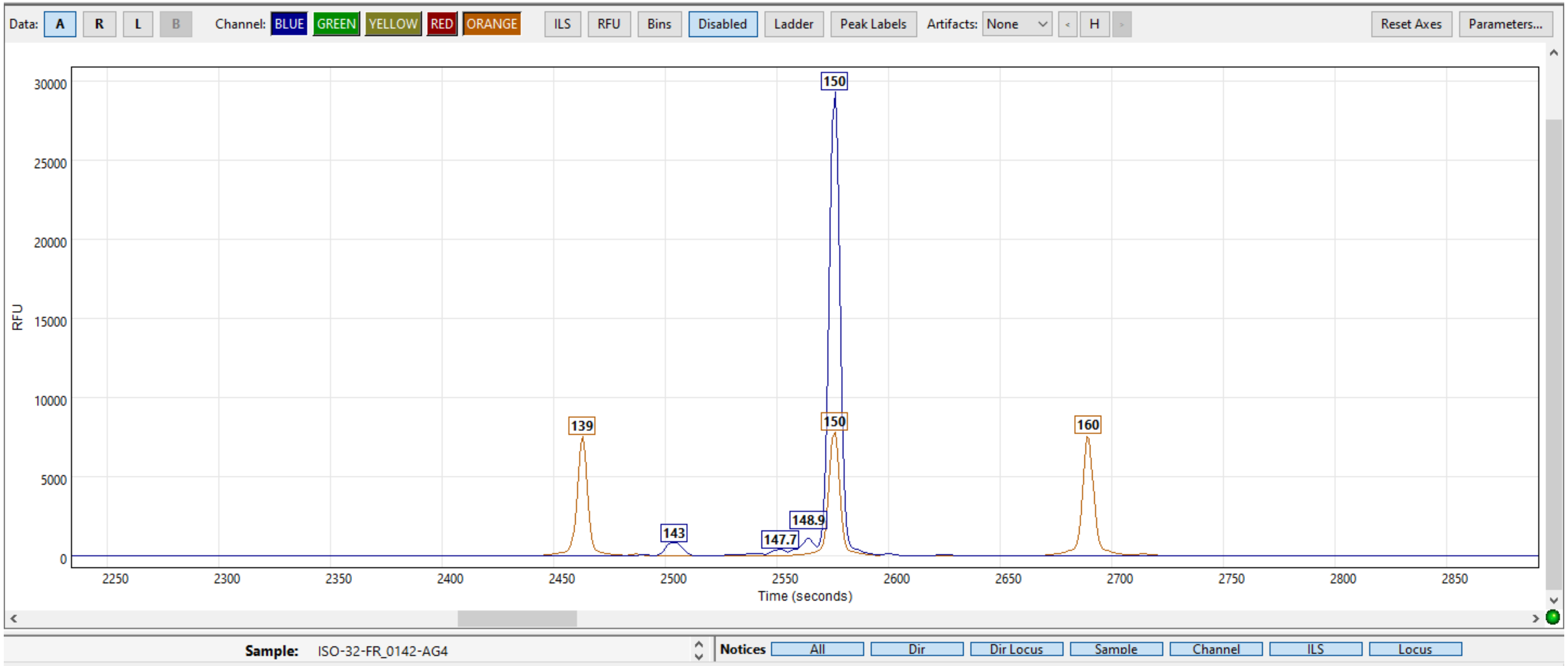


| | |
|---|---|
| **Observer 1** | **150** |
| **Observer 2** | **150** |
| **Observer 3** | **150** |

**30- Colony. Locus ISO AG4 sample 33 (0143)**

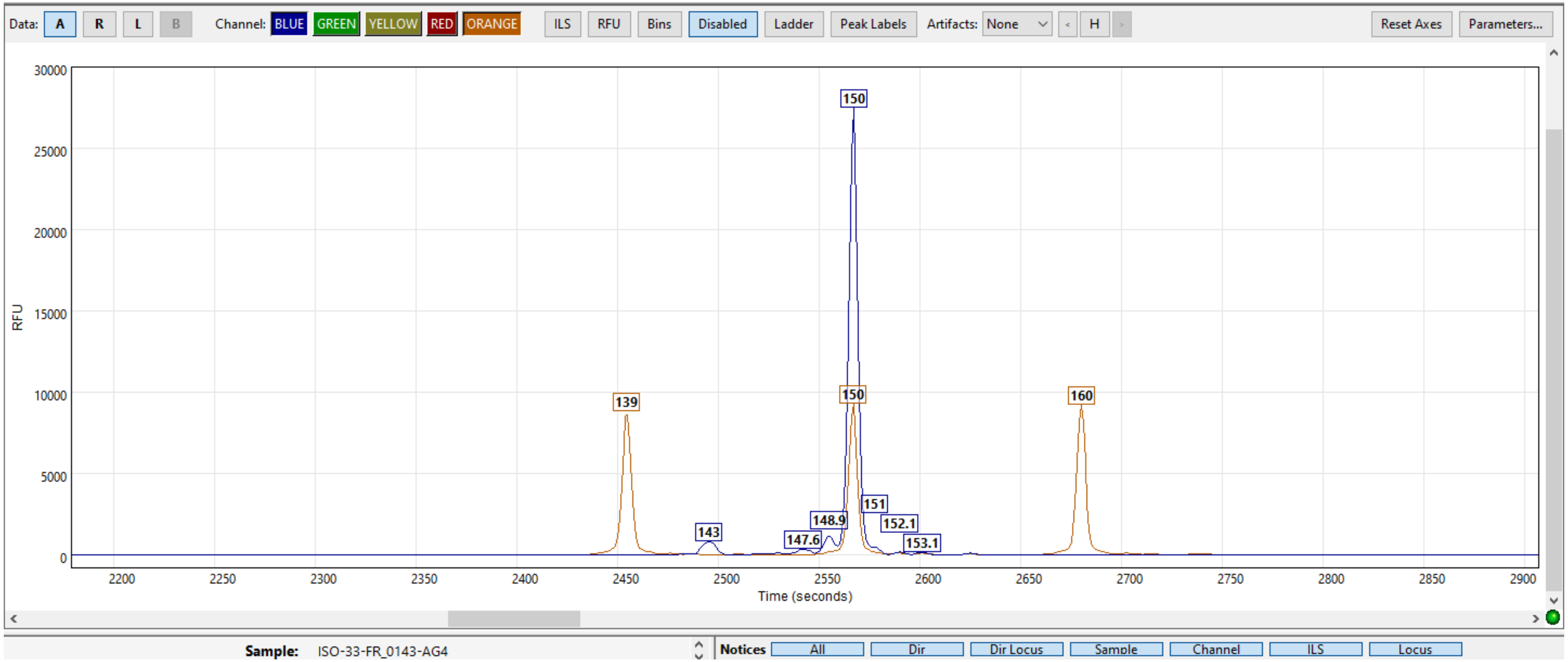


| | |
|---|---|
| **Observer 1** | **150** |
| **Observer 2** | **150** |
| **Observer 3** | **150** |

## AG7

### 1- Colony. Locus ISO AG7 sample 01 (0114)

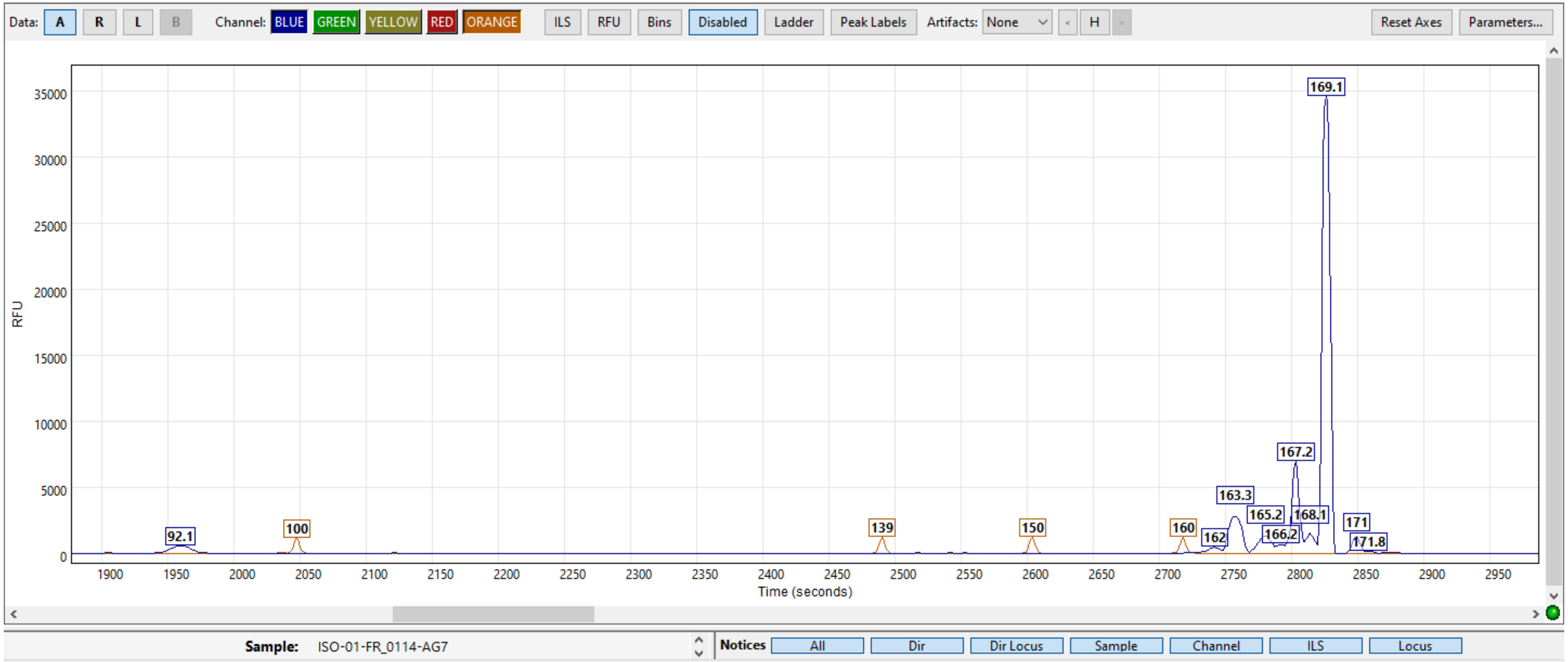


| | |
|---|---|
| **Observer 1** | **169** |
| **Observer 2** | **169.1** |
| **Observer 3** | **169.1** |

## 2- Colony. Locus ISO AG7 sample 02 (0115)

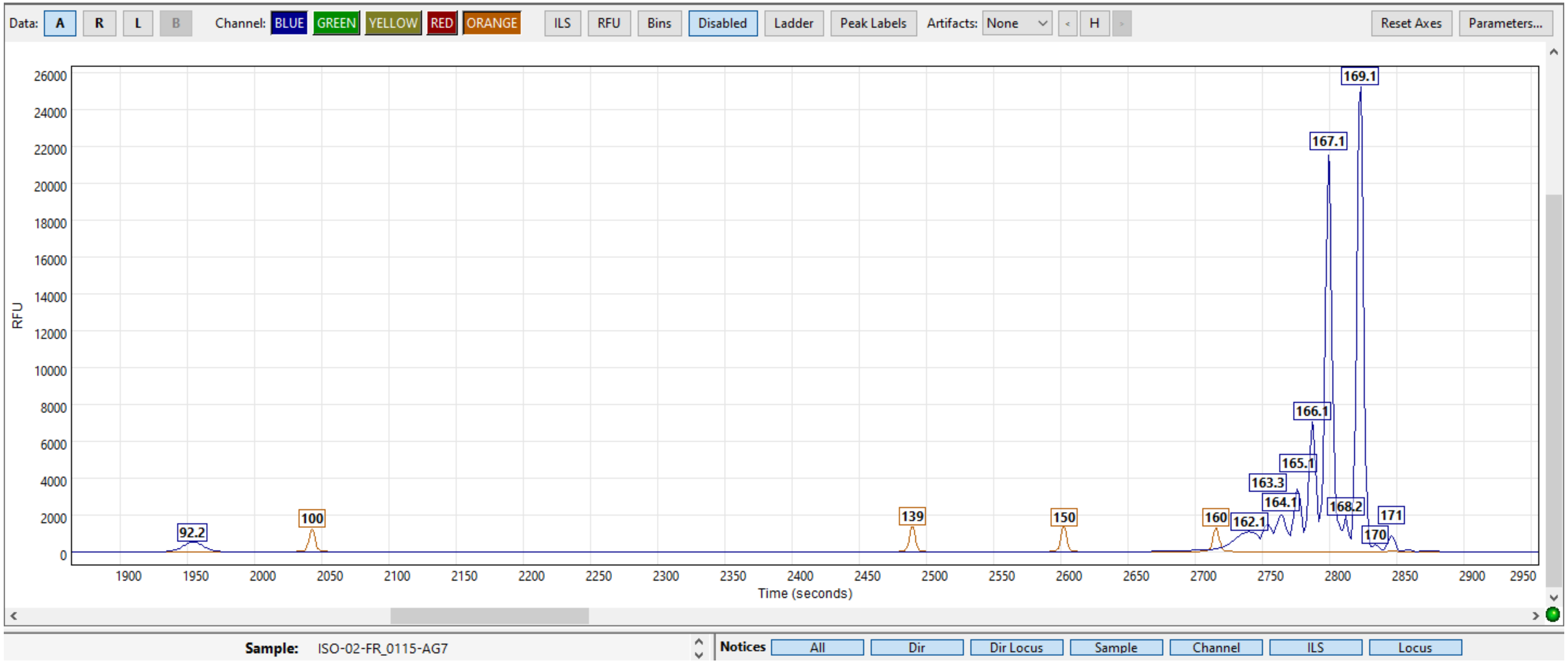


| | |
|---|---|
| **Observer 1** | **167; 169** |
| **Observer 2** | **167.1;169.1** |
| **Observer 3** | **167.1;169.1** |

### 3- Colony. Locus ISO AG7 sample 03 (0116)

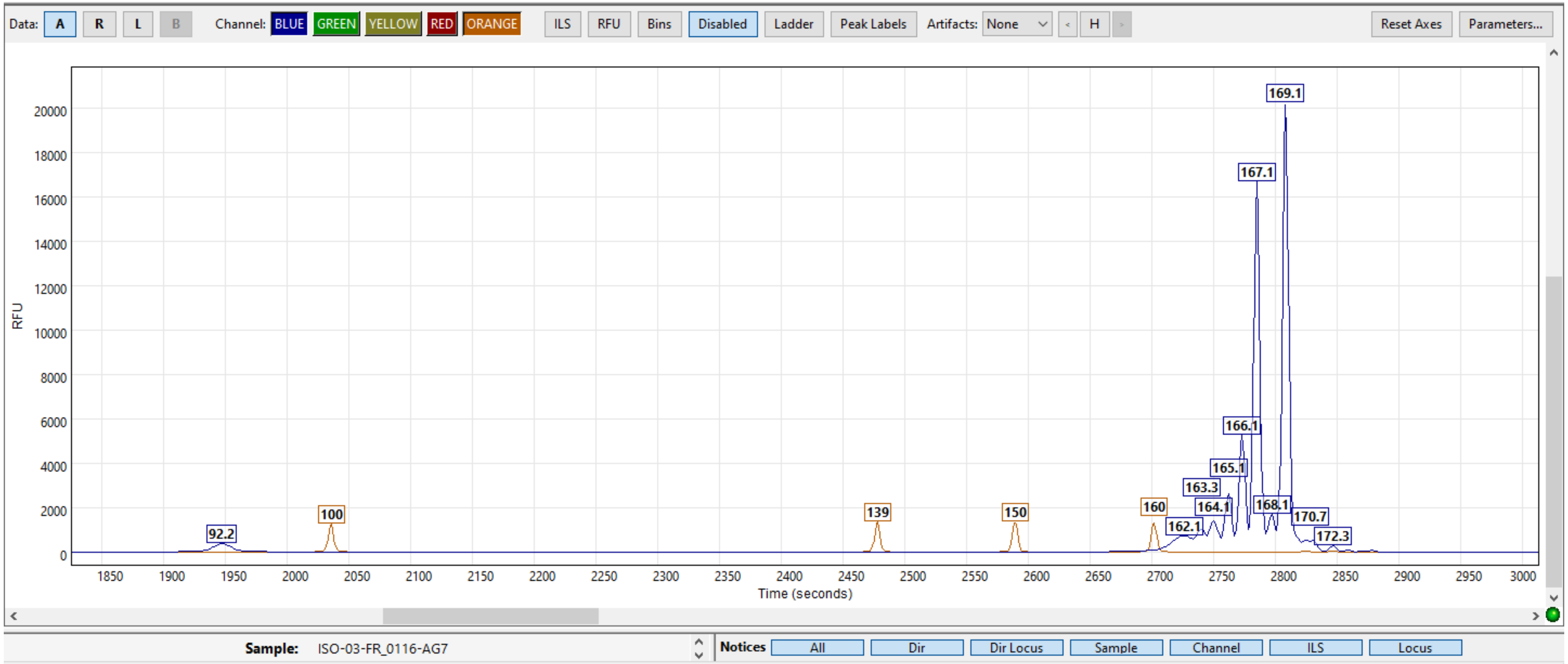


| | |
|---|---|
| **Observer 1** | **169 (167)** |
| **Observer 2** | **167.1;169.1** |
| **Observer 3** | **167.1;169.1** |

## 4- Colony. Locus ISO AG7 sample 04 (0117)

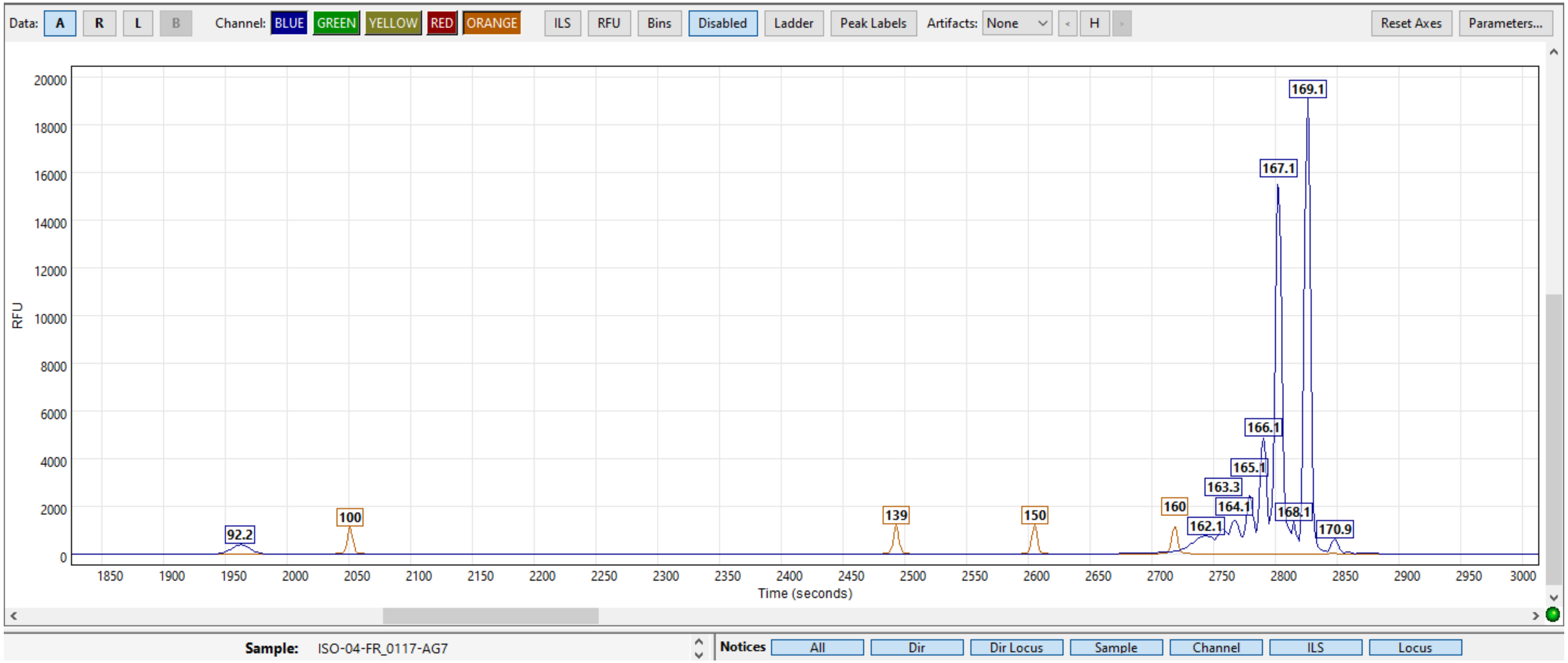


| | |
|---|---|
| **Observer 1** | **169 (167)** |
| **Observer 2** | **167.1;169.1** |
| **Observer 3** | **167.1;169.1** |

**5- Colony. Locus ISO AG7 sample 05 (0118)**

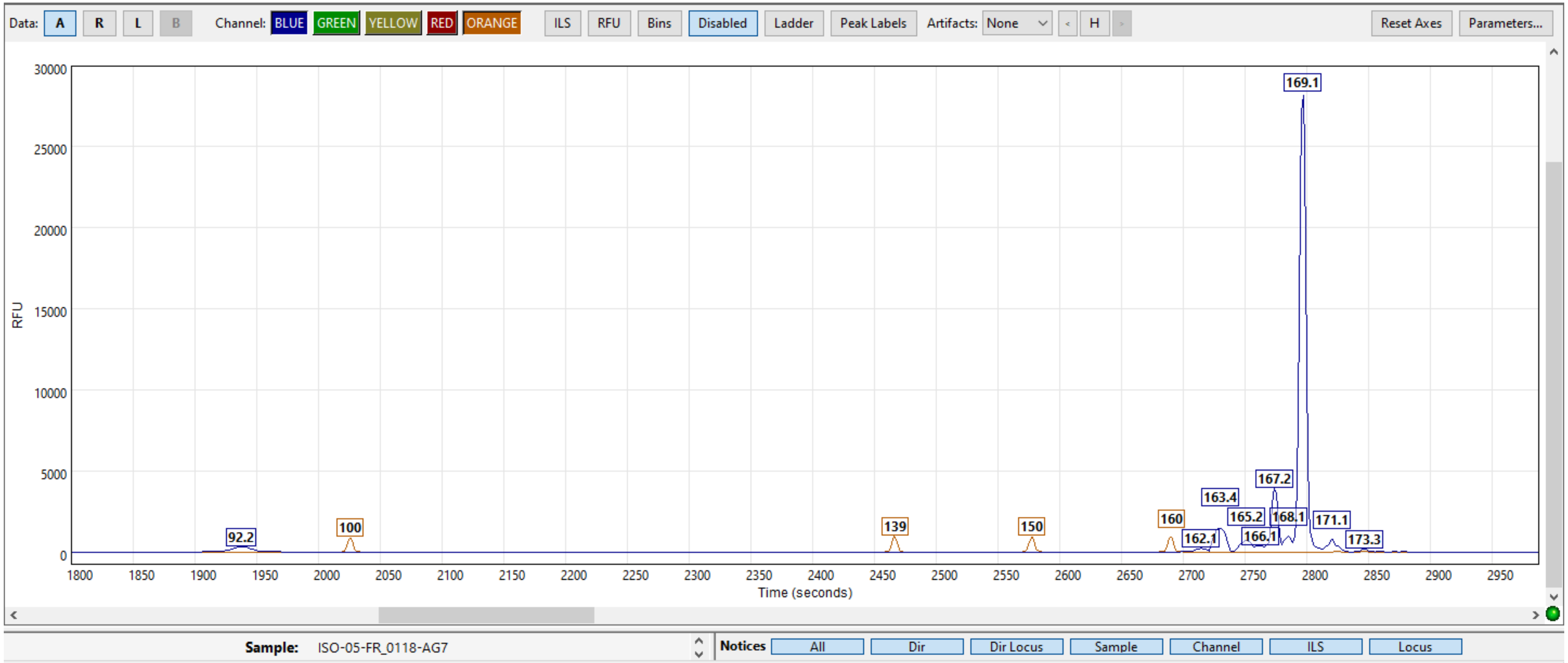


| | |
|---|---|
| **Observer 1** | **169 (167)** |
| **Observer 2** | **169.1** |
| **Observer 3** | **169.1** |

## 6- Colony. Locus ISO AG7 sample 06 (0119)

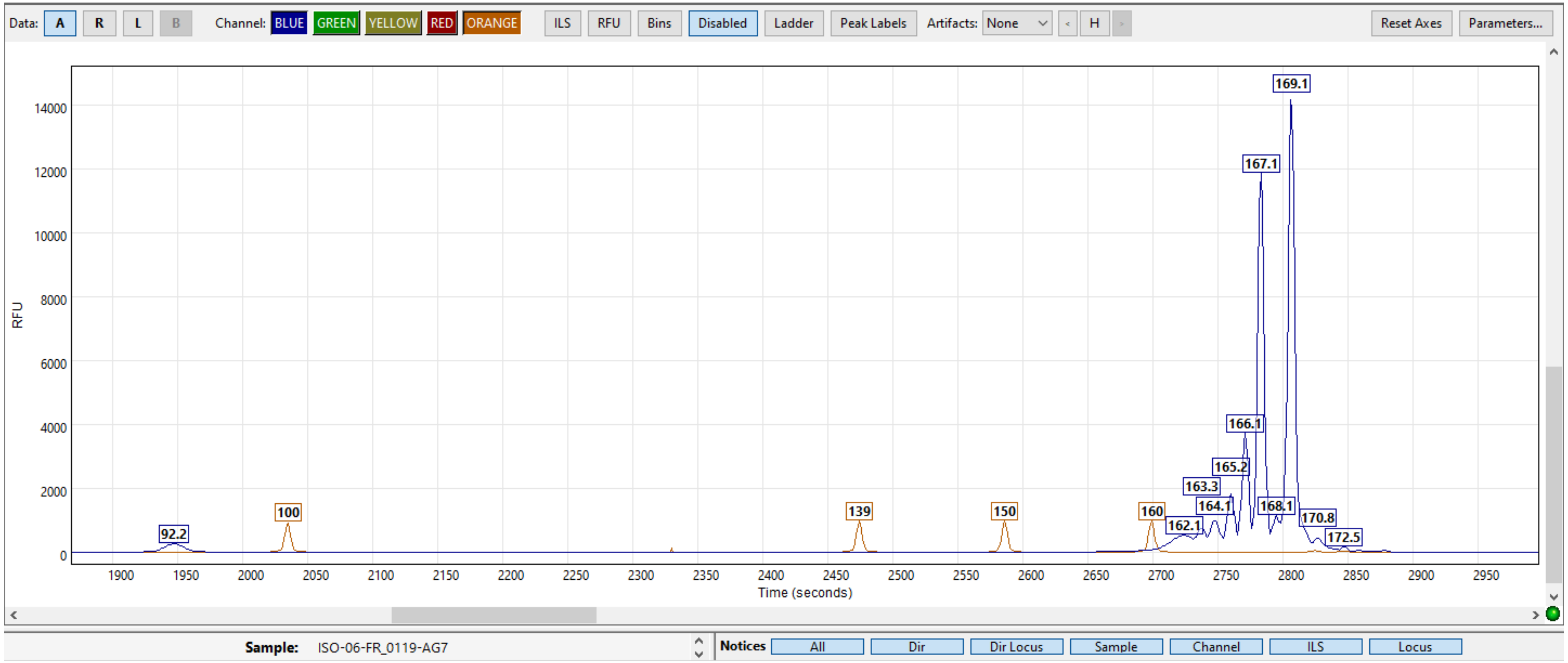


| | |
|---|---|
| **Observer 1** | **169 (167)** |
| **Observer 2** | **167.1;169.1** |
| **Observer 3** | **167.1;169.1** |

## 7- Colony. Locus ISO AG7 sample 07 (0120)

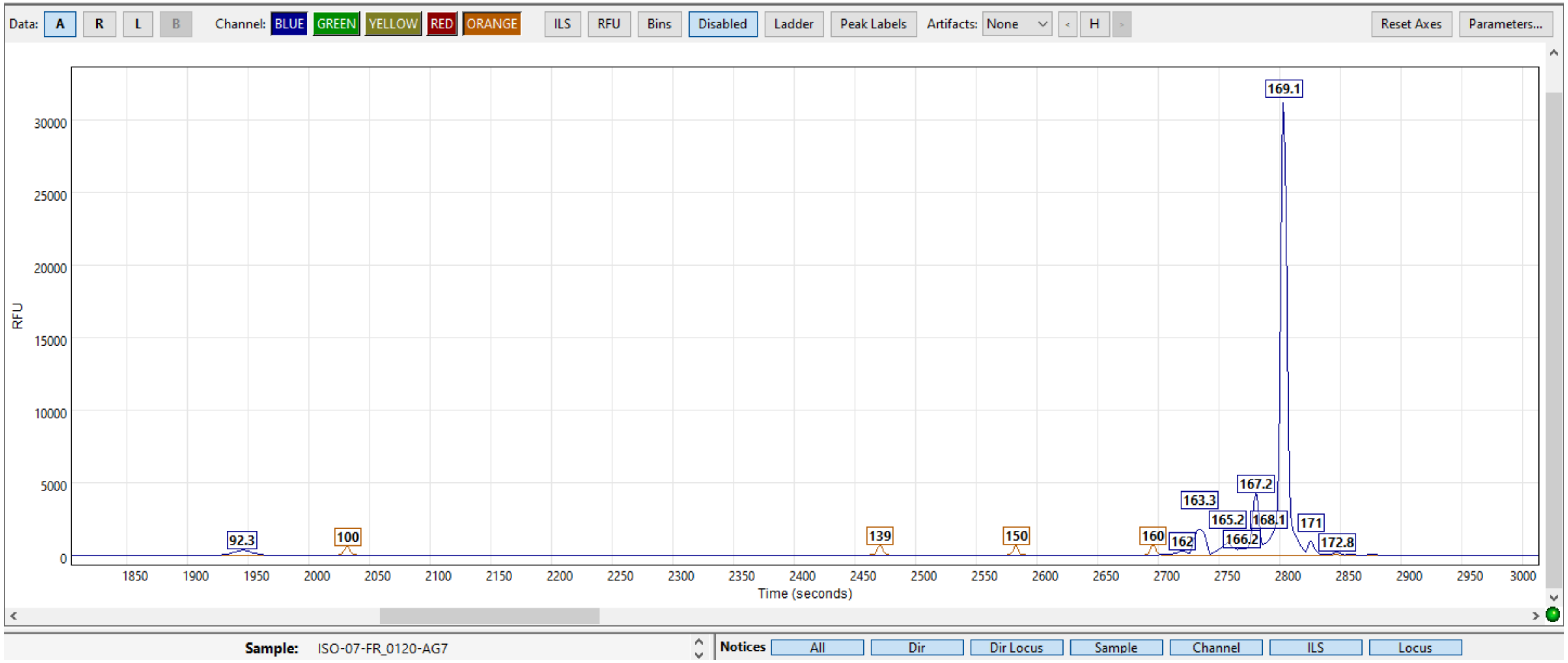


| | |
|---|---|
| **Observer 1** | **169 (167)** |
| **Observer 2** | **169.1** |
| **Observer 3** | **169.1** |

## 8- Colony. Locus ISO AG7 sample 08 (0121)

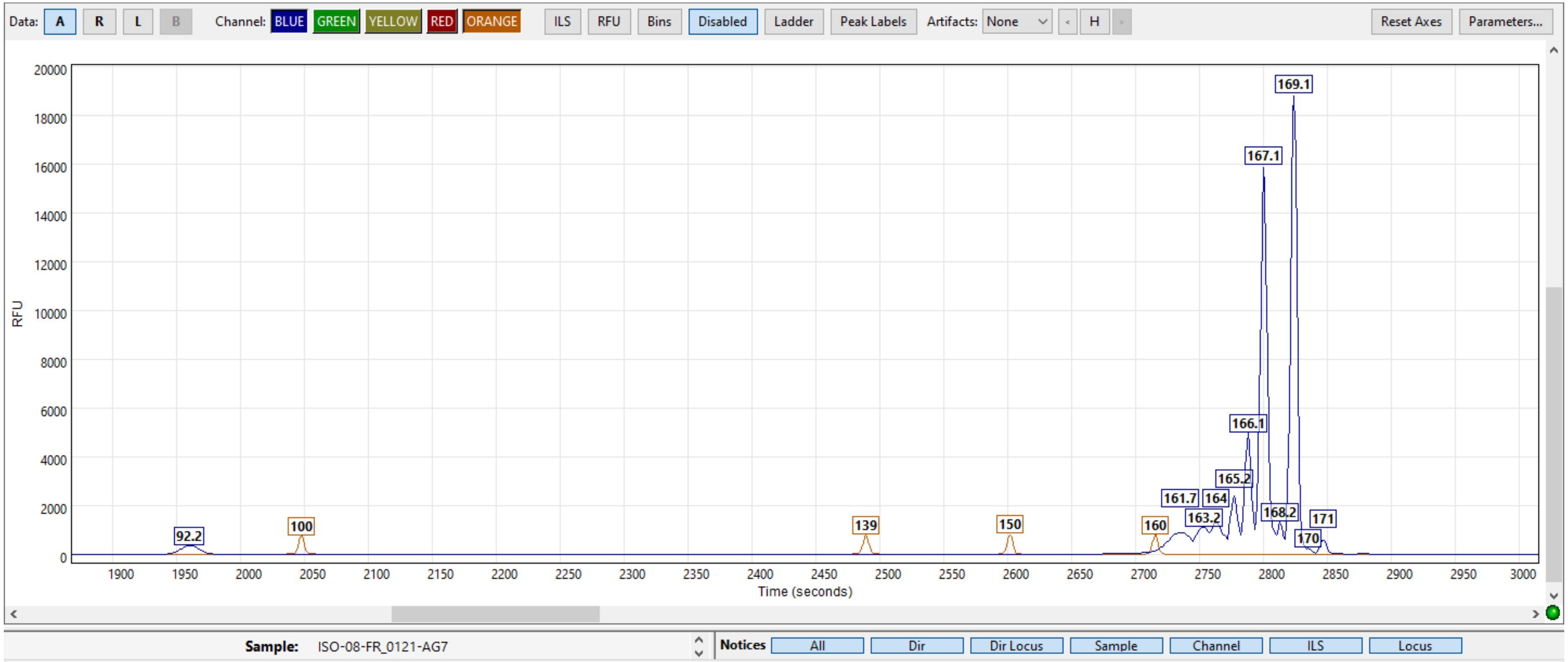


| | |
|---|---|
| **Observer 1** | **169 (167)** |
| **Observer 2** | **167.1;169.1** |
| **Observer 3** | **167.1;169.1** |

## 9- Colony. Locus ISO AG7 sample 09 (0122)

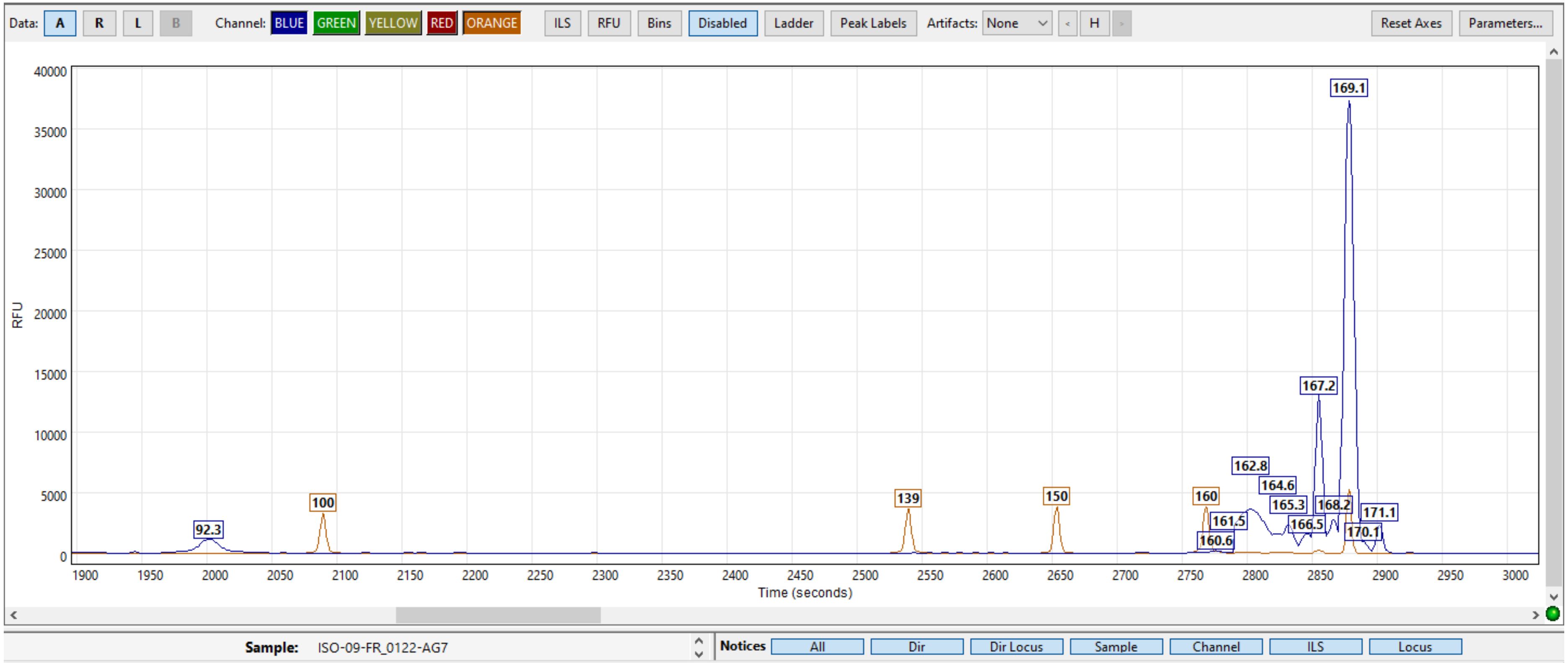


| | |
|---|---|
| **Observer 1** | **169 (167)** |
| **Observer 2** | **169.1** |
| **Observer 3** | **167;169** |

**10- Colony. Locus ISO AG7 sample 10 (0123)**

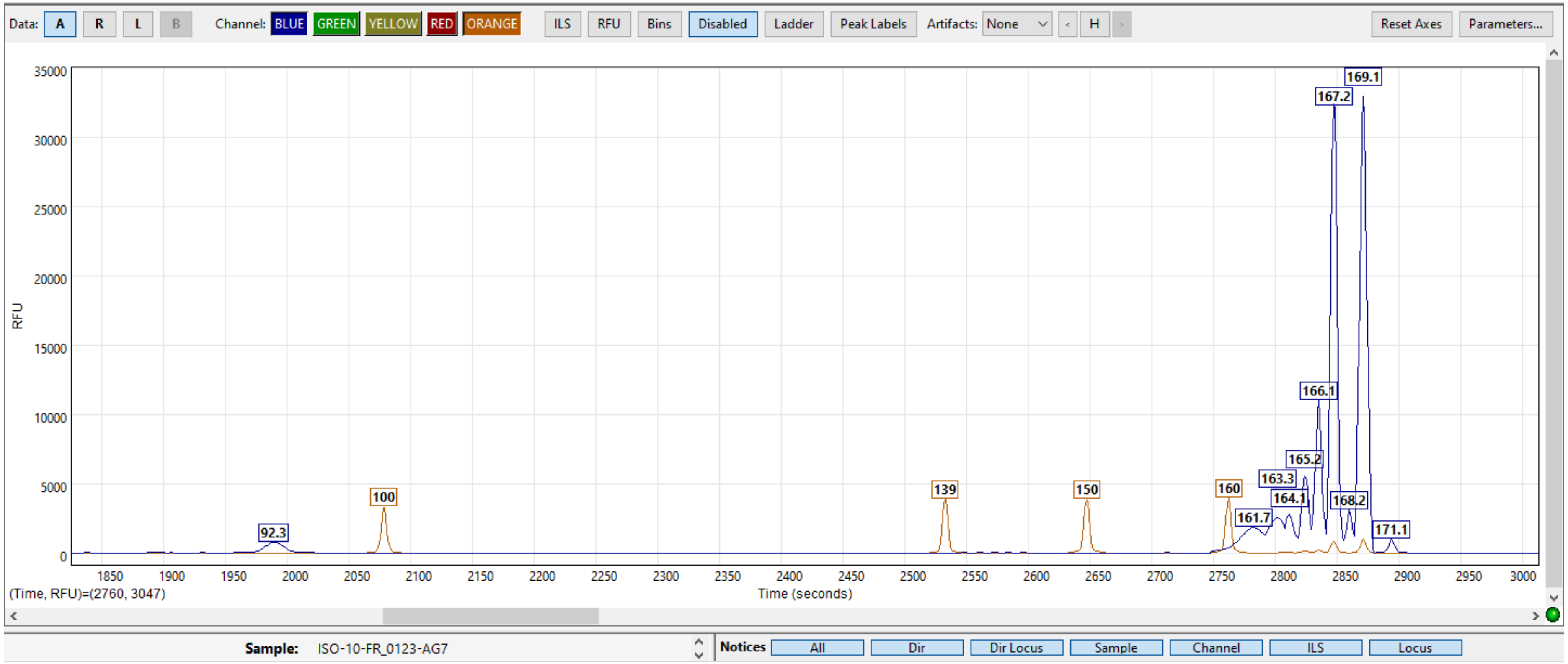


| | |
|---|---|
| **Observer 1** | **169 (167)** |
| **Observer 2** | **167.2;169.1** |
| **Observer 3** | **167.2;169.1** |

### 11- Colony. Locus ISO AG7 sample 11 (0124)

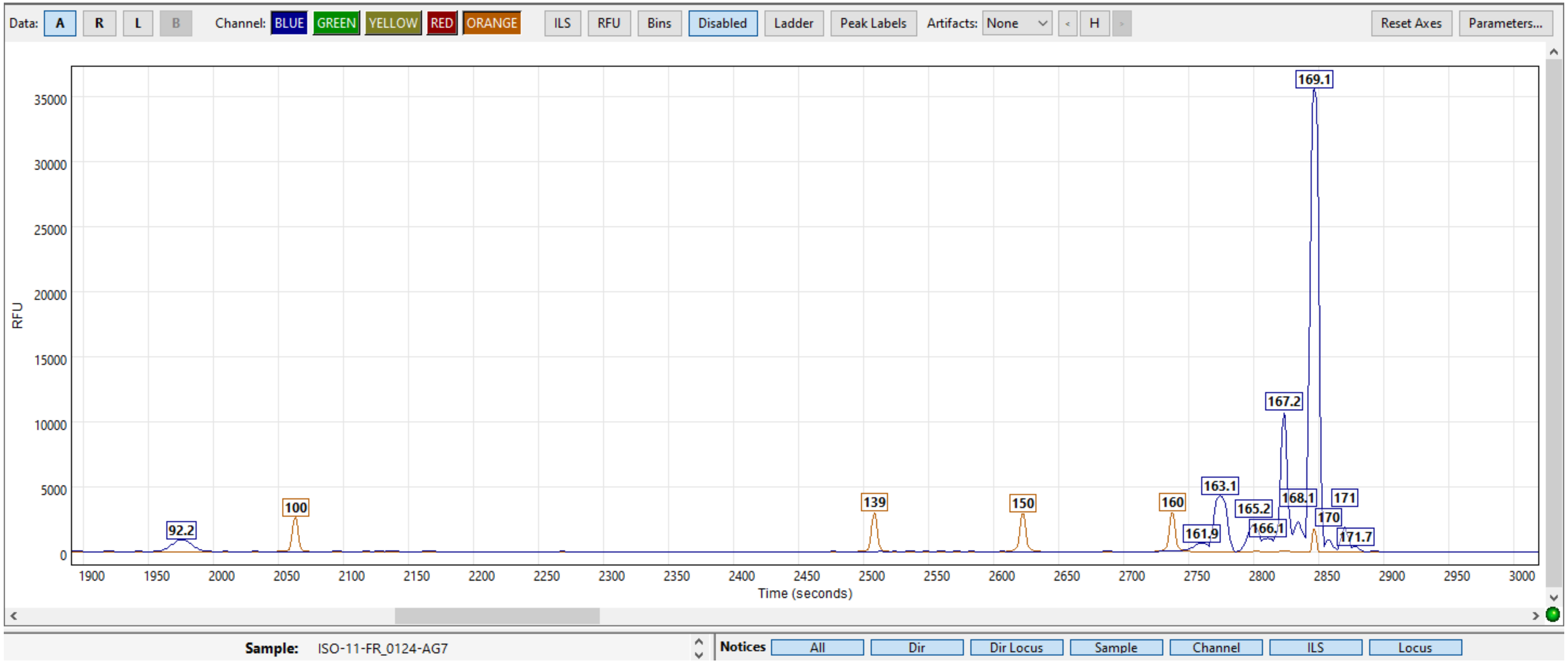


| | |
|---|---|
| **Observer 1** | **169 (167)** |
| **Observer 2** | **169.1** |
| **Observer 3** | **169.1** |

## 12- Colony. Locus ISO AG7 sample 12 (0125)

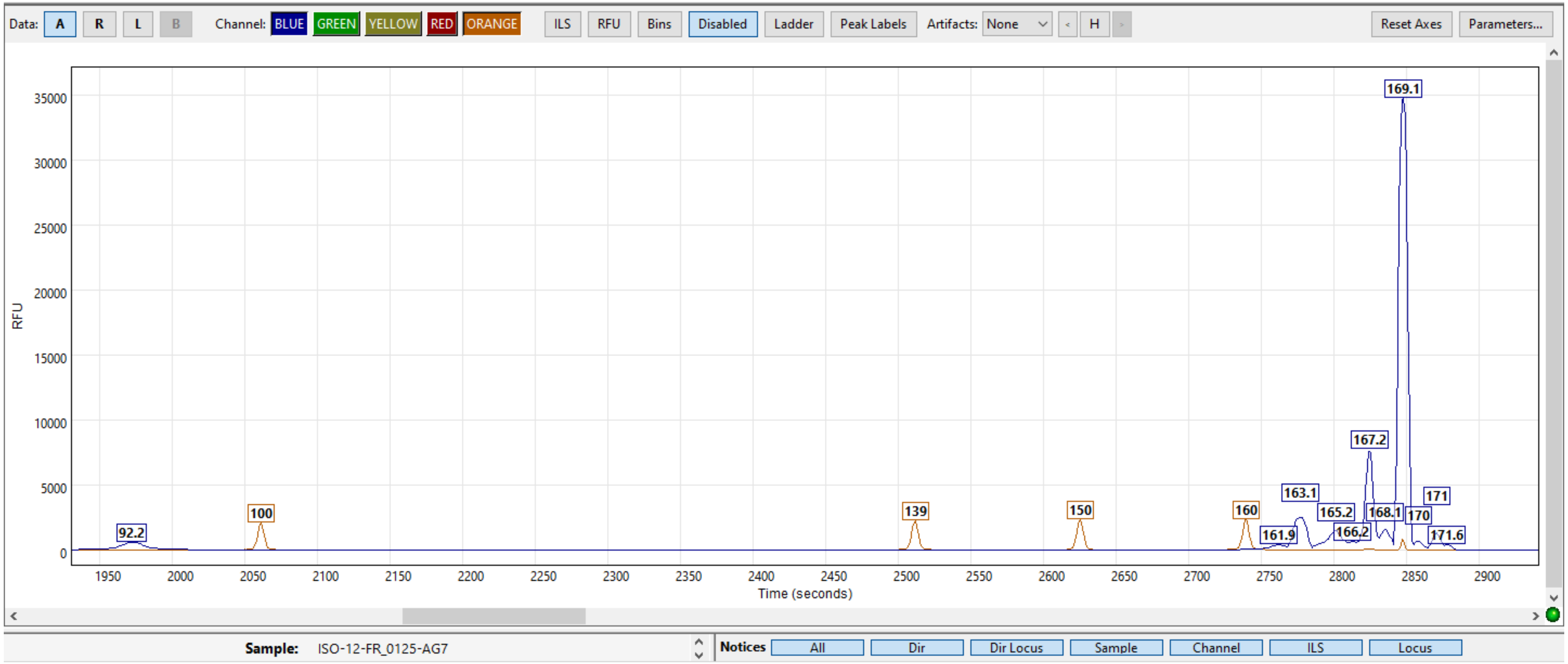


| | |
|---|---|
| **Observer 1** | **169** |
| **Observer 2** | **169.1** |
| **Observer 3** | **169.1** |

**13- Colony. Locus ISO AG7 sample 13 (0126)**

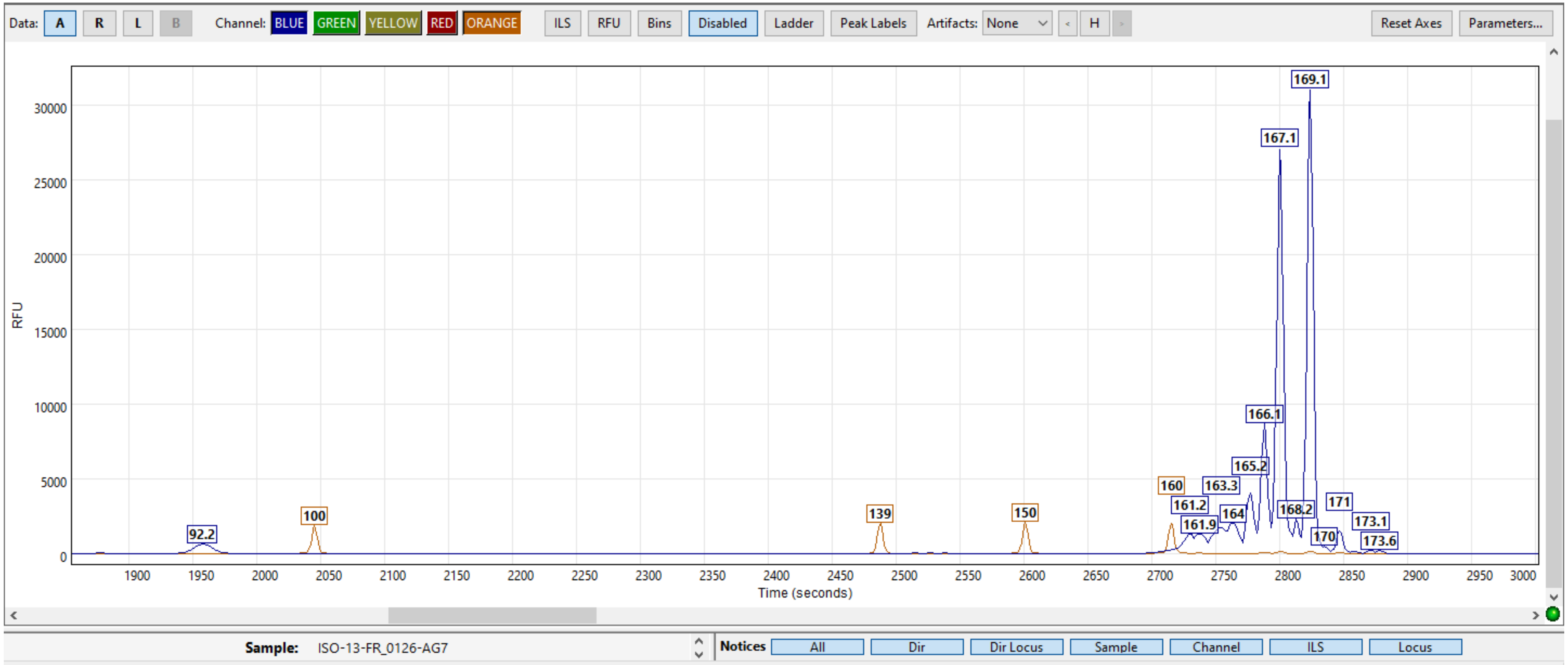


| | |
|---|---|
| **Observer 1** | **169 (167)** |
| **Observer 2** | **167.1;169.1** |
| **Observer 3** | **167.1;169.1** |

**14- Colony. Locus ISO AG7 sample 14 (0127)**

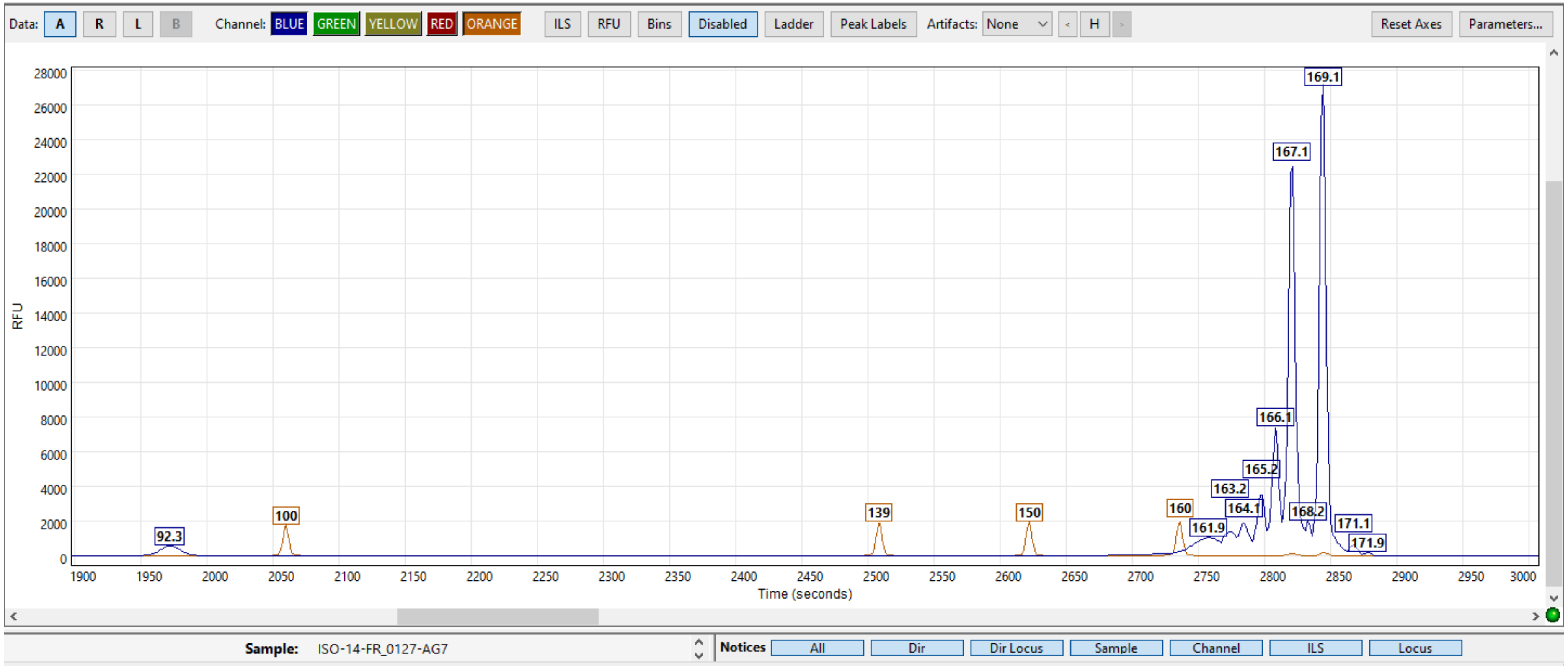


| | |
|---|---|
| **Observer 1** | **169 (167)** |
| **Observer 2** | **167.1;169.1** |
| **Observer 3** | **167.1;169.1** |

## 15- Colony. Locus ISO AG7 sample 15 (0128)

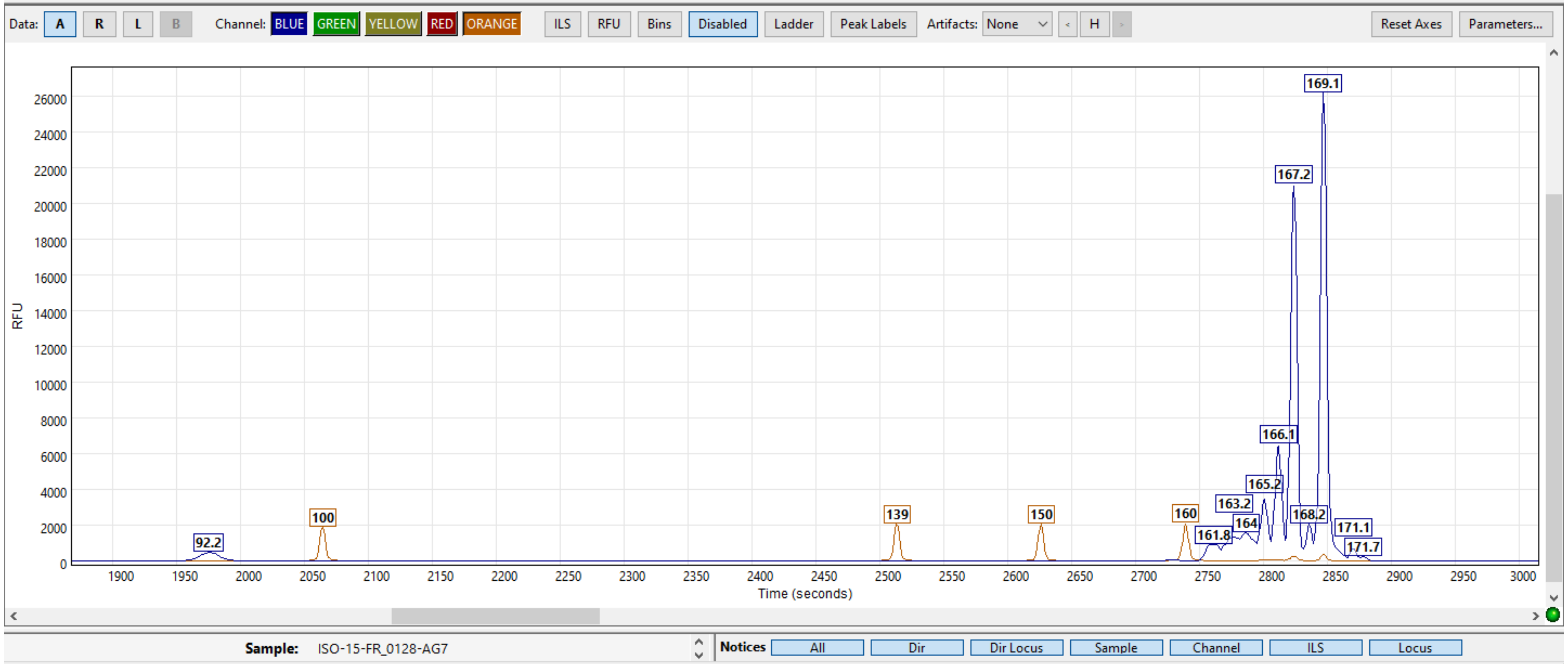


| | |
|---|---|
| **Observer 1** | **169 (167)** |
| **Observer 2** | **167.2;169.1** |
| **Observer 3** | **167.2;169.1** |

**16- Colony. Locus ISO AG7 sample 16 (0129)**

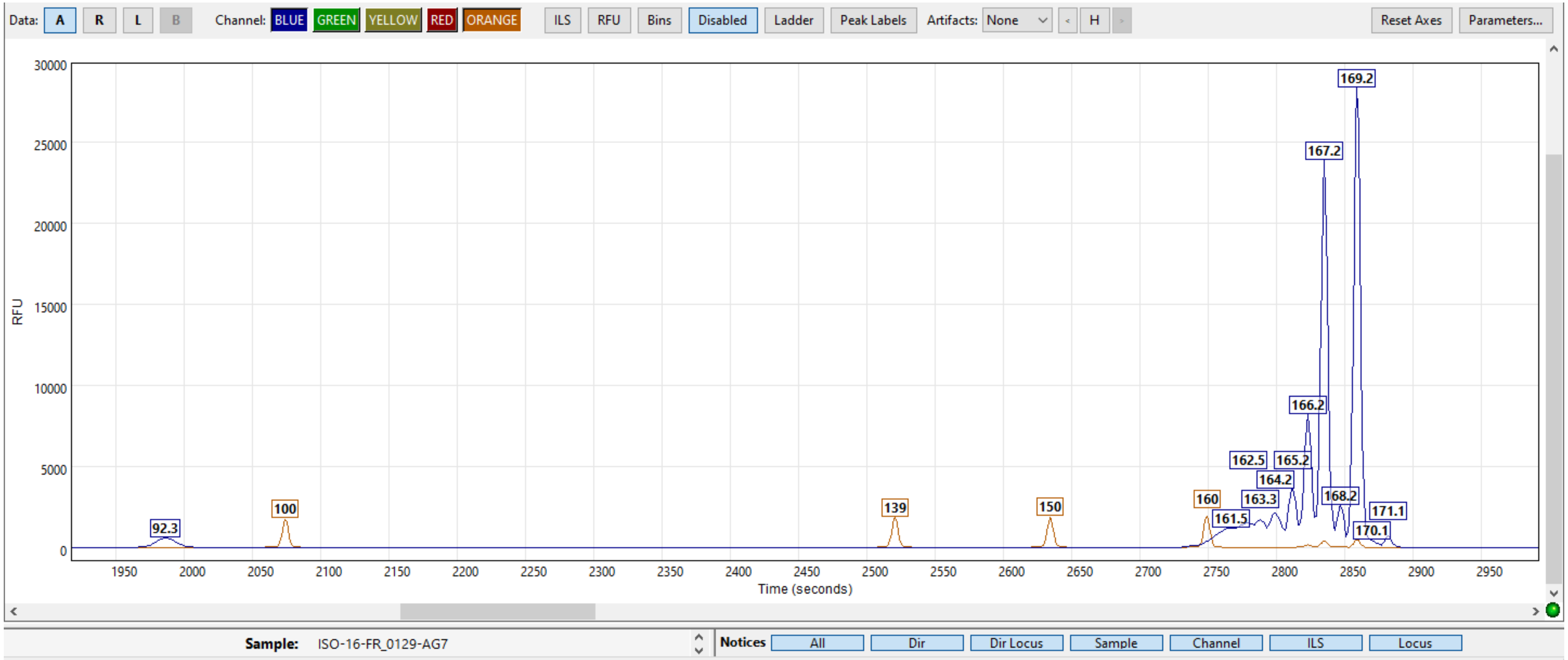


| | |
|---|---|
| **Observer 1** | **169 (167)** |
| **Observer 2** | **167.2;169.2** |
| **Observer 3** | **167.2;169.2** |

**17- Colony. Locus ISO AG7 sample 17 (0130)**

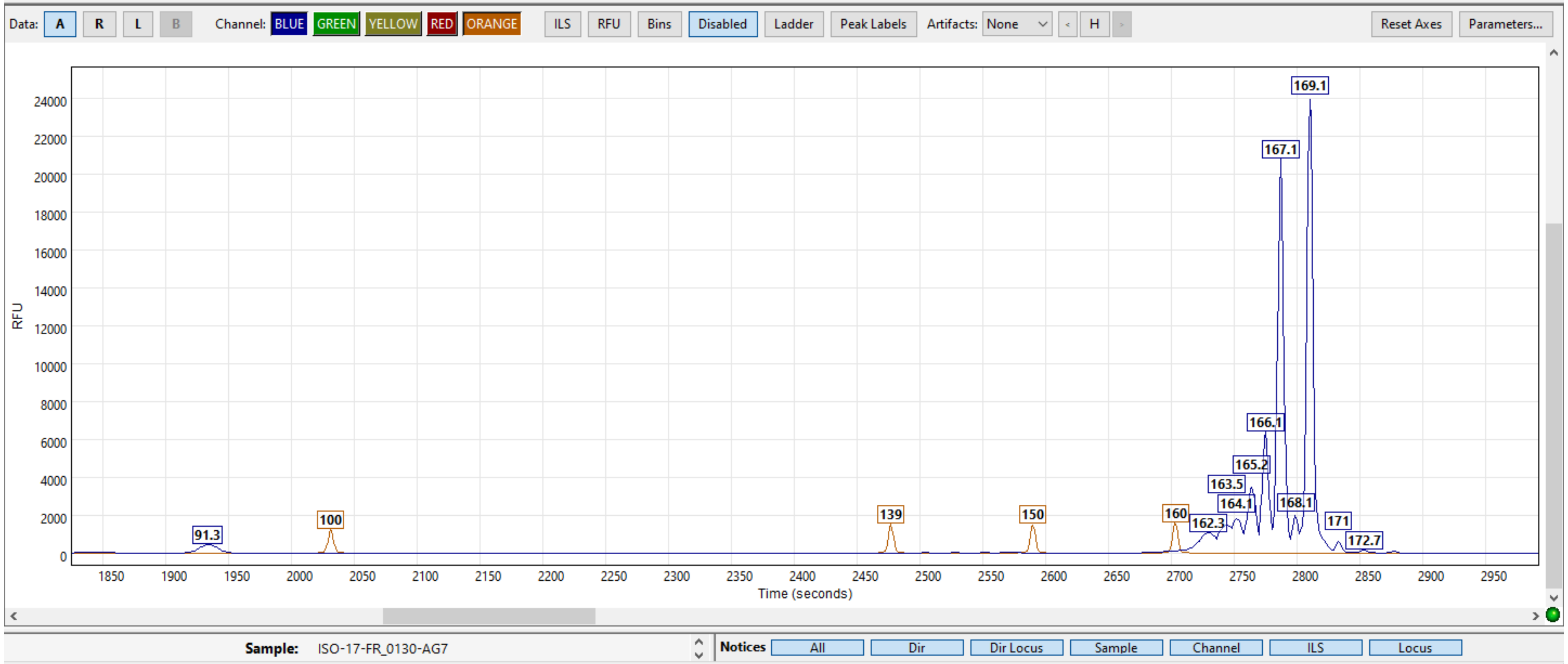


| | |
|---|---|
| **Observer 1** | **169 (167)** |
| **Observer 2** | **167.1;169.1** |
| **Observer 3** | **167.1;169.1** |

### 18- Colony. Locus ISO AG7 sample 19 (0131)

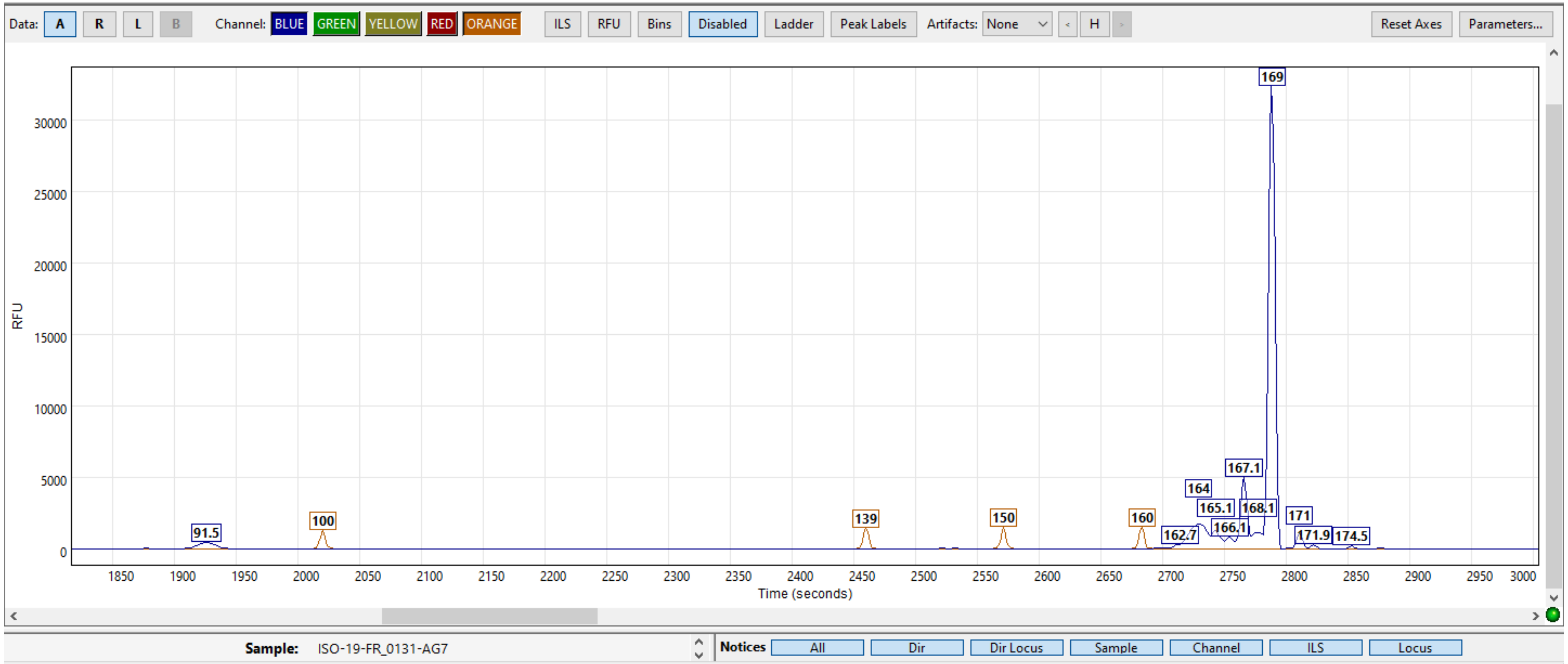


| | |
|---|---|
| **Observer 1** | **169 (167)** |
| **Observer 2** | **169** |
| **Observer 3** | **169** |

## 19- Colony. Locus ISO AG7 sample 20 (0132)

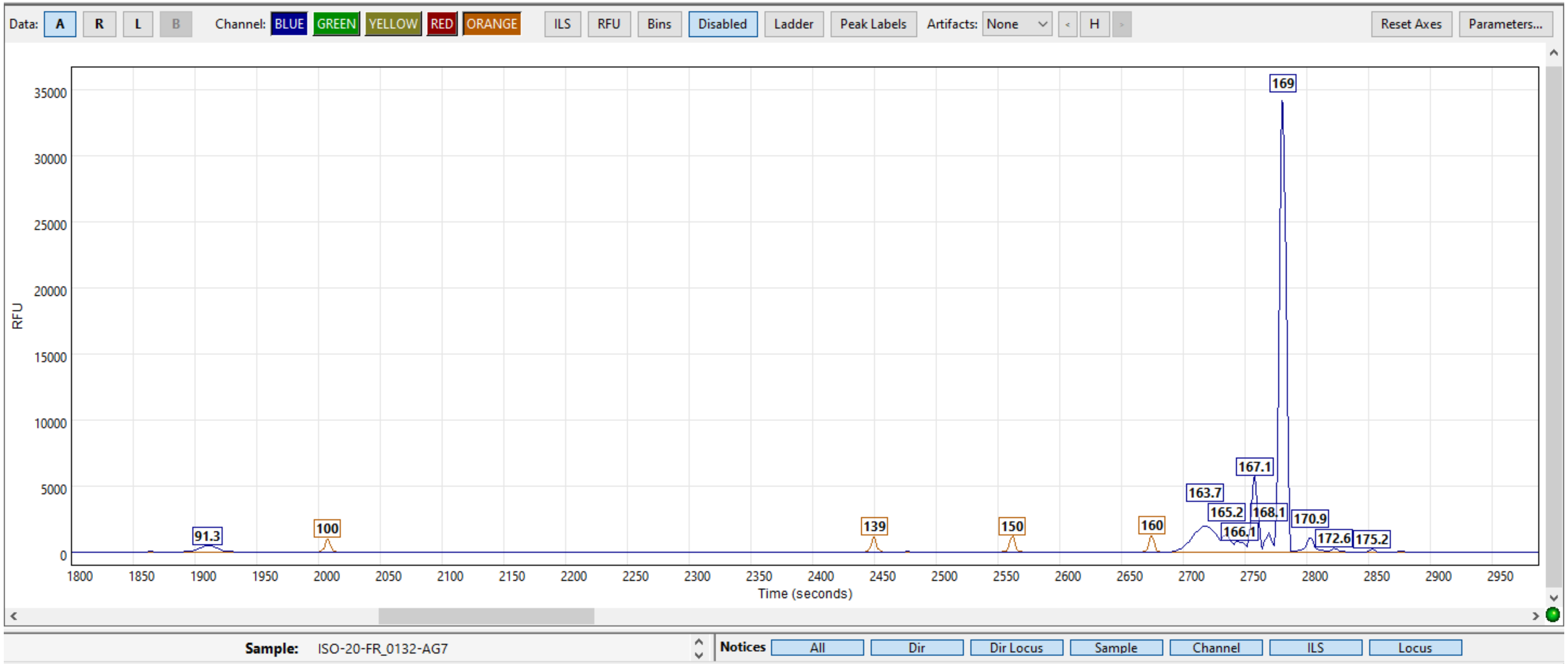


| | |
|---|---|
| **Observer 1** | **169 (167)** |
| **Observer 2** | **169** |
| **Observer 3** | **169** |

**20- Colony. Locus ISO AG7 sample 21 (0133)**

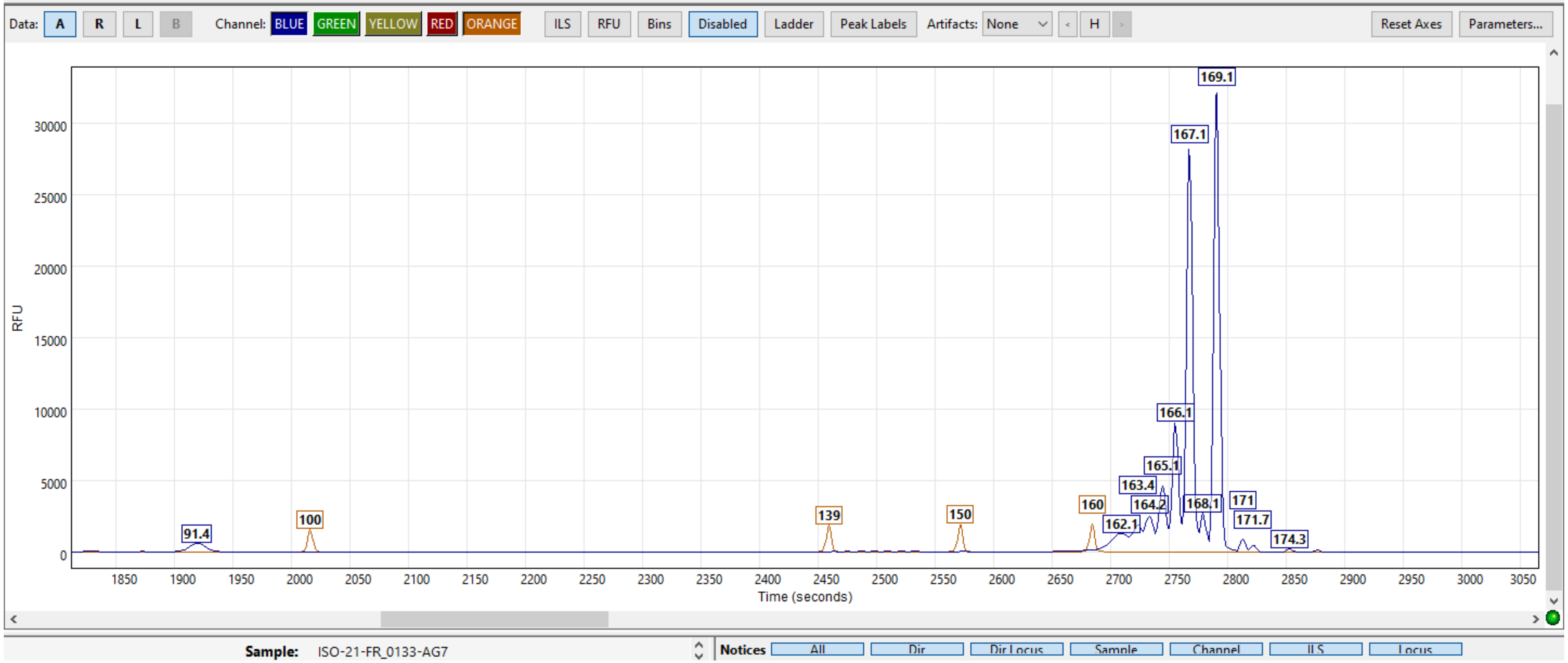


| | |
|---|---|
| **Observer 1** | **169 (167)** |
| **Observer 2** | **167.1;169.1** |
| **Observer 3** | **167.1;169.1** |

## 21- Colony. Locus ISO AG7 sample 23 (0134)

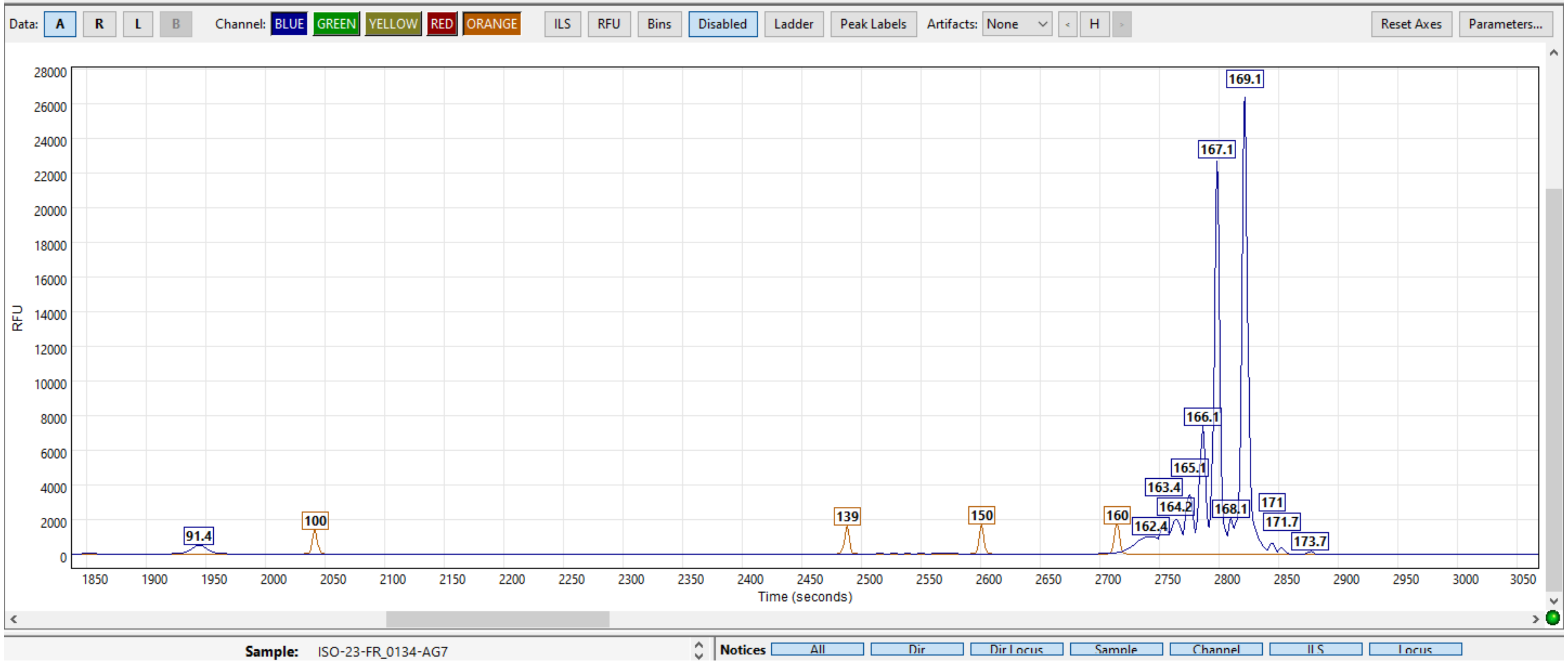


| | |
|---|---|
| **Observer 1** | **169 (167)** |
| **Observer 2** | **167.1;169.1** |
| **Observer 3** | **167.1;169.1** |

**22- Colony. Locus ISO AG7 sample 24 (0135)**

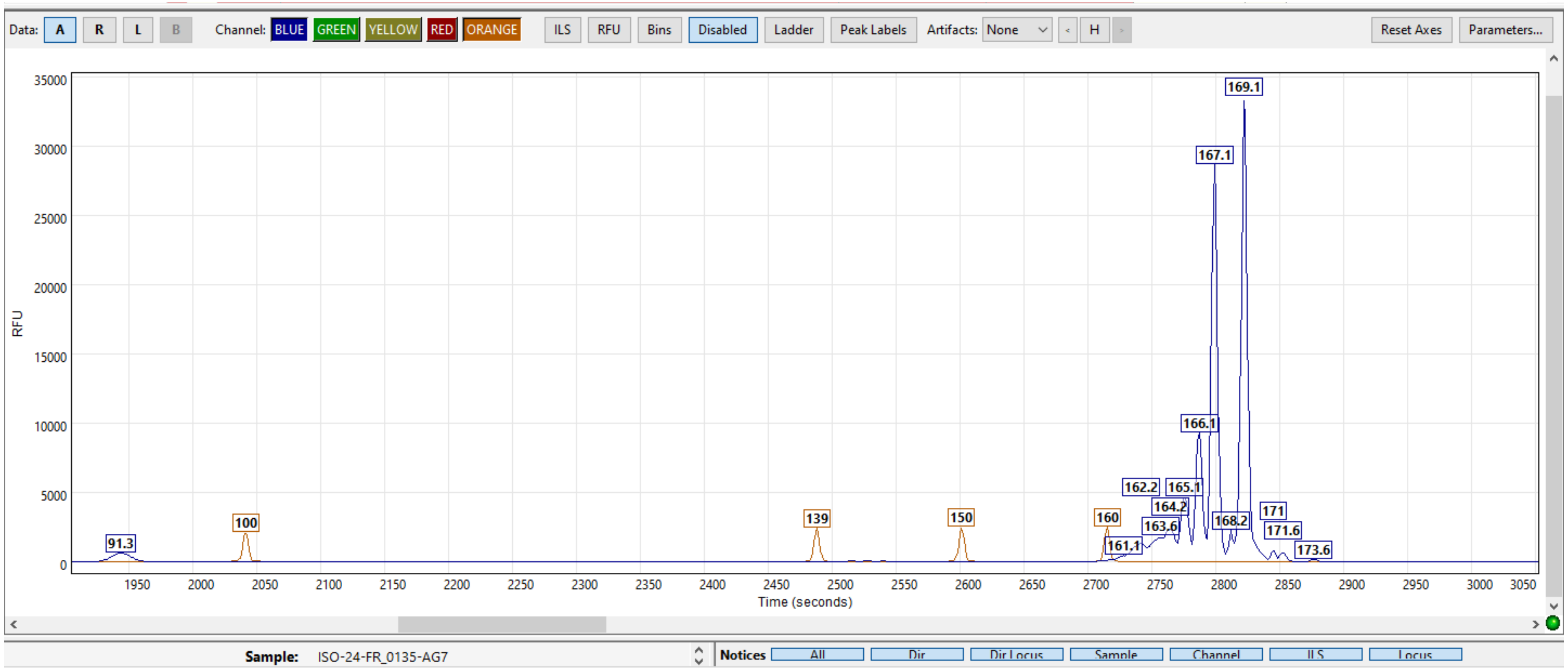


| | |
|---|---|
| **Observer 1** | **169 (167)** |
| **Observer 2** | **167.1;169.1** |
| **Observer 3** | **167.1;169.1** |

**23- Colony. Locus ISO AG7 sample 25 (0136)**

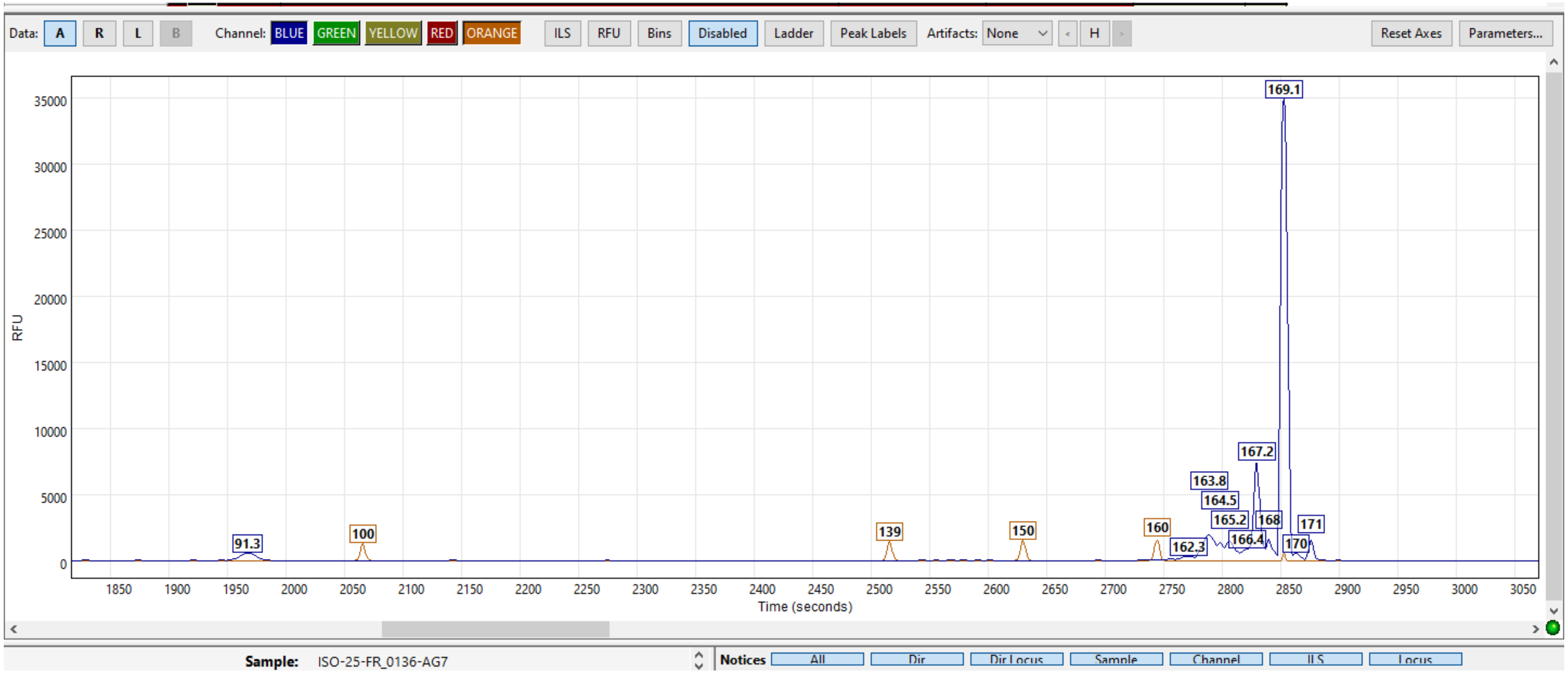


| | |
|---|---|
| **Observer 1** | **169 (167)** |
| **Observer 2** | **169.1** |
| **Observer 3** | **169.1** |

## 24- Colony. Locus ISO AG7 sample 26 (0137)

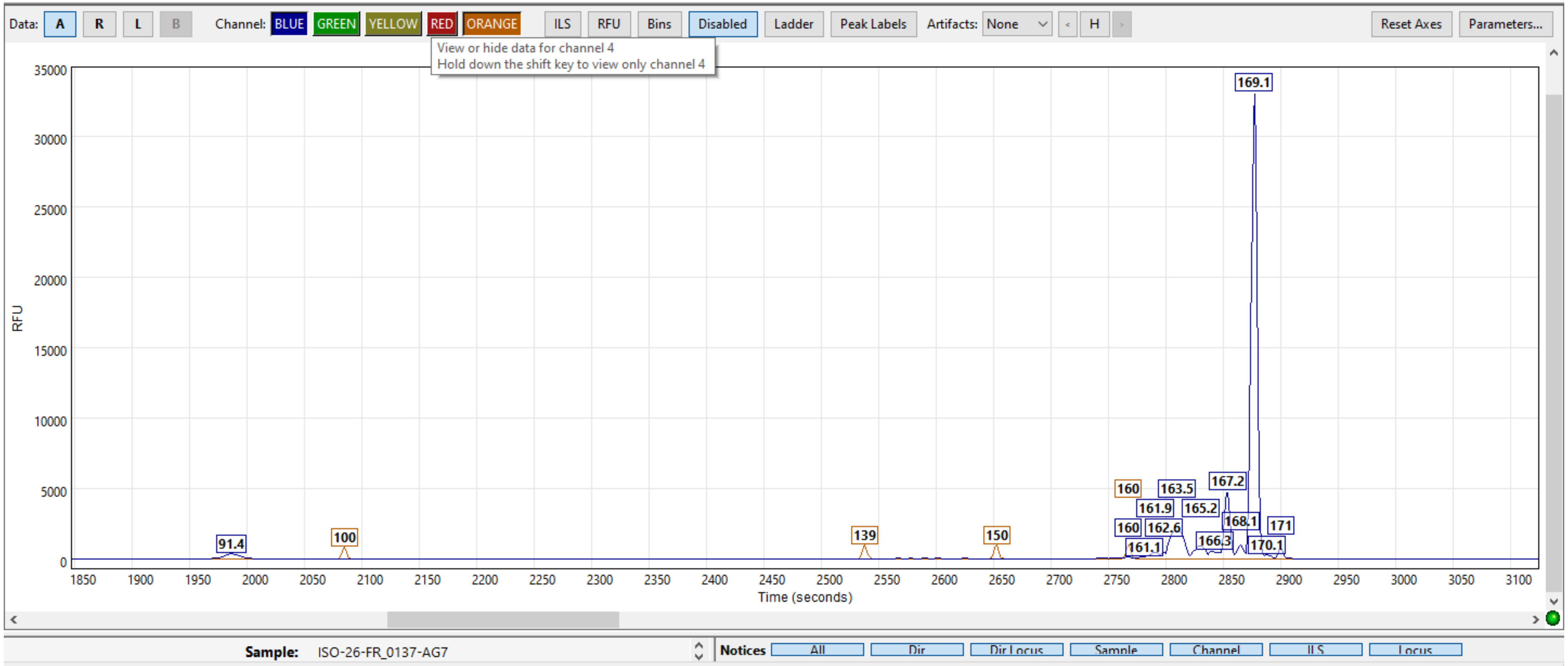


| | |
|---|---|
| **Observer 1** | **169 (167)** |
| **Observer 2** | **169.1** |
| **Observer 3** | **169.1** |

**25- Colony. Locus ISO AG7 sample 27 (0138)**

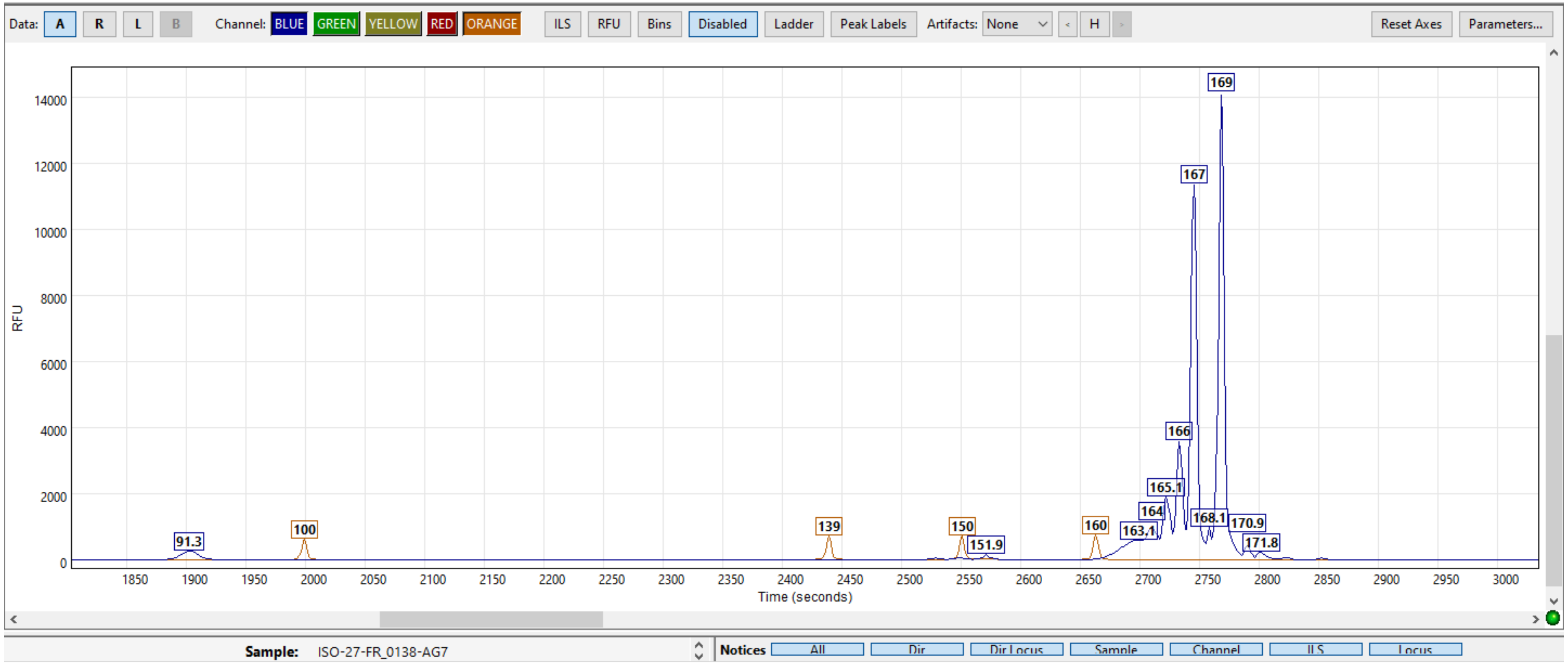


| | |
|---|---|
| **Observer 1** | **169 (167)** |
| **Observer 2** | **167;169** |
| **Observer 3** | **167;169** |

## 26- Colony. Locus ISO AG7 sample 29 (0139)

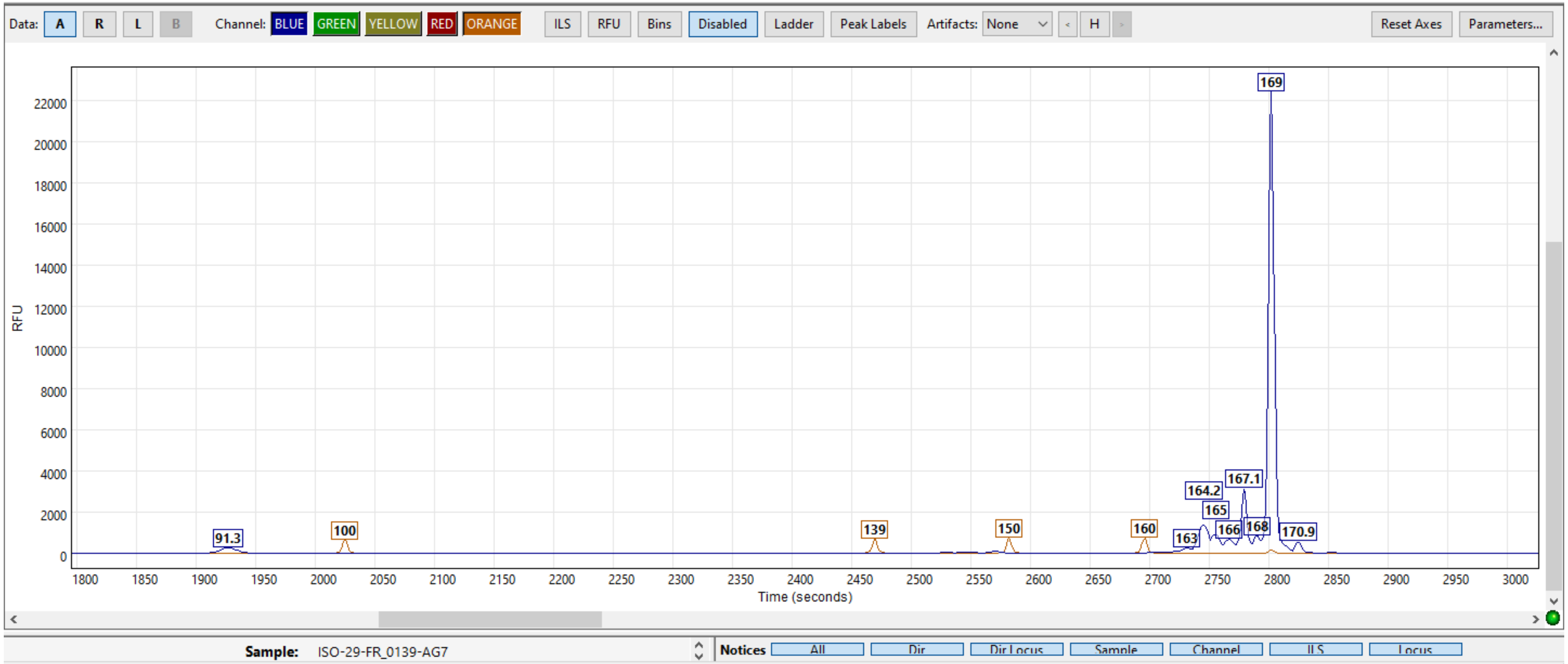


| | |
|---|---|
| **Observer 1** | **169 (167)** |
| **Observer 2** | **169** |
| **Observer 3** | **169** |

**27- Colony. Locus ISO AG7 sample 30 (0140)**

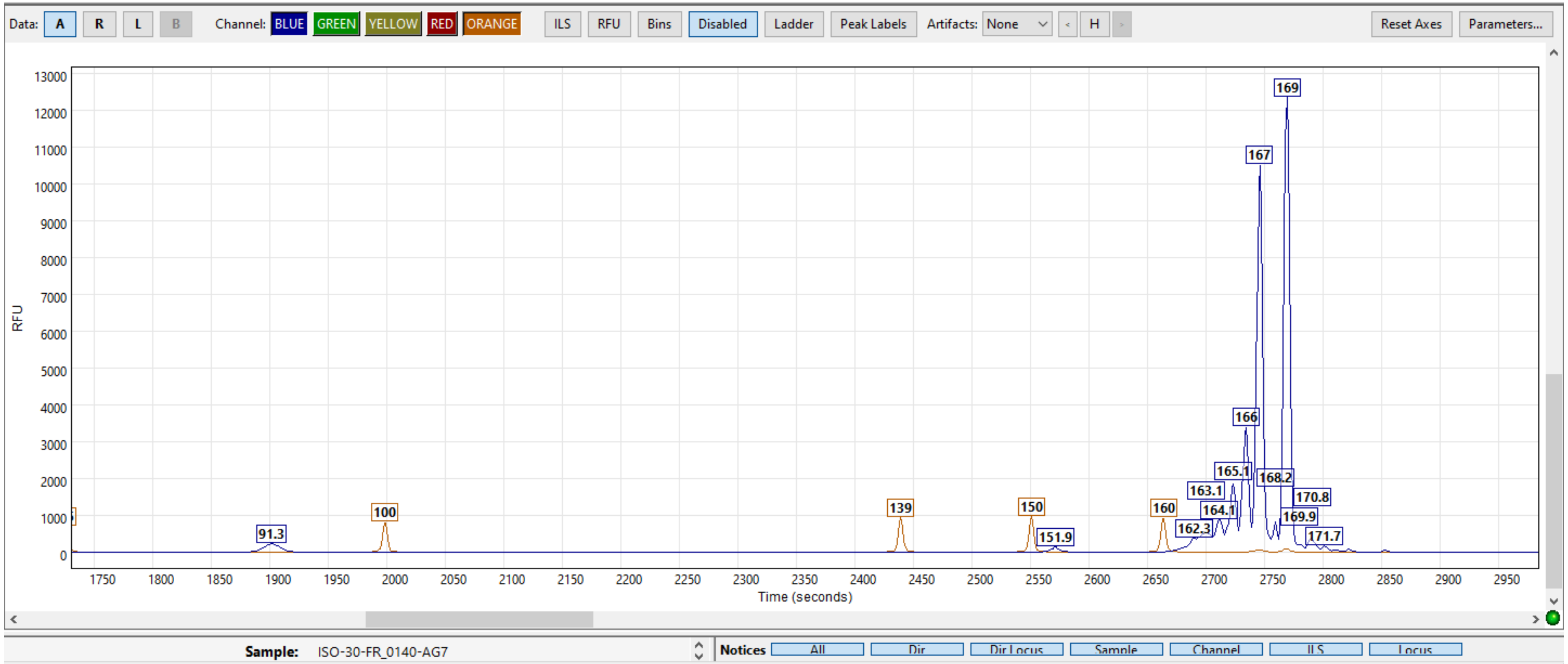


| | |
|---|---|
| **Observer 1** | **169 (167)** |
| **Observer 2** | **167;169** |
| **Observer 3** | **167;169** |

**28- Colony. Locus ISO AG7 sample 31 (0141)**

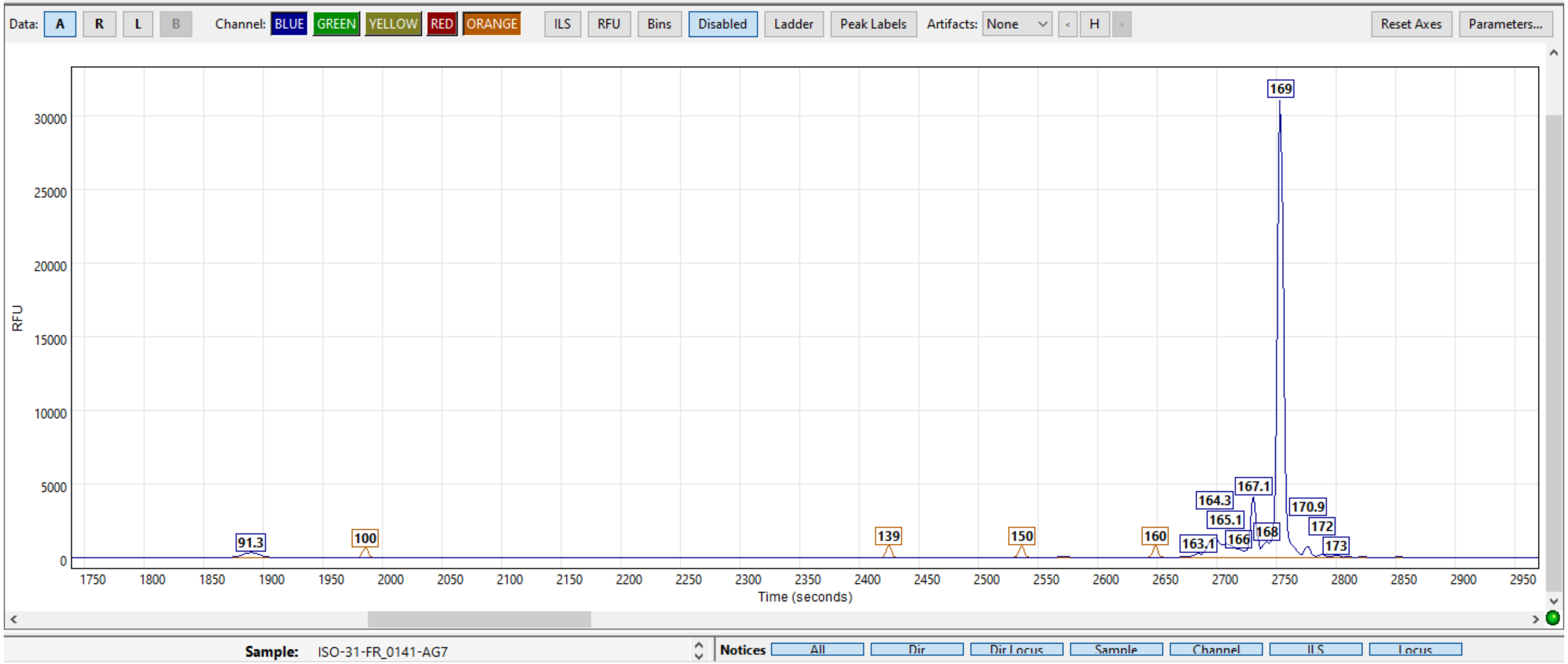


| | |
|---|---|
| **Observer 1** | **169 (167)** |
| **Observer 2** | **169** |
| **Observer 3** | **169** |

**29- Colony. Locus ISO AG7 sample 32 (0142)**

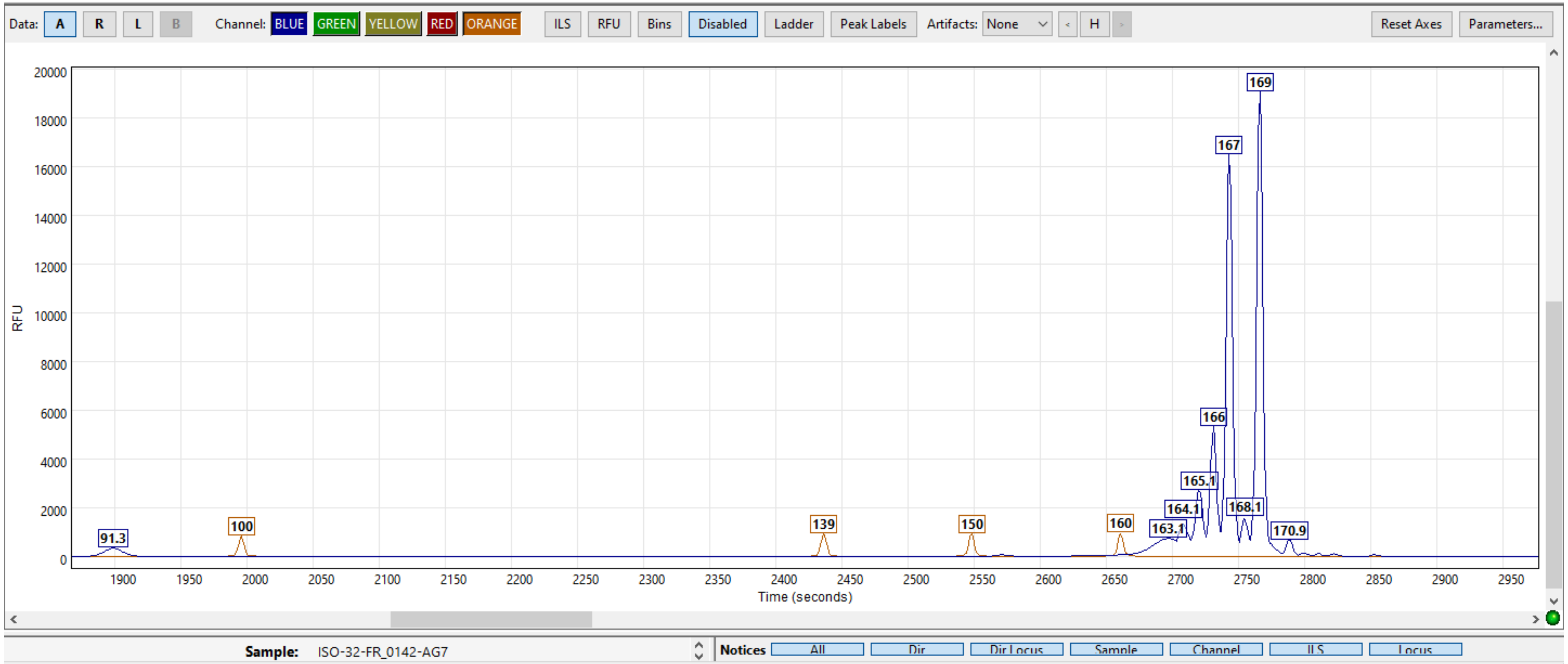


| | |
|---|---|
| **Observer 1** | **169 (167)** |
| **Observer 2** | **167;169** |
| **Observer 3** | **167;169** |

## 30- Colony. Locus ISO AG7 sample 33 (0143)

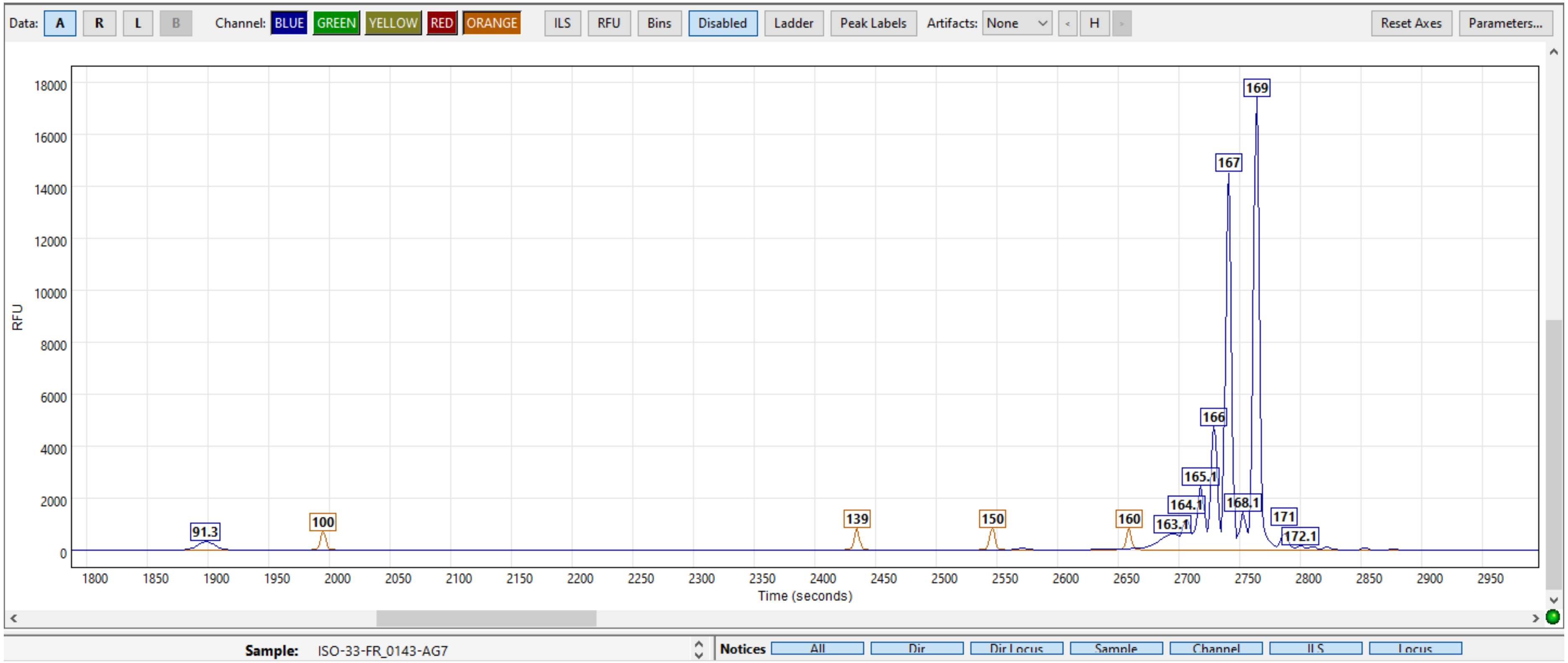


| | |
|---|---|
| **Observer 1** | **169 (167)** |
| **Observer 2** | **167;169** |
| **Observer 3** | **167;169** |

**AT1**

**1- Colony. Locus ISO AT1 sample 01 (0114)**

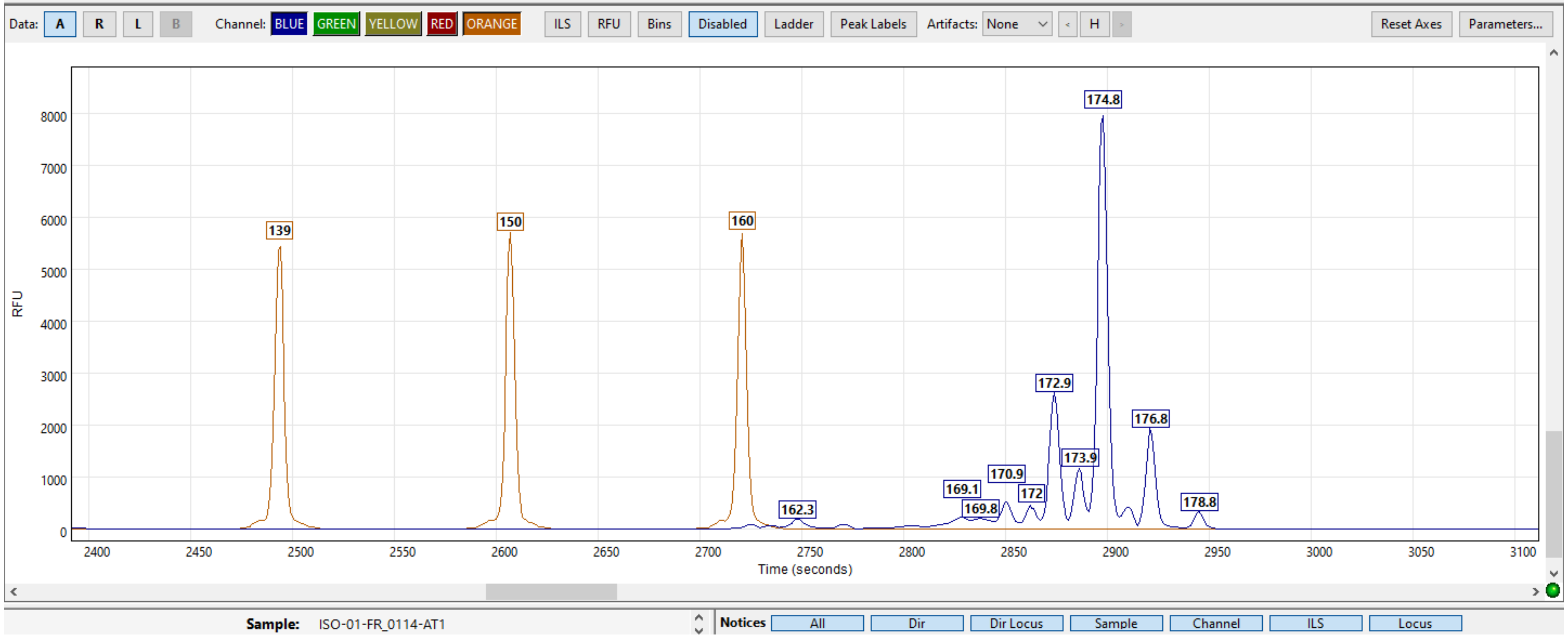


| | |
|---|---|
| **Observer 1** | **175 (173)** |
| **Observer 2** | **174.8** |
| **Observer 3** | **175** |

## 2- Colony. Locus ISO AT1 sample 02 (0115)

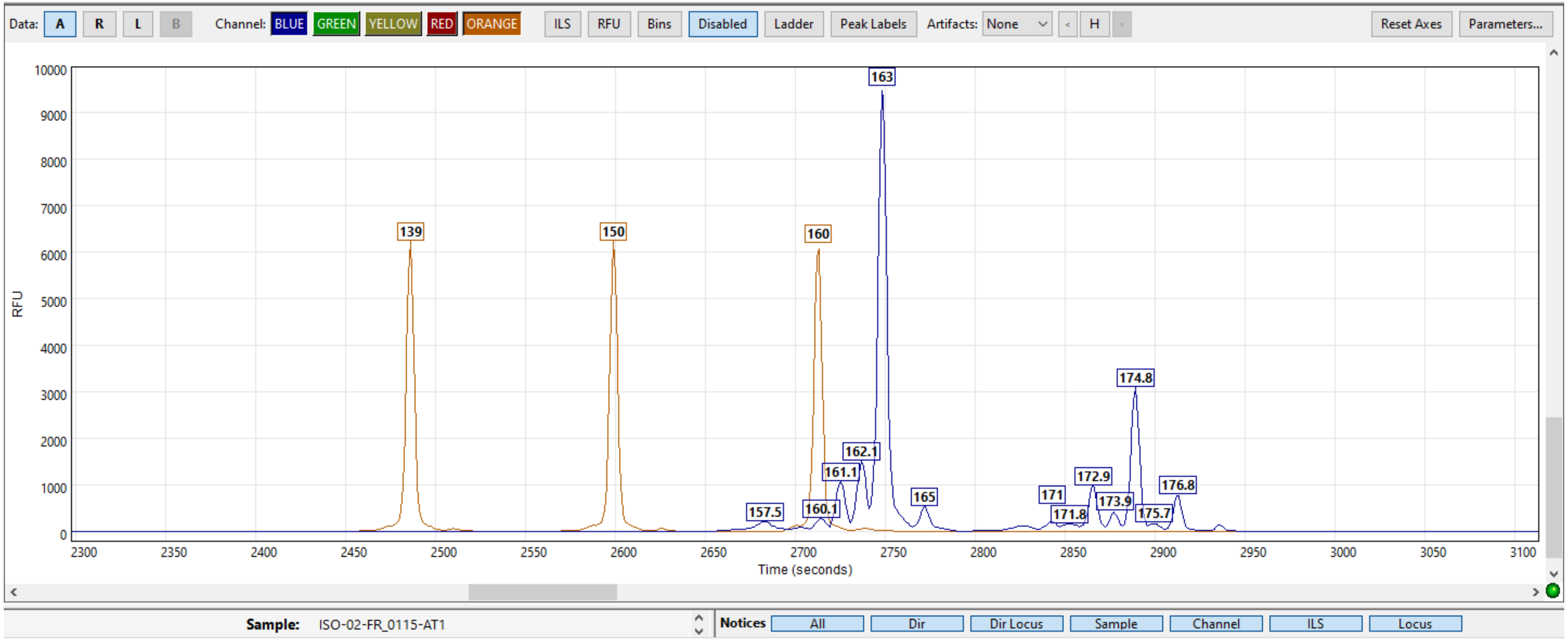


| | |
|---|---|
| **Observer 1** | **163;175** |
| **Observer 2** | **163;174.8** |
| **Observer 3** | **163;174.8** |

### 3- Colony. Locus ISO AT1 sample 03 (0116)

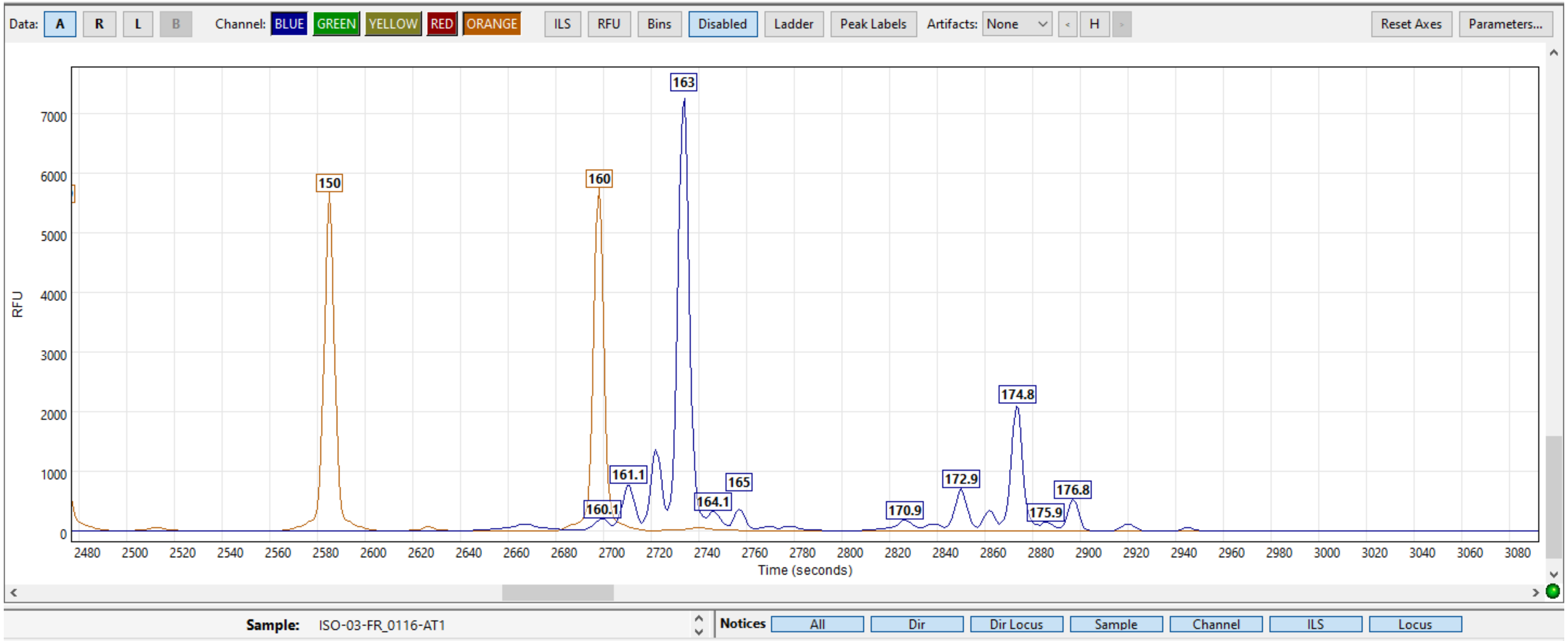


| | |
|---|---|
| **Observer 1** | **163;175** |
| **Observer 2** | **163;174.8** |
| **Observer 3** | **163;174.8** |

**4- Colony. Locus ISO AT1 sample 04 (0117)**

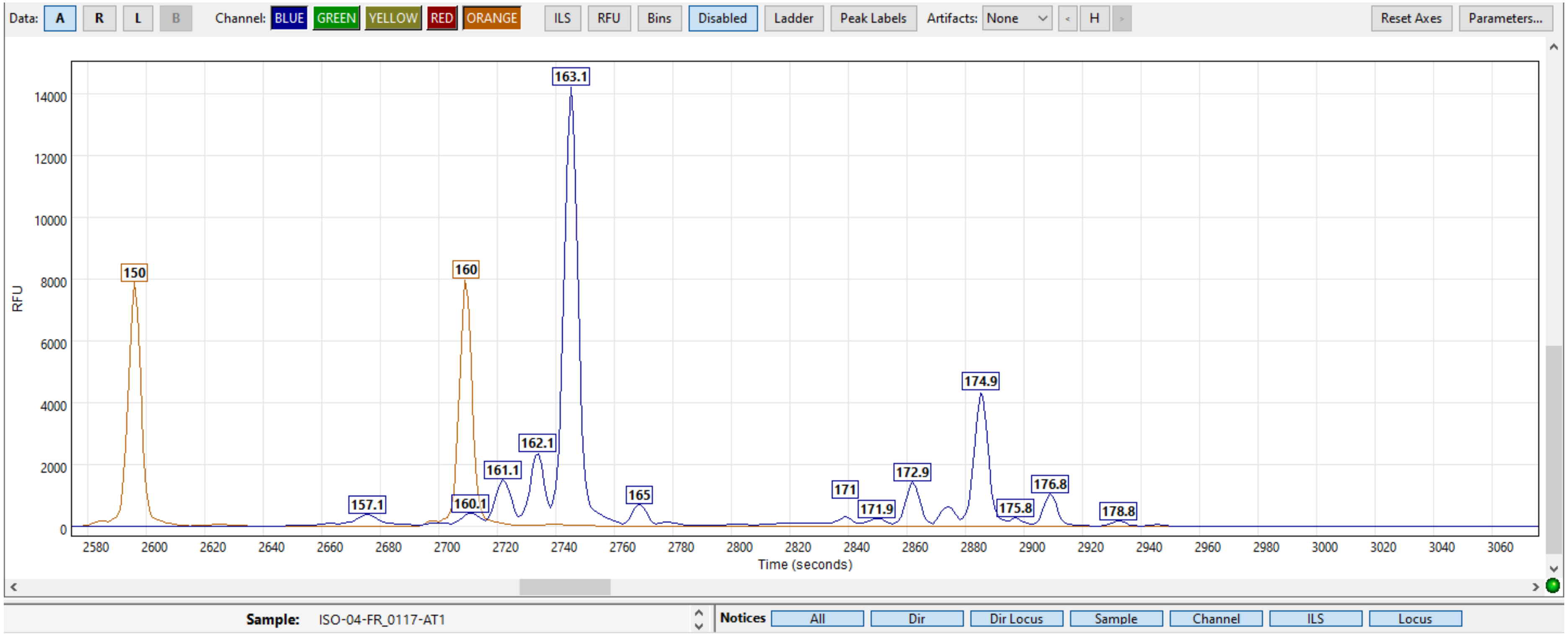


| | |
|---|---|
| **Observer 1** | **163;175** |
| **Observer 2** | **163.1;174.9** |
| **Observer 3** | **163; 175** |

## 5- Colony. Locus ISO AT1 sample 05 (0118)

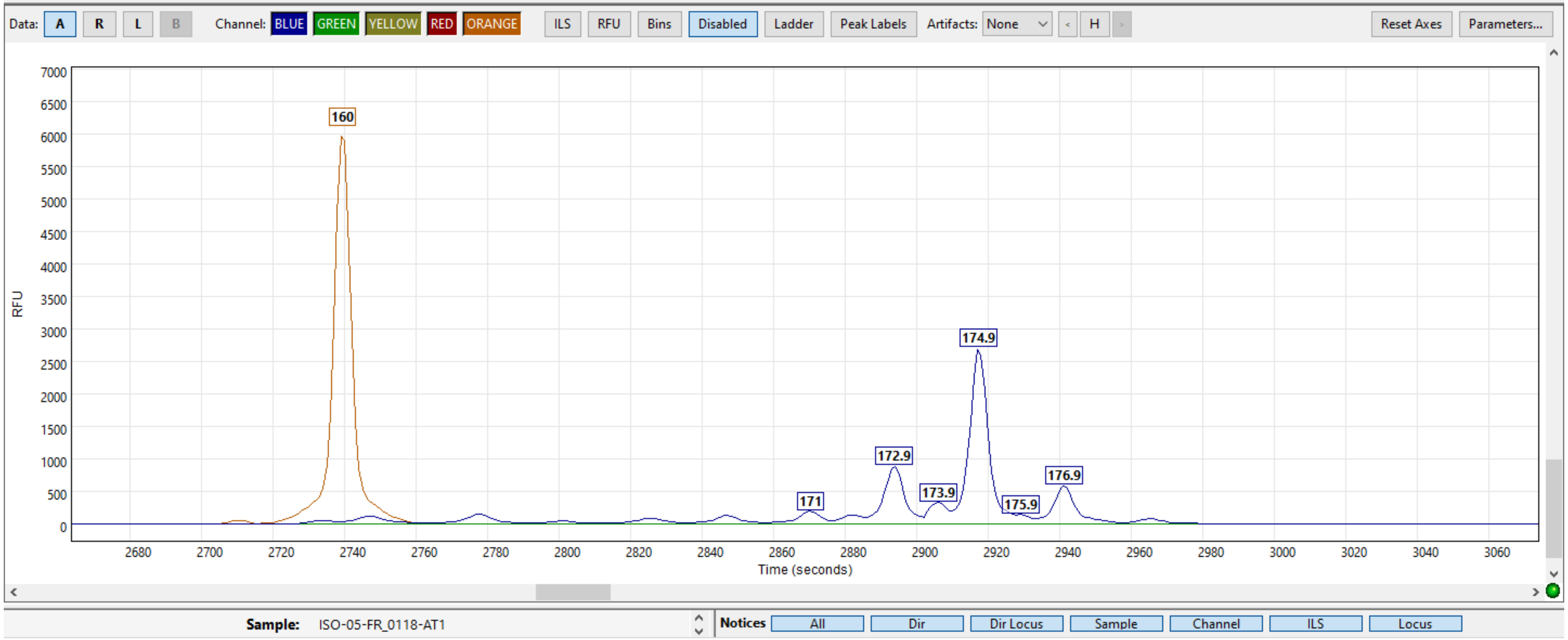


| | |
|---|---|
| **Observer 1** | **175** |
| **Observer 2** | **174.9** |
| **Observer 3** | **174.9** |

## 6- Colony. Locus ISO AT1 sample 06 (0119)

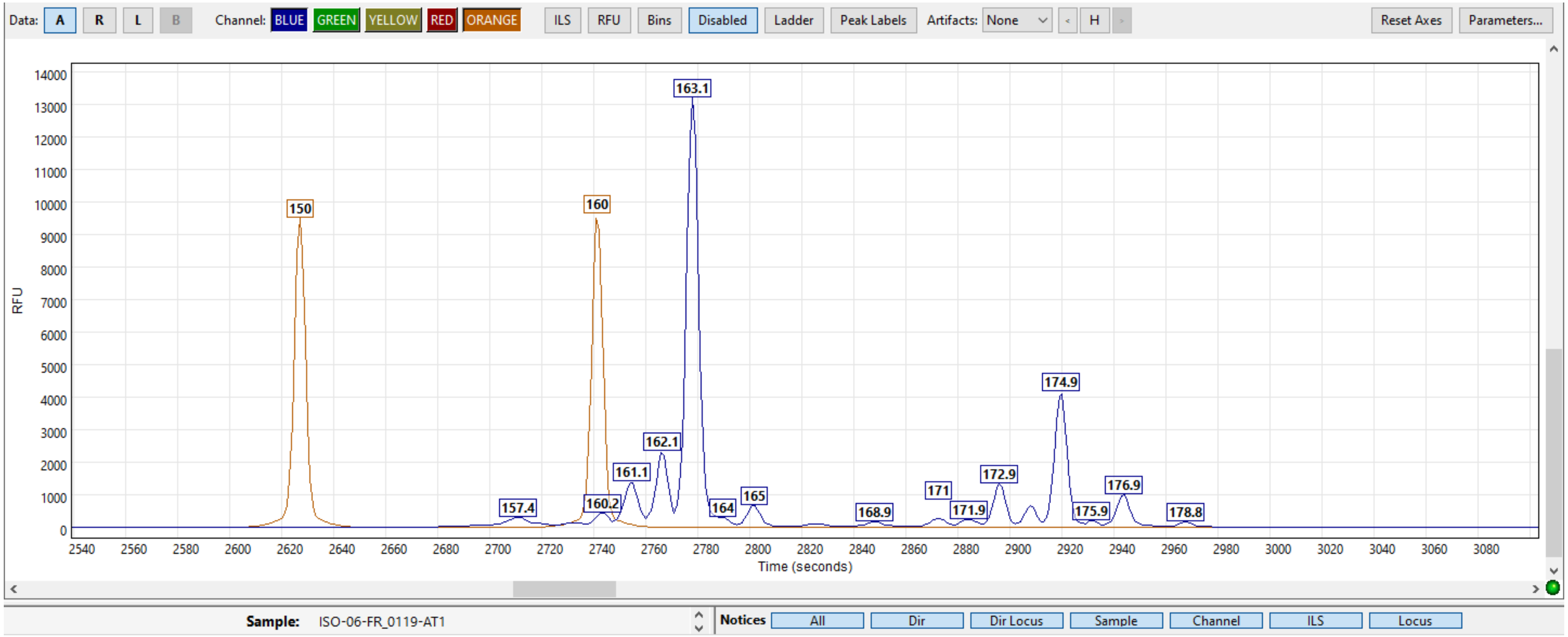


| | |
|---|---|
| **Observer 1** | **163;175** |
| **Observer 2** | **163.1;174.9** |
| **Observer 3** | **163.1;174.9** |

### 7- Colony. Locus ISO AT1 sample 07 (0120)

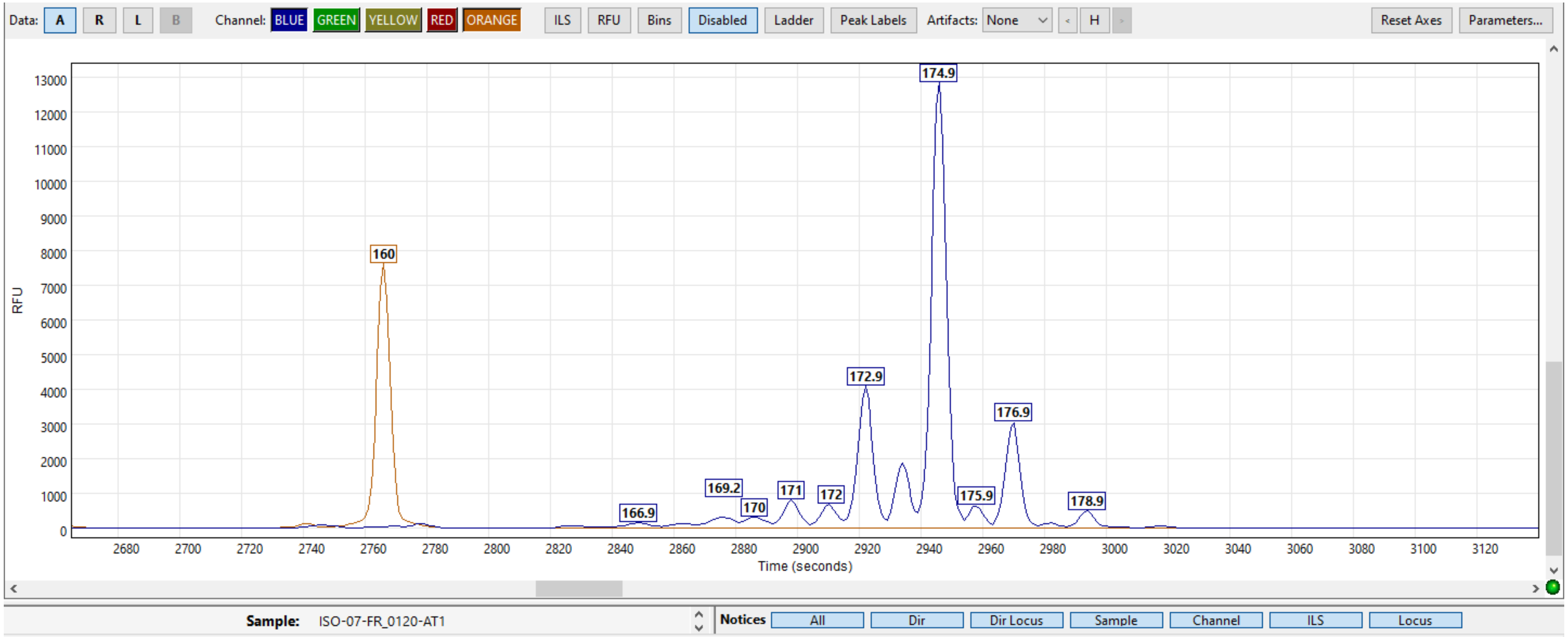


| | |
|---|---|
| **Observer 1** | **175** |
| **Observer 2** | **174.9** |
| **Observer 3** | **175** |

**8- Colony. Locus ISO AT1 sample 08 (0121)**

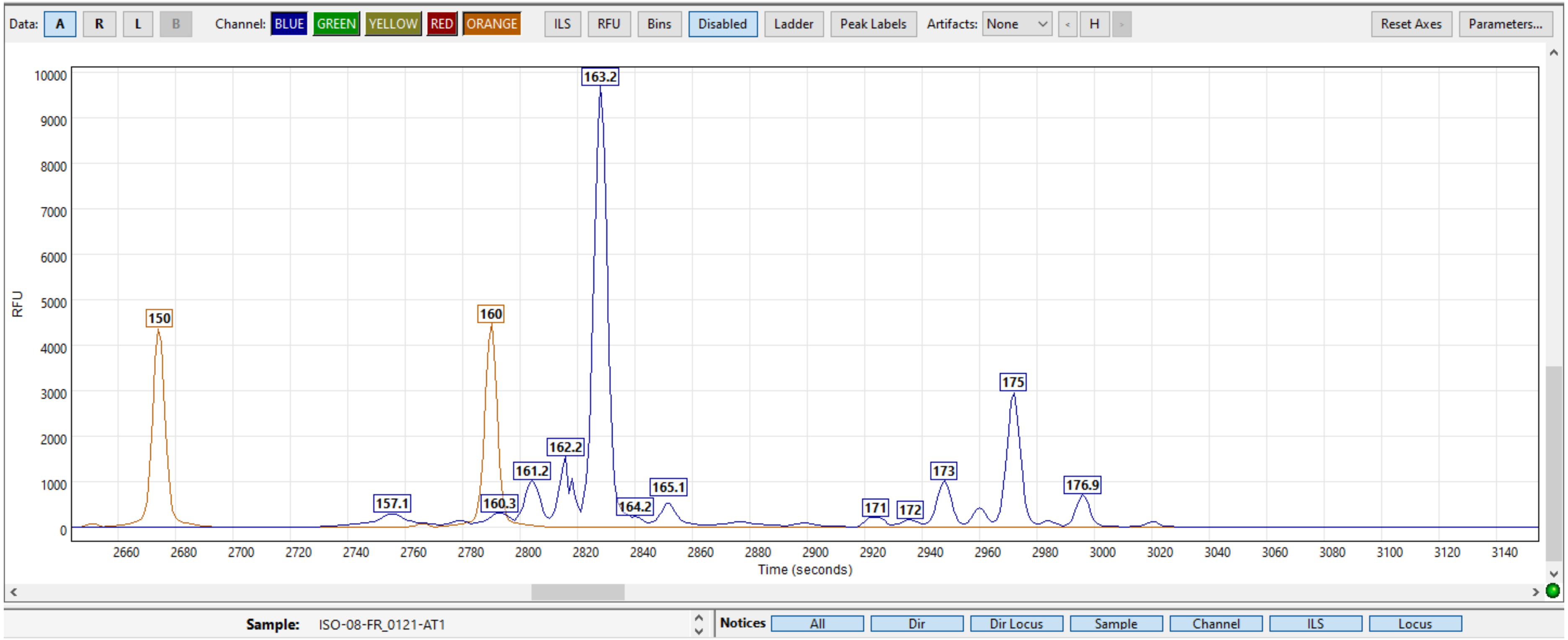


| | |
|---|---|
| **Observer 1** | **163;175** |
| **Observer 2** | **163.2;175** |
| **Observer 3** | **163.2;175** |

**9- Colony. Locus ISO AT1 sample 09 (0122)**

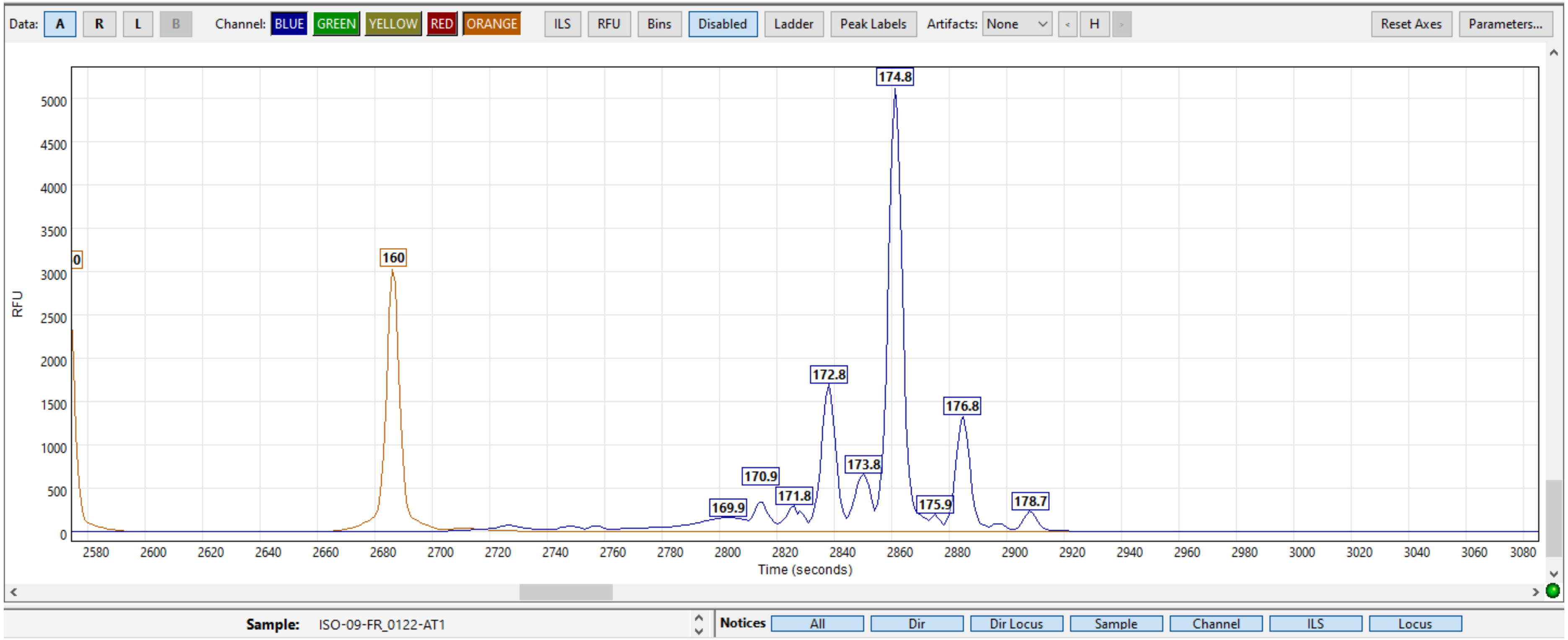


| | |
|---|---|
| **Observer 1** | **175** |
| **Observer 2** | **174.8** |
| **Observer 3** | **174.8** |

**10- Colony. Locus ISO AT1 sample 10 (0123)**

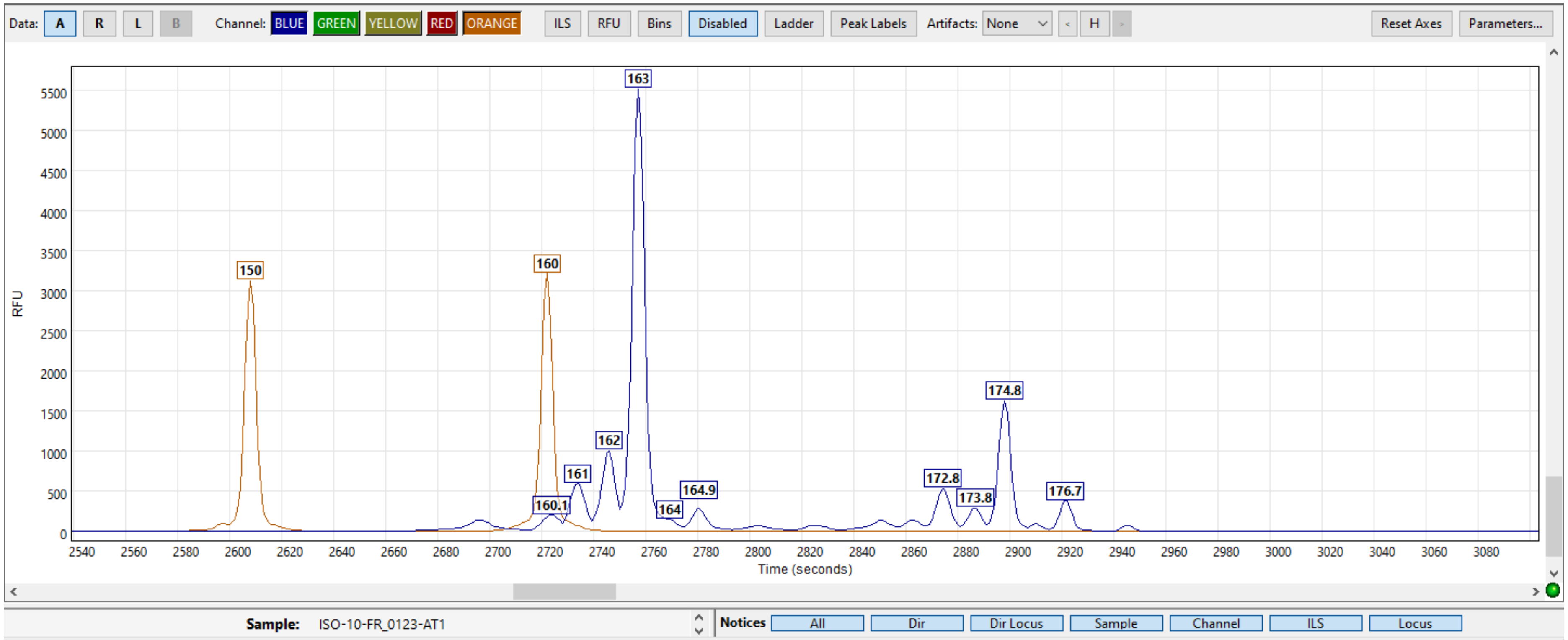


| | |
|---|---|
| **Observer 1** | **163;175** |
| **Observer 2** | **163;174.8** |
| **Observer 3** | **163;174.8** |

**11- Colony. Locus ISO AT1 sample 11 (0124)**

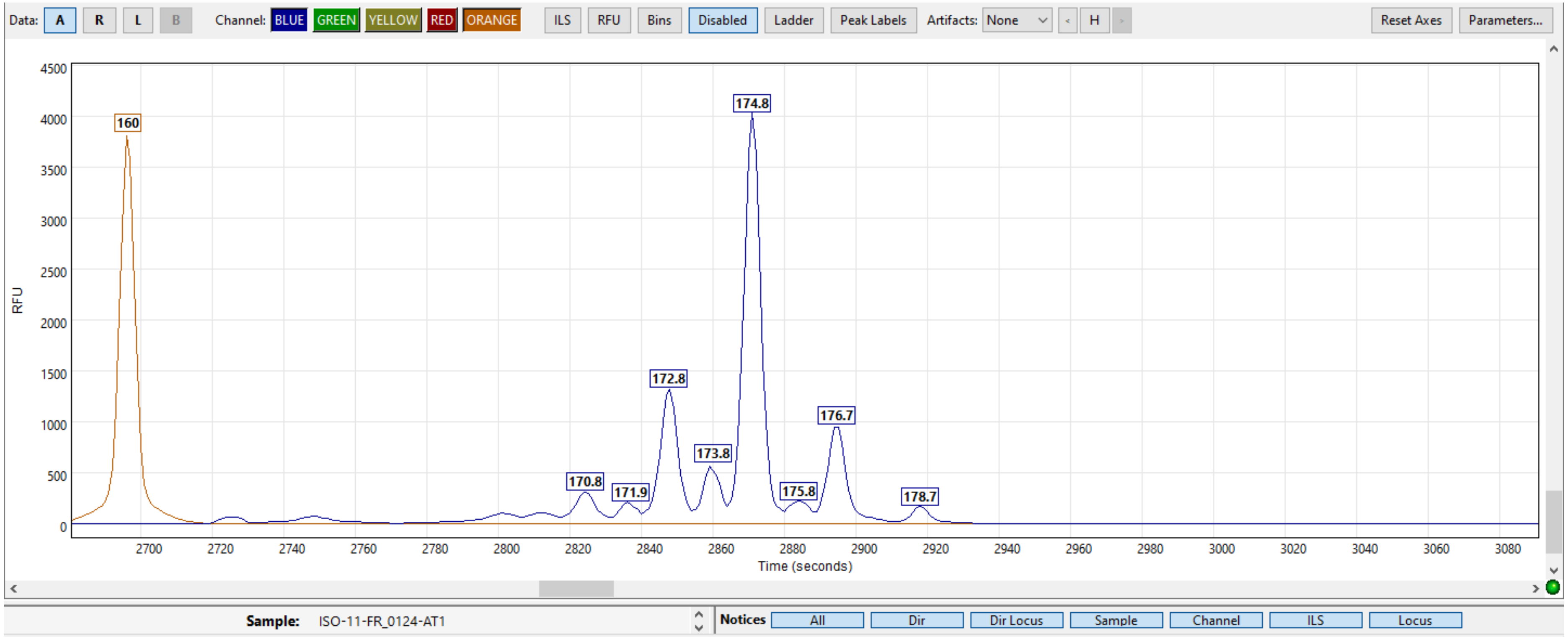


| | |
|---|---|
| **Observer 1** | **175** |
| **Observer 2** | **174.8** |
| **Observer 3** | **174.8** |

## 12- Colony. Locus ISO AT1 sample 12 (0125)

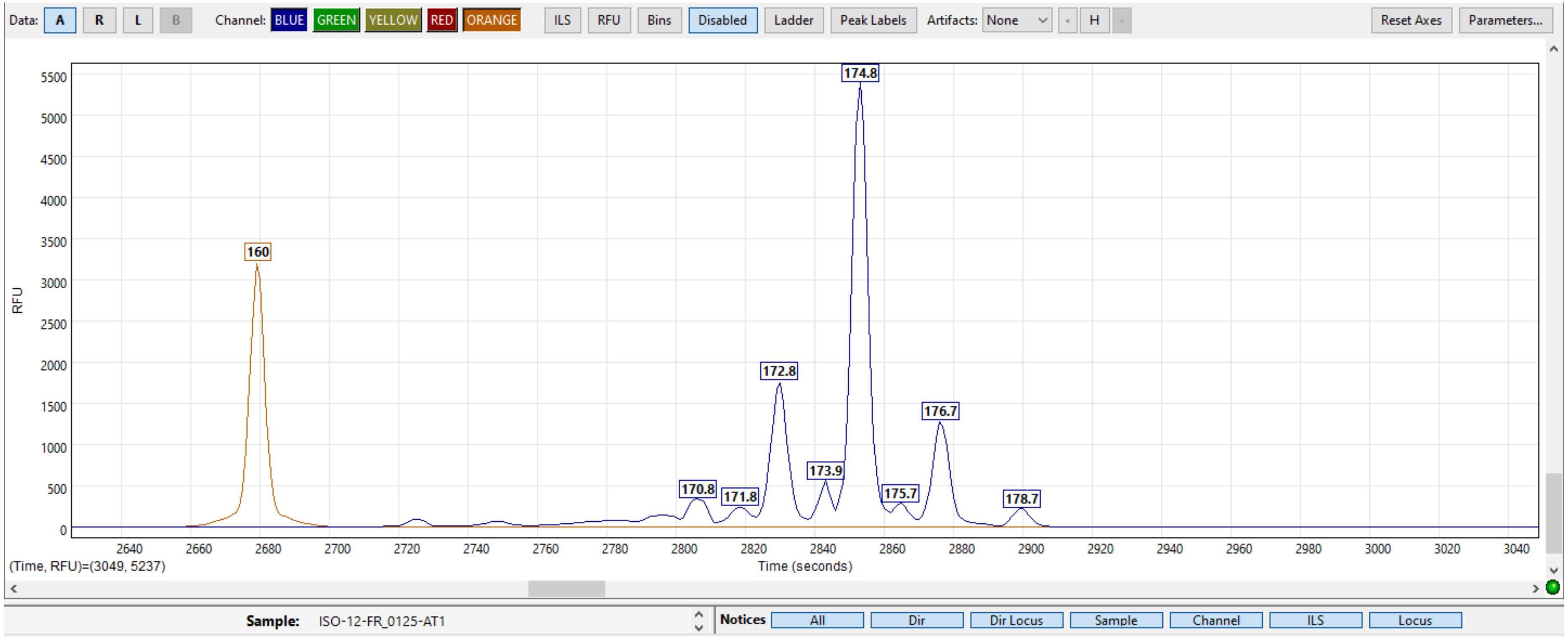


| | |
|---|---|
| **Observer 1** | **175** |
| **Observer 2** | **174.8** |
| **Observer 3** | **174.8** |

**13- Colony. Locus ISO AT1 sample 13 (0126)**

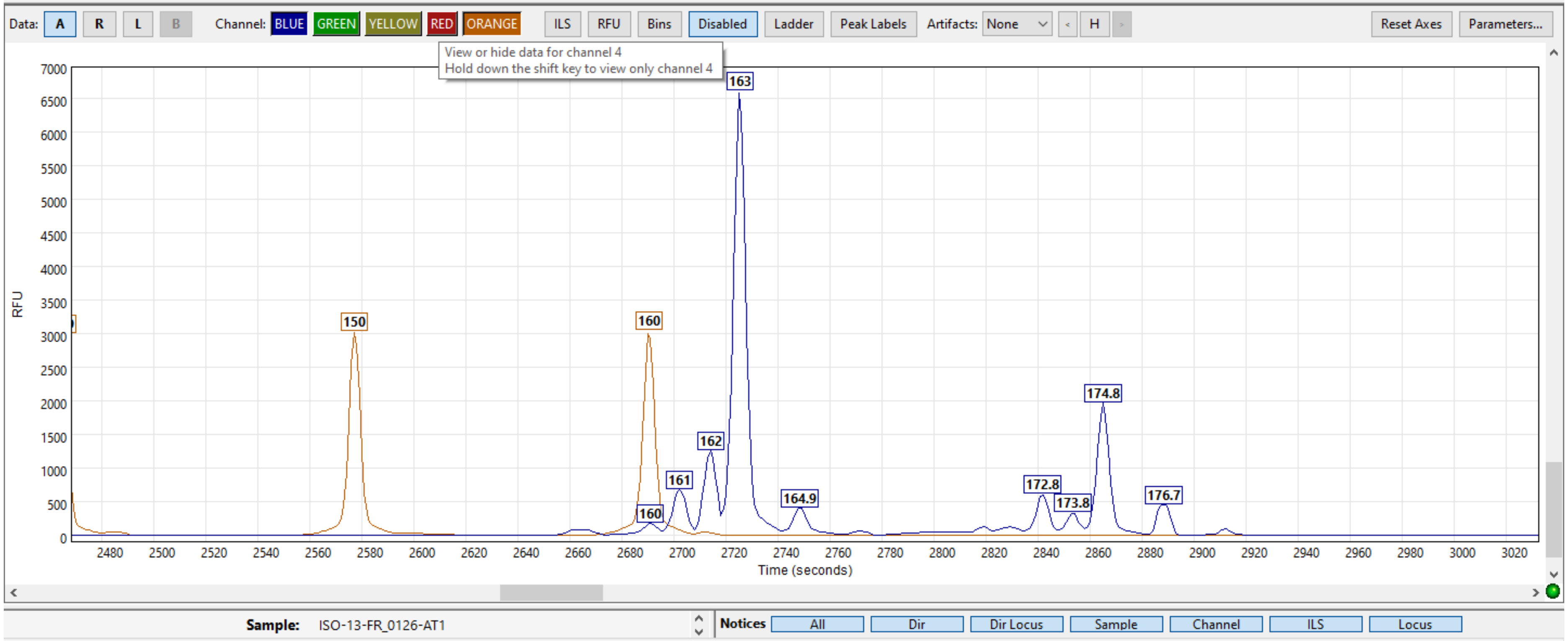


| | |
|---|---|
| **Observer 1** | **163;175** |
| **Observer 2** | **163;174.8** |
| **Observer 3** | **163;174.8** |

### 14- Colony. Locus ISO AT1 sample 14 (0127)

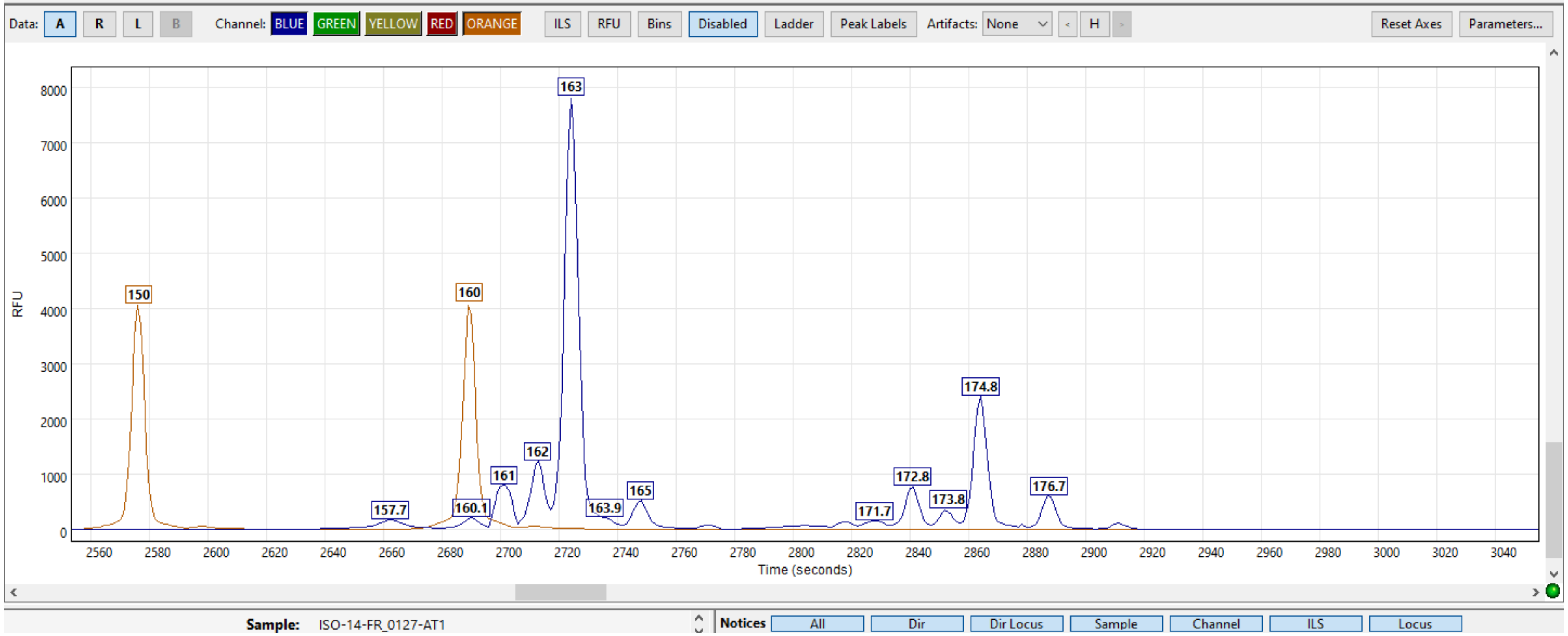


| | |
|---|---|
| **Observer 1** | **163;175** |
| **Observer 2** | **163;174.8** |
| **Observer 3** | **163;174.8** |

## 15- Colony. Locus ISO AT1 sample 15 (0128)

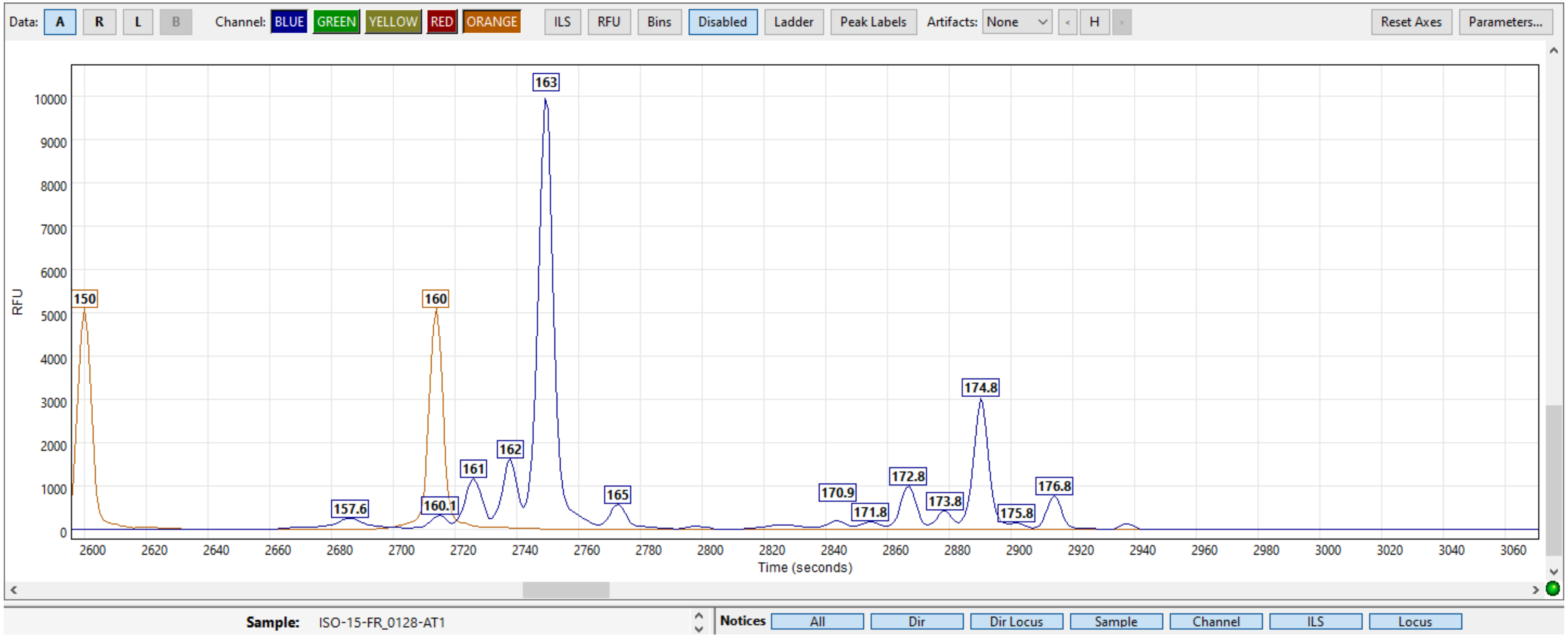


| | |
|---|---|
| **Observer 1** | **163;175** |
| **Observer 2** | **163;174.8** |
| **Observer 3** | **163;174.8** |

## 16- Colony. Locus ISO AT1 sample 16 (0129)

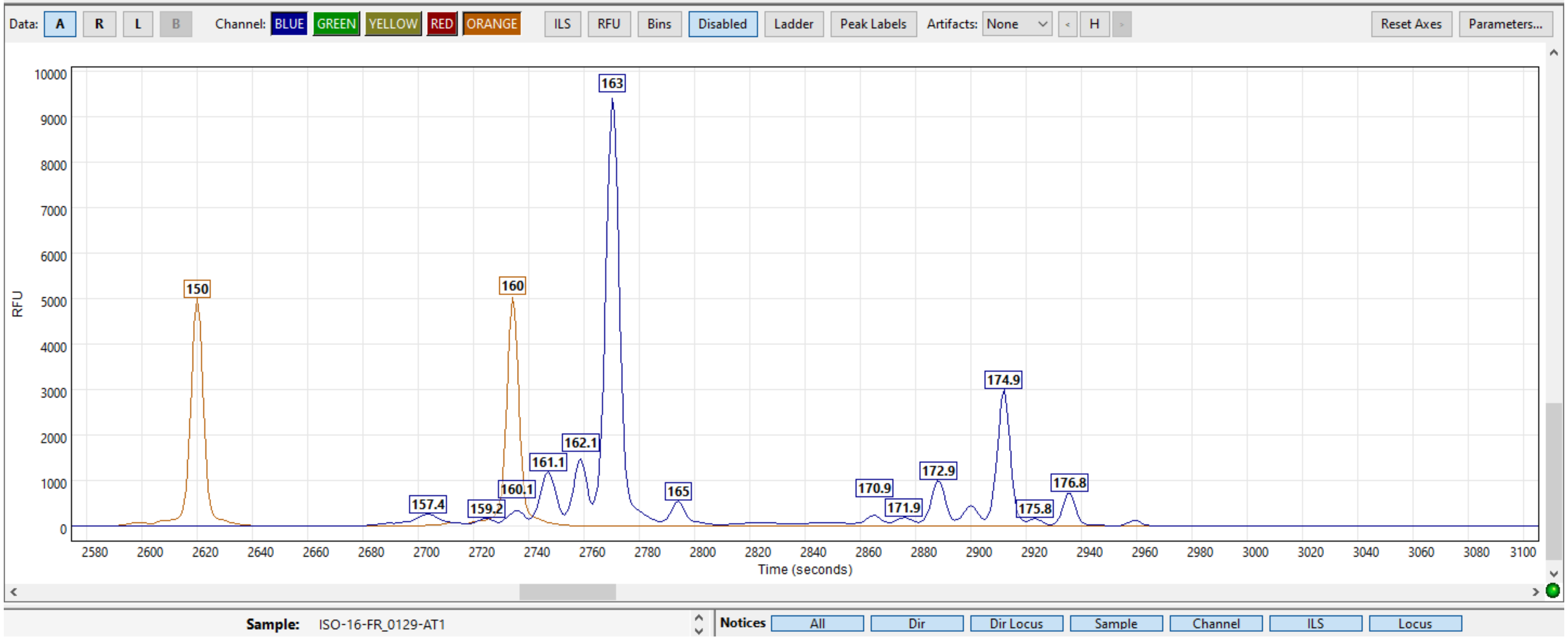


| | |
|---|---|
| **Observer 1** | **163;175** |
| **Observer 2** | **163;174.9** |
| **Observer 3** | **163;174.9** |

**17- Colony. Locus ISO AT1 sample 17 (0130)**

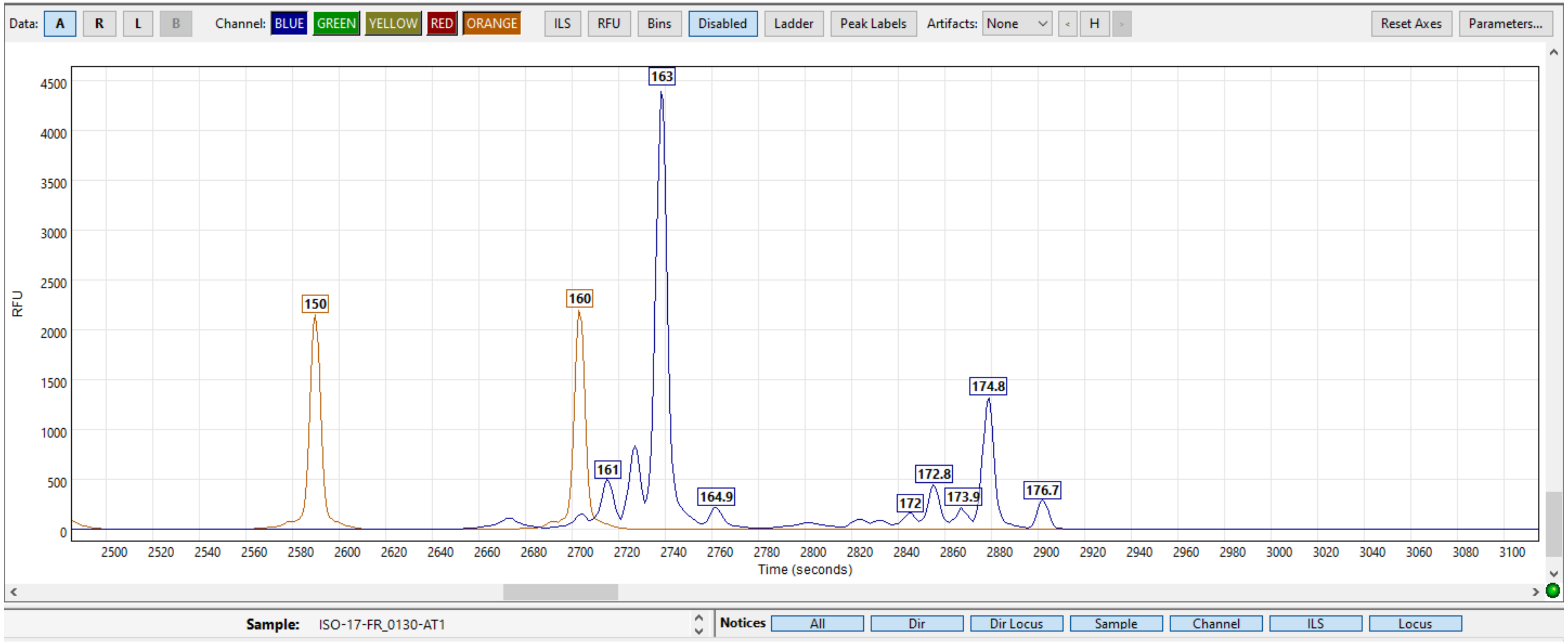


| | |
|---|---|
| **Observer 1** | **163;175** |
| **Observer 2** | **163;174.8** |
| **Observer 3** | **163;174.8** |

### 18- Colony. Locus ISO AT1 sample 19 (0131)

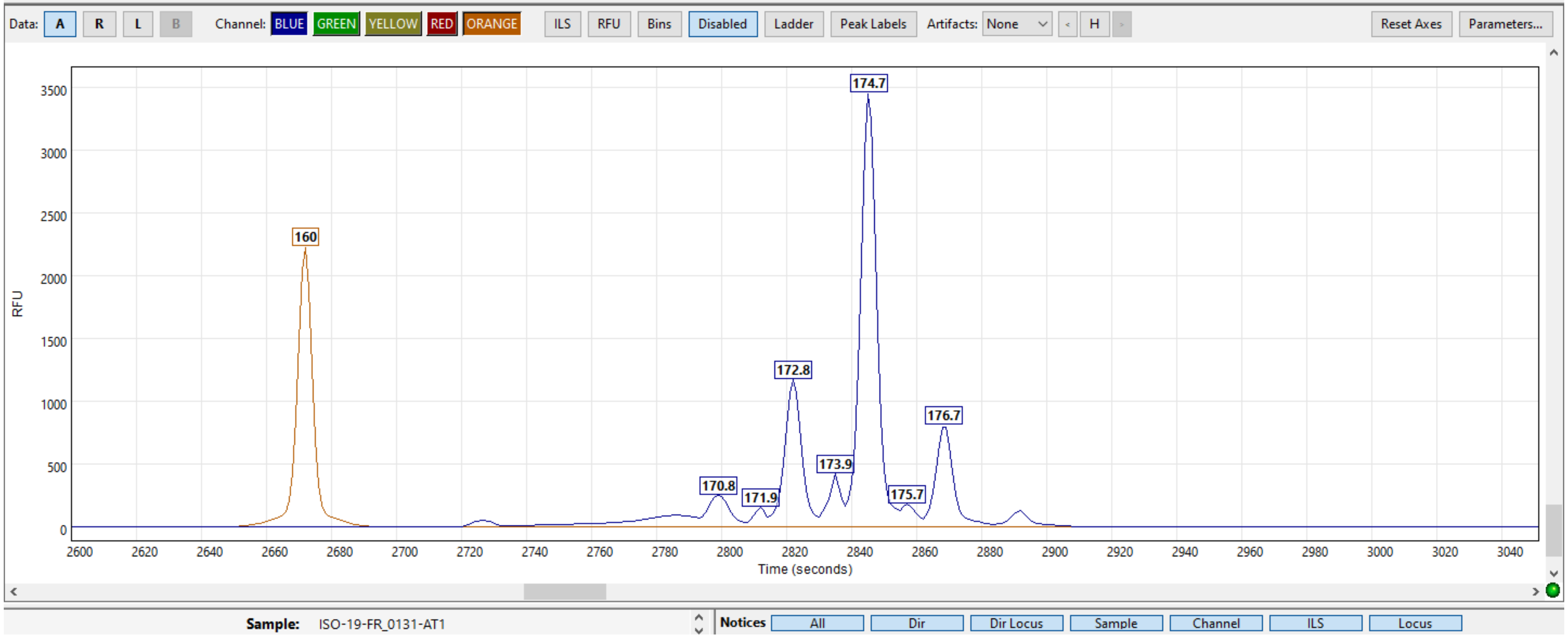


| | |
|---|---|
| **Observer 1** | **175** |
| **Observer 2** | **174.7** |
| **Observer 3** | **174.7** |

### 19- Colony. Locus ISO AT1 sample 20 (0132)

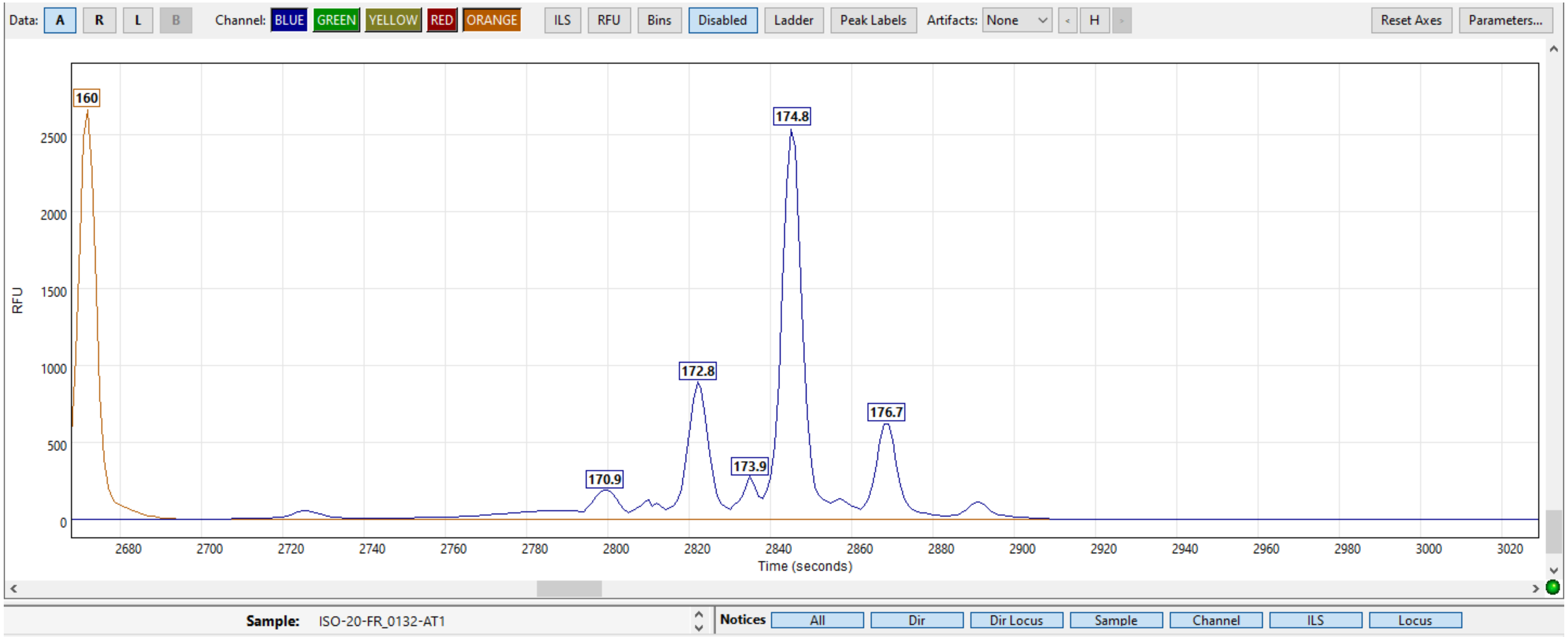


| | |
|---|---|
| **Observer 1** | **175** |
| **Observer 2** | **174.8** |
| **Observer 3** | **174.8** |

## 20- Colony. Locus ISO AT1 sample 21 (0133)

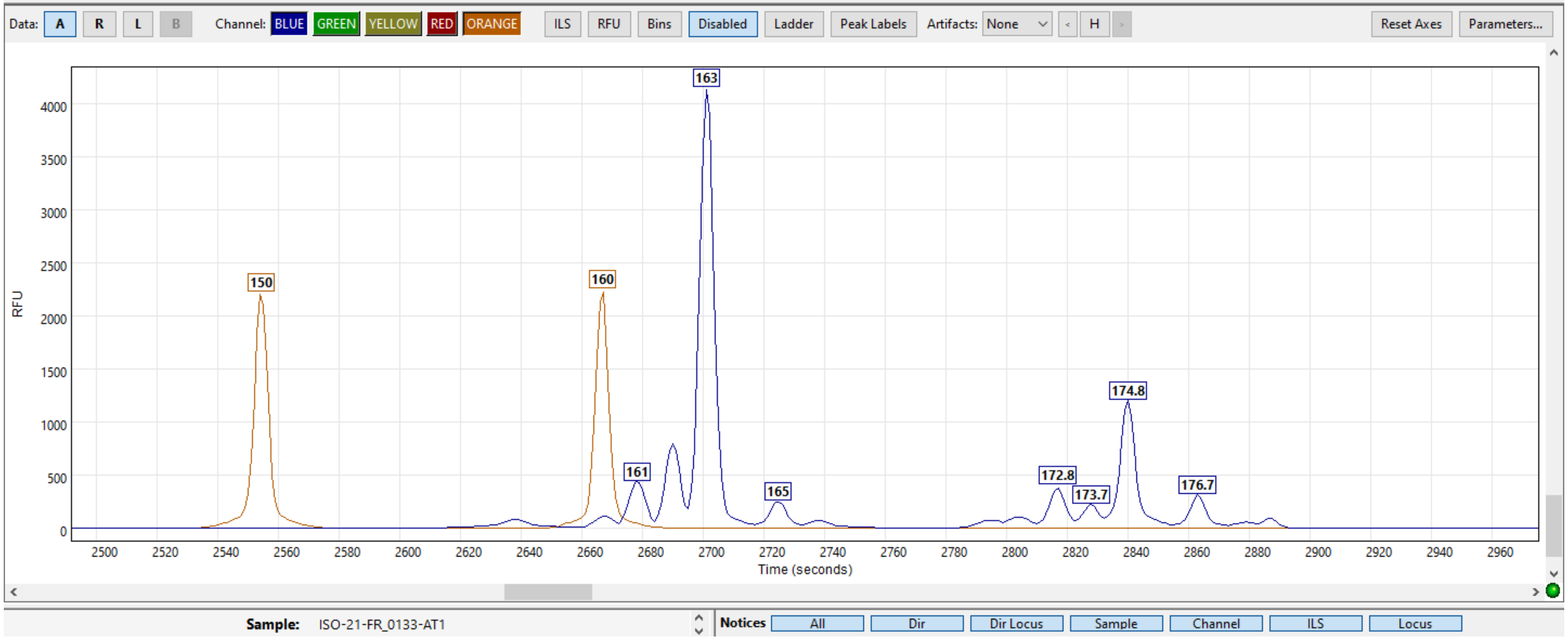


| | |
|---|---|
| **Observer 1** | **163;175** |
| **Observer 2** | **163;174.8** |
| **Observer 3** | **163;174.8** |

**21- Colony. Locus ISO AT1 sample 23 (0134)**

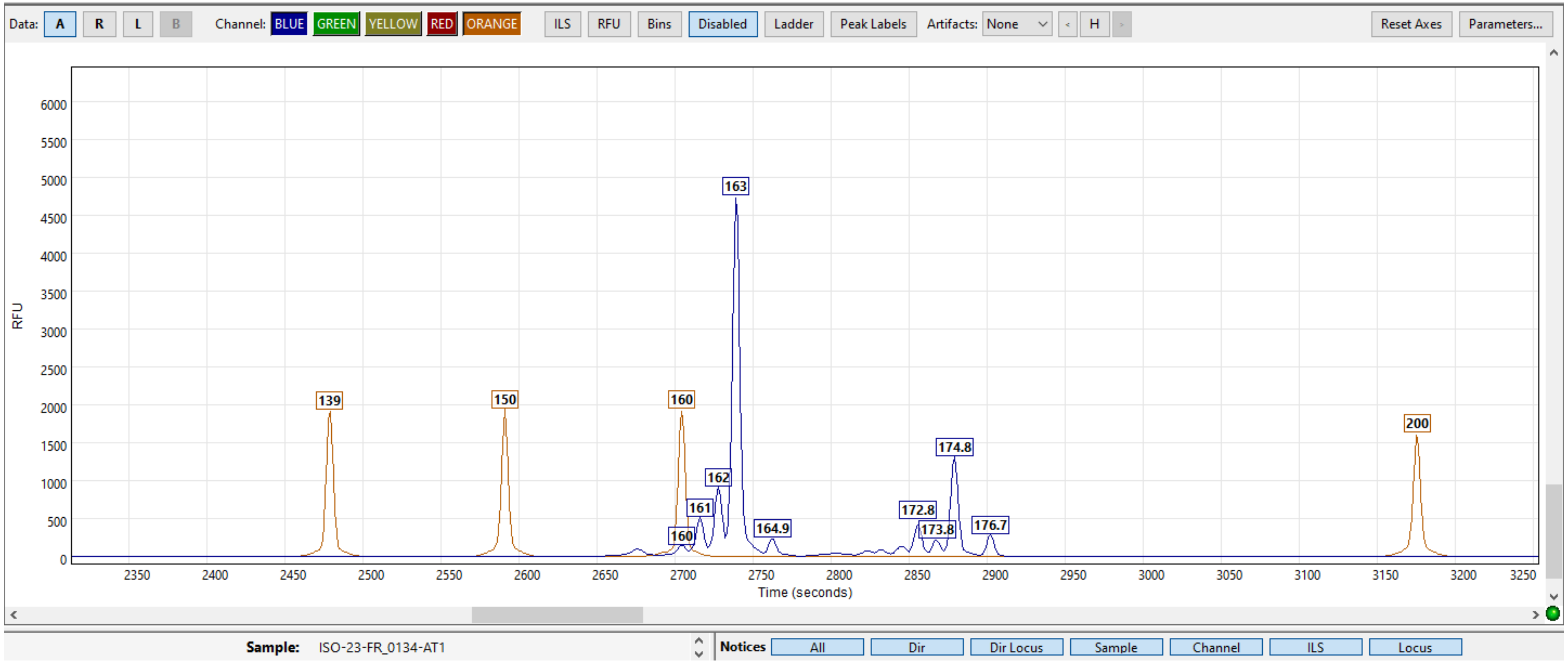


| | |
|---|---|
| **Observer 1** | **163;175** |
| **Observer 2** | **163;174.8** |
| **Observer 3** | **163;174.8** |

## 22- Colony. Locus ISO AT1 sample 24 (0135)

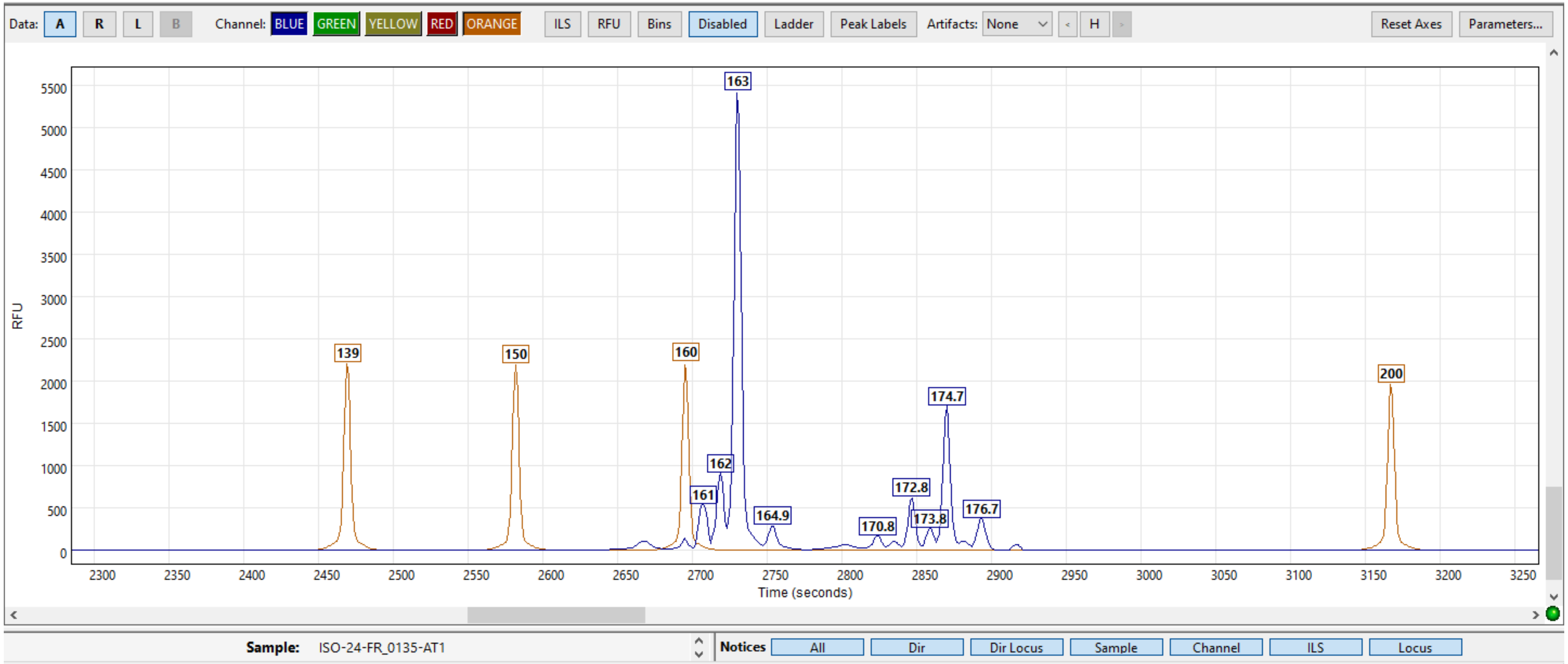


| | |
|---|---|
| **Observer 1** | **163;175** |
| **Observer 2** | **163;174.7** |
| **Observer 3** | **163;174.7** |

## 23- Colony. Locus ISO AT1 sample 25 (0136)

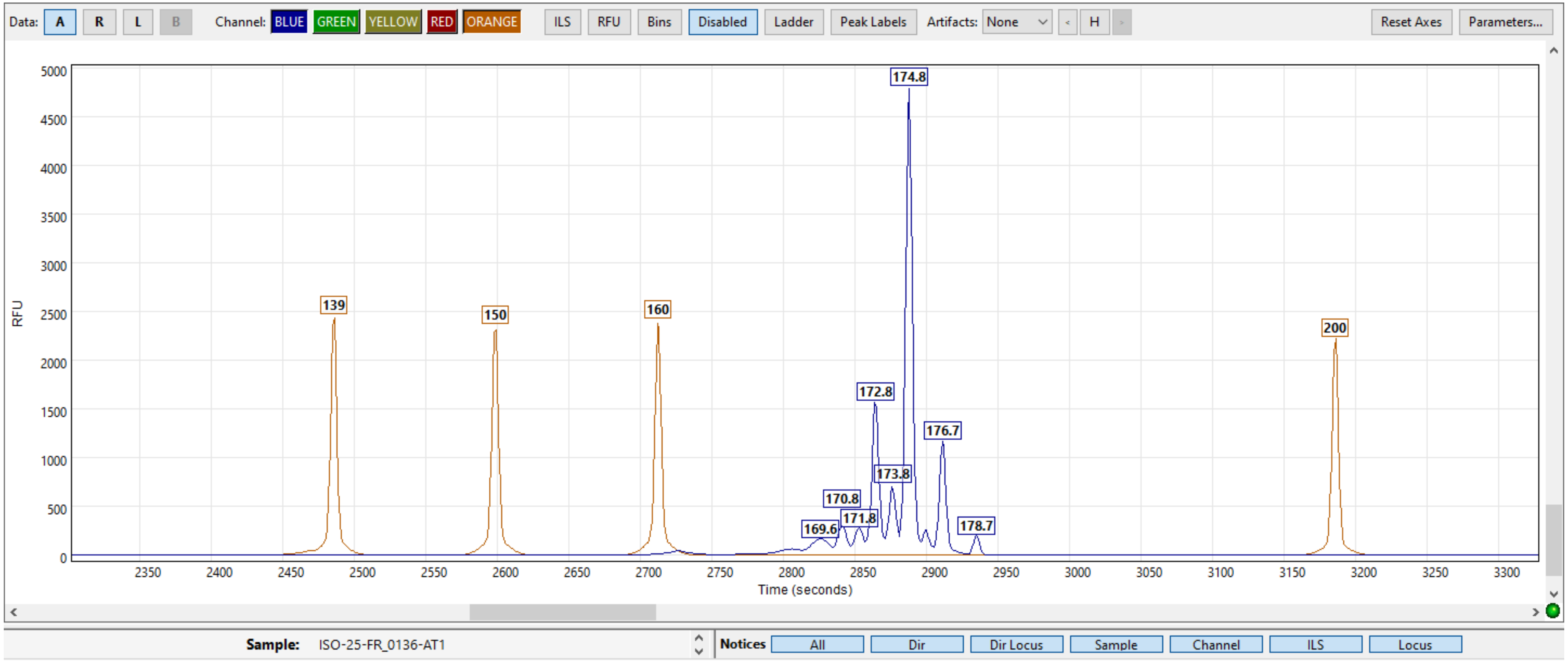


| | |
|---|---|
| **Observer 1** | **175** |
| **Observer 2** | **174.8** |
| **Observer 3** | **174.8** |

## 24- Colony. Locus ISO AT1 sample 26 (0137)

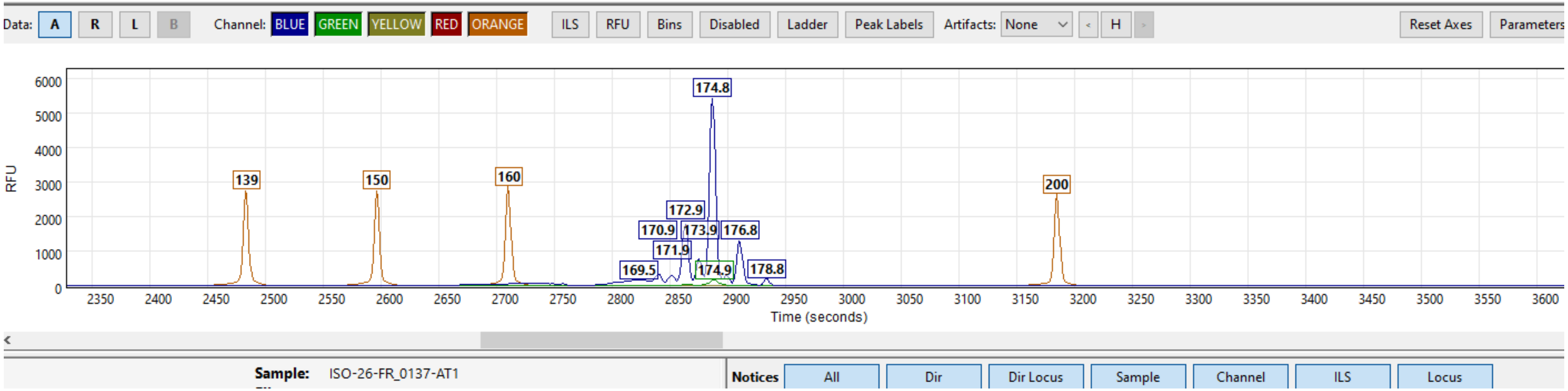


| | |
|---|---|
| **Observer 1** | **175** |
| **Observer 2** | **174.8** |
| **Observer 3** | **174.8** |

## 25- Colony. Locus ISO AT1 sample 27 (0138)

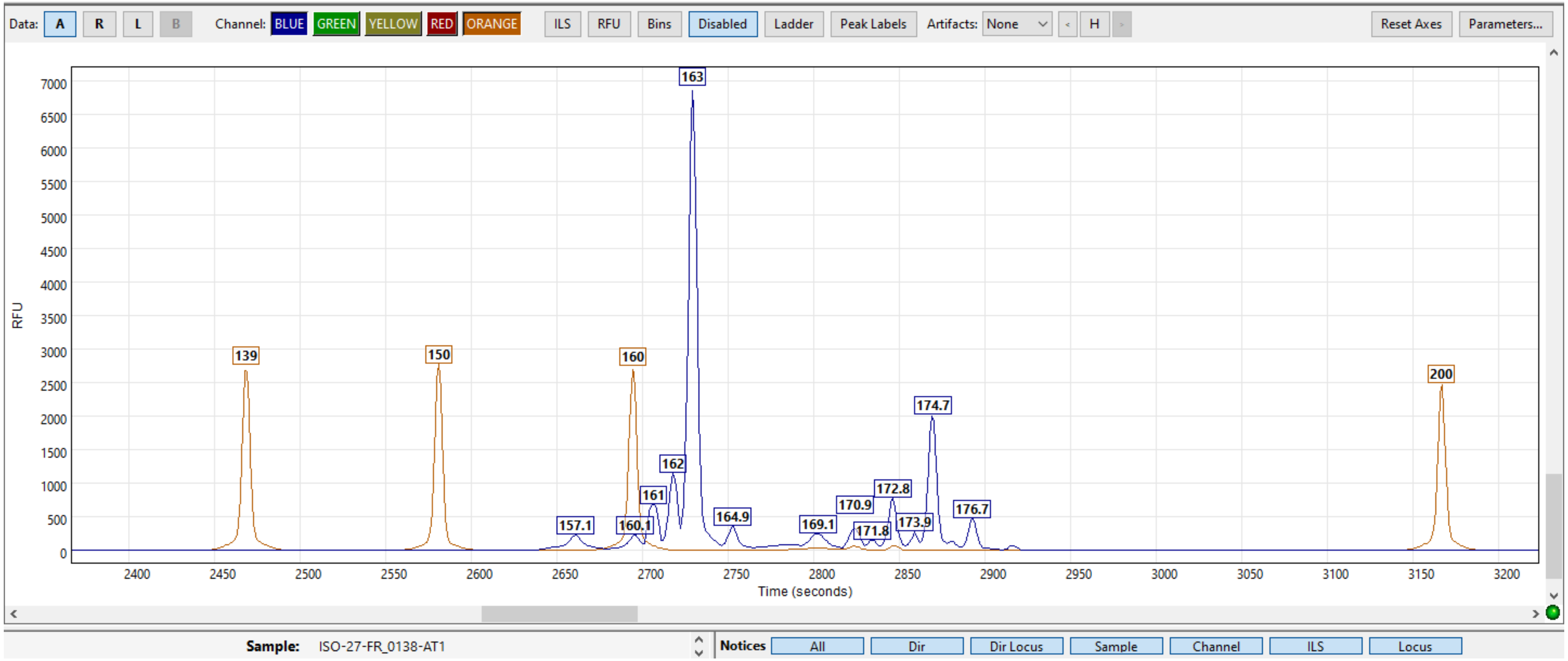


| | |
|---|---|
| **Observer 1** | **163;175** |
| **Observer 2** | **163;174.7** |
| **Observer 3** | **163;174.7** |

**26- Colony. Locus ISO AT1 sample 29 (0139)**

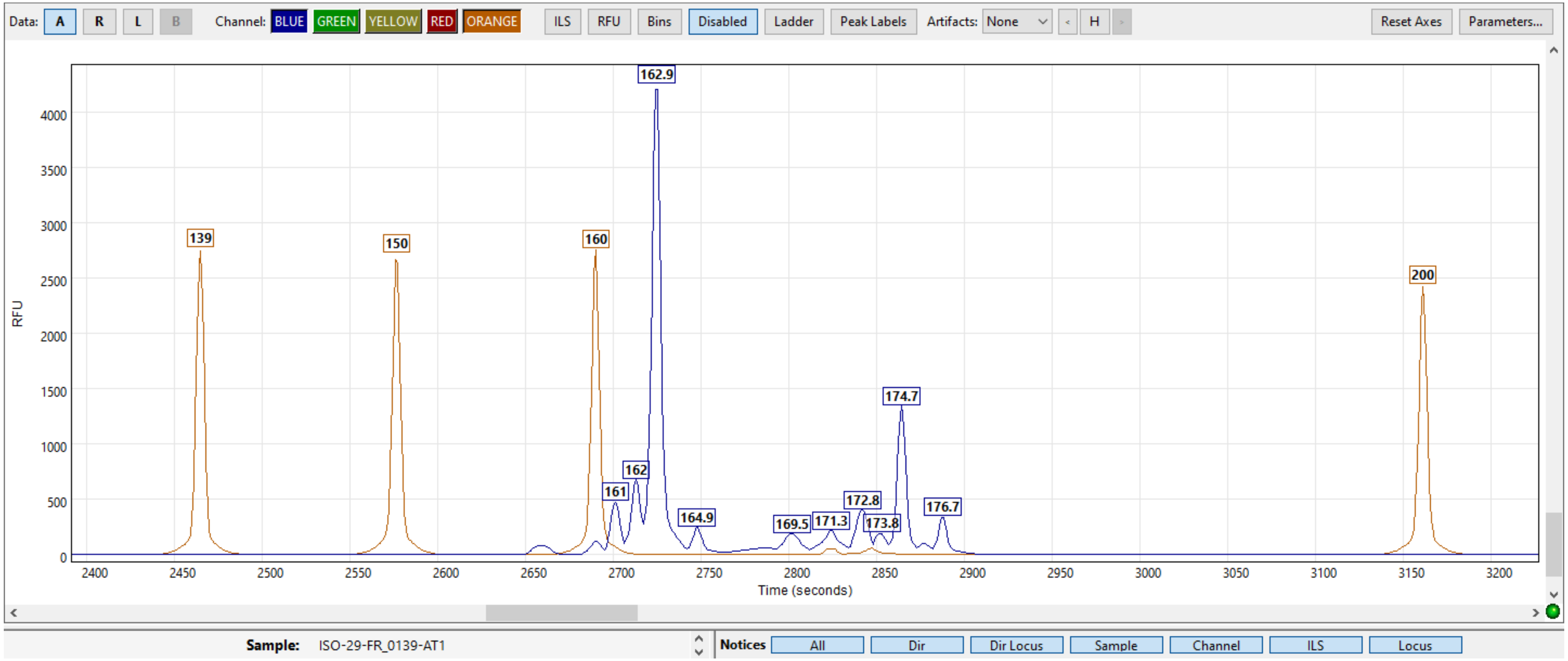


| | |
|---|---|
| **Observer 1** | **163;175** |
| **Observer 2** | **162.9;174.7** |
| **Observer 3** | **162.9;174.7** |

**27- Colony. Locus ISO AT1 sample 30 (0140)**

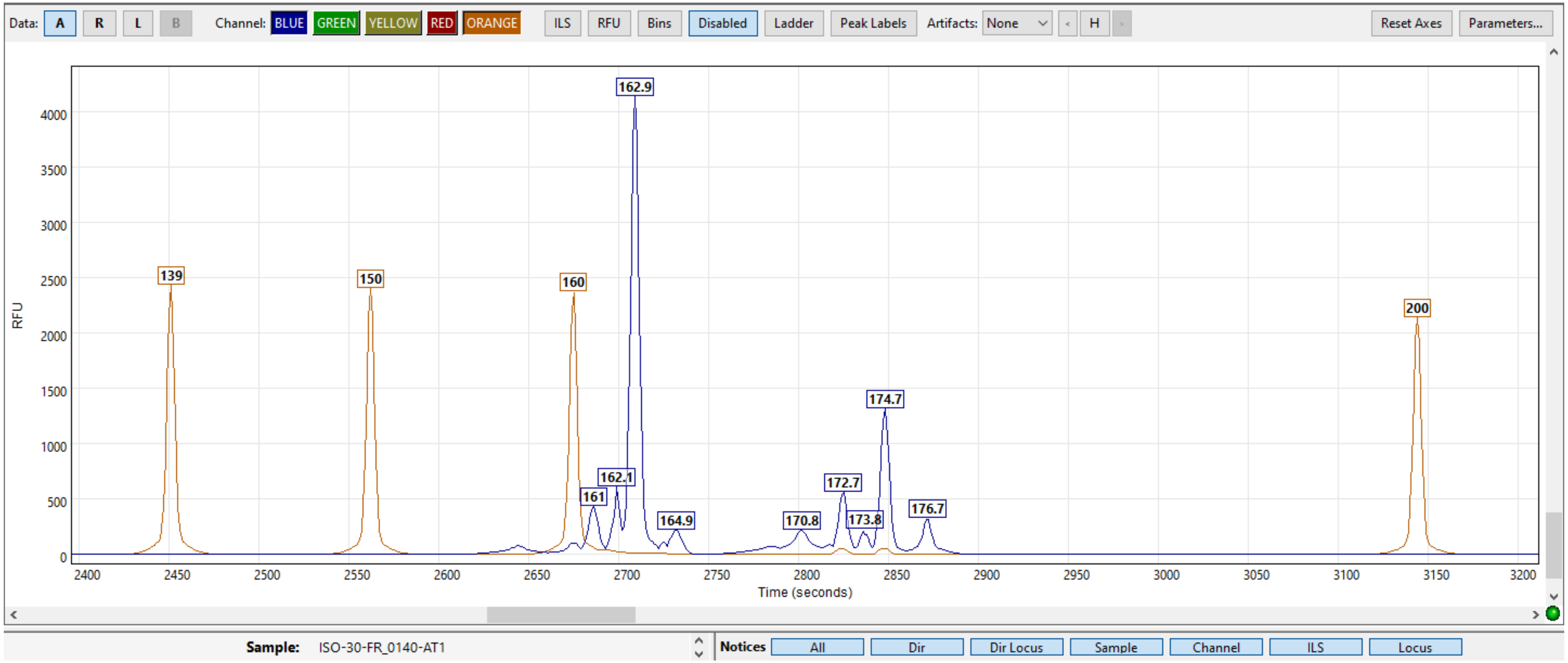


| | |
|---|---|
| **Observer 1** | **163;175** |
| **Observer 2** | **162.9;174.7** |
| **Observer 3** | **163;175** |

## 28- Colony. Locus ISO AT1 sample 31 (0141)

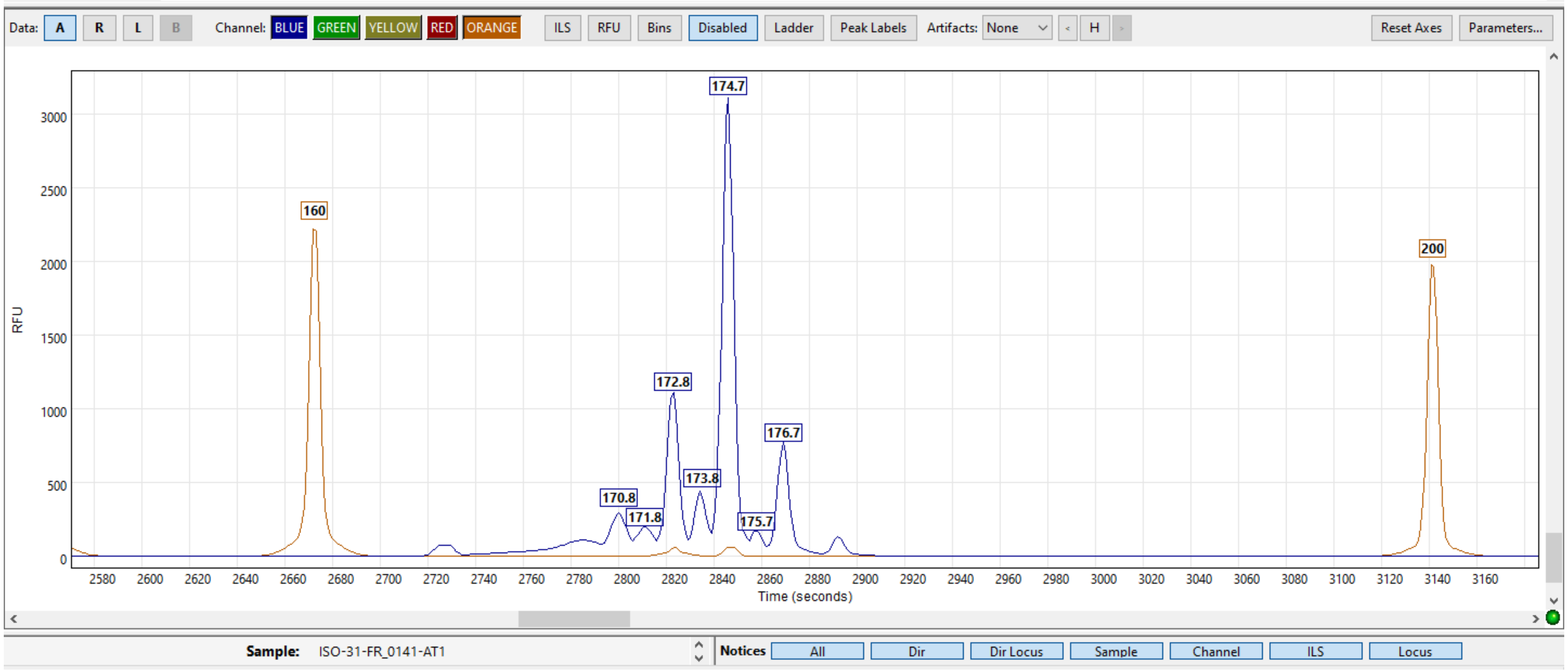


| | |
|---|---|
| **Observer 1** | **175** |
| **Observer 2** | **174.7** |
| **Observer 3** | **175** |

**29- Colony. Locus ISO AT1 sample 32 (0142)**

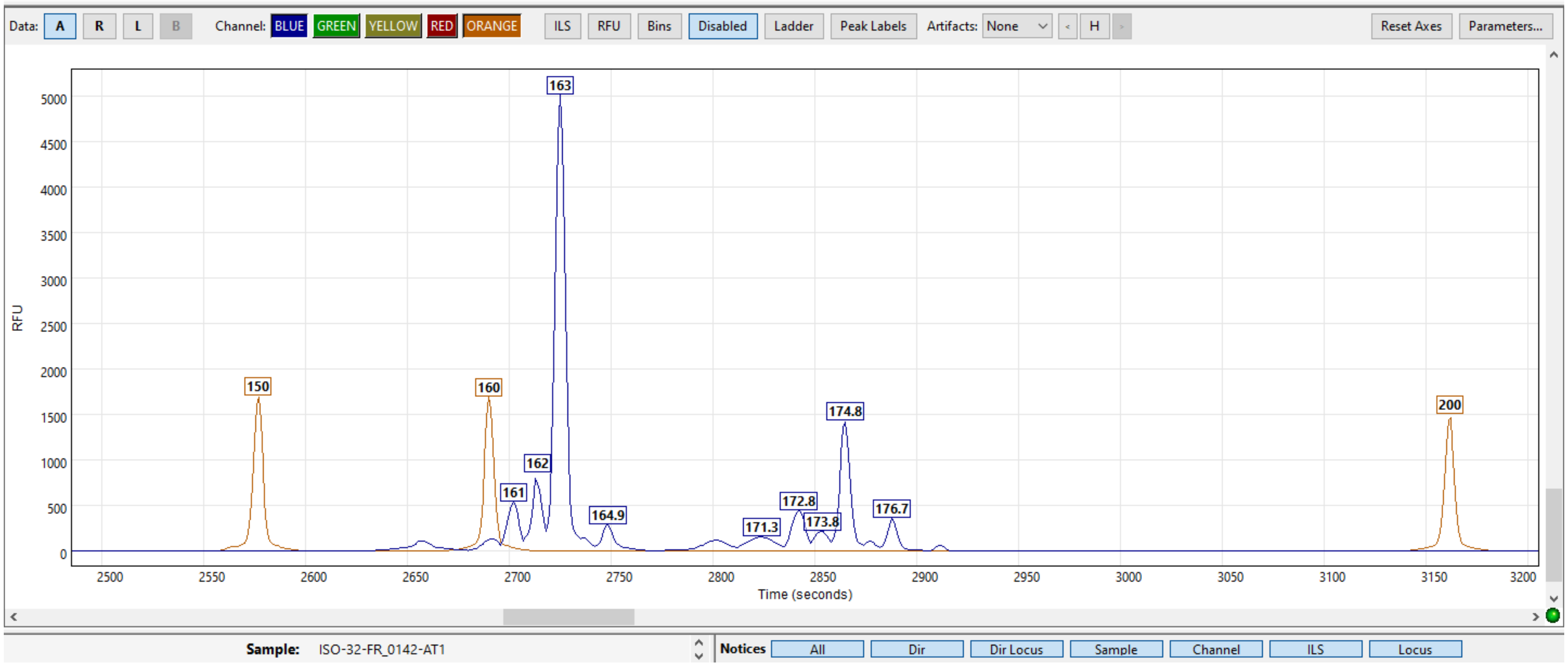


| | |
|---|---|
| **Observer 1** | **163;175** |
| **Observer 2** | **163;174.8** |
| **Observer 3** | **163;175** |

**30- Colony. Locus ISO AT1 sample 33 (0143)**

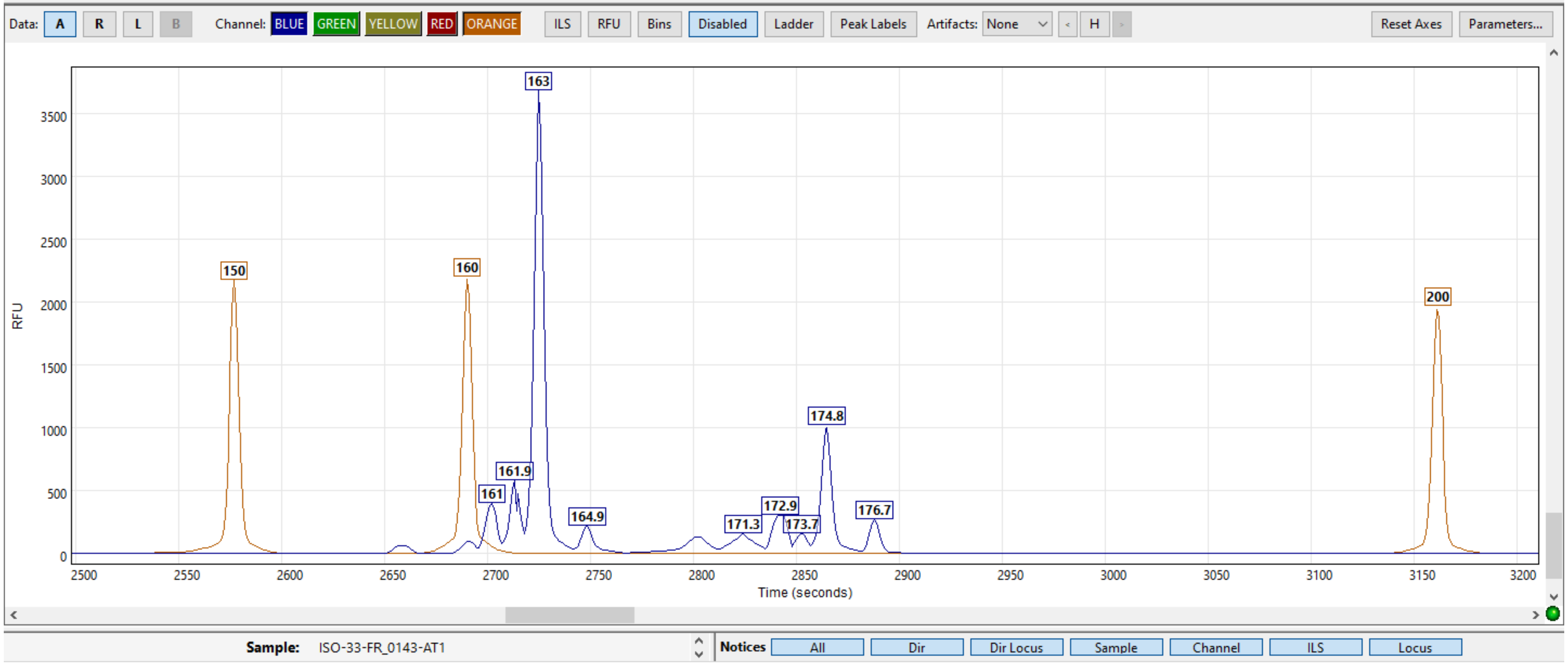


| | |
|---|---|
| **Observer 1** | **163;175** |
| **Observer 2** | **163;174.8** |
| **Observer 3** | **163;174.8** |

Fig S3. Calculation of Morphological Diversity of *Aedes aegypti* from clusters A sylvatic (higher) and Cluster B, non sylvatic (lower). Based on data from Agboli et al 2025, herein cited.

| | | Centroid Size | Individuals | Mean Centroid Size |
|---|---|---|---|---|
| RIGHT | Cluster A sylvatic | 250.66 | 10 | **25.07** |
| RIGHT | Cluster B non sylvatic | 837.19 | 55 | **15.22** |
| LEFT | Cluster A sylvatic | 268.73 | 10 | **26.87** |
| LEFT | Cluster B non sylvatic | 736.10 | 58 | **12.69** |

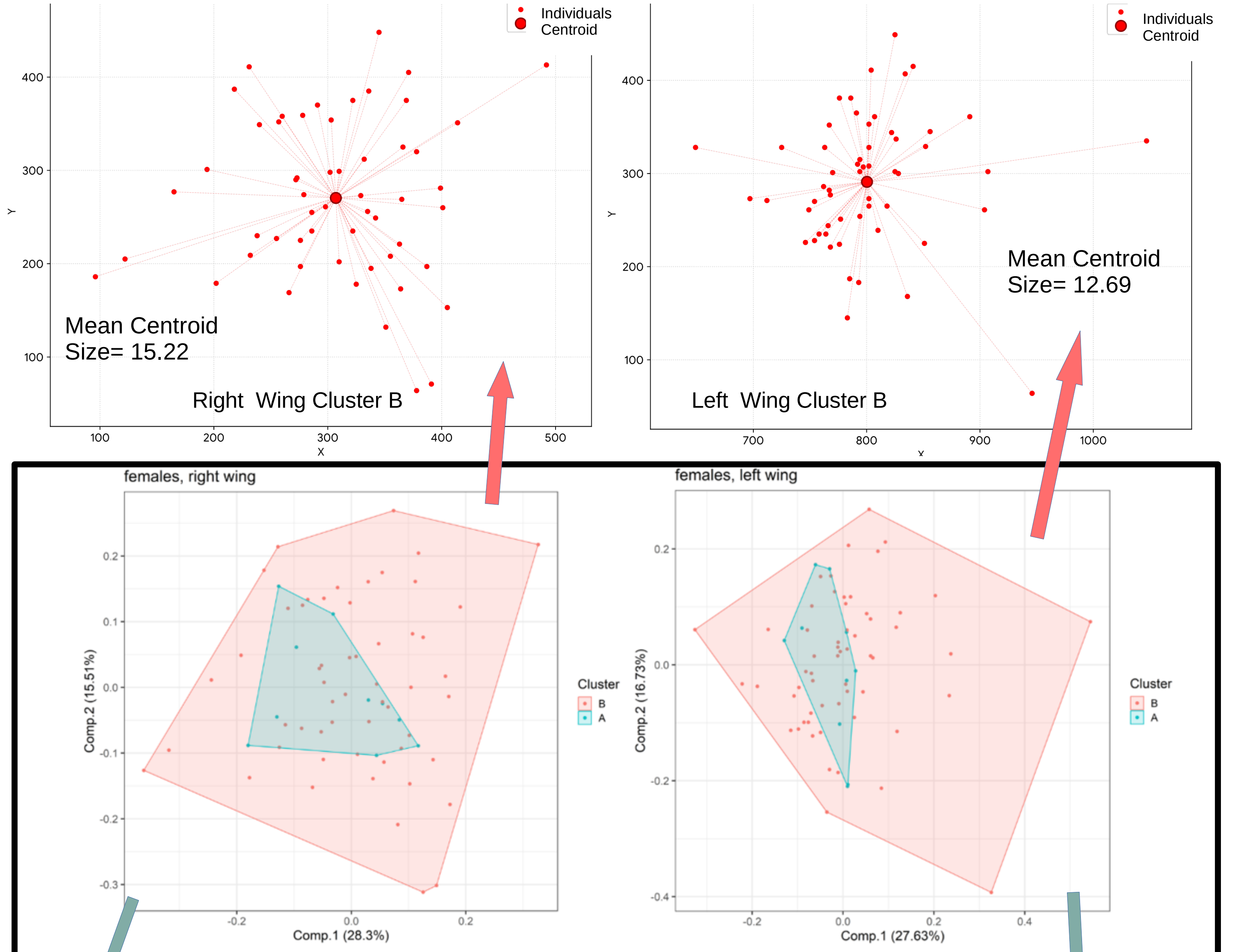


*Figure S1: PCA plots on the wing shape using the females belonging to the two identified genetic clusters (A and B).* ***Reproduced from:*** *Agboli, E., J. Z. B.Zahouli, F. G.Sauer, et al. 2025. "Morphological and Genetic Heterogeneity in Aedes aegypti (Diptera: Culicidae) Populations Across Diverse Landscapes in West Africa." Ecology and Evolution15, no. 12: e72748.* ***https://doi.org/10.1002/ece3.72748.***

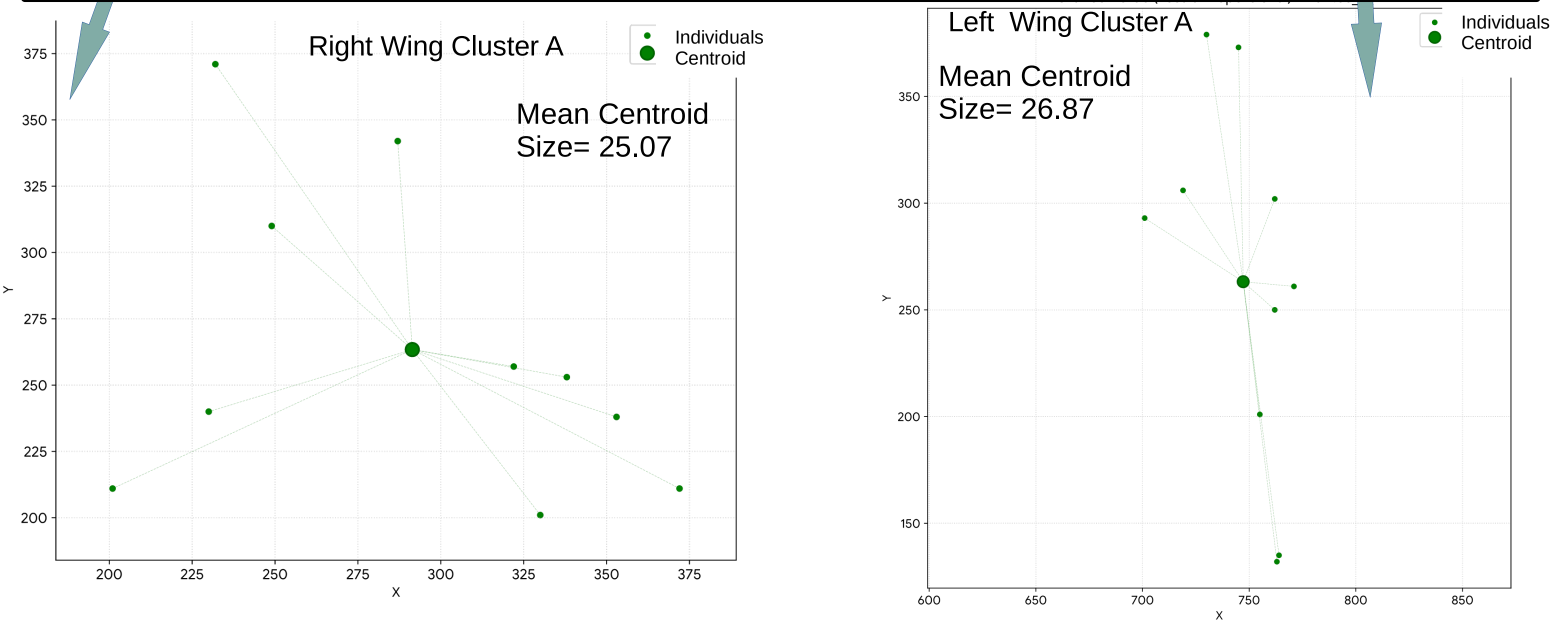

Supplement: Supplementary file 1 [file insects-17-00469-s001.zip › insects-4224215-supplementary.pdf]
